# Culturable mycota from Karst caves in Yunnan Province, China

**DOI:** 10.3897/imafungus.17.190343

**Published:** 2026-08-26

**Authors:** Xiang-Fu Liu, Saowaluck Tibpromma, K. W. Thilini Chethana, Li Lu, Kevin D. Hyde, Wen-Hua Lu, Rui-Fang Xu, Xue-Mei Chen, Xian Zhang, Ming-Xiu Chen, Xing-Juan Xiao, Chang-Lin Zhao, Tian-Ye Du, Dong-Qin Dai, Al-Bandari F. Al-Arjani, Abdallah M. Elgorban, Nakarin Suwannarach, Jaturong Kumla, Fu-Qiang Yu, Mei-Mei Wang, Samantha C. Karunarathna

**Affiliations:** 1 Center for Yunnan Plateau Biological Resources Protection and Utilization & Yunnan International Joint Laboratory of Fungal Sustainable Utilization in South and Southeast Asia, College of Biology and Food Engineering, Qujing Normal University, Qujing 655099, China Center for Yunnan Plateau Biological Resources Protection and Utilization & Yunnan International Joint Laboratory of Fungal Sustainable Utilization in South and Southeast Asia, College of Biology and Food Engineering, Qujing Normal University Qujing China; 2 Key Laboratory of Yunnan Provincial Department of Education of the Deep-Time Evolution on Biodiversity from the Origin of the Pearl River, Qujing Normal University, Qujing, Yunnan Province, 655011, China Key Laboratory of Yunnan Provincial Department of Education of the Deep-Time Evolution on Biodiversity from the Origin of the Pearl River, Qujing Normal University Qujing China; 3 School of Science, Mae Fah Luang University, Chiang Rai 57100, Thailand School of Science, Mae Fah Luang University Chiang Rai Thailand; 4 Center of Excellence in Fungal Research, Mae Fah Luang University, Chiang Rai 57100, Thailand Center of Excellence in Fungal Research, Mae Fah Luang University Chiang Rai Thailand; 5 College of Biological Science and Food Engineering, Southwest Forestry University, Kunming 650224, China College of Biological Science and Food Engineering, Southwest Forestry University Kunming China; 6 Modern Industry School of Edible Fungi, Southwest Forestry University, Kunming 650224, China Modern Industry School of Edible Fungi, Southwest Forestry University Kunming China; 7 Center of Excellence in Microbial Diversity and Sustainable Utilization, Chiang Mai University, Chiang Mai 50200, Thailand Center of Excellence in Microbial Diversity and Sustainable Utilization, Chiang Mai University Chiang Mai Thailand; 8 School of Food and Pharmaceutical Engineering, Guizhou Institute of Technology, Guiyang 550025, China School of Food and Pharmaceutical Engineering, Guizhou Institute of Technology Guiyang China; 9 Botany and Microbiology Department, College of Science, King Saud University, Riyadh 11451, Saudi Arabia Botany and Microbiology Department, College of Science, King Saud University Riyadh Saudi Arabia; 10 Center of Excellence in Biotechnology Research (CEBR), King Saud University, Riyadh, Saudi Arabia Center of Excellence in Biotechnology Research (CEBR), King Saud University Riyadh Saudi Arabia; 11 Department of Biology, Faculty of Science, Chiang Mai University, Chiang Mai 50200, Thailand Department of Biology, Faculty of Science, Chiang Mai University Chiang Mai Thailand; 12 Office of Research Administration, Chiang Mai University, Chiang Mai 50200, Thailand Office of Research Administration, Chiang Mai University Chiang Mai Thailand; 13 Yunnan Key Laboratory for Fungal Diversity and Green Development & Yunnan International Joint Laboratory of Fungal Sustainable Utilization in South and Southeast Asia, Germplasm Bank of Wild Species, Kunming Institute of Botany, Chinese Academy of Science, Kunming, China Yunnan Key Laboratory for Fungal Diversity and Green Development & Yunnan International Joint Laboratory of Fungal Sustainable Utilization in South and Southeast Asia, Germplasm Bank of Wild Species, Kunming Institute of Botany, Chinese Academy of Science Kunming China

**Keywords:** 12 novel taxa, *
Ascomycota
*, culturable fungi, fungi associated with caves, morphology, phylogeny

## Abstract

Caves, characterized by enclosed, unique, and stable environments, provide favorable conditions for fungal growth and harbor high levels of fungal diversity. Although Yunnan Province is home to more than 200 caves, studies on cave-associated fungi in this region remain scarce. To address this knowledge gap, we surveyed six accessible karst caves in the eastern, western, and southeastern Yunnan. A total of 34 fungal strains representing 17 species were isolated. Morphological characteristics and multi-gene phylogenetic analyses revealed taxonomic diversity among culturable fungi isolated from the sampled Yunnan caves, including numerous previously undescribed taxa. This study introduces one new genus (*Neoachaetomiella*), 12 novel species (*Aspergillus dehongensis*, *Cosmospora cavernicola*, *Neoachaetomiella cavernicola*, *Pithoascus cavernicola*, *Trichoderma dehongense*, *T. hongheense*, *T. mangshiense*, *T. neogongcheniae*, *T. neopyramidale*, *T. nujiangense*, *T. yingjiangense*, and *Trichocladium cavernicola*), and reports five species (*Aspergillus baeticus*, *Chaetomium subaffine*, *Daldinia eschscholtzii*, *Mucor phayaoensis*, and *Trichoderma obovatum*) as new records for caves in Yunnan, China. Detailed descriptions, illustrations, and phylogenetic analyses of all 17 taxa are provided. Additionally, a global checklist of cave-associated fungi is presented.

## Introduction

Caves are unique subterranean ecosystems characterized by generally exhibiting strong light limitation, relatively stable temperatures, high humidity, and limited nutrient availability (Kuzmina et al. 2012; Gabriel and Northup 2013; Liu et al. 2021; Zhang et al. 2021). These distinctive environmental conditions, shaped by karst geomorphology and subterranean hydrology, create isolated habitats that support the evolution of specialized microbial life (Kuzmina et al. 2012; Gabriel and Northup 2013; Paula et al. 2019; Zhang et al. 2021). The enclosed, isolated nature of cave systems significantly reduces external disturbances, resulting in ecological networks that harbor distinct biological communities. These communities make caves valuable natural laboratories for studies in ecology, microbiology, geology, and evolutionary biology (Ogórek et al. 2013; Ortiz et al. 2014; Zhang et al. 2021). Cave environments have garnered increasing attention for their role in harboring novel organisms and sustaining biodiversity within isolated and distinctive ecological niches (Gabriel and Northup 2013; Zhang et al. 2021). China is among the world’s leading karst regions, with carbonate rock covering approximately 3.44 million km^2^, and abundant cave resources, primarily concentrated in Yunnan, Guizhou, Guangxi, Hunan, Sichuan, Hubei, and Guangdong provinces (Li and Luo 1983; Yuan and Cheng 2002). To date, cave studies in China have primarily focused on flora, fauna, invertebrates, and ecosystem ecology, while investigations into microbes remain sparse (Wang and Cao 1998; Xu 2007; Zhao et al. 2010; Man 2017). Yunnan, which houses the country’s highest cave density, with over 200 documented (Zhang and Zhu 2012), has seen only a few microbial surveys. Karunarathna et al. (2020) described seven fungal species, including four novelties from bat carcasses in a Yunnan limestone cave, while Liu et al. (2021, 2025) reported two new species and a new host record from caves in eastern Yunnan. However, most caves in Yunnan lack microbial characterization, pointing to a substantial gap in karst cave microbiology that warrants future attention.

Early research in cave biology primarily focused on cave-dwelling fauna, with the limited records and reports concerning fungi (Zhang et al. 2017, 2021). A major turning point in cave mycology was the emergence of white-nose syndrome in North American bat populations, caused by *Pseudogymnoascus destructans* (Blehert and Gargas) Minnis & D.L. Lindner (Liu et al. 2021; Zhang et al. 2021). Although a few sporadic reports of cave-associated fungi existed prior to this outbreak, the appearance of white-nose syndrome in 2006 prompted systematic and extensive research into fungal diversity and pathogenicity in subterranean environments, leading to a surge in cave mycological studies (Blehert et al. 2009; Vanderwolf et al. 2013a, 2013b; Liu et al. 2021; Zhang et al. 2021). Since then, over 2,000 fungal species, representing more than 600 genera, have been documented from caves and mines worldwide, with *Ascomycota* identified as the most dominant phylum (Cunha et al. 2020; Liu et al. 2021).

Fungi play an indispensable role in cave ecosystems, contributing to organic matter decomposition, nutrient recycling, biomineralization, and serving as food sources or symbionts for cave fauna, and forming diverse ecological associations with other cave organisms (Northup and Lavoie 2001; Barton and Northup 2007; Nováková 2009; Li et al. 2015; Liu et al. 2021; Zhang et al. 2021). However, some fungi are also pathogenic and pose threats to native cave dwellers and even to human visitors (Savković et al. 2025). For example, *Pseudogymnoascus destructans* causes white-nose syndrome in bats (Vanderwolf et al. 2016; Sharma et al. 2019; Savković et al. 2025), resulting in significant declines in bat populations. Additionally, certain cave fungi may serve as sources of airborne pollutants, potentially impacting the respiratory health of tourists and cave staff (Liu et al. 2025; Savković et al. 2025). Moreover, many cave-derived fungi produce unique secondary metabolites with antimicrobial, enzymatic, or cytotoxic properties, highlighting their potential for pharmaceutical and biotechnological applications (Vanderwolf et al. 2013a; Belyagoubi et al. 2018; Tavares et al. 2018; Zhang et al. 2021). The long-term isolation and unique selective pressures of cave habitats often result in fungal taxa exhibiting high degrees of novelty and ecological specialization, making them essential subjects for taxonomic, ecological, and functional studies (Vanderwolf et al. 2013a).

Despite their ecological and applied significance, cave fungi remain globally understudied. Most documented cave mycobiota originate from Europe and North America, while Asia, particularly China, lags in systematic investigations (Vanderwolf et al. 2013a; Zhang et al. 2017, 2021; Liu et al. 2021). Notably, China boasts one of the world’s largest and most complex karst landscapes, particularly in southwestern provinces such as Yunnan and Guizhou, which are renowned for their high cave density and exceptionally rich biodiversity (Zhou et al. 2007; Zhang et al. 2017, 2021). Although Zhang et al. (2017, 2021) conducted systematic surveys across many caves in southwestern China, isolating thousands of fungal strains and discovering more than 50 new species from multiple phyla, these studies represent only a very small fraction of the caves in the Yunnan-Guizhou Plateau. Consequently, many caves in this region remain unexplored.

Currently, little is known about the taxonomy, diversity, and ecological functions of cave-associated fungi in Yunnan. In this study, we investigated six caves located in eastern, western, and southeastern Yunnan Province, China. We isolated and identified 12 novel species and five new records, thereby significantly contributing to the limited knowledge of cave ecosystems in the region. Detailed morphological descriptions of the new species are provided, accompanied by photo plates illustrating microscopic features and comparisons with closely related taxa. Phylogenetic analyses were conducted to determine the systematic placement of these newly described and known species. Additionally, we compiled a comprehensive global checklist of 2,240 cave-associated fungal species, serving as a valuable resource for future cave mycological studies.

## Materials and methods

### Sampling collection and isolation of cave-associated fungi

**Figure 1.**
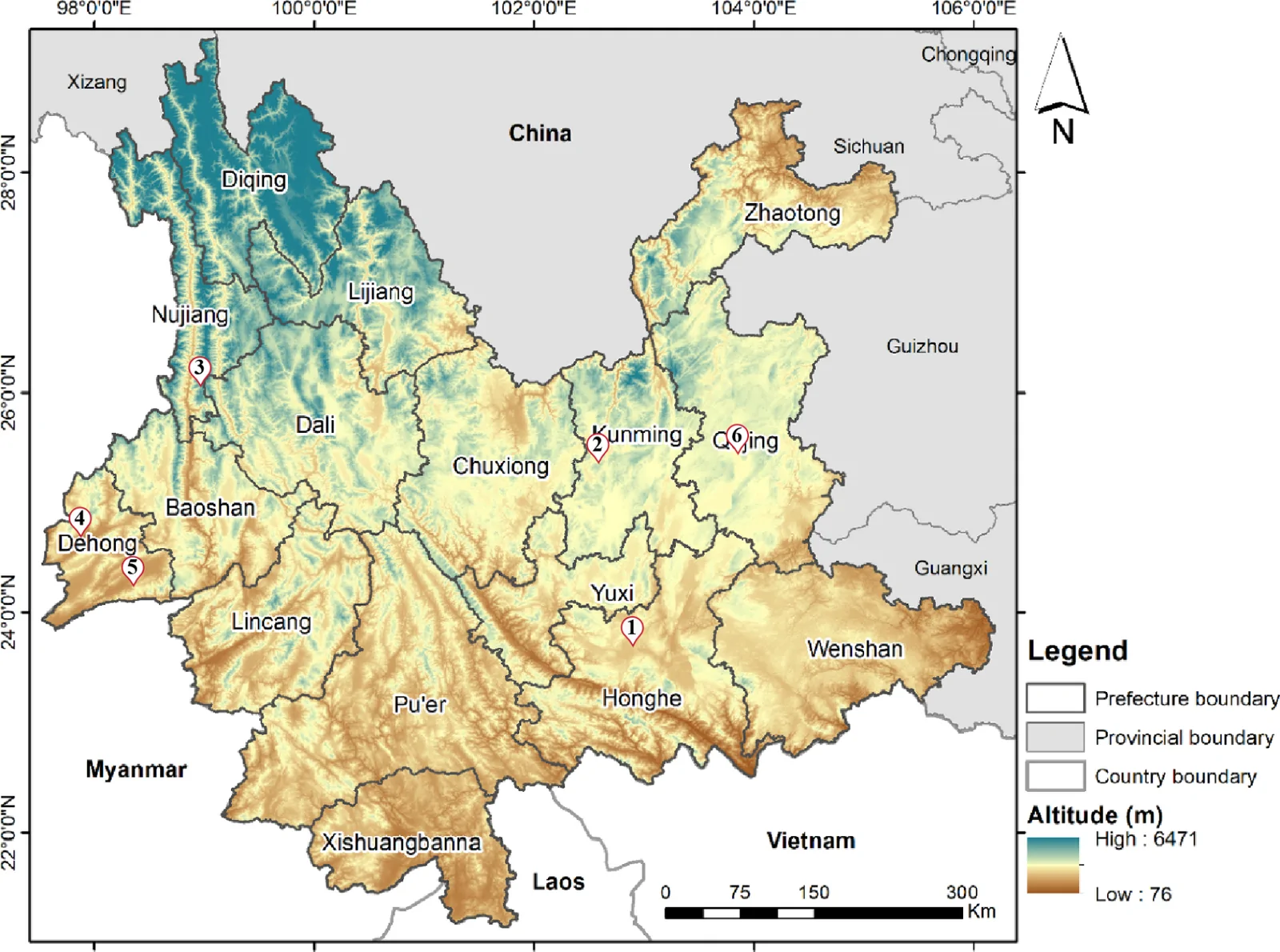
Sampling locations in Yunnan Province of China. Location names are denoted by numbers and full names (Table 1). Source: the original map was extracted from Lapuz et al. (2022).

**Figure 2.**
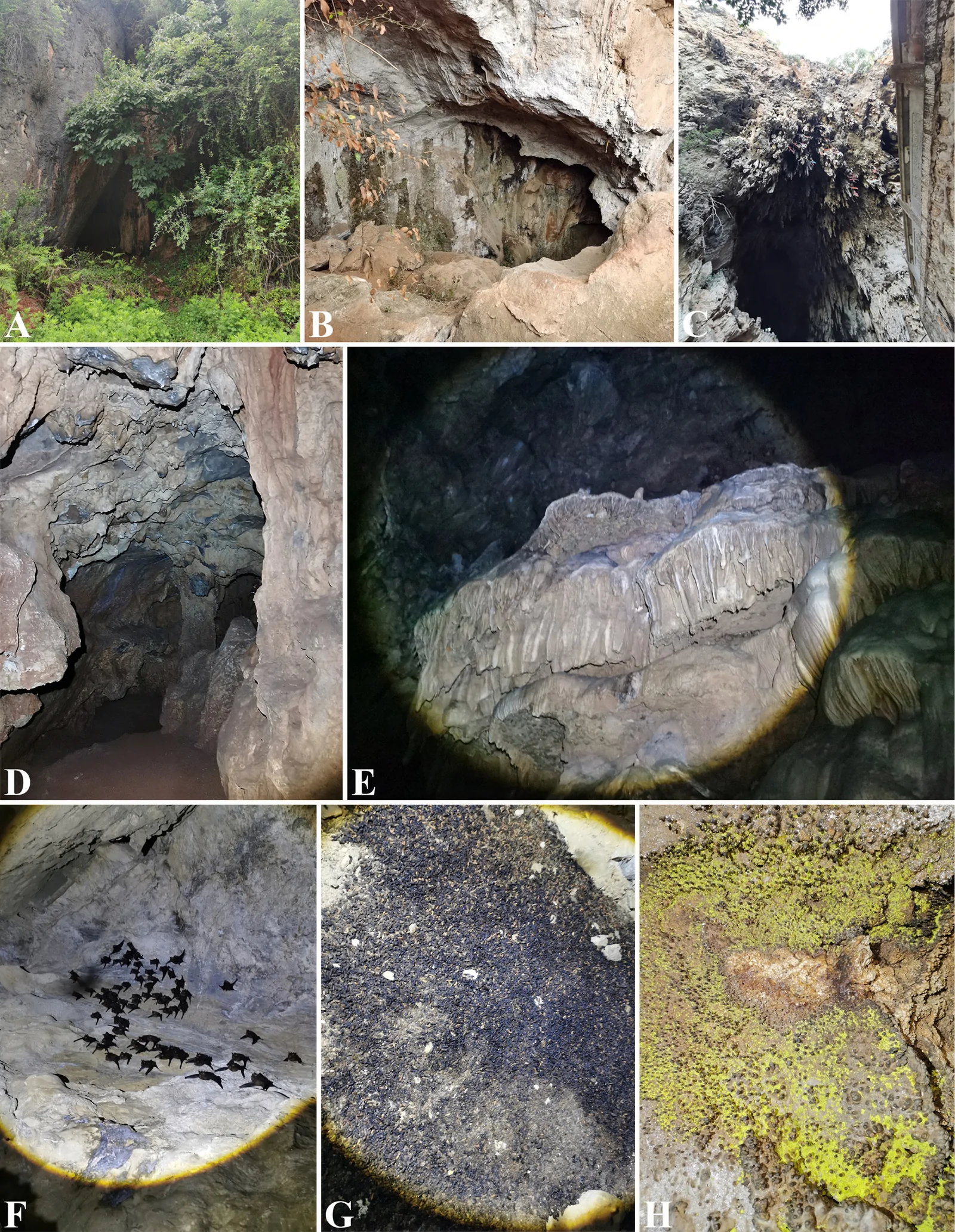
Scenes of visited caves. **A–C** Entrances to caves; **D, E** Rocks, stalactite; **F** Bats; **G** Bat guano; **H** Fungal community on rock surface.

Yunnan Province, situated at the heart of East Asia, encompasses a substantial portion of the world’s largest and most complex developing karst region (Zhou et al. 2007). In this study, six accessible caves located in the eastern, western, and southeastern regions of Yunnan Province, China, were selected for fungal sampling (Figs 1, 2; Table 1). Samples of air, rock, soil, twigs, feathers, and bat guano were collected from each cave using sterile tweezers and immediately placed into sterile 50 mL centrifuge tubes (BS-500-MJL, Beijing Labgic Technology Co., Ltd., P.R. China) after recording relevant collection information (Rathnayaka et al. 2024). Sampling sites were established at regular intervals from the cave entrance, with equal distances between adjacent sites, depending on the total length of each cave.

**Table 1.** Information on the sampled locations for cave fungi. Nu: Number; Colle. date: Collection date.

**Nu**.	**Name**	**Location (Yunnan Province)**	**Latitude, Longitude**	**Altitude (m)**	**Colle. date**
1	Swallow Cave	Miandian Town, Jianshui County, Honghe	23.636843°N, 103.053584°E	1343	24/12/2020
2	Swallow Cave	Chijiu Town, Fumin County, Kunming	25.388333°N, 102.537778°E	1864.03	7/5/2021
3	Walaya Cave	Daxingdi Town, Lushui City, Nujiang	26.080743°N, 98.848391°E	850.46	21/4/2024
4	Xianren Cave	Nongchang Town, Yingjiang County, Dehong	24.524816°N, 97.801635°E	816.49	22/4/2024
5	Sanxian Cave	Mengga Town, Mangshi City, Dehong	24.232220°N, 98.426103°E	1530.89	23/4/2024
6	Longyangxian Cave	Longyang County, Qujing	25.434721°N, 103.509524°E	1924.77	29/4/2024

All collected samples were transported to the mycology laboratory at Qujing Normal University and stored at 4 °C prior to fungal isolation. Fungal taxa were isolated using a serial dilution plating method with potato dextrose agar (PDA) and malt agar (MA) supplemented with amoxicillin (50 μg/mL) using sterile cotton swabs. Each sample was plated in triplicate. The PDA and MA plates were incubated at room temperature until single fungal colonies developed. Individual fungal colonies were then subcultured onto fresh PDA plates in triplicate and incubated at room temperature (25 ± 2 °C). Dry cultures (containing fungal mass) were deposited in the herbarium of Guizhou Medical University, Guiyang, China (GMB), and the Herbarium of the Kunming Institute of Botany, Chinese Academy of Sciences, Kunming, China (HKAS). Living cultures were deposited in the Guizhou Medical University Culture Collection (GMBCC) and the Guizhou Culture Collection (GZCC), Guiyang, China. Index Fungorum numbers were registered as outlined in Index Fungorum (https://www.indexfungorum.org). Novel fungal species were introduced in accordance with the guidelines of Chethana et al. (2021).

### Morphological observations

Morphological characteristics of the cultures sporulated on PDA and MA after 20–60 days at room temperature (25 ± 2 °C) were examined. Morphological observations were performed using an Olympus SZ61 (Japan) series stereomicroscope and photographed using an Olympus SZ2-ILST imaging system coupled with an Industrial Digital Camera 16NP USB 3.0 (Panasonic, Japan). Fungal structures were measured using the Tarosoft Image Framework (IFW) program, version 0.97, and the images used for figures were processed in Adobe Photoshop CS6 (Adobe Systems, USA).

### DNA extraction, PCR amplifications, and sequencing

Fresh mycelia grown on PDA were scraped from living cultures incubated at room temperature (25 ± 2 °C) for 2–4 weeks. BioSpain Fungus Genomic DNA Extraction Kit-BSC14S1 (BioFlux, P.R. China) was used to extract genomic DNA according to the manufacturer’s protocol. Genomic DNA was stored at -20 °C until use. The polymerase chain reactions (PCR) were performed for the genes using the primers shown in Table 2. The PCR amplifications were carried out in a 25 µL reaction volume, containing 12.5 µL of 2 × Power Taq PCR Master Mix (Beijing Bomaide Biotechnology Co., Ltd., Haidian District, Beijing, China), 8.5 µL distilled-deionized water (ddH_2_O), 2 µL of DNA template, and 1 µL of each forward and reverse primers (Tibpromma et al. 2018). The PCR products were detected by electrophoresis on 1% agarose gels stained with TS-GelRed (TSJ002, Beijing Kinco Biotechnology Co., Ltd., Kunming Branch, P.R. China). The purification and sequencing of PCR products were performed by Tsingke Biological Engineering Technology and Services Co., Ltd (Yunnan, P.R. China).

**Table 2.** Details of genes/loci with PCR primers used in each fungal genus.

**Genera name**	**Genes/loci**	**Primers (forward/reverse)**	**References**
* Aspergillus *	ITS	ITS5/ITS4	White et al. (1990)
	*BenA*	Bt-2a/Bt-2b	Glass and Donaldson (1995)
	*CaM*	Cmd5/Cmd6	Hong et al. (2006)
	*rpb*2	RPB2-5f/RPB2-7cR	Liu et al. (1999)
* Chaetomium *	ITS	ITS5/ITS4	White et al. (1990)
	LSU	LR0R/LR5	Vilgalys and Hester (1990)
	*rpb*2	RPB2-5f/RPB2-7cR	Liu et al. (1999)
	*tub*2	T1/T22	O’Donnell and Cigelnik (1997), Glass and Donaldson (1995)
* Cosmospora *	ITS	ITS1/ITS4	White et al. (1990)
	LSU	LR0R/LR5	Vilgalys and Hester (1990)
	*rpb*2	RPB2-5f/RPB2-7cR	Liu et al. (1999)
	*tub*2	Bt-2a/Bt-2b	Glass and Donaldson (1995)
* Daldinia *	ITS	ITS5/ITS4	White et al. (1990)
	LSU	LR0R/LR7	Vilgalys and Hester (1990)
	*rpb*2	RPB2-5f/RPB2-7cR	Liu et al. (1999)
	*tub*2	T1/T22	O’Donnell and Cigelnik (1997), Glass and Donaldson (1995)
* Mucor *	ITS	ITS5/ITS4	White et al. (1990)
	LSU	LR0R/LR7	Vilgalys and Hester (1990)
* Neoachaetomiella *	ITS	ITS1/ITS4	White et al. (1990)
	*rpb*2	fRPB2-5f/fRPB2-7cR	Liu et al. (1999)
	*tub2*	T1/T22	O’Donnell and Cigelnik (1997), Glass and Donaldson (1995)
* Pithoascus *	ITS	ITS5/ITS4	White et al. (1990)
	LSU	LR0R/LR5	Vilgalys and Hester (1990)
	*tef*1-α	EF1-983F/EF1-2218R	Rehner and Buckley (2005)
	*tub*2	Bt-2a/Bt-2b	Glass and Donaldson (1995)
* Trichocladium *	ITS	ITS5/ITS4	White et al. (1990)
	*rpb*2	fRPB2-5f/fRPB2-7cR	Liu et al. (1999)
	*tub*2	T1/T22	O’Donnell and Cigelnik (1997), Glass and Donaldson (1995)
* Trichoderma *	ITS	ITS1/ITS4	White et al. (1990)
	*rpb*2	fRPB2-5f/fRPB2-7cR	Liu et al. (1999)
	*tef*1-α	EF1/EF2	O’Donnell et al. (1998)

### Phylogenetic analyses

Phylogenetic analyses were conducted as described by Dissanayake et al. (2020). Newly generated sequences were initially subjected to a BLASTn search in NCBI to identify the most probable closely related taxa available in GenBank (http://blast.ncbi.nlm.nih.gov/). Reference sequences were retrieved from GenBank (https://www.ncbi.nlm.nih.gov/nuccore/) based on recent publications. Raw sequences were initially checked with BioEdit V.7.0.5.2 (Hall 1999). Forward and reverse sequences were assembled with SeqMan v. 7.0.0 (DNASTAR, Madison, WI, USA). Each gene dataset was aligned with MAFFT v7.215 (https://mafft.cbrc.jp/alignment/server/; Katoh and Standley 2016) and trimmed with Trimal.v1.2rev59 (Capella-Gutiérrez et al. 2009) and edited manually where necessary in BioEdit V.7.0.5.2 (Hall 1999). Multi-locus datasets were concatenated using SequenceMatrix 1.7.8 (Vaidya et al. 2011), AliView (Larsson 2014), or BioEdit V.7.0.5.2 (Hall 1999). The final FASTA file formats were converted to PHYLIP (for Maximum Likelihood analysis) and NEXUS (for Bayesian Inference analysis) formats using the online Alignment Transformation Environment (ALTER) tool (http://sing.ei.uvigo.es/ALTER/) (Glez-Peña et al. 2010).

CIPRES Science Gateway platform was used to carry out the Randomized Axelerated Maximum Likelihood (ML) and Bayesian Inference analyses (BI) (Miller et al. 2010). The RAxML analyses were performed using RAxML-HPC2 on XSEDE (8.2.12) (Stamatakis et al. 2008; Stamatakis 2006, 2014) under the GTRGAMMA nucleotide substitution model, with 1,000 bootstrap replicates. BI analyses were conducted using MrBayes on XSEDE (version 3.2.7a) (Ronquist et al. 2012) employing a Markov Chain Monte Carlo (MCMC) approach to evaluate Bayesian posterior probabilities (BYPP) (Richard and Lippmann 1991; Rannala and Yang 1996; Zhaxybayeva and Gogarten 2002). The optimal nucleotide substitution model for each partition was estimated using MrModeltest v. 2.3 (Nylander 2004). Six simultaneous Markov chains were run for 2,000,000 to 8,000,000 generations (depending on the fungal group), with trees sampled at every 100^th^ generation. The phylogenetic tree was visualized using FigTree v.1.4.3 (http://tree.bio.ed.ac.uk/software/figtree/) and edited in Microsoft PowerPoint 2021 and Adobe Photoshop CS6 (Adobe Systems, California, USA). All newly generated sequences in this study were deposited in GenBank, and the accession numbers are listed in Suppl. material 1: table S1.

### Genealogical concordance phylogenetic species recognition (GCPSR) analysis

The GCPSR analysis was performed using the pairwise homoplasy index (PHI) test to assess species limits and detect significant recombination events (Quaedvlieg et al. 2014). The PHI test was implemented in SplitsTree4 V4.17.1 (Bruen et al. 2006; Huson and Bryant 2006; Quaedvlieg et al. 2014) on multi-locus and single-locus datasets including closely related species. A PHI test result below 0.05 (Φw < 0.05) indicates significant recombination within the dataset. To visualize relationships among closely related taxa, split graphs were constructed from concatenated datasets using the LogDet transformation and split decomposition options, and subsequently edited in Microsoft PowerPoint 2019.

### Checklist of cave-associated fungi

The taxonomic status of cave-associated fungi obtained in this study has been determined based on morphology and multi-gene phylogeny. The information on the distribution and cave substrates of taxa recovered here, as well as those reported previously, was compiled from the literature. Current names were verified and updated using Index Fungorum (https://wwwindexfungorumorg/Names/Namesasp; accessed 16 January 2026). The checklist was assembled from searches of online databases and search engines (Web of Science, Google, and Baidu), as well as books, graduation theses, and research articles published since Vanderwolf et al. (2013a).

## Results

### Taxonomy

***Ascomycota* Caval.-Sm**.

***Eurotiomycetes* O.E. Erikss. & Winka**

***Eurotiales* G.W. Martin ex Benny & Kimbr., Mycotaxon 12(1): 23 (1980)**

#### Aspergillaceae

Taxon classificationAnimaliaEurotialesAspergillaceae

Link, Abh. K. Akad. Wiss. Berlin: 165 (1826)

C6E89813-B606-5699-A359-AC3E687987C8

##### Notes.

*Aspergillaceae* was established by Link (1826), with *Aspergillus* P. Micheli ex Haller designated as the type genus. Houbraken and Samson (2011) reinstated this family based on multi-locus phylogeny and recognized seven distinct clades. Some species in this family can tolerate extreme conditions, including high sugar and salt concentrations, low or high temperatures, low acidity, and low oxygen levels (Houbraken et al. 2014; Zhang et al. 2021). *Aspergillaceae* species are predominantly saprobic and commonly occur in soil, although some are plant and animal pathogens (Houbraken et al. 2014). Many species in this family are of great economic importance and are widely used in food fermentation, biotechnology, and biomedicine (Mogensen et al. 2009; Perrone et al. 2011; Houbraken et al. 2014). Morphologically, *Aspergillaceae* members are characterized by asci within cleistothecia or stromata, or enclosed by Hülle cells, and typically have oblate to ellipsoidal ascospores with a distinct furrow or slit (Houbraken and Samson 2011; Houbraken et al. 2014). Conidia are mostly formed on lanceolate, flask-shaped, or cylindrical phialides (Houbraken et al. 2014). *Aspergillaceae* is the most abundant family reported from cave environments, with 13 genera comprising over 315 species worldwide (Suppl. material 1: table S2).

#### Aspergillus

Taxon classificationAnimaliaEurotialesAspergillaceae

P. Micheli ex Haller, Hist. stirp. Helv. (Bernae) 3: 113 (1768)

C6C2CB1C-C92E-592F-A33F-1B2E453610E7

##### Notes.

The genus *Aspergillus* was established by Haller (1768), with *A. glaucus* (L.) Link as the type species. It is one of the most economically important and widely distributed fungal genera (Wijayawardene et al. 2017; Houbraken et al. 2020; Zhang et al. 2021). Currently comprising 428 accepted species (Sklenář et al. 2021; Wijayawardene et al. 2022), this taxonomically diverse genus is classified into six subgenera (*Aspergillus*, *Cremei*, *Circumdati*, *Fumigati*, *Nidulantes*, and *Polypaecilum*), 27 sections, and 75 series according to the latest revision (Houbraken et al. 2020; Sklenář et al. 2021). Members of *Aspergillus* are characterized by their distinctive aspergillum-like conidiophores, consisting of a foot cell, an aseptate stipe, and a terminal vesicle (Houbraken et al. 2020). Most species are terrestrial saprobes, though some occur as aquatic fungi or as opportunistic pathogens (Peterson 2008; Sigler et al. 2010; Varga et al. 2011; Guarro et al. 2012; Samson et al. 2014; Jurjević et al. 2015; Arzanlou et al. 2016; Visagie et al. 2017; Wijayawardene et al. 2017). They are commonly found in soil, plants, and food, with some species posing significant threats as producers of dangerous mycotoxins (aflatoxins and ochratoxins), and others causing aspergillosis in humans, animals, and plants (Peterson 2008; Samson et al. 2014; Al-Bedak and Faysal 2024). *Aspergillus* is the second most common fungal genus reported from caves, with more than 122 species worldwide, many of which are new cave isolates (Suppl. material 1: table S2). In this study, we describe a new species, *Aspergillus dehongensis*, and report a new geographical record for *A. baeticus* A. Nováková & Hubka.

### Phylogenetic analyses: *Aspergillus*

*Aspergillus* phylogeny was based on the combined ITS, *BenA*, *CaM*, and *rpb*2 sequence data. The combined alignment encompassed 75 strains, representing 32 taxa in *Aspergillus* section *Usti*, rooted with *A. ochraceoroseus* (CBS 550.77) and *A. funiculosus* (NRRL 4744). The tree topologies from the two analyses (ML and BI) were identical. *Aspergillus dehongensis* formed a distinct clade sister to *A. heterothallicus* Kwon-Chung, Fennell & Raper and *A. puniceus* Kwon-Chung & Fennell with high statistical support (ML/BI = 98/1.00), and the strain GMBCC2264 clustered together with *A. baeticus* (CMF 2181, NRRL 62487, and NRRL 62501 (ex-type)). Phylogenetic analysis of *Aspergillus* section *Usti* followed the methods of Samson et al. (2011) and Sun et al. (2020).

#### Aspergillus baeticus

Taxon classificationAnimaliaEurotialesAspergillaceae

A. Nováková & Hubka, in Nováková, Hubka, Sáiz-Jiménez & Kolařík, Int. J. Syst. Evol. Microbiol. 62(11): 2783 (2012)

CF4867C6-51EF-568E-9B2E-3BFE26A32AF2

Index Fungorum: IF564188

Fig. 4

##### Description.

Associated with the rock surface in the cave environment. Asexual morph on PDA: Conidial heads radiate, splitting into three or more columns. ***Conidiophores*** uniseriate, light brown with a darker brown upper part. ***Stipes*** 150–300 × 4–9 μm (x̄ = 246.88 × 7.44 μm), hyaline to yellow brown, septate. ***Vesicles*** 7–28 μm wide, globose to spathulate, smooth. ***Metulae*** 8–15 × 5–10 μm (x̄ = 10.18 × 8.38 μm, n = 30), hyaline to pale brown, uni- to biseriate. ***Phialides*** 6–13 × 1–3 μm (x̄ = 9.31 × 2.54 μm, n = 30), ampulliform, with minute collarette. ***Conidia*** 3–5 × 2–5 μm (x̄ = 3.67 × 3.44 μm, n = 60), globose to subglobose, ornamented with warts or spines, hyaline to yellow brown, arranged in chains. Sexual morph: Undetermined.

##### Culture characteristics.

Colonies on PDA attaining 20–40 mm after 15 days at room temperature (25 ± 2 °C), colonies at the surface, floccose, wrinkled, with mycelial areas white to cream, greenish-gray to green; reverse white to yellowish gray. Sporulation moderately dense on PDA after 60 days.

##### Substrata.

Cave sediment (Nováková et al. 2012), rock surface (this study).

##### Distribution.

Brazil (GBIF 2025), China (this study), India (GBIF 2025), Madagascar (GBIF 2025), Rwanda (GBIF 2025), South Africa (GBIF 2025), Spain (Nováková et al. 2012), Tanzania (GBIF 2025).

##### Material examined.

CHINA • Yunnan Province, Nujiang Lisu Autonomous Prefecture, Lushui City, Daxingdi Town, Walaya Cave, rock surface, 26.080743°N, 98.848391°E, elev. ca 850.46 m, 23 April 2024, Xiangfu Liu and Xuemei Chen, NJ3D (GMB-W1249), living culture GMBCC2264.

##### Notes.

**Figure 3.**
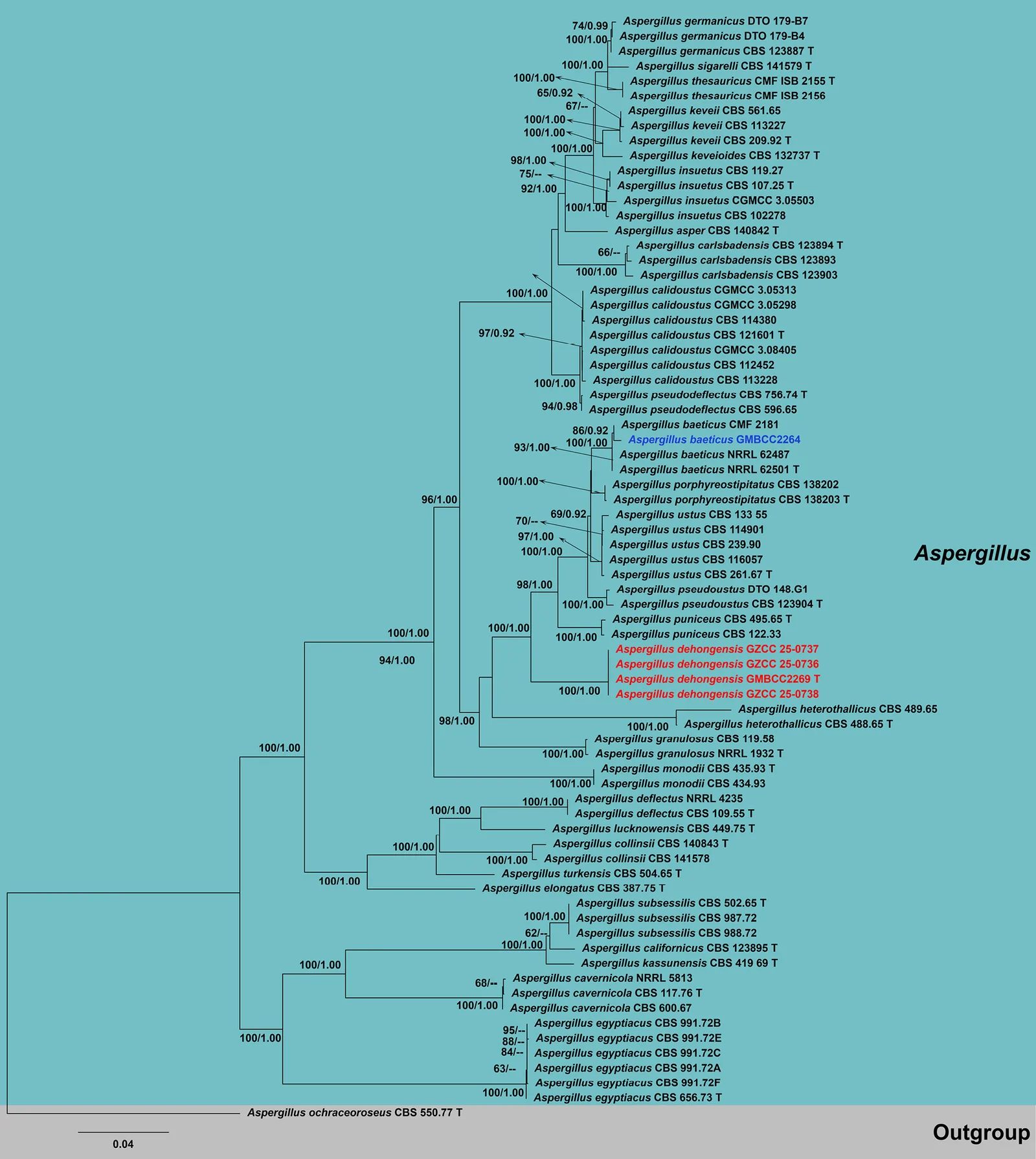
Consensus phylogram of 1,000 trees resulting from a RAxML analysis of the (ITS, *BenA*, *CaM*, and *rpb*2) alignment of the analyzed *Aspergillus* section *Usti* sequences. The RAxML analysis of the combined dataset yielded the best-scoring tree, with a final ML optimization likelihood value of -20276.030668. This tree was generated from 602, 595, 575, and 1,008 base pairs of ITS, *BenA*, *CaM*, and *rpb*2 characters, respectively. The matrix comprises 813 distinct alignment patterns, with 24.90% of the sequences containing gaps and indeterminate traits. Base frequencies were estimated as follows A = 0.234792, C = 0.272713, G = 0.265844, T = 0.226651; substitution rates AC = 1.106181, AG = 4.456639, AT = 1.721038, CG = 0.962803, CT = 7.239247, GT = 1.000000; proportion of invariable sites I = 0.551494; gamma distribution shape parameter α = 1.467907. The trees from both analyses (ML and BI) showed identical topologies. RAxML bootstrap support values (ML equal to or above 70%) and Bayesian inference (BYPP equal to or above 0.90) are given at the nodes (ML/BYPP). Newly generated sequences in this study are in blue and red. Type strains are denoted with ^T^. The scale bar represents the expected number of changes per site.

*Aspergillus baeticus* was introduced by Nováková et al. (2012) from cave sediment in Spain. Our isolate (GMBCC2264) clustered with *A. baeticus* (NRRL 62501 (type), NRRL 62487, and CMF 2181) within the section *Usti* with 89% ML and 0.97 BYPP support (Fig. 3). The nucleotide comparison between our strain (GMBCC2264) and *A. baeticus* (NRRL 62501, type) showed 1.34% (7/524 bp, 3 gaps) differences in *BenA*, 1.07% (5/469 bp, 1 gap) differences in *CAM*, and 1.31% (12/914 bp, without gap) differences in *rpb*2. Morphologically, the new isolate (GMBCC2264) is similar to *A. baeticus*, with dark brown conidiophores on the upper part and globose to subglobose conidia ornamented with warts or spines (Nováková et al. 2012). Based on phylogenetic and morphological analyses, the new isolate (GMBCC2264) is identified as *A. baeticus*. This is the first report of *A. baeticus* isolated from cave environments in China. In addition, this species is known exclusively from cave environments and is reported here for the first time from China.

**Figure 4.**
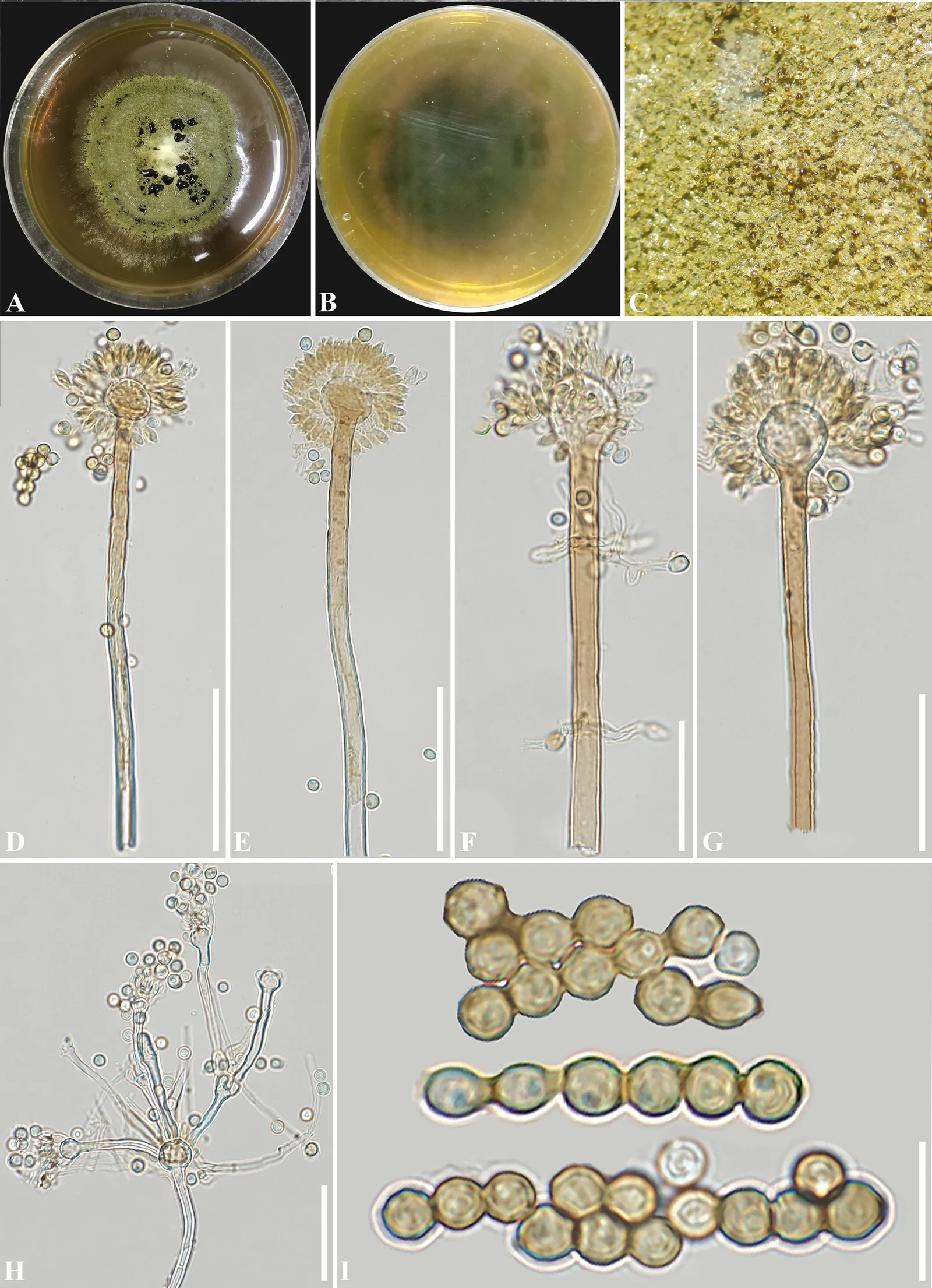
*Aspergillus baeticus* (GMBCC2264, new geographical record). **A, B** Colony on PDA (above and below); **C** Sporulation on PDA (30 days old culture); **D, H** Conidiophore with conidium head and conidia; **I** Conidia; Scale bars: 50 μm (**D, E**); 30 μm (**F–H**); 10 μm (**I**).

#### Aspergillus dehongensis

Taxon classificationAnimaliaEurotialesAspergillaceae

X.F. Liu, Karun., Tibpromma & K.D. Hyde sp. nov.

5934A62C-0DDC-502D-B6B9-77185147FBD1

Index Fungorum: IF905012

Fig. 5

##### Etymology.

Named after the type locality, Dehong.

##### Holotype.

GMB-W1540.

##### Description.

Associated with bamboo sticks, candles, and bat guano in the cave environment. Asexual morph on PDA: Conidial heads radiate, splitting into 3 or more columns. ***Conidiophores*** uniseriate, branched at apex. *Stipes* 200–450 × 3–8 μm (x̄ = 365.67 × 5.43), hyaline, septate. *Vesicles* 7–28 μm wide, globose to spathulate, smooth-walled. ***Metulae*** 6–21 × 3–6 μm (x̄ = 9.78 × 4.38 μm, n = 30), hyaline to pale brown, uni- to biseriate. ***Phialides*** 6–16 × 1–3 μm (x̄ = 9.31 × 2.54 μm, n = 30), ampulliform, with minute collarette. ***Conidia*** 3–7 × 2–6 μm (x̄ = 4.67 × 3.44 μm, n = 60), broadly ellipsoid to globose, yellowish-green, smooth-walled, hyaline, arranged in chains. Sexual morph: Undetermined.

##### Culture characteristics.

Colonies on PDA attaining 20–40 mm after 15 days at room temperature (25 ± 2 °C), colonies at surface, floccose, wrinkled, with mycelial areas white to cream, greenish-gray to yellowish-gray; reverse white to brown. Sporulation moderately dense on PDA after 20 days.

##### Material examined.

CHINA • Yunnan Province, Dehong Dai and Jingpo Autonomous Prefecture, Yingjiang County, Nongzhang Town, Xainren Cave, on the candle, 24.524816°N, 97.801635°E, elev. ca 816.49 m, 22 April 2024, Xiangfu Liu and Xuemei Chen, YJ33 (GMB-W1540, holotype), ex-type GMBCC2269, *ibid*., YJ33C, living culture GZCC 25-0737; *ibid*., bamboo stick, YJ35C2, living culture GZCC 25-0738; *ibid*., bat guano, YJ11C, living culture GZCC 25-0736.

**Figure 5.**
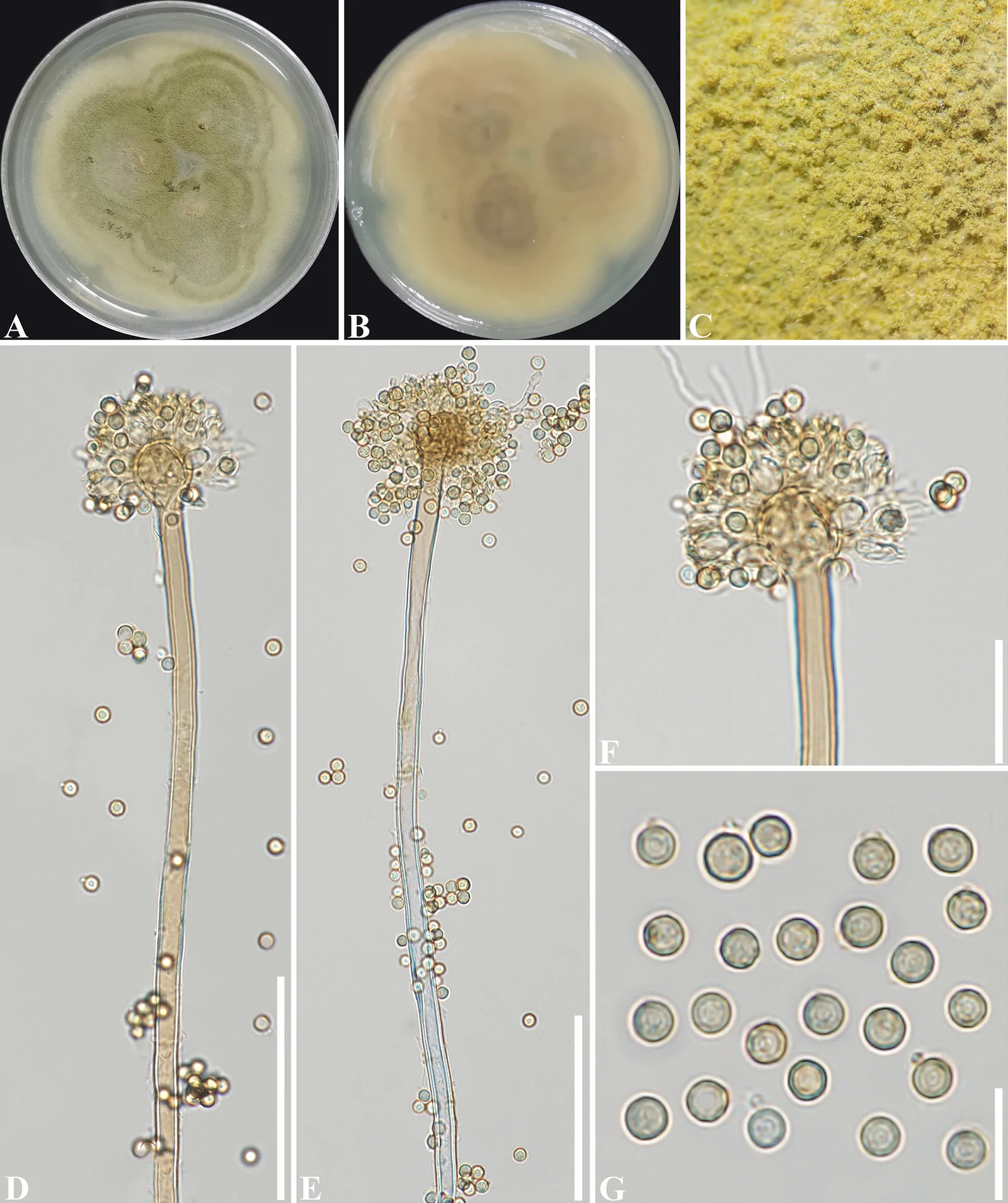
*Aspergillus dehongensis* (GMBCC2269, ex-ype). **A, B** Colony on PDA (above and below); **C** Sporulation on PDA (30 days old culture); **D, F** Conidiophore with a conidium head and conidia; **G** Conidia. Scale bars: 50 μm (**D, E**); 20 μm (**F**); 10 μm (**G**).

##### Notes.

**Figure 6.**
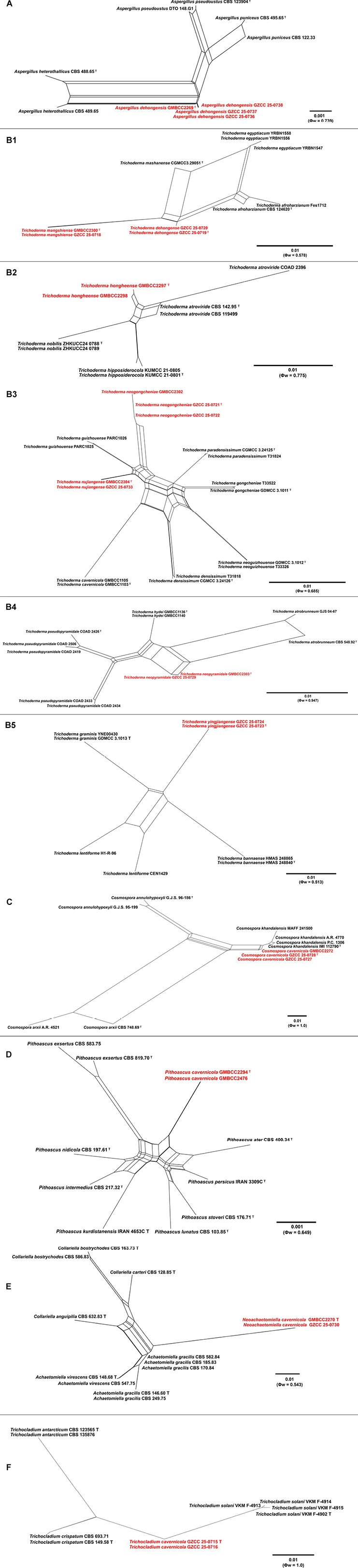
Split graphs showing the results of the pairwise homoplasy index (PHI) test of the new taxa and closely related taxa using LogDet transformation and splits decomposition. **A***Aspergillus dehongensis*; **B1***Trichoderma dehongense* and *T. mangshiense*; **B2***T. hongheense*; **B3***T. neogongcheniae* and *T. nujiangense*; **B4***T. neopyramidale*; B5 *T. yingjiangense*; **C***Cosmospora cavernicola*; **D***Pithoascus cavernicola*; **E***Neoachaetomiella cavernicola*; **F***Trichocladium cavernicola*. PHI test result P‐value (Φw) ≤ 0.05 indicates that there is significant recombination between the isolates included in the alignment. The new taxa are in red, “^T^” indicates the type species, and each PHI test value and scale bars are given in the bottom right corner.

In our phylogenetic analyses of ITS, *BenA*, *CaM*, and *rpb*2, *Aspergillus dehongensis* (GMBCC2269 (type), GZCC 25-0736, GZCC 25-0737, and GZCC 25-0738) formed a distinct lineage sister to *A. heterothallicus* (CBS 488.65 (type) and CBS 489.65) and *A. puniceus* (CBS 122.33 and CBS 495.65 (type)) with 100% ML and 1.00 PP support in the *Aspergillus* section *Usti* (Fig. 3). However, the nucleotide comparisons between *Aspergillus dehongensis* (GMBCC2269, type) and *A. heterothallicus* (CBS 488.65, type) revealed 10.30% (64/427 bp, with 3 gaps) differences in *BenA*, and 9.97% (109/1011 bp, without gap) base pair differences in *rpb*2. *Aspergillus dehongensis* (GMBCC2269, type) and *A. puniceus* (CBS 495.65, type) revealed 8.41% (36/428 bp, with 6 gaps) differences in *BenA*, and 6.43% (65/1011 bp, without gaps) base pair differences in *rpb*2. Morphologically, *Aspergillus dehongensis* differs from *A. heterothallicus* in that the latter has aperturate echinulata, smaller conidia (3–7 × 2–6 μm vs. 2.5–4.0 μm in diameter), and irregularly ovoid to elongate, twisted hülle cells compared to unobserved hülle cells of *A. dehongensis* (Raper and Fennell 1965). Morphologically, *Aspergillus dehongensis* differs from *A. puniceus* in that the latter has slightly rough, smaller conidia (3–7 × 2–6 μm vs. 2.5–3.3 μm in diameter), and elongated, crescent-shaped, or irregularly coiled hyphal cells, which aggregate into conspicuous yellow masses (Raper and Fennell 1965). In addition, the PHI test results (Fig. 6A) revealed no significant recombination relationships between *A. dehongensis* and its phylogenetically related taxa within the dataset, and no strongly supported conflict among individual gene trees (Suppl. material 1: figs S1–S3). Based on the phylogenetic analyses and morphological characteristics, *Aspergillus dehongensis* is introduced as a new species.

###### *Sordariomycetes* O.E. Erikss. & Winka

***Hypocreales* Lindau, in Engler & Prantl, Nat. Pflanzenfam., Teil. I (Leipzig) 1(1): 343 (1897)**

#### Hypocreaceae

Taxon classificationAnimaliaHypocrealesHypocreaceae

De Not. [as ‘Hypocreacei’], G. bot. ital. 2(1): 48 (1845)

CAE90DD4-A85B-5F90-ADAB-3F81E4C979AD

##### Notes.

*Hypocreaceae* was introduced by Lindau (1897) and redefined by Rossman et al. (1999), and is typified by *Hypocrea* Fr. The sexual morphs of *Hypocreaceae* are characterized by superficial or immersed, erumpent ascomata, often with papillate apex and rarely bear thick-walled setae, pallid to brightly pigmented, brown, blue or violet peridium, J- asci, often lacking an apical ring. Ascospores are uniseriate, biseriate, or arranged in fascicles, hyaline, yellowish, pinkish to greenish, or occasionally brown, verruculose or longitudinally striate, 1-celled or have one to several septa, occasionally with longitudinal septa (Hyde et al. 2020). In some cases, ascospores disarticulate into part-spores or bud to form conidia within the ascus. The asexual morphs are characterized by hyphomycetous or coelomycetous forms that produce hyaline to brightly colored conidia, although aleurioconidia and ampulloconidia are less commonly observed (Hyde et al. 2020). Some species exhibit a Gliocladium-like asexual morph or possess transversely striate ascospores (Rossman et al. 1999; Perera et al. 2023). Members of the family have been reported as saprobes, endophytes, and pathogens of animals and mushrooms (Dighton 1995; Sung et al. 2007; Back et al. 2010; Yu et al. 2020; Perera et al. 2023). Species of *Hypocreaceae* are important in agriculture as biocontrol agents and produce microbial natural compounds that are valuable in the pharmaceutical and drug industries (Hyde et al. 2020; Perera et al. 2023). To date, *Hypocreaceae* comprises 18 accepted genera (Hyde et al. 2024). In previous studies, 67 species belonging to 13 genera of *Hypocreaceae* have been reported from caves worldwide (Suppl. material 1: table S2).

#### Trichoderma

Taxon classificationAnimaliaHypocrealesHypocreaceae

Pers., Neues Mag. Bot. 1: 92 (1794)

9133A4B4-19E3-5BDA-8ECE-9ED09E8AB9A3

##### Notes.

*Trichoderma* was established by Persoon (1794), with *T. viride* as the type and only species retained from the original four (Zhao et al. 2023b; Liu et al. 2025). It is now a large genus with over 500 accepted species, widely distributed in soil, decaying wood, plant leaves, bark, root systems, or other tissues, as well as in caves and even the human body (Zheng et al. 2021; Zhao et al. 2024; Liu et al. 2025). *Trichoderma* species exhibit saprobic, endophytic, and parasitic lifestyles (Zheng et al. 2021; Zhao et al. 2023b). Members are morphologically characterized by fast-growing colonies, branched conidiophores, flask-shaped phialides, and mostly green conidia (Bissett 1984; Zhao et al. 2023b). Traditional identification relies on morphology; however, species are highly similar. Subsequently, multi-locus approaches have been widely accepted and applied to species identification in *Trichoderma* (Chaverri and Samuels 2003; Li et al. 2013; Zhu and Zhuang 2015; Zheng et al. 2021; Cao et al. 2022; Zhao et al. 2023b). Recently, Cai and Druzhinina (2021) published an authoritative molecular identification framework for *Trichoderma*, supported by the International *Trichoderma* Commission (Cai and Druzhinina 2021; Zhao et al. 2023b, 2024; Liu et al. 2025). At present, more than 500 species have been recognized across 13 clades in *Trichoderma* (Chaverri et al. 2015; Jaklitsch and Voglmayr 2015; Bustamante et al. 2021; Liu et al. 2025; Xiao et al. 2025). Ecologically and economically, *Trichoderma* species serve as biocontrol agents, plant growth promoters, and producers of enzymes and antibiotics; however, some strains are pathogenic to mushrooms and animals (Cao et al. 2024; Zeng et al. 2024; Zhao et al. 2024; Liu et al. 2025). *Trichoderma* is one of the most common genera found in caves, following *Aspergillus* and *Penicillium*, with 41 species reported to date from cave environments, including the newly described species reported in Hongsanan et al. (2025) and Liu et al. (2025). According to the standards of Cai and Druzhinina (2021), we introduced seven new species, viz. *Trichoderma dehongense*, *T. hongheense*, *T. mangshiense*, *T. neogongcheniae*, *T. neopyramidale*, *T. nujiangense*, *T. yingjiangense*, and one new host record in cave habitat.

### Phylogenetic analyses: *Trichoderma*

**Figure 7.**
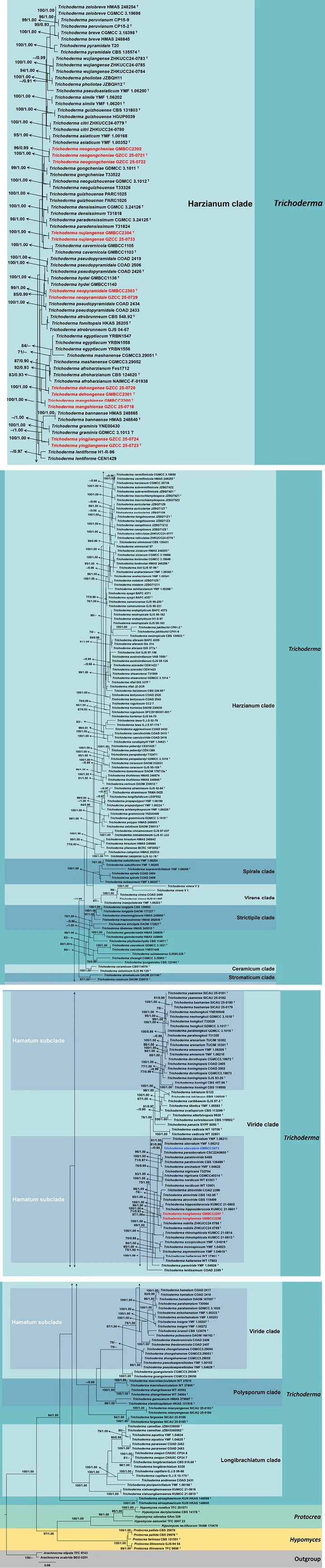
Consensus phylogram of 1,000 trees resulting from a RAxML analysis of the (ITS, *rpb*2, and *tef*1-α) alignment of the analyzed *Trichoderma* sequences. The RAxML analysis of the combined dataset yielded the best-scoring tree, with a final ML optimization likelihood value of -44522.773404. This tree was generated from 630, 1,005, and 566 base pairs of ITS, *rpb*2, and *tef*1-α characters, respectively. The matrix comprises 1514 distinct alignment patterns, with 24.35% of the sequences containing gaps and indeterminate traits. Base frequencies were estimated as follows A = 0.239220, C = 0.269107, G = 0.247011, T = 0.244663, substitution rates AC = 1.236378, AG = 3.614687, AT = 1.134273, CG = 0.838985, CT = 5.208442, GT = 1.000000, proportion of invariable sites I = 0.327543, gamma distribution shape parameter α = 0.714228. The trees from both analyses (ML and BI) were identical. RAxML bootstrap support values (ML equal to or above 70%) and Bayesian inference (BYPP equal to or above 0.90) are given at the nodes (ML/BYPP). Newly generated sequences in this study are in blue and red. Type strains are denoted with ^T^. The scale bar represents the expected number of changes per site.

The phylogeny of *Trichoderma* was based on the combined ITS, *rpb*2, and *tef*1-α sequence data. The aligned dataset encompassed 297 strains, representing 182 taxa, including *Hypomyces* (Fr.) Tul. & C. Tul. (five strains representing five taxa), *Protocrea* Petch (five strains representing three taxa), *Trichoderma* (285 strains representing 172 taxa), and the outgroup taxa of *Arachnocrea scabrida* Yoshim. Doi (BEO 0201) and *A. stipata* (Lib. ex Fuckel) Z. Moravec (TFC 9743). Multi-locus phylogenetic analyses based on the combined ITS, *rpb*2, and *tef*1-α show that our strains belong to *Trichoderma* (Fig. 6). *Trichoderma neogongcheniae* formed a distinct clade closely related to *T. gongcheniae* and *T. paradensissimum*, *Trichoderma nujiangense* formed a distinct clade closely related to *T. guizhouense*, *Trichoderma yingjiangense* formed a distinct clade closely related to *T. bannaense and T. graminis*, *Trichoderma neopyramidale* formed a distinct clade closely related to *T. pseudopyramidale*, and *Trichoderma dehongense* formed a distinct clade closely related to *T. afroharzianum* and *T. mangshiense*. *Trichoderma hongheense* formed a distinct clade closely related to *T. hipposiderocola* and *T. atroviride*. The strain GMBCC2473 clustered together with *T. obovatum* Z.F. Yu & Y.F. Lv (YMF 1.06211 (type) and YMF 1.06212). Furthermore, PARC1025 and PARC1026 did not cluster with *T. guiguiensis* (CBS 131803 (type), HGUP0039) in the present phylogenetic analyses (Fig. 6). Pairwise sequence comparisons revealed notable molecular divergence between PARC1025 and CBS 131803 (type), specifically, a 2.00% (16/801 bp, without gap) difference in *rpb*2, and 0.27% (1/377 bp, with one gap) difference in *tef*1-α. According to the species delimitation criteria proposed by Cai and Druzhinina (2021), these levels of sequence divergence indicate that PARC1025 and PARC1026 are unlikely to represent *T. guiguiensis*. The 13 clades of *Trichoderma* labelled in the phylogenetic tree (Fig. 7) are in accordance with Qin and Zhuang (2016), Zheng et al. (2021), and Zhao et al. (2023c, 2024).

#### Trichoderma dehongense

Taxon classificationAnimaliaHypocrealesHypocreaceae

X.F. Liu, Karun., Tibpromma & K.D. Hyde sp. nov.

3521FADF-769E-5685-B61B-58AFF2D57C80

Index Fungorum: IF905013

Fig. 8

##### Etymology.

Named after the type locality, Dehong.

##### Holotype.

HKAS149895.

##### Description.

Associated with soil in the cave environment. Asexual morph on PDA: ***Vegetative hyphae*** 2–5 μm wide, septate, branched, hyaline, smooth-walled. ***Conidiophores*** pyramidal with verticillate, comprising a recognizable main axis, pairs or solitary branches, longer branches near the base, and short branches or solitary phialides arising near the apex. ***Phialides*** 5–13 × 3–5 µm (x̄ = 10.47 × 3.41 μm, n = 50), ampulliform to lageniform, generally formed on terminal branches in divergent whorls of 2–5. ***Supporting cells*** 5–17 × 2–4 µm (x̄ = 10.09 × 2.97 μm, n = 50). ***Conidia*** 4–5 × 3–4 µm (x̄ = 4.01 × 3.79 μm, n = 100), subglobose to globose, or ovoid, light gray to green, smooth-walled. ***Chlamydospores*** 4–12 × 4–9 µm (x̄ = 8.28 × 6.23 μm, n = 40), single or in clusters, terminal and intercalary, variable in shape, mostly subglobose to globose, thick-walled, hyaline. Sexual morph: Undetermined.

##### Culture characteristics.

Colonies on PDA attaining 60 mm after 7 days at room temperature (25 ± 2 °C), white, gray to green, sparse aerial mycelium on the surface, regularly circular with water drops; reverse reddish-brown. The white reproductive mycelium covers the stromatic colonies after 30 days.

##### Material examined.

CHINA • Yunnan Province, Dehong Dai and Jingpo Autonomous Prefecture, Mang City, Mengga Town, Sanxian Cave, soil, 24.232220°N, 98.426103°E, elev. ca 1528.89 m, 23 April 2024, Xiangfu Liu and Xuemei Chen, MS14D (HKAS149895, holotype), ex-type GMBCC2301 = GZCC 25-0719, other living culture GZCC 25-0720.

##### Notes.

In our phylogenetic analyses of ITS, *rpb*2, and *tef*1-α, *Trichoderma dehongense* (GZCC 25-0719 (ex-type), and GZCC 25-0720) formed a distinct lineage with 83% ML and 0.93 PP support, clustering as the sister taxon to *T. afroharzianum* and *T. mangshiense* in the harzianum clade (Fig. 7). Moreover, nucleotide comparisons between *Trichoderma dehongense* (GMBCC2301, ex-type) and *T. afroharzianum* (CBS 124620, ex-type) revealed 1.04% (9/836 bp, without gap) base pair differences in *rpb*2, and 6.04% (20/331 bp, with 4 gaps) differences in *tef*1-α. *Trichoderma dehongense* (GMBCC2301, ex-type) and *T. mangshiense* (GMBCC2300, ex-type) showed 2.59% (22/849 bp, 2 gaps) base pair differences in *rpb*2, and 4.02% (14/348 bp, with 3 gaps) differences in *tef*1-α. Morphologically, *T. dehongense* is different from *T. afroharzianum* by having more whorls (2–5 vs 1–3), longer phialides (5–13 × 3–5 µm vs 5.2–10.2 × 2.5–3.5 µm) on terminal branches, larger conidia (4–5 × 3–4 µm vs 2.7–3.5 × 2.5–3.2 μm), and subglobose to globose, thick-walled chlamydospores, compared to rare chlamydospores of *T. afroharzianum* (Chaverri et al. 2015). *Trichoderma dehongense* is different from *T. mangshiense* by more whorls (2–5 vs 2–3), shorter phialides (5–13 × 3–5 µm vs 7–15 × 3–5 µm) on terminal branches, and longer chlamydospores (4–12 × 4–9 µm vs 4–9.5 × 4–9 µm). *Trichoderma afroharzianum* was first introduced in a phylogenetic study of *T. harzianum* by Druzhinina et al. (2010) but was not validly published because it lacked a formal

**Figure 8.**
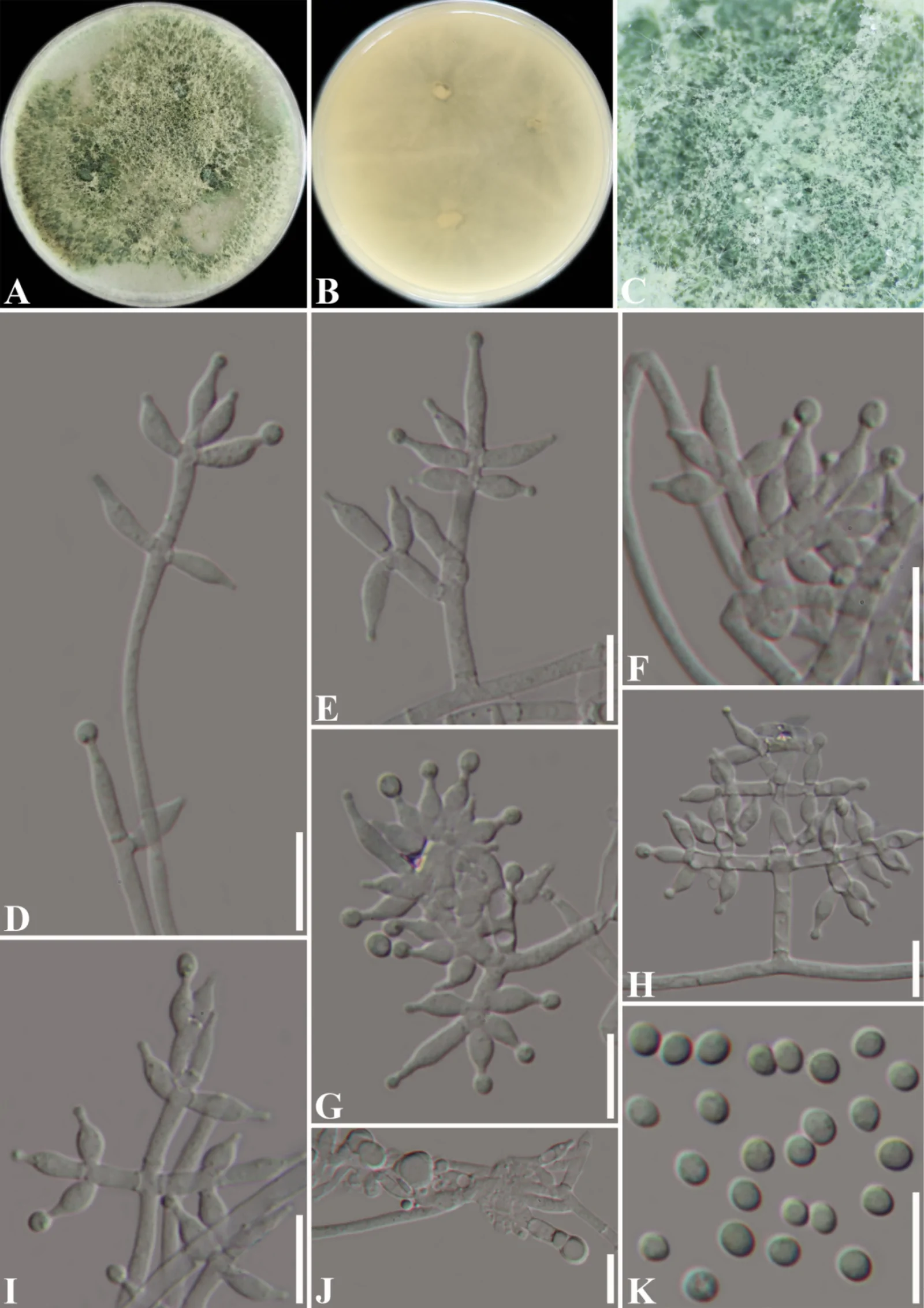
*Trichoderma dehongense* (GZCC 25-0719, ex-type). **A, B** Colony on PDA (above and below); **C** Sporulation on PDA (35 days old culture); **D, I** Conidiophores, phialides, and conidia; **J** Chlamydospores; **K** Conidia. Scale bars: 10 μm (**D–K**).

##### description.

Chaverri et al. (2015) later reintroduced and validly published the species under the name *T. afroharzianum*, providing detailed descriptions. In addition, the PHI test results (Fig. 6B1) revealed no significant recombination relationships between *T. dehongense* and its phylogenetically related taxa within the dataset. Based on phylogenetic analyses and morphological characteristics, *Trichoderma dehongense* is herein described as a new species.

#### Trichoderma hongheense

Taxon classificationAnimaliaHypocrealesHypocreaceae

X.F. Liu, Karun., Tibpromma & K.D. Hyde sp. nov.

0D6F27D9-29C8-5547-8896-6EED7FA0B33B

Index Fungorum: IF905014

Fig. 9

##### Etymology.

Named after the type locality, Honghe.

##### Holotype.

HKAS149896.

##### Description.

Associated with the air in the cave environment. Asexual morph on PDA: ***Vegetative hyphae*** 2–4 μm wide, septate, branched, hyaline, smooth-walled. ***Conidiophores*** pyramidal with verticillate, comprising a recognizable main axis, pairs or solitary branches, longer branches near the base, and short branches or solitary phialides arising near the tip. ***Phialides*** 5–13 × 2–5 µm (x̄ = 8.26 × 2.98 μm, n = 60), ampulliform to lageniform, generally formed on terminal branches in divergent whorls of 2–4. ***Supporting cells*** 5–11 × 2–4 µm (x̄ = 8.17 × 2.81 μm, n = 50). ***Conidia*** 2–5 × 2–4 µm (x̄ = 3.59 × 3.24 μm, n = 60), subglobose to globose, or ovoid, green, smooth-walled. ***Chlamydospores*** 5–11 × 3–9 µm (x̄ = 8.15 × 6.66 μm, n = 10), single or in clusters, terminal and intercalary, variable in shape, mostly subglobose to globose, thick-walled, hyaline, granulate. Sexual morph: Undetermined.

##### Culture characteristics.

Colonies on PDA attaining 60 mm after 7 days at room temperature (25 ± 2 °C), white, gray to yellow or green, sparse aerial mycelium on the surface, regularly circular, green conidiation occurs over time; reverse is green in the middle and white on the edges.

##### Material examined.

CHINA • Yunnan Province, Honghe Hani and Yi Autonomous Prefecture, Jianshui County, Miandian Town, Swallow Cave, 23.63684309°N, 103.05537745°E, elev. ca 1864.03 m, air, 24 December 2020, Xiangfu Liu, A7 (HKAS149896, holotype), ex-type GMBCC2297; *ibid*., A7B, living culture GMBCC2298.

**Figure 9.**
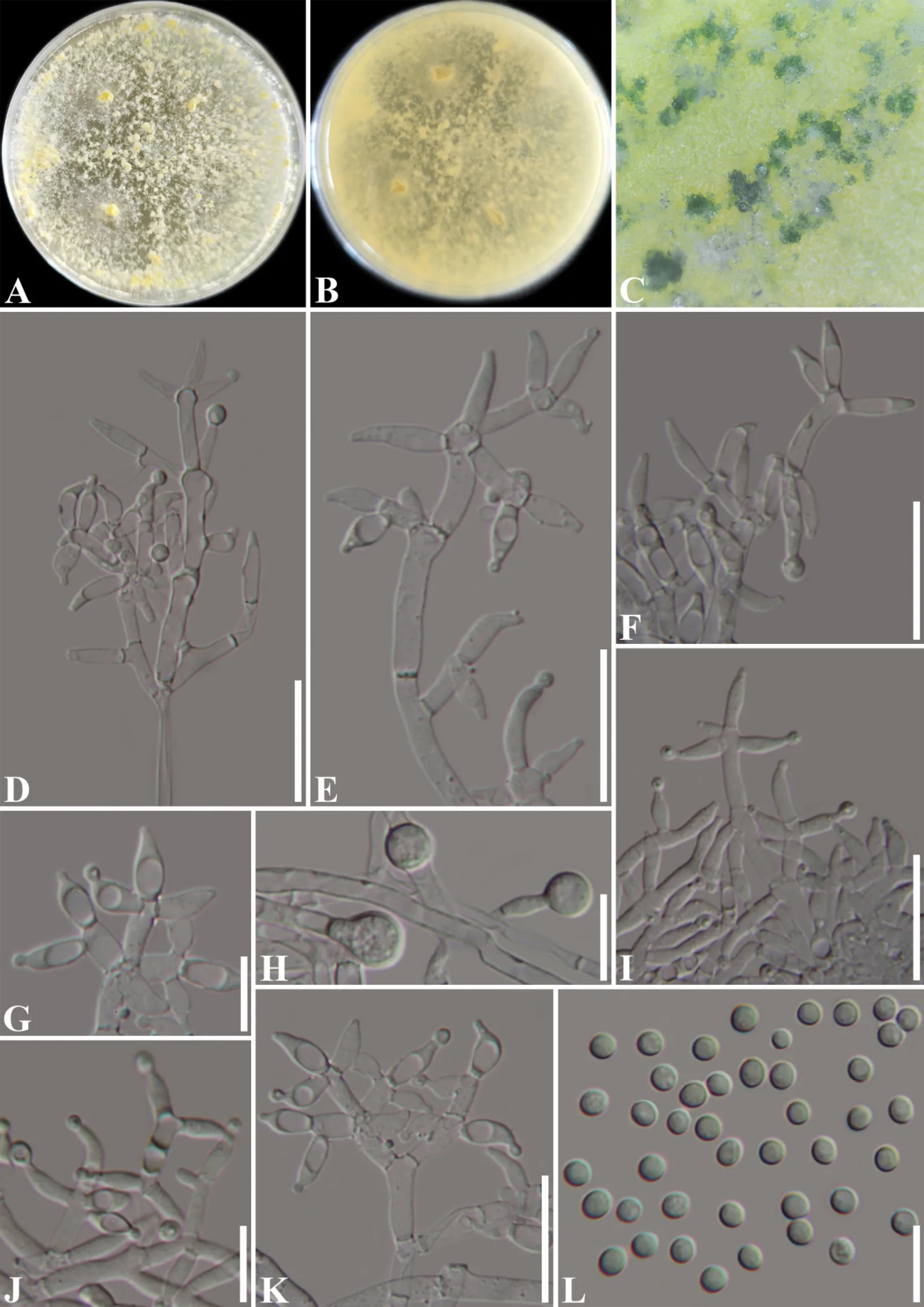
*Trichoderma hongheense* (GMBCC2297, ex-type). **A, B** Colony on PDA (above and below); **C** Sporulation on PDA (35 days old culture); **D–G, I–K** Conidiophores, phialides and conidia; **H** Chlamydospores; **L** Conidia. Scale bars: 20 μm (**D–F, I, K**); 10 μm (**G, H, J, L**).

##### Notes.

Phylogenetic analyses revealed that *Trichoderma hongheense* (GMBCC2297 (ex-type), and GMBCC2298) forms a distinct lineage with 100% ML and 1.00 PP support, and is closely related to *T. hipposidericola* Karun., Tibpromma & X.F. Liu, and *T. atroviride* P. Karst. in the atroviride clade (Fig. 7). The nucleotide comparisons between *Trichoderma hongheense* and *T. hipposidericola* (KUMCC 21-0801, ex-type) revealed 1.51% (13/869 bp, without gap) differences in *rpb*2 and 2.19% (16/731 bp, with 5 gaps) differences in *tef*1-α. *Trichoderma hongheense* and *T. atroviride* (CBS 142.95, ex-type) revealed 2.30% (20/869 bp, without gap) differences in *rpb*2, and 2.05% (15/731 bp, with 5 gaps) differences in *tef*1-α. Morphologically, *T. hongheense* differs from *T. hipposidericola* in having more whorls of phialides (2–4 vs 2–5) on terminal branches and lacking chlamydospores (Liu et al. 2023). In addition, the PHI test results (Fig. 6B2) revealed no significant recombination relationships between *T. hongheense* and its phylogenetically related taxa within the dataset. Based on the multi-gene phylogenetic analyses and morphological characteristics, *Trichoderma hongheense* is described as a new species.

#### Trichoderma mangshiense

Taxon classificationAnimaliaHypocrealesHypocreaceae

X.F. Liu, Karun., Tibpromma & K.D. Hyde sp. nov.

BC16036C-0CF5-5024-92A4-44683FFD2086

Index Fungorum: IF905015

Fig. 10

##### Etymology.

Named after the type locality, Mangshi.

##### Holotype.

GMB-W1539.

##### Description.

Associated with soil in the cave environment. Asexual morph on PDA: ***Vegetative hyphae*** 2–4 μm wide, septate, branched, hyaline, smooth-walled. ***Conidiophores*** pyramidal with verticillate, comprising a recognizable main axis, pairs or solitary branches, longer branches near the base, and short branches or solitary phialides arising near the tip. ***Phialides*** 7–15 × 3–5 µm (x̄ = 10.27 × 3.43 μm, n = 50), ampulliform to lageniform, generally formed on terminal branches in divergent whorls of 2–3. ***Supporting cells*** 5–17 × 2–4 µm (x̄ = 10.09 × 2.97 μm, n = 50). ***Conidia*** 4–5 × 3–4 µm (x̄ = 4.01 × 3.79 μm, n = 100), subglobose to globose or ovoid, light gray to green, smooth-walled. ***Chlamydospores*** 4–9.5 × 4–9 µm (x̄ = 6.28 × 5.92 μm, n = 40), single or in clusters, terminal and intercalary, variable in shape, mostly subglobose to globose, thick-walled, hyaline, granulate. Sexual morph: Undetermined.

**Figure 10.**
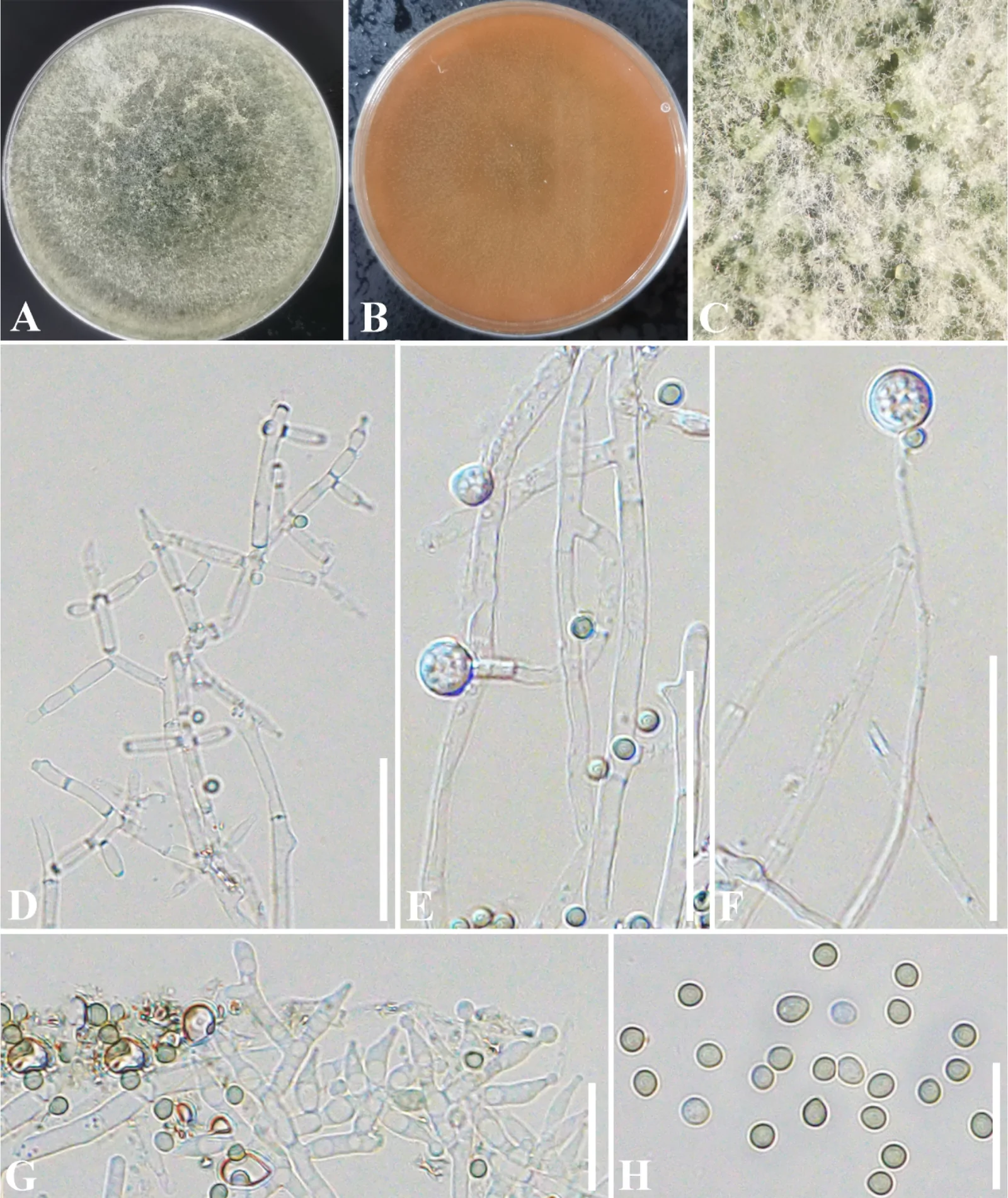
*Trichoderma mangshiense* (GMBCC2300, ex-type). **A, B** Colony on PDA (above and below); **C** Sporulation on PDA (35 days old culture); **D, G** Conidiophores, phialides and conidia; **E, F** Chlamydospores; **H** Conidia. Scale bars: 20 μm (**D–F**); 10 μm (**G, H**).

##### Culture characteristics.

Colonies on PDA attaining 60 mm after 7 days at room temperature (25 ± 2 °C), white, gray to green, sparse aerial mycelium on the surface, regularly circular with water drops; reverse reddish-brown. The white reproductive mycelium covers the stromatic colonies after 30 days.

##### Material examined.

CHINA • Yunnan Province, Dehong Dai and Jingpo Autonomous Prefecture, Mang City, Mengga Town, Sanxian Cave, soil, 24.232220°N, 98.426103°E, elev. ca 1528.89 m, 23 April 2024, Xiangfu Liu and Xuemei Chen, MS14A (GMB-W1539, holotype), ex-type GMBCC2300, other living culture GZCC 25-0718.

##### Notes.

Phylogenetic analysis of combined ITS, *tef*1-α, and *rpb*2 sequence data, *Trichoderma mangshiense* (GMBCC2300 (ex-type), and GZCC 25-0718) forms a lineage separated from *T. dehongense* and *T. afroharzianum* in the harzianum clade (Fig. 7). Moreover, nucleotide comparisons between *T. mangshiense* (GMBCC2300, ex-type) and *T. afroharzianum* (CBS 124620, ex-type) revealed 2.91% (30/886 bp, with 2 gaps) base pair differences in *rpb*2, and 5.41% (18/333 bp, with 3 gaps) differences in *tef*1-α. Morphologically, *T. mangshiense* differs from *T. afroharzianum* by having larger phialides (7–15 × 3–5 µm vs. 5.2–10.2 × 2.5–3.5 µm), smaller conidia (4–5 × 3–4 µm vs 2.7–3.5 × 2.5–3.2 µm), and rarely observed chlamydospores (Chaverri et al. 2015). Morphological differences between *T. mangshiense* and *T. dehongense* were listed under the notes of *T. dehongense*. In addition, the PHI test results (Fig. 6B1) revealed no significant recombination relationships between *T. mangshiense* and its phylogenetically related taxa within the dataset. Based on phylogenetic analyses and morphological characteristics, *Trichoderma mangshiense* is introduced as a new species.

#### Trichoderma neogongcheniae

Taxon classificationAnimaliaHypocrealesHypocreaceae

X.F. Liu, Karun., Tibpromma & K.D. Hyde sp. nov.

4F56C2A3-B647-5225-8BBE-377EB43038BD

Index Fungorum: IF905016

Fig. 11

##### Etymology.

Named after its phylogenetic closeness to *T. gongcheniae*.

##### Holotype.

GMB-W1538.

##### Description.

Associated with soil in the cave environment. Asexual morph on PDA: ***Vegetative hyphae*** 2–4 μm wide, septate, branched, hyaline, smooth-walled. ***Conidiophores*** pyramidal with verticillate, comprising a recognizable main axis, pairs or solitary branches, longer branches near the base, and short branches or solitary phialides arising near the tip. ***Phialides*** 3–11 × 3–8 µm (x̄ = 6.28 × 4.51 μm, n = 50), ampulliform to lageniform, generally formed on terminal branches in divergent whorls of 2–3. ***Supporting cells*** (3.8–) 6–14 (–21) × 2–4.5 µm (x̄ = 9.08 × 3.36 μm, n = 50). ***Conidia*** 3–4 × 2.5–4 µm (x̄ = 3.08 × 2.98 μm, n = 50), subglobose to globose or ovoid, green, smooth-walled. ***Chlamydospores*** 6–9 (–11) × 5–8 µm (x̄ = 7.87 × 6.07 μm, n = 15), single or in clusters, terminal and intercalary, variable in shape, mostly subglobose to globose, thick-walled, hyaline, granulate. Sexual morph: Undetermined.

**Figure 11.**
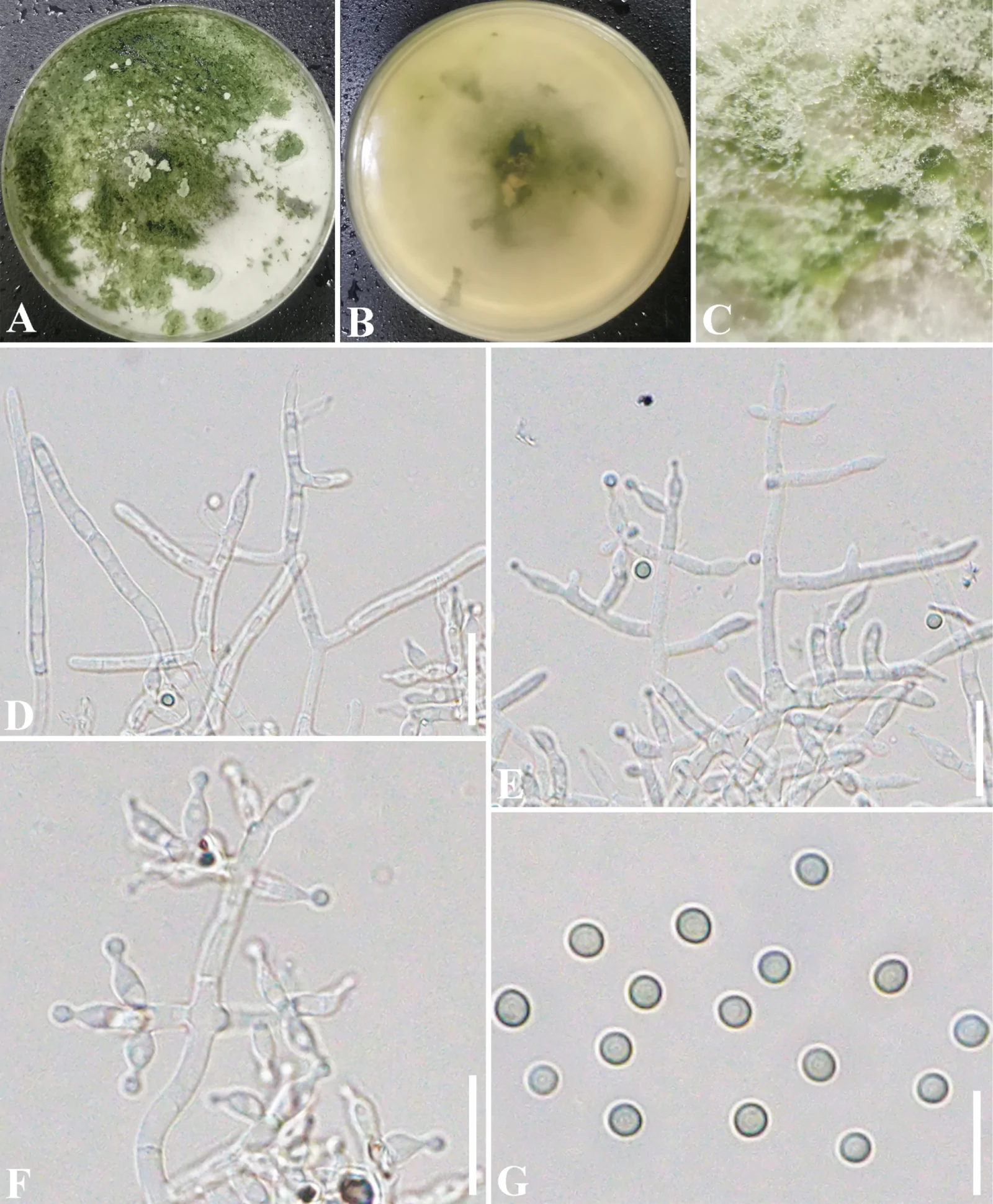
*Trichoderma neogongcheniae* (GZCC 25-0721, ex-type). **A, B** Colony on PDA (above and below); **C** Sporulation on PDA (35 days old culture); **D–F** Conidiophores, phialides and conidia; **G** Conidia. Scale bars: 10 μm (**D–G**).

##### Culture characteristics.

Colonies on PDA attaining 60 mm after 7 days at room temperature (25 ± 2 °C), white, gray to green, sparse aerial mycelium on the surface, regularly circular, green conidiation occurs over time; reverse is green in the middle and white on the edges.

##### Material examined.

CHINA • Yunnan Province, Dehong Dai and Jingpo Autonomous Prefecture, Mang City, Mengga Town, Sanxian Cave, feather, 24.232220°N, 98.426103°E, elev. ca 1528.89 m, 23 April 2024, Xiangfu Liu and Xuemei Chen, MS17B (GMB-W1538, holotype), ex-type GZCC 25-0721, other living cultures GMBCC2302, GZCC 25-0722.

##### Notes.

*Trichoderma neogongcheniae* (GZCC 25-0721 (ex-type), GZCC 25-0722, and GMBCC2302) is phylogenetically closely related to *T. gongcheniae* (Fig. 7). The morphology of these two species is similar in phialides, supporting cells, and conidia. However, the size of the phialides in *T. gongcheniae* (5.6–8.4 × 2.8–3.7 μm) is smaller than that in *T. neogongcheniae* (3–11 × 3–8 μm). Additionally, the conidia of *T. neogongcheniae* (3–4 × 2.5–4 μm) are larger than those of *T. gongcheniae* (2.8–3.3 × 2.7–3.1 μm). In terms of chlamydospores, *T. neogongcheniae* has bigger chlamydospores (6–9 × 5–8 μm) compared to *T. gongcheniae* (5.2–6.8 × 4.8–6.3 μm) (Zhao et al. 2024). Meanwhile, nucleotide comparisons between *Trichoderma neogongcheniae* (GZCC 25-0721, ex-type) and *T. gongcheniae* (GDMCC 3.1011, ex-type) revealed 2.68% (24/897 bp) base pair differences in *rpb*2. In addition, the PHI test results (Fig. 6B3) revealed no significant recombination relationships between *T. neogongcheniae* and its phylogenetically related taxa within the dataset. Therefore, we introduce *Trichoderma neogongcheniae* as a new species.

#### Trichoderma neopyramidale

Taxon classificationAnimaliaHypocrealesHypocreaceae

X.F. Liu, Karun., Tibpromma & K.D. Hyde sp. nov.

E7FF4B15-3540-53C3-9957-4FCCD8DAFDCC

Index Fungorum: IF905017

Fig. 12

##### Etymology.

Named after its phylogenetically closeness to *Trichoderma pseudopyramidale* and morphological similarity to *Trichoderma pyramidale*.

##### Holotype.

GMB-W1534.

##### Description.

Associated with rock surface in the cave environment. Asexual morph on PDA: ***Vegetative hyphae*** 2–8 μm wide, septate, branched, hyaline, smooth-walled. ***Conidiophores*** pyramidal with verticillate, comprising a recognizable main axis, pairs or solitary branches, longer branches near the base, and short branches or solitary phialides arising near the tip. ***Phialides*** 5–15 × 2–4 µm (x̄ = 8.82 × 2.79 μm, n = 60), ampulliform to lageniform, generally formed on terminal branches in divergent whorls of 1–3. ***Supporting cells*** 5–13 × 2–4 µm (x̄ = 7.91 × 2.63 μm, n = 50). ***Conidia*** 2–5 × 2–4 µm (x̄ = 3.44 × 2.95 μm, n = 90), subglobose to globose, or ovoid, green, smooth-walled. ***Chlamydospores*** 4–6 × 3–5 µm (x̄ = 5.14 × 4.34 μm, n = 10), single or in clusters, terminal and intercalary, variable in shape, mostly subglobose to globose, thick-walled, hyaline, granulate. Sexual morph: Undetermined.

**Figure 12.**
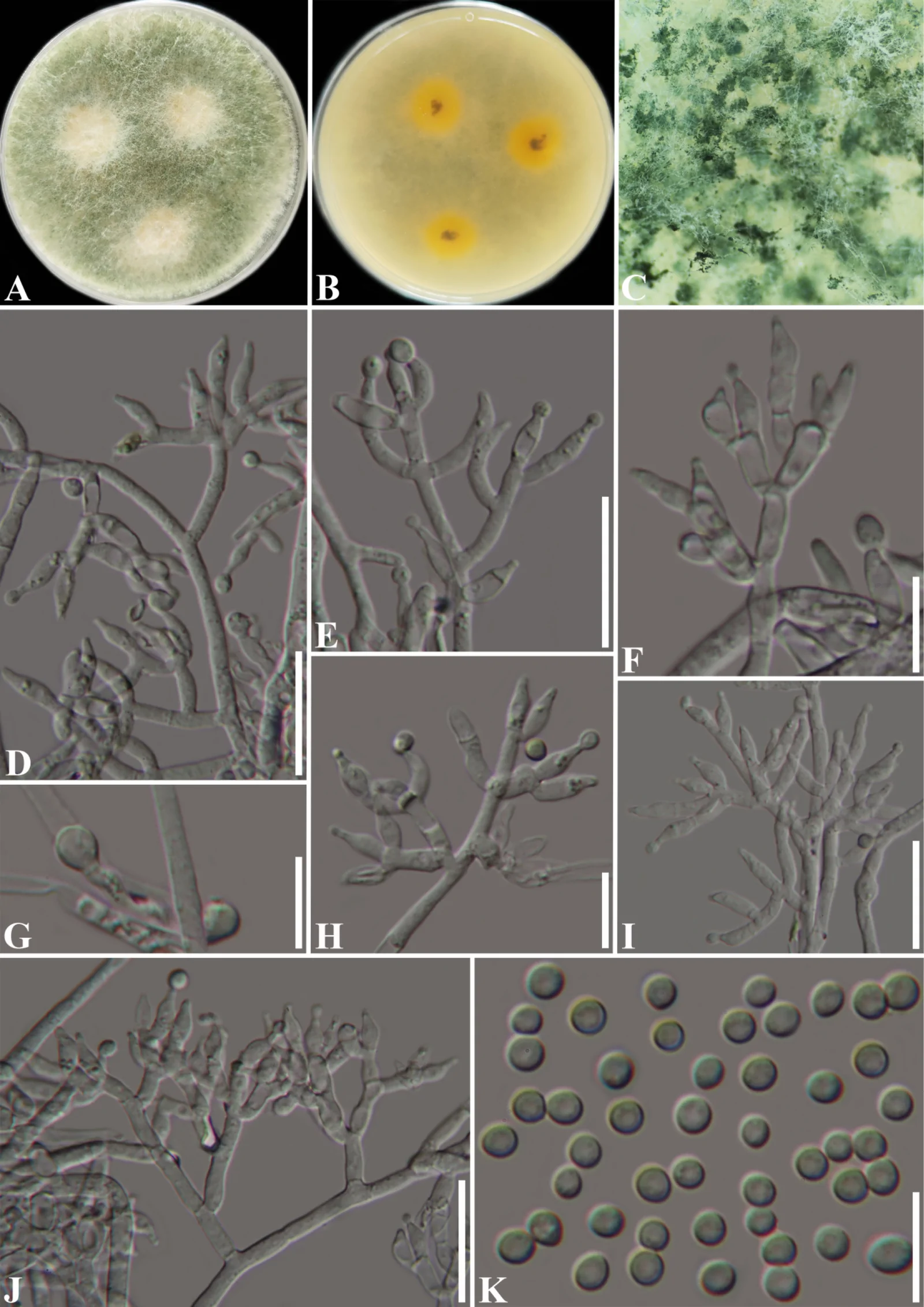
*Trichoderma neopyramidale* (GMBCC2303, ex-type). **A, B** Colony on PDA (above and below); **C** Sporulation on PDA (35 days old culture); **D–F, H–J** Conidiophores, phialides and conidia; **G** Chlamydospores; **K** Conidia. Scale bars: 20 μm (**D, E, I, J**); 10 μm (**F–H, K**).

##### Culture characteristics.

Colonies on PDA attaining 60 mm after 7 days at room temperature (25 ± 2 °C), white, gray to green, sparse aerial mycelium on the surface, regularly circular, green conidiation occurs over time, reverse is green in the middle and white on the edges.

##### Material examined.

CHINA • Yunnan Province, Dehong Dai and Jingpo Autonomous Prefecture, Mang City, Mengga Town, Sanxian Cave, rock surface, 24.232220°N, 98.426103°E, elev. ca 1528.89 m, 23 April 2024, Xiangfu Liu and Xuemei Chen, MS32C (GMB-W1534, holotype), ex-type GMBCC2303, *ibid*., MS32D (GMB-W1535, isotype), ex-isotype GZCC 25-0729.

##### Notes.

Phylogenetic analyses revealed that *Trichoderma neopyramidale* (GMBCC2303 (ex-type), and GZCC 25-0729) forms a distinct lineage and is closely related to *T. pseudopyramidale* and *T. hydei* X.F. Liu, Karun. & Tibpromma (Fig. 7). *Trichoderma neopyramidale* (GMBCC2303, ex-type) and *T. pseudopyramidale* (GMBCC2300, ex-type) revealed 2.52% (21/833 bp, 2gaps) base pair differences in *rpb*2, and 6.04% (35/579 bp, without gaps) differences in *tef*1-α. *Trichoderma neopyramidale* (GMBCC2303, ex-type) and *T. hydei* (GMBCC1136, ex-type) revealed 1.77% (16/904 bp, without gap) base pair differences in *rpb*2, and 2.92% (23/787 bp, without gap) differences in *tef*1-α. Morphologically, *T. neopyramidale* differs from *T. pseudopyramidale* by the latter having smaller phialides (5–15 × 2–4 µm vs. 5.3–8.6 × 2.2–2.9 µm), smaller supporting cells (5–13 × 2–4 µm vs. 3.3–7.1 × 1.9–2.8 µm), and larger conidia (2–5 × 2–4 µm vs. 2.1–2.9 × 2.3–2.9 µm) (Rodríguez et al. 2021). *Trichoderma neopyramidale* differs from *T. hydei* by the latter having white, gray to green culture, more (1–3 whorls vs. 2–3 whorls) and slightly longer (5–15 × 2–4 µm vs. 6–17 × 2–4 µm) phialides, bigger supporting cells (5–13 × 2–4 µm vs. 6–15 × 2–4 µm), and smaller conidia (2–5 × 2–4 µm vs. 2–4 × 2–3 µm) (Hongsanan et al. 2025). *Trichoderma neopyramidale* and *T. pyramidale* share several characteristics in common, such as pyramidal conidiophores with 2−4 whorls phialides. *Trichoderma pyramidale* has smalller phialides ((4.0−)5.5−11.5(−17.5) × (2.5−)2.8−3.7(−4.5)) and conidia ((2.8−) 3.2− 4.0(−4.7) × (2.5−) 3.0−3.5(−4.0) μm), and uncommon chlamydospores (Chaverri et al. 2015). In addition, the PHI test results (Fig. 6B4) revealed no significant recombination relationships between *T. neopyramidale* and its phylogenetically related taxa within the dataset. Based on phylogenetic analyses and morphological characteristics, *Trichoderma neopyramidale* is introduced as a new species.

#### Trichoderma nujiangense

Taxon classificationAnimaliaHypocrealesHypocreaceae

X.F. Liu, Karun., Tibpromma & K.D. Hyde sp. nov.

8589527A-C95F-5EAC-AEDF-B92991322655

Index Fungorum: IF905018

Fig. 13

##### Etymology.

Named after the type locality, Nujiang.

##### Holotype.

GMB-W1546.

##### Description.

Associated with rock surface in the cave environment. Asexual morph on PDA: ***Vegetative hyphae*** 2–6 μm wide, septate, branched, hyaline, smooth-walled. ***Conidiophores*** pyramidal with verticillate, comprising a recognizable main axis, pairs or solitary branches, longer branches near the base, and short branches or solitary phialides arising near the tip. ***Phialides*** 5–11 × 2–5 µm (x̄ = 7.94 × 3.32 μm, n = 50), ampulliform to lageniform, generally formed on terminal branches in divergent whorls of 2–3. ***Supporting cells*** 5–13 (–18) × 2–4 µm (x̄ = 9.11 × 2.92 μm, n = 50). ***Conidia*** 3–4 × 2–4 µm (x̄ = 3.28 × 3.21 μm, n = 50), subglobose to globose, or ovoid, green, smooth-walled. ***Chlamydospores*** 5–8 (–14) × 5–8 (–9) µm (x̄ = 7.13 × 6.76 μm, n = 20), single or in clusters, terminal and intercalary, variable in shape, mostly subglobose to globose, thick-walled, hyaline, granulate. Sexual morph: Undetermined.

**Figure 13.**
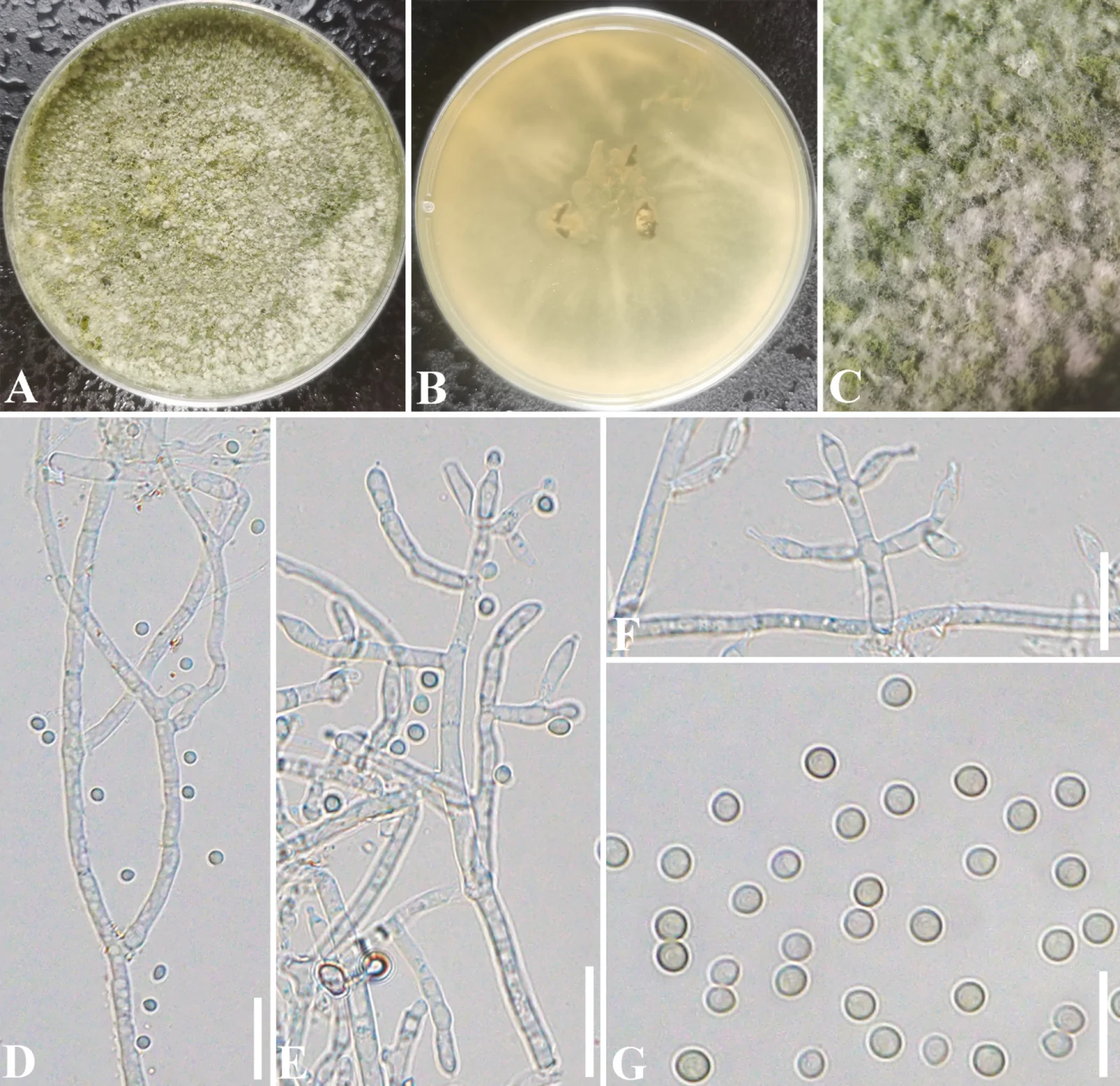
*Trichoderma nujiangense* (GMBCC2304, ex-type). **A, B** Colony on PDA (above and below); **C** Sporulation on PDA (35 days old culture); **D–F** Conidiophores, phialides and conidia; **G** Conidia. Scale bars: 10 μm (**D–G**).

##### Culture characteristics.

Colonies on PDA attaining 60 mm after 7 days at room temperature (25 ± 2 °C), white, gray to green, sparse aerial mycelium on the surface, regularly circular, green conidiation occurs over time; reverse is green in the middle and white on the edges.

##### Material examined.

CHINA • Yunnan Province, Nujiang Lisu Autonomous Prefecture, Lushui City, Daxingdi Town, Walaya Cave, rock surface, 26.080743°N, 98.848391°E, elev. ca 850.46 m, 23 April 2024, Xiangfu Liu and Xuemei Chen, NJ49A (GMB-W1546, holotype), ex-type GMBCC2304, other living culture GZCC 25-0733.

##### Notes.

Phylogenetic analyses revealed that *Trichoderma nujiangense* (GMBCC2304, ex-type) is closely related to *T. cavernicola* (GMBCC1103 (ex-type) and GMBCC1105) in Fig. 7. Morphologically, *Trichoderma cavernicola* differs from *T. nujiangense* by having more (2–4(–5) whorls vs. 2–3 whorls) and longer (6–17 × 2–4 µm vs. 5–11 × 2–5 µm) phialides on terminal branches, bigger supporting cells (4–22 × 2–8 µm vs. 5–13 (–18) × 2–4 µm), and smaller conidia (4–7 × 2–5 µm vs. 3–4 × 2–4 µm) (Liu et al. 2025). The nucleotide comparisons between *T. nujiangense* (GMBCC2304, ex-type) and *T. cavernicola* (GMBCC1103, ex-type) revealed 2.35% (15/638 bp, with 2 gaps) base pair differences in ITS, 4.37% (39/892 bp, without gaps) differences in *rpb*2, and 0.38% (3/787 bp, without gaps) differences in *tef*1-α. In addition, the PHI test results (Fig. 6B3) revealed no significant recombination relationships between *T. nujiangense* and its phylogenetically related taxa within the dataset. Therefore, we introduce *Trichoderma nujiangense* as a new species.

#### Trichoderma obovatum

Taxon classificationAnimaliaHypocrealesHypocreaceae

Z.F. Yu & Y.F. Lv, in Zheng, Qiao, Lv, Du, Zhang & Yu, Journal of Fungi 7 (6, no. 467): 22 (2021)

BD6427DD-FF7A-5C4E-9EE1-F43241308B7E

Index Fungorum: IF834570

Fig. 14

##### Description.

Associated with petroleum wax in the cave environment. Asexual morph on PDA: ***Vegetative hyphae*** septate, branched, hyaline, smooth-walled, 1–5 μm wide. ***Conidiophores*** pyramidal with verticillate, paired lateral branches. ***Phialides*** 3–12 × 1–4 µm (x̄ = 6.83 × 2.64 μm, n = 30), generally formed on terminal branches, ampulliform, in divergent whorls of 1–4. ***Supporting cells*** 3–16 × 1–4 µm (x̄ = 8.99 × 2.97 μm, n = 30). ***Conidia*** 3–6 × 2–5 µm (x̄ = 4.26 × 3.55 μm, n = 60) globose, ellipsoidal to ovoid, green, smooth-walled. ***Chlamydospores*** not observed. **Sexual morph**: Undetermined.

**Figure 14.**
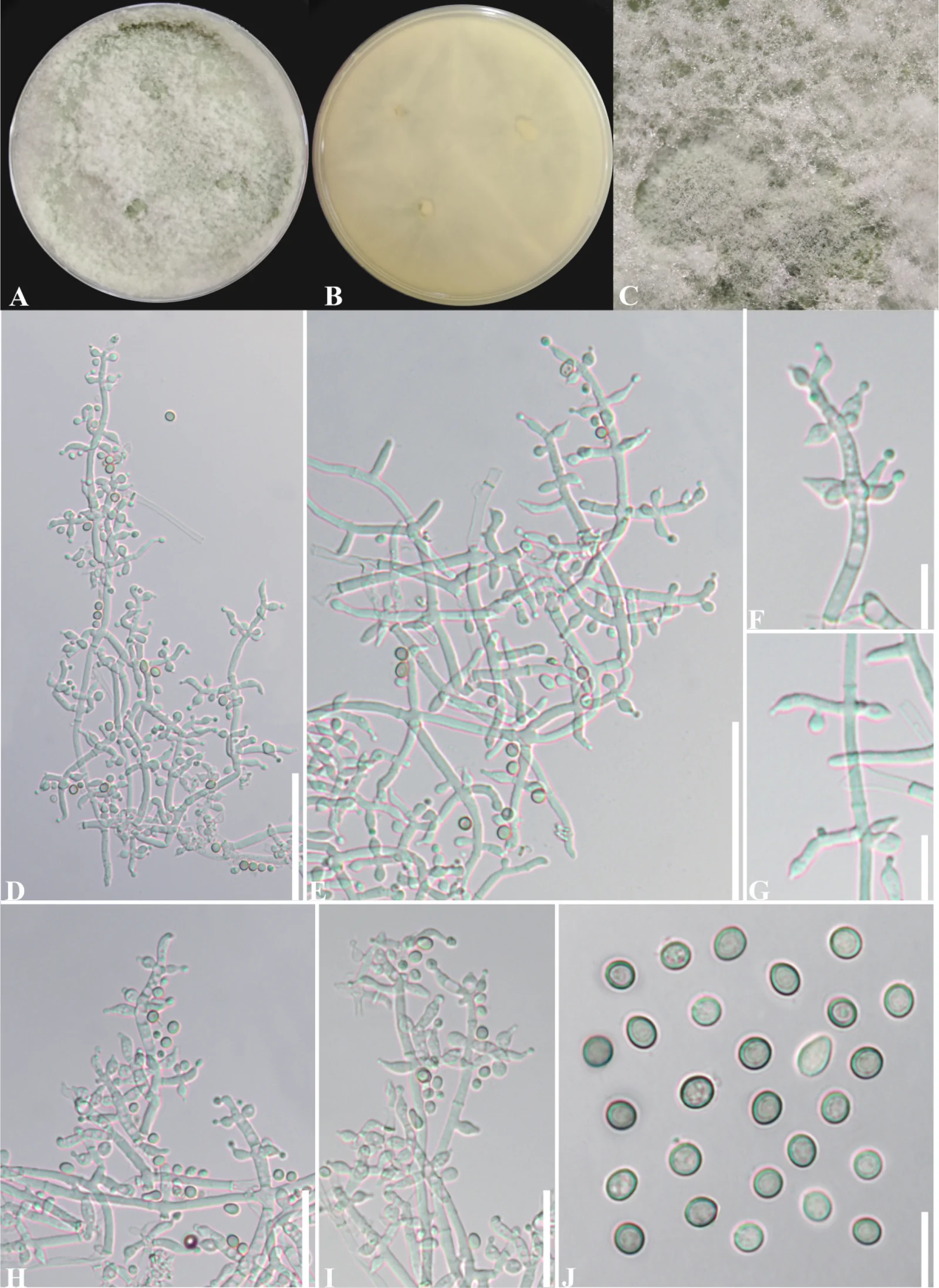
*Trichoderma obovatum* (GMBCC2473, new substrate record in cave habitat). **A, B** Colony on PDA (above and below); **C** Sporulation on PDA (35 days old culture); **D–I** Conidiophores, phialides, and conidia; **J** Conidia. Scale bars: μm (**D, E**); 30 μm (**H, I**); 10 μm (**F, G, J**).

##### Culture characteristics.

Colonies on PDA attaining 50–60 mm after 7 days at room temperature (25 ± 2 °C), white, pale yellow to green, sparse aerial mycelium on the surface, reverse white to pale yellow. The white reproductive mycelium covers the stromatic colonies after 25 days. Odor absent.

##### Substratum.

Feet surface of living bats (Liu et al. 2023), petroleum wax (this study), and soil (Zheng et al. 2021).

##### Distribution.

China (Zheng et al. 2021; Liu et al. 2023; this study).

##### Material examined.

CHINA • Yunnan Province, Nujiang Lisu Autonomous Prefecture, Lushui City, Daxingdi Town, Walaya Cave, petroleum wax, 26.080743°N, 98.848391°E, elev. ca 850.46 m, 23 April 2024, Xiangfu Liu and Xuemei Chen, NJ5C (GMB-W1250), living culture GMBCC2473.

##### Notes.

*Trichoderma obovatum* was erected by Zheng et al. (2021) and was isolated from soil in Yunnan, China. Our isolate (GMBCC2473) clustered with *T. obovatum* (YMF 1.06211 (ex-type) and YMF 1.06212) within the *Atroviride* clade (Fig. 7). The nucleotide comparison between our strain (GMBCC2473) and *T. obovatum* (YMF 1.06211, ex-type) showed 0.35% (2/572 bp, 2 gaps) base pair differences in ITS, 0.44% (4/904 bp, 1 gap) differences in *rpb*2, and no (0/712 bp, without gap) differences in *tef*1-α. Morphologically, the new isolate (GMBCC2473) is similar to *T. obovatum* in having curved to sinuous phialides, globose to oval or obovate conidia of slightly different sizes, and the absence of chlamydospores (Zheng et al. 2021; Liu et al. 2023). Based on combined phylogeny and morphological evidence, our strains were identified as *T. obovatum*. This species has previously been isolated from soil and bat surfaces in China (Zheng et al. 2021; Liu et al. 2023); however, this represents the first record of *T. obovatum* from a cave environment.

#### Trichoderma yingjiangense

Taxon classificationAnimaliaHypocrealesHypocreaceae

X.F. Liu, Karun., Tibpromma & K.D. Hyde sp. nov.

5FAAC421-4FAF-5E2C-90A0-4437D671E1F7

Index Fungorum: IF905019

Fig. 15

##### Etymology.

Named after the type locality, Yingjiang.

##### Holotype.

HKAS149894.

##### Description.

Associated with bat guano in a cave environment. Asexual morph on PDA: ***Vegetative hyphae*** 2–8 μm wide, septate, branched, hyaline, smooth-walled. ***Conidiophores*** pyramidal with verticillate, comprising a recognizable main axis, pairs or solitary branches, longer branches near the base, and short branches or solitary phialides arising near the tip. ***Phialides*** 6–12 × 2–4 µm (x̄ = 7.99 × 2.83 μm, n = 100), ampulliform to lageniform, generally formed on terminal branches in divergent whorls of 2–4. ***Supporting cells*** 4–14 × 2–4 µm (x̄ = 7.64 × 2.61 μm, n = 40). ***Conidia*** 2–5 × 2–4 µm (x̄ = 3.17 × 2.72 μm, n = 100), subglobose to globose, or ovoid, green, smooth-walled. ***Chlamydospores*** 4–9 × 3–7 µm (x̄ = 6.21 × 5.14 μm, n = 10), single or in clusters, terminal and intercalary, variable in shape, mostly subglobose to globose, thick-walled, hyaline, granulate. Sexual morph: Undetermined.

##### Culture characteristics.

Colonies on PDA attaining 60 mm after 7 days at room temperature (25 ± 2 °C), white, gray to green, sparse aerial mycelium on the surface, regularly circular, green conidiation occurs over time; reverse is green in the middle and white on the edges.

##### Material examined.

CHINA • Yunnan Province, Dehong Dai and Jingpo Autonomous Prefecture, Mang City, Mengga Town, Sanxian Cave, bat guano, 24.232220°N, 98.426103°E, elev. ca 1530.89 m, 23 April 2024, Xiangfu Liu and Xuemei Chen, MS19B (HKAS149894, holotype), ex-type GZCC 25-0723, other living culture GZCC 25-0724.

##### Notes.

*Trichoderma yingjiangense* (GZCC 25-0723, ex-type) is phylogenetically closely related to *T. graminis* C.L. Zhang and *T. bannaense* Kai Chen & W.Y. Zhuang in the harzianum clade (Fig. 7). However, *Trichoderma yingjiangense* is different from *T. graminis* by the latter having 2–5 whorls and of shorter phialides (6–12 × 2–4 µm vs 5.2–8.1 × 2.7–3.9 µm) on terminal branches, slightly smaller conidia (2–5 × 2–4 µm vs 3.1–4.2 × 2.9–3.9 µm), and smaller chlamydospores (4–9 × 3–7 µm vs 5.6–7.6 × (5.1–)5.3–7.1 μm) (Zhao et al. 2024). Furthermore, *T. graminis* was isolated as an endophyte from living leaf sheaths of *Oplismenus undulatifolius* (Zhao et al. 2024). *Trichoderma yingjiangense* differs from *T. bannaense* in that the latter has more whorls (2–4 vs 2–5) of shorter phialides (6–12 × 2–4 µm vs 7.5–10 × 2.6–3.6 μm) on terminal branches, smaller conidia (2–5 × 2–4 µm vs 2.5–3.6 × 2.5–3.1 μm), and lacks chlamydospores (Chen and Zhuang 2017). The nucleotide comparisons between *T. yingjiangense* (GZCC 25-0723, ex-type) and *T. graminis* (GDMCC 3.1013, ex-type) showed 4.89% (44/900 bp, without gap) base pair differences in *rpb*2 and 2.03% (16/790 bp, with 6 gaps) differences in *tef*1-α. Nucleotide comparison between *T. yingjiangense* (GZCC 25-0723, ex-type) and *T. bannaense* (HMAS 248840, ex-type) showed 2.55% (15/589 bp, without gap) base pair differences in ITS, 3.43% (26/757 bp, without gap) base pair differences in *rpb*2, and 2.03% (16/790 bp, with 6 gaps) differences in *tef*1-α. In addition, the PHI test results (Fig. 6B5) revealed no significant recombination relationships between *T. yingjiangense* and its phylogenetically related taxa within the dataset. Based on the phylogenetic analyses and morphological characteristics, *Trichoderma yingjiangense* is introduced as a new species.

**Figure 15.**
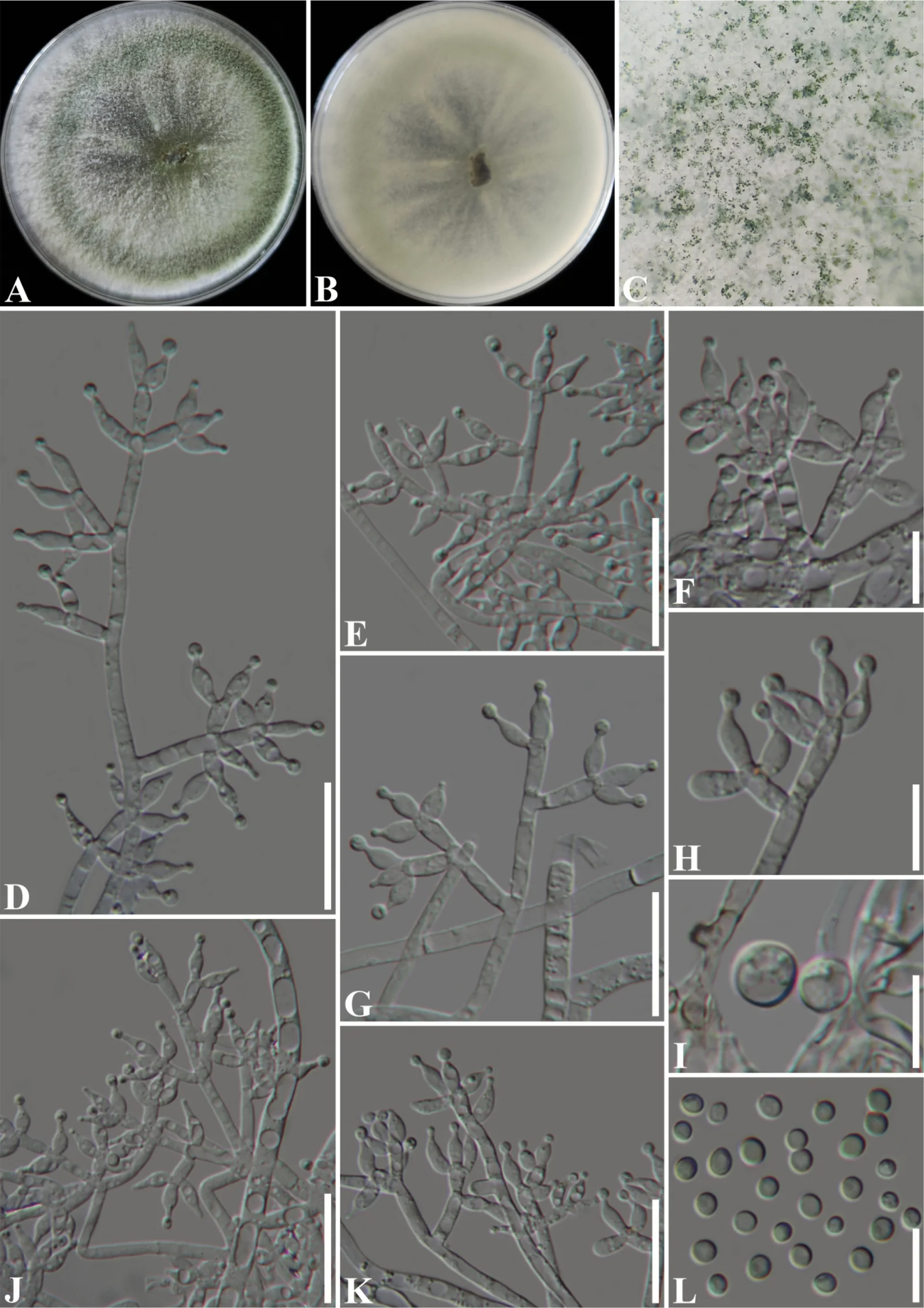
*Trichoderma yingjiangense* (GZCC 25-0723, ex-type). **A, B** Colony on PDA (above and below); **C** Sporulation on PDA (35 days old culture); **D–H, J, K** Conidiophores, phialides and conidia; **I** Chlamydospores; **L** Conidia. Scale bars: 20 μm (**D, E, G, J, K**); 10 μm (**F, H, I, L**).

#### Nectriaceae

Taxon classificationAnimaliaHypocrealesNectriaceae

Tul. & C. Tul. [as ‘Nectriei’], Select. fung. carpol. (Paris) 3: 3 (1865)

2270958E-9AF7-5F7D-91C8-75C8C779273F

##### Notes.

*Nectriaceae* was established by Tulasne and Tulasne (1865) for the genus *Nectria* (Fr.) Fr. and was later accepted as a distinct family by Rossman et al. (1999) based on morphological characteristics. The sexual morphs of *Nectriaceae* are characterized by uniloculate ascomata that are yellow, orange, red, purple, or brown, and often exhibit color changes when treated with 3% potassium hydroxide (KOH) or 100% lactic acid (LA). These ascomata are globose to long-fusiform, with ellipsoid forms also occurring, and produce non-disarticulating ascospores (Rossman et al. 1999; Lombard et al. 2015; Zeng and Zhuang 2022; Perera et al. 2023). The asexual morphs are characterized by phialidic conidiogenous cells, producing aseptate to multi-septate conidia, which may be constricted at the septa or not, with or without distinct abscission scars; chlamydospores may be present or absent (Maharachchikumbura et al. 2016; Perera et al. 2023). Species of *Nectriaceae* have been reported as saprobes, pathogens, or endophytes, and have been isolated from a wide range of substrates, including living and decaying wood material, soil, freshwater habitats, insects, fruiting bodies of other fungi, and various other materials across tropical and subtropical regions worldwide (Rossman et al. 1999; Morrison et al. 2021; Zeng and Zhuang 2022; Rissi et al. 2024). Species of *Nectriaceae* are of considerable importance in biotechnological, medicinal, commercial, pharmaceutical, and industrial applications (Abdel-Azeem et al. 2019; Peitl et al. 2020; Cleveland et al. 2021; Ibrahim et al. 2021; Perera et al. 2023). To date, 38 species belonging to eight genera of *Nectriaceae* have been reported from caves worldwide (Suppl. material 1: table S2).

#### Cosmospora

Taxon classificationAnimaliaHypocrealesNectriaceae

Rabenh., Hedwigia 2: 59 (1862)

59B1FB6A-0EAB-5D2D-9168-91F525DB6E6F

##### Notes.

*Cosmospora* was introduced by Rabenhorst (1862) and typified by *C. coccinea* Rabenh. The genus is distributed across temperate and tropical regions (Gräfenhan et al. 2011), with approximately 87 epithets currently recorded in Index Fungorum (http://www.indexfungorum.org; accessed date 14 July 2026). Species of *Cosmospora* are mainly reported as saprobic or endophytic and are found in warm-temperate to tropical regions (Peintner et al. 2002; Gräfenhan et al. 2011; Herrera et al. 2015; Zeng and Zhuang 2016; Bao et al. 2023). The sexual morph of *Cosmospora* is characterized by orange-red to bright-red perithecia that turn dark red in KOH. The ascomata initially contain hyaline asci that become yellow-brown to reddish-brown and eventually develop into tuberculate structures at maturity. Ascospores are multi-septate and verrucose or tuberculate. The asexual morph is characterized by monophialidic phialides and aseptate conidia that are ellipsoidal, oblong, clavate, or allantoid (Lombard et al. 2015; Zeng and Zhuang 2016; Bao et al. 2023). Previous studies have reported more than five *Cosmospora* species from cave environments (Suppl. material 1: table S2). In this study, we introduce *Cosmospora cavernicola* sp. nov., isolated from the rock surface in a cave.

### Phylogenetic analyses: *Cosmospora*

**Figure 16.**
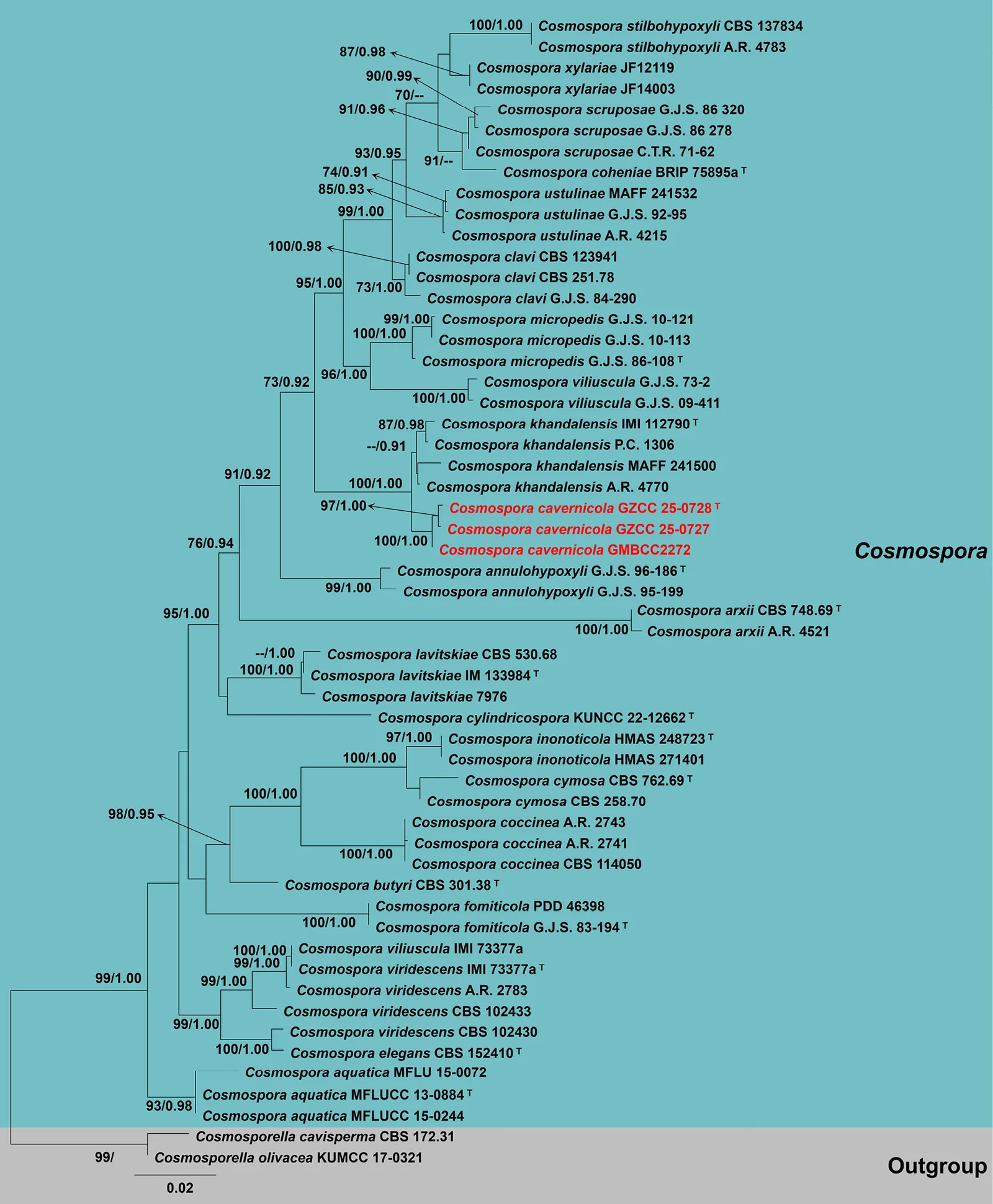
Consensus phylogram of 1,000 trees resulting from a RAxML analysis of the (ITS, LSU, *rpb*2, and *tub*2) alignment of the analyzed *Cosmospora* sequences. The RAxML analysis of the combined dataset yielded the best-scoring tree, with a final ML optimization likelihood value of -12849.664027. This tree was generated from 558, 760, 690, and 562 base pairs of ITS, LSU, *rpb*2, and *tub*2 characters, respectively. The matrix comprises 813 distinct alignment patterns, with 41.73% of the sequences containing gaps and indeterminate traits. Base frequencies were estimated as follows A = 0.235448, C = 0.266594, G = 0.270116, T = 0.227842; substitution rates AC = 1.422656, AG = 5.592205, AT = 1.731116, CG = 1.058597, CT = 10.946369, GT = 1.000000; proportion of invariable sites I = 0.631370; gamma distribution shape parameter α = 1.261061. The trees from both analyses (ML and BI) were identical. RAxML bootstrap support values (ML equal to or above 70%) and Bayesian inference (BYPP equal to or above 0.90) are given at the nodes (ML/BYPP). Newly generated sequences in this study are in blue and red. Type strains are denoted with ^T^. The scale bar represents the expected number of changes per site.

*Cosmospora* phylogeny was based on the combined ITS, LSU, *rpb*2, and *tub*2 sequence data. The aligned dataset encompassed 55 strains, representing 23 taxa, including *Cosmospora* Rabenh. (53 strains, and 21 taxa), and the outgroup *Cosmosporella cavisperma* (CBS 172.31) and *C. olivacea* (KUMCC 17-0321). The tree topologies from the two analyses (ML and BI) were identical. Multi-locus phylogenetic analyses based on ITS, LSU, *rpb*2, and *tub*2 show our new isolates (GZCC 25-0728 (type), GZCC 25-0727, and GMBCC2272) belonging to *Cosmospora* and form a distinct clade sister to *Co. khandalensis* (Thirum. & Sukapure) Gräfenhan & Seifert, with statistical support (ML/BI = 100/1.00) (Fig. 16).

#### Cosmospora cavernicola

Taxon classificationAnimaliaHypocrealesNectriaceae

X.F. Liu, Karun., Tibpromma & K.D. Hyde sp. nov.

EE4A841E-C93F-5EE3-AB6D-42051841B875

Index Fungorum: IF905020

Fig. 17

##### Etymology.

“Caverna” means cave in Latin, and “cola” means dweller or inhabitant, hence the creation of the epithet.

##### Holotype.

GMB-W1537.

##### Description.

Associated with rock surface in the cave environment. Asexual morph on PDA: ***Mycelium*** consisting of branched, septate, hyaline to subhyaline or yellowish-orange in mass, smooth hyphae, 1.1–3.2 µm diam., with occasional anastomoses. ***Conidiophores*** arising laterally or terminally from somatic hyphae, verticillate, up to 230 μm long, branched, hyaline, smooth and thin-walled, bearing terminal and lateral conidiogenous cells, mostly bearing whorls of 2 conidiogenous cells. ***Conidiogenous cells*** 21–92 µm long, 1–4 µm wide at the base, enteroblastic, monophialidic, subulate or subcylindrical, terminal or lateral, discrete, straight, septate, tapering towards the apex, smooth-walled, hyaline, without a distinct periclinal thickening, 0.6–2.2 µm wide at the apex. ***Microconidia*** 3–5 × 1–3 μm (x̄ = 3.85 × 2.97 μm, n = 90), ellipsoidal, cylindrical, slightly allantoid or obovoid to clavate, sometimes flattened on one side, hyaline, smooth- and thin-walled, aseptate. ***Chlamydospores*** not observed. Sexual morph: Undetermined.

**Figure 17.**
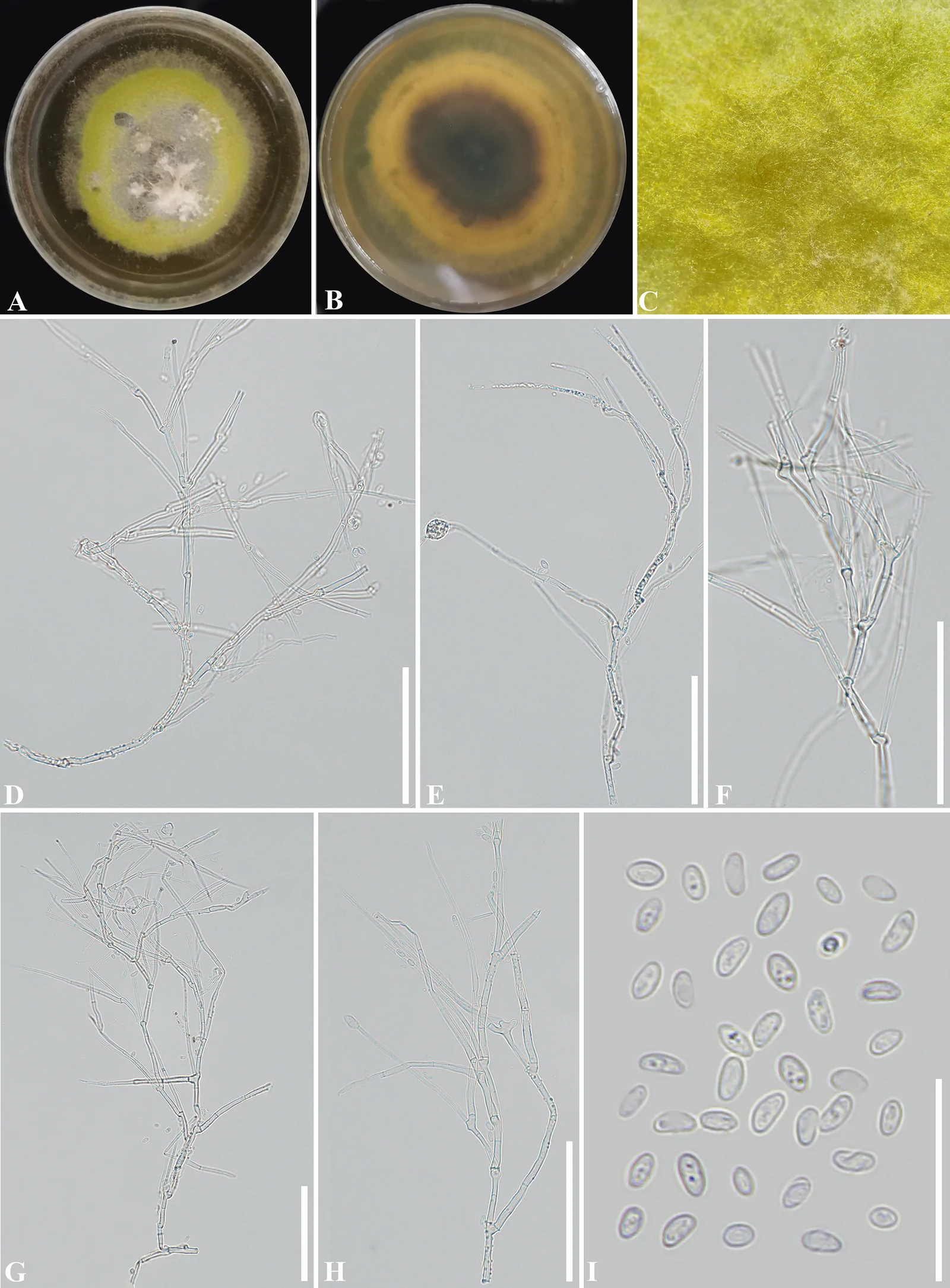
*Cosmospora cavernicola* (GZCC 25-0728, ex-type). **A, B** Colony on PDA (above and below); **C** Sporulation on PDA (35 days old culture); **D–H** Conidiophores, phialides, and conidia; **I** Conidia. Scale bars: 50 μm (**D–H**); 10 μm (**I**).

##### Culture characteristics.

Colonies on PDA attaining 50 mm after 30 days at room temperature (25 ± 2 °C), creamy at the center, becoming yellow toward the outer part of the colony with a white margin, with a fimbriate margin; reverse brown and dark yellow with a light-yellow margin. Odor absent.

##### Material examined.

CHINA • Yunnan Province, Dehong Dai and Jingpo Autonomous Prefecture, Mang City, Mengga Town, Sanxian Cave, rock surface, 24.232220°N, 98.426103°E, elev. ca 1528.89 m, 23 April 2024, Xiangfu Liu and Xuemei Chen, MS25D (GMB-W1537, holotype), ex-type GZCC 25-0728; *ibid*., MS25B2 (GMB-W1243, isotype), ex-isotype GMBCC2272, *ibid*., MS25C (GMB-W1244, isotype), ex-isotype GZCC 25-0727.

##### Note.

In the multi-gene phylogenetic analyses of the combined ITS, LSU, *rpb*2, and *tub*2 regions, *Cosmospora cavernicola* (GZCC 25-0728 (ex-type), GMBCC2272 and GZCC 25-0727) formed a distinct lineage to *Co. khandalensis* (Thirum. & Sukapure) Gräfenhan & Seifert with 100% ML and 1.00 PP support (Fig. 16). However, the nucleotide comparisons between *Co. cavernicola* (GZCC 25-0728, ex-type) and *Co. khandalensis* (IMI 112790, ex-type) showed 1.61% (18/544 bp, 2 gaps) base pair differences in ITS, 0.13% (1/788 bp, without gap) differences in LSU, 2.20% (7/318 bp, without gap) differences in *tub*2, and 0.81% (7/867 bp, without gap) differences in *rpb*2. Morphologically, *Cosmospora cavernicola* can be easily distinguished from *Co. khandalensis* by its verticillate conidiophores and longer, septate conidiogenous cells (21–92 µm vs. 34–64 µm) (Herrera et al. 2015). In addition, the PHI test results (Fig. 6C) revealed no significant recombination relationships between *C. cavernicola* and its phylogenetically related taxa within the dataset. This fungus is, therefore, introduced as a new species of *Cosmospora*.

#### *Microascales* Luttr. ex Benny & R.K. Benj., Mycotaxon 12(1): 40 (1980)

##### Microascaceae

Taxon classificationAnimaliaMicroascalesMicroascaceae

Luttr. ex Malloch, Mycologia 62(4): 734 (1970)

F73EE262-BBCB-507D-A6FB-042ED9AA6B74

###### Notes.

*Microascaceae* was established by Malloch (1970) and currently comprises a morphologically heterogeneous assemblage of fungi, including saprobic and plant-pathogenic species, as well as several opportunistic human pathogens that exhibit intrinsic resistance to antifungal agents (Lackner et al. 2014; Sandoval-Denis et al. 2016). Members of this family are primarily characterized by asexual morphs that typically possess annellidic conidiogenous cells and produce dry, aseptate conidia. The sexual morphs form cleistothecial or perithecial, carbonaceous ascomata bearing distinctive reniform, lunate, or triangular ascospores, which may or may not possess germ pores (Sandoval-Denis et al. 2016). To date, 79 species belonging to 20 genera of *Microascaceae* have been reported from caves worldwide (Suppl. material 1: table S2).

##### Pithoascus

Taxon classificationAnimaliaMicroascalesMicroascaceae

Arx, Proc. K. Ned. Akad. Wet., Ser. C, Biol. Med. Sci. 76(3): 295 (1973)

8D10EF67-01BC-527E-8E9C-4BA73FB98F8E

###### Notes.

*Pithoascus* was introduced by Arx (1973) and typified by *P. nidicola*. Species of *Pithoascus* have been reported from various habitats, including plants, soil, human skin, and nails (Tazik et al. 2020). The sexual morph is characterized by globose ascomata, gregarious, immersed or superficial, often aggregated on dense crusts. The peridium is black and composed of thick-walled, flattened cells (*textura angularis*), with an inconspicuous ostiole or a short neck. Asci are unitunicate, 8-spored, and broadly clavate to barrel-shaped. Ascospores are unicellular, asymmetrical (navicular, fusiform, or falcate), yellow to honey-colored, and dextrinoid when young (Arx 1973; Jagielski et al. 2016; Sandoval-Denis et al. 2016). The asexual morph is characterized by annellidic conidiogenous cells borne laterally on hyphae, producing solitary, unicellular, globose to pyriform conidia with a truncate base (Sandoval-Denis et al. 2016). The genus currently comprises six species, and phylogenetic analyses based on ITS, LSU, and *tef*1-α sequences indicate considerable genetic distance among *P. ater*, *P. exsertus*, *P. lunatus*, *P. nidicola*, *P. persica*, and *P. stoveri* (Tazik et al. 2020). In the present study, we introduce *Pithoascus cavernicola* as a new species, representing the third known species of *Pithoascus* isolated from cave environments, following *P. ater* (Zach) Sand.-Den., Cano & Guarro and *P. platysporus* Arx & Veenb.-Rijks (Suppl. material 1: table S2).

### Phylogenetic analyses: *Pithoascus*.

*Pithoascus* phylogeny was based on the combined ITS, LSU, *tef*1-α, and *tub*2 sequence data. The aligned dataset encompassed 91 strains, representing 47 taxa, including *Gamsia* M. Morelet (two strains, two taxa), *Microascus* Zukal (48 strains, 21 taxa), *Petriella* Curzi (one strain, one taxon), *Petriellopsis* Gilgado, Cano, Guarro & Gené (one strain, one taxon), *Pithoascus* Arx (ten strains, eight taxa), *Pseudoscopulariopsis* Sand.-Den., Gené & Guarro (two strains, two taxa), *Scopulariopsis* Bainier (24 strains, nine taxa), *Scedosporium* Sacc. ex Castell. & Chalm. (two strains, two taxa), *Wardomycopsis* Udagawa & Furuya (two strains, two taxa), and the outgroup taxon *Trichoderma atroviride* P. Karst. (CBS 142 95). The tree topologies from the two analyses (ML and BI) were identical. Multi-gene phylogenetic analyses based on the combined ITS, LSU, *tef*1-α, and *tub*2 analyses indicate that our new isolates (GMBCC2294 and GMBCC2476) belong to *Pithoascus* and form a distinct clade with 85% ML bootstrap support and 1.00 Bayesian posterior probability.

#### Pithoascus cavernicola

Taxon classificationAnimaliaMicroascalesMicroascaceae

X.F. Liu, Karun., Tibpromma & K.D. Hyde sp. nov.

DD513932-F250-5309-AF49-5F939073BA32

Index Fungorum: IF905022

Fig. 19

##### Etymology.

“Caverna” means cave, and “cola” means dweller or inhabitant in Latin; thus, “cavernicola” refers to the cave habitat of the holotype.

##### Holotype.

GMB-W1482.

##### Description.

Associated with rock surface in the cave. Asexual morph on PDA: ***Vegetative hyphae*** 1–2.5 μm wide, branched, hyaline, septate, smooth-walled. ***Conidiophores*** arising from substrate, mycelium simple to indistinctive. ***Conidiogenous cells*** 2–7 × 1–4 µm (x̄ = 4.648 × 2.34 μm, n = 30), hyaline, cylindrical to ampulliform, reduced to short swollen supporting cells, annellidic, septate, smooth, thin-walled, born singly, often laterally and rarely apically on aerial hyphae. ***Conidia*** 3–6 × 2–5 µm (x̄ = 4.62 × 3.77 μm, n = 60), globose to pyriform, with a truncate base, hyaline to pale brown, smooth and thin-walled, aseptate, arranged in chains, with a disjuncture between adjacent conidia. ***Chlamydospores*** not observed. Sexual morph: Undetermined.

##### Culture characteristics.

Colonies on PDA are 35–45 mm diam in 14 d at room temperature (25 ± 2 °C), flat, felty to floccose, and pale gray near the center, with a pale brown irregular margin; the reverse is pale brown near the center and white near the margin.

##### Material examined.

CHINA • Yunnan Province, Honghe Hani and Yi Autonomous Prefecture, Jianshui County, Miandian Town, Swallow Cave, rock surfaces, 26.080743°N, 98.848391°E, elev. ca 1343 m, 24 December 2020, Xiangfu Liu, YZD14C (GMB-W1482, holotype), ex-type GMBCC2294; *ibid*., YZD14C2 (GMB-W1258, isotype), ex-isotype GMBCC2476.

##### Notes.

**Figure 18.**
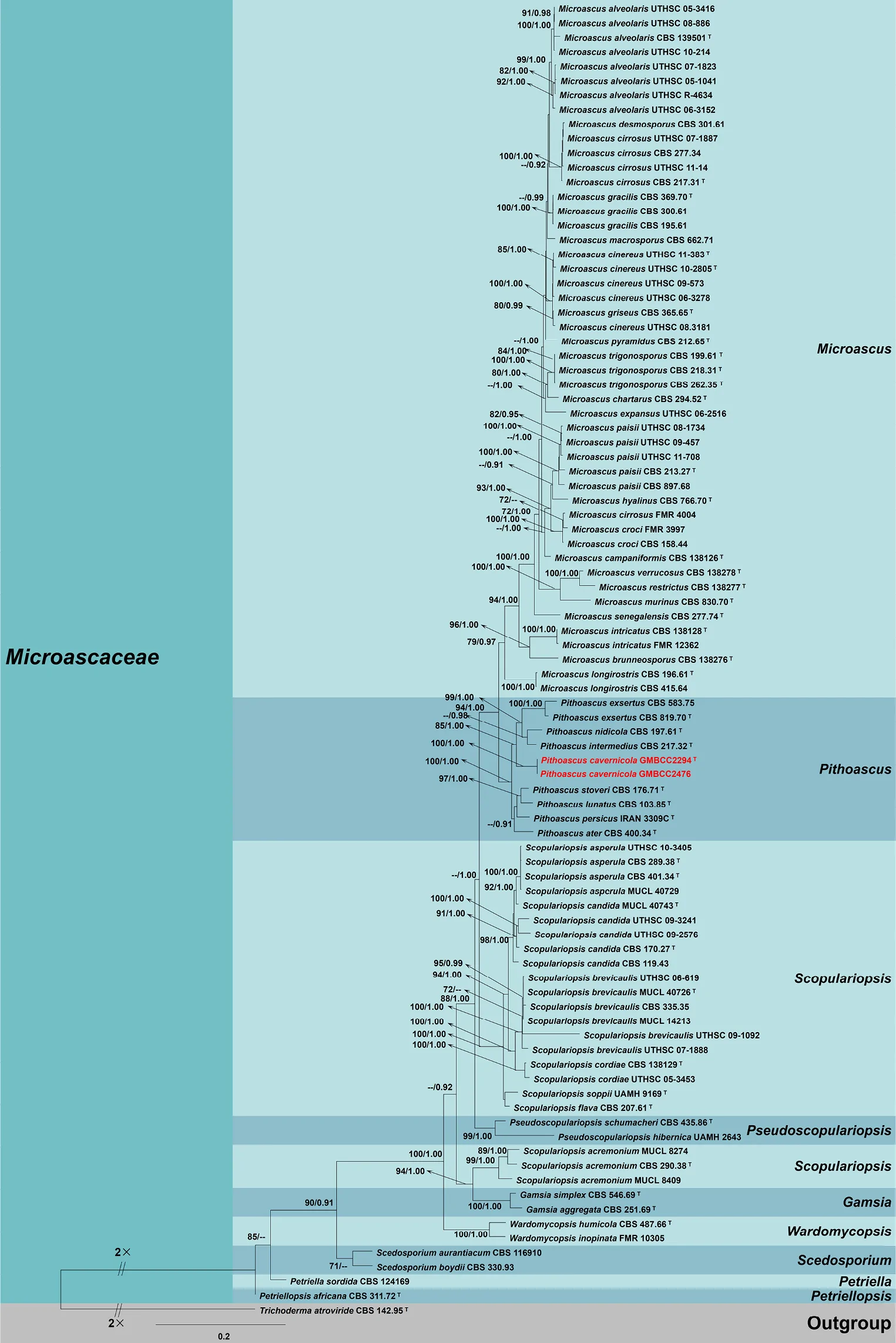
Consensus phylogram of 1,000 trees resulting from a RAxML analysis of the (ITS, LSU, *tef*1-α, and *tub*2) alignment of the analyzed *Pithoascus* sequences. The RAxML analysis of the combined dataset yielded the best-scoring tree, with a final ML optimization likelihood value of -22169.941997. This tree was generated from 639, 583, 950, and 414 base pairs of ITS, LSU, *tef*1-α, and *tub*2 characters, respectively. The matrix comprises 1,205 distinct alignment patterns with 13.01% of gaps and indeterminate traits. Base frequencies were estimated as follows A = 0.213314, C = 0.304675, G = 0.278843, T = 0.203169 with substitution rates AC = 0.902039, AG = 1.725203, AT = 1.373249, CG = 0.985292, CT = 4.374981, GT = 1.000000; proportion of invariable sites I = 0.191583; gamma distribution shape parameter α = 0.445769. The trees from both analyses (ML and BI) showed identical topologies. RAxML bootstrap support values (ML equal to or above 70%) and Bayesian inference (BYPP equal to or above 0.90) are given at the nodes (ML/BYPP). The sequences of new strains are in red. Type strains are denoted with ^T^. The scale bar represents the expected number of changes per site.

The new species was identified as a member of *Pithoascus* based on combined morphological and phylogenetic evidence. In our phylogenetic analyses of combined ITS, LSU, *tef*1-α, and *tub*2, *Pithoascus cavernicola* (GMBCC2294 and GMBCC2476) formed a distinct lineage with 85% ML and 1.00 PP support (Fig. 18). Pairwise nucleotide comparisons between *P. cavernicola* (GMBCC2294, ex-type) and closely related species revealed significant sequence divergence. *Pithoascus cavernicola* differs from *P. ater* (CBS 400.34, ex-type) by 6.72% (32/476 bp, 13 gaps) base pair differences in ITS, 2.03% (11/543 bp, 1 gap) differences in LSU, and 5.57% (53/952 bp, 4 gaps) differences in *tef*1-α. Comparisons with *P. exsertus* (CBS 81 9.70, ex-type) showed 9.57% (51/533 bp, 18 gaps) base pair differences in ITS, 1.49% (8/536 bp, without gap) differences in LSU, and 7.18% (69/961 bp, 5 gaps) differences in *tef*1-α. *Pithoascus cavernicola* (GMBCC2294, ex-type) differs from *P. intermedius* (CBS 217.32, ex-type) by 5.44% (29/533 bp, 19 gaps) base pair differences in ITS, 1.50% (8/535 bp, 1 gap) differences in LSU, and 6.26% (60/959 bp, 4 gaps) differences in *tef*1-α.

**Figure 19.**
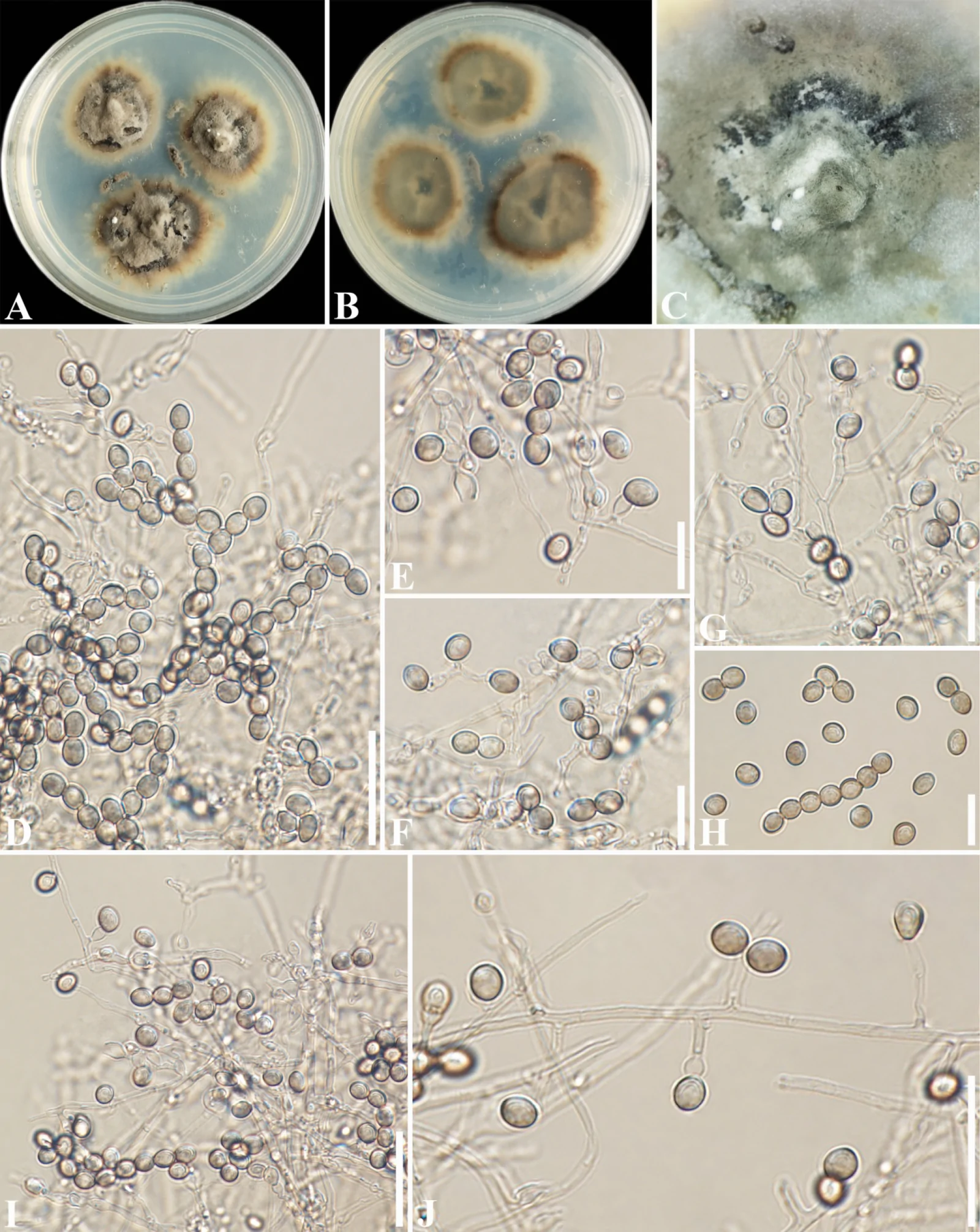
*Pithoascus cavernicola* (GMBCC2294, ex-type). **A, B** Colony on PDA (above and below); **C** Conidiophores arise from the colony (60 days old culture); **D–F, I, J** Conidiophores, annellides and conidia; **H** Conidia. Scale bars: 20 μm (**D, I, J**); 10 μm (**E–H**).

Sequence comparisons with *P. kurdistanensis* (IRAN 4653C, type) showed 5.22% (50/958 bp, 4 gaps) base pair differences in *tef*1-α. *Pithoascus cavernicola* (GMBCC2294, ex-type) differs from *P. lunatus* (CBS 103.85, ex-type) by 6.83% (20/293 bp, 7 gaps) base pair differences in ITS, 1.32% (11/831 bp, 1 gap) differences in LSU, and 6.36% (64/1006 bp, 6 gaps) differences in *tef*1-α. *Pithoascus cavernicola* (GMBCC2294, ex-type) differs from *P. persicus* (IRAN 3309C, ex-type) by 9.19% (49/533 bp, 34 gaps) base pair differences in ITS, 2.46% (20/812 bp, 2 gaps) differences in LSU, and 6.46% (48/743 bp, 3 gaps) differences in *tef*1-α. *Pithoascus cavernicola* (GMBCC2294, ex-type) and *P. stoveri* (CBS 176.71, ex-type) sequences showed 11.44% (61/533 bp, 35 gaps) base pair differences in ITS, 2.43% (13/536 bp, 3 gaps) base pair differences in LSU, and 5.31% (51/961 bp, 4 gaps) base pair differences in *tef*1-α.

Morphologically, *P. cavernicola* differs from *P. exsertus* and *P. lunatus* in the characteristics of its asexual morph. It differs from *P. ater* by the latter producing solitary, globose, and larger conidia (3–6 × 2–5 µm vs. 4–9 × 4.5–8.5 µm) (Sandoval-Denis et al. 2016). *Pithoascus cavernicola* differs from *P. intermedius* in that the latter has solitary, hyaline, and larger (3–6 × 2–5 µm vs. 4–8 × 4.5–7.5 µm) conidia, whereas those of *P. cavernicola* are arranged in conidia (Mohammadi et al. 2024). *Pithoascus cavernicola* differs from *P. kurdistanensis* by the latter having cylindrical to ampulliform conidiogenous cells, rarely reduced to short swollen supporting cells, slightly smaller (3–6 × 2–5 µm vs 3.5–5 × 3.5–4 µm) conidia, and setae, compared to conidia of *P. cavernicola* that are arranged in chains (Mohammadi et al. 2024). *Pithoascus cavernicola* differs from *P. persicus* in that the latter has annellidic, short, ampulliform conidiogenous cells, and smaller (3–6 × 2–5 µm vs. 2.5 µm in diameter) globose conidia (Tazik et al. 2020). *Pithoascus cavernicola* differs from *P. stoveri* in that the latter has annellidic, short, ampulliform conidiogenous cells, and hyaline, obovate to pyriform conidia that are larger (3–6 × 2–5 µm vs 5–8 × 3–4 µm) (Sandoval-Denis et al. 2016; Mohammadi et al. 2024). In addition, the PHI test results (Fig. 6D) revealed no significant recombination relationships between *P. cavernicola* and its phylogenetically related taxa within the dataset. The phylogenetic and morphological results indicate that *P. cavernicola* is a distinct new species.

#### *Sordariales* Chadef. ex D. Hawksw. & O.E. Erikss., Syst. Ascom. 5(1): 182 (1986)

##### Chaetomiaceae

Taxon classificationAnimaliaSordarialesChaetomiaceae

G. Winter [as ‘ Chaetomieae’], Rabenh. Krypt.-Fl., Edn 2 (Leipzig) 1.2: 153 (1885)

3F6FC342-3AD1-5851-BA10-9B906C43ACD3

###### Notes.

*Chaetomiaceae* was established by Winter (1885), with *Chaetomium* as the type genus. Members of this family are cosmopolitan and occur mainly as saprobes on substrates such as dung, seeds, paper, plant debris, soil, air, and wood, but may also be endophytic, parasitic, mycoparasitic, or opportunistic human pathogens (Mehrabi et al. 2020). The family is characterized by ostiolate or non-ostiolate ascomata that are solitary or gregarious, superficial or immersed, and often covered with hairs or setae. Asci are clavate to cylindrical, pedicellate, and unitunicate, containing 4–8 ascospores; ascospores are brown to black at maturity, opaque, aseptate, thick-walled, smooth, and exhibit various shapes such as ellipsoidal, globose, subglobose, oval, fusiform, or triangular, usually with one and occasionally two germ pores (Maharachchikumbura et al. 2016). Numerous asexual morphs are associated with *Chaetomiaceae*, including *Acremonium*-, *Botryotrichum*-, *Chrysonilia*-, *Chrysosporium*-, *Humicola*-, *Myceliophthora*-, *Scytalidium*-, and *Trichocladium*-like forms (Mehrabi et al. 2020). To date, 49 species across 19 genera have been reported from caves (Suppl. material 1: table S2).

### Phylogenetic analyses: *Chaetomiaceae*

**Figure 20.**
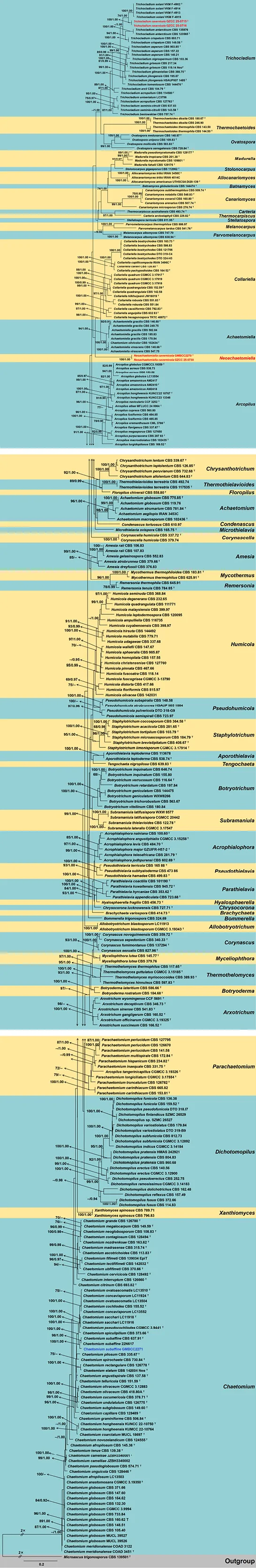
Consensus phylogram of 1,000 trees resulting from a RAxML analysis of the (ITS, LSU, *rpb*2, and *tub*2) alignment of the analyzed *Chaetomiaceae* sequences. The RAxML analysis of the combined dataset yielded the best-scoring tree, with a final ML optimization likelihood value of -12849.664027. This tree was generated from 558, 760, 690, and 562 base pairs of ITS, LSU, *rpb*2, and *tub*2 characters, respectively. The matrix comprises 813 distinct alignment patterns, with 41.73% of the sequences containing gaps and indeterminate traits. Base frequencies were estimated as follows A = 0.235448, C = 0.266594, G = 0.270116, T = 0.227842; substitution rates AC = 1.422656, AG = 5.592205, AT = 1.731116, CG = 1.058597, CT = 10.946369, GT = 1.000000; proportion of invariable sites I = 0.631370; gamma distribution shape parameter α = 1.261061. The tree topologies from both analyses (ML and BI) were identical. RAxML bootstrap support values (ML equal to or above 70%) and Bayesian inference (BYPP equal to or above 0.90) are given at the nodes (ML/BYPP). Newly generated sequences in this study are in red. Type strains are denoted with ^T^. The scale bar represents the expected number of changes per site.

*Chaetomiaceae* phylogeny was based on the combined ITS, LSU, *rpb*2, and *tub*2 sequence data. The aligned dataset encompassed 310 strains, representing 233 taxa, including *Achaetomiella* (seven strains, two taxa), *Achaetomium* (five strains, four taxa), *Acrophialophora* (six strains, six taxa), *Chaetomium* (48 strains, 21 taxa), *Canariomyces* (ten strains, seven taxa), *Trichocladium* (27 strains, one taxon), *Petriellopsis* Gilgado, Cano, Guarro & Gené (one strain, one taxon), *Pithoascus* Arx (ten strains, eight taxa), *Pseudoscopulariopsis* Sand.-Den., Gené & Guarro (two strains, two taxa), *Scopulariopsis* Bainier (24 strains, nine taxa), *Scedosporium* Sacc. ex Castell. & Chalm. (two strains, two taxa), *Wardomycopsis* Udagawa & Furuya (two strains, two taxa), and the outgroup taxon *Microascus trigonosporus* (CBS 139501). Fifty-one genera in *Chaetomiaceae* formed well-separated clades in the phylogenetic tree, and each genus formed separate branches (Fig. 20). *Chaetomium olivicolor* is phylogenetically related to *Achaetomiella*, and its taxonomic placement requires further research, as suggested by Wang et al. (2022). Multi-locus phylogenetic analyses based on the combined ITS, LSU, *rpb*2, and *tub*2 datasets indicate that our new isolates (GZCC 25-0715 (ex-type) and GZCC 25-0716) belong to *Trichocladium* and form a distinct clade with 74% ML and 1.00 PP support. Another new isolate (GMBCC2270, ex-type) forms a distinct branch positioned between *Achaetomiella* and *Arcopilus* and is therefore introduced as a new genus, *Neoachaetomiella*, typified by *Neoachaetomiella cavernicola* sp. nov. The remaining isolate (GMBCC2271) clusters with *Chaetomium subaffine* within *Chaetomium*.

#### Neoachaetomiella

Taxon classificationAnimaliaSordarialesChaetomiaceae

X.F. Liu, Karun., Tibpromma & K.D. Hyde gen. nov.

8A34F430-A8CB-52B0-891E-9B1A9AC80AD1

Index Fungorum: IF905023

Fig. 21

##### Etymology.

Named after its morphological similarity to *Achaetomiella*.

##### Type species.

*Neoachaetomiella cavernicola* X.F. Liu, Karun., Tibpromma & K.D. Hyde.

##### Description.

Associated with rock surfaces of caves. Sexual morph: ***Ascomata*** superficial, subglobose or ovate, with brown walls of *textura angularis* in surface view, ostiolate. ***Terminal hairs*** flexuous, undulate, or spirally coiled. *Lateral hairs* straight, flexuous. ***Asci*** clavate or fusiform, stalked, evanescent. ***Ascospores*** biseriate or irregularly arranged, olivaceous to olivaceous brown at maturity, limoniform or fusiform, bilaterally flattened, with an apical germ pore, usually more than 7 μm and less than 15 μm in length. Asexual morph: not observed.

**Figure 21.**
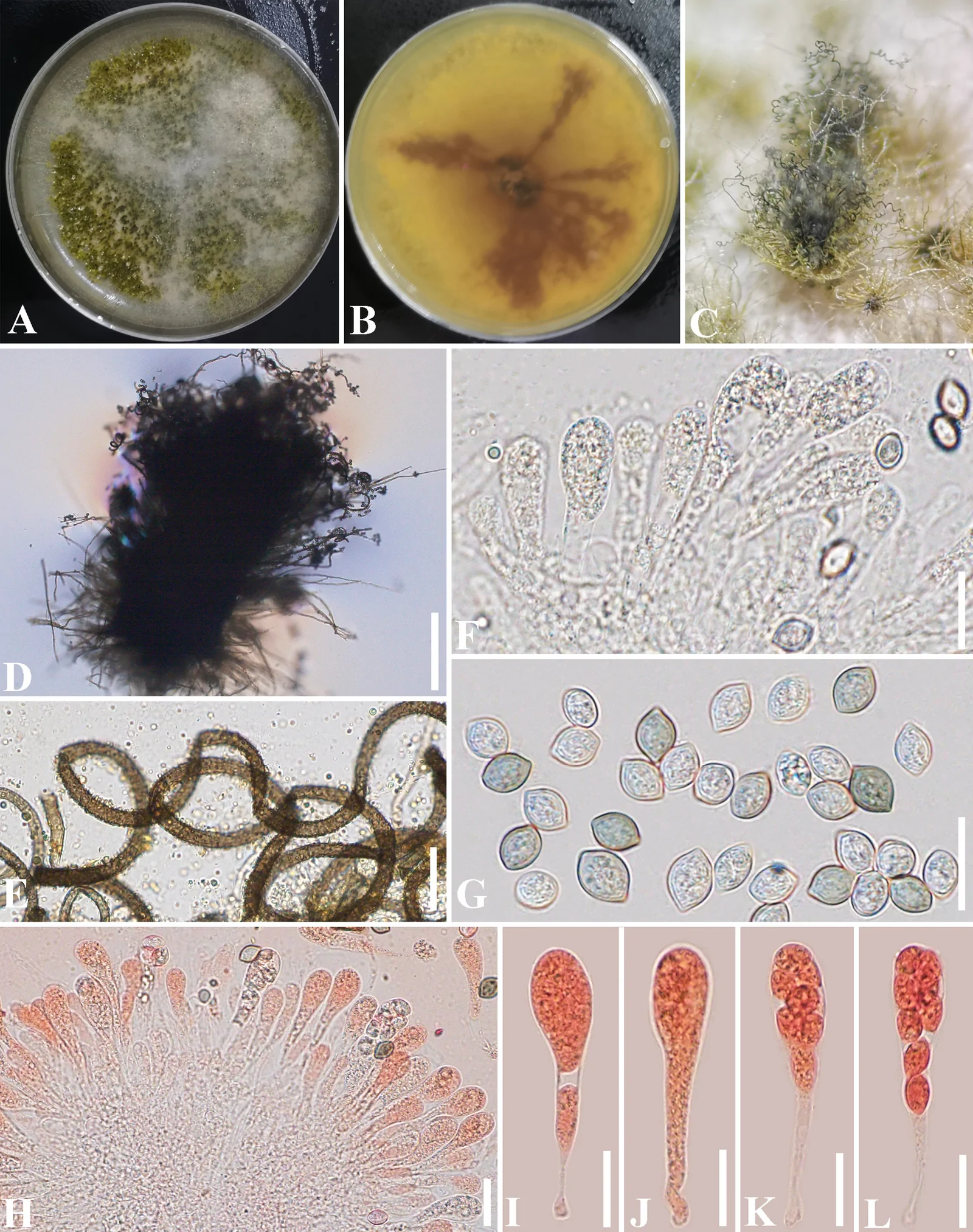
*Neoachaetomiella cavernicola* (GMBCC2270, ex-type) **A, B** Colony on PDA (above and below); **C, D** Mature ascomata on PDA; **E** Ascomatal hairs; **G** Ascospores; **F, H–L** Asci. **H–L** Stained with 1% Congo red solution. Scale bars: 200 μm (**D**); 10 μm (**E–L**).

##### Notes.

Based on phylogenetic analyses, our strains formed an independent lineage well separated from *Achaetomiella* Arx and *Collariella* X. Wei Wang, Samson & Crous (Fig. 20). *Neoachaetomiella* can be distinguished from *Achaetomiella* and *Collariella* by its olivaceous to olivaceous-brown, limoniform to fusiform, bilaterally flattened ascospores, which possess an apical germ pore and measure typically 7–15 μm in length (Wang et al. 2016a, 2022). *Achaetomiella* consists of two species (*Achaetomiella gracilis* and *A. virescens*) with sexual morphs, producing brown, ellipsoidal or fusiform ascospores, usually longer than 9 μm compared to more than 7 μm and less than 15 μm of *Neoachaetomiella* (Wang et al. 2022). In contrast, *Collariella*, which includes 12 species with sexual morphs, has broadly limoniform to quadrangular, olivaceous-brown ascospores, typically less than 7.5 μm in length, occasionally up to 10.5 μm, compared with more than 7 μm and less than 15 μm in *Neoachaetomiella* (Wang et al. 2022). Megablast searches of GenBank revealed that the closest hits to *Neoachaetomiella* ITS sequences showed 93.79% similarity; LSU sequences showed 99.50% similarity; *rpb*2 sequences had 84.27% similarity, and *tub*2 sequences showed 81.78% similarity, supporting its recognition as a distinct genus.

#### Neoachaetomiella cavernicola

Taxon classificationAnimaliaSordarialesChaetomiaceae

X.F. Liu, Karun., Tibpromma & K.D. Hyde sp. nov.

A5503B5F-EC04-5EB7-B7F8-FAB188F9000B

Index Fungorum: IF905024

Fig. 21

##### Etymology.

“Caverna” means cave in Latin, and “cola” means dweller or inhabitant, hence the creation of the epithet.

##### Holotype.

GMB-W1532.

##### Description.

Associated with rock surface in the cave. Sexual morph on PDA: ***Ascomata*** 300–600 × 150–350 μm, usually covered by thick aerial hyphae, olivaceous or umber to dark-brick in reflected light owing to ascomatal hairs, obovate or ovate, ostiolate. ***Terminal hairs*** errucose, brown, erect to flexuous or slightly undulate, usually unbranched, 2–6 μm near the base, tapering towards the tips. ***Lateral hairs*** are similar. ***Asci*** 38–80 × 6–18 μm (x̄ = 56.06 × 12.34, n = 60), 8-spored, fasciculate, fusiform or clavate, evanescent. ***Ascospores*** 7–13 × 5–9 μm (x̄ = 9.58 × 7.09, n = 150), biseriate, hyaline to olivaceous brown at maturity, limoniform, some globose, verruculose, thick-walled, biapiculate, bilaterally flattened, sometimes with distinct oil droplets and an apical germ pore. Asexual morph: Undetermined.

##### Culture characteristics.

Colony on PDA attaining 50 mm after 15 days at room temperature (25 ± 2 °C), folded, white to light yellow, margin undulate to erose to fimbriate. Ascomata arise from the colony, yellowish-brown after 50 days. Odor absent.

##### Material examined.

CHINA • Yunnan Province, Dehong Dai and Jingpo Autonomous Prefecture, Mang City, Mengga Town, Sanxian Cave, on rock surface, 24.232220°N, 98.426103°E, elev. ca 1528.89 m, 23 April 2024, Xiangfu Liu and Xuemei Chen, MS41D (GMB-W1532, holotype), ex-type GMBCC2270, *ibid*., MS41D2 (GMB-W1533, isotype), ex-isotype GZCC 25-0730.

##### Notes.

In the multi-gene phylogenetic analyses of the combined ITS, LSU, *rpb*2, and *tub*2 regions, *Neoachaetomiella cavernicola* (GMBCC2270 and GZCC 25-0730) formed a distinct lineage, well separated from *Achaetomiella* and *Collariella* (Fig. 20). Morphologically, *N. cavernicola* is similar to these genera but is distinguished by olivaceous to olivaceous-brown, limoniform to fusiform, bilaterally flattened ascospores, with an apical germ pore, typically 7–15 μm in length. In contrast, *Achaetomiella* is characterized by simple ascomatal hairs evenly distributed over the ascoma, and ellipsoidal or fusiform ascospores with a length usually more than 9 μm, and *Collariella* is characterized by broadly limoniform to quadrangular, bilaterally flattened ascospores with an apical germ pore, with a length generally less than 7.5 μm (Wang et al. 2022). In addition, the PHI test results (Fig. 6E) revealed no significant recombination relationships between *N. cavernicola* and its phylogenetically related taxa within the dataset. Herein, we introduce *Neoachaetomiella cavernicola* as a new species based on both morphological comparison and phylogenetic analyses.

#### Chaetomium

Taxon classificationAnimaliaSordarialesChaetomiaceae

Kunze, in Kunze & Schmidt, Mykologische Hefte (Leipzig) 1: 15 (1817)

5B09CBBD-C7FA-5E55-8476-D5053AECF2D9

##### Notes.

*Chaetomium* was introduced by Kunze and Schmidt (1817), typified by *Chaetomium globosum* Kunze (Wijayawardene et al. 2017), is currently the largest genus in *Chaetomiaceae* (Hyde et al. 2020), comprising 359 accepted species, as outlined in the latest update by Wijayawardene et al. (2022), and 449 epithets are recorded in Index Fungorum (http://www.indexfungorum.org; accessed date 14 July 2026). Members of this genus live in plants, soil, straw, and dung as saprobic fungi worldwide, and some have potential as biological control agents (Ahmed et al. 2012; Mungai et al. 2012; Maharachchikumbura et al. 2015, 2016; Réblová et al. 2016; Wang et al. 2016a, 2016b; Wijayawardene et al. 2017). The genus is characterized by globose, ellipsoid to ovate or obovate, ostiolate ascomata with hypha-like, flexuous ascomatal hairs, limoniform to globose, bilaterally flattened ascospores, and asexual morphs that are hyphomycetous and acremonium-like (Wang et al. 2016a; Hyde et al. 2020). To date, over nine *Chaetomium* species have been reported from various cave substrates (Suppl. material 1: table S2). In this study, we report a new substrate record of *Chaetomium* species from caves in Yunnan Province, China.

#### Chaetomium subaffine

Taxon classificationAnimaliaSordarialesChaetomiaceae

Sergeeva ex X. Wei Wang & Houbraken, in Wang, Han, Bai, Luo, Bensch, Meijer, Kraak, Han, Sun, Crous & Houbraken, Stud. Mycol. 101: 167 (2022)

F2F83164-46DE-5514-8785-041F863F60DC

Index Fungorum: IF 842311

Fig. 22

##### Description.

Associated with decaying twigs in the cave environment. Sexual morph on PDA: ***Ascomata*** 200–400 × 150–350 μm, obovate or ovate, ostiolate, usually covered by thick aerial hyphae, olivaceous or umber to dark-brick in reflected light owing to ascomatal hairs. ***Terminal hairs*** verrucose, brown, erect to flexuous or slightly undulate, usually unbranched, 2–4 μm near the base, tapering towards the tips. ***Lateral hairs*** similar. ***Asci*** 8-spored, fasciculate, fusiform or clavate, spore-bearing part 42–63 × 7–17 μm (x̄ = 52.86 × 11.76, n = 30), evanescent. ***Ascospores*** 9–12 × 8–10 μm (x̄ = 11.28 × 9.19, n = 90), biseriate, hyaline to olivaceous brown with age, limoniform, some globose, verruculose, thick-walled, biapiculate, bilaterally flattened, with an apical germ pore. Asexual morph: Undetermined.

**Figure 22.**
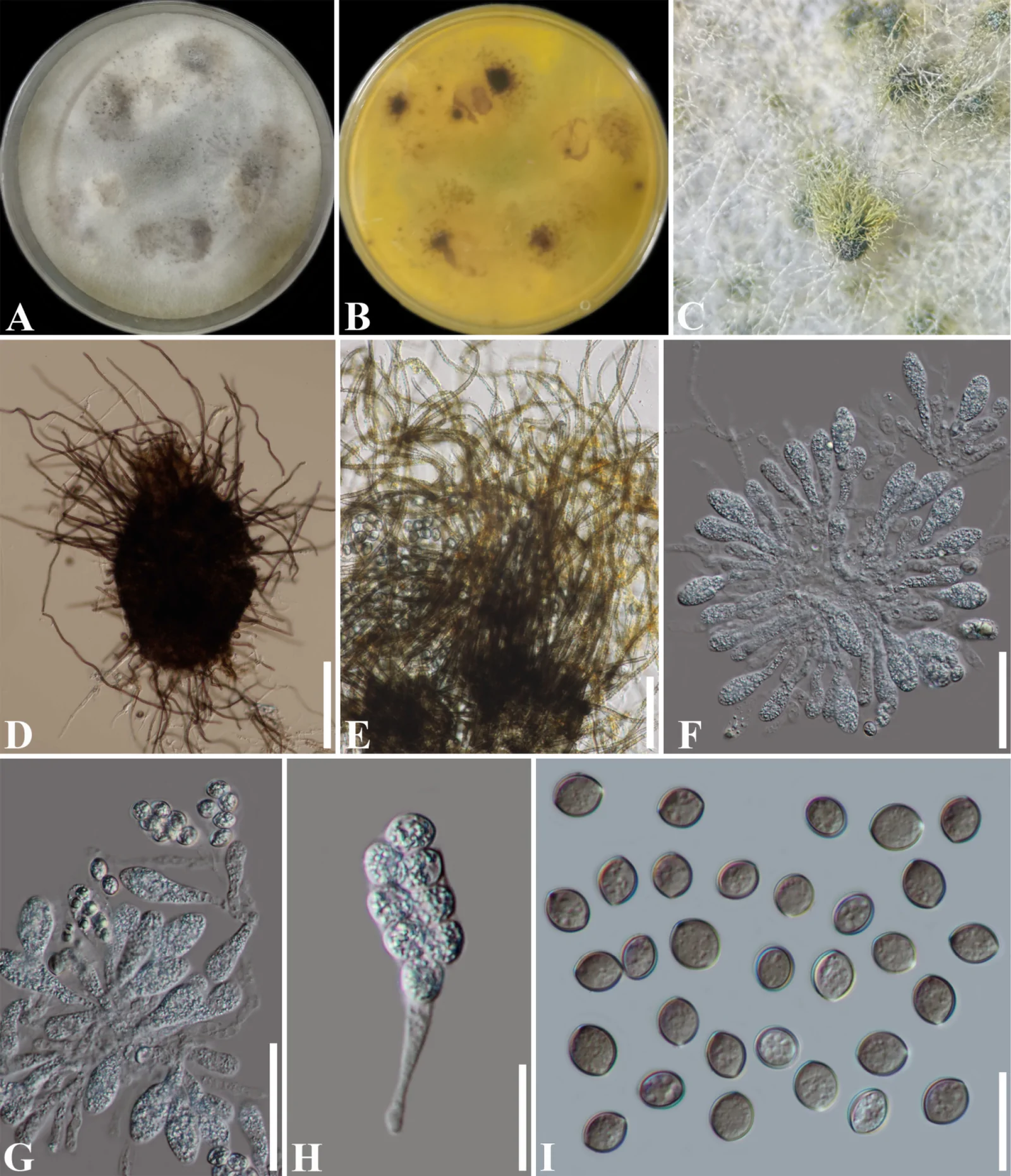
*Chaetomium subaffine* (GMBCC2271, new substrate record in cave habitat) **A, B** Colony on PDA (above and below); **C, D** Mature ascomata on PDA; **E** Terminal ascomatal hairs; **F–H** Asci and ascospores; **I** Ascospores. Scale bars: 100 μm (**D**), 50 μm (**E–G**), 20 μm (**H, I**).

##### Culture characteristics.

Colony on PDA attaining 60 mm after 15 days at room temperature (25 ± 2 °C), circular, folded, white to light yellow, with slightly entire edge, sporulated within 30 days. Reverse yellow to yellow brown. Odor absent.

##### Material examined.

CHINA • Yunnan Province, Qujing City, Malong District, Wangjiazhuang Street, Y008, Longyangxian Cave, associated with decaying twigs on the floor of the cave, 25.434721°N, 103.509524°E, elev. ca 1920.43 m, 29 April 2024, Xiangfu Liu and Xuemei Chen, QJ2C2 (GMB-W1252), living culture GMBCC2271.

##### Known distribution.

China (Wang et al. 2016b; Farr and Rossman 2026), Netherlands (Wang et al. 2016b), and Russia (Wang et al. 2016b, 2022).

##### Known hosts or substrata.

*Castanea mollissima* (Farr and Rossman 2026), *Glycine max* (Farr and Rossman 2026), *Homo sapiens* (Wang et al. 2016b), *Morus alba* (Farr and Rossman 2026), unknown plant stem (Wang et al. 2016b), seed and dead stem of cereal (Wang et al. 2022).

##### Notes.

*Chaetomium subaffine* was first described by Sergeeva (1961), but, lacking a Latin description, it was considered invalid. Then, Wang et al. (2022) reintroduced the species. In the phylogenetic analyses of the combined ITS, LSU, *rpb*2, and *tub*2 data, our isolates clustered with *Chaetomium subaffine* (CBS 637.91 (ex-type), and 22N617) within the *Chaetomium* clade, with 100% ML and 1.00 PP support (Fig. 20). Nucleotide comparisons between our strain (GMBCC2271) and *C. subaffine* (CBS 637.91, ex-type) showed 1.73% (10/578 bp, 1 gap) differences in ITS, 1.65% (10/607 bp, 1 gap) differences in LSU, 2.19% (11/503 bp, without gap) differences in *rpb*2, and 0.99% (7/704 bp, without gap) differences in *tub*2. Morphologically, the new isolate (GMBCC2271) resembles *C. subaffine*, with obovate or ovate ascomata usually covered by thick aerial hyphae, brown, erect to flexuous or slightly undulate terminal hairs, and brown, limoniform, biapiculate, bilaterally flattened ascospores (Wang et al. 2016b). Therefore, based on both morphology and phylogeny, we report *Chaetomium subaffine* as a new substrate record from a cave in Yunnan, China.

#### Trichocladium

Taxon classificationAnimaliaMonhysteridaChaetomiaceae

Harz, Bull. Soc. Imp. nat. Moscou 44(1): 125 (1871)

53DF9E5E-6641-537F-A83A-2103065CD368

##### Notes.

*Trichocladium* was introduced by Harz (1871), with *Trichocladium asperum* as the type species. Species of *Trichocladium* have been isolated from a wide range of substrates, including soil, estuarine sediments, leaves, dung, grass, meal, and *Usnea cf. aurantio*-*atra* (Wang et al. 2019). According to Index Fungorum (http://www.indexfungorum.org; accessed date 14 July 2026), the genus currently comprises 65 epithets, of which 43 are accepted species. Morphologically, *Trichocladium* is characterized by effuse colonies with mycelium partly superficial and partly immersed, absence of stromata, micronematous to semi-, macronematous, mononematous conidiophores that are straight or flexuous and pale brown, and solitary, dry, acrogenous or acropleurogenous, clavate to cylindrical, obovoid or pyriform, smooth to verrucose, thick-walled simple conidia, rounded at the apex, with one-to-many dark brown transverse septa (Park et al. 2017; Tagele et al. 2019). To date, more than six species of *Trichocladium* have been isolated from cave environments (Suppl. material 1: table S2). In the present study, *Trichocladium cavernicola* is described as a new species, representing the seventh cave-associated species known in this genus.

#### Trichocladium cavernicola

Taxon classificationAnimaliaMonhysteridaChaetomiaceae

X.F. Liu, Karun., Tibpromma & K.D. Hyde sp. nov.

A3393D27-2D24-571E-B4A3-221B1BFF1952

Index Fungorum: IF905025

Fig. 23

##### Etymology.

“Caverna” means cave in Latin, and “cola” means dweller or inhabitant, hence the creation of the epithet.

##### Holotype.

GMB-W1542.

##### Description.

Associated with bat guano in the cave environment. Asexual morph on PDA: ***Somatic hyphae*** usually hyaline, thin-walled, 2–4 μm wide, some becoming olivaceous brown and thick-walled when old, with conidia produced on the minimally differentiated conidiophores, on the top of hyphae or the side of vegetative hyphae. ***Conidiophores*** hyaline to subhyaline, unbranched, cylindrical, or slightly swollen on the top, 6–57 μm long, 2–8 μm wide near the top, sometimes lacking where the spores arise from an undifferentiated intercalary conidiogenous cell. ***Conidiogenous cell*** 6–23 × 3–8 μm (x̄ = 14.51 × 5.71 μm, n = 30), pale brown, terminal on conidiophores or intercalary in hyphae. ***Conidia*** 8–23 × 5–16 μm (x̄ = 13.57 × 11.15 μm, n = 120), single-celled, usually solitary, occasionally 2–3 in chains, olivaceous brown to dark olivaceous brown, smooth or slightly verruculose, globose or subglobose, sometimes ellipsoidal, occasionally pyriform, with a conspicuous apical, subapical or lateral germ pore. Sexual morph: Undetermined.

**Figure 23.**
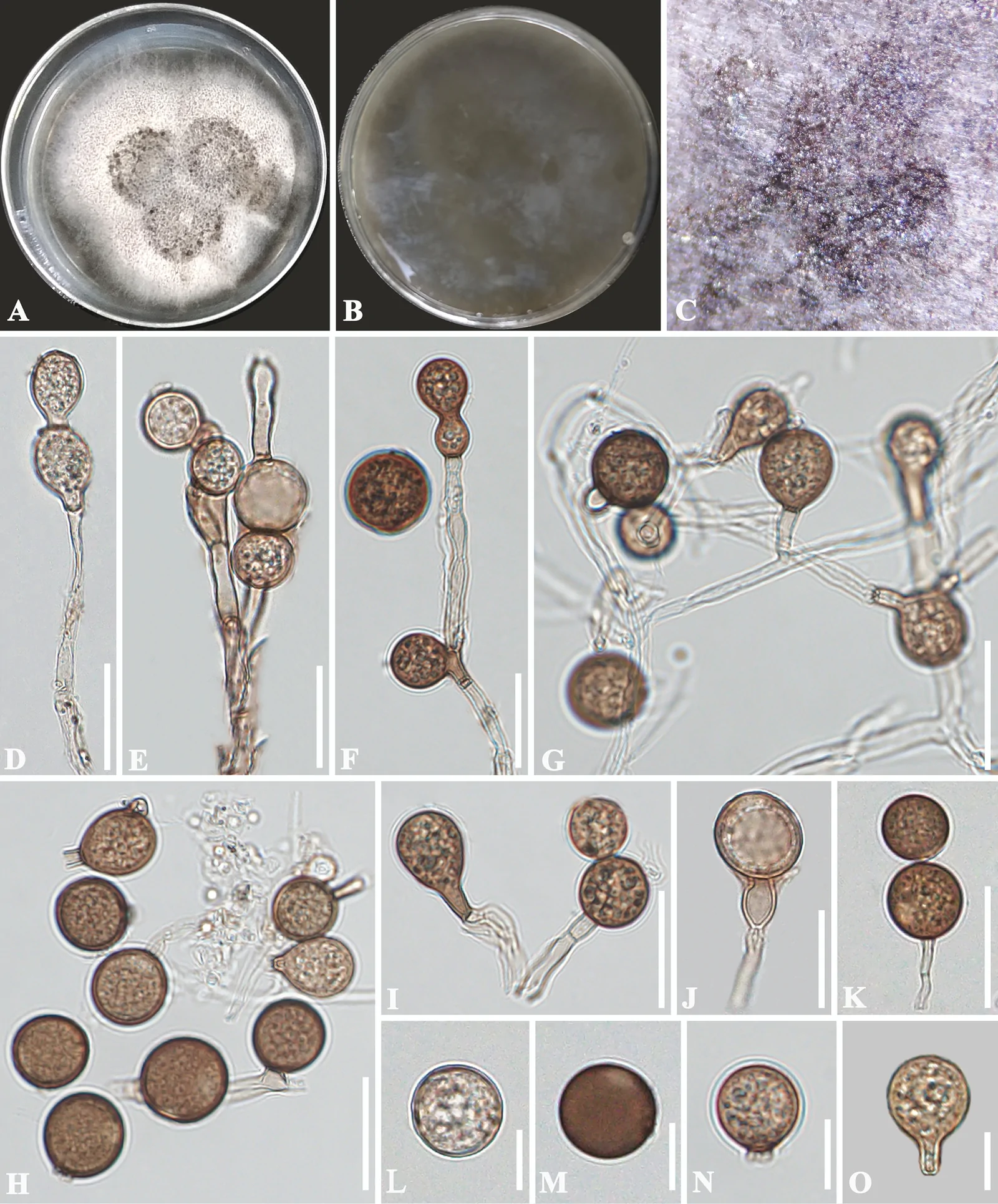
*Trichocladium cavernicola* (GZCC 25-0715, ex-type). **A, B** Colony on PDA (above and below); **C** Mycelium on PDA (50 days old culture); **D–K** Hyphae, conidiophores, and conidia; **L–O** Conidia. Scale bars: 20 μm (**D–K**); 10 μm (**L–O)**.

##### Culture characteristics.

Colony on PDA attaining 60 mm after 15 days at room temperature (25 ± 2 °C), floccose, mouse gray to iron gray; reverse pale olivaceous gray to black. Colonies sporulated on PDA after 40 days. Odor absent.

##### Material examined.

CHINA • Yunnan Province, Kunming City, Fumin County, Chijiu Township, Tianshengqiao Village, Swallow Cave, bat guano, 25.388333°N, 102.537778°E, elev. ca 1864.03 m, 7 May 2021, Xiangfu Liu, FM32E (GMB-W1542, holotype), ex-type GZCC 25-0715; *ibid*., FM32E2 (GMB-W1543, isotype), ex-isotype GZCC 25-0716.

##### Notes.

In phylogenetic analyses based on ITS, *rpb*2, and *tub*2 sequences, *Trichocladium cavernicola* (GZCC 25-0715 (ex-type), and GZCC 25-0716) formed a distinct lineage with 74% ML and 1.00 PP support, clustering as a sister taxon to *Tr. solani*, *Tr. crispatum*, and *Tr. antarcticum* (Fig. 20). Nucleotide comparisons between *Tr. cavernicola* (GZCC 25-0715, ex-type) and *Tr. solani* (VKM F-4902, ex-type) revealed 1.75% (10/572 bp, with 3 gaps) base pair differences in ITS, 2.37% (21/886 bp, with 2 gaps) differences in *rpb*2, and 2.08% (7/336 bp, with 2 gaps) differences in *tub*2. Morphologically, *Tr. cavernicola* is readily distinguished from *Tr. solani* by the latter having acremonium-like conidiophores and conidia formed basipetally in chains or curl up, that later aggregate into heads, and thick-walled, subglobose to angular chlamydospores composed of hyaline cells (Belosokhov et al. 2022). *Trichoderma solani* is a pathogenic fungus that causes potato tuber yellow rot (Belosokhov et al. 2022). In addition, the PHI test results (Fig. 6F) revealed no significant recombination relationships between *Tr. cavernicola* and its phylogenetically related taxa within the dataset. Based on both phylogenetic analyses and morphological characteristics, *Trichocladium cavernicola* is introduced as a new species.

###### *Xylariales* Nannf.

#### Hypoxylaceae

Taxon classificationAnimaliaXylarialesHypoxylaceae

DC.

D3AFEEAA-7EB5-50A0-91F1-9D1CD56B6D4F

##### Notes.

*Hypoxylaceae* was introduced by de Lamarck and de Candolle (1805), but the name was not adopted in modern classifications and was long treated as a synonym of *Xylariaceae* (Hyde et al. 2020). Integrative studies incorporating multi-gene phylogeny, morphology, and chemotaxonomy have since validated *Hypoxylaceae* as a distinct family within *Xylariales* (Wendt et al. 2018; Daranagama et al. 2018). Species of *Hypoxylaceae* are predominantly saprobic or endophytic (Hyde et al. 2020). The sexual morph of *Hypoxylaceae* is characterized by globose to subglobose or elongate, cylindrical to pyriform ascomata embedded in stromata, arranged monostichously; the interior may be zonate or filled with liquid. Asci are unitunicate, cylindrical to clavate, and 4–8-spored. Ascospores are ellipsoidal to subglobose, brown to black, rarely hyaline, and mostly possess a germ slit (Hyde et al. 2020). The asexual morph is characterized by hyaline to light-brown, smooth, branched conidiophores, cylindrical conidiogenous cells, and hyaline, smooth to roughened, ellipsoidal conidia (Daranagama et al. 2018; Wendt et al. 2018). To date, ten species from six genera in this family have been documented from cave environments worldwide (Suppl. material 1: table S2).

#### Daldinia

Taxon classificationAnimaliaXylarialesHypoxylaceae

Ces. & De Not.

68A2B9A4-0326-5470-B75A-50504310A301

##### Notes.

*Daldinia* was introduced by Cesati and De Notaris (1863) and typified by *Daldinia concentrica* Ces. & De Not, named in tribute to the Swiss monk, Agostino Daldini (Cesati and De Notaris 1863; Yin et al. 2024). *Daldinia* is recognized as one of the largest genera in *Hypoxylaceae* (*Xylariales*, *Sordariomycetes*). The latest outline by Hyde et al. (2024) lists 56 accepted species in this genus, while 113 epithets are currently recorded in Index Fungorum (http://www.indexfungorum.org; 14 July 2026). Species of *Daldinia* typically occur on angiosperm hosts and are widely distributed in temperate and tropical regions, with more than 80% of known species occurring in tropical dry forests. In contrast, species found in temperate regions show a more restricted distribution and exhibit greater host specificity (Barbosa-Reséndiz et al. 2020; Liu et al. 2025). This genus is characterized by perithecia immersed in globose to claviform stromata, which are copper, violet, wine-colored, brown, or black, and release pigments that are ochraceous, purple, greenish, or gray when treated with KOH. Asci are cylindrical, stipitate, with an apical amyloid pore. Ascospores are ellipsoid, brown, and smooth to ornamented. The asexual morph is characterized by stout conidiophores and nodulisporium-like or periconiella-like anamorphs (Ju and Rogers 1996; Daranagama et al. 2015, 2018; Liu et al. 2025). In previous studies of cave-associated fungi, only one *Daldinia* species, *D. concentrica*, has been reported from caves in Slovenia and the UK. In this study, *Daldinia eschscholtzii* is reported as a new cave record.

### Phylogenetic analyses: *Daldinia*

Phylogenetic analyses of *Daldinia* were based on a combined dataset of ITS, LSU, *rpb*2, and *tub*2 sequences. The aligned dataset encompassed 60 strains, representing 47 taxa, including *Daldinia* (34 strains, 20 taxa), *Pyrenopolyporus* (five strains, four taxa), *Hypomontagnella* (four strains, one taxon), *Annulohypoxylon* (five strains, five taxa), *Jackrogersella* (three strains, three taxa), *Hypoxylon* (12 strains, 11 taxa), and the outgroup taxa *Graphostroma platystomum* (CBS 270.87) and *Xylaria hypoxylon* (CBS12260). In the resulting phylogenetic tree, the six genera of *Hypoxylaceae* formed well-separated clades, with each genus resolved as an independent lineage. Our new isolate (GMBCC2475) clustered with *Daldinia eschscholtzii* in the *Daldinia* clade.

#### Daldinia eschscholtzii

Taxon classificationAnimaliaXylarialesHypoxylaceae

(Ehrenb.) Rehm, Annls Mycol. 2(2): 175 (1904)

864F18BF-6386-5C47-BD75-CECD37E16390

Index Fungorum: IF 544992

Fig. 25

##### Description.

Associated with rock surface in the cave environment. Asexual morph: ***Vegetative hyphae*** septate, branched, hyaline to brown, smooth-walled, 0.8–6.1 μm wide. ***Conidiophores*** sparse on aerial mycelium, straight or flexuous, erect or prostrate, smooth- and thin-walled, branched to unbranched, or reduced to conidiogenous cells borne laterally on hyphae. ***Conidiogenous cells*** mono- to polyblastic, blastic-sympodial, cylindrical, smooth- and thin-walled, 8.6–54.7 × 1.2–6.1 μm (x̄ = 27.53 × 3.22 μm, n = 30), with minute denticles after secession of conidia. ***Conidia*** 3.2–12.5 × 2.3–5.3 μm (x̄ = 6.82 × 3.30 μm, n = 60), cylindrical, reniform to lunate, or contracted at one end, hyaline to brown, aseptate, granulate, walls smooth and thin. Sexual morph: Undetermined.

##### Culture characteristics.

Colonies on PDA attaining 60 mm after 7 days at room temperature (25 ± 2 °C), with undulate edge, white to pale yellow, sparse aerial mycelium on the surface; reverse pale yellow. The white reproductive mycelium covers the stromatic colonies after 50 days.

##### Material examined.

CHINA • Yunnan Province, Nujiang Lisu Autonomous Prefecture, Lushui City, Daxingdi Town, rock surface in Walaya Cave, 26.080743°N, 98.848391°E, elev. ca 850.46 m, 23 April 2024, Xiangfu Liu and Xuemei Chen, NJ9A (GMB-W1474), living culture, GMBCC2475.

##### Known distribution.

Australia, Bahamas, Bangladesh, Brazil, Brunei Darussalam, Cook Islands, Cuba, Democratic Republic of the Congo, Federal States of Micronesia, Ghana, Japan, Kenya, Malaysia, New Zealand, Nigeria, Pakistan, Papua New Guinea, Philippines, Saint Lucia, Seychelles, Sierra Leone, Singapore, Tanzania (Farr and Rossman 2026), China (Farr and Rossman 2026; this study), France, Germany, Mexico, South Africa (Jayasiri et al. 2015), India, Indonesia, Samoa, Trinidad and Tobago, the USA (Jayasiri et al. 2015; Farr and Rossman 2026), and Thailand (Jayasiri et al. 2015; Brooks et al. 2024; Farr and Rossman 2026).

##### Known hosts or substrata.

*Cordia sebestena*, *Acacia* sp., *Albizia lebbeck*, *Albizia* sp., *Albizia zygia*, *Angelica* sp., *Annona muricata*, *Antidesma* sp., *Artocarpus altilis*, *Artocarpus integrifolius*, *Brguiera sexangula*, *Bucida palustris*, *Bursera simaruba*, *Cassia floribunda*, *Casuarina* sp., *Celtis kraussiana*, *Citrus aurantiifolia*, *Citrus aurantium*, *Citrus paradisi*, *Citrus* sp., *Citrus limon*, *Citrus nobilis*, *Cocos nucifera*, *Cordia sebastina*, *Croton* sp., *Dendrobium officinale*, *Entandrophragma cylindricum*, *Eucalyptus alba*, *Eucalyptus* sp., *Excoecaria agallocha*, *Ficus* sp., *Gliricidia sepium*, *Heteromorpha trifoliata*, *Hevea brasiliensis*, *Hevea* sp., *Hippomane mancinella*, *Jambosa vulgaris*, *Mangifera indica*, *Morus alba*, *Musa* sp., *Neohypodiscus cerebrinus*, *Nothofagus* sp., *Pinus* sp., *Platanus wrightii*, *Pogostemon cablin*, *Quercus acutissima*, *Quercus* sp., *Sizygium jambos*, *Tamarindus indica*, wood, and *Xestospongia* sponge species (Farr and Rossman 2026), *Oncosperma* sp. (Brooks et al. 2024), and rock surface in cave habitat (this study).

##### Notes.

**Figure 24.**
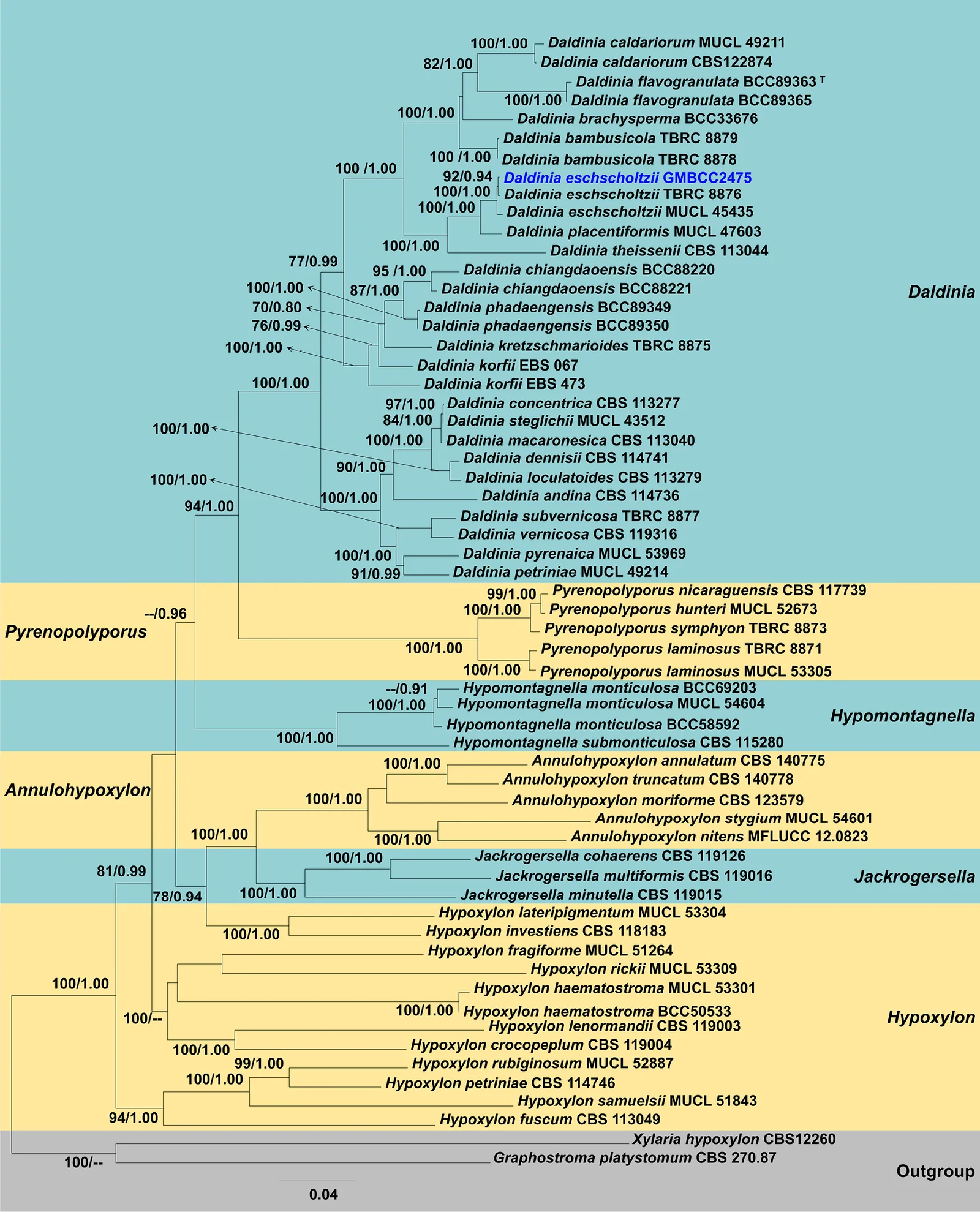
Consensus phylogram of 1,000 trees resulting from a RAxML analysis of the (ITS, LSU, *rpb*2, and *tub*2) alignment of the analyzed *Daldinia* sequences. The RAxML analysis of the combined dataset yielded the best-scoring tree, with a final ML optimization likelihood value of -25842.101346. This tree was generated from 579, 776, 1029, and 840 base pairs of ITS, LSU, *rpb*2, and *tub*2 characters, respectively. The matrix comprises 1,106 distinct alignment patterns with 9.64% of gaps and indeterminate traits. Base frequencies were estimated as follows A = 0.224919, C = 0.285712, G = 0.281671, T = 0.207698 with substitution rates AC = 1.029107, AG = 3.610183, AT = 1.189562, CG = 1.444536, CT = 5.566958, GT = 1.000000; proportion of invariable sites I = 0.521952; gamma distribution shape parameter α = 0.924023. The trees from both analyses (ML and BI) showed identical topologies. RAxML bootstrap support values (ML equal to or above 70%) and Bayesian inference (BYPP equal to or above 0.90) are given at the nodes (ML/BYPP). The sequences of new strains are in red. The scale bar represents the expected number of changes per site.

*Daldinia eschscholtzii* was first reported by Rehm (1904) based on its sexual morphological characters, including elliptic to falcate ascospores, measuring 10–12 × 5 μm, from specimens collected in the USA. Our new isolate (GMBCC2475) shows morphological similarities to *D. eschscholtzii* (MFLUCC 20–0232), sharing obovoid to ellipsoid, aseptate, smooth conidia, often with a truncate base, and conidiophores forming a dichotomous to trichotomous conidiogenous apparatus with a Nodulisporium-like branching pattern, in which 1–3 conidiogenous cells arise from each terminal branch (Samarakoon et al. 2019; Gajanayake et al. 2021). Multi-gene phylogeny of the combined ITS, LSU, *tub*2 and *rpb*2 sequences further showed that our isolate clusters with *D. eschscholtzii* and is clearly separated from other species with 100% ML and 1.00 BYPP support (Fig. 24). Therefore, based on both morphology and phylogeny evidence, we report this isolate as a new substrate record of *D. eschscholtzii* from a cave in Yunnan, China.

**Figure 25.**
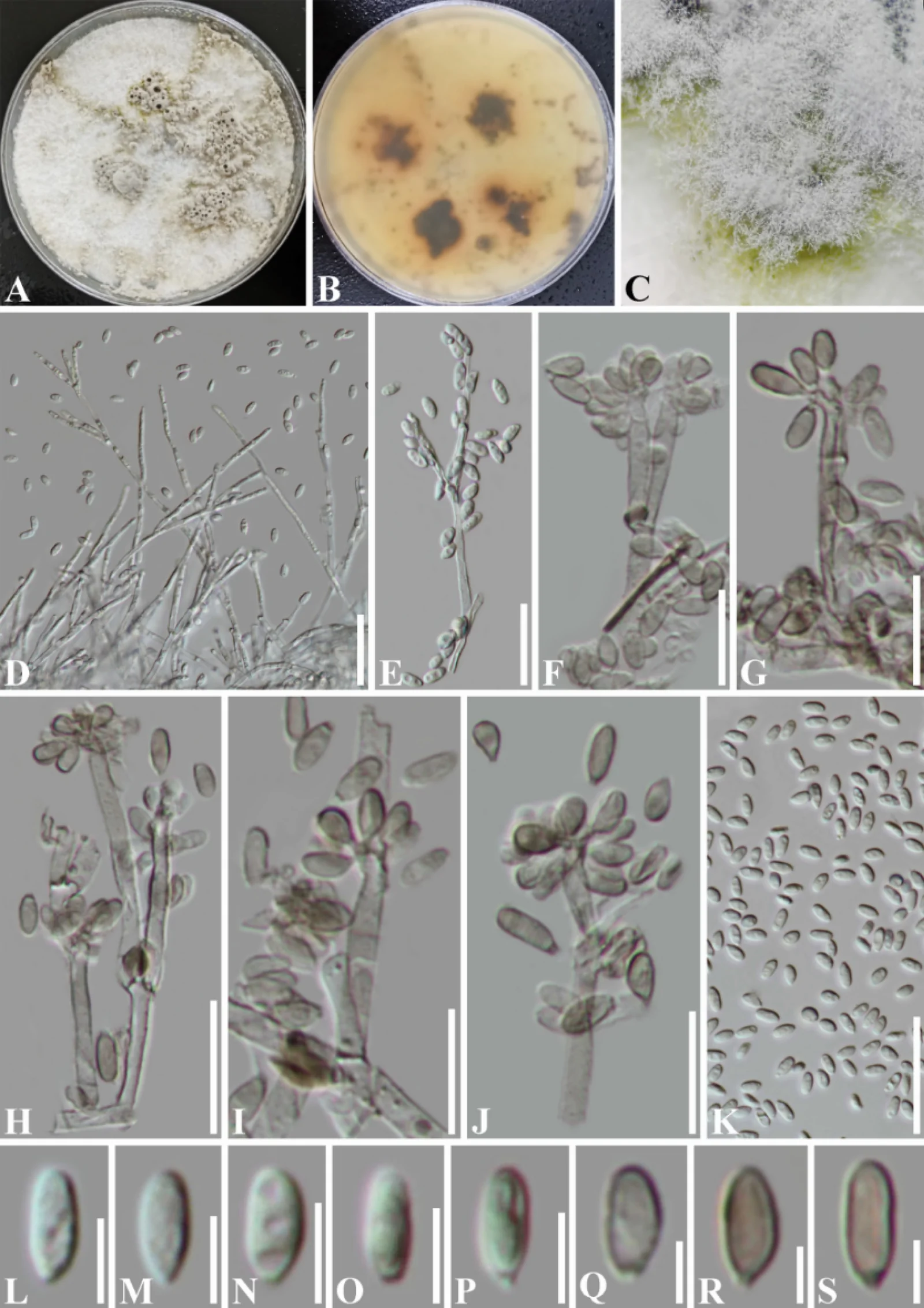
*Daldinia eschscholtzii* (GMBCC2475, new substrate record in cave habitat) **A, B** Colony characteristics on PDA (above and below); **C** Mycelium on PDA (after 50 days old culture); **D–J** Conidiogenous cells and conidia; **K–S** Conidia. Scale bars: 30 μm (**D, H, K)**; 20 μm (**E–G, I, J**); 5 μm (**L–S**).

#### *Mucoromycota* Doweld

***Mucoromycetes* Doweld**

***Mucorales* Dumort. [as ‘Mucorarieae’], Analyse des familles des plantes: avec l’indication des principaux genres qui s’y rattachent: 73 (1829)**

##### Mucoraceae

Taxon classificationAnimaliaXylarialesMucoraceae

Fr. [as ‘Mucoroidei’], Systema Mycologicum 1: xlix (1821)

3E5173E0-3A79-5A0A-94B7-C3366B4939B9

###### Notes.

*Mucoraceae* was introduced by Fries (1821) within *Mucorales* and subsequently revised by numerous mycologists, becoming a classical representative of the former class *Zygomycetes* G. Winter within the phylum *Zygomycota* Moreau (Dumortier 1822). Members of *Mucoraceae* are morphologically characterized by the production of aseptate hyphae, sporangiospores, and zygospores formed through the fusion of gametangia (Sutton 2014; Voglmayr and Clémençon 2016). With the widespread application of molecular markers, traditional generic concepts within the family have been substantially re-evaluated (Voglmayr and Clémençon 2016). Several genera previously placed in *Mucoraceae*, such as *Rhizopus* and *Lichtheimia*, were shown to be only distantly related to the core *Mucor* lineage and are now classified in separate families, *Rhizopodaceae* and *Lichtheimiaceae*, respectively (Walther et al. 2013; Hoffmann et al. 2013). Currently, *Mucoraceae* comprises approximately 26 genera (Benny et al. 2014; Hyde et al. 2024). Members of this family are widely distributed and occur in diverse ecological niches, particularly in soils, decaying organic matter, animal dung, and spoiled food. Some species are thermotolerant or psychrotolerant, enabling adaptation to extreme environmental conditions (Richardson and Lass-Flörl 2008). To date, more than 33 species belonging to four genera (*Actinomucor* Schostak., *Helicostylum* Corda, and *Thamnidium* Link) have been isolated from cave environments worldwide (Suppl. material 1: table S2).

##### Mucor

Taxon classificationAnimaliaXylarialesMucoraceae

Fresen., Beitr. Mykol. 1: 7 (1850)

CAE2AF42-6798-516B-A042-B6F6C446E3E4

###### Notes.

*Mucor* was established by Fresenius (1850) with *M. mucedo* Fresen. as the type species and is a cosmopolitan genus within *Mucorales*, the phylum *Mucoromycota*. It is among the most frequently encountered fungal genera in both natural and human-associated environments and is the second most prominent genus after *Penicillium* (Hermet et al. 2012). Currently, 781 epithets are listed under *Mucor* in Index Fungorum (http://www.indexfungorum.org; accessed date 14 July 2026). Members of *Mucor* are characterized by fast-growing colonies with abundant aerial and substrate hyphae, and by the formation of isomorphic, globose to subglobose, non-apophysate sporangia with incrusted and deliquescent walls, usually borne on unbranched sporangiophores, and no apophyses. Zygospores are formed with opposed or tong-like suspensors (Schipper 1978; Benny et al. 2016). Most species are saprobes and are commonly isolated from air, dust, soil, stored grains, decaying fruits, rotting vegetables, *Agaricus* mushrooms, and herbivore dung (Alves et al. 2002; Schoenlein-Crusius et al. 2006; Jacobs and Botha 2008; Santiago et al. 2011; Lima et al. 2015). In addition to their ecological roles, *Mucor* species exhibit considerable biotechnological potential and are widely applied in enzyme production (Alves et al. 2002; Lee et al. 2011; Huang et al. 2014), food fermentation (Morin-Sardin et al. 2017), biofuel development (Carvalho et al. 2018), as well as in biotransformation and bioremediation processes (Jabasingh and Pavithra 2010; Oliveira Silva et al. 2013; Silva et al. 2015). However, some species are opportunistic pathogens that can cause cutaneous and systemic mucormycosis in humans, as well as diseases in plants and cultivated mushrooms (Hoffmann et al. 2013; Walther et al. 2013; Chibucos et al. 2016; Panthee et al. 2021; Gajanayake et al. 2023; Zhao et al. 2023a). To date, 29 *Mucor* species have been reported from cave environments (Suppl. material 1: table S2), including one cave-adapted species, *Mucor cavernicola* X.F. Liu, Tibpromma & K.D. Hyde, described from Yunnan Province, China (Liu 2025). In this study, *Mucor phayaoensis* is reported as a new substrate and geographical record.

##### Mucor phayaoensis

Taxon classificationAnimaliaXylarialesMucoraceae

V.G. Hurdeal, E. Gentekaki & K.D. Hyde, in Hyde et al. Mycosphere 12(1): 1144 (2021)

9D64FAC1-6EE9-5853-A0BD-AD17D1DC945D

Index Fungorum: IF558522

Fig. 27

###### Description.

Associated with water in the cave environment. Asexual morph on PDA: ***Hyphae*** unbranched or simply branched, robust, hyaline, substrate hyphae abundant, root-like, branched. ***Rhizoids*** abundant, root-like, branched, swollen, and consistently produce chlamydospores. ***Sporangiophores*** 3–27 μm (x̄ = 8.34 μm, n = 60) in width, arising directly from substrate hyphae, erect to curved, having long or short, simply branched, the younger parts of the sporangiophores filled with granules, septate. ***Sporangia*** 11–30 × 10–26 μm (x̄ = 21.98 × 19.82 μm, n = 10), mostly globose, occasionally subglobose, multi-spored, brown to black, deliquescent-walled. ***Columellae*** 6–31 × 4–23 μm (x̄ = 13.62 × 10.85 μm, n = 30), mostly globose, sometimes broad ovate, hyaline to pale brown, and leaving small collars. ***Collar*** usually absent, hyaline to brown. ***Sporangiospores*** 4–11 × 2–9 μm (x̄ = 5.55 × 4.14 μm, n = 90), ellipsoidal to globose, sometimes flattened on one side, smooth, thin-walled, hyaline. ***Chlamydospores*** smooth-walled, hyaline, intercalary and terminal, variable in shape and size. ***Zygospores*** not observed.

###### Culture characteristics.

Colonies on PDA at room temperature (25 ± 2 °C) for 7 days, reaching 60 mm in diameter, granulate, broadly lobed and zonate, initially white, gradually becoming light yellow; reverse white to yellowish gray. Sporulation dense on PDA after 14 days.

###### Material examined.

CHINA • Yunnan Province, Mang City, Mengga town, Sanxian Cave, water, 24.232220°N, 98.426103°E, elev. ca 1530.89 m, 23 April 2024, Xiangfu Liu and Xuemei Chen, MS10E (GMB-W1241), living culture GMBCC2472; *ibid*., MS10A (GMB-W1240), living culture GMBCC2471.

###### Known distribution.

China (this study) and Thailand (Hyde et al. 2021).

###### Known hosts or substrata.

Soil (Hyde et al. 2021), water (this study).

###### Notes.

**Figure 26.**
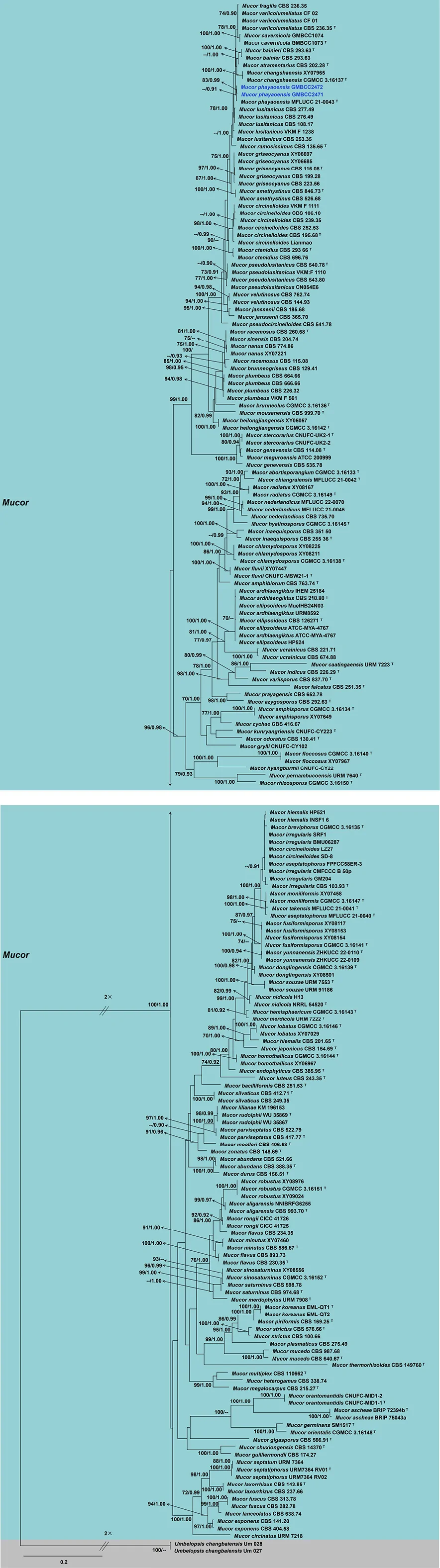
Consensus phylogram of 1,000 trees resulting from a RAxML analysis of the (ITS and LSU) alignment of the analyzed *Mucor* sequences. The RAxML analysis of the combined dataset yielded the best-scoring tree, with a final ML optimization likelihood of -22170.939495, based on 505 and 1,017 base pairs of ITS and LSU characters, respectively. The matrix comprises 962 distinct alignment patterns with 34.76% of gaps and indeterminate traits. Base frequencies were estimated as follows A = 0.302975, C = 0.170643, G = 0.228297, T = 0.298085 with substitution rates AC = 0.955315, AG = 4.107112, AT = 1.801966, CG = 0.674002, CT = 6.356781, GT = 1.000000; proportion of invariable sites I = 0.404084; gamma distribution shape parameter α = 0.569486. The tree topologies from both analyses (ML and BI) were identical. RAxML bootstrap support values (ML equal to or above 70%) and Bayesian inference (BYPP equal to or above 0.90) are given at the nodes (ML/BYPP). The sequences of new strains are in blue. The scale bar represents the expected number of changes per site.

*Mucor phayaoensis* was introduced by Hyde et al. (2021). Based on phylogenetic analyses of the combined LSU and ITS sequence data, our isolates (GMBCC2472 and GMBCC2471) clustered with the ex-type isolate (MFLUCC 21-0043) of *M. phayaoensis* with 83% ML and 0.99 BYPP support (Fig. 26). Nucleotide comparison between our strains (GMBCC2472 and GMBCC2471) and *M. phayaoensis* (MFLUCC 21-0043, ex-type) revealed 1.34% (8/598 bp, with 2 gaps) base pair differences in ITS, and 0.32% (3/948 bp, without gaps) differences in LSU. Morphologically, the new isolates (MS10E) are similar to *M. phayaoensis*, having globose to broad-ovate columellae with a short collarette and ellipsoidal to globose sporangiospores, sometimes flattened on one side (Hyde et al. 2021). Based on phylogenetic and morphological evidence, our strains were identified as *Mucor phayaoensis*, representing the first record of this species from a cave environment in China.

**Figure 27.**
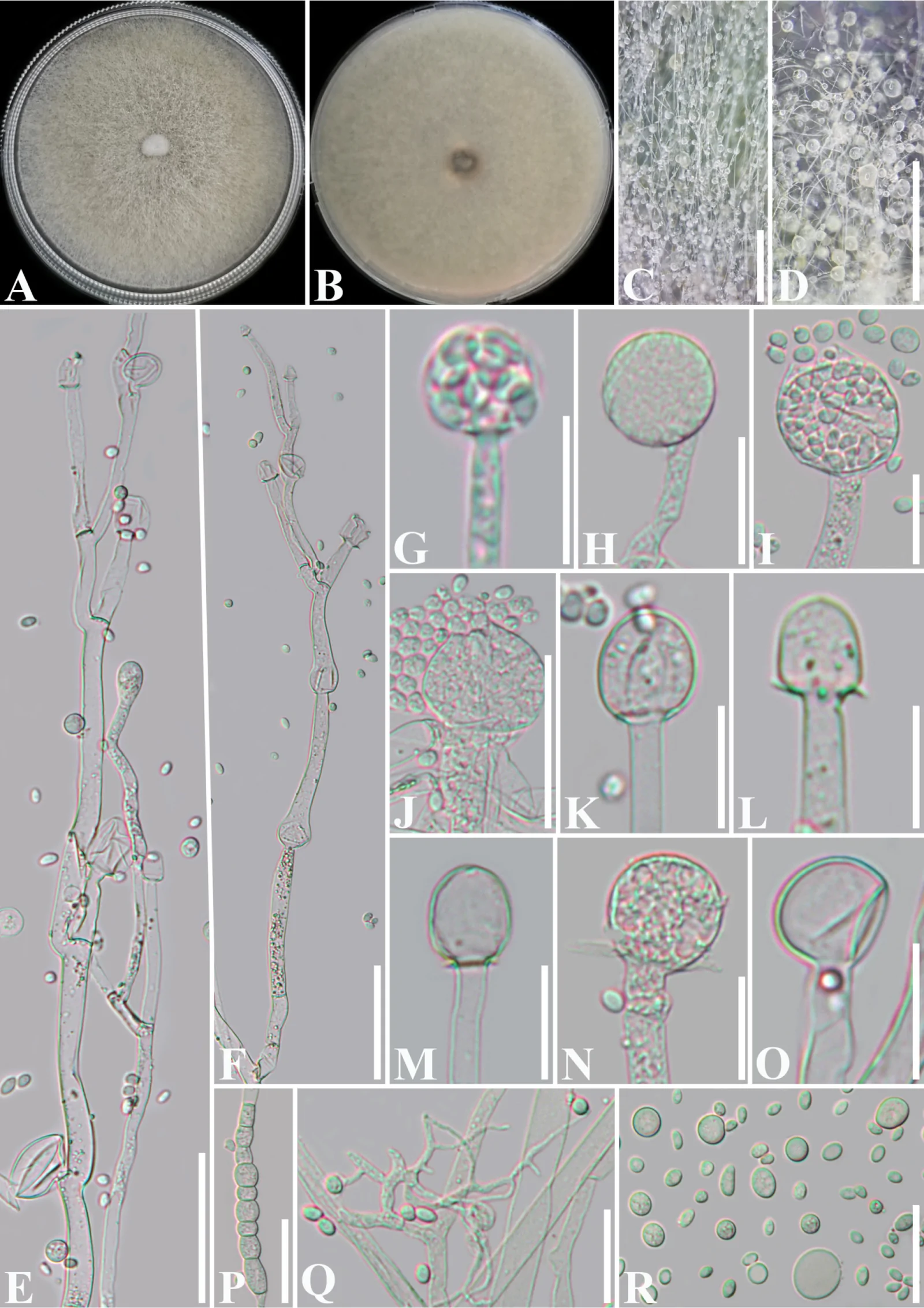
*Mucor phayaoensis* (GMBCC2471, new substrate and geographical record). **A, B** Colony on the PDA (above and below); **C, D** Sporulation on PDA; **E, F** Sporangiophores; **G–J** Sporangia; **J, K** Columellae with collars; **P** Chlamydospores; **Q** Rhizoids. **L** Sporangiospores; Scale bars: 500 μm (**C, D**); 50 μm (**E, F**); 20 μm (**G–O, Q**): 30 μm (**P, R**).

## Discussion

Karst caves represent a unique ecological niche characterized by darkness, high humidity, stable temperatures, and oligotrophic conditions (Zhang et al. 2017, 2021). These distinctive yet stable environments, marked by low interspecific competition and limited nutrient availability, favor the persistence of highly specialized or cryptic species (Savković et al. 2025). Recent research has revealed that caves harbor unexpectedly diverse fungal communities, including numerous taxa that are novel or rarely reported from other habitats (Zhang et al. 2017, 2021; Liu et al. 2021). For instance, Zhang et al. (2021) isolated over 2,300 fungal strains from 13 caves in southwest China, identifying 610 species across 253 genera, with more than 50% being newly recorded for caves and 33 described as novel species. These findings are consistent with global patterns reported by Vanderwolf et al. (2013a), who compiled 1,029 fungal species from cave environments worldwide, primarily belonging to *Ascomycota*. Subsequent studies have expanded this inventory to nearly 2,000 species (Chen et al. 2017; Jiang et al. 2017; Zhang et al. 2017, 2021; Cunha et al. 2020; Liu et al. 2021, 2025, 2026; Liu 2025).

**Figure 28.**
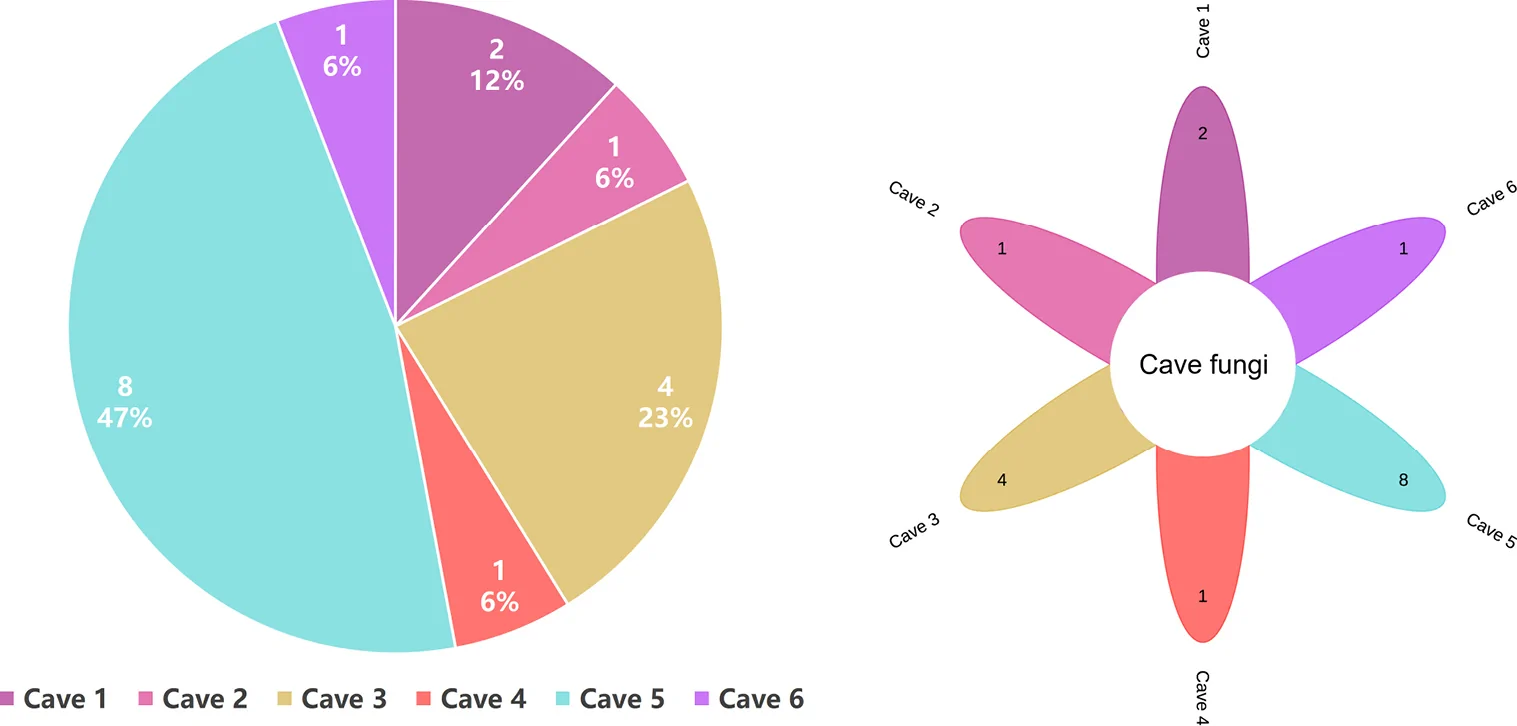
Species distribution across the six caves. Cave names are abbreviated, and full names are in Table 1.

**Figure 29.**
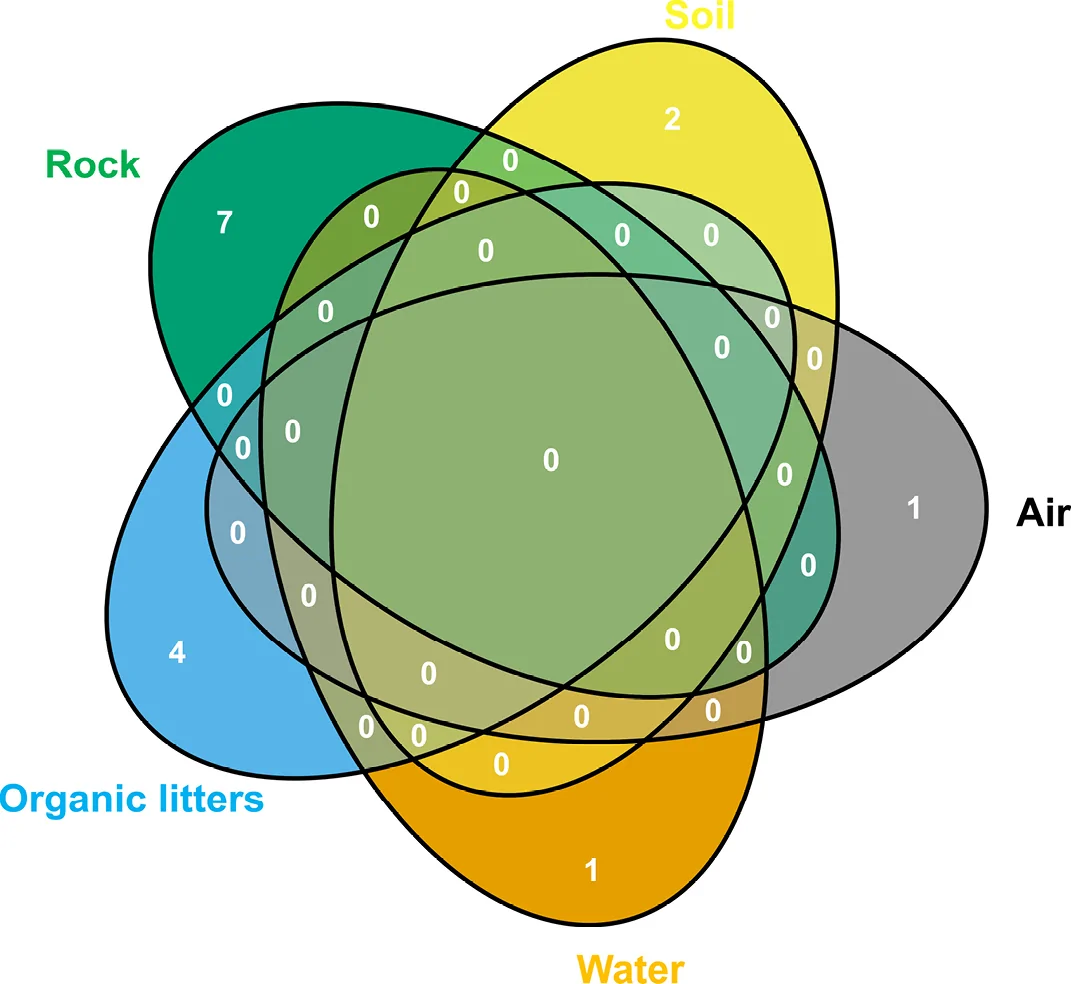
Species distribution across the substrates in the study. Organic litters include: bat guano, candle, bamboo stick, feather, and petroleum wax.

A total of 34 fungal strains were documented from six caves in Yunnan Province, representing 17 species (Fig. 28). These include one new genus, 12 new species, and five new substrate or geographic records. Of these, eight species are classified within *Trichoderma* (seven new species and one new record), two within *Aspergillus* (one new species and one new record), and the remaining species are assigned to *Chaetomium*, *Cosmospora*, *Daldinia*, *Mucor*, *Neoachaetomiella*, *Pithoascus*, and *Trichocladium*. Regarding substrate isolation, four strains from soil samples represent two species in one genus; 13 strains from organic litter correspond to six species in four genera; 13 strains from rock samples represent seven species in six genera; two strains from water samples belong to one species; and two strains from air samples belong to one species (Fig. 29).

**Figure 30.**
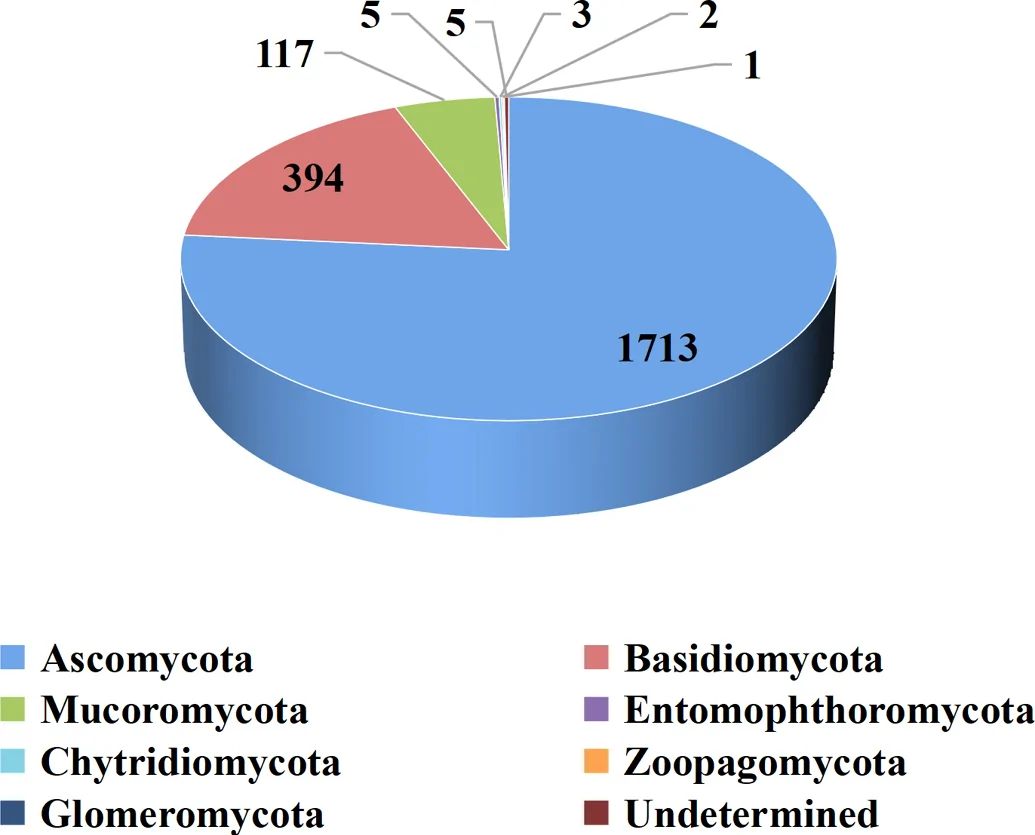
The number of fungal species in different phyla obtained worldwide.

**Figure 31.**
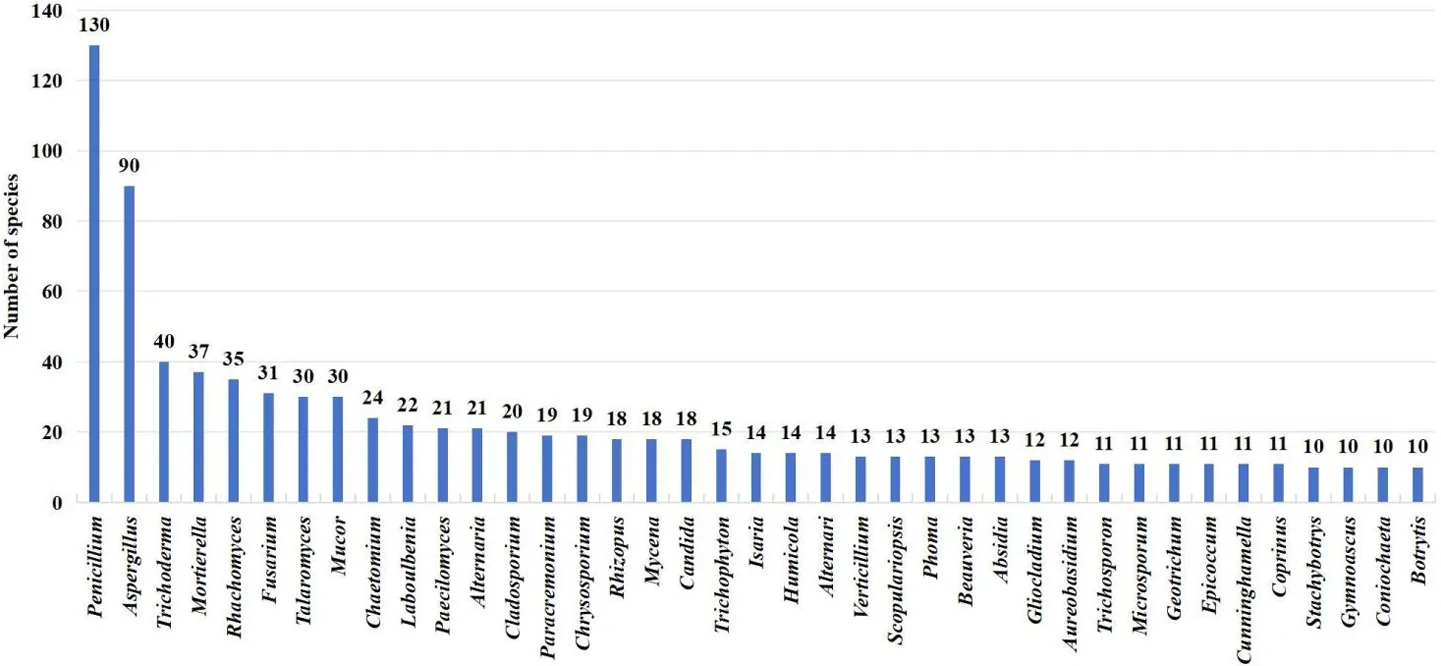
The most abundant cave-associated fungal genera reported worldwide (≥ 10 species).

Incorporating these findings, the total number of fungi and fungus-like organisms recorded from caves worldwide reaches 2,240, representing 66 countries or regions (Suppl. material 1: table S2). Of these taxa, 76.5% (1,713 species) were *Ascomycota*, 17.6% (394 species) *Basidiomycota*, 5.2% (117 species) *Mucoromycota*, 0.5% (11 species) belong to other phyla (*Entomophthoromycota*, *Chytridiomycota*, *Zoopagomycota*, *Glomeromycota*, and undetermined species), and five undetermined (Fig. 30). According to our count, there are 647 fungal genera from cave environments, with the most frequently reported genera being *Penicillium* (130), *Aspergillus* (90), *Trichoderma* (40), *Mortierella* (37), *Rhachomyces* (35), *Fusarium* (31), *Talaromyces* (30), *Mucor* (30), *Chaetomium* (24), *Laboulbenia* (22), *Paecilomyces* (21), *Alternaria* (21), and *Cladosporium* (20) (Suppl. material 1: table S2, Fig. 31). Consistent with global trends, *Trichoderma* was the most frequently isolated genus in our study, and the newly described cave-derived genus, *Neoachaetomiella*, for the first time.

Numerous fungal species isolated from caves possess significant ecological and applied importance, encompassing taxa with pathogenic potential as well as those relevant to biological control, industry, and medicine. *Pseudogymnoascus destructans*, the etiological agent of white-nose syndrome in bats, has caused severe declines in north American bat populations, underscoring its importance as a surveillance target in cave ecosystem conservation (Vanderwolf et al. 2013a; Liu et al. 2021; Zhang et al. 2021; Savković et al. 2025). Although this fungal pathogen has been detected in northeast China, its prevalence and infection load in hosts and the environment are relatively low compared with those observed in North American bats, suggesting that Chinese bats exhibit strong resistance to *Pseudogymnoascus destructans* (Hoyt et al. 2016; Sun et al. 2025). *Histoplasma capsulatum*, a prominent cave-associated pathogen responsible for histoplasmosis, constitutes a major concern in public health monitoring (Vanderwolf et al. 2013a; Barbosa et al. 2025). Additionally, *Purpureocillium lavendulum* and *Metapochonia variabilis* are recently identified nematode-trapping fungi with distinctive physiological characteristics. Due to their considerable potential for biological control and environmental adaptability, these species have garnered increasing attention in agricultural microbiology research and applications (Zhang et al. 2017; Liang et al. 2021; Bao et al. 2022). A diverse array of significant species has also been recovered from commonly encountered genera such as *Penicillium*, *Aspergillus*, and *Trichoderma*. For instance, *Aspergillus cavernicola*, *A. fumigatus*, *Penicillium expansum*, and *P. panissanguineum* have demonstrated strong antimicrobial or antitumor activities, emphasizing their value in pharmaceutical research (Zhou et al. 2018; Rystsov et al. 2021; Silva et al. 2021; Barbosa et al. 2025; Zakaria 2026). *Trichoderma harzianum* and *T. polysporum* are among the most widely utilized and effective biological control agents worldwide (Harman et al. 2004; Zhang et al. 2015).

The earliest documented study of cave fungi was by Humboldt in 1794, as described by Dobat (1967) and Megušar (1914), who published the first formal studies on cave ecology. Since then, publications on cave fungi have gradually increased and entered an explosive phase after 2006, primarily driven by the emergence of white-nose syndrome in North American bats (Vanderwolf et al. 2013a; Zhang et al. 2021). Research efforts on cave fungi are primarily concentrated in Europe and North America, followed by Asia, South America, Africa, and Oceania (Vanderwolf et al. 2013a). Among all countries, the United States (37 papers), France (25 papers), Italy (23 papers), Spain (19 papers), and the United Kingdom (17 papers) have contributed most to cave mycology research (Vanderwolf et al. 2013a). In the 21^st^ century, research on cave fungi in Asia has accelerated, with numerous papers being published, particularly in China, highlighting the high fungal diversity in East Asian karst systems.

China has become one of the countries with the most systematic research on cave fungi and has discovered several new species in recent years. Including the 17 species obtained in our study, 493 cave fungal species have now been reported in China, mainly distributed in the southwestern region. Yunnan (33 species), Sichuan (300 species), and Guizhou (160 species) (Zhang et al. 2017, 2021; Liu et al. 2021, 2025, 2026). These areas correspond to the core regions of East Asian karst landform development, which feature the world’s largest and most complex karst landforms and distinctively rich cave resources (Zhou et al. 2007; Zhang et al. 2021). Despite these advances, most caves remain unexplored, suggesting that the true fungal diversity of these habitats remains underestimated.

Although numerous cave-associated fungal species have been reported, their diversity remains limited relative to the vast number of unexplored caves and the potential fungal communities they harbor (Zhang et al. 2017, 2021; Liu et al. 2021, 2025). Current research on cave fungi primarily relies on traditional culture-based methods for isolation and identification. However, these techniques often fail to recover slow-growing or fastidious taxa. Future studies should incorporate molecular approaches, such as metagenomics and next-generation sequencing (NGS), to expand the range of detectable microorganisms, including unculturable taxa, and to provide a more comprehensive understanding of the diversity within cave-associated fungal communities.

Clarifying the taxonomic and ecological diversity of cave-associated fungi is essential for advancing our understanding of their ecological functions. However, several challenges persist in cave mycology, including unresolved questions about their origins, evolutionary history, community structure, and functional roles. Although over 2,200 fungal species, including many novel and taxa currently known only from cave collections, previous studies have seldom provided experimental evidence for their adaptive traits. Specifically, research on cave fungi has generally lacked physiological investigations, such as assessments of reduced pigmentation, altered light sensitivity, or distinct metabolic responses under oligotrophic conditions, which could clarify whether these taxa are genuinely adapted to cave environments or are merely incidental isolates. Notably, cave fungi are a promising source of bioactive compounds with potential applications in antibacterial, antioxidant, antitumor, and enzymatic activities, as well as in organic acid production (Barbosa et al. 2025). To facilitate both scientific inquiry and industrial utilization, the accurate identification and effective preservation of fungal strains are fundamental (Barbosa et al. 2025). Systematic exploration of cave fungi will not only expand the resource base for applied research but also contribute significantly to the conservation and sustainable utilization of subterranean microbial diversity.

With the rapid expansion of tourism and increasing human activities, cave ecosystems face increasing pressure, which threatens fragile habitats and their endemic microbial communities. Many newly discovered fungal species are confined to small, localized habitats that are highly susceptible to disturbance or destruction. Therefore, it is imperative to strengthen both the exploration and conservation of cave fungi. Establishing a comprehensive database of cave microbial resources, implementing long-term ecological monitoring in representative caves, and adopting a minimal ecological disturbance strategy during sampling, especially in undeveloped caves, are essential measures. Additionally, raising public awareness of the ecological and biotechnological importance of subterranean microbial resources is crucial for their sustainable protection and utilization.

## Supplementary Material

XML Treatment for Aspergillaceae

XML Treatment for Aspergillus

XML Treatment for Aspergillus baeticus

XML Treatment for Aspergillus dehongensis

XML Treatment for Hypocreaceae

XML Treatment for Trichoderma

XML Treatment for Trichoderma dehongense

XML Treatment for Trichoderma hongheense

XML Treatment for Trichoderma mangshiense

XML Treatment for Trichoderma neogongcheniae

XML Treatment for Trichoderma neopyramidale

XML Treatment for Trichoderma nujiangense

XML Treatment for Trichoderma obovatum

XML Treatment for Trichoderma yingjiangense

XML Treatment for Nectriaceae

XML Treatment for Cosmospora

XML Treatment for Cosmospora cavernicola

XML Treatment for Microascaceae

XML Treatment for Pithoascus

XML Treatment for Pithoascus cavernicola

XML Treatment for Chaetomiaceae

XML Treatment for Neoachaetomiella

XML Treatment for Neoachaetomiella cavernicola

XML Treatment for Chaetomium

XML Treatment for Chaetomium subaffine

XML Treatment for Trichocladium

XML Treatment for Trichocladium cavernicola

XML Treatment for Hypoxylaceae

XML Treatment for Daldinia

XML Treatment for Daldinia eschscholtzii

XML Treatment for Mucoraceae

XML Treatment for Mucor

XML Treatment for Mucor phayaoensis

## Additional information

### Artificial Intelligence (AI) use

The authors accept full responsibility for the content of the manuscript, including the disclosure of any use of AI.

No AI tools were used in the preparation of this manuscript.

### Adherence to national and international regulations

All the fungal strains used in this study have been legally obtained, respecting the Convention on Biological Diversity (Rio Convention).

### Author ORCIDs

X.-F. Liur https://orcid.org/0000-0003-0100-2094

S. Tibpromma https://orcid.org/0000-0002-4706-6547

K.W.T. Chethana https://orcid.org/0000-0002-5816-9269

L. Lu https://orcid.org/0000-0003-0977-6414

K. D. Hyde https://orcid.org/0000-0002-2191-0762

W.-H. Lu https://orcid.org/0000-0001-7283-7596

R.-F. Xu https://orcid.org/0000-0003-1207-8254

X.-M. Chen https://orcid.org/0009-0004-8631-0735

X. Zhang https://orcid.org/0000-0001-6097-8922

M.-X. Chen https://orcid.org/0009-0004-0732-5278

X.-J. Xiao https://orcid.org/0009-0003-8769-4534

C.-L. Zhao https://orcid.org/0000-0002-8668-1075

T.-Y. Du https://orcid.org/0000-0003-2105-1803

D.-Q. Dai https://orcid.org/0000-0001-8935-8807

A. M. Elgorban https://orcid.org/0000-0003-3664-7853

N. Suwannarach https://orcid.org/0000-0002-2653-1913

J. Kumla https://orcid.org/0000-0002-3673-6541

F.-Q. Yu https://orcid.org/0000-0001-7931-080X

M.-M. Wang https://orcid.org/0000-0003-3747-5432

S. C. Karunarathna https://orcid.org/0000-0001-7080-0781

### Data availability

All of the data that support the findings of this study are available in the main text or Supplementary Information.

## Supplementary materials

Supplementary material 1 Supplementary information Data type doc Explanation note **figure S1**. Phylogenetic trees of *Aspergillus* section *Usti* based on ITS sequences. Bootstrap support values for maximum likelihood (ML) equal to or higher than 70% and Bayesian probability (BYPP) equal to or higher than 0.90 are mentioned above the branches (ML/BI). Newly generated sequences in this study are in blue and red. Type strains are denoted with ^T^. **figure S2**. Phylogenetic trees of *Aspergillus* section *Usti* based on *BenA* sequences. Newly generated sequences in this study are in blue and red. Bootstrap support values for maximum likelihood (ML) equal to or higher than 70% and Bayesian probability (BYPP) equal to or higher than 0.90 are mentioned above the branches (ML/BI). Newly generated sequences in this study are in blue and red. Type strains are denoted with ^T^. **figure S3**. Phylogenetic trees of *Aspergillus* section *Usti* based on *rpb*2 sequences. Newly generated sequences in this study are in blue and red. Bootstrap support values for maximum likelihood (ML) equal to or higher than 70% and Bayesian probability (BYPP) equal to or higher than 0.90 are mentioned above the branches (ML/BI). Newly generated sequences in this study are in blue and red. Type strains are denoted with ^T^. **Table 1**. Details of the fungal strains included in the phylogenetic analyses; - refers to the missing data. **Table 2**. List of fungal and fungus-like organisms reported in cave environments. NF-Number of locations taxa found. https://binary.pensoft.net/file/1756079This dataset is made available under the Open Database License (http://opendatacommons.org/licenses/odbl/1.0/). The Open Database License (ODbL) is a license agreement intended to allow users to freely share, modify, and use this Dataset while maintaining this same freedom for others, provided that the original source and author(s) are credited. Xiang-Fu Liu, Saowaluck Tibpromma, K. W. Thilini Chethana, Li Lu, Kevin D. Hyde, Wen-Hua Lu, Rui-Fang Xu, Xue-Mei Chen, Xian Zhang, Ming-Xiu Chen, Xing-Juan Xiao, Chang-Lin Zhao, Tian-Ye Du, Dong-Qin Dai, Al-Bandari F. Al-Arjani, Abdallah M. Elgorban, Nakarin Suwannarach, Jaturong Kumla, Fu-Qiang Yu, Mei-Mei Wang, Samantha C. Karunarathna

Supplementary material 2 Sequences and alignment files Data type zip https://binary.pensoft.net/file/1756080This dataset is made available under the Open Database License (http://opendatacommons.org/licenses/odbl/1.0/). The Open Database License (ODbL) is a license agreement intended to allow users to freely share, modify, and use this Dataset while maintaining this same freedom for others, provided that the original source and author(s) are credited. Xiang-Fu Liu, Saowaluck Tibpromma, K. W. Thilini Chethana, Li Lu, Kevin D. Hyde, Wen-Hua Lu, Rui-Fang Xu, Xue-Mei Chen, Xian Zhang, Ming-Xiu Chen, Xing-Juan Xiao, Chang-Lin Zhao, Tian-Ye Du, Dong-Qin Dai, Al-Bandari F. Al-Arjani, Abdallah M. Elgorban, Nakarin Suwannarach, Jaturong Kumla, Fu-Qiang Yu, Mei-Mei Wang, Samantha C. Karunarathna
