# Supplementary material for: Culturable mycota from Karst caves in Yunnan Province, China
Source: IMA Fungus. 2026 Aug 26;17:e190343. doi: 10.3897/imafungus.17.190343 (PMC13539301; doi:10.3897/imafungus.17.190343)
Supplement: Supplementary file 2 [file imafungus-17-e190343-s001.doc]

**Supporting information:**

**FIGURE S1**. Phylogenetic trees of *Aspergillus* section *Usti* based on ITS sequences. Bootstrap support values for maximum likelihood (ML) equal to or higher than 70% and Bayesian probability (BYPP) equal to or higher than 0.90 are mentioned above the branches (ML/BI). Newly generated sequences in this study are in blue and red. Type strains are denoted with ^T^.

**FIGURE S2**. Phylogenetic trees of *Aspergillus* section *Usti* based on *BenA* sequences. Newly generated sequences in this study are in blue and red. Bootstrap support values for maximum likelihood (ML) equal to or higher than 70% and Bayesian probability (BYPP) equal to or higher than 0.90 are mentioned above the branches (ML/BI). Newly generated sequences in this study are in blue and red. Type strains are denoted with ^T^.

**FIGURE S3**. Phylogenetic trees of *Aspergillus* section *Usti* based on *rpb*2 sequences. Newly generated sequences in this study are in blue and red. Bootstrap support values for maximum likelihood (ML) equal to or higher than 70% and Bayesian probability (BYPP) equal to or higher than 0.90 are mentioned above the branches (ML/BI). Newly generated sequences in this study are in blue and red. Type strains are denoted with ^T^.

**Supplementary** **Table 1**. Details of the fungal strains included in the phylogenetic analyses.

| **Species** | **Strains** | **GenBank accession numbers** | | | | | | **References** |
| --- | --- | --- | --- | --- | --- | --- | --- | --- |
|  |  | **ITS** | **LSU** | ***tub*2** | ***rpb*2** | ***tef*1-*α*** | ***CaM*** |  |
| *Achaetomiella gracilis* | CBS 146.60 T | KX976648 | KX976743 | KX976990 | KX976842 | – | – | Wang et al. (2022) |
| *Achaetomiella gracilis* | CBS 170.84 | MH861716 | MH873426 | – | – | – | – | Wang et al. (2022) |
| *Achaetomiella gracilis* | CBS 185.83 | MH861570 | MH873300 | – | – | – | – | Wang et al. (2022) |
| *Achaetomiella gracilis* | CBS 249.75 | KX976649 | KX976744 | KX976991 | KX976843 | – | – | Wang et al. (2022) |
| *Achaetomiella gracilis* | CBS 582.84 | MH861783 | MH873481 | – | – | – | – | Wang et al. (2022) |
| *Achaetomiella virescens* | CBS 148.68 T | KX976654 | KX976749 | KX976996 | KX976848 | – | – | Wang et al. (2022) |
| *Achaetomiella virescens* | CBS 547.75 | KX976655 | KX976750 | KX976997 | KX976849 | – | – | Wang et al. (2022) |
| *Achaetomium aegilopis* | IRAN 3453C | MT568841 | MT568844 | MT568852 | – | – | – | Wang et al. (2022) |
| *Achaetomium globosum* | CBS 119.76 | MZ334731 | MZ351416 | MZ343009 | MZ342970 | – | – | Wang et al. (2022) |
| *Achaetomium globosum* | CBS 775.85 T | MZ334727 | – | MZ343031 | MZ342992 | – | – | Wang et al. (2022) |
| *Achaetomium macrosporum* | CBS 102436 T | MZ334718 | AJ312099 | MZ343007 | MZ342966 | – | – | Wang et al. (2022) |
| *Achaetomium strumarium* | CBS 781.84 T | MH861836 | – | MZ343033 | MZ342994 | – | – | Wang et al. (2022) |
| *Acrophialophora angustiphialis* | CGMCC 3.15258 T | KJ026972 | – | KJ147438 | – | – | – | Wang et al. (2022) |
| *Acrophialophora jodhpurensi* | CBS 602.69 T | MK926790 | MK926790 | MK926890 | MK876752 | – | – | Wang et al. (2022) |
| *Acrophialophora levis* | CBS 484.70 T | KP233038 | KM995840 | KP233044 | – | – | – | Wang et al. (2022) |
| *Acrophialophora major* | GZUIFR-H57-2 T | MK926792 | MK926792 | MK926892 | MK876754 | – | – | Wang et al. (2022) |
| *Acrophialophora nainiana* | CBS 100.60 T | MK926793 | MK926793 | MK926893 | MK876755 | – | – | Wang et al. (2022) |
| *Acrophialophora teleoafricana* | CBS 281.79 T | MK926795 | MK926795 | MK926895 | MK876757 | – | – | Wang et al. (2022) |
| *Allobotryotrichum blastosporum* | CGMCC 3.19343 T | MN215716 | MN215554 | MN329887 | MN255397 | – | – | Wang et al. (2022) |
| *Allobotryotrichum blastosporum* | LC11913 | MN215717 | MN215555 | MN329888 | MN255398 | – | – | Wang et al. (2022) |
| *Allocanariomyces americanus* | UTHSCSA DI20-139 T | MT902181 | MT902391 | MT904876 | MT904877 | – | – | Wang et al. (2022) |
| *Allocanariomyces tritici* | IRAN 3450C T | MT568839 | MT568842 | MT568850 | MT568845 | – | – | Wang et al. (2022) |
| *Allocanariomyces tritici* | IRAN 4014C | MT568840 | MT568843 | MT568851 | MT568846 | – | – | Wang et al. (2022) |
| *Amesia atrobrunnea* | CBS 379.66 T | JX280771 | JX280666 | KX976916 | KX976798 | – | – | Wang et al. (2022) |
| *Amesia dreyfussii* | CBS 376.83 | MH861613 | MH873331 | MZ343023 | MZ342985 | – | – | Wang et al. (2022) |
| *Amesia gelasinospora* | CBS 552.83 | – | – | MZ343026 | MZ342987 | – | – | Wang et al. (2022) |
| *Amesia raii* | CBS 106.83 | – | – | – | MZ342967 | – | – | Wang et al. (2022) |
| *Amesia raii* | CBS 107.83 | – | – | – | MZ342968 | – | – | Wang et al. (2022) |
| *Annulohypoxylon annulatum* | CBS 140775 | KY610418 | KY610418 | KX376353 | KY624263 | – | – | Wendt et al. (2018) |
| *Annulohypoxylon moriforme* | CBS 123579 | KX376321 | KY610425 | KX271261 | KY624289 | – | – | Wendt et al. (2018) |
| *Annulohypoxylon nitens* | MFLUCC 12.0823 | KJ934991 | KJ934992 | KJ934993 | KJ934994 | – | – | Daranagama et al. (2015) |
| *Annulohypoxylon stygium* | MUCL 54601 | KY610409 | KY610475 | KX271263 | KY624292 | – | – | Wendt et al. (2018) |
| *Annulohypoxylon truncatum* | CBS 140778 | KY610419 | KY610419 | KX376352 | KY624277 | – | – | Wendt et al. (2018) |
| *Aporothielavia leptoderma* | CBS 113678 | – | MZ351417 | MZ343008 | MZ342969 | – | – | Wang et al. (2022) |
| *Aporothielavia leptoderma* | CBS 538.74 T | NR_164219 | NG_067253 | MZ343025 | MZ342986 | – | – | Wang et al. (2022) |
| *Arachnocrea scabrida* | BEO 0201 | – | – | – | DQ834458 | DQ834457 |  | Zeng and Zhuang (2019) |
| *Arachnocrea stipata* | TFC 9743 | – | – | – | EU710770 | – | – | Zeng and Zhuang (2019) |
| *Arcopilus albae* | MFLUCC 24–0064T | PP766582 | PP766589 | PP801102 | – | – | – | Wang et al. (2022) |
| *Arcopilus amazonicus* | AM2410 | MH777083 | MH780043 | MH784466 | MH784457 | – | – | Sousa et al. (2020) |
| *Arcopilus amazonicus* | AM2417 | MH777090 | MH780050 | MH784473 | MH784464 | – | – | Sousa et al. (2020) |
| *Arcopilus amazonicus* | AM2418 | MH777091 | MH780051 | MH784474 | MH784465 | – | – | Sousa et al. (2020) |
| *Arcopilus aureus* | CBS 153.52 | KX976582 | KX976707 | KX976924 | KX976806 | – | – | Wang et al. (2016) |
| *Arcopilus aureus* | CBS 538.73 | KX976583 | KX976708 | KX976925 | KX976807 | – | – | Wang et al. (2016) |
| *Arcopilus cupreus* | CBS 560.80 | KX976584 | KX976709 | KX976926 | KX976808 | – | – | Wang et al. (2016) |
| *Arcopilus eremanthusum* | CML 3766 T | MN539886 | MN539910 | – | MN551186 | – | – | Tavares et al. (2022) |
| *Arcopilus flavigenus* | CBS 337.67 T | KX976587 | KX976712 | KX976929 | KX976811 | – | – | Wang et al. (2022) |
| *Arcopilus fusiformis* | CBS 484.85 | KX976585 | KX976710 | KX976927 | KX976809 | – | – | Wang et al. (2022) |
| *Arcopilus fusiformis* | CBS 485.85 | KX976586 | KX976711 | KX976928 | KX976810 | – | – | Wang et al. (2022) |
| *Arcopilus globulus* | CGMCC3.19359 | MN215741 | MN215579 | MZ343038 | MN255422 | – | – | Wang et al. (2022) |
| *Arcopilus globulus* | LC13554 | MW237705 | MN215580 | MN329915 | MN255423 | – | – | Wang et al. (2022) |
| *Arcopilus hongheensis* | KUNCC22-10767T | OR117280 | OR117285 | OR119776 | OR119771 | – | – | Yang et al. (2023) |
| *Arcopilus hongheensis* | KUNCC23-13346 | OR117279 | OR117284 | OR119775 | OR119770 | – | – | Yang et al. (2023) |
| *Arcopilus macrostiolatus* | CBS 102435T | MZ334722 | MZ351418 | MZ343006 | MZ342965 | – | – | Wang et al. (2022) |
| *Arcopilus megasporus* | CBS 127650 | – | – | MZ343010 | MZ342971 | – | – | Wang et al. (2022) |
| *Arcopilus navicularis* | CCF 3252 | MW798185 | MW798181 | MW816125 | MW816124 | – | – | Wang et al. (2022) |
| *Arcopilus purpurascens* | CBS 287.83 T | – | – | MZ343021 | MZ342982 | – | – | Wang et al. (2022) |
| *Arcopilus tangerinicapillus* | CGMCC 3.19326 T | MN215743 | MN215581 | MN329904 | MN255424 | – | – | Wang et al. (2022) |
| *Arcopilus turgidopilosus* | CBS 169.52 T | KX976588 | KX976713 | KX976930 | KX976812 | – | – | Wang et al. (2022) |
| *Arxotrichum deceptivum* | CBS 346.73 T | MK919276 | MK919276 | MK919390 | MK919332 | – | – | Wang et al. (2022) |
| *Arxotrichum gangligerum* | CBS 160.52 T | MK919277 | MK919277 | MK919391 | MK919333 | – | – | Wang et al. (2022) |
| *Arxotrichum officinarum* | CGMCC 3.19325 T | MN215767 | MN215605 | MN337032 | MN255448 | – | – | Wang et al. (2022) |
| *Arxotrichum sinense* | CBS 541.83 T | MK919283 | MK919283 | MK919397 | MK919339 | – | – | Wang et al. (2022) |
| *Arxotrichum succineum* | CBS 166.52 T | MK919284 | MK919284 | MK919398 | MK919340 | – | – | Wang et al. (2022) |
| *Arxotrichum wyomingense* | CCF 5691 T | LT968153 | LT968143 | LT971393 | – | – | – | Wang et al. (2022) |
| *Aspergillus asper* | CBS 140842 T | KT698840 | – | KT698838 | KT698842 | – | KT698839 | Sun et al. (2020) |
| *Aspergillus baeticus* | CMF 2181 | HE617298 | – | HE615094 | – | – | HE615119 | Novákova et al. (2012) |
| *Aspergillus baeticus* | NRRL 62487 | HE615087 | – | HE615093 | HE615125 | – | HE615118 | Novákova et al. (2012) |
| *Aspergillus baeticus* | NRRL 62501 T | HE615086 | – | HE615092 | HE615124 | – | HE615117 | Novákova et al. (2012) |
| ***Aspergillus baeticus*** | **GMBCC2264** | **PZ669373** | **–** | **PZ870993** | **PZ867796** | **–** | **–** | **This study** |
| *Aspergillus calidoustus* | CBS 112452 | MN650833 | – | MN735972 | MN735957 | – | MN735938 | Sun et al. (2020) |
| *Aspergillus calidoustus* | CBS 113228 | EF591739 | – | EF591730 | MN735959 | – | EF591715 | Sun et al. (2020) |
| *Aspergillus calidoustus* | CBS 114380 | EF591741 | – | EF591729 | MN735958 | – | EF591716 | Sun et al. (2020) |
| *Aspergillus calidoustus* | CBS 121601 T | HE616558 | – | FJ624456 | MN735956 | – | HE616559 | Sun et al. (2020) |
| *Aspergillus calidoustus* | CGMCC 3.05298 | MN650835 | – | MN735974 | MN735961 | – | MN735940 | Sun et al. (2020) |
| *Aspergillus calidoustus* | CGMCC 3.05313 | MN650836 | – | MN735975 | MN735962 | – | MN735941 | Sun et al. (2020) |
| *Aspergillus calidoustus* | CGMCC 3.08405 | MN650834 | – | MN735973 | MN735960 | – | MN735939 | Sun et al. (2020) |
| *Aspergillus californicus* | CBS 123895 T | FJ531153 | – | FJ531180 | KU866974 | – | FJ531128 | Sun et al. (2020) |
| *Aspergillus carlsbadensis* | CBS 123893 | FJ531152 | – | FJ531178 | – | – | FJ531125 | Sun et al. (2020) |
| *Aspergillus carlsbadensis* | CBS 123894 T | FJ531151 | – | FJ531179 | KU866973 | – | FJ531126 | Sun et al. (2020) |
| *Aspergillus carlsbadensis* | CBS 123903 | FJ531148 | – | FJ531169 | MN735963 | – | FJ531124 | Sun et al. (2020) |
| *Aspergillus cavernicola* | CBS 117.76 T | EF652508 | – | EF652332 | EF652244 | – | EF652420 | Sun et al. (2020) |
| *Aspergillus cavernicola* | CBS 600.67 | FJ531161 | – | FJ531190 | JN121538 | – | FJ531140 | Sun et al. (2020) |
| *Aspergillus cavernicola* | NRRL 5813 | EF652503 | – | – | EF652239 | – | EF652415 | Sun et al. (2020) |
| *Aspergillus collinsii* | CBS 140843 T | KT698845 | – | KT698843 | KT698848 |  | KT698844 | Sun et al. (2020) |
| *Aspergillus collinsii* | CBS 141578 | MN640759 | – | MN635245 | MN635268 | – | MN635256 | Sun et al. (2020) |
| *Aspergillus deflectus* | CBS 109.55 T | EF652437 | – | EF652261 | EF652173 | – | EF652349 | Sun et al. (2020) |
| *Aspergillus deflectus* | NRRL 4235 | EF652463 | – | EF652287 | EF652199 | – | EF652375 | Sun et al. (2020) |
| ***Aspergillus dehongensis*** | **GMBCC2269 T** | **PZ669374** | **–** | **PZ870994** | **PZ867797** | **–** | **–** | **This study** |
| ***Aspergillus dehongensis*** | **GZCC 25-0737** | **PZ669375** | **–** | **PZ870995** | **PZ867798** | **–** | **–** | **This study** |
| ***Aspergillus dehongensis*** | **GZCC 25-0738** | **PZ669376** | **–** | **PZ870996** | **PZ867799** | **–** | **–** | **This study** |
| ***Aspergillus dehongensis*** | **GZCC 25-0736** | **PZ669377** | **–** | **PZ870997** | **PZ867800** | **–** | **–** | **This study** |
| *Aspergillus egyptiacus* | CBS 656.73 T | EF652504 | – | EF652328 | EF652240 | – | FJ531132 | Sun et al. (2020) |
| *Aspergillus egyptiacus* | CBS 991.72A | FJ531162 | – | FJ531187 | MN735954 | – | MN735936 | Sun et al. (2020) |
| *Aspergillus egyptiacus* | CBS 991.72B | FJ531163 | – | FJ531184 | – | – | FJ531135 | Sun et al. (2020) |
| *Aspergillus egyptiacus* | CBS 991.72C | MH860638 | – | FJ531189 | – | – | MN735937 | Sun et al. (2020) |
| *Aspergillus egyptiacus* | CBS 991.72E | FJ531165 | – | FJ531188 | – | – | FJ531137 | Sun et al. (2020) |
| *Aspergillus egyptiacus* | CBS 991.72F | MN650832 | – | FJ531185 | MN735955 | – | FJ531133 | Sun et al. (2020) |
| *Aspergillus elongatus* | CBS 387.75 T | EF652502 | – | EF652326 | EF652238 | – | EF652414 | Sun et al. (2020) |
| *Aspergillus germanicus* | CBS 123887 T | FJ531146 | – | FJ531172 | KU866944 | – | FJ531141 | Sun et al. (2020) |
| *Aspergillus germanicus* | DTO 179- B4 | MN650837 | – | KP329848 | MN735964 | – | MN735942 | Sun et al. (2020) |
| *Aspergillus germanicus* | DTO 179- B7 | MN650838 | – | KP329849 | MN735965 | – | MN735943 | Sun et al. (2020) |
| *Aspergillus granulosus* | CBS 119.58 | MH857715 | – | EF591735 | – | – | MN735944 | Sun et al. (2020) |
| *Aspergillus granulosus* | NRRL 1932 T | EF652430 | – | EF652254 | EF652166 | – | EF652342 | Sun et al. (2020) |
| *Aspergillus heterothallicus* | CBS 488.65 T | EF652499 | – | EF652323 | EF652235 | – | EF652411 | Sun et al. (2020) |
| *Aspergillus heterothallicus* | CBS 489.65 | LN827680 | – | EU076369 | –– | – | EU076361 | Sun et al. (2020) |
| *Aspergillus insuetus* | CBS 102278 | EU076357 | – | MN735976 | MN735967 | – | MN735945 | Sun et al. (2020) |
| *Aspergillus insuetus* | CBS 107.25 T | EF652457 | – | EF652281 | EF652193 | – | EF652369 | Sun et al. (2020) |
| *Aspergillus insuetus* | CBS 119.27 | EU076355 | – | EU076372 | MN735966 | – | EU076367 | Sun et al. (2020) |
| *Aspergillus insuetus* | CGMCC 3.05503 | MN650839 | – | MN735977 | – | – | MN735946 | Sun et al. (2020) |
| *Aspergillus kassunensis* | CBS 419.69 T | EF652461 | – | EF652285 | EF652197 | – | EF652373 | Sun et al. (2020) |
| *Aspergillus keveii* | CBS 113227 | EU076353 | – | EU076373 | – | – | EU076363 | Sun et al. (2020) |
| *Aspergillus keveii* | CBS 209.92 T | EU076354 | – | EU076376 | KU866938 | – | EU076365 | Sun et al. (2020) |
| *Aspergillus keveii* | CBS 561.65 | EU076352 | – | EU076375 | MN735968 | – | EU076364 | Sun et al. (2020) |
| *Aspergillus keveioides* | CBS 132737 T | JN982704 | – | JN982694 | KX423660 | – | JN982684 | Sun et al. (2020) |
| *Aspergillus lucknowensis* | CBS 449.75 T | EF652459 | – | EF652283 | EF652195 | – | EF652371 | Sun et al. (2020) |
| *Aspergillus monodii* | CBS 434.93 | FJ531149 | – | FJ531170 | – | – | FJ531143 | Sun et al. (2020) |
| *Aspergillus monodii* | CBS 435.93 T | FJ531150 | – | FJ531171 | MN969082 | – | FJ531142 | Sun et al. (2020) |
| *Aspergillus ochraceoroseus* | CBS 550.77 T | EF661224 | – | EF661113 | EF661074 | – | EF661137 | Sun et al. (2020) |
| *Aspergillus porphyreostipitatus* | CBS 138202 | KJ775563 | – | KJ775071 | – | – | KJ775260 | Sun et al. (2020) |
| *Aspergillus porphyreostipitatus* | CBS 138203 T | KJ775564 | – | KJ775080 | KU866987 | – | KJ775338 | Sun et al. (2020) |
| *Aspergillus pseudodeflectus* | CBS 596.65 | EF591742 | – | EF591732 | MN735970 | – | MN735947 | Sun et al. (2020) |
| *Aspergillus pseudodeflectus* | CBS 756.74 T | EF652507 | – | EF652331 | EF652243 | – | EF652419 | Sun et al. (2020) |
| *Aspergillus pseudoustus* | CBS 123904 T | FJ531147 | – | FJ531168 | KU866978 | – | FJ531129 | Sun et al. (2020) |
| *Aspergillus pseudoustus* | DTO 148- G1 | MN650840 | – | MN735978 | – | – | MN735948 | Sun et al. (2020) |
| *Aspergillus puniceus* | CBS 122.33 | MH855382 | – | EF591723 | – | – | MN735949 | Sun et al. (2020) |
| *Aspergillus puniceus* | CBS 495.65 T | EF652498 | – | EF652322 | EF652234 | – | EF652410 | Sun et al. (2020) |
| *Aspergillus sigarelli* | CBS 141579 T | MN640758 | – | MN635244 | MN635267 | – | MN635255 | Sun et al. (2020) |
| *Aspergillus subsessilis* | CBS 502.65 T | EF652485 | – | EF652309 | EF652221 | – | EF652397 | Sun et al. (2020) |
| *Aspergillus subsessilis* | CBS 987.72 | EF652486 | – | EF652310 | EF652222 | – | EF652398 | Sun et al. (2020) |
| *Aspergillus subsessilis* | CBS 988.72 | FJ531157 | – | FJ531186 | EF652223 | – | FJ531136 | Sun et al. (2020) |
| *Aspergillus thesauricus* | CMF ISB 2155 T | HE615088 | – | HE615095 | HE615126 | – | HE615120 | Nováková et al. (2012) |
| *Aspergillus thesauricus* | CMF ISB 2156 | HE615089 | – | HE615096 | HE615127 | – | HE615121 | Nováková et al. (2012) |
| *Aspergillus turkensis* | CBS 504.65 T | FJ531160 | – | FJ531191 | EF652230 | – | FJ531145 | Sun et al. (2020) |
| *Aspergillus ustus* | CBS 114901 | MN650841 | – | MN735980 | – | – | MN735951 | Sun et al. (2020) |
| *Aspergillus ustus* | CBS 116057 | EU076358 | – | MN735979 | MN735971 | – | MN735950 | Sun et al. (2020) |
| *Aspergillus ustus* | CBS 133.55 | MH857414 | – | MN735981 | – | – | MN735952 | Sun et al. (2020) |
| *Aspergillus ustus* | CBS 239.90 | MN650842 | – | MN735982 | – | – | MN735953 | Sun et al. (2020) |
| *Aspergillus ustus* | CBS 261.67 T | EF652455 | – | EF652279 | EF652191 | – | EF652367 | Sun et al. (2020) |
| *Batnamyces globulariicola* | CBS 144474 T | MT075917 | MT075917 | MT075919 | MT075918 | – | – | Wang et al. (2022) |
| *Bommerella trigonospora* | CBS 324.69 | – | MZ351419 | MZ343022 | MZ342984 | – | – | Wang et al. (2022) |
| *Botryoderma lateritium* | CBS 586.66 T | MK919287 | MK919287 | MK919401 | MK919343 | – | – | Wang et al. (2022) |
| *Botryoderma rostratum* | CBS 184.68 T | MK919288 | MK919288 | MK919402 | MK919344 | – | – | Wang et al. (2022) |
| *Botryotrichum geniculatum* | CBS 144475 | MZ334719 | MZ351422 | MZ343011 | MZ342972 | – | – | Wang et al. (2022) |
| *Botryotrichum geniculatum* | WXW8266 | MZ334720 | MZ351423 | MZ343039 | MZ343000 | – | – | Wang et al. (2022) |
| *Botryotrichum inquinatum* | CBS 155.80 | MK919289 | MK919289 | MK919403 | MK919345 | – | – | Wang et al. (2022) |
| *Botryotrichum inquinatum* | CBS 646.74 | MK919290 | MK919290 | MK919404 | MK919346 | – | – | Wang et al. (2022) |
| *Botryotrichum retardatum* | CBS 197.84 | – | – | MZ343019 | MZ342980 | – | – | Wang et al. (2022) |
| *Botryotrichum trichorobustum* | CBS 563.67 | – | MZ351420 | MZ343027 | MZ342988 | – | – | Wang et al. (2022) |
| *Botryotrichum verrucosum* | CBS 116.64 T | LT993567 | LT993567 | LT993648 | LT993486 | – | – | Wang et al. (2022) |
| *Botryotrichum vitellinum* | CBS 180.84 | MZ334725 | MZ351421 | MZ343018 | MZ342979 | – | – | Wang et al. (2022) |
| *Brachychaeta variospora* | CBS 414.73 T | MK926797 | MK926797 | MK926897 | MK876759 | – | – | Wang et al. (2022) |
| *Canariomyces arenarius* | CBS 507.74 T | MK926798 | MK926798 | MK926898 | KM655438 | – | – | Wang et al. (2022) |
| *Canariomyces microsporus* | CBS 276.74 T | MK926799 | MK926799 | MK926899 | MK876760 | – | – | Wang et al. (2022) |
| *Canariomyces notabilis* | CBS 548.83 T | MK926802 | MK926802 | MK926902 | MK876763 | – | – | Wang et al. (2022) |
| *Canariomyces subthermophilus* | CBS 509.74 T | MK926804 | MK926804 | MK926904 | MK876764 | – | – | Wang et al. (2022) |
| *Canariomyces vonarxii* | CBS 160.80 T | MK926805 | MK926805 | MK926905 | MK876765 | – | – | Wang et al. (2022) |
| *Carteria arctostaphyli* | CBS 229.82 T | MK926807 | MK926807 | MK926907 | MK876767 | – | – | Wang et al. (2022) |
| *Chaetomium afropilosum* | CBS 145.38 T | KT214574 | KT214605 | KT214751 | KT214675 | – | – | Wang et al. (2022) |
| *Chaetomium afropilosum* | LC13503 | MN215746 | MN215584 | MN337029 | MN255427 | – | – | Wang et al. (2022) |
| *Chaetomium anastomosans* | CGMCC3.19350 T | MN215745 | MN215583 | MN337028 | MN255426 | – | – | Wang et al. (2022) |
| *Chaetomium angustispirale* | CBS 137.58 T | JN209862 | JN209862 | JN256141 | KF001824 | – | – | Wang et al. (2022) |
| *Chaetomium ascotrichoides* | CBS 113.83 T | KC109752 | KC109752 | KC109770 | KF001832 | – | – | Wang et al. (2022) |
| *Chaetomium camelliae* | JZBH3340001 T | MT535751 | MT535749 | MT535533 | MT535537 | – | – | Wang et al. (2022) |
| *Chaetomium camelliae* | JZBH3340002 | MT535752 | MT535750 | MT535534 | MT535538 | – | – | Wang et al. (2022) |
| *Chaetomium capillare* | CBS 128489 T | KT214583 | KT214614 | KT214760 | KT214686 | – | – | Wang et al. (2022) |
| *Chaetomium cervicicola* | CBS 128492 T | KT214558 | KT214592 | KT214735 | KT214662 | – | – | Wang et al. (2022) |
| *Chaetomium citrinum* | CBS 693.82 T | KT214587 | KT214617 | KT214764 | KT214691 | – | – | Wang et al. (2022) |
| *Chaetomium coarctatum* | MUCL 18697 T | JN209863 | JN209863 | JN256142 | KF001802 | – | – | Wang et al. (2022) |
| *Chaetomium cochliodes* | CBS 155.52 ET | KC109754 | KC109754 | KC109772 | KF001811 | – | – | Wang et al. (2022) |
| *Chaetomium concavisporum* | LC11924 T | MN215747 | MN215585 | MN329916 | MN255428 | – | – | Wang et al. (2022) |
| *Chaetomium concavisporum* | LC13552 | MN215748 | MN215586 | MN329917 | MN255429 | – | – | Wang et al. (2022) |
| *Chaetomium contagiosum* | CBS 128494 T | KT214555 | KT214589 | KT214732 | KT214659 | – | – | Wang et al. (2022) |
| *Chaetomium cucumericola* | CBS 378.71 T | KT214579 | KT214610 | KT214756 | KT214680 | – | – | Wang et al. (2022) |
| *Chaetomium elatum* | CBS 142034 NeoT | KX976612 | KX976733 | KX976954 | KX976832 | – | – | Wang et al. (2022) |
| *Chaetomium fifimeti* | CBS 139034 EpiT | KT214559 | KT214593 | KT214736 | KT214663 | – | – | Wang et al. (2022) |
| *Chaetomium globosum* | CBS 105.40 | KT214566 | KT214597 | KT214743 | KT214667 | – | – | Wang et al. (2022) |
| *Chaetomium globosum* | CBS 132.30 | KC109755 | KC109755 | KC109773 | KF001792 | – | – | Wang et al. (2022) |
| *Chaetomium globosum* | CBS 147.60 | JN209909 | JN209909 | JN256179 | KF001793 | – | – | Wang et al. (2022) |
| *Chaetomium globosum* | CBS 148.51 | GU563374 | GU563363 | JF772459 | KF001801 | – | – | Wang et al. (2022) |
| *Chaetomium globosum* | CBS 160.62 T | KT214565 | KT214596 | KT214742 | KT214666 | – | – | Wang et al. (2022) |
| *Chaetomium globosum* | CBS 164.62 | JN209920 | JN209920 | JN256190 | KF001796 | – | – | Wang et al. (2022) |
| *Chaetomium globosum* | CBS 371.66 | JN209871 | JN209871 | JN256148 | KF001795 | – | – | Wang et al. (2022) |
| *Chaetomium globosum* | CBS 733.84 | MH861830 | MH873520 | – | – | – | – | Wang et al. (2022) |
| *Chaetomium globosum* | CGMCC 3.9994 | JN209894 | JN209894 | JN256168 | KF001798 | – | – | Wang et al. (2022) |
| *Chaetomium globosum* | MUCL 39526 | JN209875 | JN209875 | JN256152 | KF001800 | – | – | Wang et al. (2022) |
| *Chaetomium globosum* | MUCL 39527 | JN209899 | JN209899 | JN256173 | KF001799 | – | – | Wang et al. (2022) |
| *Chaetomium graminiforme* | CBS 506.84 T | KT214584 | KT214615 | KT214761 | KT214687 | – | – | Wang et al. (2022) |
| *Chaetomium grande* | CBS 126780 T | HM365253 | HM365253 | HM365273 | KT214657 | – | – | Wang et al. (2022) |
| *Chaetomium hongheensis* | KUMCC22-10764 | OR129667 | OR129658 | OR125091 | OR125095 | – | – | Yang et al. (2024) |
| *Chaetomium hongheensis* | KUNCC22-10750 T | OR129668 | OR129659 | OR125092 | OR125096 | – | – | Wang et al. (2022) |
| *Chaetomium interruptum* | CBS 126660 T | HM365246 | HM365246 | KT214741 | KT214665 | – | – | Wang et al. (2022) |
| *Chaetomium madrasense* | CBS 315.74 T | KC109751 | KC109751 | KC109769 | KF001831 | – | – | Wang et al. (2022) |
| *Chaetomium megalocarpum* | CBS 149.59 T | KC109744 | KC109744 | KC109762 | KF001828 | – | – | Wang et al. (2022) |
| *Chaetomium meridionalense* | COAD 3122 | ON989661 | ON979680 | ON988188 | OP131291 | – | – | Wang et al. (2022) |
| *Chaetomium meridionalense* | COAD 3451 T | ON989660 | ON979679 | ON988187 | OP131292 | – | – | Wang et al. (2022) |
| *Chaetomium neoglobosporum* | CBS 108.83 T | KC109750 | KC109750 | KC109768 | KF001825 | – | – | Wang et al. (2022) |
| *Chaetomium novozelandicum* | CBS 124555 T | KT214576 | KT214607 | KT214753 | KT214677 | – | – | Wang et al. (2022) |
| *Chaetomium nozdrenkoae* | CBS 163.62 T | KT214556 | KT214590 | KT214733 | KT214660 | – | – | Wang et al. (2022) |
| *Chaetomium olivaceum* | CBS 418.80A T | JN209914 | JN209914 | JN256184 | KF001806 | – | – | Wang et al. (2022) |
| *Chaetomium olivaceum* | CGMCC 3.12883 | JN209911 | JN209911 | JN256181 | KF001807 | – | – | Wang et al. (2022) |
| *Chaetomium olivicolor* | CBS 102434 T | KM655318 | KM655357 | – | KM655428 | – | – | Wang et al. (2022) |
| *Chaetomium ovatoascomatis* | LC13504 | MN215754 | MN215592 | MN329921 | MN255435 | – | – | Wang et al. (2022) |
| *Chaetomium ovatoascomatis* | LC13510 T | MN215753 | MN215591 | MN329920 | MN255434 | – | – | Wang et al. (2022) |
| *Chaetomium pilosum* | CBS 335.67 T | KT214586 | FJ666356 | KT214763 | FJ666387 | – | – | Wang et al. (2022) |
| *Chaetomium pseudocochliodes* | CGMCC 3.9441 T | JN209925 | JN209925 | JN256195 | KF001816 | – | – | Wang et al. (2022) |
| *Chaetomium pseudoglobosum* | CBS 574.71 T | KT214573 | KT214604 | KT214750 | KT214674 | – | – | Wang et al. (2022) |
| *Chaetomium rectangulare* | CBS 126778 T | HM365239 | HM365239 | HM365285 | KT214688 | – | – | Wang et al. (2022) |
| *Chaetomium sacchari* | LC11916 | MN215760 | MN215598 | MN329927 | MN255441 | – | – | Wang et al. (2022) |
| *Chaetomium sacchari* | LC11918 T | MN215759 | MN215597 | MN329926 | MN255440 | – | – | Wang et al. (2022) |
| *Chaetomium spiculipilium* | CBS 373.66 T | KC109756 | KC109756 | KC109774 | KF001809 | – | – | Wang et al. (2022) |
| *Chaetomium spirochaete* | CBS 730.84 ET | JN209921 | JN209921 | JN256191 | KF001819 | – | – | Wang et al. (2022) |
| *Chaetomium subaffine* | 22N617 | – | OR794364 | OR805129 | OR805130 | – | – | Wang et al. (2022) |
| *Chaetomium subaffine* | CBS 637.91 T | JN209929 | MH873969 | JN256199 | KF001817 | – | – | Wang et al. (2022) |
| ***Chaetomium subaffine*** | **GMBCC2271** | **PZ669401** | **PZ669414** | **PZ871003** | **PZ867822** | **–** | **–** | **This study** |
| *Chaetomium subglobosum* | CBS 149.60 T | JN209930 | JN209930 | JN256200 | KF001808 | – | – | Wang et al. (2022) |
| *Chaetomium tectififimeti* | CBS 142032 T | KX976640 | KX976737 | KX976982 | KX976836 | – | – | Wang et al. (2022) |
| *Chaetomium telluricola* | CBS 151.59 T | KT214582 | KT214613 | KT214759 | KT214685 | – | – | Wang et al. (2022) |
| *Chaetomium tenue* | CBS 139.38 T | KT214568 | KT214599 | KT214745 | KT214669 | – | – | Wang et al. (2022) |
| *Chaetomium ubfifimeti* | CBS 370.66 T | KT214562 | FJ666354 | KT214739 | FJ666385 | – | – | Wang et al. (2022) |
| *Chaetomium undulatulum* | CBS 126775 T | HM365251 | HM365251 | HM365279 | KT214682 | – | – | Wang et al. (2022) |
| *Chaetomium unguicola* | CBS 128446 T | KT214567 | KT214598 | KT214744 | KT214668 | – | – | Wang et al. (2022) |
| *Chrysanthotrichum allolentum* | CBS 644.83 T | MK926808 | MK926808 | MK926908 | MK876768 | – | – | Wang et al. (2022) |
| *Chrysanthotrichum lentum* | CBS 339.67 T | MK926809 | MK926809 | MK926909 | MK876769 | – | – | Wang et al. (2022) |
| *Chrysanthotrichum leptolentum* | CBS 126.85 T | MK926810 | MK926810 | MK926910 | MK876770 | – | – | Wang et al. (2022) |
| *Chrysanthotrichum peruvianum* | CBS 732.68 T | MK926812 | MK926812 | MK926912 | MK876772 | – | – | Wang et al. (2022) |
| *Chrysocorona lucknowensis* | CBS 727.71 T | MK926813 | MK926813 | MK926913 | MK876773 | – | – | Wang et al. (2022) |
| *Collariella anguipilia* | CBS 632.83 T | MZ334721 | MZ351424 | MZ343028 | MZ342989 | – | – | Wang et al. (2022) |
| *Collariella bostrychodes* | CBS 121706 | KX976645 | – | KX976987 | – | – | – | Wang et al. (2022) |
| *Collariella bostrychodes* | CBS 163.73 T | KX976641 | MH877815 | KX976983 | KX976837 | – | – | Wang et al. (2022) |
| *Collariella bostrychodes* | CBS 586.83 | KX976642 | KX976739 | KX976984 | KX976838 | – | – | Wang et al. (2022) |
| *Collariella bostrychodes* | DTO 319-C4 | KX976643 | – | KX976985 | – | – | – | Wang et al. (2022) |
| *Collariella bostrychodes* | DTO 324-H3 | KX976644 | KX976740 | KX976986 | KX976839 | – | – | Wang et al. (2022) |
| *Collariella capillicompacta* | IRAN 3496C T | – | – | MN520423 | MN520427 | – | – | Wang et al. (2022) |
| *Collariella carteri* | CBS 128.85 T | KX976647 | KX976742 | KX976989 | KX976841 | – | – | Wang et al. (2022) |
| *Collariella causiiformis* | CBS 792.83 T | KX976646 | KX976741 | KX976988 | KX976840 | – | – | Wang et al. (2022) |
| *Collariella hexagonospora* | TRTC 48872 T | MH861717 | – | MZ343016 | MZ342977 | – | – | Wang et al. (2022) |
| *Collariella hilkhuijsenii* | JW16019 T | MG432011 | MG432012 | MF716586 | MF716587 | – | – | Wang et al. (2022) |
| *Collariella pachypodioides* | CBS 164.52 T | MH856980 | MH868500 | MZ343014 | MZ342975 | – | – | Wang et al. (2022) |
| *Collariella quadrangulata* | CBS 142.58 | KX976650 | KX976745 | KX976992 | KX976844 | – | – | Wang et al. (2022) |
| *Collariella quadrangulata* | CBS 152.59 T | KX976651 | KX976746 | KX976993 | KX976845 | – | – | Wang et al. (2022) |
| *Collariella quadrum* | CGMCC 3.17917 T | KU746675 | KU746721 | KU746767 | KY575870 | – | – | Wang et al. (2022) |
| *Collariella quadrum* | CGMCC 3.17918 | KU746676 | KU746722 | KU746768 | KY575871 | – | – | Wang et al. (2022) |
| *Collariella quadrum* | CGMCC 3.17919 | KU746677 | KU746723 | KU746769 | KY575872 | – | – | Wang et al. (2022) |
| *Collariella robusta* | CBS 551.83 T | KX976652 | KX976747 | KX976994 | KX976846 | – | – | Wang et al. (2022) |
| *Collariella robusta* | CBS 551.84 | KX976653 | KX976748 | KX976995 | KX976847 | – | – | Wang et al. (2022) |
| *Condenascus tortuosus* | CBS 610.97 | MK926817 | – | MK926917 | – | – | – | Wang et al. (2022) |
| *Corynascella humicola* | CBS 337.72 T | KX976656 | KX976751 | KX976998 | KX976850 | – | – | Wang et al. (2022) |
| *Corynascella humicola* | CBS 379.74 | KX976657 | KX976752 | KX976999 | KX976851 | – | – | Wang et al. (2022) |
| *Corynascus fumimontanus* | CBS 137294 T | MK919291 | MK919291 | MK919405 | MK919347 | – | – | Wang et al. (2022) |
| *Corynascus novoguineensis* | CBS 359.72 T | MK919292 | MK919292 | MK919406 | MK919348 | – | – | Wang et al. (2022) |
| *Corynascus sepedonium* | CBS 340.33 T | HQ871751 | KX976777 | KX977027 | MK919349 | – | – | Wang et al. (2022) |
| *Corynascus sexualis* | CBS 827.96 T | MK919295 | MK919295 | MK919409 | MK919352 | – | – | Wang et al. (2022) |
| *Cosmospora annulohypoxyli* | G.J.S. 95-199 | – | – | KJ676284 | – | – | – | Czachura and Janik (2025) |
| *Cosmospora annulohypoxyli* | G.J.S. 96-186 | – | – | KJ676285 | – | – | – | Czachura and Janik (2025) |
| *Cosmospora aquatica* | MFLU 15-0072 | NR_168211 | – | – | MN194020 | – | – | Luo et al. (2019) |
| *Cosmospora aquatica* | MFLUCC 13-0884 T | MK828671 | MK828238 | – | – | – | – | Luo et al. (2019) |
| *Cosmospora aquatica* | MFLUCC 15-0244 | MK828670 | MK828237 | – | – | – | – | Luo et al. (2019) |
| *Cosmospora arxii* | A.R. 4521 | – | – | KJ676255 | – | – | – | Czachura and Janik (2025) |
| *Cosmospora arxii* | CBS 748.69 T | KM231819 | KM231694 | KM232089 | HQ897725 | – | – | Czachura and Janik (2025) |
| *Cosmospora butyri* | CBS 301.38 T | MW827605 | MW827644 | – | HQ897729 | – | – | Czachura and Janik (2025) |
| ***Cosmospora cavernicola*** | **GZCC 25-0728 T** | **PZ669394** | **PZ669407** | **PZ870998** | **PZ867817** | **–** | – | **This study** |
| ***Cosmospora cavernicola*** | **GZCC 25-0727** | **PZ669395** | **PZ669408** | **PZ870999** | **PZ867818** | **–** | – | **This study** |
| ***Cosmospora cavernicola*** | **GMBCC2272** | **PZ669396** | **PZ669409** | **PZ871000** | **PZ867819** | **–** | – | **This study** |
| *Cosmospora clavi* | CBS 123941 | KJ676149 | KJ676186 | KJ676262 | – | – | – | Czachura and Janik (2025) |
| *Cosmospora clavi* | CBS 251.78 | KJ676151 | KJ676188 | KJ676264 | – | – | – | Czachura and Janik (2025) |
| *Cosmospora clavi* | G.J.S. 84-290 | KJ676159 | KJ676196 | KJ676275 | – | – | – | Czachura and Janik (2025) |
| *Cosmospora coccinea* | A.R.2741 | HM484537 | AY489734 | HM484589 | MH936685 | – | – | Czachura and Janik (2025) |
| *Cosmospora coccinea* | A.R.2743 | KJ676141 | KJ676178 | KJ676252 | – | – | – | Czachura and Janik (2025) |
| *Cosmospora coccinea* | CBS 114050 | JN049831 | GQ505990 | DQ522501 | DQ522438 | – | – | Czachura and Janik (2025) |
| *Cosmospora coheniae* | BRIP 75895a T | PQ806952 | PQ792643 | PQ799345 | PQ799343 | – | – | Czachura and Janik (2025) |
| *Cosmospora cylindricospora* | KUNCC 22-12662 T | OP876700 | OP872570 | OQ025193 | OQ077584 | – | – | Czachura and Janik (2025) |
| *Cosmospora cymosa* | CBS 258.70 | MH859596 | MH871359 | – | – | – | – | Czachura and Janik (2025) |
| *Cosmospora cymosa* | CBS 762.69 T | NR_111605 | NG_058891 | KM232087 | – | – | – | Czachura and Janik (2025) |
| *Cosmospora elegans* | CBS 152410 T | PQ484119 | PQ484126 | PQ474173 | PQ474165 | – | – | Czachura and Janik (2025) |
| *Cosmospora fomiticola* | G.J.S.83-194 T | KJ676158 | KJ676195 | KJ676274 | – | – | – | Czachura and Janik (2025) |
| *Cosmospora fomiticola* | PDD 46398 | NR_165845 | NG_058874 | – | – | – | – | Czachura and Janik (2025) |
| *Cosmospora inonoticola* | HMAS 248723 T | KU563625 | – | KU563621 | – | – | – | Czachura and Janik (2025) |
| *Cosmospora inonoticola* | HMAS 271401 | NR_171931 | – | – | – | – | – | Czachura and Janik (2025) |
| *Cosmospora khandalensis* | A.R.4770 | KJ676143 | KJ676180 | KJ676256 | – | – | – | Czachura and Janik (2025) |
| *Cosmospora khandalensis* | IMI 112790 T | KJ676166 | KJ676203 | KJ676287 | HQ897723 | – | – | Czachura and Janik (2025) |
| *Cosmospora khandalensis* | MAFF 241500 | KJ676174 | KJ676211 | KJ676295 | – | – | – | Czachura and Janik (2025) |
| *Cosmospora khandalensis* | P.C. 1306 | KJ676177 | KJ676214 | KJ676298 | – | – | – | Czachura and Janik (2025) |
| *Cosmospora lavitskiae* | 7976 | KU563624 | – | KU563620 | – | – | – | Czachura and Janik (2025) |
| *Cosmospora lavitskiae* | CBS 530.68 | – | HQ231997 | – | MW833998 | – | – | Czachura and Janik (2025) |
| *Cosmospora lavitskiae* | lM 133984 T | KJ676167 | KJ676204 | KJ676288 | – | – | – | Czachura and Janik (2025) |
| *Cosmospora micropedis* | G.J.S. 10-113 | KJ676154 | KJ676191 | KJ676269 | – | – | – | Herrera et al. (2015) |
| *Cosmospora micropedis* | G.J.S. 86-108 ^T^ | KJ676161 | KJ676198 | KJ676277 | – | – | – | Herrera et al. (2015) |
| *Cosmospora micropedis* | G.J.S.10-121 | KJ676156 | KJ676193 | KJ676271 | – | – | – | Herrera et al. (2015) |
| *Cosmospora scruposae* | C.T.R. 71-62 | KJ676152 | KJ676189 | KJ676266 | – | – | – | Czachura and Janik (2025) |
| *Cosmospora scruposae* | G.J.S.86-278 | KJ676162 | KJ676199 | KJ676278 | – | – | – | Czachura and Janik (2025) |
| *Cosmospora scruposae* | G.J.S.86-320 | KJ676163 | KJ676200 | KJ676279 | – | – | – | Czachura and Janik (2025) |
| *Cosmospora stilbohypoxyli* | A.R. 4783 | KJ676144 | KJ676181 | KJ676257 | – | – | – | Czachura and Janik (2025) |
| *Cosmospora stilbohypoxyli* | CBS 137834 | NR_164533 | NG_058873 | – | – | – | – | Czachura and Janik (2025) |
| *Cosmospora ustulinae* | A.R.4215 | JN995619 | JN939841 | KJ676254 | JQ014129 | – | – | Czachura and Janik (2025) |
| *Cosmospora ustulinae* | G.J.S.92-95 | JN995633 | JN939818 | KJ676282 | JQ014137 | – | – | Czachura and Janik (2025) |
| *Cosmospora ustulinae* | MAFF 241532 | KJ676175 | KJ676212 | KJ676296 | – | – | – | Czachura and Janik (2025) |
| *Cosmospora viliuscula* | G.J.S. 09-411 | – | – | KC291905 | – | – | – | Czachura and Janik (2025) |
| *Cosmospora viliuscula* | G.J.S. 73-2 | – | JN939823 | KJ676272 | – | – | – | Czachura and Janik (2025) |
| *Cosmospora viliuscula* | IMI 73377a | NR_154791 | NG_060412 | KJ676292 | – | – | – | Czachura and Janik (2025) |
| *Cosmospora viridescens* | A.R. 2783 | KJ676142 | KJ676179 | KJ676253 | – | – | – | Czachura and Janik (2025) |
| *Cosmospora viridescens* | CBS 102430 | KJ676147 | KJ676184 | KJ676260 | – | – | – | Czachura and Janik (2025) |
| *Cosmospora viridescens* | CBS 102433 | KJ676148 | KJ676185 | KJ676261 | MW833999 | – | – | Czachura and Janik (2025) |
| *Cosmospora viridescens* | IMI 73377a T | KJ676171 | KJ676208 | KJ676292 | – | – | – | Czachura and Janik (2025) |
| *Cosmospora xylariae* | JF12119 | MZ955630 | – | – | – | – | – | Czachura and Janik (2025) |
| *Cosmospora xylariae* | JF14003 | MZ955631 | – | – | – | – | – | Czachura and Janik (2025) |
| *Daldinia andina* | CBS 114736 T | AM749918 | KY610430 | KC977259 | KY624239 | – | – | Wongkanoun et al. (2020) |
| *Daldinia bambusicola* | BCC27937 | MN153861 | MN153876 | – | MN172217 | – | – | Wongkanoun et al. (2020) |
| *Daldinia bambusicola* | BCC33678 | MN153860 | MN153877 | – | MN172218 | – | – | Wongkanoun et al. (2020) |
| *Daldinia bambusicola* | CBS 122872 T | KY610385 | KY610431 | AY951688 | KY624241 | – | – | Wongkanoun et al. (2020) |
| *Daldinia bambusicola* | SAUCC197001 | PP145311 | PP198902 | PP277065 | PP263619 | – | – | Wongkanoun et al. (2020) |
| *Daldinia bambusicola* | TBRC 8878 | MH922869 | MH922870 | MK165422 | MK165431 | – | – | Wongkanoun et al. (2020) |
| *Daldinia bambusicola* | TBRC 8879 | MH922872 | MH938543 | MK165423 | MK165432 | – | – | Wongkanoun et al. (2020) |
| *Daldinia brachysperma* | BCC33676 | MN153854 | MN153871 | MN172205 | – | – | – | Wongkanoun et al. (2020) |
| *Daldinia caldariorum* | CBS122874 | KU683756 | KU683756 | KU684128 | KU684289 | – | – | Wongkanoun et al. (2020) |
| *Daldinia caldariorum* | MUCL 49211 | AM749934 | KY610433 | KC977282 | KY624242 | – | – | Wongkanoun et al. (2020) |
| *Daldinia chiangdaoensis* | BCC88220 T | MN153850 | MN153867 | MN172197 | MN172208 | – | – | Wongkanoun et al. (2020) |
| *Daldinia chiangdaoensis* | BCC88221 | MN153851 | MN153868 | MN172198 | MN172209 | – | – | Wongkanoun et al. (2020) |
| *Daldinia childiae* | SAUCC133401 | PP145313 | PP198904 | PP277067 | PP263621 | – | – | Wongkanoun et al. (2020) |
| *Daldinia concentrica* | CBS 113277 | AY616683 | KY610434 | KC977274 | KY624243 | – | – | Wongkanoun et al. (2020) |
| *Daldinia dennisii* | CBS 114741 T | JX658477 | KY610435 | KC977262 | KY624244 | – | – | Wongkanoun et al. (2020) |
| *Daldinia ehretiae* | SAUCC228302 T | PP145319 | PP198888 | PP277051 | PP263613 | – | – | Wongkanoun et al. (2020) |
| *Daldinia ehretiae* | SAUCC228303 | PP145320 | PP198889 | PP277052 | PP263614 | – | – | Wongkanoun et al. (2020) |
| *Daldinia eschscholtzii* | BCC27887 | – | MN153878 | – | MN172214 | – | – | Wongkanoun et al. (2020) |
| *Daldinia eschscholtzii* | BCC28091 | MN153862 | MN153879 | – | MN172215 | – | – | Wongkanoun et al. (2020) |
| *Daldinia eschscholtzii* | BCC62428 | MN153863 | MN153880 | – | MN172216 | – | – | Wongkanoun et al. (2020) |
| ***Daldinia eschscholtzii*** | **GMBCC2475** | **PZ669404** | **PZ669417** | **PZ871004** | **PZ867825** | **–** | **–** | **This study** |
| *Daldinia eschscholtzii* | MUCL 45435 | JX658484 | KY610437 | KC977266 | KY624246 | – | – | Wongkanoun et al. (2020) |
| *Daldinia eschscholtzii* | SAUCC265301 | PP145315 | PP198906 | PP277069 | PP263623 | – | – | Wongkanoun et al. (2020) |
| *Daldinia eschscholtzii* | TBRC 8876 | MH938532 | MH938541 | MK165420 | MK165429 | – | – | Wongkanoun et al. (2020) |
| *Daldinia flavogranulata* | BCC89363 T | MN153856 | MN153873 | MN172200 | MN172211 | – | – | Wongkanoun et al. (2020) |
| *Daldinia flavogranulata* | BCC89365 | MN153857 | MN153874 | MN172201 | MN172212 | – | – | Wongkanoun et al. (2020) |
| *Daldinia flavogranulata* | BCC89376 | MN153858 | MN153875 | MN172202 | MN172213 | – | – | Wongkanoun et al. (2020) |
| *Daldinia guizhouensis* | GMB0719 T | PQ884703 | PQ885415 | PQ893600 | PQ893623 | – | – | Wongkanoun et al. (2020) |
| *Daldinia guizhouensis* | GMB5611 | PQ884704 | PQ885416 | PQ893601 | PQ893624 | – | – | Wongkanoun et al. (2020) |
| *Daldinia jianfengensis* | SAUCC373804 T | PP145325 | PP198890 | PP277053 | PP263615 | – | – | Wongkanoun et al. (2020) |
| *Daldinia jianfengensis* | SAUCC373805 | PP145326 | PP198891 | PP277054 | PP263616 | – | – | Wongkanoun et al. (2020) |
| *Daldinia korfii* | EBS 067 | KY204018 | – | KY204014 | – | – | – | Wongkanoun et al. (2020) |
| *Daldinia korfii* | EBS 473 | KY204020 | – | KY204016 | – | – | – | Wongkanoun et al. (2020) |
| *Daldinia kretzschmarioides* | TBRC 8875 | MH938531 | MH938540 | MK165416 | MK165425 | – | – | Wongkanoun et al. (2020) |
| *Daldinia ledongensis* | SAUCC393602 T | PP145327 | PP198892 | PP277055 | – | – | – | Wongkanoun et al. (2020) |
| *Daldinia ledongensis* | SAUCC393603 | PP145328 | PP198893 | PP277056 | – | – | – | Wongkanoun et al. (2020) |
| *Daldinia loculatoides* | CBS 113279 | AF176982 | KY610438 | KX271246 | KY624247 | – | – | Wongkanoun et al. (2020) |
| *Daldinia macaronesica* | CBS 113040 | KY610398 | KY610477 | KX271266 | KY624294 | – | – | Wongkanoun et al. (2020) |
| *Daldinia menghaiensis* | SAUCC242404 T | PP145323 | PP198894 | PP277057 | PP263617 | – | – | Wongkanoun et al. (2020) |
| *Daldinia menghaiensis* | SAUCC242405 | PP145324 | PP198895 | PP277058 | PP263618 | – | – | Wongkanoun et al. (2020) |
| *Daldinia petriniae* | MUCL 49214 | AM749937 | KY610439 | KC977261 | KY624248 | – | – | Wongkanoun et al. (2020) |
| *Daldinia phadaengensis* | BCC89349 T | MN153852 | MN153869 | MN172195 | MN172206 | – | – | Wongkanoun et al. (2020) |
| *Daldinia phadaengensis* | BCC89350 | MN153853 | MN153870 | MN172196 | MN172207 | – | – | Wongkanoun et al. (2020) |
| *Daldinia placentiformis* | MUCL 47603 | AM749921 | KY610440 | KC977278 | KY624249 | – | – | Wongkanoun et al. (2020) |
| *Daldinia pyrenaica* | MUCL 53969 | KY610413 | KY610413 | KY624312 | KY624274 | – | – | Wongkanoun et al. (2020) |
| *Daldinia rhododendri* | SAUCC460001 T | PP145330 | PP198896 | PP277059 | – | – | – | Wongkanoun et al. (2020) |
| *Daldinia rhododendri* | SAUCC460002 | PP145329 | PP198897 | PP277060 | – | – | – | Wongkanoun et al. (2020) |
| *Daldinia spatholobi* | SAUCC203501 T | PP145318 | PP198898 | PP277061 | – | – | – | Wongkanoun et al. (2020) |
| *Daldinia spatholobi* | SAUCC203502 | PP145317 | PP198899 | PP277062 | – | – | – | Wongkanoun et al. (2020) |
| *Daldinia steglichii* | MUCL 43512 | KY610399 | KY610479 | KX271269 | KY624250 | – | – | Wongkanoun et al. (2020) |
| *Daldinia subvernicosa* | TBRC 8877 T | MH938533 | MH938542 | MK165421 | MK165430 | – | – | Wongkanoun et al. (2020) |
| *Daldinia theissenii* | CBS 113044 | KY610388 | KY610441 | KX271247 | KY624251 | – | – | Wongkanoun et al. (2020) |
| *Daldinia thunbergiae* | SAUCC228601 T | PP145322 | PP198900 | PP277063 | – | – | – | Wongkanoun et al. (2020) |
| *Daldinia thunbergiae* | SAUCC228602 | PP145321 | PP198901 | PP277064 | – | – | – | Wongkanoun et al. (2020) |
| *Daldinia vernicosa* | CBS 119316 | KY610395 | KY610442 | KC977260 | KY624252 | – | – | Wongkanoun et al. (2020) |
| *Dichotomopilus dolichotrichus* | CBS 162.48 | NR_147624 | NG_059090 | JF772462 | KX976852 | – | – | Wang et al. (2022) |
| *Dichotomopilus erectus* | CBS 140.56 | NR_077201 | MH869086 | JF772458 | KX976854 | – | – | Wang et al. (2022) |
| *Dichotomopilus erectus* | CGMCC 3.12900 | KC109760 | KC109760 | KC109778 | KX976855 | – | – | Wang et al. (2022) |
| *Dichotomopilus finlandicus* | SZMC 26529 | MW541926 | – | MZ665529 | MZ665531 | – | – | Wang et al. (2022) |
| *Dichotomopilus funicola* | CBS 136.38 | MH855931 | MH867431 | JF772457 | KX976857 | – | – | Wang et al. (2022) |
| *Dichotomopilus funicola* | CBS 159.52 T | GU563369 | GU563354 | JF772461 | KX976856 | – | – | Wang et al. (2022) |
| *Dichotomopilus fusus* | CBS 114.83 | KX976661 | KX976755 | KX977003 | KX976860 | – | – | Wang et al. (2022) |
| *Dichotomopilus fusus* | CBS 372.66 | NR_147673 | NG_067492 | KX977002 | KX976859 | – | – | Wang et al. (2022) |
| *Dichotomopilus indicus* | CGMCC 3.14184 | GU563367 | GU563360 | JF772453 | KX976861 | – | – | Wang et al. (2022) |
| *Dichotomopilus pratensis* | CBS 804.83 | KX976665 | KX976759 | KX977007 | KX976867 | – | – | Wang et al. (2022) |
| *Dichotomopilus pratensis* | CBS 860.68 | KX976666 | KX976760 | KX977008 | KX976868 | – | – | Wang et al. (2022) |
| *Dichotomopilus pratensis* | HMAS 242921 | GU563372 | GU563357 | JF772450 | KX976866 | – | – | Wang et al. (2022) |
| *Dichotomopilus pseudoerectus* | CBS 252.75 | KX976667 | KX976761 | KX977009 | KX976869 | – | – | Wang et al. (2022) |
| *Dichotomopilus pseudofunicola* | DTO 318-I7 | KX976668 | KX976762 | KX977010 | KX976870 | – | – | Wang et al. (2022) |
| *Dichotomopilus ramosissimus* | CGMCC 3.14183 | GU563371 | GU563361 | JF772452 | KX976871 | – | – | Wang et al. (2022) |
| *Dichotomopilus reflexus* | CBS 157.49 | HM449051 | HM449055 | JF772460 | KX976873 | – | – | Wang et al. (2022) |
| *Dichotomopilus* sp. | SZMC 26527 | MW541925 | – | MZ665528 | MZ665530 | – | – | Wang et al. (2022) |
| *Dichotomopilus subfunicola* | CBS 812.73 | KX976670 | KX976764 | KX977012 | KX976877 | – | – | Wang et al. (2022) |
| *Dichotomopilus subfunicola* | CGMCC 3.12892 | JX867125 | JX867125 | JX867122 | KX976875 | – | – | Wang et al. (2022) |
| *Dichotomopilus variostiolatus* | CBS 179.84 | KX976672 | KX976766 | KX977014 | KX976879 | – | – | Wang et al. (2022) |
| *Dichotomopilus variostiolatus* | DTO 319-B9 | KX976674 | KX976768 | KX977016 | KX976881 | – | – | Wang et al. (2022) |
| *Floropilus chiversii* | CBS 558.80 T | MK926818 | MK926818 | MK926918 | MK876778 | – | – | Wang et al. (2022) |
| *Gamsia aggregata* | CBS 251.69 T | LM652378 | LM652500 | – | – | – | – | Sandoval-Denis et al. (2016) |
| *Gamsia simplex* | CBS 546.69 T | LM652379 | LM652501 | – | – | – | – | Sandoval-Denis et al. (2016) |
| *Graphostroma platystomum* | CBS 270.87 | JX658535 | DQ836906 | HG934108 | KY624296 | – | – | Wongkanoun et al. (2020) |
| *Humicola ampulliella* | CBS 116735 | LT993568 | LT993568 | LT993649 | LT993487 | – | – | Wang et al. (2022) |
| *Humicola christenseniae* | CBS 127760 | LT993571 | LT993571 | LT993652 | LT993490 | – | – | Wang et al. (2022) |
| *Humicola cuyabenoensis* | CBS 398.97 | LT993573 | LT993573 | LT993654 | LT993492 | – | – | Wang et al. (2022) |
| *Humicola degenerans* | CBS 232.65 | LT993574 | LT993574 | LT993655 | LT993493 | – | – | Wang et al. (2022) |
| *Humicola distorta* | CBS 417.66 | LT993577 | LT993577 | LT993658 | LT993496 | – | – | Wang et al. (2022) |
| *Humicola floriformis* | CBS 815.97 | LT993578 | LT993578 | LT993659 | LT993497 | – | – | Wang et al. (2022) |
| *Humicola fuscoatra* | CBS 118.14 | LT993579 | LT993579 | LT993660 | LT993498 | – | – | Wang et al. (2022) |
| *Humicola fuscogrisea* | CGMCC:3.13790 | LT993581 | LT993581 | LT993662 | LT993500 | – | – | Wang et al. (2022) |
| *Humicola hirsuta* | CBS 144492 | MZ334726 | MZ351425 | MZ343013 | MZ342974 | – | – | Wang et al. (2022) |
| *Humicola homopilata* | CBS 157.55 | LT993582 | LT993582 | LT993663 | LT993501 | – | – | Wang et al. (2022) |
| *Humicola leptodermospora* | CBS 120095 | LT993584 | LT993584 | LT993665 | LT993503 | – | – | Wang et al. (2022) |
| *Humicola malaysiensis* | CBS 399.97 | LT993586 | LT993586 | LT993667 | LT993505 | – | – | Wang et al. (2022) |
| *Humicola mutabilis* | CBS 779.71 | LT993588 | LT993588 | LT993669 | LT993507 | – | – | Wang et al. (2022) |
| *Humicola olivacea* | CBS 142031 | LT993589 | LT993589 | LT993670 | LT993508 | – | – | Wang et al. (2022) |
| *Humicola pinnata* | CBS 467.66 | LT993590 | LT993590 | LT993671 | LT993509 | – | – | Wang et al. (2022) |
| *Humicola quadrangulata* | CBS 111771 | LT993593 | LT993593 | LT993674 | LT993512 | – | – | Wang et al. (2022) |
| *Humicola seminuda* | CBS 368.84 | LT993594 | LT993594 | LT993675 | LT993513 | – | – | Wang et al. (2022) |
| *Humicola sphaeralis* | CBS 985.87 | LT993598 | LT993598 | LT993679 | LT993517 | – | – | Wang et al. (2022) |
| *Humicola udagawae* | CBS 337.68 | LT993601 | LT993601 | LT993682 | LT993520 | – | – | Wang et al. (2022) |
| *Humicola wallefii* | CBS 147.67 | LT993602 | LT993602 | LT993683 | LT993521 | – | – | Wang et al. (2022) |
| *Hyalosphaerella fragilis* | CBS 456.73 T | KX976693 | KX976791 | KX977042 | MK876779 | – | – | Wang et al. (2022) |
| *Hypomontagnella monticulosa* | BCC58592 | MN153864 | MN153881 | MN172204 | MN172219 | – | – | Wongkanoun et al. (2020) |
| *Hypomontagnella monticulosa* | BCC69203 | MN153865 | MN153882 | MN172203 | MN172220 | – | – | Wongkanoun et al. (2020) |
| *Hypomontagnella monticulosa* | MUCL 54604 | KY610404 | KY610487 | KX271273 | KY624305 | – | – | Wongkanoun et al. (2020) |
| *Hypomontagnella submonticulosa* | CBS 115280 | KC968923 | KY610457 | KC977267 | KY624226 | – | – | Wongkanoun et al. (2020) |
| *Hypomyces dactylarioides* | CBS 14178 T | NR_111430 | – | – | FN868683 | HF911722 |  | Liu et al. (2025) |
| *Hypomyces lactifluorum* | TAAM 170476 | FN859432 | – | – | EU710773 | FN868751 |  | Liu et al. (2025) |
| *Hypomyces odoratus* | GAm 329 | FN859434 | – | – | FN868688 | FN868753 | – | Liu et al. (2025) |
| *Hypomyces rosellus* | TFC 201071 | FN859443 | – | – | FN868697 | HF911779 | – | Liu et al. (2025) |
| *Hypomyces samuelsii* | TFC 2007-23 | FN859451 | – | – | FN868705 | FN868769 | – | Liu et al. (2025) |
| *Hypoxylon crocopeplum* | CBS 119004 | KC968907 | KY610445 | KC977268 | KY624255 | – | – | Wongkanoun et al. (2020) |
| *Hypoxylon fragiforme* | MUCL 51264 | KC477229 | KM186295 | KX271282 | KM186296 | – | – | Wongkanoun et al. (2020) |
| *Hypoxylon fuscum* | CBS 113049 | KY610401 | KY610482 | KX271271 | KY624299 | – | – | Wongkanoun et al. (2020) |
| *Hypoxylon haematostroma* | BCC50533 | MN153866 | MN153883 | – | MN172221 | – | – | Wongkanoun et al. (2020) |
| *Hypoxylon haematostroma* | MUCL 53301 | KC968911 | KY610484 | KC977291 | KY624301 | – | – | Wongkanoun et al. (2020) |
| *Hypoxylon investiens* | CBS 118183 | KC968925 | KY610450 | KC977270 | KY624259 | – | – | Wongkanoun et al. (2020) |
| *Hypoxylon lateripigmentum* | MUCL 53304 | KC968933 | KY610486 | KC977290 | KY624304 | – | – | Wongkanoun et al. (2020) |
| *Hypoxylon lenormandii* | CBS 119003 | KC968943 | KY610452 | KC977273 | KY624261 | – | – | Wongkanoun et al. (2020) |
| *Hypoxylon petriniae* | CBS 114746 | KY610405 | KY610491 | KX271274 | KY624279 | – | – | Wongkanoun et al. (2020) |
| *Hypoxylon rickii* | MUCL 53309 | KC968932 | KY610416 | KC977288 | KY624281 | – | – | Wongkanoun et al. (2020) |
| *Hypoxylon rubiginosum* | MUCL 52887 | KC477232 | KY610469 | KY624311 | KY624266 | – | – | Wongkanoun et al. (2020) |
| *Hypoxylon samuelsii* | MUCL 51843 | KC968916 | KY610466 | KC977286 | KY624269 | – | – | Wongkanoun et al. (2020) |
| *Jackrogersella cohaerens* | CBS 119126 | KY610396 | KY610497 | KY624314 | KY624270 | – | – | Wongkanoun et al. (2020) |
| *Jackrogersella minutella* | CBS 119015 | KY610381 | KY610424 | KX271240 | KY624235 | – | – | Wongkanoun et al. (2020) |
| *Jackrogersella multiformis* | CBS 119016 | KC477234 | KY610473 | KX271262 | KY624290 | – | – | Wongkanoun et al. (2020) |
| *Madurella fahalii* | CBS 129176 T | MK926819 | MK926819 | MK926919 | MK876780 | – | – | Wang et al. (2022) |
| *Madurella mycetomatis* | CBS 109801 T | MK926820 | MK926820 | MK926920 | MK876781 | – | – | Wang et al. (2022) |
| *Madurella pseudomycetomatis* | CBS 129177 T | MK926821 | MK926821 | MK926921 | MK876782 | – | – | Wang et al. (2022) |
| *Madurella tropicana* | CBS 201.38 T | MK926824 | MK926824 | MK926924 | MK876785 | – | – | Wang et al. (2022) |
| *Melanocarpus albomyces* | CBS 638.94 T | KX976679 | KX976773 | KX977021 | KX976886 | – | – | Wang et al. (2022) |
| *Melanocarpus albomyces* | CBS 747.70 | KX976680 | KX976774 | KX977022 | KX976887 | – | – | Wang et al. (2022) |
| *Microascus alveolaris* | CBS 139501 T | LM652385 | HG380484 | LM652601 | – | HG380407 | – | Sandoval-Denis et al. (2016) |
| *Microascus alveolaris* | UTHSC 05-1041 | LM652382 | HG380488 | LM652598 | – | HG380411 | – | Sandoval-Denis et al. (2016) |
| *Microascus alveolaris* | UTHSC 05-3416 | LM652381 | HG380483 | LM652597 | – | HG380406 | – | Sandoval-Denis et al. (2016) |
| *Microascus alveolaris* | UTHSC 06-3152 | LM652383 | HG380487 | LM652599 | – | HG380410 | – | Sandoval-Denis et al. (2016) |
| *Microascus alveolaris* | UTHSC 07-1823 | LM652384 | HG380489 | LM652600 | – | HG380412 | – | Sandoval-Denis et al. (2016) |
| *Microascus alveolaris* | UTHSC 08-886 | LM652386 | HG380485 | LM652602 | – | HG380408 | – | Sandoval-Denis et al. (2016) |
| *Microascus alveolaris* | UTHSC 10-214 | LM652387 | HG380486 | LM652603 | – | HG380409 | – | Sandoval-Denis et al. (2016) |
| *Microascus alveolaris* | UTHSC R-4634 | LM652388 | HG380490 | LM652604 | – | HG380413 | – | Sandoval-Denis et al. (2016) |
| *Microascus brunneosporus* | CBS 138276 T | LM652390 | HG380497 | LM652605 | – | HG380420 | – | Sandoval-Denis et al. (2016) |
| *Microascus campaniformis* | CBS 138126 T | LM652391 | HG380495 | LM652606 | – | HG380418 | – | Sandoval-Denis et al. (2016) |
| *Microascus chartarus* | CBS 294.52 T | LM652393 | HG380463 | LM652607 | – | HG380386 | – | Sandoval-Denis et al. (2016) |
| *Microascus cinereus* | UTHSC 06-3278 | LM652394 | HG380424 | LM652608 | – | HG380347 | – | Sandoval-Denis et al. (2016) |
| *Microascus cinereus* | UTHSC 08-3181 | LM652395 | HG380425 | LM652609 | – | HG380348 | – | Sandoval-Denis et al. (2016) |
| *Microascus cinereus* | UTHSC 09-573 | LM652396 | HG380426 | LM652610 | – | HG380349 | – | Sandoval-Denis et al. (2016) |
| *Microascus cinereus* | UTHSC 10-2805 T | LM652397 | HG380427 | LM652611 | – | HG380350 | – | Sandoval-Denis et al. (2016) |
| *Microascus cinereus* | UTHSC 11-383 T | LM652398 | HG380428 | LM652612 | – | HG380351 | – | Sandoval-Denis et al. (2016) |
| *Microascus cirrosus* | CBS 217.31 T | LM652400 | HG380429 | LM652614 | – | HG380352 | – | Sandoval-Denis et al. (2016) |
| *Microascus cirrosus* | CBS 277.34 | LM652401 | LM652504 | LM652615 | – | LM652556 | – | Sandoval-Denis et al. (2016) |
| *Microascus cirrosus* | FMR 4004 | LM652406 | LM652507 | LM652620 | – | LM652559 | – | Sandoval-Denis et al. (2016) |
| *Microascus cirrosus* | UTHSC 07-1887 | LM652403 | HG380431 | LM652617 | – | HG380354 | – | Sandoval-Denis et al. (2016) |
| *Microascus cirrosus* | UTHSC 11-14 | LM652404 | HG380432 | LM652618 | – | HG380355 | – | Sandoval-Denis et al. (2016) |
| *Microascus croci* | CBS 158.44 | LM652407 | LM652508 | LM652621 | – | LM652560 | – | Sandoval-Denis et al. (2016) |
| *Microascus croci* | FMR 3997 | LM652405 | LM652506 | LM652619 | – | LM652558 | – | Sandoval-Denis et al. (2016) |
| *Microascus desmosporus* | CBS 301.61 | LM652402 | LM652505 | LM652616 | – | LM652557 | – | Sandoval-Denis et al. (2016) |
| *Microascus expansus* | UTHSC 06-2516 | LM652409 | HG380491 | LM652623 | – | HG380414 | – | Sandoval-Denis et al. (2016) |
| *Microascus gracilis* | CBS 195.61 | LM652416 | HG380468 | LM652629 | – | HG380391 | – | Sandoval-Denis et al. (2016) |
| *Microascus gracilis* | CBS 300.61 | LM652417 | LM652512 | LM652630 | – | LM652563 | – | Sandoval-Denis et al. (2016) |
| *Microascus gracilis* | CBS 369.70 T | LM652412 | HG380467 | LM652625 | – | HG380390 | – | Sandoval-Denis et al. (2016) |
| *Microascus griseus* | CBS 365.65 T | LM652399 | HG380423 | LM652613 | – | HG380346 | – | Sandoval-Denis et al. (2016) |
| *Microascus hyalinus* | CBS 766.70 T | LM652418 | LM652513 | LM652631 | – | LM652564 | – | Sandoval-Denis et al. (2016) |
| *Microascus intricatus* | CBS 138128 T | LM652419 | HG380496 | LM652632 | – | HG380419 | – | Sandoval-Denis et al. (2016) |
| *Microascus intricatus* | FMR 12362 | LM652420 | LM652514 | LM652633 | – | LM652565 | – | Sandoval-Denis et al. (2016) |
| *Microascus longirostris* | CBS 196.61 T | LM652421 | LM652515 | LM652634 | – | LM652566 | – | Sandoval-Denis et al. (2016) |
| *Microascus longirostris* | CBS 415.64 | LM652422 | LM652516 | LM652635 | – | LM652567 | – | Sandoval-Denis et al. (2016) |
| *Microascus macrosporus* | CBS 662.71 | LM652423 | LM652517 | LM652636 | – | LM652568 | – | Sandoval-Denis et al. (2016) |
| *Microascus murinus* | CBS 830.70 T | LM652424 | HG380481 | LM652637 | – | HG380404 | – | Sandoval-Denis et al. (2016) |
| *Microascus paisii* | CBS 213.27 T | LM652434 | LM652518 | LM652647 | – | LM652569 | – | Sandoval-Denis et al. (2016) |
| *Microascus paisii* | CBS 897.68 | LM652436 | LM652520 | LM652649 | – | LM652571 | – | Sandoval-Denis et al. (2016) |
| *Microascus paisii* | UTHSC 08-1734 | LM652426 | HG380452 | LM652639 | – | HG380375 | – | Sandoval-Denis et al. (2016) |
| *Microascus paisii* | UTHSC 09-457 | LM652428 | HG380454 | LM652641 | – | HG380377 | – | Sandoval-Denis et al. (2016) |
| *Microascus paisii* | UTHSC 11-708 | LM652431 | HG380457 | LM652644 | – | HG380380 | – | Sandoval-Denis et al. (2016) |
| *Microascus pyramidus* | CBS 212.65 T | LM652439 | HG380435 | LM652652 | – | HG380358 | – | Sandoval-Denis et al. (2016) |
| *Microascus restrictus* | CBS 138277 T | LM652440 | HG380494 | LM652653 | – | HG380417 | – | Sandoval-Denis et al. (2016) |
| *Microascus senegalensis* | CBS 277.74 T | LM652441 | LM652523 | LM652654 | – | LM652574 | – | Sandoval-Denis et al. (2016) |
| *Microascus trigonosporus* | CBS 139501 T | KX923829 | KX924029 | KX924263 | – | – | – | Sandoval-Denis et al. (2016) |
| *Microascus trigonosporus* | CBS 199.61T | LM652444 | HG380438 | LM652656 | – | HG380361 | – | Sandoval-Denis et al. (2016) |
| *Microascus trigonosporus* | CBS 218.31 T | LM652443 | HG380436 | LM652655 | – | HG380359 | – | Sandoval-Denis et al. (2016) |
| *Microascus trigonosporus* | CBS 262.35T | LM652445 | LM652525 | LM652657 | – | LM652575 | – | Sandoval-Denis et al. (2016) |
| *Microascus verrucosus* | CBS 138278 T | LM652446 | HG380493 | LM652658 | – | HG380416 | – | Sandoval-Denis et al. (2016) |
| *Microthielavia ovispora* | CBS 165.75 T | MK926826 | MK926826 | MK926926 | MK876787 | – | – | Wang et al. (2022) |
| *Mucor abortisporangium* | CGMCC 3.16133 T | OL678180 | – | – | – | – | – | Zhao et al. (2023a) |
| *Mucor abundans* | CBS 388.35 T | JN206111 | NG_063979 | – | – | – | – | Gajanayake et al. (2023) |
| *Mucor abundans* | CBS 521.66 | JN206110 | JN206457 | – | – | – | – | Gajanayake et al. (2023) |
| *Mucor aligarensis* | CBS 993.70 T | NR_103634 | NG_057920 | – | – | – | – | Gajanayake et al. (2023) |
| *Mucor aligarensis* | NNIBRFG6255 | MN267431 | – | – | – | – | – | Gajanayake et al. (2023) |
| *Mucor amethystinus* | [CBS 526.68](https://www.ncbi.nlm.nih.gov/nuccore/JN206015.1) | JN206015 | JN206426 | – | – | – | – | Gajanayake et al. (2023) |
| *Mucor amethystinus* | CBS 846.73 T | JN206014 | – | – | – | – | – | Gajanayake et al. (2023) |
| *Mucor amphibiorum* | CBS 763.74 T | NR_103615 | NG_057877 | – | – | – | – | Gajanayake et al. (2023) |
| *Mucor amphisporus* | CGMCC 3.16134 T | OL678181 | – | – | – | – | – | Zhao et al. (2023a) |
| *Mucor amphisporus* | XY07649 | OL678182 | – | – | – | – | – | Gajanayake et al. (2023) |
| *Mucor ardhlaengiktus* | ATCC-MYA-4767 | NR_111683 | NG_042602 | – | – | – | – | Gajanayake et al. (2023) |
| *Mucor ardhlaengiktus* | CBS 210.80 T | NR_152960 | NG_069778 | – | – | – | – | Gajanayake et al. (2023) |
| *Mucor ardhlaengiktus* | IHEM:25184 | OW987391 | – | – | – | – | – | Gajanayake et al. (2023) |
| *Mucor ardhlaengiktus* | URM8592 | OQ034611 | – | – | – | – | – | Gajanayake et al. (2023) |
| *Mucor ascheae* | BRIP 72394b T | NR_187100 | – | – | – | – | – | Gajanayake et al. (2023) |
| *Mucor ascheae* | BRIP 75043a | OR096133 | – | – | – | – | – | Gajanayake et al. (2023) |
| *Mucor aseptatophorus* | FPFCC58ER-3 | OR400652 | – | – | – | – | – | Gajanayake et al. (2023) |
| *Mucor aseptatophorus* | MFLUCC 21-0040 T | MZ433252 | MZ433249 | – | – | – | – | Gajanayake et al. (2023) |
| *Mucor atramentarius* | [CBS 202.28 T](https://www.ncbi.nlm.nih.gov/nuccore/JN205994.1) | MH854979 | JN206418 | – | – | – | – | Gajanayake et al. (2023) |
| *Mucor azygosporus* | CBS 292.63 T | NR_103639 | NG_057928 | – | – | – | – | Gajanayake et al. (2023) |
| *Mucor bacilliformis* | CBS 251.53 T | NR_145285 | NG_057916 | – | – | – | – | Gajanayake et al. (2023) |
| *Mucor bainier* | CBS 293.63 | MH858288 | MH869896 | – | – | – | – | Gajanayake et al. (2023) |
| *Mucor bainieri* | CBS 293.63 T | NR_103628 | JN206424 | – | – | – | – | Gajanayake et al. (2023) |
| *Mucor breviphorus* | CGMCC 3.16135 T | OL678183 | – | – | – | – | – | Zhao et al. (2023a) |
| *Mucor brunneogriseus* | [CBS 129.41](https://www.ncbi.nlm.nih.gov/nuccore/MH856086.1) | MH856086 | MT523848 | – | – | – | – | Gajanayake et al. (2023) |
| *Mucor brunneolus* | CGMCC 3.16136 T | OL678184 | – | – | – | – | – | Zhao et al. (2023a) |
| *Mucor caatingaensis* | URM 7223 T | KT960377 | KT960371 | – | – | – | – | Gajanayake et al. (2023) |
| *Mucor cavernicola* | GMBCC1073 T | PQ654019 | PQ654363 | – | – | – | – | Liu (2025) |
| *Mucor cavernicola* | GMBCC1074 | PQ654020 | PQ654364 | – | – | – | – | Liu (2025) |
| *Mucor changshaensis* | CGMCC 3.16137 T | OL678185 | – | – | – | – | – | Zhao et al. (2023a) |
| *Mucor changshaensis* | XY07965 | OL678186 | – | – | – | – | – | Gajanayake et al. (2023) |
| *Mucor chiangraiensis* | MFLUCC 21-0042 T | MZ433253 | MZ433250 | – | – | – | – | Gajanayake et al. (2023) |
| *Mucor chlamydosporus* | CGMCC 3.16138 T | OL678187 | – | – | – | – | – | Zhao et al. (2023a) |
| *Mucor chlamydosporus* | XY08211 | OL678189 | – | – | – | – | – | Gajanayake et al. (2023) |
| *Mucor chlamydosporus* | XY08225 | OL678188 | – | – | – | – | – | Gajanayake et al. (2023) |
| *Mucor chuxiongensis* | CBS 14370 T | MG255732 | MG255711 | – | – | – | – | Gajanayake et al. (2023) |
| *Mucor circinatus* | URM 7218 | KY008576 | KY008571 | – | – | – | – | Gajanayake et al. (2023) |
| *Mucor circinelloides* | CBS 195.68 T | JN205961 | NG_055735 | – | – | – | – | Gajanayake et al. (2023) |
| *Mucor circinelloides* | CBS 239.35 | JN205942 | MH867177 | – | – | – | – | Gajanayake et al. (2023) |
| *Mucor circinelloides* | CBS:106.10 | MH854593 | MH866121 | – | – | – | – | Gajanayake et al. (2023) |
| *Mucor circinelloides* | CBS:252.53 | MH857182 | MH868721 | – | – | – | – | Gajanayake et al. (2023) |
| *Mucor circinelloides* | Lianmao | OQ359158 | OQ359157 | – | – | – | – | Gajanayake et al. (2023) |
| *Mucor circinelloides* | LZ27 | MN533740 | – | – | – | – | – | Gajanayake et al. (2023) |
| *Mucor circinelloides* | SD-8 | KY807767 | – | – | – | – | – | Gajanayake et al. (2023) |
| *Mucor circinelloides* | VKM:F 1111 | OR575480 | OR575480 | – | – | – | – | Gajanayake et al. (2023) |
| *Mucor ctenidius* | CBS 293.66 T | MH858796 | MH870436 | – | – | – | – | Gajanayake et al. (2023) |
| *Mucor ctenidius* | CBS 696.76 | JN205977 | – | – | – | – | – | Gajanayake et al. (2023) |
| *Mucor donglingensis* | CGMCC 3.16139 T | OL678190 | – | – | – | – | – | Zhao et al. (2023a) |
| *Mucor donglingensis* | XY08501 | OL678191 | – | – | – | – | – | Gajanayake et al. (2023) |
| *Mucor durus* | CBS 156.51 T | NR_145295 | NG_057918 | – | – | – | – | Gajanayake et al. (2023) |
| *Mucor ellipsoideus* | ATCC MYA-4767 | NR_111683 | NG_042602 | – | – | – | – | Gajanayake et al. (2023) |
| *Mucor ellipsoideus* | CBS 126271 T | MH863952 | MH875411 | – | – | – | – | Gajanayake et al. (2023) |
| *Mucor ellipsoideus* | HP524 | – | KX980510 | – | – | – | – | Gajanayake et al. (2023) |
| *Mucor ellipsoideus* | MuelHB24N03 | OM372740 | – | – | – | – | – | Gajanayake et al. (2023) |
| *Mucor endophyticus* | CBS 385.95 T | NR_111661 | NG_057970 | – | – | – | – | Gajanayake et al. (2023) |
| *Mucor exponens* | CBS 404.58 | JN206208 | MH869358 | – | – | – | – | Gajanayake et al. (2023) |
| *Mucor exponens* | CBS 141.20 | JN206206 | MH866204 | – | – | – | – | Gajanayake et al. (2023) |
| *Mucor falcatus* | CBS 251.35 T | MH855668 | MH867181 | – | – | – | – | Gajanayake et al. (2023) |
| *Mucor flavus* | CBS 230.35 T | JN206061 | JN206464 | – | – | – | – | Gajanayake et al. (2023) |
| *Mucor flavus* | CBS 234.35 | JN206051 | JN206468 | – | – | – | – | Gajanayake et al. (2023) |
| *Mucor flavus* | CBS 893.73 | JN206062 | JN206465 | – | – | – | – | Gajanayake et al. (2023) |
| *Mucor floccosus* | CGMCC 3.16140 T | OL678192 | – | – | – | – | – | Zhao et al. (2023a) |
| *Mucor floccosus* | XY07967 | OL678193 | – | – | – | – | – | Gajanayake et al. (2023) |
| *Mucor fluvii* | CNUFC-MSW21-1 T | MF667992 | MF667995 | – | – | – | – | Gajanayake et al. (2023) |
| *Mucor fluvii* | XY07447 | OL620129 | – | – | – | – | – | Gajanayake et al. (2023) |
| *Mucor fragilis* | CBS 236.35 | FN650655 | FN650671 | – | – | – | – | Gajanayake et al. (2023) |
| *Mucor fuscus* | CBS 282.78 | JN206201 | JN206442 | – | – | – | – | Gajanayake et al. (2023) |
| *Mucor fuscus* | CBS 313.78 | JN206200 | MH872901 | – | – | – | – | Gajanayake et al. (2023) |
| *Mucor fusiformisporus* | CGMCC 3.16141 T | OL678194 | – | – | – | – | – | Zhao et al. (2023a) |
| *Mucor fusiformisporus* | XY08117 | OL678195 | – | – | – | – | – | Gajanayake et al. (2023) |
| *Mucor fusiformisporus* | XY08153 | OL678196 | – | – | – | – | – | Gajanayake et al. (2023) |
| *Mucor fusiformisporus* | XY08154 | OL678197 | – | – | – | – | – | Gajanayake et al. (2023) |
| *Mucor genevensis* | CBS 114.08 T | NR_103632 | NG_057971 | – | – | – | – | Gajanayake et al. (2023) |
| *Mucor genevensis* | CBS 535.78 | JN206045 | JN206436 | – | – | – | – | Gajanayake et al. (2023) |
| *Mucor germinans* | SM1517 T | OQ709230 | – | – | – | – | – | Gajanayake et al. (2023) |
| *Mucor gigasporus* | CBS 566.91 T | NR_103646 | NG_057926 | – | – | – | – | Gajanayake et al. (2023) |
| *Mucor griseocyanus* | CBS 116.08 T | NR_126136 | NG_056283 | – | – | – | – | Gajanayake et al. (2023) |
| *Mucor griseocyanus* | CBS 199.28 | MH854976 | – | – | – | – | – | Gajanayake et al. (2023) |
| *Mucor griseocyanus* | CBS:223.56 | MH857592 | MH869141 | – | – | – | – | Gajanayake et al. (2023) |
| *Mucor griseocyanus* | XY06685 | OL620130 | – | – | – | – | – | Gajanayake et al. (2023) |
| *Mucor griseocyanus* | XY06697 | OL620131 | – | – | – | – | – | Gajanayake et al. (2023) |
| *Mucor grylli* | CNUFC-CY102 | OM868231 | OM843127 | – | – | – | – | Gajanayake et al. (2023) |
| *Mucor guilliermondii* | CBS 174.27 | MH854920 | MH866413 | – | – | – | – | Gajanayake et al. (2023) |
| *Mucor heilongjiangensis* | CGMCC 3.16142 T | OL678198 | – | – | – | – | – | Zhao et al. (2023a) |
| *Mucor heilongjiangensis* | XY05057 | OL678199 | – | – | – | – | – | Gajanayake et al. (2023) |
| *Mucor hemisphaericum* | CGMCC 3.16143 T | OL678200 | – | – | – | – | – | Zhao et al. (2023a) |
| *Mucor heterogamus* | CBS 338.74 | JN206169 | JN206488 | – | – | – | – | Gajanayake et al. (2023) |
| *Mucor hiemalis* | CBS 201.65 T | JN206125 | HM849683 | – | – | – | – | Gajanayake et al. (2023) |
| *Mucor hiemalis* | HP521 | – | KY117233 | – | – | – | – | Gajanayake et al. (2023) |
| *Mucor hiemalis* | INSF1.6 | – | OR053888 | – | – | – | – | Gajanayake et al. (2023) |
| *Mucor homothallicus* | CGMCC 3.16144 T | OL678201 | – | – | – | – | – | Zhao et al. (2023a) |
| *Mucor homothallicus* | XY06967 | OL678202 | – | – | – | – | – | Gajanayake et al. (2023) |
| *Mucor hyalinosporus* | CGMCC 3.16145 T | OL678203 | – | – | – | – | – | Zhao et al. (2023a) |
| *Mucor hyangburmii* | CNUFC-CY22 | OM868233 | NG_149044 | – | – | – | – | Gajanayake et al. (2023) |
| *Mucor inaequisporus* | CBS 255.36 T | JN206177 | NG_057929 | – | – | – | – | Gajanayake et al. (2023) |
| *Mucor inaequisporus* | CBS 351.50 | JN206178 | MH868169 | – | – | – | – | Gajanayake et al. (2023) |
| *Mucor indicus* | CBS 226.29 T | MH855050 | NG_057878 | – | – | – | – | Gajanayake et al. (2023) |
| *Mucor irregularis* | BMU06287 | MG583964 | – | – | – | – | – | Gajanayake et al. (2023) |
| *Mucor irregularis* | CBS 103.93 T | HM623314 | HM849684 | – | – | – | – | Gajanayake et al. (2023) |
| *Mucor irregularis* | CMFCCC B 50p | JX976251 | JX976206 | – | – | – | – | Gajanayake et al. (2023) |
| *Mucor irregularis* | GM204 | OM956174 | OM956176 | – | – | – | – | Gajanayake et al. (2023) |
| *Mucor irregularis* | SRF1 | MZ710167 | – | – | – | – | – | Gajanayake et al. (2023) |
| *Mucor janssenii* | CBS 185.68 | JN206006 | MH870818 | – | – | – | – | Gajanayake et al. (2023) |
| *Mucor janssenii* | CBS 365.70 | JN206009 | MH871465 | – | – | – | – | Gajanayake et al. (2023) |
| *Mucor japonicus* | CBS 154.69 T | JN206158 | JN206446 | – | – | – | – | Gajanayake et al. (2023) |
| *Mucor koreanus* | EML-QT1 T | KT936259 | NG_068529 | – | – | – | – | Gajanayake et al. (2023) |
| *Mucor koreanus* | EML-QT2 | KT936260 | KT936254 | – | – | – | – | Gajanayake et al. (2023) |
| *Mucor kunryangriensis* | CNUFC-CY223 T | OM868236 | OM843131 | – | – | – | – | Gajanayake et al. (2023) |
| *Mucor lanceolatus* | CBS 638.74 | JN206205 | JN206443 | – | – | – | – | Gajanayake et al. (2023) |
| *Mucor laxorrhizus* | CBS 143.85 T | JN206209 | MH873554 | – | – | – | – | Gajanayake et al. (2023) |
| *Mucor laxorrhizus* | CBS 237.66 | JN206210 | MH870423 | – | – | – | – | Gajanayake et al. (2023) |
| *Mucor lilianae* | KM 196153 | NR_152978 | KT736106 | – | – | – | – | Gajanayake et al. (2023) |
| *Mucor lobatus* | CGMCC 3.16146 T | OL678204 | – | – | – | – | – | Zhao et al. (2023a) |
| *Mucor lobatus* | XY07029 | OL678205 | – | – | – | – | – | Gajanayake et al. (2023) |
| *Mucor lusitanicus* | CBS 108.17 | MH854658 | MH866177 | – | – | – | – | Gajanayake et al. (2023) |
| *Mucor lusitanicus* | CBS 276.49 | JN205984 | MT523850 | – | – | – | – | Gajanayake et al. (2023) |
| *Mucor lusitanicus* | CBS:253.35 | MH855669 | MH867183 | – | – | – | – | Gajanayake et al. (2023) |
| *Mucor lusitanicus* | CBS:277.49 | MH856522 | MH868052 | – | – | – | – | Gajanayake et al. (2023) |
| *Mucor lusitanicus* | VKM:F-1238 | OR581593 | OR581593 | – | – | – | – | Gajanayake et al. (2023) |
| *Mucor luteus* | CBS 243.35 T | JX976254 | NG_057969 | – | – | – | – | Gajanayake et al. (2023) |
| *Mucor megalocarpus* | CBS 215.27 T | NR_145286 | NG_057925 | – | – | – | – | Gajanayake et al. (2023) |
| *Mucor meguroensis* | ATCC 200999 | MT523869 | MT523844 | – | – | – | – | Gajanayake et al. (2023) |
| *Mucor merdicola* | URM 7222 T | KT960374 | KT960372 | – | – | – | – | Gajanayake et al. (2023) |
| *Mucor merdophylus* | URM 7908 T | MK775467 | MK775466 | – | – | – | – | Gajanayake et al. (2023) |
| *Mucor minutus* | CBS 586.67 T | NR_152958 | JN206463 | – | – | – | – | Gajanayake et al. (2023) |
| *Mucor minutus* | XY07460 | OL620138 | – | – | – | – | – | Gajanayake et al. (2023) |
| *Mucor moelleri* | CBS 406.58 T | NR_111659 | MH869359 | – | – | – | – | Gajanayake et al. (2023) |
| *Mucor moniliformis* | CGMCC 3.16147 T | OL678206 | – | – | – | – | – | Zhao et al. (2023a) |
| *Mucor moniliformis* | XY07458 | OL678207 | – | – | – | – | – | Gajanayake et al. (2023) |
| *Mucor mousanensis* | CBS 999.70 T | NR_103629 | NG_057912 | – | – | – | – | Gajanayake et al. (2023) |
| *Mucor mucedo* | CBS 640.67 T | NR_103688 | NG_057876 | – | – | – | – | Gajanayake et al. (2023) |
| *Mucor mucedo* | CBS 987.68 | JN206089 | JN206480 | – | – | – | – | Gajanayake et al. (2023) |
| *Mucor multiplex* | CBS 110662 T | NR_111662 | NG_057924 | – | – | – | – | Gajanayake et al. (2023) |
| *Mucor nanus* | CBS 774.86 | JN205907 | – | – | – | – | – | Gajanayake et al. (2023) |
| *Mucor nanus* | XY07221 | OL620142 | – | – | – | – | – | Gajanayake et al. (2023) |
| *Mucor nederlandicus* | CBS 735.70 | MH859923 | MH871720 | – | – | – | – | Gajanayake et al. (2023) |
| *Mucor nederlandicus* | MFLUCC 21-0045 | MZ433254 | MZ433251 | – | – | – | – | Gajanayake et al. (2023) |
| *Mucor nederlandicus* | MFLUCC 22-0070 | ON911966 | ON911967 | – | – | – | – | Gajanayake et al. (2023) |
| *Mucor nidicola* | H13 | KX375786 | KX375769 | – | – | – | – | Gajanayake et al. (2023) |
| *Mucor nidicola* | NRRL 54520 T | JF299215 |  | – | – | – | – | Gajanayake et al. (2023) |
| *Mucor odoratus* | CBS 130.41 T | NR_145287 | NG_057927 | – | – | – | – | Gajanayake et al. (2023) |
| *Mucor orantomantidis* | CNUFC-MID1-1 T | NR_164587 | NG_067828 | – | – | – | – | Gajanayake et al. (2023) |
| *Mucor orantomantidis* | CNUFC-MID1-2 | MH594738 | MH591458 | – | – | – | – | Gajanayake et al. (2023) |
| *Mucor orientalis* | CGMCC 3.16148 T | OL678208 | – | – | – | – | – | Zhao et al. (2023a) |
| *Mucor parviseptatus* | CBS 417.77 T | JN206108 | JN206453 | – | – | – | – | Gajanayake et al. (2023) |
| *Mucor parviseptatus* | CBS 522.79 | JN206109 | – | – | – | – | – | Gajanayake et al. (2023) |
| *Mucor pernambucoensis* | URM 7640 T | MH155323 | MH155322 | – | – | – | – | Gajanayake et al. (2023) |
| *Mucor phayaoensis* | MFLUCC 21-0043 T | MZ379498 | MZ379501 | – | – | – | – | Gajanayake et al. (2023) |
| ***Mucor phayaoensis*** | **GMBCC2471** | **PZ669406** | **PZ669419** | **–** | **–** | **–** | **–** | **This study** |
| ***Mucor phayaoensis*** | **GMBCC2472** | **PZ669405** | **PZ669418** | **–** | **–** | **–** | **–** | **This study** |
| *Mucor piriformis* | CBS 169.25 T | JN206028 | HM849681 | – | – | – | – | Gajanayake et al. (2023) |
| *Mucor plasmaticus* | CBS 275.49 | JN206078 | JN206483 | – | – | – | – | Gajanayake et al. (2023) |
| *Mucor plumbeus* | CBS 666.66 | MH858910 | MH870586 | – | – | – | – | Gajanayake et al. (2023) |
| *Mucor plumbeus* | CBS 226.32 | JN205916 | – | – | – | – | – | Gajanayake et al. (2023) |
| *Mucor plumbeus* | CBS:664.66 | MH858909 | MH870585 | – | – | – | – | Gajanayake et al. (2023) |
| *Mucor plumbeus* | VKM:F-561 | OR581584 | OR581584 | – | – | – | – | Gajanayake et al. (2023) |
| *Mucor prayagensis* | CBS 652.78 | JN206189 | JN206498 | – | – | – | – | Gajanayake et al. (2023) |
| *Mucor pseudocircinelloides* | CBS 541.78 | JN206013 | JN206431 | – | – | – | – | Gajanayake et al. (2023) |
| *Mucor pseudolusitanicus* | CBS 540.78 T | MF495059 | MT523852 | – | – | – | – | Gajanayake et al. (2023) |
| *Mucor pseudolusitanicus* | CBS 543.80 | MF495060 | – | – | – | – | – | Gajanayake et al. (2023) |
| *Mucor pseudolusitanicus* | CN054E6 | OR500017 | OR500017 | – | – | – | – | Gajanayake et al. (2023) |
| *Mucor pseudolusitanicus* | VKM:F 1110 | OR575483 | OR575483 | – | – | – | – | Gajanayake et al. (2023) |
| *Mucor racemosus* | CBS 115.08 | JN205919 | JN939201 | – | – | – | – | Gajanayake et al. (2023) |
| *Mucor racemosus* | CBS 260.68 T | NR_126135 | HM849676 | – | – | – | – | Gajanayake et al. (2023) |
| *Mucor radiatus* | CGMCC 3.16149 T | OL678209 | – | – | – | – | – | Zhao et al. (2023a) |
| *Mucor radiatus* | XY08167 | OL678210 | – | – | – | – | – | Gajanayake et al. (2023) |
| *Mucor ramosissimus* | CBS 135.65 T | JN205932 | NG_056280 | – | – | – | – | Gajanayake et al. (2023) |
| *Mucor rhizosporus* | CGMCC 3.16150 T | OL678211 | – | – | – | – | – | Zhao et al. (2023a) |
| *Mucor robustus* | CGMCC 3.16151 T | OL678212 | – | – | – | – | – | Zhao et al. (2023a) |
| *Mucor robustus* | XY08976 | OL678213 | – | – | – | – | – | Gajanayake et al. (2023) |
| *Mucor robustus* | XY09024 | OL678214 | – | – | – | – | – | Gajanayake et al. (2023) |
| *Mucor rongii* | CICC 41725 | MK903014 | MK903013 | – | – | – | – | Gajanayake et al. (2023) |
| *Mucor rongii* | CICC 41726 | MK903012 | MK903011 | – | – | – | – | Gajanayake et al. (2023) |
| *Mucor rudolphii* | WU 35867 | KT736104 | – | – | – | – | – | Gajanayake et al. (2023) |
| *Mucor rudolphii* | WU 35869 T | NR_152977 | – | – | – | – | – | Gajanayake et al. (2023) |
| *Mucor saturninus* | CBS 598.78 | JN206074 | – | – | – | – | – | Gajanayake et al. (2023) |
| *Mucor saturninus* | CBS 974.68 T | JN206072 | JN206458 | – | – | – | – | Gajanayake et al. (2023) |
| *Mucor septatiphorus* | URM7364_RV01 T | KY849814 | KY849816 | – | – | – | – | Gajanayake et al. (2023) |
| *Mucor septatiphorus* | URM7364_RV02 | KY849815 | KY849817 | – | – | – | – | Gajanayake et al. (2023) |
| *Mucor septatum* | URM 7364 | KY849814 | KY849816 | – | – | – | – | Gajanayake et al. (2023) |
| *Mucor silvaticus* | CBS 249.35 | JN206122 | JN206455 | – | – | – | – | Gajanayake et al. (2023) |
| *Mucor silvaticus* | CBS 412.71 T | JN206124 | MT523866 | – | – | – | – | Gajanayake et al. (2023) |
| *Mucor sinensis* | CBS 204.74 | JN205899 | – | – | – | – | – | Gajanayake et al. (2023) |
| *Mucor sinosaturninus* | CGMCC 3.16152 T | OL678215 | – | – | – | – | – | Zhao et al. (2023a) |
| *Mucor sinosaturninus* | XY08556 | OL678216 | – | – | – | – | – | Gajanayake et al. (2023) |
| *Mucor souzae* | URM 7553 T | NR_165210 | NG_067797 | – | – | – | – | Gajanayake et al. (2023) |
| *Mucor souzae* | URM 91186 | KY992878 | KY992879 | – | – | – | – | Gajanayake et al. (2023) |
| *Mucor stercorarius* | CNUFC-UK2-1 T | KX839689 | KX839685 | – | – | – | – | Gajanayake et al. (2023) |
| *Mucor stercorarius* | CNUFC-UK2-2 | KX839680 | KX839682 | – | – | – | – | Gajanayake et al. (2023) |
| *Mucor strictus* | CBS 100.66 | JN206035 | JN206477 | – | – | – | – | Gajanayake et al. (2023) |
| *Mucor strictus* | CBS 576.66 T | NR_103631 | NG_076700 | – | – | – | – | Gajanayake et al. (2023) |
| *Mucor takensis* | MFLUCC 21-0041 T | MZ379497 | MZ379500 | – | – | – | – | Gajanayake et al. (2023) |
| *Mucor thermorhizoides* | CBS 149760 T | OQ034234 | OR244328 | – | – | – | – | Gajanayake et al. (2023) |
| *Mucor ucrainicus* | CBS 221.71 | MH860077 | MT523853 | – | – | – | – | Gajanayake et al. (2023) |
| *Mucor ucrainicus* | CBS 674.88 | JN206192 | JN206507 | – | – | – | – | Gajanayake et al. (2023) |
| *Mucor variicolumellatus* | CBS 236.35 T | JN205979 | JN206422 | – | – | – | – | Gajanayake et al. (2023) |
| *Mucor variicolumellatus* | CF:01 | MT124621 | – | – | – | – | – | Gajanayake et al. (2023) |
| *Mucor variicolumellatus* | [CF:02](https://www.ncbi.nlm.nih.gov/nuccore/MT124622.1) | MT124622 | – | – | – | – | – | Gajanayake et al. (2023) |
| *Mucor variisporus* | CBS 837.70 T | NR_152951 | NG_057972 | – | – | – | – | Gajanayake et al. (2023) |
| *Mucor velutinosus* | CBS 144.93 | JN206012 | – | – | – | – | – | Gajanayake et al. (2023) |
| *Mucor velutinosus* | CBS 762.74 | JN206011 | JN206416 | – | – | – | – | Gajanayake et al. (2023) |
| *Mucor yunnanensis* | ZHKUCC 22-0109 | ON921545 | ON921549 | – | – | – | – | Gajanayake et al. (2023) |
| *Mucor yunnanensis* | ZHKUCC 22-0110 T | ON921544 | ON921546 | – | – | – | – | Gajanayake et al. (2023) |
| *Mucor zonatus* | CBS 148.69 T | NR_103638 | NG_057917 | – | – | – | – | Gajanayake et al. (2023) |
| *Mucor zychae* | CBS 416.67 | NR_103641 | NG_057930 | – | – | – | – | Gajanayake et al. (2023) |
| *Myceliophthora lutea* | CBS 145.77 T | HQ871775 | KM655351 | KX977026 | KM655395 | – | – | Wang et al. (2022) |
| *Myceliophthora lutea* | CBS 379.76 | MK919302 | MK919302 | MK919416 | MK919359 | – | – | Wang et al. (2022) |
| *Mycothermus thermophiloides* | CBS 183.81 T | LT993603 | LT993603 | LT993684 | LT993522 | – | – | Wang et al. (2022) |
| *Mycothermus thermophilus* | CBS 625.91 T | LT993604 | LT993604 | LT993685 | LT993523 | – | – | Wang et al. (2022) |
| ***Neoachaetomiella cavernicola*** | **GMBCC2270 T** | **PZ669399** | **PZ669412** | **PZ871001** | **PZ867820** | **–** | **–** | **This studys** |
| ***Neoachaetomiella cavernicola*** | **GZCC 25-0730** | **PZ669400** | **PZ669413** | **PZ871002** | **PZ867821** | **–** | **–** | **This studys** |
| *Ovatospora medusarum* | CBS 148.67 T | KX976684 | KX976782 | KX977032 | KX976897 | – | – | Wang et al. (2022) |
| *Ovatospora mollicella* | CBS 583.83 T | KX976685 | KX976783 | KX977033 | KX976898 | – | – | Wang et al. (2022) |
| *Ovatospora senegalensis* | CBS 728.84 T | KX976687 | KX976785 | KX977035 | KX976900 | – | – | Wang et al. (2022) |
| *Ovatospora unipora* | CBS 109.83 T | KX976689 | KX976787 | KX977037 | KX976902 | – | – | Wang et al. (2022) |
| *Parachaetomium carinthiacum* | CBS 153.81 T | MK919298 | MK919298 | MK919412 | MK919355 | – | – | Wang et al. (2022) |
| *Parachaetomium carinthiacum* | CBS 665.82 | MK919299 | MK919299 | MK919413 | MK919356 | – | – | Wang et al. (2022) |
| *Parachaetomium hispanicum* | CBS 234.82 T | MK919304 | MK919304 | MK919418 | MK919361 | – | – | Wang et al. (2022) |
| *Parachaetomium inaequale* | CBS 331.75 T | MK919306 | MK919306 | MK919420 | MK919363 | – | – | Wang et al. (2022) |
| *Parachaetomium longiciliatum* | CGMCC 3.17554 T | KP336774 | KP336823 | KP336872 | KT149497 | – | – | Wang et al. (2022) |
| *Parachaetomium multispirale* | CBS 172.84 T | MH861718 | – | MZ343017 | MZ342978 | – | – | Wang et al. (2022) |
| *Parachaetomium perlucidum* | CBS 126670 | MK919310 | – | MK919424 | MK919367 | – | – | Wang et al. (2022) |
| *Parachaetomium perlucidum* | CBS 127795 | MK919311 | MH876143 | MK919425 | MK919368 | – | – | Wang et al. (2022) |
| *Parachaetomium perlucidum* | CBS 141.58 | MK919308 | MH869266 | MK919422 | MK919365 | – | – | Wang et al. (2022) |
| *Parachaetomium truncatulum* | CBS 126782 T | HM365263 | HM365263 | HM365298 | MT568849 | – | – | Wang et al. (2022) |
| *Parathielavia appendiculata* | CBS 723.68 T | MK926827 | MK926827 | MK926927 | MK876788 | – | – | Wang et al. (2022) |
| *Parathielavia coactilis* | CBS 101190 T | – | – | MZ343003 | MZ342962 | – | – | Wang et al. (2022) |
| *Parathielavia hyrcaniae* | CBS 353.62 T | KM655329 | KM655368 | KX977043 | KM655401 | – | – | Wang et al. (2022) |
| *Parathielavia kuwaitensis* | CBS 945.72 T | KM655332 | KM655371 | KX977044 | KM655404 | – | – | Wang et al. (2022) |
| *Parvomelanocarpus tardus* | CBS 541.76 T | KX976681 | KX976775 | KX977023 | KX976888 | – | – | Wang et al. (2022) |
| *Parvomelanocarpus thermophilus* | CBS 886.97 | KM655350 | MH874288 | MZ343037 | KM655434 | – | – | Wang et al. (2022) |
| *Petriella sordida* | CBS 124169 | GQ426957 | AY281099 | – | – | – | – | Sandoval-Denis et al. (2016) |
| *Petriellopsis africana* | CBS 311.72 T | AJ888425 | EF151331 | – | – | – | – | Sandoval-Denis et al. (2016) |
| *Pithoascus ater* | CBS 400.34 T | LM652447 | LM652526 | LM652659 | – | LM652576 | – | Sandoval-Denis et al. (2016) |
| ***Pithoascus cavernicola*** | **GMBCC2294 T** | **PZ669397** | **PZ669410** | – | – | **PZ871021** | – | **This study** |
| ***Pithoascus cavernicola*** | **GMBCC2476** | **PZ669398** | **PZ669411** | – | – | **PZ871022** | – | **This study** |
| *Pithoascus exsertus* | CBS 583.75 | LM652448 | LM652527 | LM652660 | – | LM652577 | – | Sandoval-Denis et al. (2016) |
| *Pithoascus exsertus* | CBS 819.70 T | LM652449 | LM652528 | LM652661 | – | LM652578 | – | Sandoval-Denis et al. (2016) |
| *Pithoascus intermedius* | CBS 217.32 T | LM652450 | LM652529 | LM652662 | – | LM652579 | – | Sandoval-Denis et al. (2016) |
| *Pithoascus lunatus* | CBS 103.85 T | LN850784 | LN850833 | LN850881 | – | LN850929 | – | Sandoval-Denis et al. (2016) |
| *Pithoascus nidicola* | CBS 197.61 T | LM652451 | LM652530 | LM652663 | – | LM652580 | – | Sandoval-Denis et al. (2016) |
| *Pithoascus persicus* | IRAN 3309C T | MF186873 | MH400206 | – | – | MK430530 | – | Sandoval-Denis et al. (2016) |
| *Pithoascus stoveri* | CBS 176.71 T | LM652453 | LM652532 | LM652664 | – | LM652581 | – | Sandoval-Denis et al. (2016) |
| *Protocrea farinosa* | CBS 121551 T | MH863119 | – | – | EU703935 | – | – | Liu et al. (2025) |
| *Protocrea illinoensis* | GJS 94-54 | EU703929 | – | – | EU703951 | EU703904 | – | Liu et al. (2025) |
| *Protocrea illinoensis* | TFC 9698 T | EU703930 | – | – | EU703952 | EU703905 | – | Liu et al. (2025) |
| *Protocrea pallida* | CBS 29878 | EU703924 | – | – | EU703947 | EU703899 | – | Liu et al. (2025) |
| *Protocrea pallida* | CBS 29978 T | EU703925 | – | – | EU703948 | EU703900 | – | Liu et al. (2025) |
| *Pseudohumicola atrobrunnea* | HSAUP:II05-1004 | LT993570 | LT993570 | LT993651 | LT993489 | – | – | Wang et al. (2022) |
| *Pseudohumicola pulvericola* | DTO:318-G9 | LT993591 | LT993591 | LT993672 | LT993510 | – | – | Wang et al. (2022) |
| *Pseudohumicola semispirali* | CBS 723.97 | LT993597 | LT993597 | LT993678 | LT993516 | – | – | Wang et al. (2022) |
| *Pseudohumicola subspiralis* | CBS 148.58 | LT993599 | LT993599 | LT993680 | LT993518 | – | – | Wang et al. (2022) |
| *Pseudoscopulariopsis hibernica* | UAMH 2643 | LM652454 | LM652533 | LM652665 | – | LM652582 | – | Sandoval-Denis et al. (2016) |
| *Pseudoscopulariopsis schumacheri* | CBS 435.86 T | LM652455 | LM652534 | LM652666 | – | LM652583 | – | Sandoval-Denis et al. (2016) |
| *Pseudothielavia hamadae* | CBS 499.83 T | MK926832 | MK926832 | MK926932 | MK876793 | – | – | Wang et al. (2022) |
| *Pseudothielavia subhyaloderma* | CBS 473.86 | MK926833 | MK926833 | MK926933 | MK876794 | – | – | Wang et al. (2022) |
| *Pseudothielavia terricola* | CBS 165.88 T | KX976694 | KX976792 | KX977045 | MK876795 | – | – | Wang et al. (2022) |
| *Pyrenopolyporus hunteri* | MUCL 52673 | KY610421 | KY610472 | KU159530 | KY624309 | – | – | Wongkanoun et al. (2020) |
| *Pyrenopolyporus laminosus* | MUCL 53305 | KC968934 | KY610485 | KC977292 | KY624303 | – | – | Wongkanoun et al. (2020) |
| *Pyrenopolyporus laminosus* | TBRC 8871 | MH938527 | MH938536 | MK165415 | MK165424 | – | – | Wongkanoun et al. (2020) |
| *Pyrenopolyporus nicaraguensis* | CBS 117739 | AM749922 | KY610489 | KC977272 | KY624307 | – | – | Wongkanoun et al. (2020) |
| *Pyrenopolyporus symphyon* | TBRC 8873 | MH938529 | MH938538 | MK165419 | MK165428 | – | – | Wongkanoun et al. (2020) |
| *Remersonia tenuis* | CBS 784.85 T | LT993609 | LT993609 | LT993690 | LT993528 | – | – | Wang et al. (2022) |
| *Remersonia thermophila* | CBS 645.91 | JF412016 | – | LT993692 | – | – | – | Wang et al. (2022) |
| *Scedosporium aurantiacum* | CBS 116910 | HQ231818 | EF151326 | – | – | – | – | Sandoval-Denis et al. (2016) |
| *Scedosporium boydii* | CBS 330.93 | AY863196 | AY882372 | – | – | – | – | Sandoval-Denis et al. (2016) |
| *Scopulariopsis acremonium* | CBS 290.38 T | LM652456 | HG380439 | – | – | HG380362 | – | Sandoval-Denis et al. (2016) |
| *Scopulariopsis acremonium* | MUCL 8274 | LM652457 | LM652535 | – | – | – | – | Sandoval-Denis et al. (2016) |
| *Scopulariopsis acremonium* | MUCL 8409 | LM652458 | LM652536 | – | – | – | – | Sandoval-Denis et al. (2016) |
| *Scopulariopsis asperula* | CBS 289.38 T | LM652464 | LM652538 | LM652671 | – | LM652585 | – | Sandoval-Denis et al. (2016) |
| *Scopulariopsis asperula* | CBS 401.34 T | LM652463 | HG380465 | LM652670 | – | HG380388 | – | Sandoval-Denis et al. (2016) |
| *Scopulariopsis asperula* | MUCL 40729 | LM652459 | LM652537 | LM652667 | – | LM652584 | – | Sandoval-Denis et al. (2016) |
| *Scopulariopsis asperula* | UTHSC 10-3405 | LM652462 | HG380461 | LM652669 | – | HG380384 | – | Sandoval-Denis et al. (2016) |
| *Scopulariopsis brevicaulis* | CBS 335.35 | LM652477 | LM652542 | LM652684 | – | LM652588 | – | Sandoval-Denis et al. (2016) |
| *Scopulariopsis brevicaulis* | MUCL 14213 | LM652479 | LM652544 | LM652686 | – | LM652590 | – | Sandoval-Denis et al. (2016) |
| *Scopulariopsis brevicaulis* | MUCL 40726 T | LM652465 | HG380440 | LM652672 | – | HG380363 | – | Sandoval-Denis et al. (2016) |
| *Scopulariopsis brevicaulis* | UTHSC 06-619 | LM652468 | HG380442 | LM652675 | – | HG380365 | – | Sandoval-Denis et al. (2016) |
| *Scopulariopsis brevicaulis* | UTHSC 07-1888 | LM652471 | HG380444 | LM652678 | – | HG380367 | – | Sandoval-Denis et al. (2016) |
| *Scopulariopsis brevicaulis* | UTHSC 09-1092 | LM652472 | HG380445 | LM652679 | – | HG380368 | – | Sandoval-Denis et al. (2016) |
| *Scopulariopsis candida* | CBS 119.43 | LM652482 | LM652547 | LM652688 | – | LM652592 | – | Sandoval-Denis et al. (2016) |
| *Scopulariopsis candida* | CBS 170.27 T | LM652488 | LM652549 | LM652694 | – | LM652594 | – | Sandoval-Denis et al. (2016) |
| *Scopulariopsis candida* | MUCL 40743 T | LM652484 | HG380458 | LM652690 | – | HG380381 | – | Sandoval-Denis et al. (2016) |
| *Scopulariopsis candida* | UTHSC 09-2576 | LM652486 | HG380459 | LM652692 | – | HG380382 | – | Sandoval-Denis et al. (2016) |
| *Scopulariopsis candida* | UTHSC 09-3241 | LM652485 | HG380460 | LM652691 | – | HG380383 | – | Sandoval-Denis et al. (2016) |
| *Scopulariopsis cordiae* | CBS 138129 T | LM652491 | HG380499 | LM652673 | – | HG380422 | – | Sandoval-Denis et al. (2016) |
| *Scopulariopsis cordiae* | UTHSC 05-3453 | LM652492 | HG380498 | LM652696 | – | HG380421 | – | Sandoval-Denis et al. (2016) |
| *Scopulariopsis flava* | CBS 207.61 T | LM652493 | HG380464 | LM652697 | – | HG380387 | – | Sandoval-Denis et al. (2016) |
| *Scopulariopsis soppii* | UAMH 9169 T | LM652495 | LM652552 | LM652698 | – | LM652595 | – | Sandoval-Denis et al. (2016) |
| *Staphylotrichum acaciicola* | CBS 281.65 T | LT993613 | LT993613 | LT993694 | LT993532 | – | – | Wang et al. (2022) |
| *Staphylotrichum brevistipitatum* | CBS 408.67 T | LT993619 | LT993619 | LT993700 | LT993538 | – | – | Wang et al. (2022) |
| *Staphylotrichum coccosporum* | CBS 364.58 T | LT993620 | LT993620 | LT993701 | LT993539 | – | – | Wang et al. (2022) |
| *Staphylotrichum limonisporum* | CGMCC 3.17914 T | KU746672 | KU746718 | KU746764 | KY575867 | – | – | Wang et al. (2022) |
| *Staphylotrichum microascosporum* | CBS 184.79 T | LT993624 | LT993624 | LT993705 | LT993543 | – | – | Wang et al. (2022) |
| *Staphylotrichum tortipilum* | CBS 103.79 T | LT993625 | LT993625 | LT993706 | LT993544 | – | – | Wang et al. (2022) |
| *Stellatospora terricola* | CBS 811.95 T | MK926835 | MH874188 | MK926935 | MK876797 | – | – | Wang et al. (2022) |
| *Stolonocarpus gigasporus* | CBS 112062 T | MK926836 | MK926836 | MK926936 | MK876798 | – | – | Wang et al. (2022) |
| *Subramaniula lateralis* | CGMCC 3.17547 | KP336789 | KP336838 | KP336887 | MZ342998 | – | – | Wang et al. (2022) |
| *Subramaniula latifusispora* | CGMCC 20442 | MZ334728 | MZ351428 | MZ343040 | MZ343001 | – | – | Wang et al. (2022) |
| *Subramaniula latifusispora* | WXW 8577 | MZ334729 | MZ351427 | MZ343041 | MZ343002 | – | – | Wang et al. (2022) |
| *Subramaniula thielavioides* | CBS 122.78 T | KP862597 | KP970654 | KP900708 | KP900670 | – | – | Wang et al. (2022) |
| *Tengochaeta nigropilosa* | CBS 639.83 T | MZ334730 | – | MZ343029 | MZ342990 | – | – | Wang et al. (2020) |
| *Thermocarpiscus australiensis* | CBS 493.74 T | KM655339 | KM655378 | MZ343024 | KM655419 | – | – | Wang et al. (2022) |
| *Thermochaetoides dissita* | CBS 180.67 | MK919319 | MK919319 | MK919433 | MK919375 | – | – | Wang et al. (2022) |
| *Thermochaetoides dissita* | CBS 246.90 | MK919320 | MK919320 | MK919434 | MK919376 | – | – | Wang et al. (2022) |
| *Thermochaetoides thermophila* | CBS 143.50 | MK919315 | MK919315 | MK919429 | MK919371 | – | – | Wang et al. (2022) |
| *Thermochaetoides thermophila* | CBS 144.50 T | MK919314 | MK919314 | MK919428 | KM655436 | – | – | Wang et al. (2022) |
| *Thermothelomyces guttulatus* | CGMCC 3.15185T | MK919323 | MK919323 | MK919437 | MK919380 | – | – | Wang et al. (2022) |
| *Thermothelomyces hinnuleus* | CBS 597.83 T | MK919327 | MK919327 | MK919441 | MK919384 | – | – | Wang et al. (2022) |
| *Thermothelomyces myriococcoides* | CBS 389.93 T | MK919329 | MK919329 | MK919443 | MK919386 | – | – | Wang et al. (2022) |
| *Thermothelomyces thermophilus* | CBS 117.65 T | MK919331 | MK919331 | MK919445 | MK919387 | – | – | Wang et al. (2022) |
| *Thermothielavioides terrestris* | CBS 117535 T | MK926837 | MK926837 | MK926937 | MK876799 | – | – | Wang et al. (2022) |
| *Thermothielavioides terrestris* | CBS 492.74 | MK926838 | MK926838 | MK926938 | – | – | – | Wang et al. (2022) |
| *Trichocladium acropullum* | CBS 114580 T | LT993626 | LT993626 | LT993707 | LT993545 | – | – | Wang et al. (2022) |
| *Trichocladium acropullum* | CBS 127763 T | LT993628 | LT993628 | LT993709 | LT993547 | – | – | Wang et al. (2022) |
| *Trichocladium antarcticum* | CBS 123565 T | LT993629 | LT993629 | LT993710 | LT993548 | – | – | Wang et al. (2022) |
| *Trichocladium antarcticum* | CBS 135876 | LT993630 | LT993630 | LT993711 | LT993549 | – | – | Wang et al. (2022) |
| *Trichocladium arxii* | CBS 104.79 T | LT993631 | LT993631 | LT993712 | LT993550 | – | – | Wang et al. (2022) |
| *Trichocladium asperum* | CBS 140.21 | LT993633 | LT993633 | LT993714 | LT993552 | – | – | Wang et al. (2022) |
| *Trichocladium asperum* | CBS 157.22 | LT993634 | LT993634 | LT993715 | LT993553 | – | – | Wang et al. (2022) |
| *Trichocladium asperum* | CBS 903.85 T | LT993632 | LT993632 | LT993713 | LT993551 | – | – | Wang et al. (2022) |
| *Trichocladium beniowskiae* | CBS 757.74 T | LT993635 | LT993635 | LT993716 | LT993554 | – | – | Wang et al. (2022) |
| ***Trichocladium cavernicola*** | **GZCC 25-0715 T** | **PZ669402** | **PZ669415** | **–** | **PZ867823** | **–** | **–** | **This study** |
| ***Trichocladium cavernicola*** | **GZCC 25-0716** | **PZ669403** | **PZ669416** | **–** | **PZ867824** | **–** | **–** | **This study** |
| *Trichocladium crispatum* | CBS 149.58 T | LT993636 | LT993636 | LT993717 | LT993555 | – | – | Wang et al. (2022) |
| *Trichocladium crispatum* | CBS 693.71 | LT993637 | LT993637 | LT993718 | LT993556 | – | – | Wang et al. (2022) |
| *Trichocladium gilmaniellae* | CBS 388.75 ^T^ | LT993638 | LT993638 | LT993719 | LT993557 | – | – | Wang et al. (2022) |
| *Trichocladium griseum* | CBS 119.14 NeoT | LT993639 | LT993639 | LT993720 | LT993558 | – | – | Wang et al. (2022) |
| *Trichocladium griseum* | CBS 217.34 | LT993640 | LT993640 | LT993721 | LT993559 | – | – | Wang et al. (2022) |
| *Trichocladium jilongensis* | CBS 195.87 | LT993643 | LT993643 | LT993724 | LT993562 | – | – | Wang et al. (2022) |
| *Trichocladium jilongensis* | HSAUPII07 1485 ^T^ | LT993642 | LT993642 | LT993723 | LT993561 | – | – | Wang et al. (2022) |
| *Trichocladium nigrospermum* | CBS 103.36 | LT993644 | LT993644 | LT993725 | LT993563 | – | – | Wang et al. (2022) |
| *Trichocladium seminis-citrulli* | CBS 143.58 ^T^ | LT993645 | LT993645 | LT993726 | LT993564 | – | – | Wang et al. (2022) |
| *Trichocladium seminis-citrulli* | CBS 637.83 | LT993646 | LT993646 | LT993727 | LT993565 | – | – | Wang et al. (2022) |
| *Trichocladium solani* | VKM F-4902 ^T^ | OL691125 | – | OL314244 | OM974683 | – | – | Belosokhov et al. (2022) |
| *Trichocladium solani* | VKM F-4913 | OL691123 | – | OL314246 | OL314251 | – | – | Belosokhov et al. (2022) |
| *Trichocladium solani* | VKM F-4914 | OL691121 | – | OL314248 | OL314252 | – | – | Belosokhov et al. (2022) |
| *Trichocladium solani* | VKM F-4915 | OL638311 | – | OL674250 | OL442086 | – | – | Belosokhov et al. (2022) |
| *Trichocladium tomentosum* | CBS 144476 T | MZ334732 | MZ351431 | MZ343012 | MZ342973 | – | – | Wang et al. (2022) |
| *Trichocladium uniseriatum* | LC3756 | KP336751 | KP336800 | KP336849 | KT149475 | – | – | Wang et al. (2022) |
| *Trichoderma estonicum* | GJS 96-129 T | AY737767 | – | – | AF545514 | AY737733 | – | Liu et al. (2025) |
| *Trichoderma istrianum* | CBS 130539 T | – | – | – | KJ665281 | KJ665523 | – | Liu et al. (2025) |
| *Trichoderma istrianum* | S123 | – | – | – | KJ665280 | KJ665521 | – | Liu et al. (2025) |
| *Trichoderma linzhiense* | HMAS 248846 T | KY687929 | – | – | KY687985 | KY688047 | – | Liu et al. (2025) |
| *Trichoderma linzhiense* | HMAS 248874 | KY687957 | – | – | KY688011 | KY688048 | – | Liu et al. (2025) |
| *Trichoderma paratroviride* | CBS 136489 T | – | – | – | KJ665321 | KJ665627 | – | Liu et al. (2025) |
| *Trichoderma paratroviride* | S489 | – | – | – | KJ665322 | KJ665628 | – | Liu et al. (2025) |
| *Trichoderma polypor* | HMAS 248855 T | KY687938 | – | – | KY687994 | KY688058 | – | Liu et al. (2025) |
| *Trichoderma pubescens* | DAOM 166162 T | EU280121 | – | – | KJ842138 | EU279963 | – | Liu et al. (2025) |
| *Trichoderma simmonsii* | CBS 130431 | AF443917 | – | – | FJ442757 | AF443935 | – | Liu et al. (2025) |
| *Trichoderma simmonsii* | S7 | – | – | – | KJ665337 | KJ665719 | – | Liu et al. (2025) |
| *Trichoderma velutinum* | DAOM 230013 T | – | – | – | KF134794 | KJ665769 | – | Liu et al. (2025) |
| *Trichoderma achlamydosporum* | YMF 1.06226 T | MN977791 | – | – | MT052180 | MT070156 | – | Liu et al. (2025) |
| *Trichoderma afarasin* | BAFC 4355 | – | – | – | – | MG755812 | – | Liu et al. (2025) |
| *Trichoderma afarasin* | Dis 314 | FJ442259 | – | – | FJ442778 | FJ463400 | – | Liu et al. (2025) |
| *Trichoderma afarasin* | DIS 377a T | FJ442665 | – | – | FJ442799 | FJ463322 | – | Liu et al. (2025) |
| *Trichoderma afroharzianum* | CBS 124620 T | FJ442265 | – | – | FJ442691 | FJ463301 | – | Liu et al. (2025) |
| *Trichoderma afroharzianum* | Fes1712 | – | – | – | MW014361 | MW014362 | – | Liu et al. (2025) |
| *Trichoderma afroharzianum* | NAIMCC-F-01938 | KY419889 | – | – | KY419895 | KY419891 | – | Liu et al. (2025) |
| *Trichoderma aggressivum* | COAD 2432 | – | – | – | MK044249 | MK044156 | – | Liu et al. (2025) |
| *Trichoderma albofulvopsis* | 9930 T | – | – | – | KU529138 | KU529127 | – | Liu et al. (2025) |
| *Trichoderma amoenum* | YMF 1.06209 T | MN977801 | – | – | MT052192 | MT070146 | – | Liu et al. (2025) |
| *Trichoderma amoenum* | YMF 1.06210 | MN977802 | – | – | MT070161 | MT070145 | – | Liu et al. (2025) |
| *Trichoderma anaharzianum* | YMF 1.00241 | MH262584 | – | – | MH262577 | MH236493 | – | Liu et al. (2025) |
| *Trichoderma anaharzianum* | YMF 1.00383 T | MH113931 | – | – | MH158995 | MH183182 | – | Liu et al. (2025) |
| *Trichoderma andinense* | COAD 2431 | – | – | – | MK044248 | MK044155 | – | Liu et al. (2025) |
| *Trichoderma anisohamatum* | YMF 1.00253 | MH262586 | – | – | MH262578 | MH236495 | – | Liu et al. (2025) |
| *Trichoderma anisohamatum* | YMF 1.00333 T | MH262583 | – | – | MH262576 | MH236494 | – | Liu et al. (2025) |
| *Trichoderma aquatica* | YMF 1.04624 | MH383057 | – | – | MK775511 | MK775506 | – | Liu et al. (2025) |
| *Trichoderma aquatica* | YMF 1.04625 T | MH383058 | – | – | MK775512 | MK775507 | – | Liu et al. (2025) |
| *Trichoderma arenarium* | TUCIM 10301 T | MT217123 | – | – | MT242310 | MT242303 | – | Liu et al. (2025) |
| *Trichoderma arenarium* | TUCIM 10302 | – | – | – | MT347691 | MT347687 | – | Liu et al. (2025) |
| *Trichoderma asiaticum* | YMF 1.00168 | MH262582 | – | – | MH262575 | MH236492 | – | Liu et al. (2025) |
| *Trichoderma asiaticum* | YMF 1.00352 T | MH113930 | – | – | MH158994 | MH183183 | – | Liu et al. (2025) |
| *Trichoderma asymmetricum* | YMF 1.04618 T | MK795991 | – | – | MK795987 | MK795983 | – | Liu et al. (2025) |
| *Trichoderma atrobrunneum* | CBS 548.92 T | AF443924 | – | – | – | AF443942 | – | Liu et al. (2025) |
| *Trichoderma atrobrunneum* | GJS 04-67 | – | – | – | FJ442724 | FJ463360 | – | Liu et al. (2025) |
| *Trichoderma atrosphaericum* | KUN-HKAS 146599 T | PV426441 | – | – | – | – | – | Liu et al. (2025) |
| *Trichoderma atrosphaericum* | KUN-HKAS 146600 | PV426442 | – | – | – | – | – | Liu et al. (2025) |
| *Trichoderma atroviride* | CBS 119499 | FJ860726 | – | – | FJ860518 | FJ860611 | – | Liu et al. (2025) |
| *Trichoderma atroviride* | CBS 142.95 T | AF456917 | – | – | EU341801 | AY376051 | – | Liu et al. (2025) |
| *Trichoderma atroviride* | COAD 2396 | – | – | – | MK044177 | MK044083 | – | Liu et al. (2025) |
| *[Trichoderma auriculariae](https://blast.ncbi.nlm.nih.gov/Blast.cgi#alnHdr_2644347111)* | JZBQT1Z7 T | ON653396 | – | – | ON649949 | ON649896 | – | Liu et al. (2025) |
| *[Trichoderma auriculariae](https://blast.ncbi.nlm.nih.gov/Blast.cgi#alnHdr_2644347111)* | JZBQT1Z8 | ON653397 | – | – | ON649950 | ON649897 | – | Liu et al. (2025) |
| *[Trichoderma auriculariae](https://blast.ncbi.nlm.nih.gov/Blast.cgi#alnHdr_2644347111)* | JZBQT1Z9 | ON653398 | – | – | ON649951 | ON649898 | – | Liu et al. (2025) |
| *Trichoderma austroindianum* | GJS 08-128 | – | – | – | – | MH352423 | – | Liu et al. (2025) |
| *Trichoderma austroindianum* | VAB-T050 T | – | – | – | – | MH352421 | – | Liu et al. (2025) |
| *Trichoderma awajun* | CHAXC CP24-7 | – | – | – | MW480147 | MW480138 | – | Liu et al. (2025) |
| *Trichoderma awajun* | CHAXC CP24-8 | – | – | – | MW480148 | MW480139 | – | Liu et al. (2025) |
| *Trichoderma azevedoi* | CEN1422 T | MK714902 | – | – | MK696821 | MK696660 | – | Liu et al. (2025) |
| *Trichoderma azevedoi* | CEN1423 | MK714903 | – | – | MK696822 | MK696661 | – | Liu et al. (2025) |
| *Trichoderma bannaense* | HMAS 248840 T | KY687923 | – | – | KY687979 | KY688037 | – | Liu et al. (2025) |
| *Trichoderma bannaense* | HMAS 248865 | KY687948 | – | – | KY688003 | KY688038 | – | Liu et al. (2025) |
| *Trichoderma bashaniae* | SICAU 25-0179 | PV789471 | – | – | PV828316 | PV828324 | – | Liu et al. (2025) |
| *Trichoderma bashaniae* | SICAU 25-0180 T | PV789472 | – | – | PV828317 | PV828325 | – | Liu et al. (2025) |
| *Trichoderma botryosum* | COAD 2520 | – | – | – | MK044233 | MK044140 | – | Liu et al. (2025) |
| *Trichoderma botryosum* | COAD 2543 | – | – | – | MK044234 | MK044141 | – | Liu et al. (2025) |
| *Trichoderma breve* | CGMCC 3.18398 T | KY687927 | – | – | KY687983 | KY688045 | – | Liu et al. (2025) |
| *Trichoderma breve* | HMAS 248845 | KY687928 | – | – | KY687984 | KY688046 | – | Liu et al. (2025) |
| *Trichoderma caeruleum* | GDMCC 3.1021 T | – | – | – | OR779496 | OR779525 | – | Liu et al. (2025) |
| *Trichoderma caeruleum* | YNE01448 | – | – | – | OR779497 | OR779526 | – | Liu et al. (2025) |
| *Trichoderma caeruloviride* | COAD 2415 T | – | – | – | MK044202 | MK044109 | – | Liu et al. (2025) |
| *Trichoderma caeruloviride* | COAD 2416 | – | – | – | MK044201 | MK044108 | – | Liu et al. (2025) |
| *Trichoderma caespitosus* | JZBQT1Z12 | – | – | – | OP832384 | OP832399 | – | Liu et al. (2025) |
| *Trichoderma caespitosus* | JZBQT1Z6 T | – | – | – | OP832383 | OP832398 | – | Liu et al. (2025) |
| *Trichoderma camelliae* | JZBH336000 T | MW008452 | – | – | – | – | – | Liu et al. (2025) |
| *Trichoderma camelliae* | JZBH3360002 T | MW008451 | – | – | – | – | – | Liu et al. (2025) |
| *Trichoderma camerunense* | GJS 99-230 T | – | – | – | – | AF348107 | – | Liu et al. (2025) |
| *Trichoderma camerunense* | GJS 99-231 | AY027783 | – | – | – | AF348108 | – | Liu et al. (2025) |
| *Trichoderma capillare* | G.J.S. 06-66 | – | – | – | JN175530 | JN175585 | – | Liu et al. (2025) |
| *Trichoderma capillare* | G.J.S. 10-170 T | – | – | – | JN182312 | JN182283 | – | Liu et al. (2025) |
| *Trichoderma caribbaeum* | GJS 97-3 T | NR_166015 | – | – | KJ665246 | KJ665443 | – | Liu et al. (2025) |
| *Trichoderma catoptron* | GJS 02-76 T | AY737766 | – | – | AY391900 | AY737726 | – | Liu et al. (2025) |
| *Trichoderma catoptron* | HMAS 252533 | – | – | – | KF923310 | KF923290 | – | Liu et al. (2025) |
| *Trichoderma cavernicola* | GMBCC1103 T | PQ654705 | – | – | PV067157 | PV132334 | – | Liu et al. (2025) |
| *Trichoderma cavernicola* | GMBCC1105 | PQ654706 | – | – | PV067158 | PV132335 | – | Liu et al. (2025) |
| *Trichoderma ceraceum* | DAOM 232831 | – | – | – | – | KJ871239 | – | Liu et al. (2025) |
| *Trichoderma ceraceum* | GJS 95-159 T | AF275332 | – | – | AF545508 | AY937437 | – | Liu et al. (2025) |
| *Trichoderma ceramicum* | CBS114576 T | FJ860743 | – | – | FJ860531 | FJ860628 | – | Liu et al. (2025) |
| *Trichoderma ceratophylli* | YMF 1.04621 T | MK327581 | – | – | MK327580 | MK327579 | – | Liu et al. (2025) |
| *Trichoderma cerinum* | DAOM 230012 T | – | – | – | KJ842184 | KJ871242 | – | Liu et al. (2025) |
| *Trichoderma cinnamomeum* | GJS 97-233 | – | – | – | AY391919 | AY391978 | – | Liu et al. (2025) |
| *Trichoderma cinnamomeum* | GJS 97-237 | AY737759 | – | – | AY391920 | AY391979 | – | Liu et al. (2025) |
| *Trichoderma citri* | ZHKUCC24-0779 T | PP789092 | – | – | PP848451 | PP848435 | – | Liu et al. (2025) |
| *Trichoderma citri* | ZHKUCC24-0780 | PP789093 | – | – | PP848452 | PP848436 | – | Liu et al. (2025) |
| *Trichoderma clavaticapitatum* | HKAS 131918 T | – | – | – | OR965857 | OR965859 | – | Liu et al. (2025) |
| ***Trichoderma dehongense*** | **GMBCC2301 T** | **PZ669378** | **–** | **–** | **PZ867801** | **PZ871005** | – | **This study** |
| ***Trichoderma dehongense*** | **GZCC 25-0720** | **PZ669379** | **–** | **–** | **PZ867802** | **PZ871006** | – | **This study** |
| *Trichoderma densissimum* | CGMCC 3.24126 T | – | – | – | OP357966 | OP357971 | – | Liu et al. (2025) |
| *Trichoderma densissimum* | T31818 | – | – | – | OP357965 | OP357967 | – | Liu et al. (2025) |
| *Trichoderma dorothopsis* | CGMCC3.19672 T | MH624140 | – | – | MH647795 | MK850827 | – | Liu et al. (2025) |
| *Trichoderma dorothopsis* | CGMCC3.19673 | MH624143 | – | – | MH647798 | MK850830 | – | Liu et al. (2025) |
| *Trichoderma endophyticum* | BAFC 4372 | – | – | – | – | MH371397 | – | Liu et al. (2025) |
| *Trichoderma endophyticum* | CBS 130729 T | – | – | – | – | – | – | Liu et al. (2025) |
| *Trichoderma endophyticum* | H1-S-97 | – | – | – | MT822003 | MT821957 | – | Liu et al. (2025) |
| *Trichoderma egyptiacum* | YRBN1556 | – | – | – | PQ279243 | PQ151142 | – | Liu et al. (2025) |
| *Trichoderma egyptiacum* | YRBN1547 | – | – | – | PQ279240 | PQ151140 | – | Liu et al. (2025) |
| *Trichoderma egyptiacum* | YRBN1558 | – | – | – | PQ279242 | PQ151141 | – | Liu et al. (2025) |
| *Trichoderma evansii* | CBS 123079 T | EU883568 | – | – | EU883558 | EU883566 | – | Liu et al. (2025) |
| *Trichoderma fargesiae* | SICAU 25-0185 T | PV789477 | – | – | PV828320 | PV828328 | – | Liu et al. (2025) |
| *Trichoderma fargesiae* | SICAU 25-0186 | PV789478 | – | – | PV828321 | PV828329 | – | Liu et al. (2025) |
| *Trichoderma fomitopsis* | HKAS 26205 T | JF905628 | – | – | – | – | – | Liu et al. (2025) |
| *Trichoderma ganodermatis* | HMAS 248856 T | KY687939 | – | – | KY687995 | KY688060 | – | Liu et al. (2025) |
| *Trichoderma ganodermatis* | HMAS 248869 | KY687952 | – | – | KY688007 | KY688061 | – | Liu et al. (2025) |
| *Trichoderma gansuanum* | HMAS 279687 T | MT084412 | – | – | MT087287 | MT095060 | – | Liu et al. (2025) |
| *Trichoderma gongcheniae* | GDMCC 3.1011T | – | – | – | OR779489 | OR779518 | – | Liu et al. (2025) |
| *Trichoderma gongcheniae* | T33522 | – | – | – | OR779490 | OR779519 | – | Liu et al. (2025) |
| *Trichoderma graminicola* | GDMCC 3.1015 T | – | – | – | OR779494 | OR779521 | – | Liu et al. (2025) |
| *Trichoderma graminicola* | YNE00489 | – | – | – | OR779493 | OR779520 | – | Liu et al. (2025) |
| *Trichoderma graminis* | GDMCC 3.1013 T | – | – | – | OR779491 | OR779514 | – | Liu et al. (2025) |
| *Trichoderma graminis* | YNE00430 | – | – | – | OR779492 | OR779515 | – | Liu et al. (2025) |
| *Trichoderma guizhouense* | CBS 131803 T | JN191311 | – | – | JQ901400 | JN215484 | – | Liu et al. (2025) |
| *Trichoderma guizhouense* | PARC1025 | MT448971 | – | – | MT454144 | MT454128 | – | Liu et al. (2025) |
| *Trichoderma guizhouense* | PARC1026 | MT448972 | – | – | MT454145 | MT454129 | – | Liu et al. (2025) |
| *Trichoderma hailarense* | WT 17803 | MH606226 | – | – | MH606232 | MH606229 | – | Liu et al. (2025) |
| *Trichoderma hailarense* | WT 17901 T | MH287485 | – | – | MH287506 | MH287505 | – | Liu et al. (2025) |
| *Trichoderma hamatum* | COAD 2417 | – | – | – | MK044203 | MK044110 | – | Liu et al. (2025) |
| *Trichoderma hamatum* | COAD 2418 | – | – | – | MK044204 | MK044111 | – | Liu et al. (2025) |
| *Trichoderma hamatum* | DAOM 167057 T | EU280124 | – | – | KJ842139 | EU279965 | – | Liu et al. (2025) |
| *Trichoderma harzianum* | CBS 226.95 T | – | – | – | AF545549 | AF348101 | – | Liu et al. (2025) |
| *Trichoderma harzianum* | CGMCC 20739 | – | – | – | MZ603731 | MZ603732 | – | Liu et al. (2025) |
| *Trichoderma hipposiderocola* | KUMCC 21-0801 T | ON426899 | – | – | OR025896 | OR025978 | – | Liu et al. (2025) |
| *Trichoderma hipposiderocola* | KUMCC 21-0805 | ON426894 | – | – | OR025898 | OR025980 | – | Liu et al. (2025) |
| *Trichoderma hirsutum* | HMAS 248842 | KY687925 | – | – | KY687981 | KY688041 | – | Liu et al. (2025) |
| *Trichoderma hirsutum* | HMAS 248860 | KY687943 | – | – | KY687999 | KY688042 | – | Liu et al. (2025) |
| ***Trichoderma hongheense*** | **GMBCC2297 T** | **PZ669380** | **–** | **–** | **PZ867803** | **PZ871007** | – | **This study** |
| ***Trichoderma hongheense*** | **GMBCC2298** | **PZ669381** | **–** | **–** | **PZ867804** | **PZ871008** | – | **This study** |
| *Trichoderma hongkuii* | GDMCC 3.1017 T | – | – | – | OR779477 | OR779504 | – | Liu et al. (2025) |
| *Trichoderma hongkuii* | T32028 | – | – | – | OR779478 | OR779505 | – | Liu et al. (2025) |
| *Trichoderma hortense* | DAOM 230830 | AY605743 | – | – | KJ842180 | AY605786 | – | Liu et al. (2025) |
| *Trichoderma hortense* | GJS 04-70 | – | – | – | FJ442711 | FJ463352 | – | Liu et al. (2025) |
| *Trichoderma hydei* | GMBCC1136 T | PV061860 | – | – | PV448526 | PV448528 | – | Liu et al. (2025) |
| *Trichoderma hydei* | GMBCC1140 | PV061861 | – | – | PV448527 | PV448529 | – | Liu et al. (2025) |
| *Trichoderma inaequilaterale* | YMF 1.06203 T | MN977795 | – | – | MT052186 | MT070152 | – | Liu et al. (2025) |
| *Trichoderma inconspicuum* | YMF 1.04623 | MK795993 | – | – | MK795989 | MK795985 | – | Liu et al. (2025) |
| *Trichoderma insigne* | YMF 1.00207 T | MH113925 | – | – | MH155271 | MH177911 | – | Liu et al. (2025) |
| *Trichoderma insigne* | YMF 1.00272 | MH113927 | – | – | MH155274 | MH177913 | – | Liu et al. (2025) |
| *Trichoderma jaklitschii* | CP01-6 | – | – | – | MW480150 | MW480144 | – | Liu et al. (2025) |
| *Trichoderma jaklitschii* | CP61-2 T | – | – | – | MW480149 | MW480140 | – | Liu et al. (2025) |
| *Trichoderma koningii* | CBS 119500 | – | – | – | FJ860541 | KC285594 | – | Liu et al. (2025) |
| *Trichoderma koningii* | CBS 457.96 T | – | – | – | – | AF456909 | – | Liu et al. (2025) |
| *Trichoderma koningiopsis* | COAD 2405 | – | – | – | MK044185 | MK044092 | – | Liu et al. (2025) |
| *Trichoderma koningiopsis* | COAD 2502 | – | – | – | MK044190 | MK044097 | – | Liu et al. (2025) |
| *Trichoderma koningiopsis* | GJS 93-20 T | DQ313140 | – | – | EU241506 | DQ284966 | – | Liu et al. (2025) |
| *Trichoderma lentiforme* | CEN1429 | MK714910 | – | – | MK696828 | MK696668 | – | Liu et al. (2025) |
| *Trichoderma lentiforme* | H1-R-96 | – | – | – | MT822037 | MT821992 | – | Liu et al. (2025) |
| *Trichoderma lentinulae* | CGMCC 3.19848 | MN594470 | – | – | MN605868 | MN605879 | – | Liu et al. (2025) |
| *Trichoderma lentinulae* | HMAS 248256 T | MN594469 | – | – | MN605867 | MN605878 | – | Liu et al. (2025) |
| *Trichoderma lentissimum* | COAD 2399 T | – | – | – | MK044179 | MK044086 | – | Liu et al. (2025) |
| *Trichoderma lixii* | GJS 97-106 | – | – | – | FJ442771 | AF443939 | – | Liu et al. (2025) |
| *Trichoderma lixii* | GJS 97-96 T | NR_131264 | – | – | KJ665290 | AF443938 | – | Liu et al. (2025) |
| *Trichoderma longibrachiatum* | CBS 816.68 T | MH859229 | – | – | DQ087242 | EU401591 | – | Liu et al. (2025) |
| *Trichoderma longibrachiatum* | S328 | JQ685875 | – | – | JQ685883 | JQ685867 | – | Liu et al. (2025) |
| *Trichoderma longifialidicum* | LESF552 | KT278901 | – | – | KT278955 | KT279020 | – | Liu et al. (2025) |
| *Trichoderma longipile* | CBS 120953 | FJ860770 | – | – | FJ860542 | FJ860643 | – | Liu et al. (2025) |
| *Trichoderma longipile* | DAOM 177227 T | AY865630 | – | – | AF545550 | AY937430 | – | Liu et al. (2025) |
| *Trichoderma lycogaloides* | CBS 123493 T | – | – | – | KF134792 | KF134800 | – | Liu et al. (2025) |
| *Trichoderma macrochlamydospora* | JZBQT5Z1 T | – | – | – | ON649955 | ON649902 | – | Liu et al. (2025) |
| *Trichoderma macrochlamydospora* | JZBQT5Z2 | – | – | – | ON649956 | ON649903 | – | Liu et al. (2025) |
| *Trichoderma macrofasciculatum* | WT 37805 T | MH287487 | – | – | MH287493 | MH287509 | – | Liu et al. (2025) |
| *Trichoderma macrofasciculatum* | WT 37810 | MH287488 | – | – | MH287494 | MH287510 | – | Liu et al. (2025) |
| *Trichoderma mianyangense* | SICAU 25-0183 T | PV789475 | – | – | PV828322 | PV828330 | – | Liu et al. (2025) |
| *Trichoderma mianyangense* | SICAU 25-0184 | PV789476 | – | – | PV828323 | PV828331 | – | Liu et al. (2025) |
| ***Trichoderma mangshiense*** | **GMBCC2300 T** | **PZ669382** | **–** | **–** | **PZ867805** | **PZ871009** | – | **This study** |
| ***Trichoderma mangshiense*** | **GZCC 25-0718** | **PZ669383** | **–** | **–** | **PZ867806** | **PZ871010** | – | **This study** |
| ***Trichoderma neogongcheniae*** | **GZCC 25-0721 T** | **PZ669384** | **–** | **–** | **PZ867807** | **PZ871011** | – | **This study** |
| ***Trichoderma neogongcheniae*** | **GZCC 25-0722** | **PZ669385** | **–** | **–** | **PZ867808** | **PZ871012** | – | **This study** |
| ***Trichoderma neogongcheniae*** | **GMBCC2302** | **PZ669386** | **–** | **–** | **PZ867809** | **PZ871013** | – | **This study** |
| *Trichoderma neoguizhouense* | GDMCC 3.1012 T | – | – | – | OR779487 | OR779516 | – | Liu et al. (2025) |
| *Trichoderma neoguizhouense* | T33326 | – | – | – | OR779488 | OR779517 | – | Liu et al. (2025) |
| *Trichoderma neohongkuii* | GDMCC 3.1018 T | – | – | – | OR779481 | OR779508 | – | Liu et al. (2025) |
| *Trichoderma neohongkuii* | YNE00948 | – | – | – | OR779482 | OR779509 | – | Liu et al. (2025) |
| ***Trichoderma neopyramidale*** | **GMBCC2303 T** | **PZ669387** | **–** | **–** | **PZ867810** | **PZ871014** | – | **This study** |
| ***Trichoderma neopyramidale*** | **GZCC 25-0729** | **PZ669388** | **–** | **–** | **PZ867811** | **PZ871015** | – | **This study** |
| *Trichoderma neotropicale* | CBS 130633 T | HQ022407 | – | – | – | HQ022771 | – | Liu et al. (2025) |
| *Trichoderma neotropicale* | GJS 08-182 | – | – | – | – | MG822718 | – | Liu et al. (2025) |
| *Trichoderma neotropicale* | GJS 08-183 | – | – | – | – | MG822719 | – | Liu et al. (2025) |
| *Trichoderma nigricans* | CGMCC40314 T | – | – | – | OP357959 | OP357974 | – | Liu et al. (2025) |
| *Trichoderma nigricans* | T32794 | – | – | – | OP357960 | OP357975 | – | Liu et al. (2025) |
| *Trichoderma nobilis* | ZHKUCC24-0788 T | PP789101 | – | – | PP848460 | PP848444 | – | Liu et al. (2025) |
| *Trichoderma nobilis* | ZHKUCC24-0789 T | PP789102 | – | – | PP848461 | PP848445 | – | Liu et al. (2025) |
| *Trichoderma nordicum* | WT 13001 | MH287483 | – | – | MH287502 | MH287501 | – | Liu et al. (2025) |
| *Trichoderma nordicum* | WT 61001 T | MH287484 | – | – | MH287504 | MH287503 | – | Liu et al. (2025) |
| *Trichoderma notatum* | JZBQT1Z11 | – | – | – | OP832382 | OP832397 | – | Liu et al. (2025) |
| *Trichoderma notatum* | JZBQT1Z5 T | – | – | – | OP832381 | OP832396 | – | Liu et al. (2025) |
| ***Trichoderma nujiangense*** | **GMBCC2304 T** | **PZ669389** | **–** | **–** | **PZ867812** | **PZ871016** | – | **This study** |
| ***Trichoderma nujiangense*** | **GZCC 25-0733** | **PZ669390** | **–** | **–** | **PZ867813** | **PZ871017** | – | **This study** |
| ***Trichoderma obovatum*** | **GMBCC2473** | **PZ669391** | **–** | **–** | **PZ867814** | **PZ871018** | – | **This study** |
| *Trichoderma obovatum* | YMF 1.06211 | MN977803 | – | – | MT038432 | MT070144 | – | Liu et al. (2025) |
| *Trichoderma obovatum* | YMF 1.06212 | MN977804 | – | – | MT038433 | MT070143 | – | Liu et al. (2025) |
| *Trichoderma ochroleucum* | CBS 119502 T | FJ860793 | – | – | FJ860556 | FJ860659 | – | Liu et al. (2025) |
| *Trichoderma ovalisporum* | CBS 113299 T | AY380897 | – | – | FJ442742 | AY376037 | – | Liu et al. (2025) |
| *Trichoderma panacis* | SYPF 8050 T | MF565524 | – | – | MF565525 | MF565523 | – | Liu et al. (2025) |
| *Trichoderma paradensissimum* | CGMCC 3.24125 T | – | – | – | OP357962 | OP357968 | – | Liu et al. (2025) |
| *Trichoderma paradensissimum* | T31824 | – | – | – | OP357961 | OP357969 | – | Liu et al. (2025) |
| *Trichoderma parahamatum* | GDMCC 3.1020 T | – | – | – | OR779474 | OR779501 | – | Liu et al. (2025) |
| *Trichoderma parahamatum* | T30064 | – | – | – | OR779473 | OR779500 | – | Liu et al. (2025) |
| *Trichoderma parahongkuii* | GDMCC 3.1019 T | – | – | – | OR779476 | OR779503 | – | Liu et al. (2025) |
| *Trichoderma parahongkuii* | T31355 | – | – | – | OR779475 | OR779502 | – | Liu et al. (2025) |
| *Trichoderma paraobovatum* | CSC22A0600 T | OR817754 | – | – | OR813782 | OR813797 | – | Liu et al. (2025) |
| *Trichoderma parapeberdyi* | GDMCC 3.1016 T | – | – | – | OR779483 | OR779510 | – | Liu et al. (2025) |
| *Trichoderma parapeberdyi* | T32471 | – | – | – | OR779484 | OR779511 | – | Liu et al. (2025) |
| *Trichoderma parareesei* | COAD 2482 | – | – | – | MK044247 | MK044154 | – | Liu et al. (2025) |
| *Trichoderma parareesei* | COAD 2483 | – | – | – | MK044246 | MK044153 | – | Liu et al. (2025) |
| *Trichoderma paraviride* | YMF 1.04628 T | MK775514 | – | – | MK775513 | MK775508 | – | Liu et al. (2025) |
| *Trichoderma peberdyi* | CEN1389 | MK714863 | – | – | MK696783 | MK696621 | – | Liu et al. (2025) |
| *Trichoderma peberdyi* | CEN1426 T | MK714906 | – | – | MK696825 | MK696664 | – | Liu et al. (2025) |
| *Trichoderma peruvianum* | CP15-2 T | – | – | – | MW480153 | MW480145 | – | Liu et al. (2025) |
| *Trichoderma peruvianum* | CP15-9 | – | – | – | MW480154 | MW480146 | – | Liu et al. (2025) |
| *Trichoderma pholiotae* | JZBQH11 | – | – | – | ON649971 | ON649918 | – | Liu et al. (2025) |
| *Trichoderma pholiotae* | JZBQH12 T | ON653405 | – | – | ON649972 | ON649919 | – | Liu et al. (2025) |
| *Trichoderma phyllostachydis* | CBS 114071 T | FJ860809 | – | – | FJ860570 | FJ860673 | – | Liu et al. (2025) |
| *Trichoderma pluripenicillatum* | YMF 1.06198 T | MN977788 | – | – | MT070160 | MT070159 | – | Liu et al. (2025) |
| *Trichoderma propepolypori* | YMF 1.06199 | MN977790 | – | – | MT052182 | MT070157 | – | Liu et al. (2025) |
| *Trichoderma propepolypori* | YMF 1.06224 T | MN977789 | – | – | MT052181 | MT070158 | – | Liu et al. (2025) |
| *Trichoderma pseudoasiaticum* | YMF 1.06200 T | MN977792 | – | – | MT052183 | MT070155 | – | Liu et al. (2025) |
| *Trichoderma pseudoasperelloides* | YMF 1.00152 | MH262581 | – | – | MH262574 | MH236491 | – | Liu et al. (2025) |
| *Trichoderma pseudoasperelloides* | YMF 1.04629 T | MH383059 | – | – | MK775509 | MK775504 | – | Liu et al. (2025) |
| *Trichoderma pseudopyramidale* | COAD 2419 | – | – | – | MK044206 | MK044113 | – | Liu et al. (2025) |
| *Trichoderma pseudopyramidale* | COAD 2426 T | – | – | – | MK044224 | MK044131 | – | Liu et al. (2025) |
| *Trichoderma pseudopyramidale* | COAD 2433 | – | – | – | MK044250 | MK044157 | – | Liu et al. (2025) |
| *Trichoderma pseudopyramidale* | COAD 2434 | – | – | – | MK044251 | MK044158 | – | Liu et al. (2025) |
| *Trichoderma pseudopyramidale* | COAD 2506 | – | – | – | MK044207 | MK044114 | – | Liu et al. (2025) |
| *Trichoderma pyramidale* | CBS 135574 T | – | – | – | KJ665334 | KJ665699 | – | Liu et al. (2025) |
| *Trichoderma pyramidale* | T20 | KX632513 | – | – | KX632570 | KX632627 | – | Liu et al. (2025) |
| *Trichoderma reticulatae* | ZHKUCC24-0776 T | PP789089 | – | – | PP848448 | PP848432 | – | Liu et al. (2025) |
| *Trichoderma reticulatae* | ZHKUCC24-0777 | PP789090 | – | – | PP848449 | PP848433 | – | Liu et al. (2025) |
| *Trichoderma rhinolophicola* | KUMCC 21-0813 T | OP425803 | – | – | – | OR025989 | – | Liu et al. (2025) |
| *Trichoderma rhinolophicola* | KUMCC 21-0814 | OP425804 | – | – | – | OR025990 | – | Liu et al. (2025) |
| *Trichoderma rifaii* | DIS 337F T | FJ442621 | – | – | FJ442720 | FJ463321 | – | Liu et al. (2025) |
| *Trichoderma rifaii* | J2-2CR | – | – | – | – | MK644113 | – | Liu et al. (2025) |
| *Trichoderma rossicum* | DAOM 230010 T | DQ083024 | – | – | HQ400747 | AY937424 | – | Liu et al. (2025) |
| *Trichoderma rugulosum* | SFC20180301-001 T | MH050353 | – | – | MH025986 | MH025984 | – | Liu et al. (2025) |
| *Trichoderma rugulosum* | CC2-7 | MW376897 | – | – | MW407161 | MW588806 | – | Liu et al. (2025) |
| *Trichoderma scorpioideum* | YMF 1.04616 T | MK795992 | – | – | MK795988 | MK795984 | – | Liu et al. (2025) |
| *Trichoderma shaanxiensi* | GDMCC 3.1014 T | – | – | – | OR779486 | OR779513 | – | Liu et al. (2025) |
| *Trichoderma shaanxiensi* | T31999 | – | – | – | OR779485 | OR779512 | – | Liu et al. (2025) |
| *Trichoderma shangrilaense* | WT 34004 T | MH287489 | – | – | MH287496 | MH287495 | – | Liu et al. (2025) |
| *Trichoderma shangrilaense* | WT 40502 | MH606224 | – | – | MH606230 | MH606227 | – | Liu et al. (2025) |
| *Trichoderma shennongjianum* | HMAS 245009 T | KX060605 | – | – | KT735259 | KT735253 | – | Liu et al. (2025) |
| *Trichoderma simile* | YMF 1.06201 T | MN977793 | – | – | MT052184 | MT070154 | – | Liu et al. (2025) |
| *Trichoderma simile* | YMF 1.06202 | MN977794 | – | – | MT052185 | MT070153 | – | Liu et al. (2025) |
| *Trichoderma spirale* | COAD 2404 | – | – | – | MK044184 | MK044091 | – | Liu et al. (2025) |
| *Trichoderma spirale* | COAD 2408 | – | – | – | MK044189 | MK044096 | – | Liu et al. (2025) |
| *Trichoderma stramineum* | GJS 02-84 T | AY737765 | – | – | AY391945 | AY737746 | – | Liu et al. (2025) |
| *Trichoderma stramineum* | TAMA 0425 | AB856609 | – | – | AB856748 | AB856675 | – | Liu et al. (2025) |
| *Trichoderma strictipile* | DAOM 172827 T | – | – | – | KJ842162 | AY937451 | – | Liu et al. (2025) |
| *Trichoderma stromaticum* | DAOM 231100 T | – | – | – | KJ842192 | KJ871129 | – | Liu et al. (2025) |
| *Trichoderma subazureum* | YMF 1.06207 T | MN977799 | – | – | MT052190 | MT070148 | – | Liu et al. (2025) |
| *Trichoderma subuliforme* | YMF 1.06204 | MN977796 | – | – | MT052187 | MT070151 | – | Liu et al. (2025) |
| *Trichoderma subuliforme* | YMF 1.06205 | MN977797 | – | – | MT052188 | MT070150 | – | Liu et al. (2025) |
| *Trichoderma subvermifimicola* | JZBQT4Z1 T | ON653398 | – | – | ON649952 | ON649899 | – | Liu et al. (2025) |
| *Trichoderma subvermifimicola* | JZBQT4Z2 | ON653399 | – | – | ON649953 | ON649900 | – | Liu et al. (2025) |
| *Trichoderma sulawesense* | GJS85-228 T | AY737753 | – | – | AY391954 | AY737730 | – | Liu et al. (2025) |
| *Trichoderma supraverticillatum* | YMF 1.06208 T | MN977800 | – | – | MT052191 | MT070147 | – | Liu et al. (2025) |
| *Trichoderma syagri* | BAFC 4357 T | – | – | – | – | MG822711 | – | Liu et al. (2025) |
| *Trichoderma syagri* | BAFC 4371 | – | – | – | – | MG822710 | – | Liu et al. (2025) |
| *Trichoderma tawa* | G.J.S. 97-174 T | AY737756 | – | – | AY391956 | AY392004 | – | Liu et al. (2025) |
| *Trichoderma tawa* | G.J.S. 02-79 | – | – | – | AY391955 | AY392003 | – | Liu et al. (2025) |
| *Trichoderma theobromicola* | COAD 2406 | – | – | – | MK044186 | MK044093 | – | Liu et al. (2025) |
| *Trichoderma theobromicola* | COAD 2407 | – | – | – | MK044187 | MK044094 | – | Liu et al. (2025) |
| *Trichoderma tibetense* | HMAS 245010 T | KX060607 | – | – | KT735261 | KT735254 | – | Liu et al. (2025) |
| *Trichoderma tibetica* | YMF 1.05583 T | MK779177 | – | – | MK779178 | MK779179 | – | Liu et al. (2025) |
| *Trichoderma tomentosum* | DAOM 178713a T | DQ085432 | – | – | AF545557 | EU279969 | – | Liu et al. (2025) |
| *Trichoderma tongzhouense* | JZBQT1Z1 T | – | – | – | ON649945 | ON649892 | – | Liu et al. (2025) |
| *Trichoderma tongzhouense* | JZBQT1Z2 | – | – | – | ON649946 | ON649893 | – | Liu et al. (2025) |
| *Trichoderma tropicosinense* | HMAS 252546 T | KF923302 | – | – | KF923313 | KF923286 | – | Liu et al. (2025) |
| *Trichoderma uncinatum* | YMF 1.04622 | MK795994 | – | – | MK795990 | MK795986 | – | Liu et al. (2025) |
| *Trichoderma vadicola* | WT 10708 T | MH287491 | – | – | MH287511 | MH287499 | – | Liu et al. (2025) |
| *Trichoderma vadicola* | WT 32801 | MH606225 | – | – | MH606231 | MH606228 | – | Liu et al. (2025) |
| *Trichoderma vermifimicola* | CGMCC 3.19850 | MN594472 | – | – | MN605870 | MN605881 | – | Liu et al. (2025) |
| *Trichoderma vermifimicola* | HMAS 248255 T | MN594473 | – | – | MN605871 | MN605882 | – | Liu et al. (2025) |
| *Trichoderma virens* | COAD 2400 | – | – | – | MK044180 | MK044087 | – | Liu et al. (2025) |
| *Trichoderma virens* | GJS 01-287 | DQ083023 | – | – | EU341804 | AY750894 | – | Liu et al. (2025) |
| *Trichoderma virens* | V-1 | KF144616 | – | – | KF574111 | KF668098 | – | Liu et al. (2025) |
| *Trichoderma virens* | V-2 | KF144617 | – | – | KF574108 | KF668099 | – | Liu et al. (2025) |
| [*Trichoderma wujiangense*](https://www.indexfungorum.org/Names/NamesRecord.asp?RecordID=902150) | ZHKUCC24-0783 T | PP789096 | – | – | PP848455 | PP848439 | – | Liu et al. (2025) |
| [*Trichoderma wujiangense*](https://www.indexfungorum.org/Names/NamesRecord.asp?RecordID=902150) | ZHKUCC24-0784 | PP789097 | – | – | PP848456 | PP848440 | – | Liu et al. (2025) |
| [*Trichoderma wujiangense*](https://www.indexfungorum.org/Names/NamesRecord.asp?RecordID=902150) | ZHKUCC24-0785 | PP789098 | – | – | PP848457 | PP848441 | – | Liu et al. (2025) |
| *Trichoderma xishuangbannaense* | KUMCC 21-0815 T | OP425805 | – | – | OR025907 | OR025991 | – | Liu et al. (2025) |
| *Trichoderma xishuangbannaense* | KUMCC 21-0816 | OP425806 | – | – | OR025908 | OR025992 | – | Liu et al. (2025) |
| *Trichoderma xixiacum* | CGMCC 3.19698 | MN594477 | – | – | MN605875 | MN605886 | – | Liu et al. (2025) |
| *Trichoderma xixiacum* | HMAS 248253 T | MN594476 | – | – | MN605874 | MN605885 | – | Liu et al. (2025) |
| *Trichoderma yaanense* | SICAU 25-0181 T | PV789473 | – | – | PV828318 | PV828326 | – | Liu et al. (2025) |
| *Trichoderma yaanense* | SICAU 25-0182 | PV789474 | – | – | PV828319 | PV828327 | – | Liu et al. (2025) |
| *Trichoderma yilanense* | BCRC:18F0052 T | PP786515 | – | – | PP779116 | PP779115 | – | Liu et al. (2025) |
| ***Trichoderma yingjiangense*** | **GZCC 25-0723 T** | **PZ669392** | **–** | **–** | **PZ867815** | **PZ871019** | **–** | **This study** |
| ***Trichoderma yingjiangense*** | **GZCC 25-0724** | **PZ669393** | **–** | **–** | **PZ867816** | **PZ871020** | **–** | **This study** |
| *Trichoderma zelobreve* | CGMCC 3.19696 | MN594475 | – | – | MN605873 | MN605884 | – | Liu et al. (2025) |
| *Trichoderma zelobreve* | HMAS 248254 T | MN594474 | – | – | MN605872 | MN605883 | – | Liu et al. (2025) |
| *Trichoderma zeloharzianum* | YMF 1.00268 T | MH113932 | – | – | MH158996 | MH183181 | – | Liu et al. (2025) |
| *Trichoderma zhuangii* | CGMCC 3.28903 T | PV650642 | – | – | PV664059 | PV664060 | – | Liu et al. (2025) |
| *Umbelopsis changbaiensis* | Um 027 | KC489483 | KF727446 | – | – | – | – | Zhao et al. (2023a) |
| *Umbelopsis changbaiensis* | Um 028 | KC489484 | KF727447 | – | – | – | – | Zhao et al. (2023a) |
| *Wardomycopsis humicola* | CBS 487.66 T | LM652497 | LM652554 | – | – | – | – | Sandoval-Denis et al. (2016) |
| *Wardomycopsis inopinata* | FMR 10305 | LM652498 | LM652555 | – | – | – | – | Sandoval-Denis et al. (2016) |
| *Xanthiomyces spainosus* | CBS 789.71 | MH860357 | MZ351429 | MZ343034 | MZ342995 | – | – | Wang et al. (2022) |
| *Xanthiomyces spainosus* | CBS 796.83 | MZ334724 | MZ351430 | MZ343035 | MZ342996 | – | – | Wang et al. (2022) |
| *Xylaria hypoxylon* | CBS12260 | KY610407 | KY610495 | KX271279 | KY624231 | – | – | Wongkanoun et al. (2020) |

T indicates the ex-type; – data unavailable; Newly generated sequences are in bold.

| **Class** | **Order** | **Family** | **Current name** | **Species** | **Host/substrates** | **Location** | **NF** | **References** |
| --- | --- | --- | --- | --- | --- | --- | --- | --- |
| **Fungi** |  |  |  |  |  |  |  |  |
| **Ascomycota** |  |  |  |  |  |  |  |  |
| *Arthoniomycetes* | *Lichenostigmatales* | *Phaeococcomycetaceae* | *Phaeococcomyces nigricans* | *Phaeococcomyces nigricans* | Unreported | Canada | 1 | Connell and Staudigel (2013) |
| *Ascomycetes* |  |  | Ascomycetes sp. | Ascomycete sp. | Soil-sediment, unreported | Canada, UK, USA | 3 | Vanderwolf et al. (2013a) |
| *Coelomycetes* |  |  | Coelomycete sp. | Coelomycete sp. |  | Canada | 1 | Vanderwolf et al. (2013a) |
| *Dothideomycetes* | *Botryosphaeriales* | *Aplosporellaceae* | Aplosporella sp. | Aplosporella sp. | Air | Brazil | 1 | Cunha et al. (2020) |
| *Dothideomycetes* | *Botryosphaeriales* | *Botryosphaeriaceae* | *Lasiodiplodia* sp. | *Lasiodiplodia* sp. | Rock | Thailand | 1 | Suetrong et al. (2023) |
| *Dothideomycetes* | *Botryosphaeriales* | *Botryosphaeriaceae* | *Lasiodiplodia iraniensis* | *Lasiodiplodia iraniensis* | Air, sediment | Brazil | 1 | Alves et al. (2022) |
| *Dothideomycetes* | *Botryosphaeriales* | *Botryosphaeriaceae* | *Microdiplodia* sp. | *Microdiplodia* sp. | Unreported | China | 1 | Zhang (2019) |
| *Dothideomycetes* | *Botryosphaeriales* | *Botryosphaeriaceae* | *Neofusicoccum ribis* | *Neofusicoccum ribis* | Bat | USA | 1 | Zhang et al. (2014) |
| *Dothideomycetes* | *Botryosphaeriales* | *Endomelanconiopsidaceae* | *Endomelanconiopsis sp.* | *Endomelanconiopsis sp.* | Rock | Thailand | 1 | Suetrong et al. (2023) |
| *Dothideomycetes* | *Capnodiales* | *Capnodiaceae* | *Capnodium* sp. | *Capnodium* sp. | Unreported | China | 1 | Zhang et al. (2017) |
| *Dothideomycetes* | *Capnodiales* | *Capnodiaceae* | *Tripospermum* spp. | *Tripospermum* spp. | Soil-sediment, water, air | Puerto Rico, Spain | 2 | Vanderwolf et al. (2013a) |
| *Dothideomycetes* | *Capnodiales* | *Incertae sedis* | *Toxicocladosporium irritans* | *Toxicocladosporium irritans* | Insects | Brazil | 1 | Vanderwolf et al. (2013a) |
| *Dothideomycetes* | *Cladosporiales* | *Cladosporiaceae* | *Cladosporium allicinum* | *Cladosporium allicinum* | Air | Romania | 1 | Nováková et al. (2018) |
| *Dothideomycetes* | *Cladosporiales* | *Cladosporiaceae* | *Cladosporium allicinum* | Cladosporium cf. bruhnei | Air | Romania | 1 | Nováková et al. 2018 |
| *Dothideomycetes* | *Cladosporiales* | *Cladosporiaceae* | *Cladosporium angustisporum* | *Cladosporium angustisporum* | Air | Romania | 1 | Nováková et al. (2018) |
| *Dothideomycetes* | *Cladosporiales* | *Cladosporiaceae* | *Cladosporium anthropophilum* | *Cladosporium anthropophilum* | Air | China | 1 | Jiang et al. (2017a, b) |
| *Dothideomycetes* | *Cladosporiales* | *Cladosporiaceae* | *Cladosporium asperulatum* | *Cladosporium asperulatum* | Unreported | China | 1 | Pereira et al. (2022) |
| *Dothideomycetes* | *Cladosporiales* | *Cladosporiaceae* | *Cladosporium austrohemisphaericum* | *Cladosporium austrohemisphaericum* | From air | Brazil | 1 | Pereira et al. (2022) |
| *Dothideomycetes* | *Cladosporiales* | *Cladosporiaceae* | *Cladosporium cladosporioides* | *Cladosporium cladosporioides* | Soil-sediment, water, cave wall, bat guano, air, dung, frass (not bat); wood, other, insects | Australia, Belgium, Brazil, Canada, China, Czech Republic, France, Israel, Italy, Malaysia, Poland, Puerto Rico, Russia, Slovakia, Slovenia, South Africa, Spain, Ukraine, USA | 19 | Vanderwolf et al. (2013a); Taylor et al. (2014); Jiang et al. (2017); Wasti et al. (2021) |
| *Dothideomycetes* | *Cladosporiales* | *Cladosporiaceae* | Cladosporium cladosporioides complex | Cladosporium cladosporioides complex | Air | Poland | 1 | Kokurewicz et al. (2016) |
| *Dothideomycetes* | *Cladosporiales* | *Cladosporiaceae* | *Cladosporium colombiae* | *Cladosporium colombiae* | rock surface, unreported | China | 1 | Pereira et al. (2022) |
| *Dothideomycetes* | *Cladosporiales* | *Cladosporiaceae* | *Cladosporium cucumerinum* | *Cladosporium cucumerinum* | Air | Spain | 1 | Vanderwolf et al. (2013a) |
| *Dothideomycetes* | *Cladosporiales* | *Cladosporiaceae* | *Cladosporium diamantinense* | *Cladosporium diamantinense* | Air | Brazil | 1 | Dutra et al. (2023) |
| *Dothideomycetes* | *Cladosporiales* | *Cladosporiaceae* | *Cladosporium grevilleae* | *Cladosporium grevilleae* | Unreported | Antarctica | 1 | Connell and Staudigel (2013); Zhang et al. (2021) |
| *Dothideomycetes* | *Cladosporiales* | *Cladosporiaceae* | *Cladosporium halotolerans* | *Cladosporium halotolerans* | Cave wall, Unreported | China | 1 | Zhang et al. (2021); Ma et al. (2025), |
| *Dothideomycetes* | *Cladosporiales* | *Cladosporiaceae* | *Cladosporium herbarum* | *Cladosporium herbarum* | Air, dung, frass (not bat); bat guano, soil-sediment, insects, wood, water, other, cave wall | Bahamas, Belgium, Brazil, Czech Republic, Israel, Italy, Poland, Puerto Rico, Romania, Slovakia, Slovenia, Ukraine | 12 | Taylor et al. (2013); Vanderwolf et al. (2013a) |
| *Dothideomycetes* | *Cladosporiales* | *Cladosporiaceae* | Cladosporium herbarum complex | Cladosporium herbarum complex | Air | Poland | 1 | Kokurewicz et al. (2016) |
| *Dothideomycetes* | *Cladosporiales* | *Cladosporiaceae* | *Cladosporium lebrasiae* | *Cladosporium lebrasiae* | Air | Spain | 1 | Savković et al. (2025) |
| *Dothideomycetes* | *Cladosporiales* | *Cladosporiaceae* | *Cladosporium linicola* | *Cladosporium linicola* | soil-sediment | Russia | 1 | Vanderwolf et al. (2013a) |
| *Dothideomycetes* | *Cladosporiales* | *Cladosporiaceae* | *Cladosporium macrocarpum* | *Cladosporium macrocarpum* | Wood, , air, other, cave wall, water | Belgium, Czech Republic, Israel, Poland, Romania, Slovakia | 4 | Vanderwolf et al. (2013a); Ogórek et al. (2018); Nováková et al. (2018); Ogórek (2018b) |
| *Dothideomycetes* | *Cladosporiales* | *Cladosporiaceae* | *Cladosporium oxysporum* | *Cladosporium oxysporum* | Air, bat guano, soil-sediment | Brazil, China, Puerto Rico, Romania, Serbia | 5 | Vanderwolf et al. (2013a), Nováková et al. (2018); Alves et al. (2022) |
| *Dothideomycetes* | *Cladosporiales* | *Cladosporiaceae* | *Cladosporium paracladosporioides* | *Cladosporium paracladosporioides* | underwater sediment of Airbell II, microbial mat (Airbell II); Air, cave sediment, rock | Poland, Romania | 2 | Nováková et al. (2018) |
| *Dothideomycetes* | *Cladosporiales* | *Cladosporiaceae* | *Cladosporium parahalotolerans* | *Cladosporium parahalotolerans* | From air | Brazil | 1 | Pereira et al. (2022) |
| *Dothideomycetes* | *Cladosporiales* | *Cladosporiaceae* | *Cladosporium perangustum* | *Cladosporium perangustum* | Cave wall, limestone | China | 1 | Jiang et al. (2017a, b) |
| *Dothideomycetes* | *Cladosporiales* | *Cladosporiaceae* | *Cladosporium pseudocladosporioides* | *Cladosporium pseudocladosporioides* | Unreported | Algeria | 1 | Belyagoubi et al. (2018) |
| *Dothideomycetes* | *Cladosporiales* | *Cladosporiaceae* | *Cladosporium puris* | *Cladosporium puris* | From air | Brazil | 1 | Pereira et al. (2022) |
| *Dothideomycetes* | *Cladosporiales* | *Cladosporiaceae* | *Cladosporium rectoides* | *Cladosporium rectoides* | Air | China | 1 | Jiang et al. (2017) |
| *Dothideomycetes* | *Cladosporiales* | *Cladosporiaceae* | *Cladosporium scabrellum* | *Cladosporium scabrellum* | Limestone | China | 1 | Jiang et al. (2017) |
| *Dothideomycetes* | *Cladosporiales* | *Cladosporiaceae* | Cladosporium sp. 1 C. sphaerospermum complex | Cladosporium sp. 1 C. sphaerospermum complex | Air | Brazil | 1 | Cunha et al. (2020) |
| *Dothideomycetes* | *Cladosporiales* | *Cladosporiaceae* | Cladosporium sp. 2 C. sphaerospermum complex | Cladosporium sp. 2 C. sphaerospermum complex | Bat | Brazil | 1 | Cunha et al. (2020) |
| *Dothideomycetes* | *Cladosporiales* | *Cladosporiaceae* | Cladosporium sp. 3 C. sphaerospermum complex | Cladosporium sp. 3 C. sphaerospermum complex | Air, bat | Brazil | 1 | Cunha et al. (2020) |
| *Dothideomycetes* | *Cladosporiales* | *Cladosporiaceae* | *Cladosporium speluncae* | *Cladosporium speluncae* | Soil | Brazil | 1 | Dutra et al. (2023) |
| *Dothideomycetes* | *Cladosporiales* | *Cladosporiaceae* | *Cladosporium sphaerospermum* | *Cladosporium sphaerospermum* | Air, cave sediment, insects, underwater sediment of Airbell II, bat guano, insect | Brazil, China, Czech Republic, Italy, Puerto Rico, Romania, Slovakia, Spain | 8 | Vanderwolf et al. (2013a); Nováková et al. (2018); Zhang et al. (2021) |
| *Dothideomycetes* | *Cladosporiales* | *Cladosporiaceae* | *Cladosporium spongiosum* | *Cladosporium spongiosum* | Cave wall | Israel | 1 | Vanderwolf et al. (2013a) |
| *Dothideomycetes* | *Cladosporiales* | *Cladosporiaceae* | Cladosporium spp. | Cladosporium spp. | Air, , unreported, insects, cave wall, bat guano, wood, soil-sediment, other, water | Australia, Belgium, Canada, China, Czech Republic, France, Ireland, Israel, Italy, Japan, Puerto Rico, Slovakia, Spain, Ukraine, UK, USA | 16 | Wang et al. (2010); Vanderwolf et al. (2013, 2015, 2019); Jiang et al. (2017); Zhang et al. (2021); Suetrong et al. (2023); Ma et al. (2025) |
| *Dothideomycetes* | *Cladosporiales* | *Cladosporiaceae* | *Cladosporium subuliforme* | *Cladosporium subuliforme* | Sediment | Brazil, China | 2 | Alves et al. (2022); Pereira et al. (2022) |
| *Dothideomycetes* | *Cladosporiales* | *Cladosporiaceae* | *Cladosporium tenuissimum* | *Cladosporium tenuissimum* | Air, limestone, sediment | Brazil, China, Slovakia | 3 | Vanderwolf et al. (2013a); Jiang et al. (2017); Alves et al. (2022) |
| *Dothideomycetes* | *Cladosporiales* | *Cladosporiaceae* | *Cladosporium uredinicola* | *Cladosporium uredinicola* | Rock | China, Poland | 2 | Man et al. (2015); Man (2017) |
| *Dothideomycetes* | *Cladosporiales* | *Cladosporiaceae* | *Cladosporium variabile* | *Cladosporium variabile* | Air, cave sediment, cave wall, underwater sediment of Airbell II, microbial mat (Airbell II) | Romania | 1 | Mitova et al. (2017); Nováková et al. (2018) |
| *Dothideomycetes* | *Cladosporiales* | *Cladosporiaceae* | *Cladosporium xanthochromaticum* | *Cladosporium xanthochromaticum* | Air | Brazil | 1 | Alves et al. (2022) |
| *Dothideomycetes* | *Cladosporiales* | *Cladosporiaceae* | *Hormodendrum* spp. | *Hormodendrum* spp. | Soil-sediment, cave wall | Russia, Venezuela | 2 | Vanderwolf et al. (2013a) |
| *Dothideomycetes* | *Dothideales* | *Dothioraceae* | *Sydowia polyspora* | *Sydowia polyspora* | Air | France, Poland | 2 | Pusz et al. (2017) |
| *Dothideomycetes* | *Dothideales* | *Saccotheciaceae* | *Aureobasidium pullulans* | *Aureobasidium pullulans* | Unreported, soil-sediment, air, insects, other | Argentina, Bahamas, Belgium, Czech Republic, France, Italy, Poland, Russia, South Africa, Spain, USA | 11 | Vanderwolf et al. (2013a); Zhang et al. (2021) |
| *Dothideomycetes* | *Dothideales* | *Saccotheciaceae* | *Aureobasidium* sp. | *Aureobasidium* sp. | Unreported, cave wall, air | Czech Republic, France, UK, USA | 4 | Vanderwolf et al. (2013a) |
| *Dothideomycetes* | *Hysteriales* | *Hysteriaceae* | *Rhytidhysteron rufulum* | *Rhytidhysteron rufulum* | Unreported | China | 1 | Zhang et al. (2021) |
| *Dothideomycetes* | *Incertae sedis* | *Eremomycetaceae* | *Arthrographis kalrae* | *Arthrographis kalrae* | Unreported | China | 1 | Zhang et al. (2021) |
| *Dothideomycetes* | *Incertae sedis* | *Eremomycetaceae* | *Arthrographis* sp. | *Arthrographis nycteribiae* | Insects | Slovakia | 1 | Vanderwolf et al. (2013a) |
| *Dothideomycetes* | *Incertae sedis* | *Eremomycetaceae* | *Arthrographis* sp. | *Arthrographis* sp. | Soil-sediment | Canada, China, France, USA | 4 | Vanderwolf et al. (2013a) |
| *Dothideomycetes* | *Incertae sedis* | *Eremomycetaceae* | *Eremomyces sp.* | *Eremomyces* sp. | Unreported | Canada | 1 | Vanderwolf et al. (2013a) |
| *Dothideomycetes* | *Incertae sedis* | *Incertae sedis* | *Brachyconidiella monilispora* | *Brachyconidiella monilispora* | Soil | Romania | 1 | Brad et al. (2018) |
| *Dothideomycetes* | *Incertae sedis* | *Incertae sedis* | *Cryomyces* sp. | *Cryomyces* sp. | Bats | Canada | 1 | Vanderwolf et al. (2015) |
| *Dothideomycetes* | *Incertae sedis* | *Incertae sedis* | *Sporidesmium atrum* | *Sporidesmium atrum* | Unreported | France | 1 | Vanderwolf et al. (2013a) |
| *Dothideomycetes* | *Incertae sedis* | *Incertae sedis* | *Tremateia arunicola* | *Tremateia arundicola* | Unreported | China | 1 | Zhang et al. (2021) |
| *Dothideomycetes* | *Incertae sedis* | *Incertae sedis* | *Tremateia chromolaenae* | *Tremateia chromolaenae* | Air | Spain | 1 | Vanderwolf et al. (2013a) |
| *Dothideomycetes* | *Incertae sedis* | *Polystomellaceae* | *Dothidella ulmi* | *Dothidella ulmi* | Unreported | China | 1 | Zhang et al. (2021) |
| *Dothideomycetes* | *Incertae sedis* | *Testudinaceae* | *Lepidosphaeria* sp. | *Lepidosphaeria* sp. | Unreported | China | 1 | Zhang et al. (2021) |
| *Dothideomycetes* | *Jahnulales* | *Aliquandostipitaceae* | *Brachiosphaera jamaicensis* | *Brachiosphaera jamaicensis* | Soil-sediment | Puerto Rico | 1 | Vanderwolf et al. (2013a) |
| *Dothideomycetes* | *Microthyriales* | *Microthyriaceae* | *Paramicrothyrium* sp. | *Paramicrothyrium* sp. | Unreported | China | 1 | Zhang et al. (2021) |
| *Dothideomycetes* | *Mycosphaerellales* | *Mycosphaerellaceae* | Asperisporium spp. | Asperisporium spp. | Air | China, Spain | 2 | Vanderwolf et al. (2013a); Zhang et al. (2021) |
| *Dothideomycetes* | *Mycosphaerellales* | *Mycosphaerellaceae* | *Globoramichloridium indicum* | *Ramichloridium indicum* | Air | Czech Republic | 1 | Vanderwolf et al. (2013a) |
| *Dothideomycetes* | *Mycosphaerellales* | *Mycosphaerellaceae* | *Mycosphaerella polygoni-cuspidati* | *Mycosphaerella polygoni-cuspidati* | Unreported | China | 1 | Zhang (2019) |
| *Dothideomycetes* | *Mycosphaerellales* | *Mycosphaerellaceae* | *Mycosphaerella* sp. | *Mycosphaerella* sp. | Unreported | China | 1 | Zhang (2019) |
| *Dothideomycetes* | *Mycosphaerellales* | *Mycosphaerellaceae* | [Mycosphaerellaceae](https://www.indexfungorum.org/Names/families.asp?FamilyName=Mycosphaerellaceae) | *Zasmidium syzygii* | Unreported | China | 1 | Zhang (2019) |
| *Dothideomycetes* | *Mycosphaerellales* | *Mycosphaerellaceae* | *Pallidocercospora heimioides* | *Pallidocercospora heimioides* | Unreported | China, Sumatera | 2 | Zhang et al. (2021) |
| *Dothideomycetes* | *Mycosphaerellales* | *Mycosphaerellaceae* | *Passalora* sp. | *Passalora* sp. | Air | Thailand | 1 | Suetrong et al. (2023) |
| *Dothideomycetes* | *Mycosphaerellales* | *Mycosphaerellaceae* | Polythrincium sp. | Polythrincium sp. | Air | Spain | 1 | Vanderwolf et al. (2013a) |
| *Dothideomycetes* | *Mycosphaerellales* | *Mycosphaerellaceae* | *Pseudocercospora* sp. | *Pseudocercospora* sp. | Organic litter, | Thailand | 1 | Suetrong et al. (2023) |
| *Dothideomycetes* | *Mycosphaerellales* | *Mycosphaerellaceae* | *Pseudocercosporella fraxini* | *Pseudocercosporella fraxini* | Unreported | China | 1 | Pereira et al. (2022) |
| *Dothideomycetes* | *Mycosphaerellales* | *Mycosphaerellaceae* | Pseudocercosporella spp. | Pseudocercosporella spp. | Air, soil-sediment, slate precipitate | China, Germany, USA | 3 | Vanderwolf et al. (2013a); Jiang et al. (2017); Burow et al. (2019) |
| *Dothideomycetes* | *Mycosphaerellales* | *Mycosphaerellaceae* | *Ramichloridium* spp. | *Ramichloridium* spp. | Soil-sediment | China, Slovakia | 2 | Vanderwolf et al. (2013a) |
| *Dothideomycetes* | *Mycosphaerellales* | *Mycosphaerellaceae* | *Septoria arundinacea* | *Septoria arundinacea* | Unreported | China | 1 | Zhang et al. (2021) |
| *Dothideomycetes* | *Mycosphaerellales* | *Mycosphaerellaceae* | *Septoria* spp. | *Septoria* spp. | Air | China, Spain | 2 | Vanderwolf et al. (2013a) |
| *Dothideomycetes* | *Mycosphaerellales* | [*Mycosphaerellaceae*](https://www.indexfungorum.org/Names/families.asp?FamilyName=Mycosphaerellaceae) | *Zasmidium cellare* | *Zasmidium cellare* | Unreported | USA | 1 | Vanderwolf et al. (2013a) |
| *Dothideomycetes* | *Mycosphaerellales* | *Teratosphaeriaceae* | *Acidomyces acidothermus* | *Acidomyces acidophilus* | Perennial ice, snottite - accidophylic biofilm - gypsum crystal surfaces | Romania, Russia | 2 | Brad et al. (2018); Zhang et al. (2021) |
| *Dothideomycetes* | *Mycosphaerellales* | *Teratosphaeriaceae* | *Acrodontium crateriforme* | *Acrodontium cf. crateriforme* | Unreported | Canada | 1 | Vanderwolf et al. (2013a) |
| *Dothideomycetes* | *Mycosphaerellales* | *Teratosphaeriaceae* | *Acrodontium crateriforme* | *Acrodontium crateriforme* | Air | Canada, China | 2 | Vanderwolf et al. (2013a); Zhang et al. (2017,2021) |
| *Dothideomycetes* | *Mycosphaerellales* | *Teratosphaeriaceae* | *Acrodontium salmoneum* | *Acrodontium salmoneum* | Air | Spain | 1 | Savković et al. (2025) |
| *Dothideomycetes* | *Mycosphaerellales* | *Teratosphaeriaceae* | *Acrodontium* sp. | *Acrodontium* sp. | Air | Slovakia | 1 | Vanderwolf et al. (2013a) |
| *Dothideomycetes* | *Mycosphaerellales* | *Teratosphaeriaceae* | *Hortaea werneckii* | *Hortaea werneckii* | Spider web with algae Dunaliella in a cave close to the ocean | Unknow |  | Savković et al. (2025) |
| *Dothideomycetes* | *Mycosphaerellales* | *Teratosphaeriaceae* | *Readeriella eucalypti* | *Readeriella eucalypti* | Unreported | North America | 1 | Belyagoubi et al. (2018) |
| *Dothideomycetes* | *Mytilinidiales* | *Gloniaceae* | *Cenococcum* sp. | *Cenococcum* sp. | Unreported | Canada | 1 | Vanderwolf et al. (2013a) |
| *Dothideomycetes* | *Phaeotrichales* | *Phaeotrichaceae* | *Phaeotrichum hystricinum* | *Phaeotrichum hystricinum* | Dung, frass (not bat) | Canada | 1 | Vanderwolf et al. (2013a) |
| *Dothideomycetes* | *Phaeotrichales* | *Phaeotrichaceae* | *Phaeotrichum* sp. | *Phaeotrichum* sp. | Bat | Canada | 1 | Vanderwolf et al. (2015) |
| *Dothideomycetes* | *Pleosporales* | *Acrocalymmaceae* | *Acrocalymma* sp. | *Acrocalymma* sp. | Organic litter, | Thailand | 1 | Suetrong et al. (2023) |
| *Dothideomycetes* | *Pleosporales* | *Acrocalymmaceae* | *Acrocalymma vagum* | *Acrocalymma vagum* | Unreported | China | 1 | Zhang et al. (2021) |
| *Dothideomycetes* | *Pleosporales* | *Astrosphaeriellaceae* | *Pithomyces* spp. | *Pithomyces* spp. | Air | France, Spain | 2 | Vanderwolf et al. (2013a); Leplat et al. (2018) |
| *Dothideomycetes* | *Pleosporales* | *Astrosphaeriellaceae* | *Pseudopithomyces chartarum* | *Pithomyces chartarum* | Air, cave wall, soil-sediment | Czech Republic, Korea, Slovakia | 3 | Vanderwolf et al. (2013a) |
| *Dothideomycetes* | *Pleosporales* | *Biatriosporaceae* | *Biatriospora* sp. | *Biatriospora* sp. | Unreported | Thailand | 1 | Suetrong et al. (2023) |
| *Dothideomycetes* | *Pleosporales* | *Biatriosporaceae* | Biatriosporaceae sp*.* | Biatriosporaceae sp*.* | Unreported | Thailand | 1 | Suetrong et al. (2023) |
| *Dothideomycetes* | *Pleosporales* | *Camarosporiaceae* | *Camarosporium aequivocum* | *Camarosporium aequivocum* | Cave wall | Israel | 1 | Vanderwolf et al. (2013a) |
| *Dothideomycetes* | *Pleosporales* | *Coniothyriaceae* | *Coniothyrium* sp. | *Coniothyrium* sp. | Air | France | 1 | , |
| *Dothideomycetes* | *Pleosporales* | *Coniothyriaceae* | *Neoconiothyrium* sp. | *Neoconiothyrium* sp. | Unreported | China | 1 | Zhang et al. (2021) |
| *Dothideomycetes* | *Pleosporales* | *Corynesporascaceae* | *Corynespora* sp. | *Corynespora* sp. | Unreported | China | 1 | Zhang et al. (2017) |
| *Dothideomycetes* | *Pleosporales* | *Cucurbitariaceae* | *Curreya* sp. | *Curreya* sp. | Unreported | China | 1 | Zhang et al. (2021) |
| *Dothideomycetes* | *Pleosporales* | *Cucurbitariaceae* | *Pyrenochaeta* sp. | *Pyrenochaeta* sp. | Unreported | Canada | 1 | Vanderwolf et al. (2013a) |
| *Dothideomycetes* | *Pleosporales* | *Delitschiaceae* | *Delitschia* sp. | *Delitschia* sp. | Air | Spain | 1 | Vanderwolf et al. (2013a) |
| *Dothideomycetes* | *Pleosporales* | *Dematiaceae* | Dematiaceae sp. | Dematiaceae sp. | Soil-sediment | Venezuela | 1 | Vanderwolf et al. (2013a) |
| *Dothideomycetes* | *Pleosporales* | [*Diaporthaceae*](https://www.indexfungorum.org/Names/families.asp?FamilyName=Diaporthaceae) | *Diaporthe eres* | *Diaporthe eres* | Unreported | China | 1 | Pereira et al. (2022) |
| *Dothideomycetes* | *Pleosporales* | [*Diaporthaceae*](https://www.indexfungorum.org/Names/families.asp?FamilyName=Diaporthaceae) | *Diaporthe fukushii* | *Diaporthe fukushii* | Unreported | China | 1 | Pereira et al. (2022) |
| *Dothideomycetes* | *Pleosporales* | [*Diaporthaceae*](https://www.indexfungorum.org/Names/families.asp?FamilyName=Diaporthaceae) | *Diaporthe melonis* | *Diaporthe melonis* | Air | China | 1 | Jiang et al. (2017) |
| *Dothideomycetes* | *Pleosporales* | [*Diaporthaceae*](https://www.indexfungorum.org/Names/families.asp?FamilyName=Diaporthaceae) | *Diaporthe nobilis* | *Diaporthe nobilis* | Unreported | China | 1 | Pereira et al. (2022) |
| *Dothideomycetes* | *Pleosporales* | [*Diaporthaceae*](https://www.indexfungorum.org/Names/families.asp?FamilyName=Diaporthaceae) | *Diaporthe phaseolorum* | *Diaporthe phaseolorum* | Unreported | China | 1 | Zhang et al. (2021) |
| *Dothideomycetes* | *Pleosporales* | [*Diaporthaceae*](https://www.indexfungorum.org/Names/families.asp?FamilyName=Diaporthaceae) | *Diaporthe phoenicicola* | *Diaporthe phoenicicola* | Unreported | China | 1 | Zhang et al. (2017) |
| *Dothideomycetes* | *Pleosporales* | [*Diaporthaceae*](https://www.indexfungorum.org/Names/families.asp?FamilyName=Diaporthaceae) | *Diaporthe rudis* | *Diaporthe rudis* | Air | Spain | 1 | Savković et al. (2025) |
| *Dothideomycetes* | *Pleosporales* | [*Diaporthaceae*](https://www.indexfungorum.org/Names/families.asp?FamilyName=Diaporthaceae) | Diaporthe sp. 1 | Diaporthe sp. 1 | Air | Brazil | 1 | Cunha et al. (2020) |
| *Dothideomycetes* | *Pleosporales* | [*Diaporthaceae*](https://www.indexfungorum.org/Names/families.asp?FamilyName=Diaporthaceae) | Diaporthe sp. 2 | Diaporthe sp. 2 | Air | Brazil | 1 | Cunha et al. (2020) |
| *Dothideomycetes* | *Pleosporales* | [*Diaporthaceae*](https://www.indexfungorum.org/Names/families.asp?FamilyName=Diaporthaceae) | *Diaporthe* spp. | *Diaporthe* spp. | Air, Organic litter, soil-sediment, Trigonilus corallinus, | Brazil, China, Malaysia, Thailand | 4 | Zhang et al. (2021) |
| *Dothideomycetes* | *Pleosporales* | [*Diaporthaceae*](https://www.indexfungorum.org/Names/families.asp?FamilyName=Diaporthaceae) | *Diaporthe vaccinii* | *Diaporthe vaccinii* | Unreported | China | 1 | Pereira et al. (2022) |
| *Dothideomycetes* | *Pleosporales* | *Diaporthaceae* | *Diaporthe vaccinii* | *Phomopsis vaccinii* | Unreported | China | 1 | Zhang (2019) |
| *Dothideomycetes* | *Pleosporales* | *Diaporthaceae* | *Phomopsis* spp. | *Phomopsis* spp. | Air | China, Czech Republic | 1 | Vanderwolf et al. (2013a); Zhang et al. (2017) |
| *Dothideomycetes* | *Pleosporales* | *Dictyosporiaceae* | *Dictyosporium elegans* | *Dictyosporium elegans* | Wood | Romania | 1 | Vanderwolf et al. (2013a) |
| *Dothideomycetes* | *Pleosporales* | *Dictyosporiaceae* | *Dictyosporium* sp. | *Dictyosporium* sp. | Soil-sediment | USA | 1 | Vanderwolf et al. (2013a) |
| *Dothideomycetes* | *Pleosporales* | *Dictyosporiaceae* | *Jalapriya pulchra* | *Jalapriya pulchra* | Air | Spain | 1 | Savković et al. (2025) |
| *Dothideomycetes* | *Pleosporales* | *Dictyosporiaceae* | Pseudocoleophoma sp. | Pseudocoleophoma sp. | Unreported | China | 1 | Zhang et al. (2021) |
| *Dothideomycetes* | *Pleosporales* | *Dictyosporiaceae* | *Speira toruloides* | *Dictyosporium toruloides* | Unreported | Canada | 1 | Vanderwolf et al. (2013a) |
| *Dothideomycetes* | *Pleosporales* | *Didymellaceae* | *Allophoma* spp. | *Allophoma* spp. | Air | Poland, China | 2 | Jiang et al. 2017a, b; Zhang et al. (2021) |
| *Dothideomycetes* | *Pleosporales* | *Didymellaceae* | *Boeremia exigua* | *Boeremia exigua* | Air, cave wall | China, Czech Republic, Israel | 3 | Vanderwolf et al. (2013a) |
| *Dothideomycetes* | *Pleosporales* | *Didymellaceae* | *Boeremia* sp. | *Boeremia* sp. | Air | China | 1 | Jiang et al. (2017) |
| *Dothideomycetes* | *Pleosporales* | *Didymellaceae* | *Didymella bellidis* | *Didymella bellidis* | Unreported | China | 1 | Zhang et al. (2021) |
| *Dothideomycetes* | *Pleosporales* | *Didymellaceae* | *Didymella glomerata* | *Didymella glomerata* | Air | China | 1 | Zhang et al. (2021) |
| *Dothideomycetes* | *Pleosporales* | *Didymellaceae* | *Didymella glomerata* | *Phoma glomerata* | Air | China | 1 | Vanderwolf et al. (2013a) |
| *Dothideomycetes* | *Pleosporales* | *Didymellaceae* | *Didymella macrostoma* | *Didymella macrostoma* | Air | China, spain | 2 | Zhang et al. (2017) |
| *Dothideomycetes* | *Pleosporales* | *Didymellaceae* | *Didymella pinodella* | *Didymella pinodella* | Unreported | China | 1 | Zhang et al. (2021) |
| *Dothideomycetes* | *Pleosporales* | *Didymellaceae* | *Didymella rhei* | *Didymella rhei* | Unreported | China | 1 | Zhang et al. (2021) |
| *Dothideomycetes* | *Pleosporales* | *Didymellaceae* | *Didymella senecionicola* | *Phoma senecionis* | Unreported | China | 1 | Zhang et al. (2017) |
| *Dothideomycetes* | *Pleosporales* | *Didymellaceae* | *Didymella* spp. | *Didymella* spp. | Air | China, Spain | 2 | Vanderwolf et al. (2013a); Jiang et al. (2017) |
| *Dothideomycetes* | *Pleosporales* | *Didymellaceae* | *Ectophoma insulana* | *Phoma insulana* | Unreported | China | 1 | Zhang et al. (2017) |
| *Dothideomycetes* | *Pleosporales* | *Didymellaceae* | *Ectophoma* sp. | *Ectophoma* sp. | Air | Thailand | 1 | Suetrong et al. (2023) |
| *Dothideomycetes* | *Pleosporales* | *Didymellaceae* | *Epicoccum draconis* | *Epicoccum draconis* | Unreported | China | 1 | Zhang et al. (2021) |
| *Dothideomycetes* | *Pleosporales* | *Didymellaceae* | *Epicoccum nigrum* | *Epicoccum nigrum* | Air, soil-sediment, wood, cave wall | Brazil, China, Czech Republic, France, Iran, Israel, Italy, Japan, Poland, Romania, Slovakia, Spain, USA | 13 | Vanderwolf et al. (2013a) |
| *Dothideomycetes* | *Pleosporales* | *Didymellaceae* | *Epicoccum plurivorum* | *Epicoccum plurivorum* | Unreported | China | 1 | Zhang et al. (2021) |
| *Dothideomycetes* | *Pleosporales* | *Didymellaceae* | *Epicoccum* spp. | *Epicoccum* spp. | Organic litter, soil, sediment, cave wall, Air, | France, Slovakia, Spain, Thailand, UK, USA | 6 | Vanderwolf et al. (2013a); Leplat et al. (2018); Suetrong et al. (2023) |
| *Dothideomycetes* | *Pleosporales* | *Didymellaceae* | *Juxtiphoma eupyrena* | *Juxtiphoma eupyrena* | Air, soil-sediment, earthworm casts, cave wall | China, Czech Republic, Israel, Slovakia | 4 | Vanderwolf et al. (2013a) |
| *Dothideomycetes* | *Pleosporales* | *Didymellaceae* | *Leptosphaerulina chartarum* | *Leptosphaerulina chartarum* | Air | China | 1 | Jiang et al. (2017) |
| *Dothideomycetes* | *Pleosporales* | *Didymellaceae* | *Leptosphaerulina* spp. | *Leptosphaerulina* spp. | Air, Sediment | Brazil, Spain, USA | 3 | Vanderwolf et al. (2013a); Alves et al. (2022) |
| *Dothideomycetes* | *Pleosporales* | *Didymellaceae* | *Microsphaeropsis arundinis* | *Microsphaeropsis arundinis* | Unreported | China | 1 | Zhang et al. (2017) |
| *Dothideomycetes* | *Pleosporales* | *Didymellaceae* | *Microsphaeropsis* sp. | *Microsphaeropsis* sp. | Air | Thailand | 1 | Suetrong et al. (2023) |
| *Dothideomycetes* | *Pleosporales* | *Didymellaceae* | *Neoascochyta paspali* | *Neoascochyta paspali* | Unreported | China | 1 | Zhang (2019) |
| *Dothideomycetes* | *Pleosporales* | *Didymellaceae* | *Neoascochyta* spp. | *Neoascochyta* spp. | Air, cave wall, limestone | China, Thailand | 2 | Jiang et al. (2017a, b); Suetrong et al. (2023) |
| *Dothideomycetes* | *Pleosporales* | *Didymellaceae* | *Neodidymella thailandica* | *Neodidymella thailandicum* | Air | Brazil | 1 | Cunha et al. (2020) |
| *Dothideomycetes* | *Pleosporales* | *Didymellaceae* | Nothophoma sp. 1 | Nothophoma sp. 1 | Bat | Brazil | 1 | Cunha et al. (2020) |
| *Dothideomycetes* | *Pleosporales* | *Didymellaceae* | Nothophoma sp. 2 | Nothophoma sp. 2 | Air | Brazil | 1 | Cunha et al. (2020) |
| *Dothideomycetes* | *Pleosporales* | *Didymellaceae* | *Paraboeremia oligotrophica* | *Paraboeremia oligotrophica* | Limestone | China | 1 | Jiang et al. (2016) |
| *Dothideomycetes* | *Pleosporales* | *Didymellaceae* | *Paraboeremia selaginellae* | *Paraboeremia selaginellae* | Unreported | China | 1 | Zhang et al. (2021) |
| *Dothideomycetes* | *Pleosporales* | *Didymellaceae* | *Paraphoma radicina* | *Phoma radicina* |  | Canada | 1 | Vanderwolf et al. (2013a) |
| *Dothideomycetes* | *Pleosporales* | *Didymellaceae* | *Phoma herbarum* | *Phoma herbarum* | Air, cave wall | China, Poland | 2 | Ogórek et al. (2014c); Zhang et al. (2017) |
| *Dothideomycetes* | *Pleosporales* | *Didymellaceae* | *Phoma leveillei* | *Phoma leveillei* | Unreported | Slovakia | 1 | Ogórek et al. (2016d) |
| *Dothideomycetes* | *Pleosporales* | *Didymellaceae* | *Stagonosporopsis cucurbitacearum* | *Stagonosporopsis cucurbitacearum* | Air, limestone | China | 1 | Jiang et al. (2017) |
| *Dothideomycetes* | *Pleosporales* | *Didymosphaeriaceae* | *Deniquelata quercina* | *Deniquelata quercina* | Bat | Brazil | 1 | Cunha et al. (2020) |
| *Dothideomycetes* | *Pleosporales* | *Didymosphaeriaceae* | *Didymosphaeria* sp. | *Didymosphaeria* sp. | Unreported | China | 1 | Zhang et al. (2021) |
| *Dothideomycetes* | *Pleosporales* | *Didymosphaeriaceae* | *Didymosphaeria variabile* | *Didymosphaeria variabile* | Unreported | China | 1 | Zhang et al. (2021) |
| *Dothideomycetes* | *Pleosporales* | *Didymosphaeriaceae* | Didymosphaeriaceae sp. | Didymosphaeriaceae sp. | Organic litter, | Thailand | 1 | Suetrong et al. (2023) |
| *Dothideomycetes* | *Pleosporales* | *Didymosphaeriaceae* | *Letendraea helminthicola* | *Letendraea helminthicola* | Unreported | China | 1 | Zhang (2019) |
| *Dothideomycetes* | *Pleosporales* | *Didymosphaeriaceae* | *Montagnula* spp. | *Montagnula* spp. | Soil-sediment, | China, Thailand | 2 | Suetrong et al. (2023) |
| *Dothideomycetes* | *Pleosporales* | *Didymosphaeriaceae* | *Paracamarosporium hawaiiense* | *Paracamarosporium hawaiiense* | Unreported | Brazil, France, Spain | 3 | Zhang et al. (2021) |
| *Dothideomycetes* | *Pleosporales* | *Didymosphaeriaceae* | *Paraconiothyrium archidendri* | *Paraconiothyrium archidendri* | Bat | Brazil | 1 | Cunha et al. (2020) |
| *Dothideomycetes* | *Pleosporales* | *Didymosphaeriaceae* | *Paraconiothyrium brasiliense* | *Paraconiothyrium brasiliense* | Unreported | China | 1 | Zhang et al. (2021) |
| *Dothideomycetes* | *Pleosporales* | *Didymosphaeriaceae* | *Paraconiothyrium estuarinum* | *Paraconiothyrium estuarinum* | Unreported | China | 1 | Zhang et al. (2021) |
| *Dothideomycetes* | *Pleosporales* | *Didymosphaeriaceae* | *Paraconiothyrium* sp. | *Paraconiothyrium* sp. | Unreported | China | 1 | Zhang (2019) |
| *Dothideomycetes* | *Pleosporales* | *Didymosphaeriaceae* | *Paraphaeosphaeria sporulosa* | *Paraconiothyrium sporulosum* | Soil-sediment | USA | 1 | Vanderwolf et al. (2013a) |
| *Dothideomycetes* | *Pleosporales* | *Didymosphaeriaceae* | *Paraphaeosphaeria sporulosa* | *Paraphaeosphaeria sporulosa* | Air, sediment | China, Poland | 2 | Vanderwolf et al. (2013a); Man (2017); Zhang et al. (2021) |
| *Dothideomycetes* | *Pleosporales* | *Didymosphaeriaceae* | *Pseudoscopulariopsis asperispora* | *Pseudoscopulariopsis asperispora* | Soil | China | 1 | zhang et al. (2021) |
| *Dothideomycetes* | *Pleosporales* | *Didymosphaeriaceae* | *Spegazzinia* sp. | *Spegazzinia* sp. | Air | Spain | 1 | Vanderwolf et al. (2013a) |
| *Dothideomycetes* | *Pleosporales* | *Herpotrichiellaceae* | Herpotrichiellaceae sp. | Herpotrichiellaceae sp. | Cave wall | France | 1 | Vanderwolf et al. (2013a) |
| *Dothideomycetes* | *Pleosporales* | *Incertae sedis* | *Clavariopsis azlanii* | *Clavariopsis azlanii* | Soil, sediment, water | Puerto Rico | 1 | Vanderwolf et al. (2013a) |
| *Dothideomycetes* | *Pleosporales* | *Incertae sedis* | *Cumuliphoma omnivirens* | *Cumuliphoma omnivirens* | Unreported | China | 1 | Zhang et al. (2021) |
| *Dothideomycetes* | *Pleosporales* | *Incertae sedis* | *Cumuliphoma* sp. | *Cumuliphoma* sp. | Soil-sediment, Organic litter, | Thailand | 1 | Suetrong et al. (2023) |
| *Dothideomycetes* | *Pleosporales* | *Incertae sedis* | *Ochrocladosporium elatum* | *Ochrocladosporium elatum* | Air | Slovakia | 1 | Vanderwolf et al. (2013a) |
| *Dothideomycetes* | *Pleosporales* | *Incertae sedis* | *Pseudorobillarda* sp. | *Pseudorobillarda* sp. | Unreported | Thailand | 1 | Suetrong et al. (2023) |
| *Dothideomycetes* | *Pleosporales* | *Incertae sedis* | Sporocybe spp. | *Sporocybe* spp. | Soil-sediment | France, Spain | 2 | Vanderwolf et al. (2013a) |
| *Dothideomycetes* | *Pleosporales* | *Latoruaceae* | *Latorua caligans* | *Latorua caligans* | Unreported | China | 1 | Zhang et al. (2017) |
| *Dothideomycetes* | *Pleosporales* | *Latoruaceae* | *Triseptata* sp. | *Triseptata* sp. | Organic litter, | Thailand | 1 | Suetrong et al. (2023) |
| *Dothideomycetes* | *Pleosporales* | *Lentitheciaceae* | *Aquilomyces* sp. | *Aquilomyces* sp. | Symptomatic specimens of *Proteus anguinus* | Slovenia | 1 | Savković et al. (2025) |
| *Dothideomycetes* | *Pleosporales* | *Lentitheciaceae* | *Keissleriella* sp. | *Keissleriella* sp. | Air | Spain | 1 | Vanderwolf et al. (2013a) |
| *Dothideomycetes* | *Pleosporales* | *Lophiostomataceae* | *Lophiostoma corticola* | *Lophiostoma corticola* | Unreported | China | 1 | Zhang et al. (2017) |
| *Dothideomycetes* | *Pleosporales* | *Lophiostomataceae* | *Lophiostoma* sp. | *Lophiostoma* sp. | Soil | China | 1 | Jiang et al. (2017) |
| *Dothideomycetes* | *Pleosporales* | *Massarinaceae* | *Helminthosporium* spp. | *Helminthosporium* spp. | Soil-sediment | Canada, France | 2 | Vanderwolf et al. (2013a) |
| *Dothideomycetes* | *Pleosporales* | *Massarinaceae* | *Helminthosporium trichellum* | *Helminthosporium trichellum* | Wood | Switzerland | 1 | Vanderwolf et al. (2013a) |
| *Dothideomycetes* | *Pleosporales* | *Massarinaceae* | *Massarina igniaria* | *Massarina igniaria* | Unreported | China | 1 | Zhang et al. (2021) |
| *Dothideomycetes* | *Pleosporales* | *Massarinaceae* | *Massarina* spp. | *Massarina* spp. | Air, unreported | China, Spain | 1 | Vanderwolf et al. (2013a) |
| *Dothideomycetes* | *Pleosporales* | *Massarinaceae* | Massarinaceae sp. | Massarinaceae sp. | Air | China | 1 | Jiang et al. (2017) |
| *Dothideomycetes* | *Pleosporales* | *Melanommataceae* | *Herpotrichia* sp. | *Herpotrichia* sp. | Air | Spain | 1 | Vanderwolf et al. (2013a) |
| *Dothideomycetes* | *Pleosporales* | *Melanommataceae* | *Pleotrichocladium opacum* | *Pleotrichocladium opacum* | Soil-sediment | Canada, Slovakia | 2 | Vanderwolf et al. (2013a) |
| *Dothideomycetes* | *Pleosporales* | *Neomassarinaceae* | *Neomassarina thailandica* | *Neomassarina thailandica* | Unreported | China | 1 | Zhang et al. (2021) |
| *Dothideomycetes* | *Pleosporales* | *Neopyrenochaetaceae* | *Neopyrenochaeta inflorescentiae* | *Neopyrenochaeta inflorescentiae* | Unreported | China | 1 | Zhang et al. (2021) |
| *Dothideomycetes* | *Pleosporales* | *Nigrogranaceae* | *Nigrograna cangshanensis* | *Nigrograna cangshanensis* | Unreported | China | 1 | Zhang (2019) |
| *Dothideomycetes* | *Pleosporales* | *Nigrogranaceae* | *Nigrograna mackinnonii* | *Nigrograna mackinnonii* | Unreported | China, Venezuela | 2 | Zhang et al. (2021) |
| *Dothideomycetes* | *Pleosporales* | *Nigrogranaceae* | *Nigrograna* spp. | *Nigrograna* spp. | Rock, water, | China, Thailand | 2 | Zhang et al. (2021); Suetrong et al. (2023) |
| *Dothideomycetes* | *Pleosporales* | *OccultibambUSAceae* | *Occultibambusa* sp. | *Occultibambusa* sp. | Soil-sediment, | Thailand | 1 | Suetrong et al. (2023) |
| *Dothideomycetes* | *Pleosporales* | *Periconiaceae* | *Periconia macroSpainosa* | *Periconia* cf. *macroSpainosa* | Earthworm casts | Slovakia | 1 | Vanderwolf et al. (2013a) |
| *Dothideomycetes* | *Pleosporales* | *Periconiaceae* | *Periconia macroSpainosa* | *Periconia macroSpainosa* | Air, cave wall, wood, soil-sediment, earthworm casts, insects, water | China, Romania, Slovakia | 3 | Vanderwolf et al. (2013a); Brad et al. (2018) |
| *Dothideomycetes* | *Pleosporales* | *Periconiaceae* | *Periconia* spp. | *Periconia* spp. | Air, rovk, soil, sediment | Australia, China, Romania, Spain, Thailand | 5 | Vanderwolf et al. (2013a); Zhang et al. (2021); Suetrong et al. (2023) |
| *Dothideomycetes* | *Pleosporales* | *Phaeosphaeriaceae* | *Ampelomyces humuli* | *Ampelomyces humuli* | Unreported | China | 1 | Zhang et al. (2021) |
| *Dothideomycetes* | *Pleosporales* | *Phaeosphaeriaceae* | *Paraphoma chrysanthemicola* | *Paraphoma chrysanthemicola* | Other, cave wall | China, Czech Republic | 2 | Vanderwolf et al. (2013a); Zhang et al. (2021) |
| *Dothideomycetes* | *Pleosporales* | *Phaeosphaeriaceae* | *Paraphoma fimeti* | *Paraphoma fimeti* | Bat | Netherlands, Slovakia | 2 | Ogórek et al. (2016b, c) |
| *Dothideomycetes* | *Pleosporales* | *Phaeosphaeriaceae* | *Paraphoma radicina* | *Paraphoma radicina* | Bat | Canada, China | 2 | Vanderwolf et al. (2013a); Zhang et al. (2017) |
| *Dothideomycetes* | *Pleosporales* | *Phaeosphaeriaceae* | *Paraphoma* sp. | *Paraphoma* sp. | Symptomatic specimens of *Proteus anguinus* | Slovenia | 1 | Savković et al. (2025) |
| *Dothideomycetes* | *Pleosporales* | *Phaeosphaeriaceae* | *Parastagonospora nodorum* | *Parastagonospora nodorum* | Unreported | China | 1 | Zhang et al. (2021) |
| *Dothideomycetes* | *Pleosporales* | *Phaeosphaeriaceae* | *Phaeosphaeria annulata* | *Phaeosphaeria annulata* | Air | Spain | 1 | Vanderwolf et al. (2013a) |
| *Dothideomycetes* | *Pleosporales* | *Phaeosphaeriaceae* | *Phaeosphaeria eriobotryae* | *Microdiplodia miyakei* | Sediment | China | 1 | Man et al. (2015); Man (2017) |
| *Dothideomycetes* | *Pleosporales* | *Phaeosphaeriaceae* | *Phaeosphaeria eriobotryae* | *Phaeosphaeria eriobotryae* | Unreported | China | 1 | Man et al. (2015); Man (2017) |
| *Dothideomycetes* | *Pleosporales* | *Phaeosphaeriaceae* | *Phaeosphaeria fusispora* | *Phaeosphaeria fusispora* | Unreported | China | 1 | Zhang et al. (2017) |
| *Dothideomycetes* | *Pleosporales* | *Phaeosphaeriaceae* | *Phaeosphaeria microscopica* | *Phaeosphaeria microscopica* | Unreported | China | 1 | Zhang (2019) |
| *Dothideomycetes* | *Pleosporales* | *Phaeosphaeriaceae* | *Phaeosphaeria musae* | *Phaeosphaeria musae* | Air | Brazil | 1 | Cunha et al. (2020) |
| *Dothideomycetes* | *Pleosporales* | *Phaeosphaeriaceae* | *Phaeosphaeria nodorum* | *Phaeosphaeria nodorum* | Soil-sediment | South Africa | 1 | Vanderwolf et al. (2013a) |
| *Dothideomycetes* | *Pleosporales* | *Phaeosphaeriaceae* | *Phaeosphaeria oryzae* | *Phaeosphaeria oryzae* | Unreported | China | 1 | Zhang (2019) |
| *Dothideomycetes* | *Pleosporales* | *Phaeosphaeriaceae* | Phaeosphaeria sp. | *Phaeosphaeria michotii* | Unreported | China | 1 | Zhang et al. (2021) |
| *Dothideomycetes* | *Pleosporales* | *Phaeosphaeriaceae* | Phaeosphaeria sp. | *Phaeosphaeria neglecta* | Unreported | China | 1 | Zhang (2019) |
| *Dothideomycetes* | *Pleosporales* | *Phaeosphaeriaceae* | *Phaeosphaeria* sp. | *Phaeosphaeria* sp. | Unreported | Canada, China | 2 | Connell and Staudigel (2013); Zhang (2019) |
| *Dothideomycetes* | *Pleosporales* | *Phaeosphaeriaceae* | Phaeosphaeriaceae sp. | Phaeosphaeriaceae sp. | Limestone | China | 1 | Jiang et al. (2017) |
| *Dothideomycetes* | *Pleosporales* | *Phaeosphaeriaceae* | Phaeosphaeriopsis spp. | Phaeosphaeriopsis spp. | Water | China, Malaysia | 1 | Zhang (2019); Wasti et al. (2021) |
| *Dothideomycetes* | *Pleosporales* | *Phaeosphaeriaceae* | *Septoriella oudemansii* | *Septoriella oudemansii* | Unreported | China | 1 | Zhang et al. (2021) |
| *Dothideomycetes* | *Pleosporales* | *Phaeosphaeriaceae* | *Setophaeosphaeria hemerocallidis* | *Setophaeosphaeria hemerocallidis* | Unreported | China | 1 | Zhang et al. (2021) |
| *Dothideomycetes* | *Pleosporales* | *Phaeosphaeriaceae* | *Setophaeosphaeria microspora* | *Setophaeosphaeria microspora* | Soil | China | 1 | zhang et al. (2021) |
| *Dothideomycetes* | *Pleosporales* | *Phaeosphaeriaceae* | *Setophaeosphaeria sp.* | *Setophaeosphaeria sp.* | Unreported | China | 1 | Zhang et al. (2021) |
| *Dothideomycetes* | *Pleosporales* | *Phaeosphaeriaceae* | *Setophoma caverna* | *Setophoma caverna* | Rock | China | 1 | Savković et al. (2025) |
| *Dothideomycetes* | *Pleosporales* | *Phaeosphaeriaceae* | *Setophoma* spp. | *Setophoma* spp. | Limestone, Organic litter | China, Thailand | 1 | Jiang et al. (2017) |
| *Dothideomycetes* | *Pleosporales* | *Phaeosphaeriaceae* | *Setophoma terrestris* | *Setophoma terrestris* | Unreported | China | 1 | Zhang (2019); Zhang et al. (2021) |
| *Dothideomycetes* | *Pleosporales* | *Phaeosphaeriaceae* | *Setophoma vernoniae* | *Setophoma vernoniae* | Unreported | China | 1 | Zhang (2019) |
| *Dothideomycetes* | *Pleosporales* | *Phaeosphaeriaceae* | *Stagonospora* sp. | *Stagonospora* sp. | Air, cave wall, limestone | China, France | 2 | Jiang et al. (2017a, b); Leplat et al. (2018) |
| *Dothideomycetes* | *Pleosporales* | [*Pleosporaceae*](https://www.indexfungorum.org/Names/families.asp?FamilyName=Pleosporaceae) | *Alternaria abundans* | *Alternaria abundans* | Air, rock | China, Poland, Slovakia | 3 | Pusz et al. 2015, Ogórek et al. (2018); Zhang et al. (2021) |
| *Dothideomycetes* | *Pleosporales* | *Pleosporaceae* | *Alternaria alternariae* | *Alternaria alternariae* | Air, rock | Poland | 1 | Ogórek et al. (2013) |
| *Dothideomycetes* | *Pleosporales* | *Pleosporaceae* | *Alternaria alternata* | *Alternaria alternata* | Air, water, limestone, soil-sediment, other, bat guano, cave wall, rock surface, wood | Bahamas, Brazil, China, Czech Republic, Egypt, Israel, Poland, Romania, Russia, Slovakia, Spain, USA | 12 | Vanderwolf et al. (2013a); Ogórek et al. (2016); Jiang et al. (2017); Man (2017); Elkhateeb et al. (2022); Ma et al. (2025) |
| *Dothideomycetes* | *Pleosporales* | *Pleosporaceae* | Alternaria alternata complex | Alternaria alternata complex | Air | Poland | 1 | Kokurewicz et al. (2016) |
| *Dothideomycetes* | *Pleosporales* | *Pleosporaceae* | *Alternaria atra* | *Alternaria atra* | Unreported | Unknow |  | Zhang et al. (2021) |
| *Dothideomycetes* | *Pleosporales* | *Pleosporaceae* | *Alternaria atra* | *Ulocladium atrum* | Air, cave wall | Israel, Slovakia | 2 | Vanderwolf et al. (2013a) |
| *Dothideomycetes* | *Pleosporales* | *Pleosporaceae* | *Alternaria botrytis* | *Alternaria botrytis* | Wood, Air | Poland, Switzerland | 2 | Vanderwolf et al. (2013a); Kokurewicz et al. (2016); Pusz et al. (2018b) |
| *Dothideomycetes* | *Pleosporales* | *Pleosporaceae* | *Alternaria botrytis* | *Alternaria oudemansii* | Air | Slovakia | 1 | Vanderwolf et al. (2013a); Zhang et al. (2021) |
| *Dothideomycetes* | *Pleosporales* | *Pleosporaceae* | *Alternaria botrytis* | *Ulocladium botrytis* | Air, soil-sediment, cave wall, wood | Czech Republic, Israel, Poland, Romania, USA | 5 | Vanderwolf et al. (2013a); Kokurewicz et al. (2016) |
| *Dothideomycetes* | *Pleosporales* | *Pleosporaceae* | *Alternaria brevicolla* | *Alternaria brevicolla* | Soil-sediment | Russia | 1 | Vanderwolf et al. (2013a) |
| *Dothideomycetes* | *Pleosporales* | *Pleosporaceae* | *Alternaria chartarum* | *Alternaria chartarum* | Unreported | Unknow |  | Zhang et al. (2021) |
| *Dothideomycetes* | *Pleosporales* | *Pleosporaceae* | *Alternaria chartarum* | *Ulocladium chartarum* | Air, cave wall | Czech Republic, Israel, Slovakia, South Africa | 4 | Vanderwolf et al. (2013a) |
| *Dothideomycetes* | *Pleosporales* | *Pleosporaceae* | *Alternaria chlamydosporigena* | *Alternaria chlamydosporigena* | Air | Spain | 1 | Savković et al. (2025) |
| *Dothideomycetes* | *Pleosporales* | *Pleosporaceae* | *Alternaria consortialis* | *Ulocladium consortiale* | Cave wall | Czech Republic | 1 | Vanderwolf et al. (2013a) |
| *Dothideomycetes* | *Pleosporales* | *Pleosporaceae* | *Alternaria humicola* | *Alternaria humicola* | Soil-sediment | Russia | 1 | Vanderwolf et al. (2013a) |
| *Dothideomycetes* | *Pleosporales* | *Pleosporaceae* | *Alternaria infectoria* | *Alternaria infectoria* | Air | Spain | 1 | Zhang et al. (2021) |
| *Dothideomycetes* | *Pleosporales* | *Pleosporaceae* | *Alternaria jacinthicola* | *Alternaria jacinthicola* | Air | Brazil | 1 | Alves et al. (2022), Savković et al. (2025) |
| *Dothideomycetes* | *Pleosporales* | *Pleosporaceae* | *Alternaria longipes* | *Alternaria longipes* | Air, cave wal | China, Romania | 2 | Nováková et al. (2018); Ma et al. (2025) |
| *Dothideomycetes* | *Pleosporales* | *Pleosporaceae* | *Alternaria mali* | *Alternaria mali* | Unreported | China | 1 | Zhang (2019); Zhang et al. (2021) |
| *Dothideomycetes* | *Pleosporales* | *Pleosporaceae* | *Alternaria mouchaccae* | *Alternaria mouchaccae* | Cave wall | Israel | 1 | Vanderwolf et al. (2013a) |
| *Dothideomycetes* | *Pleosporales* | *Pleosporaceae* | *Alternaria oudemansii* | *Ulocladium oudemansii* | Air | Slovakia | 1 | Vanderwolf et al. (2013a) |
| *Dothideomycetes* | *Pleosporales* | *Pleosporaceae* | *Alternaria radicina* | *Alternaria radicina* | Cave wall | Israel | 1 | Vanderwolf et al. (2013a) |
| *Dothideomycetes* | *Pleosporales* | *Pleosporaceae* | *Alternaria solani* | *Alternaria solani* | Unreported | China | 1 | Zhang et al. (2021) |
| *Dothideomycetes* | *Pleosporales* | *Pleosporaceae* | *Alternaria* spp. | *Alternaria* spp. | Air, rock, soil-sediment, unreported, cave wall, insects, bat guano, water | Australia, China, France, Greece, Italy, Japan, Kazakhstan, Poland, Puerto Rico, Romania, Slovakia, Slovenia, South Africa, Spain, Thailand, Ukraine, USA, Venezuela | 19 | Wang et al. (2010); Vanderwolf et al. (2013a); Bercea et al. (2018); Nováková et al. (2018); Leplat et al. (2018); Suetrong et al. (2023) |
| *Dothideomycetes* | *Pleosporales* | *Pleosporaceae* | *Alternaria tamaricis* | *Alternaria tamaricis* | rock | China, Poland, Serbia | 3 | Zhang et al. (2017); Zhang et al. (2021) |
| *Dothideomycetes* | *Pleosporales* | *Pleosporaceae* | *Alternaria tenuissima* | *Alternaria tenuissima* | Air, cave wall, rock, soil, water, , wood | China, Czech Republic, Israel, Poland, Romania, Slovakia, Spain | 7 | Vanderwolf et al. (2013a); Zhang et al. (2021) |
| *Dothideomycetes* | *Pleosporales* | *Pleosporaceae* | *Bipolaris cynodontis* | *Bipolaris cynodontis* | Air | Spain | 1 | Savković et al. (2025) |
| *Dothideomycetes* | *Pleosporales* | *Pleosporaceae* | *Bipolaris sorokiniana* | *Bipolaris sorokiniana* | Unreported | Poland | 1 | Pusz et al. (2018b) |
| *Dothideomycetes* | *Pleosporales* | *Pleosporaceae* | *Bipolaris* spp. | *Bipolaris* spp. | Unreported, soil-sediment | Puerto Rico, USA | 2 | Vanderwolf et al. (2013a) |
| *Dothideomycetes* | *Pleosporales* | *Pleosporaceae* | *Curvularia brachyspora* | *Curvularia brachyspora* | Soil-sediment | India | 1 | Vanderwolf et al. (2013a) |
| *Dothideomycetes* | *Pleosporales* | *Pleosporaceae* | *Curvularia eragrostidis* | *Curvularia eragrostidis* | Bat guano | Puerto Rico | 1 | Vanderwolf et al. (2013a) |
| *Dothideomycetes* | *Pleosporales* | *Pleosporaceae* | *Curvularia hawaiiensis* | *Bipolaris hawaiiensis* | Cave wall | Israel | 1 | Vanderwolf et al. (2013a) |
| *Dothideomycetes* | *Pleosporales* | *Pleosporaceae* | *Curvularia hawaiiensis* | *Curvularia hawaiiensis* | Cave wall | Israel | 1 | Vanderwolf et al. (2013a) |
| *Dothideomycetes* | *Pleosporales* | *Pleosporaceae* | *Curvularia lunata* | *Curvularia lunata* | Bat guano, soil-sediment | Malaysia, Puerto Rico | 2 | Vanderwolf et al. (2013a); Wasti et al. (2021) |
| *Dothideomycetes* | *Pleosporales* | *Pleosporaceae* | *Curvularia senegalensis* | *Curvularia senegalensis* | Air | USA | 1 | Suetrong et al. (2023) |
| *Dothideomycetes* | *Pleosporales* | *Pleosporaceae* | Curvularia spp. | Curvularia spp. | Soil-sediment, air, unreported | Brazil, India, Puerto Rico, Spain, Thailand | 5 | Taylor et al. (2013); Vanderwolf et al. (2013a); Cunha et al. (2020); Suetrong et al. (2023) |
| *Dothideomycetes* | *Pleosporales* | *Pleosporaceae* | *Curvularia trifolii* | *Curvularia trifolii* | Unreported | China | 1 | Zhang et al. (2021) |
| *Dothideomycetes* | *Pleosporales* | *Pleosporaceae* | *Drechslera avenacea* | *Drechslera avenacea* | Air | Czech Republic | 1 | Vanderwolf et al. (2013a) |
| *Dothideomycetes* | *Pleosporales* | *Pleosporaceae* | *Drechslera* spp. | *Drechslera* spp. | Soil-sediment, air | France, Puerto Rico, Spain | 3 | Vanderwolf et al. (2013a); Leplat et al. (2018) |
| *Dothideomycetes* | *Pleosporales* | *Pleosporaceae* | *Edenia gomezpompae* | *Edenia gomezpompae* | Air | Brazil | 1 | Alves et al. (2022); Savković et al. (2025) |
| *Dothideomycetes* | *Pleosporales* | *Pleosporaceae* | *Pleospora* spp. | *Pleospora* spp. | Air | Spain | 1 | Vanderwolf et al. (2013a) |
| *Dothideomycetes* | *Pleosporales* | *Pleosporaceae* | Pleosporaceae sp. | Pleosporaceae sp. | Unreported | Thailand | 1 | Suetrong et al. (2023) |
| *Dothideomycetes* | *Pleosporales* | *Pleosporaceae* | *Pyrenophora tritici-repentis* | *Pyrenophora tritici-repentis* | Unreported | China | 1 | Zhang et al. (2021) |
| *Dothideomycetes* | *Pleosporales* | *Pleosporaceae* | *Stemphylium botryosum* | *Stemphylium botryosum* | Insects, soil-sediment, unreported cave wall | Czech Republic, Poland, UK | 3 | Vanderwolf et al. (2013a) |
| *Dothideomycetes* | *Pleosporales* | *Pleosporaceae* | Stemphylium spp. | Stemphylium spp. | Air | Australia, Spain | 2 | Vanderwolf et al. (2013a) |
| *Dothideomycetes* | *Pleosporales* | *Pleosporaceae* | *Stemphylium vesicarium* | *Pleospora herbarum* | Cave wall | Israel | 1 | Vanderwolf et al. (2013a) |
| *Dothideomycetes* | *Pleosporales* | *Pleosporaceae* | *Stemphylium vesicarium* | *Stemphylium vesicarium* | Cave wall | China | 1 | Ma et al. (2025) |
| *Dothideomycetes* | *Pleosporales* | *Pleosporaceae* | Ulocladium spp. | Ulocladium spp. | Unreported, insects, air | Belgium, Slovakia, USA | 3 | Vanderwolf et al. (2013a) |
| *Dothideomycetes* | *Pleosporales* | *Pyrenochaetopsidaceae* | *Pyrenochaetopsis decipiens* | *Pyrenochaetopsis decipiens* | Unreported | China | 1 | Zhang (2019) |
| *Dothideomycetes* | *Pleosporales* | *Pyrenochaetopsidaceae* | *Pyrenochaetopsis* spp. | Pyrenochaetopsis spp. | Air, organic litter, soil | China, Thailand | 2 | Jiang et al. (2017a, b) |
| *Dothideomycetes* | *Pleosporales* | *Roussoellaceae* | *Neoroussoella* sp. | *Neoroussoella* sp*.* | Water, | Thailand | 1 | Suetrong et al. (2023) |
| *Dothideomycetes* | *Pleosporales* | *Roussoellaceae* | *Pseudoroussoella* sp. | *Pseudoroussoella* sp. | Air, | Thailand | 1 | Suetrong et al. (2023) |
| *Dothideomycetes* | *Pleosporales* | *Roussoellaceae* | *Roussoella siamensis* | *Roussoella siamensis* | Unreported | China | 1 | Zhang et al. (2021) |
| *Dothideomycetes* | *Pleosporales* | *Roussoellaceae* | *Roussoella* spp. | *Roussoella* spp. | Air, | China, Thailand | 2 | Zhang et al. (2021); Suetrong et al. (2023) |
| *Dothideomycetes* | *Pleosporales* | *Sporormiaceae* | *Forliomyces* sp. | *Forliomyces* sp. | Air | France | 1 | Leplat et al. (2018) |
| *Dothideomycetes* | *Pleosporales* | *Sporormiaceae* | *Preussia aemulans* | *Preussia aemulans* | Unreported | China | 1 | Zhang et al. (2017) |
| *Dothideomycetes* | *Pleosporales* | *Sporormiaceae* | *Preussia funiculata* | *Preussia funiculata* | Bat | Canada | 1 | Vanderwolf et al. (2015); |
| *Dothideomycetes* | *Pleosporales* | *Sporormiaceae* | Preussia spp. | Preussia spp. | Unreported | Canada, China | 2 | Vanderwolf et al. (2013, 2015, 2019) |
| *Dothideomycetes* | *Pleosporales* | *Sporormiaceae* | *Preussia terricola* | *Preussia terricola* | Unreported | China | 1 | Zhang et al. (2021) |
| *Dothideomycetes* | *Pleosporales* | *Sporormiaceae* | *Sporormia subticinensis* | *Sporormia subticinensis* | Unreported | China | 1 | Zhang et al. (2021) |
| *Dothideomycetes* | *Pleosporales* | *Sporormiaceae* | *Sporormiella insignis* | *Sporormiella insignis* | Soil | Romania | 1 | Nováková et al. (2018) |
| *Dothideomycetes* | *Pleosporales* | *Sporormiaceae* | *Sporormiella minima* | *Sporormiella minima* | Unreported | China | 1 | Zhang et al. (2021) |
| *Dothideomycetes* | *Pleosporales* | *Sporormiaceae* | *Sporormiella minimoides* | *Sporormiella minimoides* | Cave wall | Israel | 1 | Vanderwolf et al. (2013a) |
| *Dothideomycetes* | *Pleosporales* | *Sporormiaceae* | *Sporormiella* sp. | *Sporormiella* sp. | Air | Spain | 1 | Vanderwolf et al. (2013a) |
| *Dothideomycetes* | *Pleosporales* | *Teichosporaceae* | *Teichospora* sp. | *Teichospora* sp. | Unreported | China | 1 | Zhang et al. (2021) |
| *Dothideomycetes* | *Pleosporales* | *Telochistaceae* | Telochistaceae sp. | Telochistaceae sp. | Air | Spain | 1 | Vanderwolf et al. (2013a) |
| *Dothideomycetes* | *Pleosporales* | *Thyridariaceae* | Thyridariaceae spp. | Thyridariaceae spp. | Air | China, Thailand | 2 | Jiang et al. (2017); Suetrong et al. (2023) |
| *Dothideomycetes* | *Pleosporales* | *Torulaceae* | *Dendryphion* sp*.* | *Dendryphion* sp*.* | Unreported | Kazakhstan | 1 | Vanderwolf et al. (2013a) |
| *Dothideomycetes* | *Pleosporales* | *Torulaceae* | *Hormiscium* sp*.* | *Hormiscium* sp*.* | Air | Australia | 1 | Vanderwolf et al. (2013a) |
| *Dothideomycetes* | *Pleosporales* | *Torulaceae* | *Torula herbarum* | *Torula herbarum* | Cave wall | China, Israel | 2 | Vanderwolf et al. (2013a); Zhang et al. (2017) |
| *Dothideomycetes* | *Pleosporales* | *Torulaceae* | *Torula* spp. | *Torula* spp. | Air, soil-sediment, other, wood, unreported | Belgium, Brazil, Czech Republic, Italy, Kazakhstan, Spain | 6 | Vanderwolf et al. (2013a); Taylor et al. (2014) |
| *Dothideomycetes* | *Pleosporales* |  | Pleosporales sp. | Pleosporales sp. | Organic litter, sediments | Canada, Thailand, USA | 3 | Connell and Staudigel (2013); Vanderwolf et al. (2013) |
| *Dothideomycetes* | *Trypetheliales* | *Trypetheliaceae* | *Arthopyrenia salicis* | *Arthopyrenia salicis* | Unreported | China | 1 | Zhang et al. 2021 |
| *Dothideomycetes* | *Tubeufiales* | *Tubeufiaceae* | *Helicoma* sp. | *Helicoma* sp. | Air | Spain | 1 | Vanderwolf et al. (2013a) |
| *Dothideomycetes* | *Tubeufiales* | *Tubeufiaceae* | *Helicomyces* sp. | *Helicomyces* sp. | Air, soil-sediment | Russia, Spain | 2 | Vanderwolf et al. (2013a) |
| *Dothideomycetes* | *Tubeufiales* | *Tubeufiaceae* | *Xenosporium berkeleyi* | *Xenosporium berkeleyi* | Soil-sediment, water | Puerto Rico | 1 | Vanderwolf et al. (2013a) |
| *Dothideomycetes* | *Venturiales* | *Venturiaceae* | *Venturia* spp. | *Venturia* spp. | Air | Spain | 1 | Vanderwolf et al. (2013a) |
| *Dothideomycetes* |  |  | Dothideomycetes sp. | Dothideomycetes sp. | Air, water, soil-sediment, Organic litter, | Thailand | 1 | Suetrong et al. (2023) |
| *Eurotiomycetes* | *Arachnomycetales* | *Arachnomycetaceae* | *Arachnomyces glareosus* | *Arachnomyces glareosus* | Cave wall | USA | 1 | Vanderwolf et al. (2013a) |
| *Eurotiomycetes* | *Arachnomycetales* | *Arachnomycetaceae* | *Arachnomyces* spp. | *Arachnomyces* spp. | Cave wall | China, USA | 2 | Vanderwolf et al. (2013a) |
| *Eurotiomycetes* | *Aspergillaceae* | *Aspergillaceae* | *Aspergillus caespitosus* | *Aspergillus caespitosus* | Air, bat guano, other, rock surface, soil-sediment | Brazil, China, Czech Republic, UK, USA | 5 | Taylor et al. (2013, 2014); Vanderwolf et al. (2013a) |
| *Eurotiomycetes* | *Aspergillaceae* | *Aspergillaceae* | *Aspergillus calidoustus* | *Aspergillus calidoustus* | Limestone, soil, cave wall | China, Romania | 2 | Jiang et al. (2017) |
| *Eurotiomycetes* | *Aspergillaceae* | *Aspergillaceae* | *Aspergillus candidus* | *Aspergillus candidus* | Air, bat guano, other, bat internal tissue, soil-sediment, feather, rock surface | Bahamas, Brazil, Canada, China, Mexico, Puerto Rico, Romania, Slovakia, South Africa, Thailand, Ukraine | 11 | Taylor et al. (2013, 2014); Vanderwolf et al. (2013a) |
| *Eurotiomycetes* | *Aspergillaceae* | *Aspergillaceae* | *Aspergillus carbonarius* | *Aspergillus carbonarius* | soil-sediment | Bahamas | 1 | Vanderwolf et al. (2013a) |
| *Eurotiomycetes* | *Aspergillaceae* | *Aspergillaceae* | *Aspergillus dehongensis* | *Aspergillus dehongensis* | Bamboo stick, candle | China | 1 | This study |
| *Eurotiomycetes* | *Chaetothyriales* | *Cyphellophoraceae* | *Candida orthopsilosis* | *Candida orthopsilosis* | Bat | Brazil | 1 | Cunha et al. (2020) |
| *Eurotiomycetes* | *Chaetothyriales* | *Cyphellophoraceae* | *Cyphellophora laciniata* | *Cyphellophora laciniata* | Unreported | Antarctica |  | Connell and Staudigel (2013) |
| *Eurotiomycetes* | *Chaetothyriales* | *Cyphellophoraceae* | *Cyphellophora olivacea* | *Cyphellophora olivacea* | Air, cave wall, soil, sediment | Canada, Czech Republic, Italy, Russia, Slovakia, USA | 6 | Vanderwolf et al. (2013a) |
| *Eurotiomycetes* | *Chaetothyriales* | *Cyphellophoraceae* | *Cyphellophora pluriseptata* | *Cyphellophora pluriseptata* | Cave wall | China | 1 | Ma et al. (2025) |
| *Eurotiomycetes* | *Chaetothyriales* | *Epibryaceae* | *Epibryon minutissimum* | *Cladophialophora minutissima* | Slate precipitate | Germany | 1 | Burow et al. (2019) |
| *Eurotiomycetes* | *Chaetothyriales* | *Herpotrichiellaceae* | *Cadophora fastigiata* | *Phialophora fastigiata* | Air | Czech Republic | 1 | Vanderwolf et al. (2013a) |
| *Eurotiomycetes* | *Chaetothyriales* | *Herpotrichiellaceae* | *Cladophialophora* spp. | *Cladophialophora* spp*.* | Cave wall, earthworm casts, slate precipitate | Germany, Slovakia | 2 | Vanderwolf et al. (2013a); Burow et al. (2019) |
| *Eurotiomycetes* | *Chaetothyriales* | *Herpotrichiellaceae* | *Cyphellophora olivacea* | *Phialophora olivacea* | Cave wall | China, USA | 2 | Vanderwolf et al. (2013a) |
| *Eurotiomycetes* | *Chaetothyriales* | *Herpotrichiellaceae* | *Entimomentora hyalina* | *Phialophora hyalina* | Unreported | Canada | 1 | Vanderwolf et al. (2013a) |
| *Eurotiomycetes* | *Chaetothyriales* | *Herpotrichiellaceae* | *Exophiala castellanii* | *Exophiala castellanii* | Cave wall, sediment | France | 1 | Vanderwolf et al. (2013a) |
| *Eurotiomycetes* | *Chaetothyriales* | *Herpotrichiellaceae* | *Exophiala lecanii-corni* | *Exophiala lecanii-corni* | asymptomatic specimens of Proteus anguinus | Slovenia | 1 | Savković et al. (2025) |
| *Eurotiomycetes* | *Chaetothyriales* | *Herpotrichiellaceae* | *Exophiala moniliae* | *Exophiala moniliae* | Cave wall, sediment | France | 1 | Vanderwolf et al. (2013a) |
| *Eurotiomycetes* | *Chaetothyriales* | *Herpotrichiellaceae* | *Exophiala pisciphila* | *Exophiala pisciphila* | Unreported | China | 1 | Zhang et al. (2021) |
| *Eurotiomycetes* | *Chaetothyriales* | *Herpotrichiellaceae* | *Exophiala salmonis* | *Exophiala salmonis* | Unreported | Bulgaria | 1 | Mitova et al. (2017) |
| *Eurotiomycetes* | *Chaetothyriales* | *Herpotrichiellaceae* | *Exophiala* spp. | *Exophiala* spp. | Air, cave wall, sediment | Bulgaria, Canada, China, Czech Republic, France | 5 | Vanderwolf et al. (2013a) |
| *Eurotiomycetes* | *Chaetothyriales* | *Herpotrichiellaceae* | *Exophiala xenobiotica* | *Exophiala xenobiotica* | Air, water | China, Slovakia | 2 | Ogórek (2018a, b) |
| *Eurotiomycetes* | *Chaetothyriales* | *Herpotrichiellaceae* | *Phialophora cinerescens* | *Phialophora cinerescens* | Soil-sediment | Russia | 1 | Vanderwolf et al. (2013a) |
| *Eurotiomycetes* | *Chaetothyriales* | *Herpotrichiellaceae* | *Phialophora foetens* | *Phialophora foetens* | Unreported | China | 1 | Zhang et al. (2021) |
| *Eurotiomycetes* | *Chaetothyriales* | *Herpotrichiellaceae* | *Phialophora* spp. | *Phialophora* spp. | Cave wall, soil-sediment | Canada, Czech Republic, France, Italy, Russia, Slovakia, USA | 7 | Vanderwolf et al. (2013, 2019); Leplat et al. (2018) |
| *Eurotiomycetes* | *Chaetothyriales* | *Herpotrichiellaceae* | *Phialophora verrucosa* | *Phialophora verrucosa* | Air | Poland | 1 | Pusz et al. (2018) |
| *Eurotiomycetes* | *Chaetothyriales* | *Herpotrichiellaceae* | *Rhinocladiella similis* | *Rhinocladiella similis* | Bat | Brazil | 1 | Cunha et al. (2020) |
| *Eurotiomycetes* | *Chaetothyriales* | *Herpotrichiellaceae* | *Rhinocladiella* sp. | *Rhinocladiella* sp. | Water | Russia | 1 | Vanderwolf et al. (2013a) |
| *Eurotiomycetes* | *Chaetothyriales* | *Herpotrichiellaceae* | *Thysanorea* sp. | *Thysanorea* sp. | Cave wall | France | 1 | Vanderwolf et al. (2013a) |
| *Eurotiomycetes* | *Eurotiales* | *Aspergillaceae* | *Aspergillus aculeatus* | *Aspergillus aculeatus* | Bat guano, leaf litter, rock surface, speleothem, water | China, Malaysia, Mexico | 3 | Zhang et al. (2021) |
| *Eurotiomycetes* | *Eurotiales* | *Aspergillaceae* | *Aspergillus affinis* | *Aspergillus affinis* | Soil | Romania | 1 | Nováková et al. (2018) |
| *Eurotiomycetes* | *Eurotiales* | *Aspergillaceae* | *Aspergillus alboluteus* | *Aspergillus alboluteus* | Sediment | Brazil | 1 | Alves et al. (2022), Savković et al. (2025) |
| *Eurotiomycetes* | *Eurotiales* | *Aspergillaceae* | *Aspergillus alliaceus* | *Aspergillus alliaceus* | Soil | Romania | 1 | Nováková et al. (2018) |
| *Eurotiomycetes* | *Eurotiales* | *Aspergillaceae* | *Aspergillus amstelodami* | *Aspergillus amstelodami* | Soil, bat guano, cave wall | Israel, Italy, Slovakia | 3 | Vanderwolf et al. (2013a) |
| *Eurotiomycetes* | *Eurotiales* | *Aspergillaceae* | *Aspergillus amstelodami* | *Eurotium amstelodami* | Bat guano, soil-sediment, cave wall | Brazil, Israel, Italy, Slovakia | 4 | Taylor et al. (2013); Vanderwolf et al. (2013a) |
| *Eurotiomycetes* | *Eurotiales* | *Aspergillaceae* | *Aspergillus amylovorus* | *Aspergillus amylovorus* | Cave wall | China, Spain | 2 | Vanderwolf et al. (2013a) |
| *Eurotiomycetes* | *Eurotiales* | *Aspergillaceae* | *Aspergillus askiburgiensis* | *Aspergillus askiburgiensis* | Sediment | Czech | 1 | Savković et al. (2025) |
| *Eurotiomycetes* | *Eurotiales* | *Aspergillaceae* | *Aspergillus asperescens* | *Aspergillus asperescens* | Air, cave soil, cave wall, but dung, diplopod faeces, invertebrate faeces | Czech Republic, France, Israel, Poland, Romania, UK | 6 | Vanderwolf et al. (2013a) |
| *Eurotiomycetes* | *Eurotiales* | *Aspergillaceae* | *Aspergillus aureolatus* | *Aspergillus aureolatus* | Bamboo, bat bones, bat guano, rock sufaces, sediment, soil, wood | Brazil, China, Poland, Romania, Russia, USA | 6 | Vanderwolf et al. (2013a); Man (2017); Tavares et al. (2018) |
| *Eurotiomycetes* | *Eurotiales* | *Aspergillaceae* | *Aspergillus aureolus* | *Aspergillus aureolus* | Unreported | China | 1 | Zhang et al. (2021) |
| *Eurotiomycetes* | *Eurotiales* | *Aspergillaceae* | *Aspergillus awamori* | *Aspergillus awamori* | Soil-sediment | Russia | 1 | Vanderwolf et al. (2013a) |
| *Eurotiomycetes* | *Eurotiales* | *Aspergillaceae* | *Aspergillus baeticus* | *Aspergillus baeticus* | Air, cave sediment, rock surface | China, Romania, Spain, Slovakia | 4 | Vanderwolf et al. (2013a); Nováková et al. (2018) |
| *Eurotiomycetes* | *Eurotiales* | *Aspergillaceae* | *Aspergillus bertholletiae* | *Aspergillus bertholletiae* | Cave wall, Air | Sri Lanka, Brazil | 2 | Vanderwolf et al. (2013a); Cunha et al. (2020) |
| *Eurotiomycetes* | *Eurotiales* | *Aspergillaceae* | *Aspergillus brasiliensis* | *Aspergillus brasiliensis* | Air | Spain | 1 | Savković et al. (2025) |
| *Eurotiomycetes* | *Eurotiales* | *Aspergillaceae* | *Aspergillus brunneoviolaceus* | *Aspergillus brunneoviolaceus* | Air,unreported | Brazil, China | 2 | Alves et al. (2022), Zhang et al. (2021) |
| *Eurotiomycetes* | *Eurotiales* | *Aspergillaceae* | *Aspergillus carneus* | *Aspergillus carneus* | Air | China, South Africa | 1 | Vanderwolf et al. (2013a); Zhang et al. (2021) |
| *Eurotiomycetes* | *Eurotiales* | *Aspergillaceae* | *Aspergillus cavernicola* | *Aspergillus cavernicola* | Air,rock surface | China, France | 2 | Zhang et al. (2017) |
| *Eurotiomycetes* | *Eurotiales* | *Aspergillaceae* | *Aspergillus chevalieri* | *Aspergillus chevalieri* | sediment, soil | Italy | 1 | Vanderwolf et al. (2013a) |
| *Eurotiomycetes* | *Eurotiales* | *Aspergillaceae* | *Aspergillus chevalieri* | *Aspergillus chevalieri* var. *intermedius* | soil-sediment | Italy | 1 | Vanderwolf et al. (2013a) |
| *Eurotiomycetes* | *Eurotiales* | *Aspergillaceae* | *Aspergillus chevalieri* | *Eurotium chevalieri* | Air, cave wall, soil-sediment | India, Israel, South Africa | 3 | Vanderwolf et al. (2013a) |
| *Eurotiomycetes* | *Eurotiales* | *Aspergillaceae* | *Aspergillus cibarius* | *Aspergillus cibarius* | Sediment, soil | Spain | 1 | Chen et al. 2017 |
| *Eurotiomycetes* | *Eurotiales* | *Aspergillaceae* | *Aspergillus citocrescens* | *Aspergillus citocrescens* | Sediment | Spain | 1 | Savković et al. (2025) |
| *Eurotiomycetes* | *Eurotiales* | *Aspergillaceae* | *Aspergillus conjunctus* | *Aspergillus conjunctus* | Other | Armenia, Israel | 2 | Vanderwolf et al. (2013a) |
| *Eurotiomycetes* | *Eurotiales* | *Aspergillaceae* | *Aspergillus creber* | *Aspergillus creber* | cave sediment, Air, cave wall | China, Romania | 2 | Zhang et al. (2017); Nováková et al. (2018), |
| *Eurotiomycetes* | *Eurotiales* | *Aspergillaceae* | *Aspergillus cremeus* | *Aspergillus cremeus* | Unreported | Unknow |  | Zhang et al. (2021) |
| *Eurotiomycetes* | *Eurotiales* | *Aspergillaceae* | *Aspergillus cremeus* | *Chaetosartorya cremea* | Unreported | Unknow |  | Vanderwolf et al. (2013a) |
| *Eurotiomycetes* | *Eurotiales* | *Aspergillaceae* | *Aspergillus croceus* | *Aspergillus croceus* | Air, sediment | Spain | 1 | Savković et al. (2025) |
| *Eurotiomycetes* | *Eurotiales* | *Aspergillaceae* | *Aspergillus crustosus* | *Aspergillus crustosus* | Cave wall | Israel | 1 | Vanderwolf et al. (2013a) |
| *Eurotiomycetes* | *Eurotiales* | *Aspergillaceae* | *Aspergillus deflectus* | *Aspergillus deflectus* | Cave wall | China, Israel | 2 | Vanderwolf et al. (2013a); Zhang et al. (2021) |
| *Eurotiomycetes* | *Eurotiales* | *Aspergillaceae* | *Aspergillus dimorphicus* | *Aspergillus dimorphicus* | Air, sediment | Brazil | 1 | Alves et al. (2022), Savković et al. (2025) |
| *Eurotiomycetes* | *Eurotiales* | *Aspergillaceae* | *Aspergillus discophorus* | *Aspergillus discophorus* | Air | Spain | 1 | Savković et al. (2025) |
| *Eurotiomycetes* | *Eurotiales* | *Aspergillaceae* | *Aspergillus dobrogensis* | *Aspergillus dobrogensis* | Sediment | Romania | 1 | Savković et al. (2025) |
| *Eurotiomycetes* | *Eurotiales* | *Aspergillaceae* | *Aspergillus eburneocremeus* | *Aspergillus eburneocremeus* | Sediment | Brazil | 1 | Alves et al. (2022), Savković et al. (2025) |
| *Eurotiomycetes* | *Eurotiales* | *Aspergillaceae* | *Aspergillus elegans* | *Aspergillus elegans* | Air | Slovakia | 1 | Ogórek (2018a, b) |
| *Eurotiomycetes* | *Eurotiales* | *Aspergillaceae* | *Aspergillus europaeus* | *Aspergillus europaeus* | Air, soil, Speleothem | Malaysia, Romania, Spain | 3 | Wasti et al. (2021) |
| *Eurotiomycetes* | *Eurotiales* | *Aspergillaceae* | *Aspergillus fijiensis* | *Aspergillus fijiensis* | Unreported | China | 1 | Zhang et al. (2021) |
| *Eurotiomycetes* | *Eurotiales* | *Aspergillaceae* | *Aspergillus fischeri* | *Aspergillus fischeri* | Sediment, soil | Belgium, Italy | 2 | Vanderwolf et al. (2013a); Zhang et al. (2021) |
| *Eurotiomycetes* | *Eurotiales* | *Aspergillaceae* | *Aspergillus fischeri* | *Neosartorya fischeri* | Soil-sediment | Italy | 1 | Vanderwolf et al. (2013a) |
| *Eurotiomycetes* | *Eurotiales* | *Aspergillaceae* | *Aspergillus flavipes* | *Aspergillus flavipes* | Cave wall, soil-sediment, air | Bahamas, Czech Republic, Russia, South Africa, USA | 5 | Vanderwolf et al. (2013a) |
| *Eurotiomycetes* | *Eurotiales* | *Aspergillaceae* | *Aspergillus flavofurcatus* | *Aspergillus flavofurcatus* | bat guano, bat internal tissue | Mexico | 1 | Vanderwolf et al. (2013a) |
| *Eurotiomycetes* | *Eurotiales* | *Aspergillaceae* | *Aspergillus flavus* | *Aspergillus flavus* | Air, soil-sediment, speleothem, bat guano, other, cave wall, water, wood, bamboo stick, bat carcasse, bat guano, candle, cigarette butt, joss stick, plastic straw | Bahamas, Brazil, Canada, China, Czech Republic, Egypt, France, India, Israel, Korea, Malaysia, Poland, Puerto Rico, Romania, Slovakia, South Africa | 16 | Parkinson et al. (1989); Taylor et al. (2013, 2014); Vanderwolf et al. (2013a); Wasti et al. (2021); Elkhateeb et al. (2022) |
| *Eurotiomycetes* | *Eurotiales* | *Aspergillaceae* | *Aspergillus flavus* | *Aspergillus flavus* var. *oryzae* | Cave wall, air, soil-sediment | China, Israel, Thailand | 3 | Vanderwolf et al. (2013a); zhang et al. (2021) |
| *Eurotiomycetes* | *Eurotiales* | *Aspergillaceae* | *Aspergillus flavus* | *Aspergillus oryzae* | Unknown | China | 1 | Man (2017) |
| *Eurotiomycetes* | *Eurotiales* | *Aspergillaceae* | *Aspergillus foetidus* | *Aspergillus foetidus* | Soil-sediment | Bahamas | 1 | Vanderwolf et al. (2013a) |
| *Eurotiomycetes* | *Eurotiales* | *Aspergillaceae* | *Aspergillus fumigatus* | *Aspergillus fumigatus* | Air, soil-sediment, bat guano, branch, rock other, water, earthworm casts, wood, cave wall | Brazil, Canada, China, Czech Republic, Egypt, France, India, Israel, Italy, Kazakhstan, Nigeria, Poland, Puerto Rico, Romania, Slovakia, South Africa, Spain, Thailand, Ukraine | 20 | Vanderwolf et al. (2013a); Taylor et al. (2014); Yoder et al. (2015); Ogórek et al. (2016b, c, d); Man (2017); Zhang et al. (2017); Dyląg et al. (2019); Elkhateeb et al. (2022) |
| *Eurotiomycetes* | *Eurotiales* | *Aspergillaceae* | *Aspergillus germanicus* | *Aspergillus germanicus* | Air, sediment | Brazil | 1 | Alves et al. (2022) |
| *Eurotiomycetes* | *Eurotiales* | *Aspergillaceae* | *Aspergillus giganteus* | *Aspergillus giganteus* | Air, soil-sediment, other | Poland, Venezuela | 2 | Vanderwolf et al. (2013a) |
| *Eurotiomycetes* | *Eurotiales* | *Aspergillaceae* | *Aspergillus glaucus* | *Aspergillus glaucus* | Air, soil-sediment, cave wall, wood | Armenia, Canada, Czech Republic, Russia, Ukraine | 5 | Vanderwolf et al. (2013a) |
| *Eurotiomycetes* | *Eurotiales* | *Aspergillaceae* | *Aspergillus glaucus* | *Eurotium herbariorum* | Air, cave wall | Israel, South Africa | 2 | Vanderwolf et al. (2013a) |
| *Eurotiomycetes* | *Eurotiales* | *Aspergillaceae* | *Aspergillus granulosus* | *Aspergillus granulosus* | Air | Czech Republic | 1 | Vanderwolf et al. (2013a) |
| *Eurotiomycetes* | *Eurotiales* | *Aspergillaceae* | *Aspergillus hongkongensis* | *Aspergillus hongkongensis* | Unreported | China | 1 | Zhang et al. (2021) |
| *Eurotiomycetes* | *Eurotiales* | *Aspergillaceae* | *Aspergillus humicola* | *Aspergillus humicola* | Other | Czech Republic | 1 | Vanderwolf et al. (2013a) |
| *Eurotiomycetes* | *Eurotiales* | *Aspergillaceae* | *Aspergillus iizukae* | *Aspergillus iizukae* | Soil, rock, eartworm casts | China, Romania | 2 | Man (2017); Nováková et al. (2018) |
| *Eurotiomycetes* | *Eurotiales* | *Aspergillaceae* | *Aspergillus inflatus* | *Aspergillus inflatus* | Unreported | China | 1 | Zhang et al. (2021) |
| *Eurotiomycetes* | *Eurotiales* | *Aspergillaceae* | *Aspergillus inflatus* | *Penicillium inflatum* | Unreported | China | 1 | Zhang et al. (2017) |
| *Eurotiomycetes* | *Eurotiales* | *Aspergillaceae* | *Aspergillus insuetus* | *Aspergillus insuetus* | Air, rock surface | China, Spain | 1 | Vanderwolf et al. (2013a), |
| *Eurotiomycetes* | *Eurotiales* | *Aspergillaceae* | *Aspergillus janus* | *Aspergillus janus* | Cave wall | Israel | 1 | Vanderwolf et al. (2013a) |
| *Eurotiomycetes* | *Eurotiales* | *Aspergillaceae* | *Aspergillus japonicus* | *Aspergillus japonicus* | Air, bat guano, soil-sediment, leaf litter, rock surface, water | Brazil, China, India, Malaysia, Puerto Rico | 5 | Taylor et al. (2013, 2014); Vanderwolf et al. (2013a); Wasti et al. (2021) |
| *Eurotiomycetes* | *Eurotiales* | *Aspergillaceae* | *Aspergillus jensenii* | *Aspergillus jensenii* | Air | China, Romania | 2 | Nováková et al. (2018) |
| *Eurotiomycetes* | *Eurotiales* | *Aspergillaceae* | *Aspergillus kanagawaensis* | *Aspergillus kanagawaensis* | Air | South Africa | 1 | Vanderwolf et al. (2013a) |
| *Eurotiomycetes* | *Eurotiales* | *Aspergillaceae* | *Aspergillus keveii* | *Aspergillus keveii* | Unreported | Brazil | 1 | Tavares et al. (2018) |
| *Eurotiomycetes* | *Eurotiales* | *Aspergillaceae* | *Aspergillus lebretii* | *Aspergillus lebretii* | Air | Brazil | 1 | Savković et al. (2025) |
| *Eurotiomycetes* | *Eurotiales* | *Aspergillaceae* | *Aspergillus limoniformis* | *Aspergillus limoniformis* | Bat guano | China | 1 | zhang et al. (2021) |
| *Eurotiomycetes* | *Eurotiales* | *Aspergillaceae* | *Aspergillus melleus* | *Aspergillus melleus* | Air | Spain | 1 | Savković et al. (2025) |
| *Eurotiomycetes* | *Eurotiales* | *Aspergillaceae* | *Aspergillus movilensis* | *Aspergillus movilensis* | Soil, cave sediment, isopod feces | Romania | 1 | Nováková et al. (2018) |
| *Eurotiomycetes* | *Eurotiales* | *Aspergillaceae* | *Aspergillus neoniger* | *Aspergillus neoniger* | Air | Brazil | 1 | Alves et al. (2022) |
| *Eurotiomycetes* | *Eurotiales* | *Aspergillaceae* | *Aspergillus neoniveus* | *Aspergillus neoniveus* | Air | South Africa | 1 | Taylor et al. (2013); Vanderwolf et al. (2013a) |
| *Eurotiomycetes* | *Eurotiales* | *Aspergillaceae* | *Aspergillus neoniveus* | *Aspergillus niveus* | Air, bat guano, sediment | Brazil, South Africa | 2 | Taylor et al. (2013, 2014); Vanderwolf et al. (2013a) |
| *Eurotiomycetes* | *Eurotiales* | *Aspergillaceae* | *Aspergillus nidulans* | *Aspergillus nidulans* | Water, soil-sediment, air, wood, bat guano | Czech Republic, France, Poland, Puerto Rico, Romania, Slovakia, Thailand, USA, Venezuela | 9 | Vanderwolf et al. (2013a) |
| *Eurotiomycetes* | *Eurotiales* | *Aspergillaceae* | *Aspergillus nidulans* | *Emericella heterothallica* | Soil-sediment | Korea | 1 | Vanderwolf et al. (2013a) |
| *Eurotiomycetes* | *Eurotiales* | *Aspergillaceae* | *Aspergillus nidulans* | *Emericella nidulans* | Bat guano, cave wall, candle, cigarette butt | China, Israel, Slovakia | 3 | Vanderwolf et al. (2013a) |
| *Eurotiomycetes* | *Eurotiales* | *Aspergillaceae* | *Aspergillus niger* | *Aspergillus* cf. *niger* | Air | Brazil | 1 | Alves et al. (2022) |
| *Eurotiomycetes* | *Eurotiales* | *Aspergillaceae* | *Aspergillus niger* | *Aspergillus niger* | Air, bat guano, leaf litter, soil-sediment, speleothem, insects, water, other, cave wall, wood, bat guano, branch, rock surface | Armenia, Brazil, China, Czech Republic, Egypt, France, Greece, India, Israel, Italy, Kazakhstan, Korea, Malaysia, Mexico, Poland, Puerto Rico, Romania, Russia, Slovakia, South Africa, Thailand, Ukraine, USA, Venezuela | 24 | Ogórek et al. (2013, 2014a, b, c, 2016c, d, 2017, 2018); Taylor et al. (2013),Vanderwolf et al. (2013a); Pusz et al. (2014, 2015, 2017); Popović et al. (2015); Yoder et al. (2015); Jiang et al. (2017a, b); Zhang et al. (2017); Ogórek (2018a, b); Wasti et al. (2021); Elkhateeb et al. (2022) |
| *Eurotiomycetes* | *Eurotiales* | *Aspergillaceae* | *Aspergillus niger* | *Aspergillus phoenicis* | Soil-sediment, earthworm casts | Slovakia | 1 | Vanderwolf et al. (2013a) |
| *Eurotiomycetes* | *Eurotiales* | *Aspergillaceae* | *Aspergillus niger* | *Aspergillus welwitschiae* | Air | Spain | 1 | Savković et al. (2025) |
| *Eurotiomycetes* | *Eurotiales* | *Aspergillaceae* | *Aspergillus niveoglaucus* | *Aspergillus niveoglaucus* | Organic litter | China | 1 | Zhang et al. (2017) |
| *Eurotiomycetes* | *Eurotiales* | *Aspergillaceae* | *Aspergillus nomius* | *Aspergillus nomius* | Bat guano, speleothem, water | China, Malaysia | 2 | Wasti et al. (2021); Zhang et al. (2021) |
| *Eurotiomycetes* | *Eurotiales* | *Aspergillaceae* | *Aspergillus ochraceus* | *Aspergillus ochraceus* | Air, bat guano, other, soil-sediment, wood | Canada, Bahamas, Brazil, Poland, Czech Republic, Romania, India, South Africa, Spain | 9 | Taylor et al. (2013, 2014); Vanderwolf et al. (2013a); Cunha et al. (2020) |
| *Eurotiomycetes* | *Eurotiales* | *Aspergillaceae* | *Aspergillus okavangoensis* | *Aspergillus okavangoensis* | Bat guano contaminated soil | Botswana | 1 | Savković et al. (2025) |
| *Eurotiomycetes* | *Eurotiales* | *Aspergillaceae* | *Aspergillus pallidofulvus* | *Aspergillus pallidofulvus* | Air | Czech Republic | 1 | Savković et al. (2025) |
| *Eurotiomycetes* | *Eurotiales* | *Aspergillaceae* | *Aspergillus parasiticus* | *Aspergillus parasiticus* | Air, soil-sediment, cave wall, bat guano, soil | China, Czech Republic, India, Puerto Rico, South Africa | 5 | Vanderwolf et al. (2013a) |
| *Eurotiomycetes* | *Eurotiales* | *Aspergillaceae* | *Aspergillus penicillioides* | *Aspergillus penicillioides* | Cave wal | Brazil, France, Israel | 3 | Vanderwolf et al. (2013a) |
| *Eurotiomycetes* | *Eurotiales* | *Aspergillaceae* | *Aspergillus persii* | *Aspergillus persii* | Unreported | China | 1 | Zhang et al. (2021) |
| *Eurotiomycetes* | *Eurotiales* | *Aspergillaceae* | *Aspergillus phialiformis* | *Aspergillus phialiformis* | Rock | China | 1 | zhang et al. (2021) |
| *Eurotiomycetes* | *Eurotiales* | *Aspergillaceae* | *Aspergillus phialosimplex* | *Aspergillus phialosimplex* | animal faeces, plant debris and root | China | 1 | zhang et al. (2021) |
| *Eurotiomycetes* | *Eurotiales* | *Aspergillaceae* | *Aspergillus polyporicola* | *Aspergillus polyporicola* | Unreported | China | 1 | Zhang et al. (2021) |
| *Eurotiomycetes* | *Eurotiales* | *Aspergillaceae* | *Aspergillus pragensis* | *Aspergillus pragensis* | Bat guano, rock | China | 1 | Man (2017); Zhang et al. (2017) |
| *Eurotiomycetes* | *Eurotiales* | *Aspergillaceae* | *Aspergillus proliferans* | *Aspergillus proliferans* | Air | UK | 1 | Vanderwolf et al. (2013a) |
| *Eurotiomycetes* | *Eurotiales* | *Aspergillaceae* | *Aspergillus protuberus* | *Aspergillus protuberus* | Air,bat guano, candle, soil | China, Romania | 2 | Nováková et al. (2018) |
| *Eurotiomycetes* | *Eurotiales* | *Aspergillaceae* | *Aspergillus pseudodeflectus* | *Aspergillus pseudodeflectus* | Rock surface, soil | China, Romania | 2 | Nováková et al. (2018) |
| *Eurotiomycetes* | *Eurotiales* | *Aspergillaceae* | *Aspergillus pseudoglaucus* | *Aspergillus pseudoglaucus* | Air, isopod feces, water, rock, bat guano | China, Poland, Romania | 3 | Nováková et al. (2018) |
| *Eurotiomycetes* | *Eurotiales* | *Aspergillaceae* | *Aspergillus pulvericola* | *Aspergillus pulvericola* | Air | Spain | 1 | Savković et al. (2025) |
| *Eurotiomycetes* | *Eurotiales* | *Aspergillaceae* | *Aspergillus puniceus* | *Aspergillus puniceus* | Bamboo stick, candle, cigarette butt, Earthworm casts, other | Armenia, China, Slovakia | 3 | Vanderwolf et al. (2013a) |
| *Eurotiomycetes* | *Eurotiales* | *Aspergillaceae* | *Aspergillus puulaauensis* | *Aspergillus puulaauensis* | Air, microbial mat | Romania | 1 | Nováková et al. (2018) |
| *Eurotiomycetes* | *Eurotiales* | *Aspergillaceae* | *Aspergillus repens* | *Aspergillus repens* | Unreported | China | 1 | Zhang et al. (2021) |
| *Eurotiomycetes* | *Eurotiales* | *Aspergillaceae* | *Aspergillus reptans* | *Aspergillus reptans* | Soil, wood, sediment | China, France, Italy | 3 | Vanderwolf et al. (2013a); |
| *Eurotiomycetes* | *Eurotiales* | *Aspergillaceae* | *Aspergillus restrictus* | *Aspergillus restrictus* | Air, bat guano, other, soil-sediment | Brazil, Canada, Poland, Russia | 4 | Taylor et al. (2013); Vanderwolf et al. (2013a) |
| *Eurotiomycetes* | *Eurotiales* | *Aspergillaceae* | *Aspergillus ruber* | *Aspergillus ruber* | Air, organic litter | China | 1 | Zhang et al. (2017) |
| *Eurotiomycetes* | *Eurotiales* | *Aspergillaceae* | *Aspergillus rugulosus* | *Aspergillus rugulosus* | Unreported | Brazil | 1 | Taylor et al. (2013) |
| *Eurotiomycetes* | *Eurotiales* | *Aspergillaceae* | *Aspergillus rugulosus* | *Emericella rugulosa* | Air, bat guano, soil-sediment | Bahamas, Brazil, India | 3 | Taylor et al. (2013); Vanderwolf et al. (2013a) |
| *Eurotiomycetes* | *Eurotiales* | *Aspergillaceae* | *Aspergillus sclerotiorum* | *Aspergillus sclerotiorum* | Air, bat guano, soil-sediment, other, soil, water | Brazil, China, Poland, USA | 4 | Taylor et al. (2013, 2014); Vanderwolf et al. (2013a) |
| *Eurotiomycetes* | *Eurotiales* | *Aspergillaceae* | *Aspergillus sesamicola* | *Aspergillus cf. sesamicola* | Air | Brazil | 1 | Cunha et al. (2020) |
| *Eurotiomycetes* | *Eurotiales* | *Aspergillaceae* | *Aspergillus silvaticus* | *Aspergillus silvaticus* | Cave wall, other | Armenia, Czech Republic | 2 | Vanderwolf et al. (2013a) |
| *Eurotiomycetes* | *Eurotiales* | *Aspergillaceae* | Aspergillus sp. 1 section Aspergillus | Aspergillus sp. 1 section Aspergillus | Bat | Brazil | 1 | Cunha et al. (2020) |
| *Eurotiomycetes* | *Eurotiales* | *Aspergillaceae* | *Aspergillus* sp. 1 section *Nigri* | *Aspergillus* sp. 1 section *Nigri* | Air, bat | Brazil, Poland | 2 | Kokurewicz et al. (2016); Cunha et al. (2020) |
| *Eurotiomycetes* | *Eurotiales* | *Aspergillaceae* | Aspergillus sp. 2 section Aspergillus | Aspergillus sp. 2 section Aspergillus | Air | Brazil | 1 | Alves et al. (2022) |
| *Eurotiomycetes* | *Eurotiales* | *Aspergillaceae* | *Aspergillus* sp. sect. *Candidi* | *Aspergillus* sp. sect. *Candidi* | Underwater sediment of Airbell II | Romania | 1 | Nováková et al. (2018) |
| *Eurotiomycetes* | *Eurotiales* | *Aspergillaceae* | *Aspergillus* sp. section *Flavi* | *Aspergillus* sp. section *Flavi* | Air | Poland | 1 | Kokurewicz et al. (2016) |
| *Eurotiomycetes* | *Eurotiales* | *Aspergillaceae* | *Aspergillus* sp. section *Fumigati* | *Aspergillus* sp. section *Fumigati* | Air | Poland | 1 | Kokurewicz et al. (2016) |
| *Eurotiomycetes* | *Eurotiales* | *Aspergillaceae* | *Aspergillus* sp. section *Nidulantes* | *Aspergillus* sp. section *Nidulantes* | Air | Brazil | 1 | Cunha et al. (2020) |
| *Eurotiomycetes* | *Eurotiales* | *Aspergillaceae* | *Aspergillus* sp. section *Polypaecilum* | *Aspergillus* sp. section *Polypaecilum* | Bat guano | Brazil | 1 | Cunha et al. (2020) |
| *Eurotiomycetes* | *Eurotiales* | *Aspergillaceae* | *Aspergillus* sp.2 section *Nigri* | *Aspergillus* sp. 2 section *Nigri* | Bat | Brazil | 1 | Cunha et al. (2020) |
| *Eurotiomycetes* | *Eurotiales* | *Aspergillaceae* | *Aspergillus spelaeus* | *Aspergillus spelaeus* | Air, sediment | Brazil, Spain | 2 | Savković et al. (2025) |
| *Eurotiomycetes* | *Eurotiales* | *Aspergillaceae* | *Aspergillus spelunceus* | *Aspergillus spelunceus* | Air, cave wall, insects, soil, sediment | China, Czech Republic, Iran, Romania, Spain, USA | 6 | Vanderwolf et al. (2013a); Zhang et al. (2017) |
| *Eurotiomycetes* | *Eurotiales* | *Aspergillaceae* | *Aspergillus* spp. | *Aspergillus* spp. | Air, soil, cave wall, cave sediment, insect, bat guano, bat internal tissue, dung, frass (not bat); wood, Periplaneta americana, speleothem, Trigonilus corallinus | Australia, Belgium, Brazil, China, Czech Republic, France, Germany, Greece, India, Italy, Japan, Kazakhstan, Korea, Malaysia, Mexico, Poland, Puerto Rico, Romania, Slovakia, Slovenia, South Africa, Spain, Thailand, UK, USA | 25 | Wang et al. (2010); Taylor et al. (2013); Vanderwolf et al. (2013a); Kokurewicz et al. (2016); Jiang et al. (2017); Man (2017); Nováková et al. (2018); Wasti et al. (2021); Zhang et al. (2021); Alves et al. (2022) |
| *Eurotiomycetes* | *Eurotiales* | *Aspergillaceae* | *Aspergillus stellatus* | *Aspergillus stellatus* | Air, sediment | Brazil, China | 2 | Alves et al. (2022); Zhang et al. (2021) |
| *Eurotiomycetes* | *Eurotiales* | *Aspergillaceae* | *Aspergillus subalbidus* | *Aspergillus subalbidus* | Bat guano contaminated soil, feather | Botswana, China | 2 | Savković et al. (2025), |
| *Eurotiomycetes* | *Eurotiales* | *Aspergillaceae* | *Aspergillus sulphureus* | *Aspergillus sulphureus* | Bat internal tissue, soil-sediment, air | Bahamas, Mexico, Poland | 3 | Vanderwolf et al. (2013a) |
| *Eurotiomycetes* | *Eurotiales* | *Aspergillaceae* | *Aspergillus sydowii* | *Aspergillus sydowii* | Air, bat internal tissue, bat guano, unreported, limestone, , cave wall, other, water, wood, soil-sediment | Armenia, Brazil, China, Czech Republic, India, Italy, Malaysia, Mexico, Romania, Spain | 10 | Taylor et al. (2013); Vanderwolf et al. (2013a); Jiang et al. (2017); Cunha et al. (2020); Wasti et al. (2021); Alves et al. (2022) |
| *Eurotiomycetes* | *Eurotiales* | *Aspergillaceae* | *Aspergillus tabacinus* | *Aspergillus tabacinus* | Air | Romania | 1 | Nováková et al. (2018) |
| *Eurotiomycetes* | *Eurotiales* | *Aspergillaceae* | *Aspergillus taichungensis* | *Aspergillus taichungensis* | Bat guano contaminated soil | China, Botswana | 1 | Savković et al. (2025) |
| *Eurotiomycetes* | *Eurotiales* | *Aspergillaceae* | *Aspergillus tamarii* | *Aspergillus tamarii* | Air, other, cave wall, soil-sediment | China (Taiwan Province); Czech Republic, India, Poland, USA | 5 | Vanderwolf et al. (2013a) |
| *Eurotiomycetes* | *Eurotiales* | *Aspergillaceae* | *Aspergillus templicola* | *Aspergillus templicola* | Soil | Romania | 1 | Nováková et al. (2018) |
| *Eurotiomycetes* | *Eurotiales* | *Aspergillaceae* | *Aspergillus tennesseensis* | *Aspergillus tennesseensis* | Dead spiders | China, Romania | 2 | Zhang et al. (2017); Nováková et al. (2018) |
| *Eurotiomycetes* | *Eurotiales* | *Aspergillaceae* | *Aspergillus terreus* | *Aspergillus terreus* | Air, bat guano, bat internal tissue, soil-sediment, Bamboo, other, cave wall | China, Czech Republic, India, Israel, Mexico, Poland, South Africa, USA, Venezuela | 9 | Vanderwolf et al. (2013a) |
| *Eurotiomycetes* | *Eurotiales* | *Aspergillaceae* | *Aspergillus thesauricus* | *Aspergillus thesauricus* | Air, soil, sediment, bat guano, dead isopod bodies | China, Romania, Spain | 3 | Vanderwolf et al. (2013a); Zhang et al. (2017); Nováková et al. (2018) |
| *Eurotiomycetes* | *Eurotiales* | *Aspergillaceae* | *Aspergillus tubingensis* | *Aspergillus tubingensis* | Air, rock, sediment | Brazil, China, Spain | 3 | Zhang et al. (2017); Cunha et al. (2020); Alves et al. (2022) |
| *Eurotiomycetes* | *Eurotiales* | *Aspergillaceae* | *Aspergillus unguis* | *Aspergillus unguis* | Rock surface, soil-sediment, other | Bahamas, China, Czech Republic, Italy | 4 | Vanderwolf et al. (2013a); Man (2017) |
| *Eurotiomycetes* | *Eurotiales* | *Aspergillaceae* | *Aspergillus ustus* | *Aspergillus ustus* | Water, soil-sediment, cave wall, air, wood, other | Armenia, Bahamas, Brazil, China, Czech Republic, Israel, Poland, Romania, Russia, Slovakia, South Africa, Spain, Thailand, USA | 14 | Taylor et al. (2013); Vanderwolf et al. (2013a) |
| *Eurotiomycetes* | *Eurotiales* | *Aspergillaceae* | *Aspergillus venenatus* | *Aspergillus venenatus* | Cave wal | China | 1 | Ma et al. (2025) |
| *Eurotiomycetes* | *Eurotiales* | *Aspergillaceae* | *Aspergillus wentii* | *Aspergillus wentii* | Air, cave wall, bat guano, soil-sediment, water | Bahamas, Brazil, China (Taiwan Province); India, Israel, Poland, Russia, USA | 8 | Taylor et al. (2013); Vanderwolf et al. (2013a); Cunha et al. (2020) |
| *Eurotiomycetes* | *Eurotiales* | *Aspergillaceae* | *Aspergillus westerdijkiae* | *Aspergillus westerdijkiae* | Air, cave wal | Brazil, China, Romania | 3 | Nováková et al. (2018); Cunha et al. (2020); Ma et al. (2025) |
| *Eurotiomycetes* | *Eurotiales* | *Aspergillaceae* | *Emericella* sp. | *Emericella* sp. | Air | Spain | 1 | Vanderwolf et al. (2013a) |
| *Eurotiomycetes* | *Eurotiales* | *Aspergillaceae* | *Emericella versicolor* | *Aspergillus versicolor* | Air, soil-sediment, water, bat guano, bat internal tissue, unreported, cave wall, other, insects, limestone, soil, wood | Armenia, Bahamas, Brazil, Canada, China, Czech Republic, Egypt, France, India, Israel, Italy, Korea, Mexico, Poland, Puerto Rico, Romania, Russia, Slovakia, South Africa, Spain, Thailand, UK, Ukraine, USA, Venezuela | 25 | Vanderwolf et al. (2013a); Taylor et al. (2013, 2014); Jiang et al. (2017); Man (2017); Elkhateeb et al. (2022); Ma et al. (2025) |
| *Eurotiomycetes* | *Eurotiales* | *Aspergillaceae* | *Eupenicillium* sp. | *Eupenicillium* sp. | Earthworm casts | Slovakia | 1 | Vanderwolf et al. (2013a) |
| *Eurotiomycetes* | *Eurotiales* | *Aspergillaceae* | *Eurotium* spp. | *Eurotium* spp. | Air, soil, sediment | China, Spain, Slovakia, South Africa | 4 | Vanderwolf et al. (2013a); |
| *Eurotiomycetes* | *Eurotiales* | *Aspergillaceae* | *Hamigera ingelheimensis* | *Hamigera ingelheimensis* | Bat guano, soil-sediment | France, Czech Republic | 2 | Vanderwolf et al. (2013a) |
| *Eurotiomycetes* | *Eurotiales* | *Aspergillaceae* | *Hamigera ingelheimensis* | *Penicillium ingelheimense* | Bat guano, soil-sediment | France, Czech Republic | 2 | Vanderwolf et al. (2013a) |
| *Eurotiomycetes* | *Eurotiales* | *Aspergillaceae* | *Marquandomyces marquandii* | *Paecilomyces marquandii* | Soil-sediment, other, bat guano, earthworm casts, insects, cave wall | Bahamas, China, Czech Republic, Italy, Poland, Russia, Slovakia, Spain | 8 | Vanderwolf et al. (2013a); Man (2017); Wasti et al. (2021) |
| *Eurotiomycetes* | *Eurotiales* | *Aspergillaceae* | *Neosartorya clavata* | *Aspergillus clavatus* | bat guano, cave wall, wood, soil-sediment | Brazil, Czech Republic, Ireland, Mexico, Russia, Serbia, Slovakia | 7 | Taylor et al. (2013); Vanderwolf et al. (2013a) |
| *Eurotiomycetes* | *Eurotiales* | *Aspergillaceae* | *Paecilomyces divaricatus* | *Paecilomyces divaricatus* | Wood | Romania | 1 | Vanderwolf et al. (2013a) |
| *Eurotiomycetes* | *Eurotiales* | *Aspergillaceae* | *Paecilomyces formosus* | Paecilomyces cf. formosus | Bat guano | Brazil | 1 | Cunha et al. (2020) |
| *Eurotiomycetes* | *Eurotiales* | *Aspergillaceae* | *Paecilomyces fulvus* | *Byssochlamys fulva* | Soil-sediment | USA | 1 | Vanderwolf et al. (2013a) |
| *Eurotiomycetes* | *Eurotiales* | *Aspergillaceae* | *Paecilomyces* spp. | Byssochlamys spp. | Soil-sediment | China (Taiwan Province); USA | 2 | Vanderwolf et al. (2013a) |
| *Eurotiomycetes* | *Eurotiales* | *Aspergillaceae* | *Paecilomyces* spp. | *Paecilomyces* spp. | Air, , insects, cave wall, bat guano, soil-sediment, wood | Australia, Belgium, Brazil, Canada, China, Czech Republic, France, India, Japan, Puerto Rico, Slovakia, Thailand, UK, USA | 14 | Vanderwolf et al. (2013, 2015, 2019); Man et al. (2015); Man (2017) |
| *Eurotiomycetes* | *Eurotiales* | [*Aspergillaceae*](https://www.indexfungorum.org/Names/families.asp?FamilyName=Aspergillaceae) | *Paecilomyces tenuis* | *Paecilomyces tenuis* | Unreported | China, USA | 2 | Zhang et al. (2021) |
| *Eurotiomycetes* | *Eurotiales* | *Aspergillaceae* | *Paecilomyces variotii* | *Paecilomyces variotii* | Air, soil-sediment, other, bat guano, earthworm casts, wood | Brazil, France, Malaysia, Poland, Romania, Slovakia, South Africa, USA | 8 | Vanderwolf et al. (2013a); Taylor et al. (2014); Kokurewicz et al. (2016) |
| *Eurotiomycetes* | *Eurotiales* | *Aspergillaceae* | Paecilomyces variotii complex | Paecilomyces variotii complex | Air | Poland | 1 | Kokurewicz et al. (2016) |
| *Eurotiomycetes* | *Eurotiales* | *Aspergillaceae* | *Penicillium adametzii* | *Penicillium adametzii* | Wood | Czech Republic | 1 | Vanderwolf et al. (2013a) |
| *Eurotiomycetes* | *Eurotiales* | *Aspergillaceae* | *Penicillium adametzioides* | *Penicillium adametzioides* | Soil | China | 1 | Zhang et al. (2021) |
| *Eurotiomycetes* | *Eurotiales* | *Aspergillaceae* | *Penicillium aeneum* | *Penicillium aeneum* | Unreported | China | 1 | Zhang et al. (2021) |
| *Eurotiomycetes* | *Eurotiales* | *Aspergillaceae* | *Penicillium albidum* | *Penicillium albidum* | Cave wall, soil-sediment | Czech Republic, Russia | 2 | Vanderwolf et al. (2013a) |
| *Eurotiomycetes* | *Eurotiales* | *Aspergillaceae* | *Penicillium astrolabium* | *Penicillium astrolabium* | Unreported | China | 1 | Zhang et al. (2021) |
| *Eurotiomycetes* | *Eurotiales* | *Aspergillaceae* | *Penicillium atramentosum* | *Penicillium atramentosum* | Air, earthworm casts, soil | China, France, Romania, Slovakia | 4 | Vanderwolf et al. (2013a); Zhang et al. (2021) |
| *Eurotiomycetes* | *Eurotiales* | *Aspergillaceae* | *Penicillium atrosanguineum* | *Penicillium atrosanguineum* | Unreported | China, Romania | 2 | Nováková et al. (2018); Zhang et al. (2021) |
| *Eurotiomycetes* | *Eurotiales* | *Aspergillaceae* | *Penicillium atrosanguineum* | *Penicillium manginii* | Sediment, isopod feces | Romania | 1 | Ogórek et al. (2018) |
| *Eurotiomycetes* | *Eurotiales* | *Aspergillaceae* | *Penicillium aurantiacobrunneum* | *Penicillium aurantiacobrunneum* | Air | Slovakia | 1 | Savković et al. (2025) |
| *Eurotiomycetes* | *Eurotiales* | *Aspergillaceae* | *Penicillium aurantiogriseum* | *Penicillium aurantiogriseum* | Air, Insects, other, bat guano, earthworm casts, cave wall, soil-sediment | Belgium, Bulgaria, Czech Republic, Israel, Romania, Russia, Poland, Slovakia | 8 | Vanderwolf et al. (2013a); Mitova et al. (2017); Nováková et al. (2018) |
| *Eurotiomycetes* | *Eurotiales* | *Aspergillaceae* | *Penicillium aurantiogriseum* | *Penicillium aurantiovirens* | Air, soil-sediment | Russia, Spain | 1 | Vanderwolf et al. (2013a) |
| *Eurotiomycetes* | *Eurotiales* | *Aspergillaceae* | *Penicillium aurantiogriseum* | *Penicillium cyclopium* | Cave wall, soil-sediment, air, other, wood | USA, Italy, Poland, Czech Republic, Romania, Russia, Kazakhstan, Korea, India | 9 | Vanderwolf et al. (2013a) |
| *Eurotiomycetes* | *Eurotiales* | *Aspergillaceae* | *Penicillium aurantiogriseum* | *Penicillium lanosocoeruleum* | Soil-sediment | Italy | 1 | Vanderwolf et al. (2013a) |
| *Eurotiomycetes* | *Eurotiales* | *Aspergillaceae* | *Penicillium aurantiogriseum* | *Penicillium viridicatum* | Air, soil-sediment, other, cave wall, wood | Italy, Poland, Czech Republic, Slovakia, Romania, Kazakhstan | 6 | Vanderwolf et al. (2013a) |
| *Eurotiomycetes* | *Eurotiales* | *Aspergillaceae* | Penicillium aurantiogriseum var. aurantiogriseum | Penicillium aurantiogriseum var. aurantiogriseum | Air, soil-sediment, water, cave wall, other, wood, unreported | Italy, Poland, Czech Republic, Russia, Kazakhstan | 5 | Vanderwolf et al. (2013a) |
| *Eurotiomycetes* | *Eurotiales* | *Aspergillaceae* | *Penicillium bialowiezense* | *Penicillium bialowiezense* | Asymptomatic specimens of Proteus anguinus | Slovenia | 1 | Savković et al. (2025) |
| *Eurotiomycetes* | *Eurotiales* | *Aspergillaceae* | *Penicillium bilaiae* | *Penicillium bilaiae* | Bat guano, soil | China, Malaysia, USSR | 3 | Wasti et al. (2021); Zhang et al. (2021) |
| *Eurotiomycetes* | *Eurotiales* | *Aspergillaceae* | *Penicillium biourgeianum* | *Penicillium biourgeianum* | Air | Poland, China, Slovakia | 3 | Pusz et al. (2017); Zhang et al. (2021) |
| *Eurotiomycetes* | *Eurotiales* | *Aspergillaceae* | *Penicillium brasilianum* | *Penicillium brasilianum* | Sediment | Brazil, China, Romania | 3 | Nováková et al. (2018) |
| *Eurotiomycetes* | *Eurotiales* | *Aspergillaceae* | *Penicillium brevicompactum* | *Penicillium brevicompactum* | Air, bat guano, soil-sediment, insects, water, cave wall, limestone, other, wood, unreported | Belgium, Brazil, Canada, China, Czech Republic, Iran, Israel, Italy, Kazakhstan, Korea, MexicoNorth America, Poland, Romania, Russia, Slovakia, South Africa, Spain, Ukraine | 20 | Taylor et al. (2013); Vanderwolf et al. (2013a); Man (2017); Jiang et al. (2017); Zhang et al. (2021) |
| *Eurotiomycetes* | *Eurotiales* | *Aspergillaceae* | *Penicillium brevistipitatum* | *Penicillium brevistipitatum* | Scoliopteryx libatrix | Canada | 1 | Visagie et al. (2019) |
| *Eurotiomycetes* | *Eurotiales* | *Aspergillaceae* | *Penicillium buchwaldii* | *Penicillium buchwaldii* | Unreported | China | 1 | Zhang et al. (2017) |
| *Eurotiomycetes* | *Eurotiales* | *Aspergillaceae* | *Penicillium bussumense* | *Penicillium bussumense* | Unreported | China | 1 | Zhang et al. (2021) |
| *Eurotiomycetes* | *Eurotiales* | *Aspergillaceae* | *Penicillium cairnsense* | *Penicillium cairnsense* | Unreported | China | 1 | Zhang (2019) |
| *Eurotiomycetes* | *Eurotiales* | *Aspergillaceae* | *Penicillium camemberti* | *Penicillium camemberti* | Unreported, cave wall, soil-sediment | Australia, China, Israel, India, Poland, Slovakia | 6 | Vanderwolf et al. (2013a); Zhang et al. (2017) |
| *Eurotiomycetes* | *Eurotiales* | *Aspergillaceae* | *Penicillium canariense* | *Penicillium canariense* | Unreported | China | 1 | Zhang et al. (2021) |
| *Eurotiomycetes* | *Eurotiales* | *Aspergillaceae* | *Penicillium canescens* | *Penicillium canescens* | Insects, other, cave wall, soil-sediment | Belgium, Czech Republic, Romania, Russia | 4 | Vanderwolf et al. (2013a); Nováková et al. (2018) |
| *Eurotiomycetes* | *Eurotiales* | *Aspergillaceae* | *Penicillium capsulatum* | *Penicillium capsulatum* | Air | UK | 1 | Vanderwolf et al. (2013a) |
| *Eurotiomycetes* | *Eurotiales* | *Aspergillaceae* | *Penicillium catenatum* | *Penicillium tularense* | Bat | USA | 1 | Zhang et al. (2014) |
| *Eurotiomycetes* | *Eurotiales* | *Aspergillaceae* | *Penicillium cavernicola* | *Penicillium cavernicola* | Cave wall | New Mexico, USA | 3 | Vanderwolf et al. (2013a) |
| *Eurotiomycetes* | *Eurotiales* | *Aspergillaceae* | *Penicillium chalabudae* | *Penicillium chalabudae* | Wood | China | 1 | Zhang et al. (2021) |
| *Eurotiomycetes* | *Eurotiales* | *Aspergillaceae* | *Penicillium chermesinum* | *Penicillium chermesinum* | Air, soil-sediment, other | Poland, Italy, Poland, Czech Republic, Russia, Spain | 6 | Vanderwolf et al. (2013a) |
| *Eurotiomycetes* | *Eurotiales* | *Aspergillaceae* | *Penicillium chrysogenum* | *Penicillium chrysogenum* | Air, limestone, , soil-sediment, cave wall, bat guano, insects, other, unreported, water, earthworm casts, wood, soil | Belgium, Brazil, Canada, China, Czech Republic, Iran, Italy, Kazakhstan, Korea, Mexico, Poland, Puerto Rico, Romania, Russia, Slovakia, Slovenia, Spain, Thailand, Ukraine, USA | 20 | Taylor et al. (2013, 2014); Vanderwolf et al. (2013a); Man (2017); Jiang et al. (2017); Nováková et al. (2018); Zhang et al. (2021) |
| *Eurotiomycetes* | *Eurotiales* | *Aspergillaceae* | *Penicillium citreonigrum* | *Penicillium citreonigrum* | Soil-sediment, cave wall, other, wood, air | Australia, Canada, Czech Republic, Italy, China, Romania, India, Poland | 8 | Vanderwolf et al. (2013a); Pusz et al. (2014, 2018b) |
| *Eurotiomycetes* | *Eurotiales* | *Aspergillaceae* | *Penicillium citreosulfuratum* | *Penicillium citreosulfuratum* | Air | China, Spain | 2 | Savković et al. (2025) |
| *Eurotiomycetes* | *Eurotiales* | *Aspergillaceae* | *Penicillium citrinum* | *Penicillium citrinum* | Air, bat guano, other, soil-sediment, insects, earthworm casts, cave wall, water, bat guano, cigarette butt | Brazil, China, Czech Republic, Kazakhstan, Israel, Malaysia, Mexico, Poland, Slovakia, Slovenia,, South Africa | 10 | Taylor et al. (2013); Vanderwolf et al. (2013a); Cunha et al. (2020); Wasti et al. (2021); Alves et al. (2022) |
| *Eurotiomycetes* | *Eurotiales* | *Aspergillaceae* | *Penicillium citrinum* | *Penicillium steckii* | Soil-sediment, cave wall | USA, Italy, Israel | 3 | Vanderwolf et al. (2013a) |
| *Eurotiomycetes* | *Eurotiales* | *Aspergillaceae* | *Penicillium commune* | *Penicillium commune* | Soil, water, Air, bat guano, sediment, environmental swab, paintings, rock sufaces | Belgium, Bulgaria, China, Czech Republic, Israel, Poland, Romania, Russia, Slovakia | 10 | Vanderwolf et al. (2013a) Taylor et al. (2014); Man (2017); Zhang et al. (2021) |
| *Eurotiomycetes* | *Eurotiales* | *Aspergillaceae* | *Penicillium concentricum* | *Penicillium concentricum* | Sediment, bat guano | Bulgaria, Canada, China, Mexico, USA | 5 | Vanderwolf et al. (2013a); Zhang et al. (2021) |
| *Eurotiomycetes* | *Eurotiales* | *Aspergillaceae* | *Penicillium consobrinum* | *Penicillium consobrinum* | Cave wall | Canada | 1 | Visagie et al. (2019) |
| *Eurotiomycetes* | *Eurotiales* | *Aspergillaceae* | *Penicillium contaminatum* | *Penicillium contaminatum* | Unreported | Unknow |  | Zhang (2019) |
| *Eurotiomycetes* | *Eurotiales* | *Aspergillaceae* | *Penicillium coprobium* | *Penicillium coprobium* | Unreported | China | 1 | Zhang et al. (2021) |
| *Eurotiomycetes* | *Eurotiales* | *Aspergillaceae* | *Penicillium coprophilum* | *Penicillium coprophilum* | Bat guano, paintings | Bulgaria, China | 2 | Mitova et al. (2017); Man (2017); Zhang et al. (2017, 2021) |
| *Eurotiomycetes* | *Eurotiales* | *Aspergillaceae* | *Penicillium copticola* | *Penicillium copticola* | Sediment, soil | Brazil, China, Mexico | 3 | Zhang et al. (2021); Alves et al. (2022) |
| *Eurotiomycetes* | *Eurotiales* | *Aspergillaceae* | *Penicillium corylophilum* | *Penicillium corylophilum* | Air, soil-sediment, cave wall, bat guano | Canada, China, Poland, Romania, Russia, Slovakia, South Africa, Spain | 8 | Vanderwolf et al. (2013a); Nováková et al. (2018); Visagie et al. (2019) |
| *Eurotiomycetes* | *Eurotiales* | *Aspergillaceae* | *Penicillium crustaceum* | *Eupenicillium crustaceum* | Soil-sediment | Russia | 1 | Vanderwolf et al. (2013a) |
| *Eurotiomycetes* | *Eurotiales* | *Aspergillaceae* | *Penicillium daleae* | *Penicillium daleae* | Bat guano | China, Slovakia | 1 | Vanderwolf et al. (2013a) |
| *Eurotiomycetes* | *Eurotiales* | *Aspergillaceae* | *Penicillium decaturense* | *Penicillium decaturense* | Leaf litter | Mexico | 1 | Savković et al. (2025) |
| *Eurotiomycetes* | *Eurotiales* | *Aspergillaceae* | *Penicillium decumbens* | *Penicillium decumbens* | Soil-sediment, air, bat guano, other, wood, unreported | Brazil, Czech Republic, Italy, Kazakhstan, Poland, Russia, Slovakia | 7 | Vanderwolf et al. (2013a); Taylor et al. (2013, 2014) |
| *Eurotiomycetes* | *Eurotiales* | *Aspergillaceae* | *Penicillium dierckxii* | *Penicillium dierckxii* | Air, other, soil-sediment, bat guano, rock surface, | Canada, China, Czech Republic, India, Kazakhstan, Poland | 6 | Vanderwolf et al. (2013a); Man et al. (2015); Zhang et al. (2017) |
| *Eurotiomycetes* | *Eurotiales* | *Aspergillaceae* | *Penicillium dierckxii* | *Penicillium fellutanum* | Unknown | China | 1 | Man (2017) |
| *Eurotiomycetes* | *Eurotiales* | *Aspergillaceae* | *Penicillium digitatum* | *Penicillium digitatum* | Air, soil-sediment, unreported | China, Czech Republic, Italy, Kazakhstan, Spain | 5 | Vanderwolf et al. (2013a); Zhang et al. (2021) |
| *Eurotiomycetes* | *Eurotiales* | *Aspergillaceae* | *Penicillium dipodomyicola* | *Penicillium dipodomyicola* | Unreported | China | 1 | Zhang (2019) |
| *Eurotiomycetes* | *Eurotiales* | *Aspergillaceae* | *Penicillium dipodomyus* | *Penicillium dipodomyis* | Unreported | China | 1 | Zhang et al. (2021) |
| *Eurotiomycetes* | *Eurotiales* | *Aspergillaceae* | *Penicillium echinulatum* | *Penicillium echinulatum* | Bat guano, cave wall, soil, wood | Canada, China, Czech Republic, Slovakia, USA | 5 | Vanderwolf et al. (2013a); Out et al. (2016); Held et al. (2020) |
| *Eurotiomycetes* | *Eurotiales* | *Aspergillaceae* | *Penicillium expansum* | *Penicillium expansum* | Air, bat guano, wood, water, cave wall, soil-sediment, animal bone, asymptomatic specimens of Proteus anguinus | Brazil, China, Czech Republic, France, Germany, Iran, Italy, Poland, Romania, Slovakia, Slovenia, Spain, Ukraine, USA | 14 | Taylor et al. (2013); Vanderwolf et al. (2013a); Zhang et al. (2021) |
| *Eurotiomycetes* | *Eurotiales* | *Aspergillaceae* | *Penicillium flavigenum* | *Penicillium flavigenum* | Air, cave sediment, bamboo, bat guano, branch, soil | Brazil, China, Romania, Spain | 4 | Nováková et al. (2018); Tavares et al. (2018); Zhang et al. (2021) |
| *Eurotiomycetes* | *Eurotiales* | *Aspergillaceae* | *Penicillium freii* | *Penicillium freii* | Air, sediment | China, Spain | 2 | Man (2017) |
| *Eurotiomycetes* | *Eurotiales* | *Aspergillaceae* | *Penicillium glabrum* | *Penicillium frequentans* | Air, soil-sediment, other, cave wall, water, wood | Czech Republic, Kazakhstan, Korea, Poland, Romania, Russia, USA | 7 | Vanderwolf et al. (2013a); Man (2017) |
| *Eurotiomycetes* | *Eurotiales* | *Aspergillaceae* | *Penicillium glabrum* | *Penicillium glabrum* | Air, other, soil-sediment, bat guano, cave wall | Australia, Brazil, China, Czech Republic, Israel, Poland, Slovakia, South Africa, Spain | 9 | Vanderwolf et al. (2013a); Taylor et al. (2013, 2014); Zhang et al. (2021) |
| *Eurotiomycetes* | *Eurotiales* | *Aspergillaceae* | *Penicillium gladioli* | *Penicillium gladioli* | Unreported | China | 1 | Vanderwolf et al. (2013a) |
| *Eurotiomycetes* | *Eurotiales* | *Aspergillaceae* | *Penicillium glandicola* | *Penicillium glandicola* | Air, soil-sediment, unreported, bat guano, earthworm casts, rock surface, soil | China, Czech Republic, Italy, Korea, Poland, Slovakia | 6 | Vanderwolf et al. (2013a); Zhang et al. (2021) |
| *Eurotiomycetes* | *Eurotiales* | *Aspergillaceae* | *Penicillium glaucoalbidum* | *Penicillium glaucoalbidum* | Sediment, underwater sediment of Airbell II | Poland, Romania | 2 | Vanderwolf et al. (2013, 2015, 2019); Zhang et al. (2021) |
| *Eurotiomycetes* | *Eurotiales* | *Aspergillaceae* | *Penicillium granulatum* | *Penicillium granulatum* | air, rock | China, Slovakia | 2 | Ogórek et al. (2016b, c) |
| *Eurotiomycetes* | *Eurotiales* | *Aspergillaceae* | *Penicillium griseofulvum* | *Penicillium griseofulvum* | Bat, dead isopod bodies, cave wall, soil-sediment, wood | Brazil, China, Czech Republic, Italy, Poland, Romania, USA | 7 | Vanderwolf et al. (2013, 2019); Taylor et al. (2013, 2014); Zhang et al. (2021) |
| *Eurotiomycetes* | *Eurotiales* | *Aspergillaceae* | *Penicillium griseolum* | *Penicillium griseolum* | Soil-sediment | Australia | 1 | Vanderwolf et al. (2013a) |
| *Eurotiomycetes* | *Eurotiales* | *Aspergillaceae* | *Penicillium guaibinense* | *Penicillium guaibinense* | Bat | Brazil | 1 | Cunha et al. (2020) |
| *Eurotiomycetes* | *Eurotiales* | *Aspergillaceae* | *Penicillium guanacastense* | *Penicillium guanacastense* | Air | Brazil, China | 2 | Zhang et al. (2021) |
| *Eurotiomycetes* | *Eurotiales* | *Aspergillaceae* | *Penicillium halotolerans* | *Penicillium halotolerans* | Unreported | China | 1 | Zhang et al. (2021) |
| *Eurotiomycetes* | *Eurotiales* | *Aspergillaceae* | *Penicillium herquei* | *Penicillium herquei* | cave wall, soil-sediment, leaf litter | China, Mexico, Russia | 3 | Vanderwolf et al. (2013a); Zhang et al. (2017) |
| *Eurotiomycetes* | *Eurotiales* | *Aspergillaceae* | *Penicillium hirsutum* | *Penicillium hirsutum* | bat guano, soil-sediment | Czech Republic, Russia, Slovakia | 3 | Vanderwolf et al. (2013a) |
| *Eurotiomycetes* | *Eurotiales* | *Aspergillaceae* | *Penicillium hispanicum* | *Penicillium hispanicum* | Air | Spain | 1 | Savković et al. (2025) |
| *Eurotiomycetes* | *Eurotiales* | *Aspergillaceae* | *Penicillium hordei* | *Penicillium hordei* | Bat guano | Slovakia | 1 | Vanderwolf et al. (2013a) |
| *Eurotiomycetes* | *Eurotiales* | *Aspergillaceae* | *Penicillium ibericum* | *Penicillium ibericum* | Soil | Belgium | 1 | Savković et al. (2025) |
| *Eurotiomycetes* | *Eurotiales* | *Aspergillaceae* | *Penicillium implicatum* | *Penicillium implicatum* | Air, soil-sediment, water, other | Bahamas, China, Czech Republic, Italy, Poland, Russia | 6 | Vanderwolf et al. (2013a) |
| *Eurotiomycetes* | *Eurotiales* | *Aspergillaceae* | *Penicillium italicum* | *Penicillium italicum* | Other, unreported, soil-sediment | Italy, Poland, UK | 3 | Vanderwolf et al. (2013a) |
| *Eurotiomycetes* | *Eurotiales* | *Aspergillaceae* | *Penicillium Jacksonii* | *Penicillium Jacksonii* | Unreported | China | 1 | Zhang et al. (2021) |
| *Eurotiomycetes* | *Eurotiales* | *Aspergillaceae* | *Penicillium janczewskii* | *Penicillium janczewskii* | Air, cave wall, soil-sediment, earthworm casts, other, wood, unreported | Belgium, Czech Republic, Italy, Kazakhstan, Romania, Russia, Slovakia | 7 | Vanderwolf et al. (2013a) |
| *Eurotiomycetes* | *Eurotiales* | *Aspergillaceae* | *Penicillium javanicum* | *Eupenicillium javanicum* | Soil-sediment, earthworm casts | Italy, Slovakia | 2 | Vanderwolf et al. (2013a) |
| *Eurotiomycetes* | *Eurotiales* | *Aspergillaceae* | *Penicillium javanicum* | *Penicillium javanicum* | bat guano | China | 1 | Zhang et al. (2021) |
| *Eurotiomycetes* | *Eurotiales* | *Aspergillaceae* | *Penicillium jensenii* | *Penicillium jensenii* | Cave wall, wood, soil-sediment | Czech Republic, Russia | 2 | Vanderwolf et al. (2013a) |
| *Eurotiomycetes* | *Eurotiales* | *Aspergillaceae* | *Penicillium lagerheimii* | *Penicillium lagerheimii* | Unreported | Kazakhstan | 1 | Vanderwolf et al. (2013a) |
| *Eurotiomycetes* | *Eurotiales* | *Aspergillaceae* | *Penicillium lanosum* | *Penicillium lanosum* | Soil-sediment, cave wall, water, air, unreported | Bahamas, Czech Republic, Ireland, Israel, Kazakhstan, Mexico, Poland | 7 | Vanderwolf et al. (2013a) |
| *Eurotiomycetes* | *Eurotiales* | *Aspergillaceae* | *Penicillium lividum* | *Penicillium lividum* | Unreported | China | 1 | Zhang et al. (2021) |
| *Eurotiomycetes* | *Eurotiales* | *Aspergillaceae* | *Penicillium ludwigii* | *Penicillium ludwigii* | Unreported | China | 1 | Zhang et al. (2021) |
| *Eurotiomycetes* | *Eurotiales* | *Aspergillaceae* | *Penicillium madriti* | *Penicillium madriti* | Unreported | China | 1 | Zhang et al. (2021) |
| *Eurotiomycetes* | *Eurotiales* | *Aspergillaceae* | *Penicillium magnielliptisporum* | *Penicillium magnielliptisporum* | Unreported | China | 1 | Zhang et al. (2021) |
| *Eurotiomycetes* | *Eurotiales* | *Aspergillaceae* | *Penicillium malachiteum* | *Penicillium malachiteum* | Unreported | Bulgaria, China | 2 | Mitova et al. (2017); Zhang et al. (2017) |
| *Eurotiomycetes* | *Eurotiales* | *Aspergillaceae* | *Penicillium mallochii* | *Penicillium mallochii* | Unreported | China | 1 | Zhang et al. (2021) |
| *Eurotiomycetes* | *Eurotiales* | *Aspergillaceae* | *Penicillium melanoconidium* | *Penicillium melanoconidium* | Bat guano | Slovakia | 1 | Vanderwolf et al. (2013a) |
| *Eurotiomycetes* | *Eurotiales* | *Aspergillaceae* | *Penicillium meleagrinum* | *Penicillium meleagrinum* | Bat guano, cigarette butt | China | 1 | Man (2017) |
| *Eurotiomycetes* | *Eurotiales* | *Aspergillaceae* | *Penicillium meleagrinum* var. *viridiflavum* | *Penicillium meleagrinum* var. *viridiflavum* | Cave wall | Sri Lanka | 1 | Savković et al. (2025) |
| *Eurotiomycetes* | *Eurotiales* | *Aspergillaceae* | *Penicillium melinii* | *Penicillium melinii* | Air, earthworm casts, unreported | Kazakhstan, Slovakia | 2 | Vanderwolf et al. (2013a) |
| *Eurotiomycetes* | *Eurotiales* | *Aspergillaceae* | *Penicillium mexicanum* | *Penicillium mexicanum* | Bat guano | China | 1 | Zhang et al. (2021) |
| *Eurotiomycetes* | *Eurotiales* | *Aspergillaceae* | *Penicillium miczynskii* | *Penicillium miczynskii* | Air, cave wall, wood, unreported | Canada, Czech Republic, Kazakhstan, Spain, USA | 5 | Vanderwolf et al. (2013a); Zhang et al. (2014) |
| *Eurotiomycetes* | *Eurotiales* | *Aspergillaceae* | *Penicillium montanense* | *Penicillium montanense* | Wood | USA | 1 | Held et al. (2020) |
| *Eurotiomycetes* | *Eurotiales* | *Aspergillaceae* | *Penicillium nalgiovense* | *Penicillium nalgiovense* | Air, soil-sediment | China, Russia, South Africa | 3 | Vanderwolf et al. (2013a); Zhang et al. (2021) |
| *Eurotiomycetes* | *Eurotiales* | *Aspergillaceae* | *Penicillium notatum* | *Penicillium notatum* | Sediment | China | 1 | Parkinson et al. (1989); Zhang et al. (2017) |
| *Eurotiomycetes* | *Eurotiales* | *Aspergillaceae* | *Penicillium ochrochloron* | *Penicillium ochrochloron* | Bat guano | China, France | 2 | Zhang et al. (2021) |
| *Eurotiomycetes* | *Eurotiales* | *Aspergillaceae* | *Penicillium olsonii* | *Penicillium olsonii* | Air, cave wall | China, spain | 1 | Vanderwolf et al. (2013a); Ma et al. (2025) |
| *Eurotiomycetes* | *Eurotiales* | *Aspergillaceae* | *Penicillium oxalicum* | *Penicillium oxalicum* | Soil-sediment, unreported, cave wall, bat guano | Algeria, Bahamas, Brazil, China, France, Israel, Italy, Kazakhstan, Korea, Poland, Russia, Serbia, Spain | 14 | Vanderwolf et al. (2013a); Taylor et al. (2013, 2014); Zhang et al. (2021) |
| *Eurotiomycetes* | *Eurotiales* | *Aspergillaceae* | *Penicillium palitans* | *Penicillium palitans* | Soil-sediment | Italy, Russia | 2 | Vanderwolf et al. (2013a) |
| *Eurotiomycetes* | *Eurotiales* | *Aspergillaceae* | *Penicillium pancosmium* | *Penicillium pancosmium* | Rock, paintings | Bulgaria, China | 2 | Man (2017); Mitova et al. (2017); Zhang et al. (2017) |
| *Eurotiomycetes* | *Eurotiales* | *Aspergillaceae* | *Penicillium panissanguineum* | *Penicillium panissanguineum* | Cave wall | Unknow |  | Savković et al. (2025) |
| *Eurotiomycetes* | *Eurotiales* | *Aspergillaceae* | *Penicillium parvulum* | *Penicillium parvulum* | Unreported | China | 1 | Zhang et al. (2017) |
| *Eurotiomycetes* | *Eurotiales* | *Aspergillaceae* | *Penicillium paxilli* | *Penicillium paxilli* | Air, bat guano, soil-sediment, earthworm casts, water | China, Italy, Malaysia, Russia, Slovakia | 5 | Vanderwolf et al. (2013a); Jiang et al. (2017); Wasti et al. (2021) |
| *Eurotiomycetes* | *Eurotiales* | *Aspergillaceae* | *Penicillium phoeniceum* | *Penicillium phoeniceum* | Cave sediment | Romania | 1 | Nováková et al. (2018) |
| *Eurotiomycetes* | *Eurotiales* | *Aspergillaceae* | *Penicillium pimiteouiense* | *Penicillium pimiteouiense* | Unreported | China | 1 | Zhang et al. (2021) |
| *Eurotiomycetes* | *Eurotiales* | *Aspergillaceae* | *Penicillium polonicum* | *Penicillium polonicum* | Air, cave wall, cave water, rock | China, Poland, Romania, South Africa, USA | 5 | Vanderwolf et al. (2013a); Zhang et al. (2021) |
| *Eurotiomycetes* | *Eurotiales* | *Aspergillaceae* | *Penicillium purpurascens* | *Penicillium purpurascens* | Unreported, cave wall, soil-sediment, earthworm casts | Poland, Romania, Slovakia | 3 | Vanderwolf et al. (2013a); |
| *Eurotiomycetes* | *Eurotiales* | *Aspergillaceae* | *Penicillium raistrickii* | *Penicillium raistrickii* | Air | Czech Republic, Spain | 2 | Vanderwolf et al. (2013a) |
| *Eurotiomycetes* | *Eurotiales* | *Aspergillaceae* | *Penicillium raperi* | *Penicillium raperi* | Unreported | China | 1 | Zhang et al. (2021) |
| *Eurotiomycetes* | *Eurotiales* | *Aspergillaceae* | *Penicillium raphiae* | *Penicillium raphiae* | Rock surface, wood | China, USA | 2 | Held et al. (2020); Zhang et al. (2021) |
| *Eurotiomycetes* | *Eurotiales* | *Aspergillaceae* | *Penicillium restrictum* | *Penicillium restrictum* | Air, unreported, soil-sediment | Australia, Brazil, China, India, Kazakhstan, Slovakia | 6 | Vanderwolf et al. (2013a); Taylor et al. (2014) |
| *Eurotiomycetes* | *Eurotiales* | *Aspergillaceae* | *Penicillium robsamsonii* | *Penicillium robsamsonii* | Unreported | China | 1 | Zhang et al. (2021) |
| *Eurotiomycetes* | *Eurotiales* | *Aspergillaceae* | *Penicillium roqueforti* | *Penicillium roqueforti* | Bat guano, soil-sediment, other | Czech Republic, Kazakhstan, Puerto Rico, Slovakia, Spain, USA | 6 | Vanderwolf et al. (2013a) |
| *Eurotiomycetes* | *Eurotiales* | *Aspergillaceae* | *Penicillium roseopurpureum* | *Penicillium roseopurpureum* | Air, soil-sediment | China, Czech Republic, Mexico, Thailand | 4 | Vanderwolf et al. (2013a); Ogórek et al. (2016b, d) |
| *Eurotiomycetes* | *Eurotiales* | *Aspergillaceae* | *Penicillium rubens* | *Penicillium rubens* | Air, bat guano, rock sufaces, soil | Canada, China, Spain, USA | 4 | Visagie et al. (2019) |
| *Eurotiomycetes* | *Eurotiales* | *Aspergillaceae* | *Penicillium sacculum* | *Penicillium sacculum* | Soil-sediment, earthworm casts | Slovakia | 1 | Vanderwolf et al. (2013a) |
| *Eurotiomycetes* | *Eurotiales* | *Aspergillaceae* | *Penicillium sanguifluum* | *Penicillium sanguifluum* | Unreported | China | 1 | Zhang et al. (2021) |
| *Eurotiomycetes* | *Eurotiales* | *Aspergillaceae* | *Penicillium scabrosum* | *Penicillium scabrosum* | Soil-sediment | China, Slovakia | 2 | Vanderwolf et al. (2013a); Zhang et al. (2021) |
| *Eurotiomycetes* | *Eurotiales* | *Aspergillaceae* | *Penicillium sclerotiorum* | *Penicillium sclerotiorum* | Water | China | 1 | Zhang et al. (2021) |
| *Eurotiomycetes* | *Eurotiales* | *Aspergillaceae* | *Penicillium simplicissimum* | *Penicillium cremeogriseum* | Air,soil-sediment, bat guano, wood, cave wall | Brazil, Bulgaria, Canada, China, Czech Republic, France, India, Italy, Korea, Poland, Romania, Russia, Slovakia, Sri Lanka | 14 | Vanderwolf et al. (2013a); Zhang et al. (2021) |
| *Eurotiomycetes* | *Eurotiales* | *Aspergillaceae* | *Penicillium simplicissimum* | *Penicillium janthinellum* | Dead isopod bodies (*Trachelipus troglobius*) | China, Romania | 1 | Man (2017); Ogórek et al. (2018) |
| *Eurotiomycetes* | *Eurotiales* | *Aspergillaceae* | *Penicillium simplicissimum* | *Penicillium simplicissimum* | Bamboo, soil-sediment, air, other, cave wall, wood | Brazil, Canada, , Czech Republic, France, India, Italy, Korea, Malaysia, Poland, Russia, Slovakia | 12 | Vanderwolf et al. (2013a); Taylor et al. (2013, 2014); Wasti et al. (2021) |
| *Eurotiomycetes* | *Eurotiales* | *Aspergillaceae* | *Penicillium sizovae* | *Penicillium sizovae* | Unreported | China | 1 | Zhang et al. (2021) |
| *Eurotiomycetes* | *Eurotiales* | *Aspergillaceae* | *Penicillium solitum* | *Penicillium crustosum* | Cave wall, soil-sediment, air, other | China, USA, Poland, Czech Republic, Russia | 5 | Vanderwolf et al. (2013a); Man (2017) |
| *Eurotiomycetes* | *Eurotiales* | *Aspergillaceae* | *Penicillium solitum* | *Penicillium solitum* | Bat guano, , soil-sediment, other | Bulgaria, China, Canada, Romania, South Africa, Bahamas, Italy, Slovakia | 8 | Vanderwolf et al. (2013a); Taylor et al. (2013); Zhang et al. (2021) |
| *Eurotiomycetes* | *Eurotiales* | *Aspergillaceae* | *Penicillium* sp. | *Thysanophora* sp. | Unreported | Canada | 1 | Vanderwolf et al. (2013a) |
| *Eurotiomycetes* | *Eurotiales* | *Aspergillaceae* | Penicillium sp. 1 section Lanata-Divaricata | Penicillium sp. 1 section Lanata-Divaricata | Air | Brazil | 1 | Cunha et al. (2020) |
| *Eurotiomycetes* | *Eurotiales* | *Aspergillaceae* | Penicillium sp. 2 section Lanata-Divaricata | Penicillium sp. 2 section Lanata-Divaricata | Bat guano | Brazil | 1 | Cunha et al. (2020) |
| *Eurotiomycetes* | *Eurotiales* | *Aspergillaceae* | Penicillium sp. 3 section Brevicompacta | Penicillium sp. 3 section Brevicompacta | Bat guano | Brazil | 1 | Cunha et al. (2020) |
| *Eurotiomycetes* | *Eurotiales* | *Aspergillaceae* | *Penicillium* sp. 4 section *Fasciculata* | *Penicillium* sp. 4 section *Fasciculata* | Sediment | Brazil | 1 | Alves et al. (2022) |
| *Eurotiomycetes* | *Eurotiales* | *Aspergillaceae* | *Penicillium* sp. 5 section *Paradoxa* | *Penicillium* sp. 5 section *Paradoxa* | Sediment | Brazil | 1 | Alves et al. (2022) |
| *Eurotiomycetes* | *Eurotiales* | *Aspergillaceae* | *Penicillium spathulatum* | *Penicillium spathulatum* | Bat cave, bat guano | Canada, Mexico | 2 | Visagie et al. (2019) |
| *Eurotiomycetes* | *Eurotiales* | *Aspergillaceae* | *Penicillium speluncae* | *Penicillium speluncae* | bat cave, cave wall | Canada | 1 | Visagie et al. (2019) |
| *Eurotiomycetes* | *Eurotiales* | *Aspergillaceae* | *Penicillium Spainulosum* | *Penicillium Spainulosum* | bat guano, other, cave wall, soil-sediment, unreported, wood | China, Czech Republic, France, Poland, Romania, Russia, Kazakhstan, USA | 8 | Vanderwolf et al. (2013a); Held et al. (2020); Zhang et al. (2021) |
| *Eurotiomycetes* | *Eurotiales* | *Aspergillaceae* | *Penicillium guanacastense* | *Penicillium guanacastense* | Air | Brazil | 1 | Alves et al. (2022) |
| *Eurotiomycetes* | *Eurotiales* | *Aspergillaceae* | Penicillium spp. | Penicillium spp. | Air, precipitate, live bat fur, insect, soil-sediment, water, cave wall, bat guano, bat guano, Thereuopoda sp., Trigonilus corallinus | Australia, Bahamas, Belgium, Brazil, Bulgaria, Canada, China, Czech Republic, Egypt, France, Germany, Greece, India, Italy, Japan, Kazakhstan, Korea, Malaysia, Mexico, North America, Poland, Puerto Rico, Romania, Russia, Serbia, Slovakia, Slovenia, South Africa, Spain, Switzerland, Thailand, UK, Ukraine, USA, Venezuela | 33 | Wang et al. (2010); Vanderwolf et al. (2013, 2015, 2019); Taylor et al. (2014); Kokurewicz et al. (2016); Jiang et al. (2017); Man (2017); Burow et al. (2019); Wasti et al. (2021); Zhang et al. (2021); Elkhateeb et al. (2022); Suetrong et al. (2023); Ma et al. (2025) |
| *Eurotiomycetes* | *Eurotiales* | *Aspergillaceae* | *Penicillium sumatraense* | *Penicillium sumatraense* | Air, rock surface, soil | China, Spain | 2 | Zhang et al. (2021); Ma et al. (2025) |
| *Eurotiomycetes* | *Eurotiales* | *Aspergillaceae* | *Penicillium swiecickii* | *Penicillium swiecickii* | Air, bat | Poland, USA | 2 | Zhang et al. (2014); Pusz et al. (2017) |
| *Eurotiomycetes* | *Eurotiales* | *Aspergillaceae* | *Penicillium tardochrysogenum* | *Penicillium tardochrysogenum* | Rock | China | 1 | Zhang et al. (2021) |
| *Eurotiomycetes* | *Eurotiales* | *Aspergillaceae* | *Penicillium thomii* | *Penicillium thomii* | Bat guano, , earthworm casts, sediment | Canada, China, Slovakia, Spain, Poland | 3 | Vanderwolf et al. (2013a); Taylor et al. (2013, 2014); Zhang et al. (2021) |
| *Eurotiomycetes* | *Eurotiales* | *Aspergillaceae* | *Penicillium toxicarium* | *Penicillium toxicarium* | sediment | China | 1 | Man (2017) |
| *Eurotiomycetes* | *Eurotiales* | *Aspergillaceae* | *Penicillium ubiquetum* | *Penicillium ubiquetum* | Unreported, wood | China, USA | 2 | Held et al. (2020); Zhang et al. (2021) |
| *Eurotiomycetes* | *Eurotiales* | *Aspergillaceae* | *Penicillium vancouverens* | *Penicillium vancouverens* | Air | Spain | 1 | Savković et al. (2025) |
| *Eurotiomycetes* | *Eurotiales* | *Aspergillaceae* | *Penicillium vanluykii* | *Penicillium vanluykii* | Unreported | USA | 1 | Zhang et al. 2014 |
| *Eurotiomycetes* | *Eurotiales* | *Aspergillaceae* | *Penicillium vinaceum* | *Penicillium vinaceum* | Air, rock surface, bat guano | China Mexico | 2 | Savković et al. (2025) |
| *Eurotiomycetes* | *Eurotiales* | *Aspergillaceae* | *Penicillium virgatum* | *Penicillium virgatum* | Unreported | China | 1 | Zhang et al. (2021) |
| *Eurotiomycetes* | *Eurotiales* | *Aspergillaceae* | *Penicillium viticola* | *Penicillium viticola* | Unreported | China | 1 | Zhang et al. (2021) |
| *Eurotiomycetes* | *Eurotiales* | *Aspergillaceae* | *Penicillium vulpinum* | *Penicillium vulpinum* | Air, bat guano, cave wall | Belgium, Canada, Czech Republic, Slovakia, USA | 5 | Vanderwolf et al. (2013a); Zhang et al. (2014); |
| *Eurotiomycetes* | *Eurotiales* | *Aspergillaceae* | *Penicillium waksmanii* | *Penicillium waksmanii* | Earthworm casts, wood, other, air, cave wall | Poland, Czech Republic, Slovakia, Romania | 4 | Vanderwolf et al. (2013a); Pusz et al. (2014) |
| *Eurotiomycetes* | *Eurotiales* | *Aspergillaceae* | *Penicillium westlingii* | *Penicillium westlingii* | Bat cave | Canada | 1 | Visagie et al. (2019) |
| *Eurotiomycetes* | *Eurotiales* | *Aspergillaceae* | *Sagenomella* sp. | *Sagenomella* sp. | Unreported | Canada | 1 | Vanderwolf et al. (2013a) |
| *Eurotiomycetes* | *Eurotiales* | *Aspergillaceae* | *Talaromyces aculeatus* | *Penicillium aculeatum* | Cave wall | Russia | 1 | Vanderwolf et al. (2013a) |
| *Eurotiomycetes* | *Eurotiales* | *Aspergillaceae* | *Talaromyces aculeatus* | *Talaromyces aculeatus* | Unreported | China | 1 | Zhang et al. (2021) |
| *Eurotiomycetes* | *Eurotiales* | *Aspergillaceae* | *Talaromyces allahabadensis* | *Talaromyces allahabadensis* | Air, bat guano | Brazil | 1 | Cunha et al. (2020) |
| *Eurotiomycetes* | *Eurotiales* | *Aspergillaceae* | *Talaromyces brunneus* | *Talaromyces brunneus* | Air | Brazil, Spain | 2 | Paula et al. (2019) |
| *Eurotiomycetes* | *Eurotiales* | *Aspergillaceae* | *Talaromyces cavernicola* | *Talaromyces cavernicola* | Air | Brazil | 1 | Alves et al. (2022); Savković et al. (2025) |
| *Eurotiomycetes* | *Eurotiales* | *Aspergillaceae* | *Talaromyces cellulolyticus* | *Talaromyces cellulolyticus* | Cave water | China, Poland | 2 | Zhang (2019) |
| *Eurotiomycetes* | *Eurotiales* | *Aspergillaceae* | *Talaromyces diversus* | *Penicillium diversum* | Other | Czech Republic | 1 | Vanderwolf et al. (2013a) |
| *Eurotiomycetes* | *Eurotiales* | *Aspergillaceae* | *Talaromyces diversus* | *Talaromyces diversus* | Unreported | Czech Republic | 1 | Vanderwolf et al. (2013a) |
| *Eurotiomycetes* | *Eurotiales* | *Aspergillaceae* | *Talaromyces duclauxii* | *Penicillium duclauxii* | Soil-sediment, insects | Bahamas, Slovenia | 2 | Vanderwolf et al. (2013a) |
| *Eurotiomycetes* | *Eurotiales* | *Aspergillaceae* | *Talaromyces duclauxii* | *Talaromyces duclauxii* | Soil-sediment | Bahamas, Slovenia | 2 | Vanderwolf et al. (2013a) |
| *Eurotiomycetes* | *Eurotiales* | *Aspergillaceae* | *Talaromyces flavus* | *Penicillium vermiculatum* | Soil-sediment | Poland | 1 | Vanderwolf et al. (2013a) |
| *Eurotiomycetes* | *Eurotiales* | *Aspergillaceae* | *Talaromyces flavus* | *Talaromyces flavus* | Bat guano, earthworm casts, soil-sediment, insect, water, Bamboo, rock surface | China, Poland, Slovakia | 3 | Vanderwolf et al. (2013a); Zhang et al. (2021) |
| *Eurotiomycetes* | *Eurotiales* | *Aspergillaceae* | *Talaromyces flavus* var. *flavus* | *Talaromyces flavus* var. *flavus* | Wood | Czech Republic | 1 | Vanderwolf et al. (2013a) |
| *Eurotiomycetes* | *Eurotiales* | *Aspergillaceae* | *Talaromyces funiculosus* | *Penicillium funiculosum* | Air, other, soil-sediment | China, Poland, Russia | 3 | Vanderwolf et al. (2013a); Zhang et al. (2021) |
| *Eurotiomycetes* | *Eurotiales* | *Aspergillaceae* | *Talaromyces funiculosus* | *Talaromyces funiculosus* | Unreported | Unknow |  | Zhang et al. (2021) |
| *Eurotiomycetes* | *Eurotiales* | *Aspergillaceae* | *Talaromyces islandicus* | *Penicillium islandicum* | Bat guano | Brazil | 1 | Taylor et al. (2013) |
| *Eurotiomycetes* | *Eurotiales* | *Aspergillaceae* | *Talaromyces islandicus* | *Talaromyces islandicus* | Unreported | Brazil | 1 | Taylor et al. (2013) |
| *Eurotiomycetes* | *Eurotiales* | *Aspergillaceae* | *Talaromyces kabodanensis* | *Talaromyces kabodanensis* | Symptomatic specimens of *Proteus anguinus* | Slovenia | 1 | Savković et al. (2025) |
| *Eurotiomycetes* | *Eurotiales* | *Aspergillaceae* | *Talaromyces kendrickii* | *Talaromyces kendrickii* | Underwater sediment of Airbell II | Romania | 1 | Nováková et al. (2018) |
| *Eurotiomycetes* | *Eurotiales* | *Aspergillaceae* | *Talaromyces loliensis* | *Penicillium loliense* | soil-sediment | Thailand | 1 | Vanderwolf et al. (2013a) |
| *Eurotiomycetes* | *Eurotiales* | *Aspergillaceae* | *Talaromyces loliensis* | *Talaromyces loliensis* | Unreported | Unknow |  | Zhang et al. (2021) |
| *Eurotiomycetes* | *Eurotiales* | *Aspergillaceae* | *Talaromyces miniluteus* | *Talaromyces miniluteus* | Air, leaf litter | Spain, Mexico | 1 | Wasti et al. (2021) |
| *Eurotiomycetes* | *Eurotiales* | *Aspergillaceae* | *Talaromyces minioluteus* | *Penicillium minioluteum* | Air, bat guano, earthworm casts, insects, soil-sediment | Czech Republic, Slovakia, Russia | 3 | Vanderwolf et al. (2013a) |
| *Eurotiomycetes* | *Eurotiales* | *Aspergillaceae* | *Talaromyces phuphaphetensis* | *Talaromyces phuphaphetensis* | Soil | Thailand | 1 | Nuankaew et al. (2022) |
| *Eurotiomycetes* | *Eurotiales* | *Aspergillaceae* | *Talaromyces piceae* | *Penicillium piceum* | Bat guano, other | France, Czech Republic | 2 | Vanderwolf et al. (2013a) |
| *Eurotiomycetes* | *Eurotiales* | *Aspergillaceae* | *Talaromyces piceus* | *Talaromyces piceae* | Bat guano | France | 1 | Vanderwolf et al. (2013a) |
| *Eurotiomycetes* | *Eurotiales* | *Aspergillaceae* | *Talaromyces pinophilus* | *Penicillium pinophilum* | Air, cave wall, bat guano, earthworm casts | Brazil, Slovakia, USA | 3 | Vanderwolf et al. (2013a); Taylor et al. (2013) |
| *Eurotiomycetes* | *Eurotiales* | *Aspergillaceae* | *Talaromyces pinophilus* | *Talaromyces pinophilus* | Soil | Brazil, China | 2 | Taylor et al. (2013); Vanderwolf et al. (2013a); Zhang et al. (2017) |
| *Eurotiomycetes* | *Eurotiales* | *Aspergillaceae* | *Talaromyces purpureogenus* | *Penicillium purpurogenum* | Air, soil-sediment, bat guano | Brazil, Canada, Czech Republic, France, India, Italy, Korea, Malaysia, Poland, Russia, Slovakia | 11 | Vanderwolf et al. (2013a); Taylor et al. (2013, 2014); Nováková et al. (2018) |
| *Eurotiomycetes* | *Eurotiales* | *Aspergillaceae* | *Talaromyces purpureogenus* | *Talaromyces purpureogenus* | Unreported | China | 1 | Popkova and Mazina (2019) |
| *Eurotiomycetes* | *Eurotiales* | *Aspergillaceae* | *Talaromyces radicus* | *Talaromyces radicus* | Air | China | 1 | Jiang et al. (2017) |
| *Eurotiomycetes* | *Eurotiales* | *Aspergillaceae* | *Talaromyces ruber* | *Penicillium rubrum* | Soil-sediment | China, Italy | 2 | Vanderwolf et al. (2013a); Jiang et al. (2017a, b) |
| *Eurotiomycetes* | *Eurotiales* | *Aspergillaceae* | *Talaromyces ruber* | *Talaromyces ruber* | Bat bones, microbial mat | China, Romania | 2 | Vanderwolf et al. (2013a); Nováková et al. (2018) |
| *Eurotiomycetes* | *Eurotiales* | *Aspergillaceae* | *Talaromyces rugulosus* | *Penicillium rugulosum* | Soil-sediment, earthworm casts, insects, cave wall | Czech Republic, Israel, Italy, Poland, Slovakia, South Africa | 6 | Vanderwolf et al. (2013a) |
| *Eurotiomycetes* | *Eurotiales* | *Aspergillaceae* | *Talaromyces rugulosus* | *Penicillium tardum* | Air, soil-sediment, wood | Czech Republic, Italy, Kazakhstan | 3 | Vanderwolf et al. (2013a) |
| *Eurotiomycetes* | *Eurotiales* | *Aspergillaceae* | *Talaromyces rugulosus* | *Talaromyces rugulosus* | Air, rock surface | China, Spain | 3 | Vanderwolf et al. (2013a); Zhang et al. (2021) |
| *Eurotiomycetes* | *Eurotiales* | *Aspergillaceae* | *Talaromyces satunensis* | *Talaromyces satunensis* | Soil | Thailand | 1 | Nuankaew et al. (2022) |
| *Eurotiomycetes* | *Eurotiales* | *Aspergillaceae* | *Talaromyces soli* | *Talaromyces soli* | Bat guano | France | 1 | Savković et al. (2025) |
| *Eurotiomycetes* | *Eurotiales* | *Aspergillaceae* | Talaromyces sp. 1 section Talaromyces | Talaromyces sp. 1 section Talaromyces | Bat | Brazil | 1 | Cunha et al. (2020) |
| *Eurotiomycetes* | *Eurotiales* | *Aspergillaceae* | Talaromyces sp. 2 section Talaromyces | Talaromyces sp. 2 section Talaromyces | Bat | Brazil | 1 | Cunha et al. (2020) |
| *Eurotiomycetes* | *Eurotiales* | *Aspergillaceae* | *Talaromyces* spp. | *Talaromyces* spp. | Air, organic litter, soil-, sediment, earthworm casts | China, Malaysia, Romania, Serbia, Slovakia, Thailand | 6 | Vanderwolf et al. (2013a); Wasti et al. (2021); Zhang et al. (2021); Suetrong et al. (2023) |
| *Eurotiomycetes* | *Eurotiales* | *Aspergillaceae* | *Talaromyces sublevisporus* | *Talaromyces sublevisporus* | Unreported | China | 1 | Zhang et al. (2021) |
| *Eurotiomycetes* | *Eurotiales* | *Aspergillaceae* | *Talaromyces variabilis* | *Penicillium variabile* | Air, bat guano, earthworm casts, soil-sediment, wood | Brazil, Czech Republic, Poland, Puerto Rico, Romania, Russia, Slovakia, South Africa | 8 | Taylor et al. (2013); Vanderwolf et al. (2013a) |
| *Eurotiomycetes* | *Eurotiales* | *Aspergillaceae* | *Talaromyces variabilis* | *Talaromyces variabilis* | Cave substrata | Brazil, China | 1 | Taylor et al. (2013); Vanderwolf et al. (2013) |
| *Eurotiomycetes* | *Eurotiales* | *Aspergillaceae* | *Talaromyces varians* | *Penicillium varians* | Soil-sediment, cave wall | Russia | 1 | Vanderwolf et al. (2013a) |
| *Eurotiomycetes* | *Eurotiales* | *Aspergillaceae* | *Talaromyces varians* | *Talaromyces varians* | Soil-sediment, cave wall | Russia | 1 | Vanderwolf et al. (2013a) |
| *Eurotiomycetes* | *Eurotiales* | *Aspergillaceae* | *Talaromyces verruculosus* | *Penicillium verruculosum* | Wood, soil-sediment | Czech Republic, Russia | 2 | Vanderwolf et al. (2013a) |
| *Eurotiomycetes* | *Eurotiales* | *Aspergillaceae* | *Talaromyces verruculosus* | *Talaromyces verruculosus* | Soil-sediment, wood | Czech Republic, Russia | 3 | Vanderwolf et al. (2013a) |
| *Eurotiomycetes* | *Eurotiales* | *Aspergillaceae* | *Talaromyces wortmannii* | *Talaromyces wortmannii* | Earthworm casts | Slovakia | 1 | Vanderwolf et al. (2013a) |
| *Eurotiomycetes* | *Eurotiales* | *Aspergillaceae* | Thermoascus sp. | Thermoascus sp. | Soil-sediment | Italy | 1 | Vanderwolf et al. (2013a) |
| *Eurotiomycetes* | *Eurotiales* | *Aspergillaceae* | *Thermomyces thermophilus* | *Talaromyces thermophilus* | Soil-sediment | China (Taiwan Province) | 1 | Vanderwolf et al. (2013a) |
| *Eurotiomycetes* | *Eurotiales* | *Aspergillaceae* | *Thysanophora penicillioides* | *Thysanophora penicilliodes* | Cave wall, air | Czech Republic, Ireland | 2 | Vanderwolf et al. (2013a) |
| *Eurotiomycetes* | *Eurotiales* | *Aspergillaceae* | *Torulomyces sp.* | *Torulomyces sp.* | Unreported | France | 1 | Leplat et al. (2018) |
| *Eurotiomycetes* | *Eurotiales* | *Incertae sedis* | *Thermomyces lanuginosus* | *Thermomyces lanuginosus* | Soil-sediment | India, China (Taiwan Province) | 2 | Vanderwolf et al. (2013a) |
| *Eurotiomycetes* | *Eurotiales* | *Monascaceae* | Basipetospora sp. | Basipetospora sp. | Air | Spain | 1 | Vanderwolf et al. (2013a) |
| *Eurotiomycetes* | *Eurotiales* | *Monascaceae* | *Monascus purpureus* | *Monascus purpureus* | Unreported | China | 1 | Man et al. (2015) |
| *Eurotiomycetes* | *Eurotiales* | *Monascaceae* | *Monascus ruber* | *Monascus ruber* | Bat guano, water | China, France | 2 | Vanderwolf et al. (2013a) |
| *Eurotiomycetes* | *Eurotiales* | *Trichocomaceae* | *Ascospirella lutea* | *Talaromyces luteus* | Other, soil-sediment, unreported | Kazakhstan, Poland, Russia, USA | 4 | Vanderwolf et al. (2013a); Pusz et al. (2014) |
| *Eurotiomycetes* | *Eurotiales* |  | Eurotiales sp. | Eurotiales sp. | Painting sediment | Bulgaria | 1 | Savković et al. (2025) |
| *Eurotiomycetes* | *Onygenales* | *Ajellomycetaceae* | *Ajellomyces capsulatus* | *Emmonsiella capsulata* | Wood | France | 1 | Vanderwolf et al. (2013a) |
| *Eurotiomycetes* | *Onygenales* | *Ajellomycetaceae* | *Paracoccidioides brasiliensis* | *Paracoccidioides brasiliensis* | Cave wall | France | 1 | Vanderwolf et al. (2013a) |
| *Eurotiomycetes* | *Onygenales* | *Apinisiaceae* | *Albidomyces albicans* | *Arachnotheca albicans* | Cave wall | USA | 1 | Vanderwolf et al. (2013a) |
| *Eurotiomycetes* | *Onygenales* | *Apinisiaceae* | *Apinisia keratinophila* | *Myriodontium keratinophilum* | Air, cave sediment, corroded cave walls, underwater sediment of Airbell II | China, Romania | 2 | Man et al. (2015); Man (2017); Zhang et al. (2017); Nováková et al. (2018) |
| *Eurotiomycetes* | *Onygenales* | *Apinisiaceae* | *Apinisia* spp. | *Myriodontium* spp. | Air, soil-sediment, Organic litter, | China, France, Thailand | 3 | Leplat et al. (2018); Zhang et al. (2021); Suetrong et al. (2023) |
| *Eurotiomycetes* | *Onygenales* | *Arthrodermataceae* | *Arthroderma chinense* | *Pectinotrichum chinense* | Unreported | China | 1 | Zhang et al. (2017) |
| *Eurotiomycetes* | *Onygenales* | *Arthrodermataceae* | *Arthroderma ciferrii* | *Arthroderma ciferrii* | Unreported | China | 1 | Zhang et al. (2021) |
| *Eurotiomycetes* | *Onygenales* | *Arthrodermataceae* | *Arthroderma cuniculi* | *Arthroderma cuniculi* | Soil | USA | 1 | Savković et al. (2025) |
| *Eurotiomycetes* | *Onygenales* | *Arthrodermataceae* | *Arthroderma curreyi* | *Arthroderma curreyi* | Bat guano | China, Hungary | 2 | Vanderwolf et al. (2013a); Zhang et al. (2017) |
| *Eurotiomycetes* | *Onygenales* | *Arthrodermataceae* | *Arthroderma melis* | *Arthroderma melis* | Cave wall | Ireland | 1 | Vanderwolf et al. (2013a) |
| *Eurotiomycetes* | *Onygenales* | *Arthrodermataceae* | *Arthroderma multifidum* | *Arthroderma multifidum* | Sediment, soil | Slovakia | 1 | Savković et al. (2025) |
| *Eurotiomycetes* | *Onygenales* | *Arthrodermataceae* | *Arthroderma quadrifidum* | *Arthroderma quadrifidum* | Sediment, soil | China, Hungary, Slovakia, Spain | 4 | Vanderwolf et al. (2013a); Zhang et al. (2017) |
| *Eurotiomycetes* | *Onygenales* | *Arthrodermataceae* | *Arthroderma silverae* | *Arthroderma silverae* | Sediment, soil | Canada, USA | 2 | Vanderwolf et al. (2013, 2015, 2019) |
| *Eurotiomycetes* | *Onygenales* | *Arthrodermataceae* | *Arthroderma* spp. | *Arthroderma* spp. | Bat guano, organic litter, sediment, soil | Canada, China, Hungary, Spain, Thailand, USA | 6 | Vanderwolf et al. (2013a, 2015); Zhang et al. (2014) |
| *Eurotiomycetes* | *Onygenales* | *Arthrodermataceae* | *Arthroderma terrestre* | *Arthroderma terrestre* | Air, soil-sediemnt, bat guano | Bulgaria, Canada, China, Czech Republic, France, Hungary, Romania, Spain, USA | 9 | Vanderwolf et al. (2013a); Zhang et al. (2014) |
| *Eurotiomycetes* | *Onygenales* | *Arthrodermataceae* | *Arthroderma terrestre* | *Trichophyton terrestre* | Air, soil-sediment, bat guano, other | Bulgaria, Canada, Czech Republic, France, Hungary, Romania, USA | 7 | Vanderwolf et al. (2013a) |
| *Eurotiomycetes* | *Onygenales* | *Arthrodermataceae* | *Arthroderma tuberculatum* | *Arthroderma tuberculatum* | Bat guano, sediment, soil | China, Hungary, Israel | 3 | Vanderwolf et al. (2013a); Zhang et al. (2021) |
| *Eurotiomycetes* | *Onygenales* | *Arthrodermataceae* | *Arthroderma uncinatum* | *Arthroderma uncinatum* | Soil-sediment | Hungary | 1 | Vanderwolf et al. (2013a) |
| *Eurotiomycetes* | *Onygenales* | *Arthrodermataceae* | *Arthroderma uncinatum* | *Trichophyton ajelloi* | Bat guano | Romania | 1 | Vanderwolf et al. (2013a) |
| *Eurotiomycetes* | *Onygenales* | *Arthrodermataceae* | *Arthroderma uncinatum* | *Trichophyton ajelloi* var. *ajelloi* | Bat guano | Romania | 1 | Vanderwolf et al. (2013a) |
| *Eurotiomycetes* | *Onygenales* | *Arthrodermataceae* | *Arthroderma vanbreuseghemii* | *Arthroderma vanbreuseghemii* | Cave soil | South Africa | 1 | Savković et al. (2025) |
| *Eurotiomycetes* | *Onygenales* | *Arthrodermataceae* | *Ctenomyces serratus* | *Ctenomyces serratus* | Soil-sediment | Hungary, Israel | 2 | Vanderwolf et al. (2013a) |
| *Eurotiomycetes* | *Onygenales* | *Arthrodermataceae* | *Ctenomyces vellereus* | *Ctenomyces vellereus* | Unreported | Unknow |  | Zhang et al. (2021) |
| *Eurotiomycetes* | *Onygenales* | *Arthrodermataceae* | *Microsporum canis* | *Microsporum canis* | Unreported | Columbia | 1 | Vanderwolf et al. (2013a) |
| *Eurotiomycetes* | *Onygenales* | *Arthrodermataceae* | *Microsporum gypseum* | *Microsporum gypseum* | Air,cave sediment, bat guano, soil-sediment, other | Greece, Hungary, Panama, Romania, South Africa, Spain, Tanzania, USA, Venezuela | 9 | Vanderwolf et al. (2013a); Nováková et al. (2018) |
| *Eurotiomycetes* | *Onygenales* | *Arthrodermataceae* | *Microsporum* sp. | *Microsporum* sp. | Unreported | Brazil | 1 | Vanderwolf et al. (2013a) |
| *Eurotiomycetes* | *Onygenales* | *Arthrodermataceae* | *Nannizzia fulva* | *Arthroderma fulvum* | Unreported | Spain | 1 | Vanderwolf et al. (2013a) |
| *Eurotiomycetes* | *Onygenales* | *Arthrodermataceae* | *Nannizzia fulva* | *Nannizzia fulva* | Unreported | Unknow |  | Vanderwolf et al. (2013a); Zhang et al. (2021) |
| *Eurotiomycetes* | *Onygenales* | *Arthrodermataceae* | *Paraphyton cookei* | *Paraphyton cookei* | Unreported | Slovakia | 1 | Ogórek et al. (2019) |
| *Eurotiomycetes* | *Onygenales* | *Arthrodermataceae* | *Shanorella spp.* | *Shanorella spp.* | Unreported | Canada, Thailand | 1 | Vanderwolf et al. (2013a); Suetrong et al. (2023) |
| *Eurotiomycetes* | *Onygenales* | *Arthrodermataceae* | *Trichophyton mentagrophytes* | *Trichophyton erinacei* | Bat guano | Slovakia | 1 | Vanderwolf et al. (2013a) |
| *Eurotiomycetes* | *Onygenales* | *Arthrodermataceae* | *Trichophyton mentagrophytes* | *Trichophyton mentagrophytes* | Air, bat guano, soil-sediment | Colombia, Greece, South Africa, USA | 4 | Vanderwolf et al. (2013a) |
| *Eurotiomycetes* | *Onygenales* | *Arthrodermataceae* | *Trichophyton rubrum* | *Trichophyton rubrum* | Bat guano | China, USA | 2 | Vanderwolf et al. (2013a); Zhang et al. (2017) |
| *Eurotiomycetes* | *Onygenales* | *Arthrodermataceae* | *Trichophyton schoenleinii* | *Trichophyton schoenleinii* | Wood | Switzerland | 1 | Vanderwolf et al. (2013a) |
| *Eurotiomycetes* | *Onygenales* | *Arthrodermataceae* | *Trichophyton* spp. | *Trichophyton* spp. | Air, soil-sediment, insects, bat guano | China, France, Hungary, India, Slovakia, UK, USA | 7 | Vanderwolf et al. (2013, 2019) |
| *Eurotiomycetes* | *Onygenales* | *Gymnoascaceae* | *Arachniotus dankaliensis* | *Arachniotus dankaliensis* | Bat guano | Mexico | 1 | Vanderwolf et al. (2013a); Zhang et al. (2021) |
| *Eurotiomycetes* | *Onygenales* | *Gymnoascaceae* | *Arachniotus dankaliensis* | *Gymnoascus dankaliensis* | Bat guano | Brazil, Mexico | 2 | Vanderwolf et al. (2013a); Cunha et al. (2020) |
| *Eurotiomycetes* | *Onygenales* | *Gymnoascaceae* | *Arachniotus* spp. | *Arachniotus* spp. | Live bat fur, cave wall, Organic litter, skin | Canada, Thailand, USA | 3 | Vanderwolf et al. (2013a); Suetrong et al. (2023) |
| *Eurotiomycetes* | *Onygenales* | *Gymnoascaceae* | *Arachniotus verruculosus* | *Arachniotus verruculosus* | Unreported | China | 1 | Zhang et al. 2021 |
| *Eurotiomycetes* | *Onygenales* | *Gymnoascaceae* | *Gymnascella citrina* | *Gymnascella citrina* | Bat guano | Mexico | 1 | Vanderwolf et al. (2013a) |
| *Eurotiomycetes* | *Onygenales* | *Gymnoascaceae* | *Gymnascella hyalinospora* | *Gymnascella hyalinospora* | Cave wall, sediment | Brazil, USA | 2 | Vanderwolf et al. (2013a); Alves et al. (2022) |
| *Eurotiomycetes* | *Onygenales* | *Gymnoascaceae* | *Gymnascella minnisii* | *Gymnascella minnisii* | Bat guano | USA | 1 | Savković et al. (2025) |
| *Eurotiomycetes* | *Onygenales* | *Gymnoascaceae* | Gymnoascaceae sp. | Gymnoascaceae sp. | Unreported | Thailand | 1 | Suetrong et al. (2023) |
| *Eurotiomycetes* | *Onygenales* | *Gymnoascaceae* | *Gymnoascoideus* sp. | *Gymnoascoideus* sp. | Soil-sediment | USA | 1 | Vanderwolf et al. (2013a) |
| *Eurotiomycetes* | *Onygenales* | *Gymnoascaceae* | *Gymnoascus exasperatus* | *Gymnoascus exasperatus* | Bat guano | China | 1 | Zhang et al. (2017) |
| *Eurotiomycetes* | *Onygenales* | *Gymnoascaceae* | *Gymnoascus flavus* | *Gymnoascus flavus* | Soil | China | 1 | zhang et al. (2021) |
| *Eurotiomycetes* | *Onygenales* | *Gymnoascaceae* | *Gymnoascus intermedius* | *Gymnoascus intermedius* | Gymnoascaceae sp. | Unreported | 1 | Vanderwolf et al. (2013a) |
| *Eurotiomycetes* | *Onygenales* | *Gymnoascaceae* | *Gymnoascus reessii* | *Gymnoascus reessii* | Air, dung, frass (not bat); soil-sediment, bat guano, paintings | Bulgaria, China, France, Hungary, Slovakia, Spain | 6 | Vanderwolf et al. (2013, 2019); Zhang et al. (2021) |
| *Eurotiomycetes* | *Onygenales* | *Gymnoascaceae* | *Gymnoascus* spp. | *Gymnoascus* spp. | Unreported, soil-sediment, dead bat fur, Organic litter, skin | Italy, Thailand, USA | 3 | Vanderwolf et al. (2013a); Suetrong et al. (2023) |
| *Eurotiomycetes* | *Onygenales* | *Gymnoascaceae* | *Gymnoascus udagawae* | *Gymnoascus udagawae* | Unreported | China | 1 | Zhang et al. (2021) |
| *Eurotiomycetes* | *Onygenales* | *Gymnoascaceae* | *Pseudoarachniotus trochleosporus* | *Pseudoarachniotus trochleosporus* | Cave wall | France | 1 | Vanderwolf et al. (2013a) |
| *Eurotiomycetes* | *Onygenales* | *Gymnoascaceae* | *Sporendonema rubrum* | *Arachniotus ruber* | Cave wall | USA | 1 | Vanderwolf et al. (2013a) |
| *Eurotiomycetes* | *Onygenales* | *Gymnoascaceae* | *Uncinocarpus uncinatus* | *Gymnoascus uncinatus* | Unreported | USA | 1 | Vanderwolf et al. (2013a) |
| *Eurotiomycetes* | *Onygenales* | [*Neoarthropsidaceae*](https://www.indexfungorum.org/Names/families.asp?FamilyName=Neoarthropsidaceae) | *Albidomyces albicans* | *Albidomyces albicans* | Cave wall | USA | 1 | Vanderwolf et al. (2013a); Zhang et al. (2021) |
| *Eurotiomycetes* | *Onygenales* | *Neoarthropsidaceae* | *Neoarthropsis hispanica* | *Arthropsis hispanica* | Organic litter | China | 1 | Zhang et al. (2017) |
| *Eurotiomycetes* | *Onygenales* | *Onygenaceae* | *Amauroascus kuehnii* | *Amauroascus kuehnii* | Air, bat guano | China, USA | 2 | Vanderwolf et al. (2013a); Zhang et al. (2021) |
| *Eurotiomycetes* | *Onygenales* | *Onygenaceae* | *Amauroascus* spp. | *Amauroascus* spp. | Cave wall | China, USA | 2 | Vanderwolf et al. (2013a); Zhang et al. (2021) |
| *Eurotiomycetes* | *Onygenales* | *Onygenaceae* | *Aphanoascus fulvescens* | *Aphanoascus fulvescens* | Bat guano, unreported, other, soil-sediment | China, France, Hungary, Mexico, Spain | 5 | Vanderwolf et al. (2013a); Zhang et al. (2021) |
| *Eurotiomycetes* | *Onygenales* | *Onygenaceae* | *Aphanoascus keratinophilus* | *Aphanoascus keratinophilus* | Soil, sediment | Slovakia | 1 | Zhang et al. (2021); Ogórek et al. 2022 |
| *Eurotiomycetes* | *Onygenales* | *Onygenaceae* | *Aphanoascus keratinophilus* | *Chrysosporium keratinophilum* | Soil-sediment, bat guano, air | Greece, Hungary, Israel, Romania, Spain | 5 | Vanderwolf et al. (2013a) |
| *Eurotiomycetes* | *Onygenales* | *Onygenaceae* | *Aphanoascus* sp. | *Aphanoascus* sp. | Unreported | Spain | 1 | Vanderwolf et al. (2013a) |
| *Eurotiomycetes* | *Onygenales* | *Onygenaceae* | *Arthroderma pannicola* | *Chrysosporium pannicola* | Soil-sediment | Hungary, Spain | 2 | zhang et al. (2021) |
| *Eurotiomycetes* | *Onygenales* | *Onygenaceae* | *Auxarthron alboluteum* | *Auxarthron alboluteum* | Sediment | China | 1 | Man et al. (2015) |
| *Eurotiomycetes* | *Onygenales* | *Onygenaceae* | *Auxarthron chinense* | *Auxarthron chinense* | Animal faeces, soil | China | 1 | zhang et al. (2021) |
| *Eurotiomycetes* | *Onygenales* | *Onygenaceae* | *Auxarthron guangxiense* | *Auxarthron guangxiense* | Soil | China | 1 | zhang et al. (2021) |
| *Eurotiomycetes* | *Onygenales* | *Onygenaceae* | Auxarthron spp. | Auxarthron spp. | Soil-sediment, cave wall | Thailand, USA | 2 | Vanderwolf et al. (2013a); Zhang et al. (2014); Suetrong et al. (2023) |
| *Eurotiomycetes* | *Onygenales* | *Onygenaceae* | *Auxarthronopsis globiasca* | *Auxarthronopsis globiasca* | Soil | China | 1 | zhang et al. (2021) |
| *Eurotiomycetes* | *Onygenales* | *Onygenaceae* | *Auxarthronopsis guizhouensis* | *Auxarthronopsis guizhouensis* | Air | China | 1 | Zhang et al. (2017) |
| *Eurotiomycetes* | *Onygenales* | *Onygenaceae* | *Auxarthronopsis pedicellaris* | *Auxarthronopsis pedicellaris* | Rock | China | 1 | zhang et al. (2021) |
| *Eurotiomycetes* | *Onygenales* | *Onygenaceae* | *Auxarthronopsis pulverea* | *Auxarthronopsis pulverea* | Plant debris | China | 1 | zhang et al. (2021) |
| *Eurotiomycetes* | *Onygenales* | *Onygenaceae* | *Auxarthronopsis sp.* | *Auxarthronopsis sp.* | Air | Spain | 1 | Savković et al. (2025) |
| *Eurotiomycetes* | *Onygenales* | *Onygenaceae* | *Auxarthronopsis stercicola* | *Auxarthronopsis stercicola* | Animal faeces | China | 1 | zhang et al. (2021) |
| *Eurotiomycetes* | *Onygenales* | *Onygenaceae* | *Chrysosporium carmichaelii* | *Chrysosporium carmichaelii* | Unreported | China | 1 | Zhang et al. (2021) |
| *Eurotiomycetes* | *Onygenales* | *Onygenaceae* | *Chrysosporium chiropterorum* | *Chrysosporium chiropterorum* | Unreported | France | 1 | Vanderwolf et al. (2013a) |
| *Eurotiomycetes* | *Onygenales* | *Onygenaceae* | *Chrysosporium europae* | *Chrysosporium europae* | Soil, sediment | Slovakia | 1 | Savković et al. (2025) |
| *Eurotiomycetes* | *Onygenales* | *Onygenaceae* | *Chrysosporium lobatum* | *Chrysosporium lobatum* | Bat guano | France | 1 | Vanderwolf et al. (2013a) |
| *Eurotiomycetes* | *Onygenales* | *Onygenaceae* | *Chrysosporium mephiticum* | *Chrysosporium mephiticum* | Cave soil | Puerto Rico | 1 | Savković et al. (2025) |
| *Eurotiomycetes* | *Onygenales* | *Onygenaceae* | *Chrysosporium merdarium* | *Chrysosporium merdarium* | Soil, sediment, animal bone | Canada, Czech Republic, France, Hungary, Italy, Poland, Russia | 7 | Vanderwolf et al. (2013a) |
| *Eurotiomycetes* | *Onygenales* | *Onygenaceae* | *Chrysosporium pallidum* | *Chrysosporium pallidum* | Animal faeces | China | 1 | Vanderwolf et al. (2013a) |
| *Eurotiomycetes* | *Onygenales* | *Onygenaceae* | *Chrysosporium speluncarum* | *Chrysosporium speluncarum* | Bat guano | Czech Republic, Romania, Slovakia | 3 | Vanderwolf et al. (2013a) |
| *Eurotiomycetes* | *Onygenales* | *Onygenaceae* | *Chrysosporium* spp. | *Chrysosporium* spp. | Unreported, soil-sediment, bat guano, cave wall, insects, air, earthworm casts | Belgium, Czech Republic, France, Hungary, India, Indonesia, Italy, Japan, Mexico, Nigeria, Russia, Slovakia, Spain, Thailand, USA | 15 | Vanderwolf et al. (2013a) |
| *Eurotiomycetes* | *Onygenales* | *Onygenaceae* | *Chrysosporium tropicum* | *Chrysosporium tropicum* | Soil-sediment | Israel, Spain | 2 | Vanderwolf et al. (2013a) |
| *Eurotiomycetes* | *Onygenales* | *Onygenaceae* | *Chrysosporium vallenarense* | *Chrysosporium vallenarense* | Sediment | USA | 1 | Savković et al. (2025) |
| *Eurotiomycetes* | *Onygenales* | *Onygenaceae* | *Coccidioides immitis* | *Coccidioides immitis* | Bat guano | USA | 1 | Vanderwolf et al. (2013a) |
| *Eurotiomycetes* | *Onygenales* | *Onygenaceae* | *Emmonsia* sp. | *Emmonsia* sp. | Unreported | China | 1 | Zhang et al. (2021) |
| *Eurotiomycetes* | *Onygenales* | *Onygenaceae* | *Keratinophyton wagneri* | *Keratinophyton wagneri* | Cave soil | Slovakia | 1 | Savković et al. (2025) |
| *Eurotiomycetes* | *Onygenales* | *Onygenaceae* | *Kuehniella racovitzae* | *Kuehniella racovitzae* | Wood, insects | France, Spain | 2 | Vanderwolf et al. (2013a) |
| *Eurotiomycetes* | *Onygenales* | *Onygenaceae* | *Leucothecium emdenii* | *Leucothecium emdenii* | Unreported | China | 1 | Zhang et al. (2021) |
| *Eurotiomycetes* | *Onygenales* | *Onygenaceae* | *Malbranchea aurantiaca* | *Malbranchea aurantiaca* | Bat guano, bat internal tissue | Mexico | 1 | Vanderwolf et al. (2013a) |
| *Eurotiomycetes* | *Onygenales* | *Onygenaceae* | *Malbranchea californiensis* | *Auxarthron californiense* | Bats | Canada, Slovakia | 2 | Vanderwolf et al. (2015); Nováková et al. (2018) |
| *Eurotiomycetes* | *Onygenales* | *Onygenaceae* | *Malbranchea cavernosa* | *Malbranchea cavernosa* | Air, sediment | Brazil | 1 | Alves et al. (2022); Savković et al. (2025) |
| *Eurotiomycetes* | *Onygenales* | *Onygenaceae* | *Malbranchea* spp. | *Malbranchea* spp. | Air, soil-sediment, bat guano, Earthworm casts | China, Slovakia | 1 | Vanderwolf et al. (2013a) |
| *Eurotiomycetes* | *Onygenales* | *Onygenaceae* | *Malbranchea thaxteri* | *Auxarthron thaxteri* | Cave wall | USA | 1 | Vanderwolf et al. (2013a) |
| *Eurotiomycetes* | *Onygenales* | *Onygenaceae* | *Malbranchea umbrina* | *Malbranchea umbrina* | Unreported | China | 1 | Zhang et al. (2017) |
| *Eurotiomycetes* | *Onygenales* | *Onygenaceae* | Neogymnomyces spp. | Neogymnomyces spp. | Soil-sediment | China, USA | 2 | Vanderwolf et al. (2013a) |
| *Eurotiomycetes* | *Onygenales* | *Onygenaceae* | *Neogymnomyces virgineus* | *Neogymnomyces virgineus* | Dung, frass (not bat) | Italy | 1 | Suetrong et al. (2023) |
| *Eurotiomycetes* | *Onygenales* | *Onygenaceae* | *Paranannizziopsis* sp. | *Paranannizziopsis* sp. | Organic litter, | Thailand | 1 | Suetrong et al. (2023) |
| *Eurotiomycetes* | *Onygenales* | *Onygenaceae* | *Pseudogeomyces pseudomerdarius* | *Chrysosporium pseudomerdarium* | Air, bat guano, soil-sediment | Canada, Czech Republic, France, Japan | 4 | Vanderwolf et al. (2013a) |
| *Eurotiomycetes* | *Onygenales* | *Onygenaceae* | *Spiromastigoides gypsea* | *Malbranchea gypsea* | Soil-sediment, bat guano | Nigeria | 1 | Vanderwolf et al. (2013a) |
| *Eurotiomycetes* | *Onygenales* | *Onygenaceae* | *Uncinocarpus uncinatus* | *Uncinocarpus uncinatus* | Unreported | USA | 1 | Vanderwolf et al. (2013a) |
| *Eurotiomycetes* | *Onygenales* | *Spiromastigaceae* | *Spiromastix sp.* | *Spiromastix sp.* | Soil | Belgium | 1 | Savković et al. (2025) |
| *Eurotiomycetes* | *Onygenales* |  | Onygenales sp. | Onygenales sp. | Unreported | Thailand | 1 | Vanderwolf et al. (2013a) |
| *Hyphomycetes* |  |  | Hyphomycetes sp. | Hyphomycetes sp. | Soil-sediment | Japan | 1 | Vanderwolf et al. (2013a) |
| *Incertae sedis* | *Incertae sedis* | *Incertae sedis* | *Acrocylindrium* sp. | *Acrocylindrium* sp. | Soil-sediment | Poland | 1 | Vanderwolf et al. (2013a) |
| *Incertae sedis* | *Incertae sedis* | *Incertae sedis* | *Acrophialophora fusispora* | *Acrophialophora fusispora* | Other | Czech Republic | 1 | Vanderwolf et al. (2013a) |
| *Incertae sedis* | *Incertae sedis* | *Incertae sedis* | *Ajellomyces capsulatus* | *Ajellomyces capsulatus* | Wood | France | 1 | Vanderwolf et al. (2013a); Zhang et al. (2021) |
| *Incertae sedis* | *Incertae sedis* | *Incertae sedis* | *Ajellomyces* sp. | *Ajellomyces* sp. | Unreported | China | 1 | Zhang et al. (2021) |
| *Incertae sedis* | *Incertae sedis* | *Incertae sedis* | *Amblyosporium botrytis* | *Amblyosporium botrytis* | Wood | Switzerland | 1 | Vanderwolf et al. (2013a) |
| *Incertae sedis* | *Incertae sedis* | *Incertae sedis* | *Arxiella* sp. | *Arxiella* sp. | Unreported | China | 1 | Zhang et al. (2021) |
| *Incertae sedis* | *Incertae sedis* | *Incertae sedis* | *Bispora antennata* | *Bispora antennata* | Soil-sediment, wood | Belgium, Italy | 2 | Vanderwolf et al. (2013a) |
| *Incertae sedis* | *Incertae sedis* | *Incertae sedis* | *Bispora betulina* | *Bispora betulina* | Wood | Romania | 1 | Vanderwolf et al. (2013a) |
| *Incertae sedis* | *Incertae sedis* | *Incertae sedis* | *Bispora effusa* | *Bispora effusa* | Wood | South Africa | 1 | Vanderwolf et al. (2013a) |
| *Incertae sedis* | *Incertae sedis* | *Incertae sedis* | *Bispora sp.* | *Bispora* sp. | Air | Spain | 1 | Vanderwolf et al. (2013a) |
| *Incertae sedis* | *Incertae sedis* | *Incertae sedis* | *Blastotrichum sp.* | *Blastotrichum sp.* | Wood | Belgium | 1 | Vanderwolf et al. (2013a) |
| *Incertae sedis* | *Incertae sedis* | *Incertae sedis* | *Botryosporium longibrachiatum* | *Botryosporium longibrachiatum* | Other | China, Slovakia | 2 | Vanderwolf et al. (2013a); Man (2017) |
| *Incertae sedis* | *Incertae sedis* | *Incertae sedis* | *Corallinopsis pilulifera* | *Corallinopsis pilulifera* | Insects | Austria, France | 2 | Vanderwolf et al. (2013a) |
| *Incertae sedis* | *Incertae sedis* | *Incertae sedis* | *Culicinomyces* sp. | *Culicinomyces* sp. | Unreported | Canada | 1 | Vanderwolf et al. (2019) |
| *Incertae sedis* | *Incertae sedis* | *Incertae sedis* | *Dactylosporium* sp. | *Dactylosporium* sp. | Soil-sediment | Poland | 1 | Vanderwolf et al. (2013a) |
| *Incertae sedis* | *Incertae sedis* | *Incertae sedis* | *Dendrosporium lobatum* | *Dendrosporium lobatum* | Soil-sediment, water | Puerto Rico | 1 | Vanderwolf et al. (2013a) |
| *Incertae sedis* | *Incertae sedis* | *Incertae sedis* | *Endophoma elongata* | *Endophoma elongata* | Soil | Canada | 1 | Vanderwolf et al. (2013a) |
| *Incertae sedis* | *Incertae sedis* | *Incertae sedis* | *Fusariella* sp. | *Fusariella* sp. | Air | Spain | 1 | Vanderwolf et al. (2013a) |
| *Incertae sedis* | *Incertae sedis* | *Incertae sedis* | *Gamsia aggregata* | *Gamsia aggregata* | Unreported | China | 1 | Zhang et al. (2021) |
| *Incertae sedis* | *Incertae sedis* | *Incertae sedis* | *Gamsia columbina* | *Gamsia columbina* | Unreported | China | 1 | Zhang et al. (2021) |
| *Incertae sedis* | *Incertae sedis* | *Incertae sedis* | *Gamsia simplex* | *Gamsia simplex* | Soil | China | 1 | Jiang et al. (2017) |
| *Incertae sedis* | *Incertae sedis* | *Incertae sedis* | Gamsia sp. | Gamsia sp. | Soil | Belgium | 1 | Savković et al. (2025) |
| *Incertae sedis* | *Incertae sedis* | *Incertae sedis* | *Gilmaniella* sp. | *Gilmaniella* sp. | Soil-sediment | Puerto Rico | 1 | Vanderwolf et al. (2013a) |
| *Incertae sedis* | *Incertae sedis* | *Incertae sedis* | *Graphiothecium* sp. | *Graphiothecium* sp. | Soil-sediment | Bahamas | 1 | Vanderwolf et al. (2013a) |
| *Incertae sedis* | *Incertae sedis* | *Incertae sedis* | *Gyrothrix* sp. | *Gyrothrix* sp. | Unreported | China | 1 | Zhang et al. (2017) |
| *Incertae sedis* | *Incertae sedis* | *Incertae sedis* | *Hormiactis candida* | *Hormiactis candida* | Soil-sediment | USA | 1 | Vanderwolf et al. (2013a) |
| *Incertae sedis* | *Incertae sedis* | *Incertae sedis* | *Hormiactis* spp. | *Hormiactis* spp. | Unreported, bat guano, earthworm casts | Romania, Slovakia, USA | 3 | Vanderwolf et al. (2013a) |
| *Incertae sedis* | *Incertae sedis* | *Incertae sedis* | *Incertae sedis* | *Veronaea compacta* | Unreported | China | 1 | Zhang et al. (2021) |
| *Incertae sedis* | *Incertae sedis* | *Incertae sedis* | *Isthmolongispora quadricellularis* | *Isthmolongispora quadricellularis* | Water, soil-sediment | Puerto Rico | 1 | Vanderwolf et al. (2013a) |
| *Incertae sedis* | *Incertae sedis* | *Incertae sedis* | *Monosporium* sp. | *Monosporium* sp. | Soil-sediment | Venezuela | 1 | Vanderwolf et al. (2013a) |
| *Incertae sedis* | *Incertae sedis* | *Incertae sedis* | *Ochroconis musae* | *Ochroconis* cf. *musae* | Bat | Brazil | 1 | Cunha et al. (2020) |
| *Incertae sedis* | *Incertae sedis* | *Incertae sedis* | *Ochroconis* spp. | *Ochroconis* spp. | Air, cave wall, sediment | California, China, France | 3 | Vanderwolf et al. (2013a) |
| *Incertae sedis* | *Incertae sedis* | *Incertae sedis* | *Ochroconis tshawytschae* | *Ochroconis tshawytschae* | Cave wall | France | 1 | Vanderwolf et al. (2013a) |
| *Incertae sedis* | *Incertae sedis* | *Incertae sedis* | *Ochroconis tshawytschae* | *Scolecobasidium tshawytschae* | Cave wall, air | France, Slovakia | 2 | Vanderwolf et al. (2013a) |
| *Incertae sedis* | *Incertae sedis* | *Incertae sedis* | *Ovadendron sulphureo-ochraceum* | *Ovadendron sulphureoochraceum* | Air | Czech Republic | 1 | Vanderwolf et al. (2013a) |
| *Incertae sedis* | *Incertae sedis* | *Incertae sedis* | *Phaeocytostroma ambiguum* | *Phaeocytostroma ambiguum* | Unreported | China | 1 | Zhang et al. (2021) |
| *Incertae sedis* | *Incertae sedis* | *Incertae sedis* | *Phaeocytostroma sacchari* | *Phaeocytostroma sacchari* | Unreported | China | 1 | Zhang et al. (2021) |
| *Incertae sedis* | *Incertae sedis* | *Incertae sedis* | *Pirostoma* sp. | *Pirostoma* sp. | Unreported | France | 1 | Vanderwolf et al. (2013a) |
| *Incertae sedis* | *Incertae sedis* | *Incertae sedis* | *Polyschema* sp. | Polyschema sp. | Bat | Brazil | 1 | Cunha et al. (2020) |
| *Incertae sedis* | *Incertae sedis* | *Incertae sedis* | *Sarcopodium* sp. | *Sarcopodium* sp. | Unreported | China | 1 | Zhang et al. (2021) |
| *Incertae sedis* | *Incertae sedis* | *Incertae sedis* | *Scolecobasidium anellii* | *Ochroconis anellii* | Cave wall, soil-sediment | Czech Republic, Italy | 2 | Vanderwolf et al. (2013a) |
| *Incertae sedis* | *Incertae sedis* | *Incertae sedis* | *Scolecobasidium anellii* | *Scolecobasidium anellii* | Cave wall, soil-sediment | Czech Republic, Italy | 2 | Vanderwolf et al. (2013a) |
| *Incertae sedis* | *Incertae sedis* | *Incertae sedis* | *Scolecobasidium anomalum* | *Ochroconis anomala* | Cave wall | France | 1 | Vanderwolf et al. (2013a) |
| *Incertae sedis* | *Incertae sedis* | *Incertae sedis* | *Scolecobasidium anomalum* | *Scolecobasidium anomalum* | Sediment | France | 1 | Vanderwolf et al. (2013a) |
| *Incertae sedis* | *Incertae sedis* | *Incertae sedis* | *Scolecobasidium constrictum* | *Ochroconis constricta* | Air, soil-sediment | Czech Republic, Russia | 2 | Vanderwolf et al. (2013a) |
| *Incertae sedis* | *Incertae sedis* | *Incertae sedis* | *Scolecobasidium constrictum* | *Scolecobasidium constrictum* | Air, soil-sediment | Czech Republic, Russia | 2 | Vanderwolf et al. (2013a) |
| *Incertae sedis* | *Incertae sedis* | *Incertae sedis* | *Scolecobasidium globale* | *Scolecobasidium globale* | Asymptomatic specimens of Proteus anguinus | Slovenia | 1 | Savković et al. (2025) |
| *Incertae sedis* | *Incertae sedis* | *Incertae sedis* | *Scolecobasidium lascauxense* | *Ochroconis lascauxensis* | Cave wall | France | 1 | Vanderwolf et al. (2013a) |
| *Incertae sedis* | *Incertae sedis* | *Incertae sedis* | *Scolecobasidium lascauxense* | *Scolecobasidium lascauxense* | Air, cave wall, sediment | France | 1 | Vanderwolf et al. (2013a) |
| *Incertae sedis* | *Incertae sedis* | *Incertae sedis* | *Scolecobasidium* sp. | *Scolecobasidium* sp. | Air, Organic litter, | Thailand | 1 | Suetrong et al. (2023) |
| *Incertae sedis* | *Incertae sedis* | *Incertae sedis* | *Scolecobasidium terreum* | *Scolecobasidium terreum* | Unreported | Italy | 1 | Vanderwolf et al. (2013a) |
| *Incertae sedis* | *Incertae sedis* | *Incertae sedis* | *Septonema secedens* | *Septonema secedens* | Unreported | Canada | 1 | Vanderwolf et al. (2013a) |
| *Incertae sedis* | *Incertae sedis* | *Incertae sedis* | *Septonema* spp. | *Septonema* spp. | Air | China, Spain | 2 | Vanderwolf et al. (2013a); Jiang et al. (2017a, b) |
| *Incertae sedis* | *Incertae sedis* | *Incertae sedis* | *Staphylotrichum boninense* | *Staphylotrichum boninense* | Unreported | China | 1 | Zhang et al. (2017) |
| *Incertae sedis* | *Incertae sedis* | *Incertae sedis* | *Staphylotrichum coccosporum* | *Staphylotrichum coccosporum* | Air | China, South Africa | 2 | Vanderwolf et al. (2013a) |
| *Incertae sedis* | *Incertae sedis* | *Incertae sedis* | *Staphylotrichum limonisporum* | *Humicola limonispora* | Unreported | China | 1 | Zhang et al. (2017) |
| *Sordariomycetes* | *Sordariales* | *Chaetomiaceae* | *Pseudohumicola debertoldii* | *Humicola lutea* | Sediment | Brazil | 1 | Alves et al. (2022) |
| *Sordariomycetes* | *Sordariales* | *Chaetomiaceae* | *Pseudohumicola cecavii* | *Pseudohumicola cecavii* | Air, sediment | Brazil | 1 | Alves et al. (2022) |
| *Sordariomycetes* | *Hypocreales* | *Cordycipitaceae* | *Pseudolecanicillium caatingaense* | *Pseudolecanicillium caatingaense* | Air | Brazil | 1 | Alves et al. (2022) |
| *Sordariomycetes* | *Sordariales* | [*Chaetomiaceae*](https://www.indexfungorum.org/Names/families.asp?FamilyName=Chaetomiaceae) | *Humicola* sp. | Humicola sp. | Sediment | Brazil | 1 | Alves et al. (2022) |
| *Incertae sedis* | *Incertae sedis* | *Incertae sedis* | *Staphylotrichum* sp. | *Staphylotrichum* sp. | Unreported | China | 1 | Zhang et al. (2017) |
| *Incertae sedis* | *Incertae sedis* | *Incertae sedis* | *Tetracoccosporium paxianum* | *Tetracoccosporium paxianum* | Soil-sediment, insects | Slovakia | 1 | Vanderwolf et al. (2013a) |
| *Incertae sedis* | *Incertae sedis* | *Incertae sedis* | *Tricellula aquatica* | *Tricellula* cf. *aquatica* | Unreported | Canada | 1 | Vanderwolf et al. (2019) |
| *Incertae sedis* | *Incertae sedis* | *Incertae sedis* | *Trichobotrys effusus* | *Trichobotrys effusa* | Soil-sediment | Japan | 1 | Vanderwolf et al. (2013a) |
| *Incertae sedis* | *Incertae sedis* | *Incertae sedis* | *Trichobotrys effusus* | *Trichobotrys effusus* | Unreported | Unknow |  | Zhang et al. (2021) |
| *Incertae sedis* | *Incertae sedis* | *Incertae sedis* | *Trichobotrys* spp. | *Trichobotrys* spp | Organic litter | China, Thailand | 1 | Zhang (2019); Suetrong et al. (2023) |
| *Incertae sedis* | *Incertae sedis* | *Incertae sedis* | *Trichosporiella cerebriformis* | *Trichosporiella cerebriformis* | Air, soil-sediment | Czech Republic, Russia,, Slovakia | 3 | Vanderwolf et al. (2013a) |
| *Incertae sedis* | *Incertae sedis* | *Incertae sedis* | *Trichosporiella multisporum* | *Trichosporiella multisporum* | Unreported | Canada | 1 | Vanderwolf et al. (2013a) |
| *Incertae sedis* | *Incertae sedis* | *Incertae sedis* | *Trichosporiella* sp. | *Trichosporiella* sp. | Unreported | Canada | 1 | Vanderwolf et al. (2013, 2015, 2019) |
| *Incertae sedis* | *Incertae sedis* | *Incertae sedis* | *Xepicula* spp. | *Xepicula* spp. | Unreported | China | 1 | Zhang et al. (2021) |
| *Incertae sedis* | *Incertae sedis* | *Incertae sedis* | *Yunnania carbonaria* | *Yunnania carbonaria* | Unreported | China | 1 | Zhang et al. (2021) |
| *Incertae sedis* | *Incertae sedis* | *Incertae sedis* | *Yunnania penicillata* | *Yunnania penicillata* | Unreported | China | 1 | Zhang et al. (2021) |
| *Incertae sedis* | *Incertae sedis* | *Stilbosporaceae* | Prosthecium sp. | Prosthecium sp. | Unreported | China | 1 | Zhang et al. (2021) |
| *Incertae sedis* | *Incertae sedis* | *Tarzettaceae* | *Tarzetta* sp. | *Tarzetta* sp. | Unreported | Croatia | 1 | Vanderwolf et al. (2013a) |
| *Incertae sedis* | *Lauriomycetales* | *Lauriomycetaceae* | *Dematioscypha catenata* | *Lauriomyces catenatus* | Soil-sediment | Italy | 1 | Vanderwolf et al. (2013a) |
| *Kickxellomycetes* | *Kickxellales* | *Kickxellaceae* | *Linderina pennispora* | *Linderina pennispora* | Soil-sediment | Russia | 1 | Vanderwolf et al. (2013a) |
| *Kickxellomycetes* | *Kickxellales* | *Kickxellaceae* | *Ramicandelaber* sp. | *Ramicandelaber* sp. | Soil-sediment, | Thailand | 1 | Suetrong et al. (2023) |
| *Laboulbeniomycetes* | *Herpomycetales* | *Herpomycetaceae* | *Herpomyces arietinus* | *Herpomyces arietinus* | Insects | USA | 1 | Vanderwolf et al. (2013a) |
| *Laboulbeniomycetes* | *Laboulbeniales* | *Euceratomycetaceae* | *Cochliomyces trinitatis* | *Cochliomyces trinitatis* | Insects | Cuba, Trinidad and Tobago | 2 | Vanderwolf et al. (2013a) |
| *Laboulbeniomycetes* | *Laboulbeniales* | *Laboulbeniaceae* | *Arthrorhynchus acrandros* | *Arthrorhynchus acrandros* | Insects | Italy | 1 | Vanderwolf et al. (2013a) |
| *Laboulbeniomycetes* | *Laboulbeniales* | *Laboulbeniaceae* | *Arthrorhynchus eucampsipodae* | *Arthrorhynchus eucampsipodae* | Bat | Bulgaria | 1 | Savković et al. (2025) |
| *Laboulbeniomycetes* | *Laboulbeniales* | *Laboulbeniaceae* | *Arthrorhynchus nycteribiae* | *Arthrorhynchus nycteribiae* | Insects, bat | Bulgaria, Slovakia | 2 | Vanderwolf et al. (2013a) |
| *Laboulbeniomycetes* | *Laboulbeniales* | *Laboulbeniaceae* | *Asaphomyces tubanticus* | *Asaphomyces tubanticus* | Insects | Sweden | 1 | Vanderwolf et al. (2013a) |
| *Laboulbeniomycetes* | *Laboulbeniales* | *Laboulbeniaceae* | *Compsomyces lestevae* | *Compsomyces lestevae* | Insect | France | 1 | Vanderwolf et al. (2013a) |
| *Laboulbeniomycetes* | *Laboulbeniales* | *Laboulbeniaceae* | *Compsomyces lestevae* | *Compsomyces lestevi* | Insects | France | 1 | Vanderwolf et al. (2013a) |
| *Laboulbeniomycetes* | *Laboulbeniales* | *Laboulbeniaceae* | *Diplopodomyces callipodos* | *Diplopodomyces callipodos* | Insects | France, Italy | 2 | Vanderwolf et al. (2013a) |
| *Laboulbeniomycetes* | *Laboulbeniales* | *Laboulbeniaceae* | *Laboulbenia arawaka* | *Laboulbenia arawaka* | Insects | Guadeloupe, Spain, Venezuela | 3 | Vanderwolf et al. (2013a) |
| *Laboulbeniomycetes* | *Laboulbeniales* | *Laboulbeniaceae* | *Laboulbenia bolivarii* | *Laboulbenia bolivarii* | Insects | Spain | 1 | Vanderwolf et al. (2013a) |
| *Laboulbeniomycetes* | *Laboulbeniales* | *Laboulbeniaceae* | *Laboulbenia bordonii* | *Laboulbenia bordonii* | Insects | Venezuela | 1 | Vanderwolf et al. (2013a) |
| *Laboulbeniomycetes* | *Laboulbeniales* | *Laboulbeniaceae* | *Laboulbenia cantabrica* | *Laboulbenia cantabrica* | Insects | Spain | 1 | Vanderwolf et al. (2013a) |
| *Laboulbeniomycetes* | *Laboulbeniales* | *Laboulbeniaceae* | *Laboulbenia coiffaitii* | *Laboulbenia coiffaitii* | Insects | France, Italy, Spain | 3 | Vanderwolf et al. (2013a) |
| *Laboulbeniomycetes* | *Laboulbeniales* | *Laboulbeniaceae* | *Laboulbenia flagellata* | *Laboulbenia flagellata* | Insects | Romania | 1 | Vanderwolf et al. (2013a) |
| *Laboulbeniomycetes* | *Laboulbeniales* | *Laboulbeniaceae* | *Laboulbenia lecoareri* | *Laboulbenia lecoareri* | insect | France | 1 | Vanderwolf et al. (2013a) |
| *Laboulbeniomycetes* | *Laboulbeniales* | *Laboulbeniaceae* | *Laboulbenia lecoareri* | *Laboulbenia subterranea subsp. lecoareri* | Insects | France | 1 | Vanderwolf et al. (2013a) |
| *Laboulbeniomycetes* | *Laboulbeniales* | *Laboulbeniaceae* | *Laboulbenia nebriae* | *Laboulbenia nebriae* | Insects | Morocco | 1 | Vanderwolf et al. (2013a) |
| *Laboulbeniomycetes* | *Laboulbeniales* | *Laboulbeniaceae* | *Laboulbenia nebriae* | *Laboulbenia nebriae subsp. maghrebiana* | Insects | Morocco | 1 | Vanderwolf et al. (2013a) |
| *Laboulbeniomycetes* | *Laboulbeniales* | *Laboulbeniaceae* | *Laboulbenia orghidanii* | *Laboulbenia orghidanii* | Insects | Venezuela | 1 | Vanderwolf et al. (2013a) |
| *Laboulbeniomycetes* | *Laboulbeniales* | *Laboulbeniaceae* | *Laboulbenia picardii* | *Laboulbenia picardii* | Insects | Italy | 1 | Vanderwolf et al. (2013a) |
| *Laboulbeniomycetes* | *Laboulbeniales* | *Laboulbeniaceae* | *Laboulbenia polyphaga* | *Laboulbenia polyphaga* | Insects | Algeria | 1 | Vanderwolf et al. (2013a) |
| *Laboulbeniomycetes* | *Laboulbeniales* | *Laboulbeniaceae* | *Laboulbenia sbordonii* | *Laboulbenia sbordonii* | Insects | Mexico | 1 | Vanderwolf et al. (2013a) |
| *Laboulbeniomycetes* | *Laboulbeniales* | *Laboulbeniaceae* | *Laboulbenia shanorii* | *Laboulbenia shanorii* | Insects | Albania, Bosnia, Croatia | 3 | Vanderwolf et al. (2013a) |
| *Laboulbeniomycetes* | *Laboulbeniales* | *Laboulbeniaceae* | *Laboulbenia* sp. | *Laboulbenia* sp. | Unreported | Croatia | 1 | Vanderwolf et al. (2013a) |
| *Laboulbeniomycetes* | *Laboulbeniales* | *Laboulbeniaceae* | *Laboulbenia stilicicola* | *Laboulbenia stilicicola* | Insects | USA | 1 | Vanderwolf et al. (2013a) |
| *Laboulbeniomycetes* | *Laboulbeniales* | *Laboulbeniaceae* | *Laboulbenia subterranea* | *Laboulbenia subterranea* | Insects | Czech Republic, France, Hungary, Italy, Japan, Romania | 6 | Vanderwolf et al. (2013a) |
| *Laboulbeniomycetes* | *Laboulbeniales* | *Laboulbeniaceae* | *Laboulbenia vignae* | *Laboulbenia vignae* | Insects | Turkey | 1 | Vanderwolf et al. (2013a) |
| *Laboulbeniomycetes* | *Laboulbeniales* | *Laboulbeniaceae* | *Laboulbenia vulgaris* | *Laboulbenia vulgaris* | Insects | Romania | 1 | Vanderwolf et al. (2013a) |
| *Laboulbeniomycetes* | *Laboulbeniales* | *Laboulbeniaceae* | *Laboulbenia vulgaris* | *Laboulbenia vulgaris* subsp. *trechiphila* | Insects | France, Italy, Japan, Romania, Slovenia | 5 | Vanderwolf et al. (2013a) |
| *Laboulbeniomycetes* | *Laboulbeniales* | *Laboulbeniaceae* | *Laboulbenia vulgaris* | *Laboulbenia vulgaris* var*. extensa* | Insects | Romania | 1 | Vanderwolf et al. (2013a) |
| *Laboulbeniomycetes* | *Laboulbeniales* | *Laboulbeniaceae* | *Rhachomyces alluaudii* | *Rhachomyces alluaudii* | Insects | Morocco | 1 | Vanderwolf et al. (2013a) |
| *Laboulbeniomycetes* | *Laboulbeniales* | *Laboulbeniaceae* | *Rhachomyces anophthalmi* | *Rhachomyces anophthalmi* | Insects | Slovenia | 1 | Vanderwolf et al. (2013a) |
| *Laboulbeniomycetes* | *Laboulbeniales* | *Laboulbeniaceae* | *Rhachomyces aphaenopsis* | *Rhachomyces aphaenopsis* | Insects | Austria, Croatia, France, Slovenia | 4 | Vanderwolf et al. (2013a) |
| *Laboulbeniomycetes* | *Laboulbeniales* | *Laboulbeniaceae* | *Rhachomyces aphaenopsis* | Rhachomyces aphaenopsis var. jeannelii | Insects | Austria, Croatia, France, Slovenia | 4 | Vanderwolf et al. (2013a) |
| *Laboulbeniomycetes* | *Laboulbeniales* | *Laboulbeniaceae* | *Rhachomyces beronii* | *Rhachomyces beronii* | Insects | New Guinea | 1 | Vanderwolf et al. (2013a) |
| *Laboulbeniomycetes* | *Laboulbeniales* | *Laboulbeniaceae* | *Rhachomyces bolivarii* | *Rhachomyces bolivarii* | Insects | Spain | 1 | Vanderwolf et al. (2013a) |
| *Laboulbeniomycetes* | *Laboulbeniales* | *Laboulbeniaceae* | *Rhachomyces bucciarellii* | *Rhachomyces bucciarellii* | Insects | Italy | 1 | Vanderwolf et al. (2013a) |
| *Laboulbeniomycetes* | *Laboulbeniales* | *Laboulbeniaceae* | *Rhachomyces canariensis* | *Rhachomyces canariensis* | Insects | Spain | 1 | Vanderwolf et al. (2013a) |
| *Laboulbeniomycetes* | *Laboulbeniales* | *Laboulbeniaceae* | *Rhachomyces capucinus* | *Rhachomyces capucinus* | Insects | France | 1 | Vanderwolf et al. (2013a) |
| *Laboulbeniomycetes* | *Laboulbeniales* | *Laboulbeniaceae* | *Rhachomyces dedyi* | *Rhachomyces dedyi* | Insects | France, Romania | 2 | Vanderwolf et al. (2013a) |
| *Laboulbeniomycetes* | *Laboulbeniales* | *Laboulbeniaceae* | *Rhachomyces girardii* | *Rhachomyces girardii* | Insects | France, Spain | 2 | Vanderwolf et al. (2013a) |
| *Laboulbeniomycetes* | *Laboulbeniales* | *Laboulbeniaceae* | *Rhachomyces gratiellae* | *Rhachomyces gratiellae* | Insects | Turkey | 1 | Vanderwolf et al. (2013a) |
| *Laboulbeniomycetes* | *Laboulbeniales* | *Laboulbeniaceae* | *Rhachomyces hypogaeus* | *Rhachomyces hypogaeus* | Insects | Bosnia, Croatia, France, Slovenia | 4 | Vanderwolf et al. (2013a) |
| *Laboulbeniomycetes* | *Laboulbeniales* | *Laboulbeniaceae* | *Rhachomyces hypogaeus* | Rhachomyces hypogaeus subsp. richardii | Insects | France | 1 | Vanderwolf et al. (2013a) |
| *Laboulbeniomycetes* | *Laboulbeniales* | *Laboulbeniaceae* | *Rhachomyces ilerdensis* | *Rhachomyces ilerdensis* | Insects | Spain | 1 | Vanderwolf et al. (2013a) |
| *Laboulbeniomycetes* | *Laboulbeniales* | *Laboulbeniaceae* | *Rhachomyces maublancii* | *Rhachomyces maublancii* | Insects | France, Italy, Romania | 3 | Vanderwolf et al. (2013a) |
| *Laboulbeniomycetes* | *Laboulbeniales* | *Laboulbeniaceae* | *Rhachomyces middelhoekii* | *Rhachomyces middelhoekii* | Insects | Bulgaria | 1 | Vanderwolf et al. (2013a) |
| *Laboulbeniomycetes* | *Laboulbeniales* | *Laboulbeniaceae* | *Rhachomyces orotrechorum* | *Rhachomyces orotrechorum* | Insects | Italy | 1 | Vanderwolf et al. (2013a) |
| *Laboulbeniomycetes* | *Laboulbeniales* | *Laboulbeniaceae* | *Rhachomyces pacei* | *Rhachomyces pacei* | Insects | Italy | 1 | Vanderwolf et al. (2013a) |
| *Laboulbeniomycetes* | *Laboulbeniales* | *Laboulbeniaceae* | *Rhachomyces peyerimhoffii* | *Rhachomyces peyerimhoffii* | Insects | Algeria | 1 | Vanderwolf et al. (2013a) |
| *Laboulbeniomycetes* | *Laboulbeniales* | *Laboulbeniaceae* | *Rhachomyces proliferans* | *Rhachomyces proliferans* | Insects | Hungary, Romania | 2 | Vanderwolf et al. (2013a) |
| *Laboulbeniomycetes* | *Laboulbeniales* | *Laboulbeniaceae* | *Rhachomyces pyrenaeus* | *Rhachomyces pyrenaeus* | Insects | Spain | 1 | Vanderwolf et al. (2013a) |
| *Laboulbeniomycetes* | *Laboulbeniales* | *Laboulbeniaceae* | *Rhachomyces quetzalcoatl* | *Rhachomyces quetzalcoatl* | Insects | Mexico | 1 | Vanderwolf et al. (2013a) |
| *Laboulbeniomycetes* | *Laboulbeniales* | *Laboulbeniaceae* | *Rhachomyces reveilletii* | *Rhachomyces reveilletii* | Insects | France | 1 | Vanderwolf et al. (2013a) |
| *Laboulbeniomycetes* | *Laboulbeniales* | *Laboulbeniaceae* | *Rhachomyces reymondi* | *Rhachomyces reymondi* | Insects | France | 1 | Vanderwolf et al. (2013a) |
| *Laboulbeniomycetes* | *Laboulbeniales* | *Laboulbeniaceae* | *Rhachomyces richardi* | *Rhachomyces richardi* | Insects | France, Italy | 2 | Vanderwolf et al. (2013a) |
| *Laboulbeniomycetes* | *Laboulbeniales* | *Laboulbeniaceae* | *Rhachomyces spadiceus* | *Rhachomyces spadiceus* | Insects | France | 1 | Vanderwolf et al. (2013a) |
| *Laboulbeniomycetes* | *Laboulbeniales* | *Laboulbeniaceae* | *Rhachomyces speluncalis* | *Rhachomyces speluncalis* | Insects | USA | 1 | Vanderwolf et al. (2013a) |
| *Laboulbeniomycetes* | *Laboulbeniales* | *Laboulbeniaceae* | *Rhachomyces* spp. | *Rhachomyces* spp. | Insects, unreported | Croatia, France, Italy, New Guinea | 4 | Vanderwolf et al. (2013a) |
| *Laboulbeniomycetes* | *Laboulbeniales* | *Laboulbeniaceae* | *Rhachomyces stipitatus* | *Rhachomyces stipitatus* | Insects | Algeria, France, Italy, Spain | 4 | Vanderwolf et al. (2013a) |
| *Laboulbeniomycetes* | *Laboulbeniales* | *Laboulbeniaceae* | *Rhachomyces stipitatus* | *Rhachomyces stipitatus var. pallidus* | Insects | Algeria, France | 2 | Vanderwolf et al. (2013a) |
| *Laboulbeniomycetes* | *Laboulbeniales* | *Laboulbeniaceae* | *Rhachomyces urbaini* | *Rhachomyces urbaini* | Insects | Romania | 1 | Vanderwolf et al. (2013a) |
| *Laboulbeniomycetes* | *Laboulbeniales* | *Laboulbeniaceae* | *Rhachomyces vayssierei* | *Rhachomyces vayssierei* | Insects | France, Italy, Morocco | 3 | Vanderwolf et al. (2013a) |
| *Laboulbeniomycetes* | *Laboulbeniales* | *Laboulbeniaceae* | *Rhachomyces venetianus* | *Rhachomyces venetianus* | Insects | Italy | 1 | Vanderwolf et al. (2013a) |
| *Laboulbeniomycetes* | *Laboulbeniales* | *Laboulbeniaceae* | *Rhachomyces vignae* | *Rhachomyces vignae* | Insects | Italy | 1 | Vanderwolf et al. (2013a); Zhang et al. (2021) |
| *Laboulbeniomycetes* | *Laboulbeniales* | *Laboulbeniaceae* | *Stigmatomyces oecotheae* | *Stigmatomyces oecotheae* | Insects | USA | 1 | Vanderwolf et al. (2013a) |
| *Laboulbeniomycetes* | *Laboulbeniales* | *Laboulbeniaceae* | *Symplectromyces* sp. | *Symplectromyces sp.* | Unreported | Croatia | 1 | Vanderwolf et al. (2013a) |
| *Laboulbeniomycetes* | *Laboulbeniales* | *Laboulbeniaceae* | *Symplectromyces vulgaris* | *Symplectromyces vulgaris* | Insects | France | 1 | Vanderwolf et al. (2013a) |
| *Laboulbeniomycetes* | *Laboulbeniales* | *Laboulbeniaceae* | *Troglomyces bilabiatus* | *Troglomyces bilabiatus* | Unreported | Spain | 1 | Enghoff and Santamaria (2015) |
| *Laboulbeniomycetes* | *Laboulbeniales* | *Laboulbeniaceae* | *Troglomyces manfrediae* | *Troglomyces manfredii* | Insects | Italy | 1 | Vanderwolf et al. (2013a) |
| *Laboulbeniomycetes* | *Laboulbeniales* | *Laboulbeniaceae* | *Troglomyces pusillus* | *Troglomyces pusillus* | Unreported | Spain | 1 | Enghoff and Santamaria (2015) |
| *Laboulbeniomycetes* | *Laboulbeniales* | *Laboulbeniaceae* | *Troglomyces triandrus* | *Troglomyces triandrus* | Unreported | Spain | 1 | Enghoff and Santamaria (2015) |
| *Lecanoromycetes* | *Ostropales* | *Stictidaceae* | *Cyanodermella* sp. | *Cyanodermella* sp. | Rock | Thailand | 1 | Suetrong et al. (2023) |
| *Leotiomycetes* | *Chaetomellales* | *Chaetomellaceae* | *Pilidium concavum* | *Pilidium concavum* | Unreported | China | 1 | Zhang (2019) |
| *Leotiomycetes* | *Helotiales* | *Amorphothecaceae* | *Amorphotheca resinae* | *Amorphotheca resinae* | Air, cave wall, wood | Czech Republic | 1 | Vanderwolf et al. (2013a); Zhang et al. (2021) |
| *Leotiomycetes* | *Helotiales* | *Amorphothecaceae* | *Amorphotheca resinae* | *Hormoconis resinae* | Air, cave wall, wood | Czech Republic | 1 | Vanderwolf et al. (2013a) |
| *Leotiomycetes* | *Helotiales* | *Discinellaceae* | *Varicosporium giganteum* | *Varicosporium giganteum* | Soil-sediment, water | Puerto Rico | 1 | Vanderwolf et al. (2013a) |
| *Leotiomycetes* | *Helotiales* | *Discinellaceae* | *Varicosporium* spp. | *Varicosporium* spp. | Soil-sediment | Switzerland | 1 | Vanderwolf et al. (2013a) |
| *Leotiomycetes* | *Helotiales* | *Erysiphaceae* | *Erysiphe polygoni* | *Erysiphe polygoni* | Unreported | Antarctica | 1 | Connell and Staudigel (2013) |
| *Leotiomycetes* | *Helotiales* | *Gelatinodiscaceae* | *Neobulgaria* sp. | *Neobulgaria* sp. | Bat | USA | 1 | Zhang et al. (2014) |
| *Leotiomycetes* | *Helotiales* | *Gelatinodiscaceae* | *Ombrophila alba* | *Ombrophila alba* | wood | Spain | 1 | Vanderwolf et al. (2013a) |
| *Leotiomycetes* | *Helotiales* | *Gelatinodiscaceae* | *Ombrophila janthina* | *Ombrophila janthina* | Wood | France | 1 | Vanderwolf et al. (2013a) |
| *Leotiomycetes* | *Helotiales* | *Gelatinodiscaceae* | *Ombrophila speluncarum* | *Ombrophila speluncarum* | Wood | France | 1 | Vanderwolf et al. (2013a) |
| *Leotiomycetes* | *Helotiales* | *Hamatocanthoscyphaceae* | *Constrictochalara holubovae* | *Chalara holubovae* | Unreported | China | 1 | Zhang et al. (2017) |
| *Leotiomycetes* | *Helotiales* | *Hamatocanthoscyphaceae* | *Infundichalara microchona* | *Infundichalara microchona* | Air | Spain | 1 | Vanderwolf et al. (2013a) |
| *Leotiomycetes* | *Helotiales* | *Hyaloscyphaceae* | *Dematioscypha catenata* | *Dematioscypha catenata* | Unreported | Unknow |  | Zhang et al. (2021) |
| *Leotiomycetes* | *Helotiales* | *Hyaloscyphaceae* | *Mycoarthris* sp. | *Mycoarthris* sp. | Soil-sediment | USA | 1 | Vanderwolf et al. (2013a) |
| *Leotiomycetes* | *Helotiales* | *Incertae sedis* | *Acidea extrema* | *Acidea extrema* | Slate precipitate | Germany | 1 | Burow et al. (2019); Zhang et al. (2021) |
| *Leotiomycetes* | *Helotiales* | *Incertae sedis* | *Dactylaria lanosa* | *Dactylaria lanosa* | Wood | UK | 1 | Vanderwolf et al. (2013a) |
| *Leotiomycetes* | *Helotiales* | *Incertae sedis* | *Dactylaria* sp. | *Dactylaria* sp. | Unreported | China | 1 | Zhang et al. (2021) |
| *Leotiomycetes* | *Helotiales* | *Incertae sedis* | *Rhexocercosporidium sp.* | *Rhexocercosporidium sp.* | Asymptomatic specimens of *Proteus anguinus* | Slovenia | 1 | Savković et al. (2025) |
| *Leotiomycetes* | *Helotiales* | *Incertae sedis* | *Scytalidium album* | *Scytalidium album* | wood | USA | 1 | Held et al. (2020) |
| *Leotiomycetes* | *Helotiales* | *Incertae sedis* | *Scytalidium circinatum* | *Scytalidium circinatum* | wood | USA | 1 | Held et al. (2020) |
| *Leotiomycetes* | *Helotiales* | *Incertae sedis* | *Scytalidium cuboideum* | *Scytalidium cuboideum* | Other | Czech Republic | 1 | Vanderwolf et al. (2013a) |
| *Leotiomycetes* | *Helotiales* | *Incertae sedis* | *Scytalidium lignicola* | *Scytalidium lignicola* | Air, soil-sediment, wood, earthworm casts | China, France, Slovakia | 3 | Vanderwolf et al. (2013a) |
| *Leotiomycetes* | *Helotiales* | *Incertae sedis* | *Scytalidium spp.* | *Scytalidium* spp. | Organic litter, | Canada, Thailand | 1 | Vanderwolf et al. (2015); Suetrong et al. (2023) |
| *Leotiomycetes* | *Helotiales* | *Incertae sedis* | *Tetracladium furcatum* | *Tetracladium furcatum* | Unreported | Canada | 1 | Vanderwolf et al. (2013a) |
| *Leotiomycetes* | *Helotiales* | *Incertae sedis* | *Tetracladium* sp. | *Tetracladium* sp. | Soil, environmental swab | Canada, USA | 2 | Zhang et al. (2014); Out et al. (2016) |
| *Leotiomycetes* | *Helotiales* | *Lachnaceae* | *Dasyscyphella* sp. | *Dasyscyphella* sp. | Unreported | Croatia | 1 | Vanderwolf et al. (2013a) |
| *Leotiomycetes* | *Helotiales* | *Lachnaceae* | *Lachnellula resinaria* | *Lachnellula resinaria* | Unreported | France | 1 | Vanderwolf et al. (2013a) |
| *Leotiomycetes* | *Helotiales* | *Lachnaceae* | *Lachnum brevipilosum* | *Lachnum brevipilosum* | Unreported | Unknow |  | Vanderwolf et al. (2013a); Zhang et al. (2021) |
| *Leotiomycetes* | *Helotiales* | *Lachnaceae* | *Lachnum virgineum* | *Dasyscyphus virgineus* | Unreported | France | 1 | Vanderwolf et al. (2013a) |
| *Leotiomycetes* | *Helotiales* | *Leptodontidiaceae* | *Leptodontidium elatius* var. *elatius* | *Leptodontidium elatius* var. *elatius* | Unreported | Canada | 1 | Vanderwolf et al. (2013a) |
| *Leotiomycetes* | *Helotiales* | *Leptodontidiaceae* | *Leptodontidium trabinellum* | *Leptodontidium trabinellum* | Unreported | Unknow |  | Vanderwolf et al. (2013a); Zhang et al. (2021) |
| *Leotiomycetes* | *Helotiales* | *Leptodontidiaceae* | *Leptosphaeria* spp. | *Leptosphaeria* spp. | Air, limestone | China, Spain | 2 | Vanderwolf et al. (2013a); Jiang et al. (2017) |
| *Leotiomycetes* | *Helotiales* | *Mollisiaceae* | *Diplococcium* sp. | *Diplococcium* sp. | Bats | Canada | 1 | Vanderwolf et al. (2015) |
| *Leotiomycetes* | *Helotiales* | *Mollisiaceae* | *Phialocephala* spp. | *Phialocephala* spp. | Air, live bat fur, organic litter, skin, water | Canada, France, Thailand | 3 | Vanderwolf et al. (2013a); Suetrong et al. (2023) |
| *Leotiomycetes* | *Helotiales* | *Mollisiaceae* | *Tapesia fusca* | *Tapesia fusca* | Wood | Poland | 1 | Vanderwolf et al. (2013a) |
| *Leotiomycetes* | *Helotiales* | *Myxotrichaceae* | *Myxotrichum chartarum* | *Myxotrichum chartarum* | Bat guano | Hungary | 1 | Vanderwolf et al. (2013a) |
| *Leotiomycetes* | *Helotiales* | *Myxotrichaceae* | *Myxotrichum deflexum* | *Myxotrichum deflexum* | Air, soil-sediment, earthworm casts, bat guano | Slovakia, Hungary, Czech Republic | 3 | Vanderwolf et al. (2013a) |
| *Leotiomycetes* | *Helotiales* | *Myxotrichaceae* | *Myxotrichum setosum* | *Myxotrichum setosum* | Unreported | USA | 1 | Vanderwolf et al. (2013a) |
| *Leotiomycetes* | *Helotiales* | *Myxotrichaceae* | *Oidiodendron cereale* | *Oidiodendron cereale* | Air, soil-sediment, bat guano, earthworm casts | France, Slovakia, Russia | 3 | Vanderwolf et al. (2013a) |
| *Leotiomycetes* | *Helotiales* | *Myxotrichaceae* | *Oidiodendron cereale* | *Oidiodendron nigrum* | Wood | Belgium | 1 | Vanderwolf et al. (2013a) |
| *Leotiomycetes* | *Helotiales* | *Myxotrichaceae* | *Oidiodendron flavum* | *Oidiodendron flavum* | Cave wall, soil | Austria, Israel | 2 | Vanderwolf et al. (2013a) |
| *Leotiomycetes* | *Helotiales* | *Myxotrichaceae* | *Oidiodendron griseum* | *Oidiodendron griseum* | Cave wall, , wood, bat guanosoil-sediment, | Sweden, Romania, Canada, Belgium, Czech Republic, Slovakia, USA | 7 | Vanderwolf et al. (2013a); Nováková et al. (2018); Held et al. (2020) |
| *Leotiomycetes* | *Helotiales* | *Myxotrichaceae* | *Oidiodendron majus* | *Oidiodendron citrinum* | Bat guano | Slovakia | 1 | Vanderwolf et al. (2013a) |
| *Leotiomycetes* | *Helotiales* | *Myxotrichaceae* | *Oidiodendron majus* | *Oidiodendron maius* | Air | Poland | 1 | Vanderwolf et al. (2013a) |
| *Leotiomycetes* | *Helotiales* | *Myxotrichaceae* | *Oidiodendron myxotrichoides* | *Oidiodendron myxotrichoides* | Soil | Ontario, Canada | 2 | Vanderwolf et al. (2013a) |
| *Leotiomycetes* | *Helotiales* | *Myxotrichaceae* | *Oidiodendron rhodogenum* | *Oidiodendron rhodogenum* | Wood | Norway, Belgium | 2 | Vanderwolf et al. (2013a) |
| *Leotiomycetes* | *Helotiales* | *Myxotrichaceae* | *Oidiodendron* spp. | *Oidiodendron* spp. | Air, soil-sediment, bat guano, soil | Canada, China, USA, Slovakia, France | 5 | Vanderwolf et al. (2013, 2019); Man (2017); Zhang et al. (2021) |
| *Leotiomycetes* | *Helotiales* | *Myxotrichaceae* | *Oidiodendron tenuissimum* | *Oidiodendron fuscum* | Wood | Belgium | 1 | Vanderwolf et al. (2013a) |
| *Leotiomycetes* | *Helotiales* | *Myxotrichaceae* | *Oidiodendron tenuissimum* | *Oidiodendron tenuissimum* | Wood, soil-sediment | China, UK, USA, Slovakia, Russia | 5 | Vanderwolf et al. (2013a) |
| *Leotiomycetes* | *Helotiales* | *Myxotrichaceae* | *Oidiodendron truncatum* | *Oidiodendron truncatum* | Air, soil-sediment, wood | Canada, China, Czech Republic, Russia, Slovakia, USA | 6 | Vanderwolf et al. (2013, 2015, 2019); Zhang et al. (2014); Held et al. (2020) |
| *Leotiomycetes* | *Helotiales* | *Pezizellaceae* | *Calycina citrina* | *Bisporella citrina* | Wood | France | 1 | Vanderwolf et al. (2013a) |
| *Leotiomycetes* | *Helotiales* | *Pezizellaceae* | *Chalara* spp. | *Chalara* spp. | Air, insect, wood | Belgium, China, Czech Republic | 3 | Vanderwolf et al. (2013, 2019) |
| *Leotiomycetes* | *Helotiales* | *Pezizellaceae* | *Cylindrocephalum stellatum* | *Cylindrocephalum stellatum* | Wood | Belgium | 1 | Vanderwolf et al. (2013a) |
| *Leotiomycetes* | *Helotiales* | *Pezizellaceae* | *Cylindrocladium spp.* | *Cylindrocladium spp.* | Unreported, insects | USA, Slovenia | 2 | Vanderwolf et al. (2013a) |
| *Leotiomycetes* | *Helotiales* | *Pezizellaceae* | *Minichalara microspora* | *Chalara microspora* | Air | Canada, Spain | 2 | Vanderwolf et al. (2013a); Zhang et al. (2021) |
| *Leotiomycetes* | *Helotiales* | *Ploettnerulaceae* | *Cadophora fastigiata* | *Cadophora fastigiata* | Soil, sediment | Canada, China, USA | 3 | Out et al. (2016); Pereira et al. (2022) |
| *Leotiomycetes* | *Helotiales* | *Ploettnerulaceae* | *Cadophora malorum* | *Cadophora malorum* | Isopod feces, soil and sediment | Romania, Iran | 2 | Nováková et al. (2018) |
| *Leotiomycetes* | *Helotiales* | *Ploettnerulaceae* | *Cadophora melinii* | *Cadophora melinii* | Soil-sediment | USA | 1 | Vanderwolf et al. (2013a) |
| *Leotiomycetes* | *Helotiales* | *Ploettnerulaceae* | *Cadophora* sp. | *Cadophora speluncarum* | Bat guano | Czech Republic, Slovakia, Romania | 3 | Vanderwolf et al. (2013a); Zhang et al. (2021) |
| *Leotiomycetes* | *Helotiales* | *Ploettnerulaceae* | *Cadophora sp.* | *Cadophora tropicum* | Soil, sediment | Israel, Spain | 2 | Vanderwolf et al. (2013a); Zhang et al. (2021) |
| *Leotiomycetes* | *Helotiales* | *Ploettnerulaceae* | *Cadophora spp.* | *Cadophora spp.* | Bats, cave wall | Canada, USA | 2 | Zhang et al. (2014); Vanderwolf et al. (2019) |
| *Leotiomycetes* | *Helotiales* | *Ploettnerulaceae* | *Pyrenopeziza dilutella* | *Pyrenopeziza dilutella* | Wood | France | 1 | Vanderwolf et al. (2013a) |
| *Leotiomycetes* | *Helotiales* | *Rutstroemiaceae* | Lambertella sp. | *Lambertella arawaka* | Unreported | China | 1 | Zhang (2019) |
| *Leotiomycetes* | *Helotiales* | *Sclerotiniaceae* | *Botrytis cinerea* | *Botryotinia fuckeliana* | Air, soil-sediment | China, Spain | 1 | Vanderwolf et al. (2013a); Man (2017) |
| *Leotiomycetes* | *Helotiales* | *Sclerotiniaceae* | *Botrytis cinerea* | *Botrytis cinerea* | Air, soil-sediment, wood, earthworm casts, other, cave wall | China, Italy, Belgium, Poland, Czech Republic, Slovakia, Romania, Spain, India | 9 | Vanderwolf et al. (2013a); Zhang et al. (2021) |
| *Leotiomycetes* | *Helotiales* | *Sclerotiniaceae* | *Botrytis spp.* | *Botrytis* spp. | Air, soil-sediment, water | France, Puerto Rico, Spain, Russia | 4 | Vanderwolf et al. (2013a); Leplat et al. (2018) |
| *Leotiomycetes* | *Helotiales* | *Sclerotiniaceae* | *Dumontinia tuberosa* | *Dumontinia tuberosa* | Air | Poland | 1 | Savković et al. (2025) |
| *Leotiomycetes* | *Helotiales* | *Sclerotiniaceae* | *Sclerotinia sclerotiorum* | *Sclerotinia sclerotiorum* | Air | China, Poland | 1 | Ogórek et al. (2013, 2014a, b, c); Man (2017) |
| *Leotiomycetes* | *Helotiales* | *Sclerotiniaceae* | *Sclerotinia* sp. | *Sclerotinia* sp. | Unreported | France | 1 | Vanderwolf et al. (2013a) |
| *Leotiomycetes* | *Helotiales* | *Tricladiaceae* | *Halenospora varia* | *Halenospora varia* | Bat | USA | 1 | Zhang et al. (2014) |
| *Leotiomycetes* | *Helotiales* | *Tricladiaceae* | *Helotium* spp. | *Helotium* spp. | Unreported, wood | UK, Germany | 2 | Vanderwolf et al. (2013a) |
| *Leotiomycetes* | *Helotiales* | *Tricladiaceae* | *Ombrophila alba* | *Helotium album* | Wood | Spain | 1 | Vanderwolf et al. (2013a) |
| *Leotiomycetes* | *Helotiales* | *Tricladiaceae* | *Tricladium brunneum* | *Tricladium brunneum* | Soil-sediment, water | Puerto Rico | 1 | Vanderwolf et al. (2013a) |
| *Leotiomycetes* | *Helotiales* | *Vibrisseaceae* | *Vibrissea truncorum* | *Vibrissea truncorum* | Wood | Switzerland | 1 | Vanderwolf et al. (2013a) |
| *Leotiomycetes* | *Phacidiales* | *Phacidiaceae* | [*Allantophomopsiella pseudotsugae*](http://www.speciesfungorum.org/Names/SynSpecies.asp?RecordID=809674) | [*Allantophomopsiella pseudotsugae*](http://www.speciesfungorum.org/Names/SynSpecies.asp?RecordID=809674) | Air | Poland | 1 | Pusz et al. 2017 |
| *Leotiomycetes* | *Phacidiales* | *Phacidiaceae* | *Bulgaria inquinans* | *Bulgaria inquinans* | Wood | Slovenia, Bosnia, Czzech Republic, Romania | 4 | Vanderwolf et al. (2013a) |
| *Leotiomycetes* | *Thelebolales* | *Pseudeurotiaceae* | *Geomyces auratus* | *Geomyces auratus* | Unreported | China | 1 | Zhang et al. (2021) |
| *Leotiomycetes* | *Thelebolales* | *Pseudeurotiaceae* | *Geomyces* spp. | *Geomyces* spp. | Air, soil, sediment, precipitatewing of Perimyotis subflavus, bat guano | Canada, China, USA, France, Czech Republic, Germany, Slovakia | 7 | Vanderwolf et al. (2013a); Man (2017); Burow et al. (2019); Pfendler et al. (2019); Zhang et al. (2021) |
| *Leotiomycetes* | *Thelebolales* | *Pseudeurotiaceae* | *Geomyces vinaceus* | *Geomyces vinaceus* | Soil-sediment | Italy | 1 | Vanderwolf et al. (2013a) |
| *Leotiomycetes* | *Thelebolales* | *Pseudeurotiaceae* | *Gymnostellatospora* sp. | *Gymnostellatospora* sp. | Unreported | Canada | 1 | Vanderwolf et al. (2013a) |
| *Leotiomycetes* | *Thelebolales* | *Pseudeurotiaceae* | *Leuconeurospora capsici* | *Leuconeurospora capsici* | Unreported | Canada | 1 | Vanderwolf et al. (2015, 2019); Malloch et al. (2016) |
| *Leotiomycetes* | *Thelebolales* | *Pseudeurotiaceae* | *Leuconeurospora polypaeciloides* | *Leuconeurospora polypaeciloides* | Unreported | Canada | 1 | Vanderwolf et al. (2015, 2019); Malloch et al. (2016) |
| *Leotiomycetes* | *Thelebolales* | *Pseudeurotiaceae* | *Leuconeurospora pulcherrima* | *Leuconeurospora pulcherrima* | Bat | USA | 1 | Zhang et al. (2014) |
| *Leotiomycetes* | *Thelebolales* | *Pseudeurotiaceae* | *Leuconeurospora* sp. | *Leuconeurospora* sp. | Unreported | Canada | 1 | Vanderwolf et al. (2013a) |
| *Leotiomycetes* | *Thelebolales* | *Pseudeurotiaceae* | *Pseudeurotium bakeri* | *Pseudeurotium bakeri* | Water, floor | Germany, Belgium | 1 | Burow et al. (2019) |
| *Leotiomycetes* | *Thelebolales* | *Pseudeurotiaceae* | *Pseudeurotium hygrophilum* | *Pseudeurotium hygrophilum* | Unreported | Romania | 1 | Brad et al. (2018) |
| *Leotiomycetes* | *Thelebolales* | *Pseudeurotiaceae* | *Pseudeurotium hygrophilum* | *Pseudeurotium zonatum* | Bat guano | Hungary | 1 | Vanderwolf et al. (2013a) |
| *Leotiomycetes* | *Thelebolales* | *Pseudeurotiaceae* | Pseudeurotium spp. | Pseudeurotium spp. | Soil, insect | Canada, China, USA, Belgium | 4 | Vanderwolf et al. (2013a); Out et al. (2016); |
| *Leotiomycetes* | *Thelebolales* | *Pseudeurotiaceae* | *Pseudogymnoascus destructans* | *Pseudogymnoascus destructans* | Precipitate, wing of Perimyotis subflavus, from the skin of wings, ears and muzzles, soil | Germany, China, Estonia, Czech Republic, Poland, Italy, Russia, USA | 8 | Kokurewicz et al. (2016); Burow et al. (2019); Zhang et al. (2021) |
| *Leotiomycetes* | *Thelebolales* | *Pseudeurotiaceae* | *Pseudogymnoascus hyalinus* | *Ciliciopodium hyalinum* | Water | Germany, China | 2 | Burow et al. (2019); Zhang et al. (2021) |
| *Leotiomycetes* | *Thelebolales* | *Pseudeurotiaceae* | *Pseudogymnoascus palmeri* | *Pseudogymnoascus palmeri* | Sediment | USA | 1 | Savković et al. (2025) |
| *Leotiomycetes* | *Thelebolales* | *Pseudeurotiaceae* | *Pseudogymnoascus pannorum* | *Geomyces pannorum* | Air, soil-sediment, cave wall, insects, wood, other, bat guano, earthworm casts | Belgium, Canada, China, Czech Republic, France, Hungary, Iran, Italy, Japan, Poland, Russia, Slovakia, Slovenia, Spain, UK, USA | 16 | Vanderwolf et al. (2013, 2015, 2019); Zhang et al. (2021) |
| *Leotiomycetes* | *Thelebolales* | *Pseudeurotiaceae* | *Pseudogymnoascus pannorum* | Geomyces pannorum var. pannorum | Soil-sediment | Italy | 1 | Vanderwolf et al. (2013a) |
| *Leotiomycetes* | *Thelebolales* | *Pseudeurotiaceae* | *Pseudogymnoascus pannorum* | *Pseudogymnoascus pannorum* | cawe wall, sediment, animal bone, Air | Belgium, France, Poland, Spain | 4 | Jiang et al. (2017) |
| *Leotiomycetes* | *Thelebolales* | *Pseudeurotiaceae* | *Pseudogymnoascus roseus* | *Pseudogymnoascus roseus* | Unreported | Canada | 1 | Vanderwolf et al. (2013a) |
| *Leotiomycetes* | *Thelebolales* | *Pseudeurotiaceae* | *Pseudogymnoascus* spp. | *Pseudogymnoascus* spp. | soil, precipitate, cave sediment, bat guano, sediment, wood | Germany, Canada, USA, South Korea, Czech Republic, Belgium, Bulgaria | 7 | Out et al. (2016); Burow et al. (2019); Held et al. (2020) |
| *Leotiomycetes* | *Thelebolales* | *Pseudeurotiaceae* | *Pseudogymnoascus verrucosus* | *Pseudogymnoascus verrucosus* | Cawe wall | Belgium | 1 | Savković et al. (2025) |
| *Leotiomycetes* | *Thelebolales* | *Pseudeurotiaceae* | *Pseudopithomyces chartarum* | *Pseudopithomyces chartarum* | Air | Poland | 1 | Pusz et al. (2015) |
| *Leotiomycetes* | *Thelebolales* | *Pseudeurotiaceae* | *Pseudopithomyces maydicus* | *Pseudopithomyces maydicus* | Unreported | China | 1 | Zhang et al. (2021) |
| *Leotiomycetes* | *Thelebolales* | *Thelebolaceae* | *Hyphozyma* sp. | *Hyphozyma* sp. | Bat | Canada | 1 | Vanderwolf et al. (2015, 2019) |
| *Leotiomycetes* | *Thelebolales* | *Thelebolaceae* | *Thelebolus crustaceus* | *Thelebolus crustaceus* | Unreported | Canada | 1 | Vanderwolf et al. (2013a) |
| *Leotiomycetes* | *Thelebolales* | *Thelebolaceae* | *Thelebolus ellipsoideus* | *Thelebolus ellipsoideus* | Soil | Canada, China | 2 | Out et al. (2016) |
| *Leotiomycetes* | *Thelebolales* | *Thelebolaceae* | *Thelebolus globosus* | *Thelebolus globosus* | Unreported | Canada | 1 | Vanderwolf et al. (2013a) |
| *Leotiomycetes* | *Thelebolales* | *Thelebolaceae* | *Thelebolus microsporus* | *Thelebolus microsporus* | Unreported | Romania | 1 | Brad et al. (2018) |
| *Leotiomycetes* | *Thelebolales* | *Thelebolaceae* | *Thelebolus* sp. | *Thelebolus* sp. | Unreported | Canada | 1 | Vanderwolf et al. (2013a) |
| *Microbotryomycetes* | *Sporidiobolales* | *Sporidiobolaceae* | *Sporobolomyces salmonicolor* | *Blastoderma salmonicolor* | Soil-sediment | Italy | 1 | Vanderwolf et al. (2013a) |
| *Orbiliomycetes* | *Orbiliales* | *Arthrobotryaceae* | *Dactylella* sp*.* | *Dactylella* sp*.* | Soil-sediment | India | 1 | Vanderwolf et al. (2013a) |
| *Orbiliomycetes* | *Orbiliales* | *Arthrobotryaceae* | *Orbilia oligospora* | *Arthrobotrys oligosporus* | Air, other | Poland | 1 | Vanderwolf et al. (2013a) |
| *Orbiliomycetes* | *Orbiliales* | *Arthrobotryaceae* | *Pyrenopeziza dilutella* | *Hyalinia dilutella* | Wood | France | 1 | Vanderwolf et al. (2013a) |
| *Orbiliomycetes* | *Orbiliales* | *Orbiliaceae* | *Arthrobotrys arthrobotryoides* | *Arthrobotrys arthrobotryoides* | Soil-sediment | Russia | 1 | Vanderwolf et al. (2013a) |
| *Pezizomycetes* | *Incertae sedis* | *Tarzettaceae* | *Humaria jeannelii* | *Pustularia jeannelii* | Unreported | Spain | 1 | Vanderwolf et al. (2013a) |
| *Pezizomycetes* | *Pezizales* | *Ascobolaceae* | *Ascobolus* sp. | *Ascobolus* sp. | Air, insects | Spain | 1 | Vanderwolf et al. (2013a) |
| *Pezizomycetes* | *Pezizales* | *Incertae sedis* | *Oedocephalum sp.* | *Oedocephalum sp.* | Cave wall | UK | 1 | Vanderwolf et al. (2013a) |
| *Pezizomycetes* | *Pezizales* | *Pezizaceae* | *Adelphella babingtonii* | *Adelphella babingtonii* | wood | Czech Republic | 1 | Vanderwolf et al. (2013a); Zhang et al. (2021) |
| *Pezizomycetes* | *Pezizales* | *Pezizaceae* | *Adelphella babingtonii* | *Pachyella babingtonii* | Wood | Czech Republic | 1 | Vanderwolf et al. (2013a) |
| *Pezizomycetes* | *Pezizales* | *Pezizaceae* | *Boudiera* sp. | *Boudiera* sp. | Unreported | USA | 1 | Vanderwolf et al. (2013a); Zhang et al. (2021) |
| *Pezizomycetes* | *Pezizales* | *Pezizaceae* | *Calycina citrina* | *Peziza citrina* | Wood | France | 1 | Vanderwolf et al. (2013a) |
| *Pezizomycetes* | *Pezizales* | *Pezizaceae* | *Peziza micropus* | *Peziza micropus* | Unreported | France | 1 | Vanderwolf et al. (2013a) |
| *Pezizomycetes* | *Pezizales* | *Pezizaceae* | *Peziza* spp. | *Peziza* spp. | Unreported, wood, bat guano | USA, UK, Italy | 3 | Vanderwolf et al. (2013a) |
| *Pezizomycetes* | *Pezizales* | *Pezizaceae* | Pezizaceae sp. | Pezizaceae sp. | Unreported, wood | Germany | 1 | Vanderwolf et al. (2013a) |
| *Pezizomycetes* | *Pezizales* | *Pyronemataceae* | *Humaria jeannelii* | *Humaria jeannelii* | Unreported | Spain | 1 | Vanderwolf et al. (2013a) |
| *Pezizomycetes* | *Pezizales* | *Pyronemataceae* | *Lachnea spelaea* | *Lachnea spelaea* | Unreported | Italy | 1 | Vanderwolf et al. (2013a) |
| *Pezizomycetes* | *Pezizales* | *Pyronemataceae* | *Scutellinia sp.* | *Scutellinia sp.* | Unreported | China | 1 | Zhang et al. (2017) |
| *Pezizomycetes* | *Pezizales* | *Rhizinaceae* | *Phymatotrichopsis omnivora* | *Phymatotrichopsis omnivora* | Unreported | USA | 1 | Vanderwolf et al. (2013a) |
| *Pezizomycetes* | *Pezizales* |  | Pezizales sp. | Pezizales sp. | Wood | UK | 1 | Vanderwolf et al. (2013a) |
| *Pichiomycetes* | *Saccharomycetales* | *Dipodascaceae* | *Yarrowia deformans* | *Candida deformans* | Bat internal tissue | Mexico | 1 | Vanderwolf et al. (2013a) |
| *Pichiomycetes* | *Serinales* | *Debaryomycetaceae* | *Candida parapsilosis* | *Candida parapsilosis* | Soil-sediment, air | Brazil, South Africa | 2 | Vanderwolf et al. (2013a); Cunha et al. (2020) |
| *Saccharomycetes* | *Saccharomycetales* | *Debaryomycetaceae* | *Candida albicans* | *Candida albicans* | Air, rock, insect | Poland, USA | 2 | Ogórek et al. (2013, 2016c, d); Vanderwolf et al. (2013a); Kokurewicz et al. (2016) |
| *Saccharomycetes* | *Saccharomycetales* | *Debaryomycetaceae* | *Candida albicans* | *Candida albicans* var. *albicans* | Insects, other | USA | 1 | Vanderwolf et al. (2013a) |
| *Saccharomycetes* | *Saccharomycetales* | *Debaryomycetaceae* | *Candida norvegica* | *Candida norvegica* | Soil-sediment | Italy | 1 | Vanderwolf et al. (2013a) |
| *Saccharomycetes* | *Saccharomycetales* | *Debaryomycetaceae* | *Candida palmioleophila* | *Candida palmioleophila* | Bat guano, air | Italy, Japan, South Africa | 3 | Vanderwolf et al. (2013a) |
| *Saccharomycetes* | *Saccharomycetales* | *Debaryomycetaceae* | *Candida pseudoglaebosa* | *Candida pseudoglaebosa* | Bats | USA | 1 | Zhang et al. (2014) |
| *Saccharomycetes* | *Saccharomycetales* | *Debaryomycetaceae* | *Candida saitoana* | *Candida saitoana* | Soil-sediment | Italy | 1 | Vanderwolf et al. (2013a) |
| *Saccharomycetes* | *Saccharomycetales* | *Debaryomycetaceae* | *Candida* spp. | *Candida* spp. | Air, cave wall, bat guano, , water, soil-sediment, slate precipitate, unreported | Brazil, Canada, China, France, Germany, Italy, South Africa, USA | 8 | Vanderwolf et al. (2013a); Burow et al. (2019); Alves et al. (2022) |
| *Saccharomycetes* | *Saccharomycetales* | *Debaryomycetaceae* | *Candida tropicalis* | *Candida tropicalis* | Soil, sediment | Italy | 1 | Vanderwolf et al. (2013a) |
| *Saccharomycetes* | *Saccharomycetales* | *Debaryomycetaceae* | *Candida tropicalis* | *Candida tropicalis* var. *tropicalis* | Soil-sediment | Italy | 1 | Vanderwolf et al. (2013a) |
| *Saccharomycetes* | *Saccharomycetales* | *Debaryomycetaceae* | *Candida viswanathii* | *Candida viswanathii* | Air | South Africa | 1 | Vanderwolf et al. (2013a) |
| *Saccharomycetes* | *Saccharomycetales* | *Debaryomycetaceae* | *Candida zeylanoides* | *Candida zeylanoides* | Soil, sediment, water | China | 1 | Vanderwolf et al. (2013a) |
| *Saccharomycetes* | *Saccharomycetales* | *Debaryomycetaceae* | *Candida zeylanoides* | *Candida zeylanoides* var. *zeylanoides* | Soil-sediment, water | China | 1 | Vanderwolf et al. (2013a) |
| *Saccharomycetes* | *Saccharomycetales* | *Debaryomycetaceae* | *Clavispora lusitaniae* | *Candida lusitaniae* | Bat guano | Japan | 1 | Vanderwolf et al. (2013a) |
| *Saccharomycetes* | *Saccharomycetales* | *Debaryomycetaceae* | *Debaryomycetaceae* sp. | *Debaryomycetaceae* sp. | Bat guano | Bulgaria | 1 | Savković et al. (2025) |
| *Saccharomycetes* | *Saccharomycetales* | *Debaryomycetaceae* | *Diutina catenulata* | *Candida catenulata* | Bat guano, air | Mexico, South Africa | 2 | Vanderwolf et al. (2013a) |
| *Saccharomycetes* | *Saccharomycetales* | *Debaryomycetaceae* | *Meyerozyma caribbica* | Meyerozyma cf. caribbica | Bat | Brazil | 1 | Cunha et al. (2020) |
| *Saccharomycetes* | *Saccharomycetales* | *Debaryomycetaceae* | *Meyerozyma guilliermondii* | *Candida guilliermondii* | Bat guano | Mexico, France | 2 | Vanderwolf et al. (2013a) |
| *Saccharomycetes* | *Saccharomycetales* | *Debaryomycetaceae* | *Nakaseomyces glabratus* | *Candida glabrata* | Bat guano | USA, France | 2 | Vanderwolf et al. (2013a) |
| *Saccharomycetes* | *Saccharomycetales* | *Dipodascaceae* | *Dipodascus fermentans* | *Dipodascus fermentans* | Unreported | Unknow |  | Zhang et al. (2021) |
| *Saccharomycetes* | *Saccharomycetales* | *Dipodascaceae* | *Dipodascus fermentans* | *Geotrichum fermentans* | Air | Czech Republic | 1 | Vanderwolf et al. (2013a) |
| *Saccharomycetes* | *Saccharomycetales* | *Dipodascaceae* | *Galactomyces* sp. | *Galactomyces* sp. | Soil-sediment, Organic litter, | Thailand | 1 | Suetrong et al. (2023) |
| *Saccharomycetes* | *Saccharomycetales* | *Dipodascaceae* | *Geotrichum candidum* | *Galactomyces candidus* | Sediment | Romania | 1 | Nováková et al. (2018) |
| *Saccharomycetes* | *Saccharomycetales* | *Dipodascaceae* | *Geotrichum candidum* | *Geotrichum candidum* | Air, insects, soil-sediment, bat guano, wood, bat guano, rock surface, soil | China, India, Italy, Puerto Rico, Romania, Russia, Slovakia, Slovenia, USA | 9 | Vanderwolf et al. (2013a) |
| *Saccharomycetes* | *Saccharomycetales* | *Dipodascaceae* | *Geotrichum galactomycetum* | *Dipodascus geotrichum* | Unreported | China | 1 | Zhang et al. (2017); Zhang et al. (2021) |
| *Saccharomycetes* | *Saccharomycetales* | *Dipodascaceae* | *Geotrichum galactomycetum* | *Galactomyces geotrichum* | Unreported | Spain | 1 | Vanderwolf et al. (2013a) |
| *Saccharomycetes* | *Saccharomycetales* | *Dipodascaceae* | *Geotrichum* spp. | *Geotrichum* spp. | Air, soil-sediment, bat guano, insects | Brazil, France, Puerto Rico, Slovenia, Venezuela | 5 | Taylor et al. (2013, 2014); Vanderwolf et al. (2013a) |
| *Saccharomycetes* | *Saccharomycetales* | *Dipodascaceae* | *Yarrowia bubula* | *Yarrowia bubula* | Water | Germany | 1 | Burow et al. (2019) |
| *Saccharomycetes* | *Saccharomycetales* | *Incertae sedis* | *Diutina catenulata* | *Diutina catenulata* | Air, bat guano, leaf litter | Mexico, South Africa | 2 | Vanderwolf et al. (2013a) |
| *Saccharomycetes* | *Saccharomycetales* | *Incertae sedis* | *Diutina rugosa* | *Candida rugosa* var. *rugosa* | Air | South Africa | 7 | Vanderwolf et al. (2013a) |
| *Saccharomycetes* | *Saccharomycetales* | *Incertae sedis* | *Diutina rugosa* | *Diutina rugosa* | Air | South Africa | 1 | Vanderwolf et al. (2013a) |
| *Saccharomycetes* | *Saccharomycetales* | *Incertae sedis* | *Hyphopichia burtonii* | *Hyphopichia burtonii* | Soil-sediment | Bahamas | 1 | Vanderwolf et al. (2013a) |
| *Saccharomycetes* | *Saccharomycetales* | *Incertae sedis* | *Nakazawaea holstii* | *Nakazawaea holstii* | Air | Slovakia | 1 | Savković et al. (2025) |
| *Saccharomycetes* | *Saccharomycetales* | *Metschnikowiaceae* | *Clavispora lusitaniae* | *Clavispora lusitaniae* | Unreported | Antarctica | 1 | Connell and Staudigel (2013) |
| *Saccharomycetes* | *Saccharomycetales* | *Metschnikowiaceae* | *Metschnikowia pulcherrima* | *Metschnikowia pulcherrima* | Bat guano | Italy | 1 | Vanderwolf et al. (2013a) |
| *Saccharomycetes* | *Saccharomycetales* | *Saccharomycetaceae* | *Debaryomyces hansenii* | *Debaryomyces hansenii* | Cave wall, soil, sediment, bat guano, symptomatic specimens of Proteus anguinus | Venezuela, Italy, Japan, USA, Slovenia | 5 | Vanderwolf et al. (2013a) |
| *Saccharomycetes* | *Saccharomycetales* | *Saccharomycetaceae* | *Debaryomyces hansenii* | *Debaryomyces subglobosus* | Bat guano, bat internal tissue | Mexico | 1 | Vanderwolf et al. (2013a) |
| *Saccharomycetes* | *Saccharomycetales* | *Saccharomycetaceae* | *Debaryomyces maramus* | *Debaryomyces maramus* | Sediment | USA | 1 | Zhang et al. (2014) |
| *Saccharomycetes* | *Saccharomycetales* | *Saccharomycetaceae* | *Debaryomyces nepalensis* | *Debaryomyces nepalensis* | Air | South Africa | 1 | Vanderwolf et al. (2013a) |
| *Saccharomycetes* | *Saccharomycetales* | *Saccharomycetaceae* | *Debaryomyces prosopidis* | *Debaryomyces prosopidis* | Cave wall | Italy | 1 | Vanderwolf et al. (2013a) |
| *Saccharomycetes* | *Saccharomycetales* | *Saccharomycetaceae* | *Debaryomyces psychrosporus* | *Debaryomyces psychrosporus* | Cave wall | Venezuela | 1 | Vanderwolf et al. (2013a) |
| *Saccharomycetes* | *Saccharomycetales* | *Saccharomycetaceae* | *Debaryomyces singareniensis* | *Debaryomyces singareniensis* | Soil-sediment | India | 1 | Vanderwolf et al. (2013a) |
| *Saccharomycetes* | *Saccharomycetales* | *Saccharomycetaceae* | *Debaryomyces* spp. | *Debaryomyces* spp. | Air, soil, sediment, bat guano | France, USA, Italy | 3 | Vanderwolf et al. (2013a); , Mitova et al. (2017) |
| *Saccharomycetes* | *Saccharomycetales* | *Saccharomycetaceae* | *Hanseniaspora* sp. | *Hanseniaspora* sp. | Bat guano | Japan | 1 | Vanderwolf et al. (2013a) |
| *Saccharomycetes* | *Saccharomycetales* | *Saccharomycetaceae* | *Lachancea kluyveri* | *Lachancea kluyveri* | Cave wall, bat guano | Italy, Japan | 2 | Vanderwolf et al. (2013a) |
| *Saccharomycetes* | *Saccharomycetales* | *Saccharomycetaceae* | *Lachancea thermotolerans* | *Lachancea thermotolerans* | Unreported | Italy | 1 | Vanderwolf et al. (2013a) |
| *Saccharomycetes* | *Saccharomycetales* | *Saccharomycetaceae* | *Pichia fermentans* | *Candida fimetaria* var. *fimetaria* | Bat guano | France | 1 | Vanderwolf et al. (2013a) |
| *Saccharomycetes* | *Saccharomycetales* | *Saccharomycetaceae* | *Saccharomyces bayanus* | *Saccharomyces bayanus* | Cave wall | Italy | 1 | Vanderwolf et al. (2013a) |
| *Saccharomycetes* | *Saccharomycetales* | *Saccharomycetaceae* | *Saccharomyces cerevisiae* | *Saccharomyces cerevisiae* | Air, soil-sediment, cave wall, bat guano | Spain, France, Italy, Japan | 4 | Vanderwolf et al. (2013a) |
| *Saccharomycetes* | *Saccharomycetales* | *Saccharomycetaceae* | *Saccharomyces cerevisiae* | *Saccharomyces italicus* | Soil-sediment | Italy | 1 | Vanderwolf et al. (2013a) |
| *Saccharomycetes* | *Saccharomycetales* | *Saccharomycetaceae* | *Saccharomyces cerevisiae* | *Saccharomyces oviformis* | Unreported | Italy | 1 | Vanderwolf et al. (2013a) |
| *Saccharomycetes* | *Saccharomycetales* | *Saccharomycetaceae* | Saccharomyces cerevisiae var. cerevisiae | Saccharomyces cerevisiae var. cerevisiae | Soil-sediment, unreported, cave wall | Italy | 1 | Vanderwolf et al. (2013a) |
| *Saccharomycetes* | *Saccharomycetales* | *Saccharomycetaceae* | *Saccharomyces paradoxus* | *Saccharomyces paradoxus* | Bat guano | Italy | 1 | Vanderwolf et al. (2013a) |
| *Saccharomycetes* | *Saccharomycetales* | *Saccharomycetaceae* | *Saccharomyces pastorianus* | *Saccharomyces carlsbergensis* | Cave wall | Italy | 1 | Vanderwolf et al. (2013a) |
| *Saccharomycetes* | *Saccharomycetales* | *Saccharomycetaceae* | *Saccharomyces sensu lato* | *Saccharomyces sensu lato* | Bat guano | Italy | 1 | Vanderwolf et al. (2013a) |
| *Saccharomycetes* | *Saccharomycetales* | *Saccharomycetaceae* | *Torulaspora delbrueckii* | *Torulaspora delbrueckii* | Unreported | Bulgaria | 1 | Mitova et al. (2017) |
| *Saccharomycetes* | *Saccharomycetales* | *Saccharomycetaceae* | *Torulaspora* sp. | *Torulaspora* sp. | Sediment | Bulgaria | 1 | Savković et al. (2025) |
| *Saccharomycetes* | *Saccharomycetales* | *Saccharomycetaceae* | *Zygosaccharomyces microellipsoides* | *Torulaspora microellipsoides* | Cave wall, bat guano | Italy | 1 | Vanderwolf et al. (2013a) |
| *Saccharomycetes* | *Saccharomycetales* | *Saccharomycetaceae* | *Zygosaccharomyces microellipsoides* | *Zygosaccharomyces microellipsoides* | Cave wall, bat guano | Italy | 1 | Vanderwolf et al. (2013a) |
| *Saccharomycetes* | *Saccharomycetales* | *Saccharomycodaceae* | *Hanseniaspora osmophila* | *Hanseniaspora osmophila* | Unreported | Unknow |  | Zhang et al. (2021) |
| *Saccharomycetes* | *Saccharomycetales* | *Saccharomycodaceae* | *Hanseniaspora osmophila* | *Kloeckera corticis* | Unreported | Italy | 1 | Vanderwolf et al. (2013a) |
| *Saccharomycetes* | *Saccharomycetales* | *Saccharomycopsidaceae* | *Saccharomycopsis fibuligera* | *Saccharomycopsis fibuligera* | Unreported | China | 1 | Zhang et al. (2021) |
| *Saccharomycetes* | *Saccharomycetales* | *Trichomonascaceae* | *Blastobotrys chiropterorum* | *Blastobotrys chiropterorum* | Bat guano, insects, bat internal tissue | Columbia | 1 | Vanderwolf et al. (2013a) |
| *Saccharomycetes* | *Saccharomycetales* | *Trichomonascaceae* | *Blastobotrys malaysiensis* | *Blastobotrys malaysiensis* | bat gunao | Brazil, China | 2 | Zhang et al. (2021) |
| *Saccharomycetes* | *Saccharomycetales* | *Trichomonascaceae* | *Blastobotrys persicus* | *Blastobotrys persicus* | Yeast species isolated from cave soil | Iran | 1 | Nouri et al. (2017) |
| *Saccharomycetes* | *Saccharomycetales* | *Trichomonascaceae* | *Blastobotrys* sp. | *Blastobotrys* sp. | Unreported | China | 1 | Zhang et al. (2021) |
| *Saccharomycetes* | *Saccharomycetales* | *Wickerhamomycetaceae* | *Barnettozyma californica* | *Barnettozyma californica* | Bat guano, rock surface | China, Italy, Japan | 3 | Vanderwolf et al. (2013a); Zhang et al. (2021) |
| *Saccharomycetes* | *Saccharomycetales* | *Wickerhamomycetaceae* | *Wickerhamomyces anomalus* | *Wickerhamomyces anomalus* | Soil-sediment, cave wall, discarded tissue, rock sufaces | China, Italy | 2 | Vanderwolf et al. (2013a) |
| *Saccharomycetes* | *Saccharomycetales* | *Wickerhamomycetaceae* | *Wickerhamomyces subpelliculosus* | *Wickerhamomyces subpelliculosus* | Cave wall | Italy | 1 | Vanderwolf et al. (2013a) |
| *Saccharomycetes* | *Saccharomycetales* |  | Saccharomycetales sp. | Saccharomycetales sp. | Rock | Thailand | 1 | Suetrong et al. (2023) |
| *Sordariomycetes* | *Amphisphaeriales* | *Amphisphaeriaceae* | *Monographella* sp. | *Monographella* sp. | Unreported | China | 1 | Zhang (2019) |
| *Sordariomycetes* | *Amphisphaeriales* | *Apiosporaceae* | *Apiospora arundinis* | *Apiospora arundinis* | Air, sediment, rock surface | China, Spain, Bulgaria | 3 | Vanderwolf et al. (2013a); Man et al. (2015); Jiang et al. (2017a, b); Mitova et al. (2017); Zhang et al. (2017); Savković et al. (2025) |
| *Sordariomycetes* | *Amphisphaeriales* | *Apiosporaceae* | *Apiospora arundinis* | *Arthrinium arundinis* | Air, bat guano, earthworm casts | China, Slovakia | 2 | Vanderwolf et al. (2013a); Jiang et al. (2017); Man (2017); Wang et al. (2018) |
| *Sordariomycetes* | *Amphisphaeriales* | *Apiosporaceae* | *Apiospora guizhouensis* | *Apiospora guizhouensis* | Air | China | 1 | Wang et al. (2018) |
| *Sordariomycetes* | *Amphisphaeriales* | *Apiosporaceae* | *Apiospora guizhouensis* | *Arthrinium guizhouense* | Air | China | 1 | Wang et al. (2018) |
| *Sordariomycetes* | *Amphisphaeriales* | *Apiosporaceae* | *Apiospora kogelbergensis* | *Apiospora kogelbergensis* | Air | Slovakia | 1 | Ogórek et al. (2018) |
| *Sordariomycetes* | *Amphisphaeriales* | *Apiosporaceae* | *Apiospora malaysiana* | *Arthrinium malaysianum* | Organic litter | China | 1 | Zhang et al. (2017) |
| *Sordariomycetes* | *Amphisphaeriales* | *Apiosporaceae* | *Apiospora marii* | *Arthrinium marii* | Air, rock surface | China | 1 | Zhang et al. (2017) |
| *Sordariomycetes* | *Amphisphaeriales* | *Apiosporaceae* | *Apiospora sacchari* | *Arthrinium sacchari* | Air | China | 1 | Zhang et al. (2017) |
| *Sordariomycetes* | *Amphisphaeriales* | *Apiosporaceae* | *Apiospora sphaerosperma* | *Arthrinium phaeospermum* | Air, cave soil, cave wall, sediment, soil, earthworm casts | China, Czech Republic, Italy, Serbia, Slovakia, USA | 6 | Vanderwolf et al. (2013a); Zhang et al. (2017) |
| *Sordariomycetes* | *Amphisphaeriales* | *Bartaliniaceae* | *Bartalinia robillardoides* | *Bartalinia robillardoides* | Unreported | China | 1 | Zhang et al. (2021) |
| *Sordariomycetes* | *Amphisphaeriales* | *Bartaliniaceae* | *Truncatella angustata* | *Truncatella angustata* | Soil-sediment, slate precipitate | Germany, Slovakia | 2 | Vanderwolf et al. (2013a); Burow et al. (2019) |
| *Sordariomycetes* | *Amphisphaeriales* | *Bartaliniaceae* | *Truncatella* spp. | *Truncatella* spp. | Slate precipitate | Germany | 1 | Burow et al. (2019) |
| *Sordariomycetes* | *Amphisphaeriales* | *Discosiaceae* | *Discosia pseudoartocreas* | *Discosia pseudoartocreas* | Unreported | China | 1 | Zhang et al. (2021) |
| *Sordariomycetes* | *Amphisphaeriales* | *Discosiaceae* | *Discosia* sp. | *Discosia* sp. | Air, rock | Slovakia | 1 | Ogórek et al. ( 2018a); Ogórek (2018a, b) |
| *Sordariomycetes* | *Amphisphaeriales* | *Pestalotiopsidaceae* | *Pestalotia cocculi* | *Pestalotia cocculi* | Unreported | China | 1 | Zhang et al. (2021) |
| *Sordariomycetes* | *Amphisphaeriales* | *Pestalotiopsidaceae* | *Pestalotia* sp*.* | *Pestalotia* sp*.* | Air, insects, soil-sediment | Puerto Rico, USA, Australia | 3 | Vanderwolf et al. (2013a) |
| *Sordariomycetes* | *Amphisphaeriales* | *Sporocadaceae* | *Discostroma corticola* | *Discostroma corticola* | Unreported | China | 1 | Zhang et al. (2021) |
| *Sordariomycetes* | *Amphisphaeriales* | *Sporocadaceae* | *Discostroma fuscellum* | *Leptosphaeria fuscella* | Unreported | China | 1 | Zhang (2019) |
| *Sordariomycetes* | *Amphisphaeriales* | *Sporocadaceae* | *Neopestalotiopsis cavernicola* | *Neopestalotiopsis cavernicola* | Cave substrata, rock | China | 1 | Liu et al. (2021) |
| *Sordariomycetes* | *Amphisphaeriales* | *Sporocadaceae* | *Pestalotiopsis cocculi* | *Pestalotiopsis cocculi* | Unreported | China | 1 | Zhang (2019) |
| *Sordariomycetes* | *Amphisphaeriales* | *Sporocadaceae* | *Pestalotiopsis guepinii* | *Pestalotiopsis guepinii* | Unreported | China | 1 | Zhang et al. (2017) |
| *Sordariomycetes* | *Amphisphaeriales* | *Sporocadaceae* | *Pestalotiopsis hainanensis* | *Pestalotiopsis hainanensis* | Unreported | China | 1 | Zhang (2019) |
| *Sordariomycetes* | *Amphisphaeriales* | *Sporocadaceae* | *Pestalotiopsis maculiformans* | *Pestalotiopsis maculiformans* | Unreported | Canada | 1 | Vanderwolf et al. (2013a) |
| *Sordariomycetes* | *Amphisphaeriales* | *Sporocadaceae* | *Pestalotiopsis mangiferae* | *Pestalotiopsis mangiferae* | Unreported | China | 1 | Zhang et al. (2021) |
| *Sordariomycetes* | *Amphisphaeriales* | *Sporocadaceae* | *Pestalotiopsis microspora* | *Pestalotiopsis microspora* | Unreported | China | 1 | Zhang et al. (2017) |
| *Sordariomycetes* | *Amphisphaeriales* | *Sporocadaceae* | *Pestalotiopsis palmarum* | *Pestalotiopsis palmarum* | Soil-sediment | Bahamas | 1 | Vanderwolf et al. (2013a) |
| *Sordariomycetes* | *Amphisphaeriales* | *Sporocadaceae* | *Pestalotiopsis* sp*.* | *Pestalotiopsis* sp. | Soil-sediment | Canada, Japan | 2 | Vanderwolf et al. (2013a) |
| *Sordariomycetes* | *Amphisphaeriales* | *Sporocadaceae* | *Pestalotiopsis trachicarpicola* | *Pestalotiopsis trachicarpicola* | Cave substrata | China | 1 | Hyde et al. (2024) |
| *Sordariomycetes* | *Amphisphaeriales* | *Sporocadaceae* | *Pestalotiopsis uvicola* | *Pestalotiopsis uvicola* | Unreported | China | 1 | Zhang (2019) |
| *Sordariomycetes* | *Amphisphaeriales* | *Sporocadaceae* | *Pestalotiopsis vismiae* | *Pestalotiopsis vismiae* | Unreported | China | 1 | Zhang et al. (2021) |
| *Sordariomycetes* | *Amphisphaeriales* | *Sporocadaceae* | *Seimatosporium* sp. | *Seimatosporium* sp. | Unreported | China | 1 | Zhang et al. (2021) |
| *Sordariomycetes* | *Calosphaeriales* | *Calosphaeriaceae* | *Jattaea reniformis* | *Jattaea reniformis* | Unreported | China | 1 | Zhang et al. (2021) |
| *Sordariomycetes* | *Cephalothecales* | *Cephalothecaceae* | Cephalotheca sp. | Cephalotheca sp. | Unreported | China | 1 | Pereira et al. (2022) |
| *Sordariomycetes* | *Cephalothecales* | *Cephalothecaceae* | *Phialemonium dimorphosporum* | *Phialemonium dimorphosporum* | Unreported | China | 1 | Zhang et al. (2021) |
| *Sordariomycetes* | *Cephalothecales* | *Cephalothecaceae* | *Phialemonium inflatum* | *Paecilomyces inflatus* | Unreported | Canada | 1 | Vanderwolf et al. (2013a) |
| *Sordariomycetes* | *Cephalothecales* | *Cephalothecaceae* | *Phialemonium inflatum* | *Phialemonium inflatum* | Unreported | Unknow |  | Vanderwolf et al. (2013a); Zhang et al. (2021) |
| *Sordariomycetes* | *Cephalothecales* | *Cephalothecaceae* | *Phialemonium* spp. | *Phialemonium* spp. | Soil | China | 1 | Zhang et al. (2017) |
| *Sordariomycetes* | *Chaetosphaeriales* | *Chaetosphaeriaceae* | *Chaetosphaeria inaequalis* | *Chaetosphaeria inaequalis* | Wood | Switzerland | 1 | Vanderwolf et al. (2013a) |
| *Sordariomycetes* | *Chaetosphaeriales* | *Chaetosphaeriaceae* | *Chaetosphaeria vermicularioides* | *Chloridium virescens* | Earthworm casts | Slovakia | 1 | Vanderwolf et al. (2013a); Zhang et al. (2021) |
| *Sordariomycetes* | *Chaetosphaeriales* | *Chaetosphaeriaceae* | *Chaetosphaeria vermicularioides* | *Chloridium virescens* var. *caudigerum* | Earthworm casts | Slovakia | 1 | Vanderwolf et al. (2013a) |
| *Sordariomycetes* | *Chaetosphaeriales* | *Chaetosphaeriaceae* | *Chloridium humicola* | *Phialocephala humicola* | Unreported | China | 1 | Zhang et al. (2021) |
| *Sordariomycetes* | *Chaetosphaeriales* | *Chaetosphaeriaceae* | *Chloridium minus* | *Chloridium minus* | Wood | Belgium | 1 | Vanderwolf et al. (2013a) |
| *Sordariomycetes* | *Chaetosphaeriales* | *Chaetosphaeriaceae* | *Chloridium* spp. | *Chloridium* spp. | Soil, sediment | China, Slovakia | 2 | Zhang et al. (2017) |
| *Sordariomycetes* | *Chaetosphaeriales* | *Chaetosphaeriaceae* | *Menispora cobaltina* | *Menispora cobaltina* | Soil-sediment | Russia | 1 | Vanderwolf et al. (2013a) |
| *Sordariomycetes* | *Coniochaetales* | *Coniochaetaceae* | *Coniochaeta hoffmannii* | *Coniochaeta hoffmannii* | Unreported | China | 1 | Zhang et al. (2021) |
| *Sordariomycetes* | *Coniochaetales* | *Coniochaetaceae* | *Coniochaeta mutabilis* | *Coniochaeta mutabilis* | Unreported | China | 1 | Zhang et al. (2021) |
| *Sordariomycetes* | *Coniochaetales* | *Coniochaetaceae* | *Coniochaeta* spp. | *Coniochaeta* spp. | Air, rock sufaces, unreported | China | 1 | Zhang et al. (2021) |
| *Sordariomycetes* | *Coniochaetales* | *Coniochaetaceae* | *Lecythophora* sp. | *Lecythophora* sp. | Unreported | Canada | 1 | Vanderwolf et al. (2019) |
| *Sordariomycetes* | *Diaporthales* | *Gnomoniaceae* | *Didymella macrostoma* | *Phoma macrostoma* | Air | Spain | 1 | Vanderwolf et al. (2013a) |
| *Sordariomycetes* | *Diaporthales* | *Gnomoniaceae* | *Diplodina* sp. | *Diplodina* sp. | Air | Spain | 1 | Vanderwolf et al. (2013a) |
| *Sordariomycetes* | *Diaporthales* | *Gnomoniaceae* | *Gnomoniopsis* sp. | *Gnomoniopsis* sp. | Unreported | China | 1 | Zhang et al. (2021) |
| *Sordariomycetes* | *Diaporthales* | *Gnomoniaceae* | *Juxtiphoma eupyrena* | *Phoma eupyrena* | Air, soil-sediment, earthworm casts, cave wall | Czech Republic, Israel, Slovakia | 3 | Vanderwolf et al. (2013a) |
| *Sordariomycetes* | *Diaporthales* | *Gnomoniaceae* | *Leptosphaeria maculans* | *Phoma lingam* | Air | Slovakia | 1 | Vanderwolf et al. (2013a) |
| *Sordariomycetes* | *Diaporthales* | *Gnomoniaceae* | *Ophiognomonia ischnostyla* | *Ophiognomonia ischnostyla* | Unreported | China | 1 | Zhang (2019) |
| *Sordariomycetes* | *Diaporthales* | *Gnomoniaceae* | *Phoma* spp. | *Phoma* spp. | Air, soil-sediment, insects, earthworm casts | China, Puerto Rico, USA, Belgium, Poland, Slovenia, Czech Republic, Slovakia, Korea, Australia, France, Spain, Thailand | 13 | Vanderwolf et al. (2013a, 2015); Kokurewicz et al. (2016); Man (2017); Zhang et al. (2021); Suetrong et al. (2023) |
| *Sordariomycetes* | *Diaporthales* | *Gnomoniaceae* | *Plagiostoma pulchellum* | *Plagiostoma pulchellum* | Unreported | China | 1 | Zhang (2019) |
| *Sordariomycetes* | *Diaporthales* | *Gnomoniaceae* | *Plagiostoma* sp. | *Plagiostoma* sp. | Unreported | China | 1 | Zhang (2019) |
| *Sordariomycetes* | *Diaporthales* | *Gnomoniaceae* | *Radulum* sp. | *Radulum* sp. | Unreported | USA | 1 | Vanderwolf et al. (2013a) |
| *Sordariomycetes* | *Diaporthales* | *Melanconidaceae* | *Melanconium* sp. | *Melanconium* sp. | Unreported | China | 1 | Zhang et al. (2021) |
| *Sordariomycetes* | *Diaporthales* |  | Diaporthales sp. | Diaporthales sp. | Unreported | Thailand | 1 | Suetrong et al. (2023) |
| *Sordariomycetes* | *Glomerellales* | *Glomerellaceae* | *Colletotrichum acutatum* | *Colletotrichum acutatum* | Insects | USA | 1 | Vanderwolf et al. (2013a) |
| *Sordariomycetes* | *Glomerellales* | *Glomerellaceae* | *Colletotrichum coccodes* | *Colletotrichum coccodes* | Air | Spain | 1 | Savković et al. (2025) |
| *Sordariomycetes* | *Glomerellales* | *Glomerellaceae* | *Colletotrichum fioriniae* | *Colletotrichum fioriniae* | Unreported | China | 1 | Zhang et al. (2021) |
| *Sordariomycetes* | *Glomerellales* | *Glomerellaceae* | *Colletotrichum gloeosporioides* | *Colletotrichum gloeosporioides* | Unreported | China | 1 | Zhang et al. (2017) |
| *Sordariomycetes* | *Glomerellales* | *Glomerellaceae* | *Colletotrichum karsti* | *Colletotrichum karsti* | Unreported | China | 1 | Zhang et al. (2017) |
| *Sordariomycetes* | *Glomerellales* | *Glomerellaceae* | *Colletotrichum pisi* | *Colletotrichum pisi* | Unreported | China | 1 | Zhang et al. (2021) |
| *Sordariomycetes* | *Glomerellales* | *Glomerellaceae* | Colletotrichum spp. | Colletotrichum spp. | Cave wall, limestone, unreported | China, India | 2 | Vanderwolf et al. (2013a); Jiang et al. (2017) |
| *Sordariomycetes* | *Glomerellales* | *Plectosphaerellaceae* | *Brunneochlamydosporium nepalense* | *Brunneochlamydosporium nepalense* | Air, cave wall, soil, sediment | China, France | 2 | Vanderwolf et al.2013, Martin-Sanchez et al.2014, Zhang et al.2017,Zhang et al. (2021) |
| *Sordariomycetes* | *Glomerellales* | *Plectosphaerellaceae* | *Brunneomyces brunnescens* | *Brunneomyces brunnescens* | Unreported | China | 1 | Pereira et al. (2022) |
| *Sordariomycetes* | *Glomerellales* | *Plectosphaerellaceae* | *Gibellulopsis nigrescens* | *Gibellulopsis nigrescens* | Air | China, Czech Republic | 2 | Vanderwolf et al. (2013a) |
| *Sordariomycetes* | *Glomerellales* | *Plectosphaerellaceae* | *Gibellulopsis* sp. | *Gibellulopsis* sp. | Water | China | 1 | Jiang et al. (2017) |
| *Sordariomycetes* | *Glomerellales* | *Plectosphaerellaceae* | *Lectera colletotrichoides* | *Lectera colletotrichoides* | Unreported | Antarctica | 1 | Connell and Staudigel (2013) |
| *Sordariomycetes* | *Glomerellales* | *Plectosphaerellaceae* | *Leptobacillium leptobactrum* | *Verticillium leptobactrum* | Air, soil-sediment, cave wall | France, Italy, Czech Republic | 3 | Vanderwolf et al. (2013a) |
| *Sordariomycetes* | *Glomerellales* | *Plectosphaerellaceae* | *Plectosphaerella cucumerina* | *Plectosphaerella cucumerina* | Air, cave wall, limestone, sediment, soil | China, France, Malaysia | 3 | Vanderwolf et al. (2013a); Jiang et al. (2017); Wasti et al. (2021) |
| *Sordariomycetes* | *Glomerellales* | *Plectosphaerellaceae* | *Plectosphaerella cucumerina* | *Plectosporium tabacinum* | Soil-sediment | Japan | 1 | Vanderwolf et al. (2013a) |
| *Sordariomycetes* | *Glomerellales* | *Plectosphaerellaceae* | *Plectosphaerella niemeijerarum* | *Plectosphaerella niemeijerarum* | Unreported | China | 1 | Zhang (2019) |
| *Sordariomycetes* | *Glomerellales* | *Plectosphaerellaceae* | *Plectosphaerella oligotrophica* | *Plectosphaerella oligotrophica* | Limestone | China | 1 | Jiang et al. (2017) |
| *Sordariomycetes* | *Glomerellales* | *Plectosphaerellaceae* | *Plectosphaerella* sp. | *Plectosphaerella* sp. | Unreported | China | 1 | Zhang et al. (2017) |
| *Sordariomycetes* | *Glomerellales* | *Plectosphaerellaceae* | Plectosphaerellaceae sp. | Plectosphaerellaceae sp. | Mn-oxide-rich cave sediments | USA | 1 | Savković et al. (2025) |
| *Sordariomycetes* | *Glomerellales* | *Plectosphaerellaceae* | *Verticillium alboatrum* | *Verticillium alboatrum* | Wood | China, Romania | 2 | Vanderwolf et al. (2013a) |
| *Sordariomycetes* | *Glomerellales* | *Plectosphaerellaceae* | *Verticillium dahliae* | *Verticillium dahliae* | Air | Czech Republic | 1 | Vanderwolf et al. (2013a) |
| *Sordariomycetes* | *Glomerellales* | *Plectosphaerellaceae* | *Verticillium insectorum* | *Verticillium insectorum* | Air | Czech Republic | 1 | Vanderwolf et al. (2013a) |
| *Sordariomycetes* | *Glomerellales* | *Plectosphaerellaceae* | *Verticillium nanum* | *Verticillium nanum* | Insects, bat guano | Belgium | 1 | Vanderwolf et al. (2013a) |
| *Sordariomycetes* | *Glomerellales* | *Plectosphaerellaceae* | *Verticillium* spp. | *Verticillium* spp. | Air, soil-sediment, cave wall, insects, wood, bat guano, water | USA, Bahamas, UK, France, Belgium, Slovenia, Czech Republic, Slovakia, Russia, Japan, Australia, France | 12 | Vanderwolf et al. (2013a) |
| *Sordariomycetes* | *Glomerellales* | *Plectosphaerellaceae* | *Verticillium terrestre* | *Verticillium terrestre* | Wood | Romania | 1 | Vanderwolf et al. (2013a) |
| *Sordariomycetes* | *Glomerellales* | *Plectosphaerellaceae* | *Verticillium tricorpus* | *Verticillium tricorpus* | Limestone, cave wall | China | 1 | Jiang et al. (2017) |
| *Sordariomycetes* | *Hypocreales* | *Bionectriaceae* | *Acremoniella atra* | *Acremoniella atra* | Unreported | Poland | 1 | Pusz et al. (2018); Zhang et al. (2021) |
| *Sordariomycetes* | *Hypocreales* | *Bionectriaceae* | *Acremonium alternatum* | *Acremonium alternatum* | Cave wall, soil-sediment | China, Israel, Russia | 3 | Vanderwolf et al. (2013a); zhang et al. (2021) |
| *Sordariomycetes* | *Hypocreales* | *Bionectriaceae* | *Acremonium antarcticum* | *Acremonium antarcticum* | Unreported | China | 1 | Vanderwolf et al. (2013a) |
| *Sordariomycetes* | *Hypocreales* | *Bionectriaceae* | *Acremonium charticola* | *Acremonium charticola* | Cave wall, soil-sediment, bat guano | China, Slovakia, Russia | 3 | Vanderwolf et al. (2013a) |
| *Sordariomycetes* | *Hypocreales* | *Bionectriaceae* | *Acremonium hennebertii* | *Acremonium hennebertii* | unreported | China | 1 | Zhang (2019) |
| *Sordariomycetes* | *Hypocreales* | *Bionectriaceae* | *Acremonium kiliense* | *Acremonium kiliense* | Air, other, soil-sediment | Czech Republic, Russia | 2 | Vanderwolf et al. (2013a) |
| *Sordariomycetes* | *Hypocreales* | *Bionectriaceae* | *Acremonium longisporum* | *Acremonium longisporum* | unreported | China | 1 | zhang et al. (2021) |
| *Sordariomycetes* | *Hypocreales* | *Bionectriaceae* | *Acremonium murorum* | *Acremonium murorum* | Air, bat internal tissue, sediment, soi, bat guano, earthworm casts, other, cave wall | Romania, Belgium, China, Czech Republic, France, Japan, Mexico, Slovakia | 8 | Vanderwolf et al. (2013a); , Zhang et al. (2017) |
| *Sordariomycetes* | *Hypocreales* | *Bionectriaceae* | *Acremonium murorum* | Acremonium murorum var. felinum | Air | China, Poland | 2 | Vanderwolf et al. (2013a); zhang et al. (2021) |
| *Sordariomycetes* | *Hypocreales* | *Bionectriaceae* | *Acremonium rutilum* | *Acremonium rutilum* | soil-sediment, cave wall | Russia, Israel | 2 | Vanderwolf et al. (2013a) |
| *Sordariomycetes* | *Hypocreales* | *Bionectriaceae* | Acremonium spp. | Acremonium spp. | Air, cave wall, earthworm casts, bat guano, insects, live  bat fur, sediment, skin, soil, wood, Organic litter, | Brazil, Canada, China, Czech Republic, France, Germany, Japan, Mexico, Russia, Slovakia, Slovenia,, Spain, South Africa, Thailand, USA | 15 | Vanderwolf et al. (2013a); Taylor et al. (2014); Man et al. (2015); Jiang et al. (2017); Man (2017); Zhang et al. (2017); Leplat et al. (2018); Suetrong et al. (2023) |
| *Sordariomycetes* | *Hypocreales* | *Bionectriaceae* | *Acremonium vitis* | *Acremonium vitis* | Soil-sediment | Bahamas | 1 | Vanderwolf et al. (2013a) |
| *Sordariomycetes* | *Hypocreales* | *Bionectriaceae* | *Beltrania* sp*.* | *Beltrania* sp*.* | Air | Spain | 1 | Vanderwolf et al. (2013a) |
| *Sordariomycetes* | *Hypocreales* | *Bionectriaceae* | *Bionectria ochroleuca* | *Bionectria ochroleuca* | Soil-sediment, other | China, USA, Spain | 3 | Vanderwolf et al. (2013a); Zhang et al. (2021) |
| *Sordariomycetes* | *Hypocreales* | *Bionectriaceae* | *Bionectria* sp. | *Bionectria* sp. | Unreported | China | 1 | Zhang (2019) |
| *Sordariomycetes* | *Hypocreales* | *Bionectriaceae* | Bionectriaceae sp. | Bionectriaceae sp. | Organic litter, soil-sediment | Thailand, USA | 5 | Vanderwolf et al. (2013a); Suetrong et al. (2023) |
| *Sordariomycetes* | *Hypocreales* | *Bionectriaceae* | *Brunneochlamydosporium nepalense* | *Acremonium nepalense* | Cave wall | China, France | 2 | Vanderwolf et al. (2013a); zhang et al. (2021) |
| *Sordariomycetes* | *Hypocreales* | *Bionectriaceae* | *Clonostachys candelabrum* | *Clonostachys candelabrum* | Air | Czech Republic, Slovakia | 2 | Vanderwolf et al. (2013a) |
| *Sordariomycetes* | *Hypocreales* | *Bionectriaceae* | *Clonostachys intermedia* | *Clonostachys intermedia* | Unreported | China | 1 | Zhang et al. (2021) |
| *Sordariomycetes* | *Hypocreales* | *Bionectriaceae* | *Clonostachys rhizophaga* | *Clonostachys rhizophaga* | Bat guano, unreported | China | 1 | Zhang et al. (2021) |
| *Sordariomycetes* | *Hypocreales* | *Bionectriaceae* | *Clonostachys rogersoniana* | *Clonostachys rogersoniana* | Unreported | China | 1 | Zhang et al. (2021) |
| *Sordariomycetes* | *Hypocreales* | *Bionectriaceae* | *Clonostachys rosea* | *Clonostachys rosea* | Air, soil-sediment, bat guano, earthworm casts, insects, limestone, bat guano, candle, rock sufaces | China, India, Poland, Russia, Slovakia, Romania | 6 | Vanderwolf et al. (2013a); Kokurewicz et al. (2016); Jiang et al. (2017); Nováková et al. (2018); Zhang et al. (2021) |
| *Sordariomycetes* | *Hypocreales* | *Bionectriaceae* | *Clonostachys rosea* | Clonostachys rosea f. catenulata | Air, other, cave wall | Poland, Czech Republic | 2 | Vanderwolf et al. (2013a) |
| *Sordariomycetes* | *Hypocreales* | *Bionectriaceae* | *Clonostachys rosea* | *Gliocladium roseum* | Air, bat guano, other, insects, cave wall, soil-sediment, wood | Brazil, China, Czech Republic, Japan, Poland, Puerto Rico, Romania, Slovenia, UK, USA | 10 | Taylor et al. (2013); Vanderwolf et al. (2013a); Jiang et al. (2017a, b) |
| *Sordariomycetes* | *Hypocreales* | *Bionectriaceae* | *Clonostachys solani* | *Bionectria solani* | Air | Romania | 1 | Nováková et al. (2018) |
| *Sordariomycetes* | *Hypocreales* | *Bionectriaceae* | *Clonostachys* spp. | *Clonostachys* spp. | Air, soil, wood, sediment, water, rock | Bulgaria, China, UK, Slovakia, Thailand | 5 | Vanderwolf et al. (2013a, 2015); Jiang et al. (2017a, b); Zhang et al. (2021); Suetrong et al. (2023) |
| *Sordariomycetes* | *Hypocreales* | *Bionectriaceae* | *Cosmospora berkeleyana* | *Acremonium berkeleyanum* | Air, bat guano, insects, cave wall | Czech Republic, Hungary, Slovakia | 3 | Vanderwolf et al. (2013a) |
| *Sordariomycetes* | *Hypocreales* | *Bionectriaceae* | *Cosmospora cavernicola* | *Cosmospora cavernicola* | Rock surface | China | 1 | This study |
| *Sordariomycetes* | *Hypocreales* | *Bionectriaceae* | *Emericellopsis minima* | *Emericellopsis minima* | Unreported | China | 1 | Zhang et al. (2021) |
| *Sordariomycetes* | *Hypocreales* | *Bionectriaceae* | *Emericellopsis* sp. | *Emericellopsis* sp. | Sediment | China | 1 | Man et al. (2015); Man (2017) |
| *Sordariomycetes* | *Hypocreales* | *Bionectriaceae* | *Emericellopsis terricola* | *Emericellopsis terricola* | Soil-sediment, other, cave wall | Czech Republic, Slovakia | 2 | Vanderwolf et al. (2013a) |
| *Sordariomycetes* | *Hypocreales* | *Bionectriaceae* | *Furcasterigmium furcatum* | *Acremonium furcatum* | Air, cave wall, limestone, soil-sediment, unreported | China, Czech Repubilic, Japan, Thiland, USA | 4 | Vanderwolf et al. (2013a); Jiang et al. (2016, 2017); zhang et al. (2021) |
| *Sordariomycetes* | *Hypocreales* | *Bionectriaceae* | *Geosmithia carolliae* | *Geosmithia carolliae* | Bat | Brazil | 1 | Cunha et al. (2020) |
| *Sordariomycetes* | *Hypocreales* | *Bionectriaceae* | *Geosmithia namyslowskii* | *Geosmithia namyslowskii* | Cave wall | France | 1 | Vanderwolf et al. (2013a) |
| *Sordariomycetes* | *Hypocreales* | *Bionectriaceae* | *Geosmithia putterillii* | *Geosmithia putterillii* | Cave wall | France | 1 | Vanderwolf et al. (2013a) |
| *Sordariomycetes* | *Hypocreales* | *Bionectriaceae* | *Geosmithia sp.* | *Geosmithia sp.* | Unreported | France | 1 | Leplat et al. (2018) |
| *Sordariomycetes* | *Hypocreales* | *Bionectriaceae* | *Lasionectria bisepta* | *Acremonium biseptum* | Cave wall | Czech Republic | 1 | Vanderwolf et al. (2013a) |
| *Sordariomycetes* | *Hypocreales* | *Bionectriaceae* | *Lasionectria cerealis* | *Acremonium cereale* | Soil-sediment | Canada, USA | 2 | Vanderwolf et al. (2013a) |
| *Sordariomycetes* | *Hypocreales* | *Bionectriaceae* | *Ovicillium* sp. | *Ovicillium* sp. | Water, | Thailand | 1 | Suetrong et al. (2023) |
| *Sordariomycetes* | *Hypocreales* | *Bionectriaceae* | *Paragliomastix luzulae* | *Gliomastix luzulae* | Unreported | China | 1 | Zhang et al. (2021) |
| *Sordariomycetes* | *Hypocreales* | *Bionectriaceae* | *Phialemonium atrogriseum* | *Acremonium atrogriseum* | Air, cave wall | Czech Republic | 1 | Vanderwolf et al. (2013a) |
| *Sordariomycetes* | *Hypocreales* | *Bionectriaceae* | *Selinia* sp. | *Selinia* sp. | Unreported | Croatia | 1 | Vanderwolf et al. (2013a) |
| *Sordariomycetes* | *Hypocreales* | *Bionectriaceae* | *Sesquicillium phyllophilum* | *Clonostachys phyllophila* | Unreported | China | 1 | Zhang et al. (2021) |
| *Sordariomycetes* | *Hypocreales* | *Bionectriaceae* | *Sesquicillium rossmaniae* | *Bionectria rossmaniae* | Unreported | Bulgaria, China | 2 | Mitova et al. (2017) |
| *Sordariomycetes* | *Hypocreales* | *Bionectriaceae* | *Stephanonectria keithii* | *Stephanonectria keithii* | Air | China | 1 | Jiang et al. (2017) |
| *Sordariomycetes* | *Hypocreales* | *Bionectriaceae* | *Valsonectria roseola* | *Acremonium roseolum* | Air | South Africa | 1 | Vanderwolf et al. (2013a) |
| *Sordariomycetes* | *Hypocreales* | *Bionectriaceae* | *Verruciconidia persicina* | *Acremonium persicinum* | Cave wall unreported | China, USA | 2 | Vanderwolf et al. (2013); Zhang et al. (2017, 2019) |
| *Sordariomycetes* | *Hypocreales* | *Bionectriaceae* | *Verruciconidia persicina* | *Acremonium polychromum* | Soil-sediment | Slovakia | 1 | Vanderwolf et al. (2013a) |
| *Sordariomycetes* | *Hypocreales* | *Bionectriaceae* | *Verruciconidia verruculosa* | *Acremonium verruculosum* | Other, cave wall, soil-sediment | Czech Republic | 1 | Vanderwolf et al. (2013a) |
| *Sordariomycetes* | *Hypocreales* | *Bionectriaceae* | *Waltergamsia fusidioides* | *Waltergamsia fusidioides* | Cave wall | China | 1 | Ma et al. (2025) |
| *Sordariomycetes* | *Hypocreales* | *Calcarisporiaceae* | *Calcarisporium arbuscula* | *Calcarisporium arbuscula* | Wood | USA | 1 | Held et al. (2020) |
| *Sordariomycetes* | *Hypocreales* | *Calcarisporiaceae* | *Calcarisporium cordycipiticola* | *Calcarisporium cordycipiticola* | Wood | USA | 1 | Held et al. (2020) |
| *Sordariomycetes* | *Hypocreales* | *Calcarisporiaceae* | *Calcarisporium* spp. | *Calcarisporium* spp. | Air, organic litter | Brazil, China | 2 | Taylor et al. (2013); Zhang et al. (2017) |
| *Sordariomycetes* | *Hypocreales* | *Clavicipitaceae* | *Claviceps purpurea* | *Claviceps purpurea* | Cave wall | France | 1 | Vanderwolf et al. (2013a) |
| *Sordariomycetes* | *Hypocreales* | *Clavicipitaceae* | *Claviceps* sp. | *Claviceps* sp. | Unreported | China | 1 | Zhang et al. (2021) |
| *Sordariomycetes* | *Hypocreales* | *Clavicipitaceae* | Clavicipitaceae sp. | Clavicipitaceae sp. | Cave wall | France | 1 | Vanderwolf et al. (2013a) |
| *Sordariomycetes* | *Hypocreales* | *Clavicipitaceae* | Clavicipitaceae spp. | Clavicipitaceae spp. | Soil-sediment, organic litter, Unreported | Romania, Thailand | 2 | Suetrong et al. (2023) |
| *Sordariomycetes* | *Hypocreales* | [*Clavicipitaceae*](https://www.indexfungorum.org/Names/families.asp?FamilyName=Clavicipitaceae) | *Conoideocrella luteorostrata* | *Conoideocrella luteorostrata* | Cave wall | France | 1 | Vanderwolf et al. (2013a) |
| *Sordariomycetes* | *Hypocreales* | *Clavicipitaceae* | *Marquandomyces marquandii* | *Marquandomyces marquandii* | Sediment, soil, gunao, earthworm casts, insect, cave wall, rock | Bahamas, China, Czech Republic, Italy, Poland, Romania, Russia, Slovakia, Spain | 9 | Vanderwolf et al. (2013a); Man et al. (2015); Jiang et al. (2017a, b),Nováková et al. (2018) |
| *Sordariomycetes* | *Hypocreales* | *Clavicipitaceae* | *Marquandomyces marquandii* | *Metarhizium marquandii* | Air | China | 1 | Jiang et al. (2017) |
| *Sordariomycetes* | *Hypocreales* | *Clavicipitaceae* | *Metacordyceps chlamydosporia* | *Metacordyceps chlamydosporia* | Air, cave sediment, cave wall, soil-sediment, insect, rock, bat guano | China, France, Peru, Romania, Spain, UK | 6 | Vanderwolf et al. (2013a); Man et al. (2015); Man (2017); Nováková et al. (2018) |
| *Sordariomycetes* | *Hypocreales* | *Clavicipitaceae* | *Metacordyceps chlamydosporia* | *Pochonia chlamydosporia* | Air, cave wall, bat guano, insects, soil-sediment | Argentina, France, Malaysia, Spain | 4 | Vanderwolf et al. (2013a); Wasti et al. (2021) |
| *Sordariomycetes* | *Hypocreales* | *Clavicipitaceae* | *Metacordyceps chlamydosporia* | *Pochonia chlamydosporia var. catenulata* | Air, soil-sediment,bat guano, earthworm casts | Italy, Slovakia | 2 | Vanderwolf et al. (2013a) |
| *Sordariomycetes* | *Hypocreales* | *Clavicipitaceae* | *Metapochonia bulbillosa* | *Metapochonia bulbillosa* | Unreported, wood | China, USA | 2 | Zhang et al. (2017); Held et al. (2020) |
| *Sordariomycetes* | *Hypocreales* | *Clavicipitaceae* | *Metapochonia rubescens* | *Metapochonia rubescens* | Unreported | China | 1 | Zhang et al. (2017) |
| *Sordariomycetes* | *Hypocreales* | *Clavicipitaceae* | *Metapochonia* sp. | *Metapochonia* sp. | Unreported | Bulgaria | 1 | Mitova et al. (2017) |
| *Sordariomycetes* | *Hypocreales* | *Clavicipitaceae* | *Metapochonia suchlasporia* | *Metapochonia suchlasporia* | Air, cave wall, soil | Belgium, China, France, USA | 4 | Vanderwolf et al. (2013a) |
| *Sordariomycetes* | *Hypocreales* | *Clavicipitaceae* | *Metapochonia suchlasporia* | *Pochonia suchlasporia* | Cave wall, air | France, USA | 2 | Vanderwolf et al. (2013a) |
| *Sordariomycetes* | *Hypocreales* | *Clavicipitaceae* | *Metapochonia variabilis* | *Metapochonia variabilis* | Unreported | China | 1 | Zhang et al. (2017) |
| *Sordariomycetes* | *Hypocreales* | *Clavicipitaceae* | *Metarhizium anisopliae* | *Metarhizium anisopliae* | Isopod feces, cave sediment, soil, insect | China, Japan, Romania, Slovakia, Slovenia | 5 | Vanderwolf et al. (2013a); Zhang et al. (2017); Nováková et al. (2018) |
| *Sordariomycetes* | *Hypocreales* | *Clavicipitaceae* | *Metarhizium rileyi* | *Metarhizium rileyi* | Cave wall | China, France | 2 | Vanderwolf et al. (2013a); Zhang (2019) |
| *Sordariomycetes* | *Hypocreales* | *Clavicipitaceae* | *Metarhizium rileyi* | *Nomuraea rileyi* | Cave wall | France | 1 | Vanderwolf et al. (2013a) |
| *Sordariomycetes* | *Hypocreales* | *Clavicipitaceae* | *Metarhizium robertsii* | *Metarhizium robertsii* | Unreported | China | 1 | Zhang (2019) |
| *Sordariomycetes* | *Hypocreales* | *Clavicipitaceae* | *Metarhizium* spp. | *Metarhizium* spp*.* | Air, water, soil-sediment, Organic litter | China, France, Slovakia, Thailand | 4 | Vanderwolf et al. (2013a); Jiang et al. (2017); Zhang et al. (2021); Suetrong et al. (2023) |
| *Sordariomycetes* | *Hypocreales* | *Clavicipitaceae* | *Pochonia chlamydosporia* var. *chlamydosporia* | *Pochonia chlamydosporia* var. *chlamydosporia* | Soil-sediment | Slovakia | 1 | Vanderwolf et al. (2013a) |
| *Sordariomycetes* | *Hypocreales* | *Clavicipitaceae* | *Pochonia* spp. | *Pochonia* spp. | Air, cave wall, sediment | Bulgaria, China, France, USA | 4 | Vanderwolf et al. (2013a); |
| *Sordariomycetes* | *Hypocreales* | *Cordycipitaceae* | *Akanthomyces lecanii* | *Akanthomyces lecanii* | Dung, frass (not bat); sediment, soil, wood, insects | Austria, China, France, Italy | 4 | Vanderwolf et al. (2013a); Zhang et al. (2021) |
| *Sordariomycetes* | *Hypocreales* | *Cordycipitaceae* | *Akanthomyces lecanii* | *Cordyceps confragosa* | Cave wall | France | 1 | Vanderwolf et al. (2013a) |
| *Sordariomycetes* | *Hypocreales* | *Cordycipitaceae* | *Akanthomyces lecanii* | *Lecanicillium lecanii* | Soil-sediment, wood, insects | France, Italy, Slovenia | 3 | Vanderwolf et al. (2013a) |
| *Sordariomycetes* | *Hypocreales* | *Cordycipitaceae* | *Akanthomyces muscarius* | *Akanthomyces muscarius* | Soil and sediment | Slovakia | 1 | Vanderwolf et al. (2013a); Savković et al. (2025) |
| *Sordariomycetes* | *Hypocreales* | *Cordycipitaceae* | *Akanthomyces muscarius* | *Lecanicillium muscarium* | Soil-sedimentbat guano, insects | Czech Republic, Slovakia | 2 | Vanderwolf et al. (2013a) |
| *Sordariomycetes* | *Hypocreales* | *Cordycipitaceae* | *Amphichorda cavernicola* | *Amphichorda cavernicola* | Animal faeces, bat guano, plant debris, and soil | China | 1 | Zhang et al. (2021) |
| *Sordariomycetes* | *Hypocreales* | *Cordycipitaceae* | *Amphichorda felina* | *Amphichorda felina* | Air, rock | China, North America, Spain | 3 | Zhang et al. (2021) |
| *Sordariomycetes* | *Hypocreales* | *Cordycipitaceae* | *Amphichorda felina* | *Beauveria felina* | Air, bat guano, dung, frass (not bat), bat guano | Bahamas, China, Spain | 3 | Vanderwolf et al. (2013a); Man (2017) |
| *Sordariomycetes* | *Hypocreales* | *Cordycipitaceae* | *Beauveria bassiana* | *Beauveria bassiana* | Air, insects, unreported, other, soil-sediment, bat guano, cave wall | Belgium, Brazil, Canada, China, Czech Republic, France, Japan, Netherlands, Slovakia, Slovenia, Switzerland, UK, USA | 13 | Vanderwolf et al. (2013a); Cunha et al. (2020); Zhang et al. (2021) |
| *Sordariomycetes* | *Hypocreales* | *Cordycipitaceae* | *Beauveria brongniartii* | *Beauveria brongniartii* | Air, insects | China, Czech Republic, Slovakia | 3 | Vanderwolf et al. (2013a) |
| *Sordariomycetes* | *Hypocreales* | *Cordycipitaceae* | *Beauveria caledonica* | *Beauveria caledonica* | soil, insect | Belgium, China, USA | 3 | Yoder et al. (2015) |
| *Sordariomycetes* | *Hypocreales* | *Cordycipitaceae* | *Beauveria* spp. | *Beauveria* spp. | Insects, bat guano | France, Netherlands, Slovakia, USA | 4 | Vanderwolf et al. (2013, 2019); Leplat et al. (2018) |
| *Sordariomycetes* | *Hypocreales* | *Cordycipitaceae* | *Beauveria varroae* | *Beauveria varroae* | Air | Spain | 1 | Savković et al. (2025) |
| *Sordariomycetes* | *Hypocreales* | *Cordycipitaceae* | *Conoideocrella luteorostrata* | *Torrubiella luteorostrata* | Cave wall | France | 1 | Vanderwolf et al. (2013a) |
| *Sordariomycetes* | *Hypocreales* | *Cordycipitaceae* | *Cordyceps amoene-rosea* | *Isaria amoene-rosea* | Unreported | Unknow |  | Zhang et al. (2021) |
| *Sordariomycetes* | *Hypocreales* | *Cordycipitaceae* | *Cordyceps amoene-rosea* | *Paecilomyces amoene-roseus* | Cave wall | France | 1 | Vanderwolf et al. (2013a) |
| *Sordariomycetes* | *Hypocreales* | *Cordycipitaceae* | *Cordyceps farinosa* | *Cordyceps farinosa* | Air, insect, soil-sediment, cave wall, bat guano, earthworm casts | Canada, China, Czech Republic, France, Italy, Netherlands, Slovakia, Slovenia, UK | 9 | Vanderwolf et al. (2013a, 2015); Zhang et al. (2017); |
| *Sordariomycetes* | *Hypocreales* | *Cordycipitaceae* | *Cordyceps farinosa* | *Isaria farinosa* | Bat guano, insects, soil-sediment, cave wall, earthworm casts, other | Canada, Czech Republic, France, Italy, Netherlands, Slovakia, Slovenia, UK | 8 | Vanderwolf et al. (2013a) |
| *Sordariomycetes* | *Hypocreales* | *Cordycipitaceae* | *Cordyceps fumosorosea* | *Cordyceps fumosorosea* | Air, cave wall, cave water, insect, soil-sediment, bat guano | China, Czech Republic, France, Poland, Slovakia, Slovenia | 6 | Vanderwolf et al. (2013a); Jiang et al. (2017a, b); Zhang et al. (2017) |
| *Sordariomycetes* | *Hypocreales* | *Cordycipitaceae* | *Cordyceps fumosorosea* | *Isaria fumosorosea* | Air, cave wall, insects, soil-sediment, bat guano, earthworm casts, water | China, Czech Republic, France, Poland, Slovakia, Slovenia, | 6 | Vanderwolf et al. (2013a); Jiang et al. (2017) |
| *Sordariomycetes* | *Hypocreales* | *Cordycipitaceae* | *Cordyceps fumosorosea* | *Paecilomyces fumosoroseus* | Air | Poland | 1 | Kokurewicz et al. (2016) |
| *Sordariomycetes* | *Hypocreales* | *Cordycipitaceae* | *Cordyceps militaris* | *Cordyceps militaris* | Unreported | China | 1 | Zhang et al. (2021) |
| *Sordariomycetes* | *Hypocreales* | *Cordycipitaceae* | *Cordyceps odyneri* | *Cordyceps odyneri* | Unreported | Switzerland | 1 | Vanderwolf et al. (2013a) |
| *Sordariomycetes* | *Hypocreales* | *Cordycipitaceae* | *Cordyceps polyarthra* | *Cordyceps polyarthra* | Unreported | China | 1 | Zhang et al. (2021) |
| *Sordariomycetes* | *Hypocreales* | *Cordycipitaceae* | *Cordyceps riverae* | *Cordyceps riverae* | Insects | Croatia, Italy | 2 | Vanderwolf et al. (2013a) |
| *Sordariomycetes* | *Hypocreales* | *Cordycipitaceae* | Cordyceps spp. | Cordyceps spp. | Air, insects, soil-sediment, unreported | Algeria, Austria, Croatia, Czech Republic, France, UK | 4 | Vanderwolf et al. (2013a, 2015); Suetrong et al. (2023) |
| *Sordariomycetes* | *Hypocreales* | *Cordycipitaceae* | *Cordyceps tenuipes* | *Cordyceps tenuipes* | Unreported | China | 1 | Zhang et al. (2021) |
| *Sordariomycetes* | *Hypocreales* | *Cordycipitaceae* | *Cordycipitaceae* | *Pseudolecanicillium caatingaense* | Air | Brazil | 1 | Alves et al. (2022) |
| *Sordariomycetes* | *Hypocreales* | *Cordycipitaceae* | Cordycipitaceae sp. | Cordycipitaceae sp. | Unreported | Thailand | 1 | Suetrong et al. (2023) |
| *Sordariomycetes* | *Hypocreales* | *Cordycipitaceae* | *Corniculantispora aranearum* | *Lecanicillium aphanocladii* | Air | Brazil, China, Spain | 3 | Tavares et al. (2018); Cardoso et al. (2019); |
| *Sordariomycetes* | *Hypocreales* | *Cordycipitaceae* | *Corniculantispora psalliotae* | *Lecanicillium psalliotae* | Other, cave wall, insects, air | France, Spain, Slovenia, Czech Republic, Slovakia | 5 | Vanderwolf et al. (2013a) |
| *Sordariomycetes* | *Hypocreales* | *Cordycipitaceae* | *Engyodontium parvisporum* | *Engyodontium parvisporum* | Insects | Czech Republic | 1 | Vanderwolf et al. (2013a) |
| *Sordariomycetes* | *Hypocreales* | *Cordycipitaceae* | *Engyodontium rectidentatum* | *Engyodontium rectidentatum* | Insects, air, cave wall | Czech Republic | 1 | Vanderwolf et al. (2013a) |
| *Sordariomycetes* | *Hypocreales* | *Cordycipitaceae* | Engyodontium spp. | Engyodontium spp. | Cave wall, air | USA, France | 2 | Vanderwolf et al. (2013a) |
| *Sordariomycetes* | *Hypocreales* | *Cordycipitaceae* | *Gamszarea humicola* | *Gamszarea humicola* | Soil | China | 1 | zhang et al. (2021) |
| *Sordariomycetes* | *Hypocreales* | *Cordycipitaceae* | *Gamszarea kalimantanensis* | *Gamszarea kalimantanensis* | Unreported | China | 1 | Zhang et al. (2021) |
| *Sordariomycetes* | *Hypocreales* | *Cordycipitaceae* | *Gamszarea kalimantanensis* | *Lecanicillium kalimantanense* | Cave wall | USA | 1 | Vanderwolf et al. (2013a) |
| *Sordariomycetes* | *Hypocreales* | *Cordycipitaceae* | *Gamszarea lunata* | *Gamszarea lunata* | Rock, unreported | China | 1 | Zhang et al. (2021) |
| *Sordariomycetes* | *Hypocreales* | *Cordycipitaceae* | *Gamszarea microspora* | *Gamszarea microspora* | Rock | China | 1 | zhang et al. (2021) |
| *Sordariomycetes* | *Hypocreales* | *Cordycipitaceae* | *Gibellula arachnophila* | *Isaria arachnophila* | Insects | France | 1 | Vanderwolf et al. (2013a) |
| *Sordariomycetes* | *Hypocreales* | *Cordycipitaceae* | *Gibellula arachnophila* | *Torrubiella arachnophila* | Unreported | Unknow |  | Zhang et al. (2021) |
| *Sordariomycetes* | *Hypocreales* | *Cordycipitaceae* | *Gibellula* sp. | *Gibellula* sp. | Unreported | Croatia | 1 | Vanderwolf et al. (2013a) |
| *Sordariomycetes* | *Hypocreales* | *Cordycipitaceae* | *Hirsutella guignardii* | *Isaria guignardii* | Insects | France, Spain, Netherlands | 3 | Vanderwolf et al. (2013a) |
| *Sordariomycetes* | *Hypocreales* | *Cordycipitaceae* | *Isaria cicadae* | *Cordyceps cicadae* | Unreported | China | 1 | Zhang et al. (2021) |
| *Sordariomycetes* | *Hypocreales* | *Cordycipitaceae* | *Isaria spp.* | *Isaria* spp. | Air, soil-sediment, insects, dung, frass (not bat); unreported, cave wall, bat guano | Algeria, Australia, France, Puerto Rico, Slovakia, Spain, USA | 7 | Vanderwolf et al. (2013a) |
| *Sordariomycetes* | *Hypocreales* | *Cordycipitaceae* | *Lecanicillium dimorphum* | *Lecanicillium dimorphosporum* | Sediment | Romania | 1 | Nováková et al. (2018) |
| *Sordariomycetes* | *Hypocreales* | *Cordycipitaceae* | *Lecanicillium fusisporum* | *Lecanicillium fusisporum* | Insects | Slovenia | 1 | Vanderwolf et al. (2013a) |
| *Sordariomycetes* | *Hypocreales* | *Cordycipitaceae* | *Lecanicillium magnisporum* | *Lecanicillium magnisporum* | Soil | China | 1 | zhang et al. (2021) |
| *Sordariomycetes* | *Hypocreales* | *Cordycipitaceae* | *Lecanicillium saksenae* | *Lecanicillium saksenae* | Air | Spain | 1 | Savković et al. (2025) |
| *Sordariomycetes* | *Hypocreales* | *Cordycipitaceae* | *Lecanicillium* spp. | *Lecanicillium* spp. | Air, Bamboo, rock surface, cave wall, insect, Organic litter | China, Czech Republic, France, Thailand, USA | 5 | Vanderwolf et al. (2013a); Man et al. (2015); Leplat et al. (2018); Suetrong et al. (2023) |
| *Sordariomycetes* | *Hypocreales* | *Cordycipitaceae* | *Lecanicillium tenuipes* | *Engyodontium aranearum* | Insects | Italy | 1 | Vanderwolf et al. (2013a) |
| *Sordariomycetes* | *Hypocreales* | *Cordycipitaceae* | *Leptobacillium cavernicola* | *Leptobacillium cavernicola* | Air, cave wall | France | 1 | Leplat et al. 2022 |
| *Sordariomycetes* | *Hypocreales* | *Cordycipitaceae* | *Leptobacillium leptobactrum* | *Leptobacillium leptobactrum* | Air, water, soil-sediment | Czech Republic, France, Germany, Italy, USA | 5 | Vanderwolf et al. (2013a); Zhang et al. (2014); Burow et al. (2019) |
| *Sordariomycetes* | *Hypocreales* | *Cordycipitaceae* | *Neogamszarella antillana* | *Lecanicillium antillanum* | Wood | Germany | 1 | Vanderwolf et al. (2013a) |
| *Sordariomycetes* | *Hypocreales* | *Cordycipitaceae* | *Parengyodontium album* | *Engyodontium album* | Cave wall, air, soil-sediment | China, Czech Republic, France, Slovakia, USA | 5 | Vanderwolf et al. (2013a); Ma et al. (2025) |
| *Sordariomycetes* | *Hypocreales* | *Cordycipitaceae* | *Parengyodontium album* | *Parengyodontium album* | Air, soil, asymptomatic specimens of Proteus anguinus | Belgium, China, France, Romania, Slovenia, Spain, UK | 7 | Vanderwolf et al. (2013a); Nováková et al. (2018); Zhang et al. (2021) |
| *Sordariomycetes* | *Hypocreales* | *Cordycipitaceae* | *Parengyodontium* spp. | *Parengyodontium* spp. | Organic litter, air, | China, Thailand | 1 | Zhang (2019); Suetrong et al. (2023) |
| *Sordariomycetes* | *Hypocreales* | *Cordycipitaceae* | *Samsoniella hepiali* | *Paecilomyces hepiali* | Unreported | China | 1 | Zhang et al. (2021) |
| *Sordariomycetes* | *Hypocreales* | *Cordycipitaceae* | *Simplicillium album* | *Simplicillium album* | Animal faeces, soil | China | 1 | zhang et al. (2021) |
| *Sordariomycetes* | *Hypocreales* | *Cordycipitaceae* | *Simplicillium aogashimaense* | *Simplicillium aogashimaense* | Unreported | China | 1 | Zhang et al. (2021) |
| *Sordariomycetes* | *Hypocreales* | *Cordycipitaceae* | *Simplicillium calcicola* | *Simplicillium calcicola* | Unreported | China | 1 | Zhang et al. (2017) |
| *Sordariomycetes* | *Hypocreales* | *Cordycipitaceae* | *Simplicillium cylindrosporum* | *Simplicillium cylindrosporum* | Unreported | China | 1 | Zhang (2019) |
| *Sordariomycetes* | *Hypocreales* | *Cordycipitaceae* | *Simplicillium humicola* | *Simplicillium humicola* | Soil | China | 1 | zhang et al. (2021) |
| *Sordariomycetes* | *Hypocreales* | *Cordycipitaceae* | *Simplicillium lamellicola* | *Simplicillium lamellicola* | Air, cave sediment, cave wall, , insect, gunao | Belgium, Canada, China, Czech Republic, Romania, Slovakia, USA | 7 | Vanderwolf et al. (2013a); Nováková et al. (2018) |
| *Sordariomycetes* | *Hypocreales* | *Cordycipitaceae* | *Simplicillium lanosoniveum* | *Cephalosporium lanosoniveum* | Unreported | China | 1 | Pereira et al. (2022) |
| *Sordariomycetes* | *Hypocreales* | *Cordycipitaceae* | *Simplicillium lanosoniveum* | *Simplicillium lanosoniveum* | bat | USA | 1 | Zhang et al. (2014) |
| *Sordariomycetes* | *Hypocreales* | *Cordycipitaceae* | *Simplicillium minatense* | *Simplicillium minatense* | Unreported | Bulgaria, China | 2 | Mitova et al. (2017) |
| *Sordariomycetes* | *Hypocreales* | *Cordycipitaceae* | *Simplicillium pechmerlense* | *Simplicillium pechmerlense* | Air | France | 1 | Savković et al. (2025) |
| *Sordariomycetes* | *Hypocreales* | *Cordycipitaceae* | *Simplicillium* spp. | *Simplicillium* spp. | Air, cave wall, soil, water | Bulgaria, China, France, Thailand, USA | 5 | Vanderwolf et al. (2013a); Jiang et al. (2017a, b); Leplat et al. (2018); Suetrong et al. (2023) |
| *Sordariomycetes* | *Hypocreales* | *Cordycipitaceae* | *Simplicillium subtropicum* | *Simplicillium subtropicum* | Unreported | China | 1 | Zhang et al. (2021) |
| *Sordariomycetes* | *Hypocreales* | *Cordycipitaceae* | *Torrubiella minutissima* | *Torrubiella minutissima* | Insects | France | 1 | Vanderwolf et al. (2013a) |
| *Sordariomycetes* | *Hypocreales* | *Cordycipitaceae* | *Torrubiella* spp. | *Torrubiella* spp. | Unreported, cave wall, air | Croatia, Czech Republic | 2 | Vanderwolf et al. (2013a) |
| *Sordariomycetes* | *Hypocreales* | *Hypocreaceae* | *Acrostalagmus luteoalbus* | *Acrostalagmus luteoalbus* | Air, bat guano, water, soil-sediment, wood | China, Japan, Romania, Slovakia, USA | 5 | Vanderwolf et al. (2013a); Man et al. (2015); Man (2017); Zhang et al. (2017,2021) |
| *Sordariomycetes* | *Hypocreales* | *Hypocreaceae* | Acrostalagmus sp. | *Acrostalagmus alboluteum* | Unreported | China | 1 | Man (2017) |
| *Sordariomycetes* | *Hypocreales* | *Hypocreaceae* | *Brunneochlamydosporium cibotii* | *Gliocladium cibotii* | Soil-sediment | Japan | 1 | Vanderwolf et al. (2013a) |
| *Sordariomycetes* | *Hypocreales* | *Hypocreaceae* | *Cladobotryum* sp. | *Cladobotryum* sp. | Soil | Romania | 1 | Nováková et al. (2018) |
| *Sordariomycetes* | *Hypocreales* | *Hypocreaceae* | *Gliocladium atrum* | *Gliocladium atrum* | Cave wall | Czech Republic | 1 | Vanderwolf et al. (2013a) |
| *Sordariomycetes* | *Hypocreales* | *Hypocreaceae* | *Gliocladium* spp. | *Gliocladium* spp. | Unreported, bat guano, soil-sediment, insects, wood | Belgium, France, Kazakhstan, Puerto Rico, USA | 6 | Vanderwolf et al. (2013a) |
| *Sordariomycetes* | *Hypocreales* | *Hypocreaceae* | *Hypocrea fragarioides* | *Hypocrea fragarioides* | Air, sediment | France | 1 | Vanderwolf et al. (2013a) |
| *Sordariomycetes* | *Hypocreales* | *Hypocreaceae* | *Hypocrea lactea* | *Hypocrea lactea* | Unreported | China | 1 | Zhang et al. (2021) |
| *Sordariomycetes* | *Hypocreales* | *Hypocreaceae* | *Hypocrea pachybasioides* | *Hypocrea pachybasioides* | Air, sediment, wood | China, Poland, USA | 3 | Zhang et al. (2014); Pusz et al. (2015); Held et al. (2020) |
| *Sordariomycetes* | *Hypocreales* | *Hypocreaceae* | Hypocrea sp. | *Hypocrea monticulosum* | Unreported | China | 1 | Zhang et al. (2021) |
| *Sordariomycetes* | *Hypocreales* | *Hypocreaceae* | Hypocrea sp. | *Hypocrea perforatum* | Unreported | China | 1 | Zhang et al. (2017) |
| *Sordariomycetes* | *Hypocreales* | *Hypocreaceae* | *Hypocrea* sp. | *Hypocrea* sp. | Bat guano | Slovakia | 1 | Vanderwolf et al. (2013a) |
| *Sordariomycetes* | *Hypocreales* | *Hypocreaceae* | Hypocreaceae sp. | Hypocreaceae sp. | Bat guano, rock | China | 1 | Man (2017) |
| *Sordariomycetes* | *Hypocreales* | *Hypocreaceae* | *Hypomyces rosellus* | *Hypomyces rosellus* | Unreported | Romania | 1 | Vanderwolf et al. (2013a) |
| *Sordariomycetes* | *Hypocreales* | *Hypocreaceae* | *Hypomyces* sp. | *Hypomyces* sp. | Soil-sediment | USA | 1 | Vanderwolf et al. (2013a) |
| *Sordariomycetes* | *Hypocreales* | *Hypocreaceae* | *Mycogone nigra* | *Mycogone nigra* | Soil-sediment | Russia | 1 | Vanderwolf et al. (2013a) |
| *Sordariomycetes* | *Hypocreales* | *Hypocreaceae* | *Mycogone* sp. | *Mycogone* sp. | Soil-sediment | India | 1 | Vanderwolf et al. (2013a) |
| *Sordariomycetes* | *Hypocreales* | *Hypocreaceae* | *Protocrea farinosa* | *Protocrea farinosa* | Unreported | China | 1 | Zhang et al. (2017) |
| *Sordariomycetes* | *Hypocreales* | *Hypocreaceae* | *Sepedonium* spp. | Sepedonium spp. | Unreported, bat guano, soil-sediment | India, Puerto Rico, USA | 3 | Vanderwolf et al. (2013a) |
| *Sordariomycetes* | *Hypocreales* | *Hypocreaceae* | *Sphaerostilbella penicillioides* | *Gliocladium penicillioides* | Unreported | Kazakhstan | 1 | Vanderwolf et al. (2013a) |
| *Sordariomycetes* | *Hypocreales* | *Hypocreaceae* | *Sphaerostilbella penicillioides* | *Sphaerostilbella penicillioides* | Unreported | Kazakhstan | 1 | Vanderwolf et al. (2013a) |
| *Sordariomycetes* | *Hypocreales* | *Hypocreaceae* | *Trichoderma asperelloides* | *Trichoderma asperelloides* | Air, microbial mat (Airbell II); cave sediment | China, Romania | 2 | Nováková et al. (2018) |
| *Sordariomycetes* | *Hypocreales* | *Hypocreaceae* | *Trichoderma asperellum* | *Trichoderma asperellum* | bat guano, speleothem | China, Malaysia | 1 | Wasti et al. (2021); Zhang et al. (2021); |
| *Sordariomycetes* | *Hypocreales* | *Hypocreaceae* | *Trichoderma atroviride* | *Trichoderma atroviride* | Air, dead isopod bodies, soil-sediment, bat guano, insect, petroleum wax, rock surface | China, Romania, Slovakia | 3 | Vanderwolf et al. (2013a); Zhang et al. (2017); Nováková et al. (2018); Ma et al. (2025) |
| *Sordariomycetes* | *Hypocreales* | *Hypocreaceae* | *Trichoderma aureoviride* | *Trichoderma aureoviride* | Unreported | China | 1 | Zhang et al. (2021) |
| *Sordariomycetes* | *Hypocreales* | *Hypocreaceae* | *Trichoderma brevicompactum* | *Trichoderma brevicompactum* | Air | Algeria, Brazil, Canada, China, Poland, Romania, Slovakia, Spain | 8 | Zhang et al. (2021) |
| *Sordariomycetes* | *Hypocreales* | *Hypocreaceae* | *Trichoderma capillare* | *Trichoderma capillare* | Decaying wood | China | 1 | Liu et al. (2025) |
| *Sordariomycetes* | *Hypocreales* | *Hypocreaceae* | *Trichoderma cavernicola* | *Trichoderma cavernicola* | Bat guano | China | 1 | Liu et al. (2025) |
| *Sordariomycetes* | *Hypocreales* | *Hypocreaceae* | *Trichoderma citrinoviride* | *Trichoderma citrinoviride* | Air, cave water | China, Poland, Slovakia | 3 | Zhang et al. (2017); Ogórek (2018a, b) |
| *Sordariomycetes* | *Hypocreales* | *Hypocreaceae* | *Trichoderma dehongense* | *Trichoderma dehongense* | Soil | China | 1 | This study |
| *Sordariomycetes* | *Hypocreales* | *Hypocreaceae* | *Trichoderma deliquescens* | *Gliocladium deliquescens* | Wood | Romania | 1 | Vanderwolf et al. (2013a) |
| *Sordariomycetes* | *Hypocreales* | *Hypocreaceae* | *Trichoderma deliquescens* | *Trichoderma deliquescens* | Wood | Romania | 1 | Vanderwolf et al. (2013a) |
| *Sordariomycetes* | *Hypocreales* | *Hypocreaceae* | *Trichoderma erinaceum* | *Trichoderma erinaceum* | Bat guano | Peru | 1 | Savković et al. (2025) |
| *Sordariomycetes* | *Hypocreales* | *Hypocreaceae* | *Trichoderma gamsii* | *Trichoderma gamsii* | Air | China, Spain | 2 | Zhang et al. (2021) |
| *Sordariomycetes* | *Hypocreales* | *Hypocreaceae* | *Trichoderma ghanense* | *Trichoderma ghanense* | Bat guano | France | 1 | Savković et al. (2025) |
| *Sordariomycetes* | *Hypocreales* | *Hypocreaceae* | *Trichoderma hamatum* | *Trichoderma hamatum* | Bat guano, soil-sediment, insects | China, France, Japan, Slovakia | 4 | Vanderwolf et al. (2013a); Man (2017); Zhang et al. (2021) |
| *Sordariomycetes* | *Hypocreales* | *Hypocreaceae* | *Trichoderma harzianum* | *Trichoderma harzianum* | Air, bat carcasses, soil-sediment, other, cave wall, water, bat guano, feather, rock sufaces, soil | Bulgaria, China, Indonesia, Italy, Japan, Korea, Malaysia, Poland, Romania, Slovakia, Thailand, USA | 12 | Vanderwolf et al. (2013a); Kokurewicz et al. (2016); Jiang et al. (2017); Karunarathna et al. (2020); Wasti et al. (2021) |
| *Sordariomycetes* | *Hypocreales* | *Hypocreaceae* | *Trichoderma hongheense* | *Trichoderma hongheense* | Air | China | 1 | This study |
| *Sordariomycetes* | *Hypocreales* | *Hypocreaceae* | *Trichoderma hydei* | *Trichoderma hydei* | Rock surface | China | 1 | Hongsanan et al. (2025) |
| *Sordariomycetes* | *Hypocreales* | *Hypocreaceae* | *Trichoderma koningii* | *Hypocrea koningii* | Soil-sediment | Australia | 1 | Vanderwolf et al. (2013a) |
| *Sordariomycetes* | *Hypocreales* | *Hypocreaceae* | *Trichoderma koningii* | *Trichoderma koningii* | Air, soil-sediment, other, wood | China, Czech Republic, France, Italy, Poland, Puerto Rico, Romania, Russia, Slovakia | 9 | Vanderwolf et al. (2013a); Nováková et al. (2018) |
| *Sordariomycetes* | *Hypocreales* | *Hypocreaceae* | *Trichoderma koningiopsis* | *Trichoderma koningiopsis* | Bat guano | China, Poland | 2 | Pusz et al. (2017); Zhang et al. (2017) |
| *Sordariomycetes* | *Hypocreales* | *Hypocreaceae* | *Trichoderma lixii* | *Hypocrea lixii* | Soil-sediment, wood | Germany, Spain | 2 | Vanderwolf et al. (2013a) |
| *Sordariomycetes* | *Hypocreales* | *Hypocreaceae* | *Trichoderma lixii* | *Trichoderma lixii* | Air, bat guano, soil-sediment, wood | China, Germany, Spain | 3 | Vanderwolf et al. (2013a); Zhang et al. (2017) |
| *Sordariomycetes* | *Hypocreales* | *Hypocreaceae* | *Trichoderma longibrachiatum* | *Trichoderma longibrachiatum* | Air, soil-sediment, bat guano, petroleum wax | Australia, China, Slovakia | 3 | Vanderwolf et al. (2013a); Man (2017); Zhang et al. (2021) |
| *Sordariomycetes* | *Hypocreales* | *Hypocreaceae* | *Trichoderma mangshiense* | *Trichoderma mangshiense* | Soil | China | 1 | This study |
| *Sordariomycetes* | *Hypocreales* | *Hypocreaceae* | *Trichoderma neogongcheniae* | *Trichoderma neogongcheniae* | Feather | China | 1 | This study |
| *Sordariomycetes* | *Hypocreales* | *Hypocreaceae* | *Trichoderma neopyramidale* | *Trichoderma neopyramidale* | Rock surface | China | 1 | This study |
| *Sordariomycetes* | *Hypocreales* | *Hypocreaceae* | *Trichoderma nujiangensis* | *Trichoderma nujiangensis* | Rock surface | China | 1 | This study |
| *Sordariomycetes* | *Hypocreales* | *Hypocreaceae* | *Trichoderma oblongisporum* | *Trichoderma oblongisporum* | Wood | USA | 1 | Held et al. (2020) |
| *Sordariomycetes* | *Hypocreales* | *Hypocreaceae* | *Trichoderma obovatum* | *Trichoderma obovatum* | Petroleum wax | China | 1 | This study |
| *Sordariomycetes* | *Hypocreales* | *Hypocreaceae* | *Trichoderma parareesei* | *Trichoderma parareesei* | Unreported | China | 1 | Zhang et al. (2021) |
| *Sordariomycetes* | *Hypocreales* | *Hypocreaceae* | *Trichoderma paraviridescens* | *Trichoderma paraviridescens* | Bat guano, sediment | China, Malaysia, USA | 3 | Wasti et al. (2021); Zhang et al. (2021) |
| *Sordariomycetes* | *Hypocreales* | *Hypocreaceae* | *Trichoderma piluliferum* | *Trichoderma piluliferum* | Soil-sediment | Thailand | 1 | Vanderwolf et al. (2013a) |
| *Sordariomycetes* | *Hypocreales* | *Hypocreaceae* | *Trichoderma polysporum* | *Trichoderma polysporum* | Air, other, soil-sediment, wood, bat guano, earthworm casts, insects | China, Czech Republic, France, Russia, Slovakia, UK, USA | 7 | Vanderwolf et al. (2013a) |
| *Sordariomycetes* | *Hypocreales* | *Hypocreaceae* | *Trichoderma reesei* | *Trichoderma reesei* | Sediment | China | 1 | Man et al. (2015); Man (2017) |
| *Sordariomycetes* | *Hypocreales* | *Hypocreaceae* | *Trichoderma rossicum* | *Trichoderma rossicum* | Unreported | China | 1 | Zhang et al. (2017) |
| *Sordariomycetes* | *Hypocreales* | *Hypocreaceae* | *Trichoderma samuelsii* | *Trichoderma samuelsii* | Unreported | China | 1 | Zhang et al. (2021) |
| *Sordariomycetes* | *Hypocreales* | *Hypocreaceae* | *Trichoderma saturnisporum* | *Trichoderma saturnisporum* | Unreported | China | 1 | Zhang et al. (2021) |
| *Sordariomycetes* | *Hypocreales* | *Hypocreaceae* | *Trichoderma simmonsii* | *Trichoderma simmonsii* | Bat guano | China, Slovenia | 1 | Savković et al. (2025) |
| *Sordariomycetes* | *Hypocreales* | *Hypocreaceae* | *Trichoderma spirale* | *Trichoderma spirale* | Unreported | China | 1 | Zhang et al. (2021) |
| *Sordariomycetes* | *Hypocreales* | *Hypocreaceae* | *Trichoderma* spp. | *Trichoderma* spp. | Air,l, insects, bat guano, soil-sediment, cave wall, air, earthworm casts, organic litter, other, unreported | Australia, Bulgaria, Canada, China, France, Japan, Kazakhstan, Korea, Puerto Rico, Romania, Slovakia, Slovenia, Thailand, USA | 14 | Vanderwolf et al. (2013, 2015, 2019); Man (2017); Zhang et al. (2021); Suetrong et al. (2023) |
| *Sordariomycetes* | *Hypocreales* | *Hypocreaceae* | *Trichoderma stellatum* | *Hypocrea stellata* | Earthworm casts | Slovakia | 1 | Vanderwolf et al. (2013a) |
| *Sordariomycetes* | *Hypocreales* | *Hypocreaceae* | *Trichoderma stramineum* | *Trichoderma stramineum* | Unreported | China | 1 | Zhang et al. (2021) |
| *Sordariomycetes* | *Hypocreales* | *Hypocreaceae* | *Trichoderma strictipile* | *Trichoderma strictipile* | Unreported | China | 1 | Zhang et al. (2021) |
| *Sordariomycetes* | *Hypocreales* | *Hypocreaceae* | *Trichoderma tomentosum* | *Trichoderma tomentosum* | Air | China | 1 | Jiang et al. (2017) |
| *Sordariomycetes* | *Hypocreales* | *Hypocreaceae* | *Trichoderma trixiae* | *Trichoderma trixiae* | Art Galery | Bulgaria | 1 | Savković et al. (2025) |
| *Sordariomycetes* | *Hypocreales* | *Hypocreaceae* | *Trichoderma velutinum* | *Trichoderma velutinum* | Unreported | China | 1 | Zhang et al. (2021) |
| *Sordariomycetes* | *Hypocreales* | *Hypocreaceae* | *Trichoderma virens* | *Hypocrea virens* | Soil-sediment | USA | 1 | Vanderwolf et al. (2013a) |
| *Sordariomycetes* | *Hypocreales* | *Hypocreaceae* | *Trichoderma virens* | *Trichoderma virens* | Air, soil, cave sediment, isopod feces, dead isopod bodies (*Trachelipus troglobius*); underwater sediment of Airbell II, microbial mat (Airbell II) | Romania, Serbia, USA | 3 | Vanderwolf et al. (2013a); Nováková et al. (2018) |
| *Sordariomycetes* | *Hypocreales* | *Hypocreaceae* | *Trichoderma viridarium* | *Trichoderma viridarium* | Soil | Belgium | 1 | Savković et al. (2025) |
| *Sordariomycetes* | *Hypocreales* | *Hypocreaceae* | *Trichoderma viride* | *Trichoderma viride* | Air, Unreported, water, soil-sediment, slate precipitate, bat guano, wood, insects, other, cave wall | Belgium, Brazil, China, Czech Republic, Germany, India, Korea, Poland, Puerto Rico, Romania, Russia, Slovakia, Switzerland, UK, Ukraine, USA | 16 | Vanderwolf et al. (2013a); Taylor et al. (2013, 2014) , Pusz et al. (2017),Burow et al. (2019); Zhang et al. (2021) |
| *Sordariomycetes* | *Hypocreales* | *Hypocreaceae* | *Trichoderma viridescens* | *Trichoderma viridescens* | Unreported | China, Germany | 2 | Burow et al. (2019) |
| *Sordariomycetes* | *Hypocreales* | *Hypocreaceae* | *Trichoderma yingjiangens* | *Trichoderma yingjiangens* | Bat guano | China | 1 | This study |
| *Sordariomycetes* | *Hypocreales* | *Incertae sedis* | *Acremonium murorum* | *Gliomastix murorum* | Air, cave wall, limestone, other, soil-sediment, wood | Bulgaria, China, Poland, Romania, Thailand, USA | 6 | Vanderwolf et al. (2013a); Jiang et al. (2017a, b); Mitova et al. (2017) |
| *Sordariomycetes* | *Hypocreales* | *Incertae sedis* | *Albifimbria verrucaria* | *Myrothecium verrucaria* | Soil-sediment, wood | Italy, Kazakhstan, Romania, Slovakia | 4 | Vanderwolf et al. (2013a) |
| *Sordariomycetes* | *Hypocreales* | *Incertae sedis* | *Cephalosporium atrum* | *Cephalosporium atrum* | Soil-sediment, cave wall | Russia | 1 | Vanderwolf et al. (2013a) |
| *Sordariomycetes* | *Hypocreales* | *Incertae sedis* | *Cephalosporium* spp. | *Cephalosporium* spp. | Bat guano, other, wood | Belgium, Czech Republic | 2 | Vanderwolf et al. (2013a) |
| *Sordariomycetes* | *Hypocreales* | *Incertae sedis* | *Gliomastix* spp. | *Gliomastix* spp. | Air, unreported, soil-sediment | France, Spain, Thailand, USA | 4 | Vanderwolf et al. (2013a); Leplat et al. (2018); Suetrong et al. (2023) |
| *Sordariomycetes* | *Hypocreales* | *Incertae sedis* | *Hyalopus curtipes* | *Cephalosporium curtipes* | Soil-sediment | Italy | 1 | Vanderwolf et al. (2013a) |
| *Sordariomycetes* | *Hypocreales* | *Incertae sedis* | *Hyalopus curtipes* | *Hyalopus curtipes* | Soil-sediment | Italy | 1 | Vanderwolf et al. (2013a) |
| *Sordariomycetes* | *Hypocreales* | *Incertae sedis* | *Myrothecium* spp. | *Myrothecium* spp. | Soil-sediment | China, Italy, Thailand | 3 | Vanderwolf et al. (2013a); Zhang et al. (2017); Suetrong et al. (2023) |
| *Sordariomycetes* | *Hypocreales* | *Incertae sedis* | *Myxotrichum spp.* | *Myxotrichum* spp. | Unreported | Canada, China | 2 | Vanderwolf et al. (2013a) |
| *Sordariomycetes* | *Hypocreales* | *Incertae sedis* | *Paramyrothecium roridum* | *Myrothecium roridum* | Bat guano, wood, soil-sediment, earthworm casts | Belgium, Slovakia | 2 | Vanderwolf et al. (2013a) |
| *Sordariomycetes* | *Hypocreales* | *Incertae sedis* | *Phaeostilbella spp.* | *Phaeostilbella* spp. | Dung, frass (not bat); sediment | China, France | 2 | Vanderwolf et al. (2013a) |
| *Sordariomycetes* | *Hypocreales* | *Incertae sedis* | *Sarocladium bacillisporum* | *Acremonium bacillisporum* | Insects, soil-sediment | Czech Republic, Korea | 2 | Vanderwolf et al. (2013a) |
| *Sordariomycetes* | *Hypocreales* | *Incertae sedis* | *Sarocladium bacillisporum* | *Sarocladium bacillisporum* | Unreported | Czech Republic, Korea | 2 | Vanderwolf et al. (2013a) |
| *Sordariomycetes* | *Hypocreales* | *Incertae sedis* | *Sarocladium bactrocephalum* | *Acremonium bactrocephalum* | Insects | Slovakia | 1 | Vanderwolf et al. (2013a) |
| *Sordariomycetes* | *Hypocreales* | *Incertae sedis* | *Sarocladium bactrocephalum* | *Sarocladium bactrocephalum* | Unreported | Slovakia | 1 | Vanderwolf et al. (2013a) |
| *Sordariomycetes* | *Hypocreales* | *Incertae sedis* | *Sarocladium glaucum* | *Acremonium glaucum* | Cave wall | Israel | 1 | Vanderwolf et al. (2013a) |
| *Sordariomycetes* | *Hypocreales* | *Incertae sedis* | *Sarocladium glaucum* | *Sarocladium glaucum* | Cave wall | Israel | 1 | Vanderwolf et al. (2013a) |
| *Sordariomycetes* | *Hypocreales* | *Incertae sedis* | *Sarocladium implicatum* | *Acremonium implicatum* | Air | China | 1 | Vanderwolf et al. (2013a) |
| *Sordariomycetes* | *Hypocreales* | *Incertae sedis* | *Sarocladium implicatum* | *Sarocladium implicatum* | Air | China | 1 | Vanderwolf et al. (2013a) |
| *Sordariomycetes* | *Hypocreales* | *Incertae sedis* | *Sarocladium kiliense* | *Sarocladium kiliense* | Air, soil-sidement | China, Czech Republic, Russia | 3 | Vanderwolf et al. (2013a) |
| *Sordariomycetes* | *Hypocreales* | *Incertae sedis* | *Sarocladium* sp. | *Sarocladium* sp. | Rock, soil-sediment, soil | China, Thailand | 1 | Jiang et al. (2017a, b); Suetrong et al. (2023) |
| *Sordariomycetes* | *Hypocreales* | *Incertae sedis* | *Sarocladium strictum* | *Acremonium strictum* | Air, soil-sediment, wood, other, earthworm casts, cave wall | Czech Republic, Israel, Japan, Poland, Slovakia, Spain, UK, USA | 8 | Vanderwolf et al. (2013a) |
| *Sordariomycetes* | *Hypocreales* | *Incertae sedis* | *Sarocladium strictum* | *Cephalosporium acremonium* | Air, soil-sediment, wood, other, cave wall | Belgium, Italy, Poland, Romania, Russia | 5 | Vanderwolf et al. (2013a) |
| *Sordariomycetes* | *Hypocreales* | *Incertae sedis* | *Sarocladium strictum* | *Sarocladium strictum* | Air , rock, soil-sediment, wood, Animal bone | China, Czech Republic, Israel, Japan, Poland, Slovakia, Spain, UK, USA | 10 | Vanderwolf et al. (2013a); Kokurewicz et al. (2016); Zhang et al. (2021) |
| *Sordariomycetes* | *Hypocreales* | *Incertae sedis* | *Sarocladium terricola* | *Sarocladium terricola* | Air | Brazil | 1 | Cunha et al. (2020) |
| *Sordariomycetes* | *Hypocreales* | *Incertae sedis* | *Sarocladium zeae* | *Sarocladium zeae* | Unreported | Bulgaria | 1 | Mitova et al. (2017) |
| *Sordariomycetes* | *Hypocreales* | *Incertae sedis* | *Trichothecium crotocinigenum* | *Trichothecium crotocinigenum* | Unreported | China | 1 | Zhang et al. (2021) |
| *Sordariomycetes* | *Hypocreales* | *Incertae sedis* | *Trichothecium roseum* | *Trichothecium roseum* | Soil-sediment, insect, wood | Czech Republic, Italy, Poland, Romania | 4 | Vanderwolf et al. (2013a); Pusz et al. (2018b) |
| *Sordariomycetes* | *Hypocreales* | *Nectriaceae* | *Aphanocladium album* | *Aphanocladium album* | Air, cave sediment, cave wall, insects | Canada, Czech Republic, Romania | 3 | Vanderwolf et al. (2013, 2019); Nováková et al. (2018) |
| *Sordariomycetes* | *Hypocreales* | *Nectriaceae* | *Aphanocladium* spp. | *Aphanocladium* spp. | Cave wall | Canada, USA | 2 | Zhang et al. (2014); Vanderwolf et al. (2019) |
| *Sordariomycetes* | *Hypocreales* | *Nectriaceae* | *Bisifusarium delphinoides* | *Bisifusarium delphinoides* | Unreported | China | 1 | Zhang et al. (2021) |
| *Sordariomycetes* | *Hypocreales* | *Nectriaceae* | *Bisifusarium dimerum* | *Bisifusarium dimerum* | Dead bat fur, skin | Italy | 1 | Vanderwolf et al. (2013a) |
| *Sordariomycetes* | *Hypocreales* | *Nectriaceae* | *Calonectria morganii* | *Cylindrocladium scoparium* | Soil-sediment | Russia | 1 | Vanderwolf et al. (2013a) |
| *Sordariomycetes* | *Hypocreales* | *Nectriaceae* | *Calonectria* sp. | *Calonectria* sp. | Organic litter, | Thailand | 1 | Suetrong et al. (2023) |
| *Sordariomycetes* | *Hypocreales* | *Nectriaceae* | *Cosmospora berkeleyana* | *Cosmospora berkeleyana* | Air, bat guano, insect, cave wall | Canada, Korea, Slovakia | 3 | Vanderwolf et al. (2013a) |
| *Sordariomycetes* | *Hypocreales* | *Nectriaceae* | *Cosmospora butyri* | *Cosmospora butyri* | soil-sediment | Russia | 1 | Vanderwolf et al. (2013a) |
| *Sordariomycetes* | *Hypocreales* | *Nectriaceae* | *Cosmospora diminuta* | *Cosmospora diminuta* | Unreported | China | 1 | Zhang et al. (2021) |
| *Sordariomycetes* | *Hypocreales* | *Nectriaceae* | *Cosmospora* spp. | *Cosmospora* spp. | Air, cave wall | China, France | 2 | Vanderwolf et al. (2013a); Jiang et al. (2017a, b) |
| *Sordariomycetes* | *Hypocreales* | *Nectriaceae* | *Cosmospora viridescens* | *Cosmospora viridescens* | Slate precipitate, wood | Germany, USA | 2 | Burow et al. (2019); Held et al. (2020) |
| *Sordariomycetes* | *Hypocreales* | *Nectriaceae* | *Cylindrocarpon didymum* | *Cylindrocarpon didymum* | Air, dead isopod bodies, soil, sediment | Czech Republic, Romania | 2 | Vanderwolf et al. (2013a); Nováková et al. (2018) |
| *Sordariomycetes* | *Hypocreales* | *Nectriaceae* | *Cylindrocarpon olidum* | *Cylindrocarpon olidum* | Air, soil | China | 1 | Jiang et al. (2017) |
| *Sordariomycetes* | *Hypocreales* | *Nectriaceae* | *Cylindrocarpon spp.* | *Cylindrocarpon spp.* | Earthworm casts, insects, soil-sediment | Canada, China, Japan, Slovakia, Slovenia | 5 | Vanderwolf et al. (2013, 2019); Zhang et al. (2017) |
| *Sordariomycetes* | *Hypocreales* | [*Nectriaceae*](https://www.indexfungorum.org/Names/families.asp?FamilyName=Nectriaceae) | *Cylindrocladiella lanceolata* | *Cylindrocladiella lanceolata* | Unreported | China | 1 | Zhang et al. (2021) |
| *Sordariomycetes* | *Hypocreales* | [*Nectriaceae*](https://www.indexfungorum.org/Names/families.asp?FamilyName=Nectriaceae) | *Cylindrocladiella stellenboschensis* | *Cylindrocladiella stellenboschensis* | Unreported | China | 1 | Zhang et al. (2021) |
| *Sordariomycetes* | *Hypocreales* | *Nectriaceae* | *Cylindrodendrum album* | *Cylindrodendrum album* | Unreported | Canada | 1 | Vanderwolf et al. (2013a) |
| *Sordariomycetes* | *Hypocreales* | *Nectriaceae* | *Cylindrodendrum alicantinum* | *Cylindrodendrum alicantinum* | Unreported | China | 1 | Zhang et al. (2021) |
| *Sordariomycetes* | *Hypocreales* | [*Nectriaceae*](https://www.indexfungorum.org/Names/families.asp?FamilyName=Nectriaceae) | *Dactylonectria macrodidyma* | *Dactylonectria macrodidyma* | Soil, sediment | Australia | 1 | Vanderwolf et al. (2013a) |
| *Sordariomycetes* | *Hypocreales* | *Nectriaceae* | *Dactylonectria macrodidyma* | *Ilyonectria macrodidyma* | Soil-sediment | Australia | 1 | Vanderwolf et al. (2013a) |
| *Sordariomycetes* | *Hypocreales* | *Nectriaceae* | *Dialonectria* sp. | *Dialonectria* sp. | Unreported | China | 1 | Zhang et al. (2021) |
| *Sordariomycetes* | *Hypocreales* | *Nectriaceae* | *Fusarium asiaticum* | *Fusarium asiaticum* | Cave wall, limestone | China | 1 | Jiang et al. (2017a, b) |
| *Sordariomycetes* | *Hypocreales* | *Nectriaceae* | *Fusarium avenaceum* | *Fusarium avenaceum* | Dung, frass (not bat); soil-sediment | Korea, Spain | 2 | Vanderwolf et al. (2013a); Ogórek et al. (2014b) |
| *Sordariomycetes* | *Hypocreales* | *Nectriaceae* | *Fusarium caeruleum* | *Fusarium caeruleum* | Soil-sediment | Thailand | 1 | Vanderwolf et al. (2013a) |
| *Sordariomycetes* | *Hypocreales* | *Nectriaceae* | *Fusarium chlamydosporum* | *Fusarium chlamydosporum* | Soil, sediment | China, Czech Republic, Indonesia, Romania, Thailand | 5 | Vanderwolf et al. (2013a); Nováková et al. (2018) |
| *Sordariomycetes* | *Hypocreales* | *Nectriaceae* | *Fusarium culmorum* | *Fusarium culmorum* | Soil-sediment | Bahamas, Poland | 1 | Vanderwolf et al. (2013a) |
| *Sordariomycetes* | *Hypocreales* | *Nectriaceae* | *Fusarium equiseti* | *Fusarium equiseti* | Air | Brazil, China, Italy, Poland, Slovakia | 5 | Vanderwolf et al. (2013a); Ogórek et al. (2014c, 2016c); |
| *Sordariomycetes* | *Hypocreales* | *Nectriaceae* | *Fusarium fujikuroi* | *Fusarium fujikuroi* | Unreported | China | 1 | Zhang et al. (2017) |
| *Sordariomycetes* | *Hypocreales* | *Nectriaceae* | *Fusarium fujikuroi* | *Fusarium subglutinans* | Cave wall, soil-sediment | Czech Republic, Japan, Thailand | 3 | Vanderwolf et al. (2013a) |
| *Sordariomycetes* | *Hypocreales* | *Nectriaceae* | *Fusarium fujikuroi* | *Fusarium verticillioides* | Cave wall, soil-sediment, other | Czech Republic, Poland, Russia, USA | 4 | Vanderwolf et al. (2013a) |
| *Sordariomycetes* | *Hypocreales* | *Nectriaceae* | *Fusarium graminearum* | *Fusarium graminearum* | Cave wall, limestone, rock | China, Poland | 2 | Ogórek et al. (2013); Jiang et al. (2017a, b); Zhang et al. (2017) |
| *Sordariomycetes* | *Hypocreales* | *Nectriaceae* | *Fusarium incarnatum* | *Fusarium incarnatum* | Air, bat carcasses, bat guano | China, France, South Africa | 3 | Vanderwolf et al. (2013a); Karunarathna et al. (2020) |
| *Sordariomycetes* | *Hypocreales* | *Nectriaceae* | *Fusarium lateritium* | *Fusarium lateritium* | Air, soil, sediment | China, Japan, Russia, Slovakia | 4 | Vanderwolf et al. (2013a); Ogórek (2018b) |
| *Sordariomycetes* | *Hypocreales* | *Nectriaceae* | *Fusarium merismoides* | *Fusarium merismoides* | Unreported | China | 1 | Zhang et al. (2017) |
| *Sordariomycetes* | *Hypocreales* | *Nectriaceae* | *Fusarium oxysporum* | *Fusarium oxysporum* | Air, soil-sediment, bat guano, wood, cave wall, water | Australia, Brazil, China, Czech Republic, France, Japan, Poland, Puerto Rico, Romania, Spain, USA | 11 | Taylor et al. (2013); Vanderwolf et al. (2013a); Jiang et al. (2017) |
| *Sordariomycetes* | *Hypocreales* | *Nectriaceae* | *Fusarium oxysporum complex* | *Fusarium oxysporum complex* | Air | Poland | 1 | Kokurewicz et al. (2016) |
| *Sordariomycetes* | *Hypocreales* | *Nectriaceae* | *Fusarium oxysporum* f.sp. *conglutinans* | *Fusarium oxysporum* f.sp. *conglutinans* | Soil-sediment | Bahamas | 1 | Vanderwolf et al. (2013a) |
| *Sordariomycetes* | *Hypocreales* | *Nectriaceae* | *Fusarium poae* | *Fusarium poae* | Unreported | Poland | 1 | Ogórek et al. (2014c) |
| *Sordariomycetes* | *Hypocreales* | *Nectriaceae* | *Fusarium proliferatum* | *Fusarium proliferatum* | Air, rock | China, Poland | 2 | Vanderwolf et al. (2013a) |
| *Sordariomycetes* | *Hypocreales* | *Nectriaceae* | *Fusarium sacchari* | *Fusarium sacchari* | Unreported | China | 1 | Zhang et al. (2021) |
| *Sordariomycetes* | *Hypocreales* | *Nectriaceae* | *Fusarium sambucinum* | *Fusarium roseum* | Unreported | China | 1 | Zhang et al. (2017); Zhang et al. (2021) |
| *Sordariomycetes* | *Hypocreales* | *Nectriaceae* | *Fusarium sambucinum* | *Fusarium sambucinum* | Animal bone | Poland | 1 | Zhang et al. (2017); Zhang et al. 2021 |
| *Sordariomycetes* | *Hypocreales* | *Nectriaceae* | *Fusarium sambucinum* | *Fusarium sambucinum var. sambucinum* | Soil-sediment | Russia | 1 | Vanderwolf et al. (2013a) |
| *Sordariomycetes* | *Hypocreales* | *Nectriaceae* | *Fusarium solani* | *Fusarium javanicum* | Wood | Czech Republic | 1 | Vanderwolf et al. (2013a) |
| *Sordariomycetes* | *Hypocreales* | *Nectriaceae* | *Fusarium solani* | *Fusarium solani* | Air, bat guano, cave wall, limestone, other, soil-sediment, wood | Brazil, China, Czech Republic, France, Japan, Poland, Puerto Rico, Romania, Russia, Slovakia, Spain, Thailand, USA | 13 | Vanderwolf et al. (2013a); Taylor et al. (2013, 2014); Jiang et al. (2017); Man (2017) |
| *Sordariomycetes* | *Hypocreales* | *Nectriaceae* | *Fusarium* sp. F. *fujikuroi complex* | *Fusarium* sp. F. *fujikuroi complex* | Bat | Brazil | 1 | Cunha et al. (2020) |
| *Sordariomycetes* | *Hypocreales* | *Nectriaceae* | *Fusarium sporotrichioides* | *Fusarium sporotrichioides* | Air, soil-sediment | Poland, Slovakia | 2 | Vanderwolf et al. (2013a) |
| *Sordariomycetes* | *Hypocreales* | *Nectriaceae* | *Fusarium* spp. | *Fusarium* spp. | Air, unreported, soil-sediment, water, bat guano, cave wall, wood, dung, frass (not bat); other, insects, earthworm casts | Australia, Bahamas, Brazil, Canada, China, Czech Republic, France, Greece, India, Indonesia, Italy, Malaysia, Mexico, Poland, Puerto Rico, Romania, Russia, Slovakia, Slovenia, South Africa, Spain, Thailand, Ukraine, UK, USA, Venezuela | 26 | Vanderwolf et al. (2013, 2015, 2019); Taylor et al. (2014); Zhang et al. (2021) |
| *Sordariomycetes* | *Hypocreales* | *Nectriaceae* | *Fusarium thapsinum* | *Fusarium thapsinum* | Water | China | 1 | Jiang et al. (2017) |
| *Sordariomycetes* | *Hypocreales* | *Nectriaceae* | *Fusarium tricinctum* | *Fusarium tricinctum* | Soil-sediment | China, Spain | 2 | Vanderwolf et al. (2013a); Zhang et al. (2021) |
| *Sordariomycetes* | *Hypocreales* | *Nectriaceae* | *Fusarium udum* | *Fusarium cf. udum* | Air | Czech Republic | 1 | Vanderwolf et al. (2013a) |
| *Sordariomycetes* | *Hypocreales* | *Nectriaceae* | *Fusicolla matuoi* | *Fusicolla matuoi* | Unreported | China | 1 | Zhang et al. (2021) |
| *Sordariomycetes* | *Hypocreales* | *Nectriaceae* | *Fusicolla merismoides* | *Fusicolla merismoides* | Soil-sediment | Czech Republic | 1 | Vanderwolf et al. (2013a) |
| *Sordariomycetes* | *Hypocreales* | *Nectriaceae* | *Fusicolla septimanifiniscientiae* | *Fusicolla septimanifiniscientiae* | Soil, sediment | Iran | 1 | Savković et al. (2025) |
| *Sordariomycetes* | *Hypocreales* | *Nectriaceae* | *Gliocephalotrichum simplex* | *Gliocephalotrichum simplex* | Soil-sediment | Russia | 1 | Vanderwolf et al. (2013a) |
| *Sordariomycetes* | *Hypocreales* | *Nectriaceae* | *Gliocladiopsis* sp. | *Gliocladiopsis* sp. | Unreported | China | 1 | Zhang et al. (2021) |
| *Sordariomycetes* | *Hypocreales* | *Nectriaceae* | *Hydropisphaera* sp. | *Hydropisphaera* sp. | Cave wall | USA | 1 | Vanderwolf et al. (2013a) |
| *Sordariomycetes* | *Hypocreales* | *Nectriaceae* | *Ilyonectria destructans* | *Cylindrocarpon destructans* | Earthworm casts, insects, soil-sediment | Canada, Slovakia, Slovenia, UK | 4 | Vanderwolf et al. (2013a) |
| *Sordariomycetes* | *Hypocreales* | *Nectriaceae* | *Ilyonectria destructans* | *Ilyonectria destructans* | Earthworm casts, soil-sediment, insec | Canada, China, Slovakia, Slovenia, UK | 5 | Vanderwolf et al. (2013a) |
| *Sordariomycetes* | *Hypocreales* | *Nectriaceae* | *Ilyonectria destructans* | *Ilyonectria radicicola* | Unreported | China | 1 | Zhang et al. (2021) |
| *Sordariomycetes* | *Hypocreales* | *Nectriaceae* | *Ilyonectria robusta* | *Ilyonectria robusta* | Unreported | China | 1 | Zhang et al. (2017) |
| *Sordariomycetes* | *Hypocreales* | *Nectriaceae* | *Ilyonectria* sp. | *Ilyonectria* sp. | Unreported | China | 1 | Zhang et al. (2017) |
| *Sordariomycetes* | *Hypocreales* | *Nectriaceae* | *Luteonectria nematophila* | *FUSArium nematophilum* | Cave wall, limestone | China | 1 | Jiang et al. (2017a, b) |
| *Sordariomycetes* | *Hypocreales* | *Nectriaceae* | *Mariannaea camptospora* | *Mariannaea camptospora* | Forest soil, wood | Romania, UK, USA | 3 | Vanderwolf et al. (2013a); Held et al. (2020) |
| *Sordariomycetes* | *Hypocreales* | *Nectriaceae* | *Mariannaea elegans* | *Mariannaea elegans* | wood, soil-sediment | China, Japan, Romania | 3 | Vanderwolf et al. (2013a) |
| *Sordariomycetes* | *Hypocreales* | *Nectriaceae* | *Mariannaea elegans* var. *elegans* | *Mariannaea elegans* var. *elegans* | Wood, soil-sediment | Japan, Romania | 2 | Vanderwolf et al. (2013a) |
| *Sordariomycetes* | *Hypocreales* | *Nectriaceae* | *Mariannaea nipponica* | *Mariannaea nipponica* | Insects | Slovenia | 1 | Vanderwolf et al. (2013a) |
| *Sordariomycetes* | *Hypocreales* | *Nectriaceae* | *Mariannaea* sp. | *Mariannaea* sp. | Organic litter, | Thailand | 1 | Suetrong et al. (2023) |
| *Sordariomycetes* | *Hypocreales* | *Nectriaceae* | *Microdochium nivale* | *FUSArium nivale* | Wood | UK | 1 | Vanderwolf et al. (2013a) |
| *Sordariomycetes* | *Hypocreales* | *Nectriaceae* | *Nectria ellisii* | *Nectria ellisii* | soil-sediment | Japan | 1 | Vanderwolf et al. (2013a) |
| *Sordariomycetes* | *Hypocreales* | *Nectriaceae* | *Nectria pseudotrichia* | *Nectria pseudotrichia* | Limestone | China | 1 | Jiang et al. (2017) |
| *Sordariomycetes* | *Hypocreales* | *Nectriaceae* | *Nectria ramulariae* | *Nectria ramulariae* | Bat | USA | 1 | Zhang et al. (2014) |
| *Sordariomycetes* | *Hypocreales* | *Nectriaceae* | *Nectria* spp. | *Nectria* spp. | Soil-sediment, wood | China, Germany, USA | 3 | Vanderwolf et al. (2013a) |
| *Sordariomycetes* | *Hypocreales* | *Nectriaceae* | Nectriaceae sp. | Nectriaceae sp. | Soil-sediment, | Thailand | 1 | Suetrong et al. (2023) |
| *Sordariomycetes* | *Hypocreales* | *Nectriaceae* | *Neocosmospora ipomoeae* | *Neocosmospora ipomoeae* | Unreported | China | 1 | Zhang et al. (2021) |
| *Sordariomycetes* | *Hypocreales* | *Nectriaceae* | *Neocosmospora pallidimors* | *Neocosmospora pallidimors* | Bat carcasses | China | 1 | Karunarathna et al. (2020) |
| *Sordariomycetes* | *Hypocreales* | *Nectriaceae* | Neocosmospora sp. | Neocosmospora sp. | Sediment | Brazil | 1 | Alves et al. (2022) |
| *Sordariomycetes* | *Hypocreales* | *Nectriaceae* | *Neonectria microconidia* | *Neonectria microconidia* | Cave wal | China | 1 | Ma et al. (2025) |
| *Sordariomycetes* | *Hypocreales* | *Nectriaceae* | *Neonectria obtusispora* | *Neonectria obtusispora* | Soil | Canada | 1 | Out et al. (2016) |
| *Sordariomycetes* | *Hypocreales* | *Nectriaceae* | *Neonectria punicea* | *Neonectria punicea* | Cave wal | China | 1 | Ma et al. (2025) |
| *Sordariomycetes* | *Hypocreales* | *Nectriaceae* | *Neonectria ramulariae* | *Cylindrocarpon obtusiusculum* | Cave sediment | Romania | 1 | Nováková et al. (2018) |
| *Sordariomycetes* | *Hypocreales* | *Nectriaceae* | Neonectria spp. | Neonectria spp. | Soil-sediment, wood | Australia, Germany, USA | 3 | Vanderwolf et al. (2013a) |
| *Sordariomycetes* | *Hypocreales* | *Nectriaceae* | *Paracremonium apiculatum* | *Paracremonium apiculatum* | Soil | China | 1 | zhang et al. (2021) |
| *Sordariomycetes* | *Hypocreales* | *Nectriaceae* | *Paracremonium ellipsoideum* | *Paracremonium ellipsoideum* | Sewage | China | 1 | zhang et al. (2021) |
| *Sordariomycetes* | *Hypocreales* | *Nectriaceae* | *Paracremonium* sp. | *Paracremonium* sp. | Unreported | China | 1 | Zhang et al. (2021) |
| *Sordariomycetes* | *Hypocreales* | *Nectriaceae* | *Paracremonium variiforme* | *Paracremonium variiforme* | Air, bat guano | China | 1 | Zhang et al. (2017) |
| *Sordariomycetes* | *Hypocreales* | *Nectriaceae* | *Paracremonium yunnanense* | *Paracremonium yunnanense* | Air | China | 1 | Liu et al. (2026) |
| *Sordariomycetes* | *Hypocreales* | *Nectriaceae* | *Paraphaeosphaeria hydei* | *Paraphaeosphaeria hydei* | Plant debris | China | 1 | zhang et al. (2021) |
| *Sordariomycetes* | *Hypocreales* | *Nectriaceae* | *Paraphaeosphaeria* spp. | *Paraphaeosphaeria* spp*.* | Air, organic litter, | Brazil, China, Thailand | 3 | Zhang (2019); Suetrong et al. (2023) |
| *Sordariomycetes* | *Hypocreales* | *Nectriaceae* | *Penicillifer diparietisporus* | *Penicillifer diparietisporus* | Unreported | China | 1 | Zhang et al. (2021) |
| *Sordariomycetes* | *Hypocreales* | *Nectriaceae* | *Pseudocosmospora rogersonii* | *Pseudocosmospora rogersonii* | Unreported | China | 1 | Zhang et al. (2021) |
| *Sordariomycetes* | *Hypocreales* | *Nectriaceae* | Pseudocosmospora sp. | *Pseudocosmospora roseus* | Unreported | Canada | 1 | Vanderwolf et al. (2013, 2015, 2019) |
| *Sordariomycetes* | *Hypocreales* | *Nectriaceae* | *Scolecofusarium ciliatum* | *Volutella ciliata* | Air, soil-sediment, water | China, Japan | 2 | Vanderwolf et al. (2013a); Jiang et al. (2017) |
| *Sordariomycetes* | *Hypocreales* | *Nectriaceae* | *Thelonectria discophora* | *Thelonectria discophora* | Limestone | China | 1 | Jiang et al. (2017) |
| *Sordariomycetes* | *Hypocreales* | *Nectriaceae* | *Thelonectria olida* | *Thelonectria olida* | Unreported | China | 1 | Zhang et al. (2021) |
| *Sordariomycetes* | *Hypocreales* | *Nectriaceae* | *Thelonectria* sp. | *Thelonectria* sp. | Air, Organic litter, | China, Thailand | 2 | Zhang et al. (2021); Suetrong et al. (2023) |
| *Sordariomycetes* | *Hypocreales* | *Nectriaceae* | *Tubercularia* spp. | *Tubercularia* spp. | Unreported | Canada | 1 | Vanderwolf et al. (2013a) |
| *Sordariomycetes* | *Hypocreales* | *Nectriaceae* | *Volutella aeria* | *Volutella aeria* | Air | China | 1 | Zhang et al. (2017) |
| *Sordariomycetes* | *Hypocreales* | *Nectriaceae* | *Volutella citrinella* | *Volutella citrinella* | Sediment | China, France | 2 | Zhang et al. (2021) |
| *Sordariomycetes* | *Hypocreales* | *Nectriaceae* | *Volutella roseola* | *Volutella roseola* | Soil-sediment, wood | France | 1 | Vanderwolf et al. (2013a) |
| *Sordariomycetes* | *Hypocreales* | *Nectriaceae* | *Volutella* spp. | *Volutella* spp. | Insects, Organic litter, air, | Slovenia, Thailand | 2 | Vanderwolf et al. (2013a) |
| *Sordariomycetes* | *Hypocreales* | *Nectriaceae* | *Volutellonectria consors* | *Cosmospora consors* | Soil-sediment | Spain | 1 | Vanderwolf et al. (2013a) |
| *Sordariomycetes* | *Hypocreales* | *Nectriaceae* | *Volutellonectria consors* | *Volutellonectria consors* | Soil-sediment | China, Spain | 2 | Vanderwolf et al. (2013a) |
| *Sordariomycetes* | *Hypocreales* | *Niessliaceae* | *Monocillium granulatum* | *Monocillium granulatum* | Other, cave wall | Czech Republic | 1 | Vanderwolf et al. (2013a) |
| *Sordariomycetes* | *Hypocreales* | *Niessliaceae* | *Monocillium* sp. | *Monocillium* sp. | Unreported, soil-sediment, cave wall | USA | 1 | Vanderwolf et al. (2013a) |
| *Sordariomycetes* | *Hypocreales* | *Niessliaceae* | *Niesslia indica* | *Niesslia indica* | Air | Spain | 1 | Savković et al. (2025) |
| *Sordariomycetes* | *Hypocreales* | *Niessliaceae* | Niesslia sp. | Hyaloseta sp. | Unreported | China | 1 | Zhang et al. (2021) |
| *Sordariomycetes* | *Hypocreales* | *Niessliaceae* | *Niesslia tenuis* | *Niesslia tenuis* | Asymptomatic specimens of Proteus anguinus | Slovenia | 1 | Savković et al. (2025) |
| *Sordariomycetes* | *Hypocreales* | *Ophiocordycipitaceae* | *Hirsutella dipterigena* | *Hirsutella dipterigena* | Insects | Belgium, Netherlands, UK | 3 | Vanderwolf et al. (2013a) |
| *Sordariomycetes* | *Hypocreales* | *Ophiocordycipitaceae* | *Hirsutella guignardii* | *Hirsutella guignardii* | insects | Czech Republic, Germany | 2 | Vanderwolf et al. (2013a) |
| *Sordariomycetes* | *Hypocreales* | *Ophiocordycipitaceae* | *Hirsutella guignardii* | *Mahevia guignardii* | Insects | France | 1 | Vanderwolf et al. (2013a) |
| *Sordariomycetes* | *Hypocreales* | *Ophiocordycipitaceae* | *Hirsutella guignardii* | *Troglobiomyces guignardii* | Insects | Belgium, France, Italy | 3 | Vanderwolf et al. (2013a) |
| *Sordariomycetes* | *Hypocreales* | *Ophiocordycipitaceae* | *Hirsutella* spp. | *Hirsutella* spp. | Bat guano, insects, soil-sediment, unreported | Croatia, France, Puerto Rico, UK | 4 | Vanderwolf et al. (2013a) |
| *Sordariomycetes* | *Hypocreales* | *Ophiocordycipitaceae* | *Hymenostilbe sphingum* | *Cordyceps sphingum* | Insects, unreported | Algeria, Croatia, France | 3 | Vanderwolf et al. (2013a) |
| *Sordariomycetes* | *Hypocreales* | *Ophiocordycipitaceae* | *Ophiocordyceps entomorrhiza* | *Hirsutella eleutheratorum* | Insects | France, Spain | 2 | Vanderwolf et al. (2013a) |
| *Sordariomycetes* | *Hypocreales* | *Ophiocordycipitaceae* | *Ophiocordyceps entomorrhiza* | *Ophiocordyceps entomorrhiza* | Unreported | Unknow |  | Vanderwolf et al. (2013a) |
| *Sordariomycetes* | *Hypocreales* | *Ophiocordycipitaceae* | *Ophiocordyceps sinensis* | *Ophiocordyceps sinensis* | Unreported | China | 1 | Zhang (2019) |
| *Sordariomycetes* | *Hypocreales* | *Ophiocordycipitaceae* | *Ophiocordyceps sobolifera* | *Ophiocordyceps sobolifera* | Unreported | China | 1 | Zhang et al. (2021) |
| *Sordariomycetes* | *Hypocreales* | *Ophiocordycipitaceae* | *Pleurocordyceps agarica* | *Polycephalomyces agaricus* | Unreported | China | 1 | Zhang et al. (2021) |
| *Sordariomycetes* | *Hypocreales* | *Ophiocordycipitaceae* | *Polycephalomyces ramosus* | *Polycephalomyces ramosus* | Unreported, insects, cave wall | Germany, Netherlands, UK | 3 | Vanderwolf et al. (2013a) |
| *Sordariomycetes* | *Hypocreales* | *Ophiocordycipitaceae* | *Purpureocillium lavendulum* | *Purpureocillium lavendulum* | Air, limestone, rock surface | China | 1 | Jiang et al. (2017) |
| *Sordariomycetes* | *Hypocreales* | *Ophiocordycipitaceae* | *Purpureocillium lilacinum* | *Paecilomyces lilacinus* | Cave wall, soil-sediment, bat guano, insects, air, earthworm casts | China, Czech Republic, Italy, Poland, Puerto Rico, Slovakia, Slovenia, Spain, Thailand, USA | 10 | Vanderwolf et al. (2013a); Man (2017) |
| *Sordariomycetes* | *Hypocreales* | *Ophiocordycipitaceae* | *Purpureocillium lilacinum* | Purpureocillium cf. lilacinum | Bat | Brazil | 1 | Cunha et al. (2020) |
| *Sordariomycetes* | *Hypocreales* | *Ophiocordycipitaceae* | *Purpureocillium lilacinum* | *Purpureocillium lilacinum* | Air, bat guano, corroded cave walls, isopod feces, dead isopod bodies, dead spiders, sediment, speleothem, water, rock sufaces | Brazil, China, Malaysia, Romania, Spain | 5 | Wasti et al. (2021); Taylor et al. (2013, 2014); Zhang et al. (2021) |
| *Sordariomycetes* | *Hypocreales* | *Ophiocordycipitaceae* | *Purpureocillium* spp. | *Purpureocillium* spp. | Air | China, Thailand | 2 | Jiang et al. (2017a, b); Suetrong et al. (2023) |
| *Sordariomycetes* | *Hypocreales* | *Ophiocordycipitaceae* | *Synnematium* sp. | *Synnematium* sp. | Cave wall | UK | 1 | Vanderwolf et al. (2013a) |
| *Sordariomycetes* | *Hypocreales* | *Ophiocordycipitaceae* | *Tolypocladium album* | *Tolypocladium album* | Unreported | China | 1 | Zhang et al. (2021) |
| *Sordariomycetes* | *Hypocreales* | *Ophiocordycipitaceae* | *Tolypocladium cylindrosporum* | *Tolypocladium cylindrosporum* | Cave wall, soil-sediment | Belgium, China, France, Slovakia | 4 | Vanderwolf et al. (2013a) |
| *Sordariomycetes* | *Hypocreales* | *Ophiocordycipitaceae* | *Tolypocladium inflatum* | *Tolypocladium inflatum* | Bat guano, earthworm casts | Canada, Slovakia | 2 | Vanderwolf et al. (2013a) |
| *Sordariomycetes* | *Hypocreales* | *Ophiocordycipitaceae* | *Tolypocladium* sp. | *Tolypocladium* sp. | Soil-sediment, unreported | China, New Guinea, New Zealand, USA | 4 | Vanderwolf et al. (2013, 2019); Man et al. (2015); Man (2017); Zhang et al. (2017) |
| *Sordariomycetes* | *Hypocreales* | *Pseudodiploosporaceae* | *Pseudodiploospora longispora* | *Pseudodiploospora longispora* | Porcupine dung | Unknow |  | Savković et al. (2025) |
| *Sordariomycetes* | *Hypocreales* | *Stachybotryaceae* | *Achroiostachys* sp. | *Achroiostachys* sp. | Soil-sediment, | Thailand | 1 | Suetrong et al. (2023) |
| *Sordariomycetes* | *Hypocreales* | *Stachybotryaceae* | *Albifimbria* sp. | *Albifimbria* sp. | Air | France | 1 | Leplat et al. (2018) |
| *Sordariomycetes* | *Hypocreales* | *Stachybotryaceae* | *Albifimbria verrucaria* | *Albifimbria verrucaria* | Sediment, soil, cave wall | Italy, Kazakhstan, Romania, Slovakia | 4 | Vanderwolf et al. (2013a); Nováková et al.(2018) |
| *Sordariomycetes* | *Hypocreales* | *Stachybotryaceae* | *Inaequalispora prestonii* | *Inaequalispora prestonii* | Unreported | China | 1 | Zhang (2019) |
| *Sordariomycetes* | *Hypocreales* | *Stachybotryaceae* | *Inaequalispora* sp. | *Inaequalispora* sp. | Unreported | China | 1 | Zhang (2019) |
| *Sordariomycetes* | *Hypocreales* | *Stachybotryaceae* | *Memnoniella* sp. | *Memnoniella* sp. | Unreported | China | 1 | Zhang et al. (2021) |
| *Sordariomycetes* | *Hypocreales* | *Stachybotryaceae* | *Myxospora* sp. | *Myxospora* sp. | Organic litter, | Thailand | 1 | Suetrong et al. (2023) |
| *Sordariomycetes* | *Hypocreales* | *Stachybotryaceae* | *Paramyrothecium roridum* | *Paramyrothecium roridum* | Wood, soil-sediment, bat guano, earthworm casts | Belgium, China, Italy, Slovakia | 4 | Vanderwolf et al. (2013a); Zhang et al. (2021) |
| *Sordariomycetes* | *Hypocreales* | *Stachybotryaceae* | *Sirastachys longispora* | *Sirastachys longispora* | Unreported | China | 1 | Zhang et al. (2017) |
| *Sordariomycetes* | *Hypocreales* | *Stachybotryaceae* | *Sirastachys phaeospora* | *Sirastachys phaeospora* | Unreported | China | 1 | Zhang et al. (2021) |
| *Sordariomycetes* | *Hypocreales* | *Stachybotryaceae* | *Stachybotrys chartarum* | *Stachybotrys chartarum* | Air, other, cave wall, soil-sediment, wood | China, Czech Republic, Israel, Japan, Poland, Romania, Slovakia, South Africa | 8 | Vanderwolf et al. (2013a); Zhang et al. (2017); Nováková et al. (2018) |
| *Sordariomycetes* | *Hypocreales* | *Stachybotryaceae* | *Stachybotrys chlorohalonatus* | *Stachybotrys chlorohalonata* | Air | South Africa | 1 | Vanderwolf et al. (2013a) |
| *Sordariomycetes* | *Hypocreales* | *Stachybotryaceae* | *Stachybotrys chlorohalonatus* | *Stachybotrys chlorohalonatus* | Air | China, South Africa | 2 | Zhang et al. (2021) |
| *Sordariomycetes* | *Hypocreales* | *Stachybotryaceae* | *Stachybotrys cylindrosporus* | *Stachybotrys cylindrospora* | Soil-sediment | Japan, Slovakia | 2 | Vanderwolf et al. (2013a) |
| *Sordariomycetes* | *Hypocreales* | *Stachybotryaceae* | *Stachybotrys cylindrosporus* | *Stachybotrys cylindrosporus* | Soil-sediment | Japan, Poland, Slovakia | 3 | Vanderwolf et al. (2013a); Pusz et al. (2017) |
| *Sordariomycetes* | *Hypocreales* | *Stachybotryaceae* | *Stachybotrys echinatus* | *Memnoniella echinata* | Air | South Africa | 1 | Vanderwolf et al. (2013a) |
| *Sordariomycetes* | *Hypocreales* | *Stachybotryaceae* | *Stachybotrys echinatus* | *Stachybotrys echinatus* | Unreported | Unknow |  | Zhang et al. (2021) |
| *Sordariomycetes* | *Hypocreales* | *Stachybotryaceae* | *Stachybotrys parvisporus* | *Stachybotrys parvisporus* | Unreported | China | 1 | Zhnag et al. 2021 |
| *Sordariomycetes* | *Hypocreales* | *Stachybotryaceae* | *Stachybotrys* sp. | *Stachybotrys* sp. | Air, soil-sediment, cave wall | Czech Republic, France, Russia, Spain, USA | 5 | Vanderwolf et al. (2013a); Leplat et al. (2018) |
| *Sordariomycetes* | *Hypocreales* | *Stachybotryaceae* | *Striatibotrys eucylindrosporus* | *Striatibotrys eucylindrosporus* | Unreported | China | 1 | Zhang et al. (2021) |
| *Sordariomycetes* | *Hypocreales* | [*Stachybotryaceae*](https://www.indexfungorum.org/Names/families.asp?FamilyName=Stachybotryaceae) | *Xenosporium berkeleyi* | Xenomyrothecium spp. | Organic litter, | Thailand | 1 | Suetrong et al. (2023) |
| *Sordariomycetes* | *Hypocreales* | *Tilachlidiaceae* | *Tilachlidium* sp. | *Tilachlidium* sp. | Insects | UK | 1 | Vanderwolf et al. (2013a) |
| *Sordariomycetes* | *Hypocreales* |  | Hypocreales spp. | Hypocreales spp. | Airborne | China, France, Thailand | 3 | Man (2017); Suetrong et al. (2023) |
| *Sordariomycetes* | *Incertae sedis* | *Apiosporaceae* | *Arthrinium rasikravindrii* | *Arthrinium rasikravindrii* | Soil | China | 1 | Wang et al. (2018) |
| *Sordariomycetes* | *Incertae sedis* | *Apiosporaceae* | *Arthrinium sphaerospermum* | *Arthrinium sphaerospermum* | Air, rock, , cave wall, soil-sediment, earthworm casts | Canada, Czech Republic, Italy, Slovakia, USA | 5 | Vanderwolf et al. (2013a) |
| *Sordariomycetes* | *Incertae sedis* | *Apiosporaceae* | *Arthrinium* spp. | *Arthrinium* spp. | Air, cave wall, rock, soil-sediment | China, France, Japan, Spain, Thailand, USA | 6 | Vanderwolf et al. (2013a); Zhang et al. (2017); Leplat et al. (2018); Suetrong et al. (2023) |
| *Sordariomycetes* | *Incertae sedis* | *Incertae sedis* | *Nigrospora globosa* | *Nigrospora globosa* | Soil | China | 1 | zhang et al. (2021) |
| *Sordariomycetes* | *Incertae sedis* | *Incertae sedis* | *Nigrospora oryzae* | *Nigrospora oryzae* | Cave wall | China, Israel, Sri Lanka | 3 | Vanderwolf et al. (2013a) |
| *Sordariomycetes* | *Incertae sedis* | *Incertae sedis* | *Nigrospora oryzae* | *Nigrospora sphaerica* | Soil-sediment | Bahamas | 1 | Vanderwolf et al. (2013a) |
| *Sordariomycetes* | *Incertae sedis* | *Incertae sedis* | *Nigrospora* spp. | *Nigrospora* spp. | Air | Romania, Spain | 2 | Vanderwolf et al. (2013a); Bercea et al. (2018) |
| *Sordariomycetes* | *Incertae sedis* | *Incertae sedis* | *Stilbella* spp. | *Stilbella* spp. | Unreported | China, Croatia, Thailand | 3 | Vanderwolf et al. (2013a); Man et al. (2015); Man (2017); Suetrong et al. (2023) |
| *Sordariomycetes* | *Incertae sedis* | *Thyridiaceae* | *Thyridium* sp. | *Phialemoniopsis* spp. | Cave wall, limestone | China | 1 | Jiang et al. (2017a, b) |
| *Sordariomycetes* | *Lulworthiales* | *Lulworthiaceae* | Zalerion sp. | Zalerion sp. | Unreported | Canada | 1 | Vanderwolf et al. (2019) |
| *Sordariomycetes* | *Melanosporales* | *Ceratostomataceae* | *Harzia acremonioides* | *Harzia acremonioides* | Soil-sediment | Russia | 1 | Vanderwolf et al. (2013a) |
| *Sordariomycetes* | *Melanosporales* | *Ceratostomataceae* | *Melanospora* sp. | *Melanospora* sp. | Wood | France | 1 | Vanderwolf et al. (2013a) |
| *Sordariomycetes* | *Melanosporales* | *Ceratostomataceae* | *Melanospora zamiae* | *Melanospora zamiae* | Wood | Romania | 1 | Vanderwolf et al. (2013a) |
| *Sordariomycetes* | *Melanosporales* | *Ceratostomataceae* | *Microthecium fimicola* | *Sphaerodes fimicola* | Other, wood | Poland | 1 | Vanderwolf et al. (2013a) |
| *Sordariomycetes* | *Melanosporales* | *Ceratostomataceae* | *Papulaspora rubida* | *Papulaspora rubida* | soil-sediment, wood | Argentina, Romania, UK | 3 | Vanderwolf et al. (2013a) |
| *Sordariomycetes* | *Melanosporales* | *Ceratostomataceae* | Papulaspora sp. | Papulaspora sp. | Unreported, soil-sediment, air | USA | 1 | Vanderwolf et al. (2013a) |
| *Sordariomycetes* | *Microascales* | *Ceratocystidaceae* | *Ceratocystis autographa* | *Ceratocystis autographa* | Unreported | Canada | 1 | Vanderwolf et al. (2013a) |
| *Sordariomycetes* | *Microascales* | *Ceratocystidaceae* | *Ceratocystis* sp. | *Ceratocystis* sp. | Bat guano | France | 1 | Vanderwolf et al. (2013a) |
| *Sordariomycetes* | *Microascales* | *Ceratocystidaceae* | *Cercophora solaris* | *Cercophora solaris* | Unreported | China | 1 | Zhang et al. (2021) |
| *Sordariomycetes* | *Microascales* | *Ceratocystidaceae* | *Cercophora* sp. | *Cercophora* sp. | Unreported | China | 1 | Zhang et al. (2021) |
| *Sordariomycetes* | *Microascales* | *Ceratocystidaceae* | *Cercophora sparsa* | *Cercophora sparsa* | Unreported | China | 1 | Zhang et al. (2021) |
| *Dothideomycetes* | *Mycosphaerellales* | *Mycosphaerellaceae* | *Cercospora* spp. | *Cercospora* spp. | Air | Spain, Thailand | 2 | Vanderwolf et al. (2013a); Suetrong et al. (2023) |
| *Dothideomycetes* | *Mycosphaerellales* | *Mycosphaerellaceae* | *Cercospora canescens* | *Cercospora cf. canescens* | Air | Brazil | 1 | Alves et al. (2022) |
| *Dothideomycetes* | *Mycosphaerellales* | *Mycosphaerellaceae* | *Cercospora vignigena* | *Cercospora vignigena* | Air | Brazil | 1 | Alves et al. (2022) |
| *Sordariomycetes* | *Microascales* | *Ceratocystidaceae* | *Chaetomella* sp. | *Chaetomella* sp. | Organic litter, | Thailand | 1 | Suetrong et al. (2023) |
| *Sordariomycetes* | *Microascales* | *Ceratocystidaceae* | *Parathielavia hyrcaniae* | *Thielavia hyrcaniae* | Earthworm casts | Slovakia | 1 | Vanderwolf et al. (2013a) |
| *Sordariomycetes* | *Microascales* | *Ceratocystidaceae* | *Pseudothielavia terricola* | *Thielavia terricola* | Cave wall | Israel | 1 | Vanderwolf et al. (2013a) |
| *Sordariomycetes* | *Microascales* | *Ceratocystidaceae* | *Thermothielavioides terrestris* | *Thielavia terrestris* | Insects | USA | 1 | Vanderwolf et al. (2013a) |
| *Sordariomycetes* | *Microascales* | *Ceratocystidaceae* | *Thielavia* sp. | *Thielavia* sp. | Dead bat fur, skin | China, France, Italy | 3 | Vanderwolf et al. (2013a); Zhang et al. (2017); Leplat et al. (2018) |
| *Sordariomycetes* | *Microascales* | *Ceratocystidaceae* | *Thielaviopsis* sp. | *Thielaviopsis* sp*.* | Soil-sediment, | Thailand | 1 | Suetrong et al. (2023) |
| *Sordariomycetes* | *Microascales* | *Incertae sedis* | *Cephalotrichiella penicillata* | *Cephalotrichiella penicillata* | Unreported | China | 1 | Zhang et al. (2021) |
| *Sordariomycetes* | *Microascales* | *Incertae sedis* | *Gabarnaudia* sp. | *Gabarnaudia* sp. | Unreported | France | 1 | Leplat et al. (2018) |
| *Sordariomycetes* | *Microascales* | *Microascaceae* | *Acaulium acremonium* | *Scopulariopsis acremonium* | Other | Czech Republic | 1 | Vanderwolf et al. (2013a) |
| *Sordariomycetes* | *Microascales* | *Microascaceae* | *Acaulium albonigrescens* | *Microascus albonigrescens* | Soil | USA | 1 | Vanderwolf et al. (2013a) |
| *Sordariomycetes* | *Microascales* | *Microascaceae* | *Acaulium caviariforme* | *Acaulium caviariforme* | Insects, soil | Belgium, Canada, USA | 3 | Vanderwolf et al. (2013a); Zhang et al. (2021) |
| *Sordariomycetes* | *Microascales* | *Microascaceae* | *Acaulium caviariforme* | *Microascus caviariformis* | Insects, other | Belgium, Canada | 2 | Vanderwolf et al. (2013a) |
| *Sordariomycetes* | *Microascales* | *Microascaceae* | *Cephalotrichum asperulum* | *Cephalotrichum asperulum* | Unreported | China | 1 | Zhang et al. (2021) |
| *Sordariomycetes* | *Microascales* | *Microascaceae* | *Cephalotrichum castaneum* | *Cephalotrichum castaneum* | Air | China, France | 2 | Vanderwolf et al. (2013a) |
| *Sordariomycetes* | *Microascales* | *Microascaceae* | *Cephalotrichum castaneum* | *Doratomyces castaneus* | Air | France | 1 | Vanderwolf et al. (2013a) |
| *Sordariomycetes* | *Microascales* | *Microascaceae* | *Cephalotrichum dendrocephalum* | *Cephalotrichum dendrocephalum* | Unreported | China | 1 | Pereira et al. (2022) |
| *Sordariomycetes* | *Microascales* | *Microascaceae* | *Cephalotrichum guizhouense* | *Cephalotrichum guizhouense* | Air, limestone | China | 1 | Jiang et al. (2017) |
| *Sordariomycetes* | *Microascales* | *Microascaceae* | *Cephalotrichum hinnuleum* | *Cephalotrichum hinnuleum* | Soil and sediment | Slovakia | 1 | Savković et al. (2025) |
| *Sordariomycetes* | *Microascales* | *Microascaceae* | *Cephalotrichum leave* | *Cephalotrichum leave* | Cave wall, limestone | China | 1 | Jiang et al. (2017) |
| *Sordariomycetes* | *Microascales* | *Microascaceae* | *Cephalotrichum lignatile* | *Cephalotrichum lignatile* | Timber | Belgium | 1 | Savković et al. (2025) |
| *Sordariomycetes* | *Microascales* | *Microascaceae* | *Cephalotrichum medium* | *Cephalotrichum medium* | Unreported | Unknow |  | Vanderwolf et al. (2013a) |
| *Sordariomycetes* | *Microascales* | *Microascaceae* | *Cephalotrichum medium* | *Stysanus medius* | Insects | Germany | 1 | Vanderwolf et al. (2013a) |
| *Sordariomycetes* | *Microascales* | *Microascaceae* | *Cephalotrichum microsporum* | *Cephalotrichum microsporum* | Timber | Belgium, China | 2 | Zhang et al. (2021) |
| *Sordariomycetes* | *Microascales* | *Microascaceae* | *Cephalotrichum microsporum* | *Doratomyces microsporus* | Cave wall, soil-sediment, air, insects | Bulgaria, Czech Republic, Slovakia, USA | 4 | Vanderwolf et al. (2013a) |
| *Sordariomycetes* | *Microascales* | *Microascaceae* | *Cephalotrichum nanum* | *Cephalotrichum nanum* | Soil | China, USA | 2 | Zhang et al. (2021) |
| *Sordariomycetes* | *Microascales* | *Microascaceae* | *Cephalotrichum nanum* | *Doratomyces nanus* | Air, bat guano, earthworm casts, insects, other, cave wall, soil-sediment | Czech Republic, Japan, Slovakia | 3 | Vanderwolf et al. (2013a) |
| *Sordariomycetes* | *Microascales* | *Microascaceae* | *Cephalotrichum oligotriphicum* | *Cephalotrichum oligotriphicum* | Limestone, soil | China | 1 | Jiang et al. (2017) |
| *Sordariomycetes* | *Microascales* | *Microascaceae* | *Cephalotrichum purpureofuscum* | *Cephalotrichum purpureofuscum* | Unreported | China | 1 | Zhang et al. (2021) |
| *Sordariomycetes* | *Microascales* | *Microascaceae* | *Cephalotrichum purpureofuscum* | *Doratomyces purpureofuscus* | Bat bones, rock sufaces, soil-sediment | China, Czech Republic | 2 | Vanderwolf et al. (2013a) |
| *Sordariomycetes* | *Microascales* | *Microascaceae* | *Cephalotrichum* sp. | *Stysanus* sp*.* | Air | Australia | 1 | Vanderwolf et al. (2013a) |
| *Sordariomycetes* | *Microascales* | *Microascaceae* | *Cephalotrichum* spp. | *Cephalotrichum* spp. | Air, cave wall, Organic litter, sediment | Canada, France, Thailand, USA | 4 | Vanderwolf et al. (2019); Suetrong et al. (2023) |
| *Sordariomycetes* | *Microascales* | *Microascaceae* | *Cephalotrichum stemonitis* | *Cephalotrichum stemonitis* | Air, dung, frass (not bat); microbial mat, soil-sediment, water, wood, cave wall, bat guano | Canada, China, Czech Republic, Japan, Korea, Poland, Romania, Russia, Slovakia, Spain, USA | 11 | Vanderwolf et al. (2013a) |
| *Sordariomycetes* | *Microascales* | *Microascaceae* | *Cephalotrichum stemonitis* | *Doratomyces stemonitis* | soil-sediment, bat guano, insects | Slovakia | 1 | Vanderwolf et al. (2013a) |
| *Sordariomycetes* | *Microascales* | *Microascaceae* | *Cephalotrichum verrucisporum* | *Cephalotrichum verrucisporum* | Rock surface, soil | China | 1 | Zhang et al. (2017) |
| *Sordariomycetes* | *Microascales* | *Microascaceae* | *Cephalotrichum* sp. | *Pycnostysanus* sp. | Soil-sediment | Bahamas | 1 | Vanderwolf et al. (2013a) |
| *Sordariomycetes* | *Microascales* | *Microascaceae* | *Doratomyces* spp. | *Doratomyces* spp. | Air, unreported, soil-sediment, cave wall, insects | Australia, Belgium, Bulgaria, France, USA | 5 | Vanderwolf et al. (2013a) |
| *Sordariomycetes* | *Microascales* | *Microascaceae* | *Echinobotryum parasitans* | *Echinobotryum parasitans* | Other | France | 1 | Vanderwolf et al. (2013a) |
| *Sordariomycetes* | *Microascales* | *Microascaceae* | *Echinobotryum* sp. | *Echinobotryum* sp. | Unreported | France | 1 | Vanderwolf et al. (2013a) |
| *Sordariomycetes* | *Microascales* | *Microascaceae* | *Echinobotryum subterraneum* | *Echinobotryum subterraneum* | Unreported | Unknow |  | Zhang et al. (2021) |
| *Sordariomycetes* | *Microascales* | *Microascaceae* | *Enterocarpus grenotii* | *Enterocarpus grenotii* | Soil | Belgium | 1 | Savković et al. (2025) |
| *Sordariomycetes* | *Microascales* | *Microascaceae* | *Graphium penicillioides* | *Graphium penicillioides* | Wood | China, Romania | 2 | Vanderwolf et al. (2013a); Zhang et al. (2021) |
| *Sordariomycetes* | *Microascales* | *Microascaceae* | *Kernia columnaris* | *Cephalotrichum columnare* | Unreported | China | 1 | Zhang et al. (2021) |
| *Sordariomycetes* | *Microascales* | *Microascaceae* | *Kernia columnaris* | *Kernia columnaris* | Unreported | China | 1 | Zhang (2019) |
| *Sordariomycetes* | *Microascales* | *Microascaceae* | *Kernia* spp. | *Kernia* spp. | Soil-sediment | China, USA | 2 | Vanderwolf et al. (2013a); Zhang et al. (2014, 2019) |
| *Sordariomycetes* | *Microascales* | *Microascaceae* | *Lophotrichus fimeti* | *Pseudallescheria fimeti* | Unreported | China | 1 | Zhang et al. (2017) |
| *Sordariomycetes* | *Microascales* | *Microascaceae* | *Microascaceae* sp. | *Microascaceae* sp. | Unreported | Thailand | 1 | Suetrong et al. (2023) |
| *Sordariomycetes* | *Microascales* | *Microascaceae* | *Microascus anfractus* | *Microascus anfractus* | Unreported | China | 1 | Zhang et al. (2017) |
| *Sordariomycetes* | *Microascales* | *Microascaceae* | *Microascus boulangeri* | *Microascus boulangeri* | Unreported | Canada | 1 | Vanderwolf et al. (2013a) |
| *Sordariomycetes* | *Microascales* | *Microascaceae* | *Microascus boulangeri* | *Petriella cf. boulangeri* | Unreported | Canada | 1 | Vanderwolf et al. (2013a) |
| *Sordariomycetes* | *Microascales* | *Microascaceae* | *Microascus chartarum* | *Microascus chartarum* | Soil-sediment | China, Slovakia | 2 | Vanderwolf et al. (2013a); Zhang et al. (2017) |
| *Sordariomycetes* | *Microascales* | *Microascaceae* | *Microascus chartarum* | *Scopulariopsis chartarum* | Soil-sediment | Slovakia | 1 | Vanderwolf et al. (2013a) |
| *Sordariomycetes* | *Microascales* | *Microascaceae* | *Microascus cirrosus* | *Microascus cirrosus* | Cave wall | France | 1 | Vanderwolf et al. (2013a) |
| *Sordariomycetes* | *Microascales* | *Microascaceae* | *Microascus collaris* | *Microascus collaris* | Plant debris | China | 1 | zhang et al. (2021) |
| *Sordariomycetes* | *Microascales* | *Microascaceae* | *Microascus croci* | *Microascus croci* | Air, bat guano | China, Slovakia, Spain | 3 | Vanderwolf et al. (2013a); Zhang et al. (2021) |
| *Sordariomycetes* | *Microascales* | *Microascaceae* | *Microascus croci* | *Scopulariopsis croci* | Bat guano | Slovakia | 1 | Vanderwolf et al. (2013a) |
| *Sordariomycetes* | *Microascales* | *Microascaceae* | *Microascus globulosus* | *Microascus globulosus* | Bat guano | China | 1 | Zhang et al. (2017),Zhang et al. (2021) |
| *Sordariomycetes* | *Microascales* | *Microascaceae* | *Microascus levis* | *Microascus levis* | Soil | China | 1 | zhang et al. (2021) |
| *Sordariomycetes* | *Microascales* | *Microascaceae* | *Microascus longirostris* | *Microascus longirostris* | Unreported | USA | 1 | Vanderwolf et al. (2013a) |
| *Sordariomycetes* | *Microascales* | *Microascaceae* | *Microascus murinus* | *Microascus murinus* | Unreported | China | 1 | Zhang et al. (2021) |
| *Sordariomycetes* | *Microascales* | *Microascaceae* | *Microascus paisii* | *Microascus paisii* | Unreported | China | 1 | Zhang et al. (2021) |
| *Sordariomycetes* | *Microascales* | *Microascaceae* | *Microascus sparsimycelialis* | *Microascus sparsimycelialis* | Soil | China | 1 | zhang et al. (2021) |
| *Sordariomycetes* | *Microascales* | *Microascaceae* | *Microascus* spp. | *Microascus* spp. | insects | Canada, Slovakia, Slovenia | 3 | Vanderwolf et al. (2013, 2019) |
| *Sordariomycetes* | *Microascales* | *Microascaceae* | *Microascus superficialis* | *Microascus superficialis* | Animal faeces | China | 1 | zhang et al. (2021) |
| *Sordariomycetes* | *Microascales* | *Microascaceae* | *Microascus trigonus* | *Microascus trigonus* | Animal faeces, Soil | China | 1 | zhang et al. (2021) |
| *Sordariomycetes* | *Microascales* | *Microascaceae* | *Parawardomyces giganteus* | *Wardomyces giganteus* | Unreported | Canada | 1 | Vanderwolf et al. (2019) |
| *Sordariomycetes* | *Microascales* | *Microascaceae* | *Petriella setifera* | *Petriella setifera* | Wood | Romania | 1 | Vanderwolf et al. (2013a) |
| *Sordariomycetes* | *Microascales* | *Microascaceae* | *Petriella* sp. | *Petriella* sp. | Unreported | China | 1 | Zhang et al. (2021) |
| *Sordariomycetes* | *Microascales* | *Microascaceae* | *Pithoascus ater* | *Pithoascus ater* | Unreported | China | 1 | Zhang et al. (2021) |
| *Sordariomycetes* | *Microascales* | *Microascaceae* | *Pithoascus cavernicola* | *Pithoascus cavernicola* | Rock sufaces | China | 1 | This study |
| *Sordariomycetes* | *Microascales* | *Microascaceae* | *Pithoascus platysporus* | *Pithoascus platysporus* | Unreported | China | 1 | Zhang et al. (2021) |
| *Sordariomycetes* | *Microascales* | *Microascaceae* | *Pseudallescheria boydii* | *Pseudallescheria boydii* | Rock sufaces, soil-sediment, bat guano | China, Nigeria, Venezuela | 3 | Vanderwolf et al. (2013a) |
| *Sordariomycetes* | *Microascales* | *Microascaceae* | *Pseudallescheria boydii* | *Scedosporium apiospermum* | Soil-sediment | Thailand | 1 | Vanderwolf et al. (2013a) |
| *Sordariomycetes* | *Microascales* | *Microascaceae* | Pseudallescheria spp. | Pseudallescheria spp. | Soil-sediment, Organic litter,, unreported, wood | China, Germany, Spain, Thailand | 4 | Vanderwolf et al. (2013a) |
| *Sordariomycetes* | *Microascales* | *Microascaceae* | *Pseudoscopulariopsis hibernica* | *Pseudoscopulariopsis hibernica* | Bat guano | China | 1 | Zhang et al. (2021) |
| *Sordariomycetes* | *Microascales* | *Microascaceae* | *Pseudowardomyces humicola* | *Wardomyces humicola* | Soil | Canada, USA | 2 | Vanderwolf et al. (2013a) |
| *Sordariomycetes* | *Microascales* | *Microascaceae* | *Scedosporium* spp. | *Scedosporium* spp. | Soil-sediment, unreported | China, Thailand | 1 | Zhang et al. (2021); Suetrong et al. (2023) |
| *Sordariomycetes* | *Microascales* | *Microascaceae* | *Scopulariopsis asperula* | *Scopulariopsis asperula* | Soil-sediment | China, Czech Republic | 2 | Vanderwolf et al. (2013a) |
| *Sordariomycetes* | *Microascales* | *Microascaceae* | *Scopulariopsis brevicaulis* | *Microascus brevicaulis* | Soil-sediment, gunao, insect, wood, Air | China, Czech Republic, France, Italy, Slovenia, Spain | 6 | Vanderwolf et al. (2013a); Yoder et al. (2015); Zhang et al. (2021) |
| *Sordariomycetes* | *Microascales* | *Microascaceae* | *Scopulariopsis brevicaulis* | *Scopulariopsis brevicaulis* | Air, soil-sediment, , bat guano, insects, wood, bat guano, discarded rubber ring, rock sufaces, soil | China, Czech Republic, France, Italy, Slovenia | 5 | Vanderwolf et al. (2013a) |
| *Sordariomycetes* | *Microascales* | *Microascaceae* | *Scopulariopsis brumptii* | *Scopulariopsis brumptii* | Soil-sediment, earthworm casts, insects, cave wall | Israel, Slovakia, Spain, USA | 4 | Vanderwolf et al. (2013a) |
| *Sordariomycetes* | *Microascales* | *Microascaceae* | *Scopulariopsis candida* | *Scopulariopsis candida* | animal bone | Poland | 1 | Dyląg et al. (2019) |
| *Sordariomycetes* | *Microascales* | *Microascaceae* | *Scopulariopsis crassa* | *Scopulariopsis crassa* | Unreported | China | 1 | Zhang et al. 2017 |
| *Sordariomycetes* | *Microascales* | *Microascaceae* | *Scopulariopsis flava* | *Scopulariopsis flava* | Unreported | China | 1 | Zhang et al. (2021) |
| *Sordariomycetes* | *Microascales* | *Microascaceae* | *Scopulariopsis fusca* | *Scopulariopsis fusca* | Air | Czech Republic | 1 | Vanderwolf et al. (2013a) |
| *Sordariomycetes* | *Microascales* | *Microascaceae* | *Scopulariopsis lanosa* | *Scopulariopsis lanosa* | Soil-sediment | Russia | 1 | Vanderwolf et al. (2013a) |
| *Sordariomycetes* | *Microascales* | *Microascaceae* | Scopulariopsis sp. | *Scopulariopsis arterofusca* | Insects | Slovenia | 1 | Vanderwolf et al. (2013a) |
| *Sordariomycetes* | *Microascales* | *Microascaceae* | *Scopulariopsis sphaerospora* | *Scopulariopsis sphaerospora* | Cave wall | China, Israel | 2 | Vanderwolf et al. (2013a) |
| *Sordariomycetes* | *Microascales* | *Microascaceae* | *Scopulariopsis* spp. | *Scopulariopsis* spp*.* | Air, Unreported, bat internal tissue, soil-sediment, bat guano, insects, organic litter | Australia, France, Mexico, Puerto Rico, Slovenia, Thailand, Venezuela | 7 | Vanderwolf et al. (2013a); Suetrong et al. (2023) |
| *Sordariomycetes* | *Microascales* | *Microascaceae* | *Stysanus amyli* | *Stysanus amyli* | Soil-sediment | Italy | 1 | Vanderwolf et al. (2013a) |
| *Sordariomycetes* | *Microascales* | *Microascaceae* | *Stysanus typhoides* | *Stysanus typhoides* | Dung, frass (not bat) | France | 1 | Vanderwolf et al. (2013a) |
| *Sordariomycetes* | *Microascales* | *Microascaceae* | *Trichurus* spp. | *Trichurus* spp. | Soil-sediment, dung, frass (not bat) | Australia, Spain, USA | 3 | Vanderwolf et al. (2013a) |
| *Sordariomycetes* | *Microascales* | *Microascaceae* | *Trichurus terrophilus* | *Trichurus terrophilus* | Wood | Belgium, China, Romania | 3 | Vanderwolf et al. (2013a) |
| *Sordariomycetes* | *Microascales* | *Microascaceae* | *Wardomyces anomalus* | *Wardomyces anomalus* | Earthworm casts | Slovakia | 1 | Vanderwolf et al. (2013a) |
| *Sordariomycetes* | *Microascales* | *Microascaceae* | *Wardomyces inflatus* | *Wardomyces inflatus* | Soil | Canada, China, USA | 1 | Vanderwolf et al. (2013a) |
| *Sordariomycetes* | *Microascales* | *Microascaceae* | *Wardomyces spp.* | *Wardomyces* spp. | Insect, soil-sediment, cave wall, bat guano, earthworm casts, Air | Canada, France, Japan, Slovakia, USA | 5 | Vanderwolf et al. (2013a) |
| *Sordariomycetes* | *Microascales* | *Microascaceae* | *Wardomycopsis dolichi* | *Wardomycopsis dolichi* | Soil | China | 1 | zhang et al. (2021) |
| *Sordariomycetes* | *Microascales* | *Microascaceae* | *Wardomycopsis ellipsoconidiophora* | *Wardomycopsis ellipsoconidiophora* | Animal faeces | China | 1 | zhang et al. (2021) |
| *Sordariomycetes* | *Microascales* | *Microascaceae* | *Wardomycopsis fusca* | *Wardomycopsis fusca* | Animal faeces, soil | China | 1 | zhang et al. (2021) |
| *Sordariomycetes* | *Microascales* | *Microascaceae* | *Wardomycopsis humicola* | *Wardomycopsis humicola* | Unreported | China | 1 | Zhang et al. (2021) |
| *Sordariomycetes* | *Microascales* | *Microascaceae* | *Wardomycopsis longicatenata)* | *Wardomycopsis longicatenata* | Unreported | China | 1 | Zhang et al. (2017) |
| *Sordariomycetes* | *Myrmecridiales* | *Incertae sedis* | *Myrmecridium schulzeri* | *Myrmecridium schulzeri* | Unreported | China | 1 | Zhang et al. (2021) |
| *Sordariomycetes* | *Myrmecridiales* | *Incertae sedis* | *Myrmecridium* sp. | *Myrmecridium* sp. | Rock | Thailand | 1 | Suetrong et al. (2023) |
| *Sordariomycetes* | *Ophiostomatales* | *Ophiostomataceae* | *Ophiostomataceae* sp*.* | *Ophiostomataceae* sp*.* | Dead bat fur, skin | Italy | 1 | Vanderwolf et al. (2013a) |
| *Sordariomycetes* | *Ophiostomatales* | *Ophiostomataceae* | *Sporothrix catenata* | *Sporothrix catenata* | Bat guano | Mexico | 1 | Vanderwolf et al. (2013a) |
| *Sordariomycetes* | *Ophiostomatales* | *Ophiostomataceae* | *Sporothrix inflata* | *Sporothrix inflata* | Precipitate | China, Germany | 2 | Burow et al. (2019); Zhang et al. (2021) |
| *Sordariomycetes* | *Ophiostomatales* | *Ophiostomataceae* | *Sporothrix polyporicola* | *Ophiostoma polyporicola* | Other | China, Poland, Sweden | 3 | Vanderwolf et al. (2013a) |
| *Sordariomycetes* | *Ophiostomatales* | *Ophiostomataceae* | *Sporothrix schenckii* | *Sporothrix schenckii* | Bat internal tissue, air | Columbia, Poland | 2 | Vanderwolf et al. (2013a) |
| *Sordariomycetes* | *Ophiostomatales* | *Ophiostomataceae* | *Sporothrix* spp. | *Sporothrix* spp. | Soil-sediment, other, cave wall, slate precipitate | Canada, Czech Republic, France, Germany, Mexico | 5 | Vanderwolf et al. (2013a); Leplat et al. (2018); Burow et al. (2019); Zhang et al. (2021) |
| *Sordariomycetes* | *Ophiostomatales* | *Ophiostomataceae* | *Sporothrix stenoceras* | *Ophiostoma stenoceras* | Wood | Norway, Romania | 2 | Vanderwolf et al. (2013a) |
| *Sordariomycetes* | *Pararamichloridiales* | *Pararamichloridiaceae* | *Pararamichloridium* sp. | *Pararamichloridium* sp*.* | Air | Thailand | 1 | Suetrong et al. (2023) |
| *Sordariomycetes* | *Phyllachorales* | *Phyllachoraceae* | Phyllachora sp. | Phyllachora sp. | Unreported | China | 1 | Zhang (2019) |
| *Sordariomycetes* | *Pleurotheciales* | *Pleurotheciaceae* | *Phaeoisaria clematidis* | *Phaeoisaria clematidis* | Unreported | China | 1 | Zhang (2019) |
| *Sordariomycetes* | *Sordariales* | *Bombardiaceae* | *Ramophialophora globispora* | *Ramophialophora globispora* | Unreported | China | 1 | Zhang et al. (2017) |
| *Sordariomycetes* | *Sordariales* | *Bombardiaceae* | *Ramophialophora petraea* | *Ramophialophora petraea* | Unreported | China | 1 | Zhang et al. (2017) |
| *Sordariomycetes* | *Sordariales* | *Bombardiaceae* | *Ramophialophora* sp*.* | *Ramophialophora* sp*.* | Unreported | China | 1 | Zhang et al. (2021) |
| *Sordariomycetes* | *Sordariales* | *Chaetomiaceae* | *Amesia nigricolor* | *Amesia nigricolor* | Air | China, Spain | 2 | Zhang et al. (2021) |
| *Sordariomycetes* | *Sordariales* | *Chaetomiaceae* | *Amesia sp.* | *Amesia* sp. | Sediment | Brazil | 1 | Alves et al. (2022) |
| *Sordariomycetes* | *Sordariales* | *Chaetomiaceae* | *Arcopilus aureus* | *Arcopilus aureus* | Cave wall | USA | 1 | Vanderwolf et al. (2013a); Zhang et al. (2021) |
| *Sordariomycetes* | *Sordariales* | *Chaetomiaceae* | *Arcopilus aureus* | *Chaetomium aureum* | Cave wall | USA | 1 | Vanderwolf et al. (2013a) |
| *Sordariomycetes* | *Sordariales* | *Chaetomiaceae* | *Arcopilus* sp. | *Arcopilus* sp. | Organic litter, | Thailand | 1 | Suetrong et al. (2023) |
| *Sordariomycetes* | *Sordariales* | *Chaetomiaceae* | *Arxotrichum succineu* | *Chaetomium succineum* | Cave wall | Israel | 1 | Vanderwolf et al. (2013a); Zhang et al. (2021) |
| *Sordariomycetes* | *Sordariales* | *Chaetomiaceae* | *Botryotrichum murorum* | *Botryotrichum murorum* | Cave wall, wood, soil | China, France, Israel | 3 | Vanderwolf et al. (2013a); Zhang et al. (2017); Ma et al. (2025) |
| *Sordariomycetes* | *Sordariales* | *Chaetomiaceae* | *Botryotrichum murorum* | *Chaetomium murorum* | Wood, cave wall | France, Israel | 2 | Vanderwolf et al. (2013a) |
| *Sordariomycetes* | *Sordariales* | *Chaetomiaceae* | Chaetomiaceae sp. | Chaetomiaceae sp. | Sediment | Bulgaria | 1 | Savković et al. (2025) |
| *Sordariomycetes* | *Sordariales* | *Chaetomiaceae* | *Chaetomidium fimeti* | *Chaetomidium fimeti* | Bat internal tissue, bat guano | Mexico | 1 | Vanderwolf et al. (2013a) |
| *Sordariomycetes* | *Sordariales* | *Chaetomiaceae* | *Chaetomidium spp.* | *Chaetomidium spp.* | Cave wall | Canada, China | 2 | Vanderwolf et al. (2019); Zhang et al. (2021) |
| *Sordariomycetes* | *Sordariales* | *Chaetomiaceae* | *Chaetomium ancistrocladum* | *Chaetomium ancistrocladum* | Unreported | China | 1 | Pereira et al. (2022) |
| *Sordariomycetes* | *Sordariales* | *Chaetomiaceae* | *Chaetomium elatum* | *Chaetomium elatum* | Wood, other, cave wall | China, France, Israel | 3 | Vanderwolf et al. (2013a); Zhang et al. (2021) |
| *Sordariomycetes* | *Sordariales* | *Chaetomiaceae* | *Chaetomium fimeti* | *Chaetomium fimeti* | Bat internal tissue | Mexico | 1 | Vanderwolf et al. (2013a) |
| *Sordariomycetes* | *Sordariales* | *Chaetomiaceae* | *Chaetomium globosum* | *Chaetomium globosum* | Air, soil-sediment, dung, frass (not bat); other, earthworm casts, wood, cave wall, Bamboo, bat guano | China, Israel, Poland, Puerto Rico, Romania, Russia, Slovakia, Spain, USA | 9 | Vanderwolf et al. (2013a); Zhang et al. (2017) |
| *Sordariomycetes* | *Sordariales* | *Chaetomiaceae* | Chaetomium globosum complex | Chaetomium globosum complex | Air | Poland | 1 | Kokurewicz et al. (2016) |
| *Sordariomycetes* | *Sordariales* | *Chaetomiaceae* | *Chaetomium piluliferum* | *Botryotrichum piluliferum* | Soil-sediment, bat guano, cave wall | Israel, Slovakia, Russia | 3 | Vanderwolf et al. (2013a) |
| *Sordariomycetes* | *Sordariales* | *Chaetomiaceae* | *Chaetomium piluliferum* | *Chaetomium piluliferum* | Air, bat guano, rock sufaces, soil, soil, sediment, bat guano, cave wall | China, Israel, Russia, Slovakia | 4 | Vanderwolf et al. (2013a); Mitova et al. (2017) |
| *Sordariomycetes* | *Sordariales* | *Chaetomiaceae* | *Chaetomium* spp. | *Chaetomium* spp. | Air, unreported, cave wall, wood, soil-sediment, earthworm casts, insects, water, Bamboo,bat guano, branch, rock surface | Belgium, Brazil, China, France, India, Japan, Russia, Slovakia, Spain, USA | 10 | Taylor et al. (2013); Vanderwolf et al. (2013a); Ma et al. (2025) |
| *Sordariomycetes* | *Sordariales* | *Chaetomiaceae* | *Chaetomium subaffine* | *Chaetomium subaffine* | Decaying twigs | China | 1 | This study |
| *Sordariomycetes* | *Sordariales* | *Chaetomiaceae* | *Chaetomium trigonosporum* | *Chaetomium trigonosporum* | Unreported | China | 1 | Zhang et al. (2017) |
| *Sordariomycetes* | *Sordariales* | *Chaetomiaceae* | *Collariella bostrychodes* | *Chaetomium bostrychodes* | Cave wall | China, Israel | 2 | Vanderwolf et al. (2013a); Man (2017) |
| *Sordariomycetes* | *Sordariales* | *Chaetomiaceae* | *Collariella bostrychodes* | *Collariella bostrychodes* | Leaf litter, cave wall, bat guano | China, Israel | 2 | Vanderwolf et al. (2013a); Zhang et al. (2021),Condé et al. 2023 |
| *Sordariomycetes* | *Sordariales* | *Chaetomiaceae* | *Collariella quadrum* | *Collariella quadrum* | Unreported | China | 1 | Zhang et al. (2017) |
| *Sordariomycetes* | *Sordariales* | *Chaetomiaceae* | *Ctenomyces vellereus* | *Myceliophthora vellerea* | Air | Czech Republic, Slovakia | 2 | Vanderwolf et al. (2013a) |
| *Sordariomycetes* | *Sordariales* | *Chaetomiaceae* | *Dichotomopilus funicola* | *Chaetomium funicola* | Soil-sediment, insects, wood | Italy, Romania, Slovakia | 3 | Vanderwolf et al. (2013a) |
| *Sordariomycetes* | *Sordariales* | *Chaetomiaceae* | *Dichotomopilus funicola* | *Dichotomopilus funicola* | soil, sediment, insect, wood | China, Italy, Romania, Slovakia | 4 | Vanderwolf et al. (2013a) |
| *Sordariomycetes* | *Sordariales* | *Chaetomiaceae* | *Dichotomopilus indicus* | *Chaetomium indicum* | Bat guano, soil-sediment, earthworm casts, wood | Romania, Slovakia | 2 | Vanderwolf et al. (2013a) |
| *Sordariomycetes* | *Sordariales* | *Chaetomiaceae* | *Dichotomopilus indicus* | *Dichotomopilus indicus* | Unreported | Unknow |  | Zhang et al. (2021) |
| *Sordariomycetes* | *Sordariales* | *Chaetomiaceae* | *Guanomyces polythrix* | *bat guanomyces polythrix* | Bat guano | Mexico | 1 | Vanderwolf et al. (2013a) |
| *Sordariomycetes* | *Sordariales* | *Chaetomiaceae* | *Gymnascella* spp. | *Gymnascella* spp. | Cave wall, Organic litter, soil-sediment | Thailand, USA | 2 | Vanderwolf et al. (2013a); Suetrong et al. (2023) |
| *Sordariomycetes* | *Sordariales* | *Chaetomiaceae* | *Humicola brunnea* | *Humicola brunnea* | Unreported | Czech Republic | 1 | Vanderwolf et al. (2013a) |
| *Sordariomycetes* | *Sordariales* | *Chaetomiaceae* | *Humicola brunnea* | Humicola brunnea var. africana | Other | Czech Republic | 1 | Vanderwolf et al. (2013a) |
| *Sordariomycetes* | *Sordariales* | *Chaetomiaceae* | *Humicola fuscoatra* | *Humicola fuscoatra* | Bat guano, soil-sediment, other | Czech Republic, India, Italy, Slovakia, USA | 5 | Vanderwolf et al. (2013a) |
| *Sordariomycetes* | *Sordariales* | *Chaetomiaceae* | *Humicola nigrescens* | *Humicola nigrescens* | Soil-sediment | Russia | 1 | Vanderwolf et al. (2013a) |
| *Sordariomycetes* | *Sordariales* | *Chaetomiaceae* | *Humicola seminuda* | *Chaetomium seminudum* | Air, cave wall | Brazil, Israel | 2 | Vanderwolf et al. (2013a); Cunha et al. (2020) |
| *Sordariomycetes* | *Sordariales* | *Chaetomiaceae* | *Humicola seminuda* | *Farrowia seminuda* | Cave wall | Israel | 1 | Vanderwolf et al. (2013a) |
| *Sordariomycetes* | *Sordariales* | *Chaetomiaceae* | *Humicola seminuda* | *Humicola cf. seminuda* | Air, bat guano | Brazil | 1 | Cunha et al. (2020) |
| *Sordariomycetes* | *Sordariales* | *Chaetomiaceae* | *Humicola* spp. | *Humicola* spp. | Unreported, soil-sediment, insects, earthworm casts | Bahamas, Belgium, Brazil, Canada, China, India, Korea, Puerto Rico, Slovakia, Thailand, USA | 11 | Vanderwolf et al. (2013, 2015, 2019); Suetrong et al. (2023) |
| *Sordariomycetes* | *Sordariales* | *Chaetomiaceae* | *Humicola udagawae* | *Chaetomium udagawae* | Unreported | China | 1 | Zhang et al. (2017) |
| *Sordariomycetes* | *Sordariales* | *Chaetomiaceae* | *Myceliophthora* spp. | *Myceliophthora* spp. | Air, bat guano, unreported | Brazil, France, Romania, Slovakia | 4 | Vanderwolf et al. (2013a, 2015); Nováková et al. (2018); Cunha et al. (2020) |
| *Sordariomycetes* | *Sordariales* | *Chaetomiaceae* | *Mycothermus thermophilus* | *Mycothermus thermophilus* | Soil | USA | 1 | Zhang et al. (2014) |
| *Sordariomycetes* | *Sordariales* | *Chaetomiaceae* | *Neoachaetomiella cavernicola* | *Neoachaetomiella cavernicola* | Rock surface | China | 1 | This study |
| *Sordariomycetes* | *Sordariales* | *Chaetomiaceae* | *Ovatospora* spp. | *Ovatospora* spp. | Sediment | Brazil | 1 | Vanderwolf et al. (2013a); Savković et al. (2025); Alves et al. (2022) |
| *Sordariomycetes* | *Sordariales* | *Chaetomiaceae* | *Pleotrichocladium opacum* | *Trichocladium opacum* | Soil-sediment | Canada, Slovakia, USA | 3 | Vanderwolf et al. (2013a) |
| *Sordariomycetes* | *Sordariales* | *Chaetomiaceae* | *Thermochaetoides thermophila* | *Chaetomium thermophilum* | Soil-sediment | India | 1 | Vanderwolf et al. (2013a) |
| *Sordariomycetes* | *Sordariales* | *Chaetomiaceae* | *Thermothelomyces thermophilus* | *Myceliophthora thermophila* | Bat guano | France | 1 | Vanderwolf et al. (2013a) |
| *Sordariomycetes* | *Sordariales* | *Chaetomiaceae* | *Thermothelomyces thermophilus* | *Thermothelomyces thermophilus* | Unreported | Unknow |  | Zhang et al. (2021) |
| *Sordariomycetes* | *Sordariales* | *Chaetomiaceae* | *Trichocladium arxii* | *Chaetomidium arxii* | Unreported | China | 1 | Zhang et al. (2017) |
| *Sordariomycetes* | *Sordariales* | *Chaetomiaceae* | *Trichocladium asperum* | *Trichocladium asperum* | Bat guano, rock sufaces | China | 1 | Zhang et al. (2017) |
| *Sordariomycetes* | *Sordariales* | *Chaetomiaceae* | *Trichocladium cavernicola* | *Trichocladium cavernicola* | Air | China | 1 | This study |
| *Sordariomycetes* | *Sordariales* | *Chaetomiaceae* | *Trichocladium crispatum* | *Chaetomium crispatum* | Earthworm casts | Slovakia | 1 | Vanderwolf et al. (2013a) |
| *Sordariomycetes* | *Sordariales* | *Chaetomiaceae* | *Trichocladium crispatum* | *Trichocladium crispatum* | Earthworm casts, water | China, Poland Slovakia | 3 | Vanderwolf et al. (2013a); Zhang et al. (2021) |
| *Sordariomycetes* | *Sordariales* | *Chaetomiaceae* | *Trichocladium griseum* | *Humicola grisea* | Air, soil-sediment, bat guano, earthworm casts, wood | Bulgaria, Czech Republic, France, India, Poland, Puerto Rico, Romania, Russia, Slovakia, USA | 10 | Vanderwolf et al. (2013a); Mitova et al. (2017) |
| *Sordariomycetes* | *Sordariales* | *Chaetomiaceae* | *Trichocladium griseum* | *Trichocladium griseum* | air, soil and sediment | Poland, Iran | 2 | Vanderwolf et al. (2013a); Mitova et al. (2017); Savković et al. (2025) |
| *Sordariomycetes* | *Sordariales* | *Chaetomiaceae* | *Trichocladium heterothallicum* | *Chaetomium heterothallicum* | Unreported | China | 1 | Zhang et al. (2021) |
| *Sordariomycetes* | *Sordariales* | *Chaetomiaceae* | *Trichocladium* spp. | *Trichocladium* spp. | Soil-sediment | China, USA | 2 | Vanderwolf et al. (2013a) |
| *Sordariomycetes* | *Sordariales* | *Chaetomiaceae* | *Xanthiomyces spainosus* | *Chaetomium spainosum* | Bat guano, earthworm casts | Slovakia | 1 | Vanderwolf et al. (2013a) |
| *Sordariomycetes* | *Sordariales* | *Helminthosphaeriaceae* | *Endophragmiella* sp. | *Endophragmiella* sp. | Air | Spain | 1 | Vanderwolf et al. (2013a) |
| *Sordariomycetes* | *Sordariales* | *Incertae sedis* | *Melanocarpus albomyces* | *Melanocarpus albomyces* | soil-sediment | China (Taiwan Province) | 1 | Vanderwolf et al. (2013a) |
| *Sordariomycetes* | *Sordariales* | *Lasiosphaeriaceae* | *Mammaria echinobotryoides* | *Mammaria echinobotryoides* | Soil-sediment, earthworm casts | Canada, Slovakia | 2 | Vanderwolf et al. (2013a) |
| *Sordariomycetes* | *Sordariales* | *Lasiosphaeriaceae* | *Mammaria* sp. | *Mammaria* sp. | Bat | Canada | 1 | Vanderwolf et al. (2015) |
| *Sordariomycetes* | *Sordariales* | *Lasiosphaeriaceae* | *Zopfiella pleuropora* | *Zopfiella pleuropora* | Unreported | Canada | 1 | Vanderwolf et al. (2013a) |
| *Sordariomycetes* | *Sordariales* | *Lasiosphaeriaceae* | *Zopfiella tabulata* | *Zopfiella tabulata* | Unreported | China | 1 | Zhang et al. (2021) |
| *Sordariomycetes* | *Sordariales* | *Podosporaceae* | *Cladorrhinum globisporum* | *Cladorrhinum globisporum* | Unreported | China | 1 | Zhang et al. (2017) |
| *Sordariomycetes* | *Sordariales* | *Podosporaceae* | *Cladorrhinum hyalocarpum* | *Thielavia hyalocarpa* | Unreported | China | 1 | Zhang et al. (2021) |
| *Sordariomycetes* | *Sordariales* | *Podosporaceae* | *Cladorrhinum* sp. | *Cladorrhinum* sp. | Unreported | China | 1 | Zhang et al. (2017) |
| *Sordariomycetes* | *Sordariales* | *Podosporaceae* | Podospora spp. | Podospora spp. | Soil-sediment, bat guano | China, Iran, USA | 3 | Vanderwolf et al. (2013a); Zhang et al. (2014) |
| *Sordariomycetes* | *Sordariales* | *Schizotheciaceae* | *Schizothecium inaequale* | *Schizothecium inaequale* | cave wall, limestone | China | 1 | Jiang et al. (2017a, b) |
| *Sordariomycetes* | *Sordariales* | *Schizotheciaceae* | *Schizothecium* sp. | *Schizothecium* sp. | Unreported | China | 1 | Zhang et al. (2021) |
| *Sordariomycetes* | *Sordariales* | *Sordariaceae* | *Chrysonilia crassa* | *Chrysonilia crassa* | Cave wall | Israel | 1 | Vanderwolf et al. (2013a) |
| *Sordariomycetes* | *Sordariales* | *Sordariaceae* | *Neurospora crassa* | *Neurospora crassa* | Soil-sediment | Puerto Rico | 1 | Vanderwolf et al. (2013a) |
| *Sordariomycetes* | *Sordariales* | *Sordariaceae* | *Neurospora intermedia* | *Neurospora intermedia* | Unreported | China | 1 | Zhang et al. (2017) |
| *Sordariomycetes* | *Sordariales* | *Sordariaceae* | *Neurospora* sp. | *Neurospora* sp. | Bat guano | Puerto Rico | 1 | Vanderwolf et al. (2013a) |
| *Sordariomycetes* | *Sordariales* | *Sordariaceae* | *Neurospora tetrasperma* | *Neurospora tetrasperma* | Air | Poland | 1 | Pusz et al. (2015) |
| *Sordariomycetes* | *Sordariales* | *Sordariaceae* | *Sordaria fimicola* | *Sordaria fimicola* | Wood | China, Poland, Romania | 3 | Vanderwolf et al. (2013a); Ogórek et al. (2014a, b) |
| *Sordariomycetes* | *Sordariales* | *Sordariaceae* | *Sordaria* spp. | *Sordaria* spp. | Soil-sediment, air | Spain, USA | 2 | Vanderwolf et al. (2013a) |
| *Sordariomycetes* | *Sphaeriales* |  | Sphaeriales sp. | Sphaeriales sp. | Unreported | UK | 1 | Vanderwolf et al. (2013a) |
| *Sordariomycetes* | *Togniniales* | *Togniniaceae* | *Phaeoacremonium argentinense* | *Phaeoacremonium argentinense* | Unreported | China | 1 | Zhang et al. (2017) |
| *Sordariomycetes* | *Togniniales* | *Togniniaceae* | *Phaeoacremonium iranianum* | *Phaeoacremonium iranianum* | Unreported | China | 1 | Zhang (2019) |
| *Sordariomycetes* | *Togniniales* | *Togniniaceae* | *Phaeoacremonium minimum* | *Phaeoacremonium minimum* | Unreported | China | 1 | Zhang (2019) |
| *Sordariomycetes* | *Togniniales* | *Togniniaceae* | *Phaeoacremonium novae-zealandiae* | *Phaeoacremonium novae-zealandiae* | Unreported | China | 1 | Zhang et al. (2021) |
| *Sordariomycetes* | *Togniniales* | *Togniniaceae* | *Phaeoacremonium occidentale* | *Phaeoacremonium occidentale* | Unreported | China | 1 | Zhang (2019) |
| *Sordariomycetes* | *Togniniales* | *Togniniaceae* | *Phaeoacremonium rubrigenum* | *Phaeoacremonium rubrigenum* | Water | China | 1 | Jiang et al. (2017) |
| *Sordariomycetes* | *Togniniales* | *Togniniaceae* | *Phaeoacremonium* sp. | *Togninia* sp. | Unreported | China | 1 | Zhang (2017) |
| *Sordariomycetes* | *Togniniales* | *Togniniaceae* | *Phaeoacremonium* spp. | *Phaeoacremonium* spp. | Cave wall, limestone | China | 1 | Jiang et al. (2017a, b); Vanderwolf et al. (2019) |
| *Sordariomycetes* | *Togniniales* | *Togniniaceae* | *Phaeoacremonium viticola* | *Phaeoacremonium viticola* | Unreported | China | 1 | Zhang (2019) |
| *Sordariomycetes* | *Trichosphaeriales* | *Trichosphaeriaceae* | *Brachysporium echinoides* | *Brachysporium echinoides* | Dung, frass (not bat) | France | 1 | Vanderwolf et al. (2013a) |
| *Sordariomycetes* | *Trichosphaeriales* | *Trichosphaeriaceae* | *Stachylidium* sp. | *Stachylidium* sp. | Water | Russia | 1 | Vanderwolf et al. (2013a) |
| *Sordariomycetes* | *Xylariales* | *Diatrypaceae* | Diatrypaceae sp. | Diatrypaceae sp. | Air | Spain, Thailand | 2 | Vanderwolf et al. (2013a) |
| *Sordariomycetes* | *Xylariales* | *Diatrypaceae* | *Diatrype palmicola* | *Diatrype palmicola* | Unreported | China | 1 | Pereira et al. (2022) |
| *Sordariomycetes* | *Xylariales* | *Diatrypaceae* | *Diatrype stigma* | *Diatrype stigma* | Unreported | China | 1 | Pereira et al. (2022) |
| *Sordariomycetes* | *Xylariales* | *Diatrypaceae* | *Diatrypella major* | *Diatrypella major* | Unreported | China | 1 | Zhang et al. (2021) |
| *Sordariomycetes* | *Xylariales* | *Diatrypaceae* | *Diatrypella pulvinata* | *Diatrypella pulvinata* | Unreported | China | 1 | Zhang et al. (2021) |
| *Sordariomycetes* | *Xylariales* | *Diatrypaceae* | *Eutypella* sp. | *Eutypella* sp. | Unreported | Thailand | 1 | Suetrong et al. (2023) |
| *Sordariomycetes* | *Xylariales* | *Diatrypaceae* | *Paraeutypella citricola* | *Eutypella citricola* | Unreported | China | 1 | China |
| *Sordariomycetes* | *Xylariales* | *Diatrypaceae* | *Peroneutypa scoparia* | *Eutypella scoparia* | Soil | China | 1 | Jiang et al. (2017) |
| *Sordariomycetes* | *Xylariales* | *Diatrypaceae* | *Peroneutypa scoparia* | *Peroneutypa scoparia* | Unreported | China | 1 | Zhang et al. (2017) |
| *Sordariomycetes* | *Xylariales* | *Diatrypaceae* | *Peroneutypa* spp. | *Peroneutypa* spp. | Water, soil-sediment, | China, Thailand | 2 | Zhang et al. (2021); Suetrong et al. (2023) |
| *Sordariomycetes* | *Xylariales* | *Graphostromataceae* | *Biscogniauxia petrensis* | *Biscogniauxia petrensis* | Soil | China | 1 | Zhang et al. (2017) |
| *Sordariomycetes* | *Xylariales* | *Graphostromataceae* | *Biscogniauxia* spp. | *Biscogniauxia* spp. | Organic litter, soil | China, Thailand | 2 | Zhang et al. (2017); Suetrong et al. (2023) |
| *Sordariomycetes* | *Xylariales* | *Hypoxylaceae* | *Annulohypoxylon nitens* | *Annulohypoxylon nitens* | Bat guano, speleothem | Malaysia | 1 | Wasti et al. (2021) |
| *Sordariomycetes* | *Xylariales* | *Hypoxylaceae* | Annulohypoxylon sp. | Annulohypoxylon sp. | Unreported | China | 1 | Zhang et al. (2021) |
| *Sordariomycetes* | *Xylariales* | *Hypoxylaceae* | *Annulohypoxylon stygium* | *Annulohypoxylon stygium* | Unreported | China | 1 | Zhang et al. (2021) |
| *Sordariomycetes* | *Xylariales* | *Hypoxylaceae* | *Daldinia concentrica* | *Daldinia concentrica* | Wood | Slovenia, UK | 2 | Vanderwolf et al. (2013a) |
| *Sordariomycetes* | *Xylariales* | *Hypoxylaceae* | *Daldinia eschscholtzii* | *Daldinia eschscholtzii* | Rock surface | China | 1 | This study |
| *Sordariomycetes* | *Xylariales* | *Hypoxylaceae* | *Hypoxylon fragiforme* | *Hypoxylon fragiforme* | Air, rock surface | China, France | 2 | Vanderwolf et al. (2013a) |
| *Sordariomycetes* | *Xylariales* | *Hypoxylaceae* | Hypoxylon spp. | Hypoxylon spp. | Air, bat guano, rock, water, soil-sediment, wood | Brazil, Puerto Rico, Thailand | 3 | Vanderwolf et al. (2013a); Suetrong et al. (2023); Cunha et al. (2020) |
| *Sordariomycetes* | *Xylariales* | *Hypoxylaceae* | *Jackrogersella* sp. | *Jackrogersella* sp. | Unreported | China | 1 | Zhang et al. (2021) |
| *Sordariomycetes* | *Xylariales* | *Hypoxylaceae* | *Nodulisporium* sp. | *Nodulisporium* sp*.* | Organic litter | Thailand | 1 | Suetrong et al. (2023) |
| *Sordariomycetes* | *Xylariales* | *Hypoxylaceae* | *Phylacia bomba* | Phylacia aff. bomba | Soil-sediment | Puerto Rico | 1 | Vanderwolf et al. (2013a) |
| *Sordariomycetes* | *Xylariales* | *Hypoxylaceae* | *Phylacia bomba* | *Phylacia bomba* | Soil-sediment | Puerto Rico | 1 | Vanderwolf et al. (2013a) |
| *Sordariomycetes* | *Xylariales* | *Incertae sedis* | *Pseudosubramaniomyces fusisaprophyticus* | *Pseudosubramaniomyces fusisaprophyticus* | Air | Spain | 1 | Savković et al. (2025) |
| *Sordariomycetes* | *Xylariales* | *Lopadostomataceae* | *Creosphaeria sassafras* | *Creosphaeria sassafras* | Unreported | China | 1 | Pereira et al. (2022) |
| *Sordariomycetes* | *Xylariales* | *Microdochiaceae* | *Idriella lunata* | *Idriella lunata* | Unreported | China | 1 | Zhang et al. (2021) |
| *Sordariomycetes* | *Xylariales* | *Microdochiaceae* | *Idriella* sp. | *Idriella* sp. | Unreported | China | 1 | Zhang et al. (2021) |
| *Sordariomycetes* | *Xylariales* | *Microdochiaceae* | *Microdochium bolleyi* | *Microdochium bolleyi* | Unreported | China, Poland | 2 | Pusz et al. (2018a) |
| *Sordariomycetes* | *Xylariales* | *Microdochiaceae* | *Microdochium chrysanthemoides* | *Microdochium chrysanthemoides* | Unreported | China | 1 | Zhang et al. (2017) |
| *Sordariomycetes* | *Xylariales* | *Microdochiaceae* | *Microdochium fisheri* | *Microdochium fisheri* | Unreported | China | 1 | Zhang (2019) |
| *Sordariomycetes* | *Xylariales* | *Microdochiaceae* | *Microdochium lycopodinum* | *Microdochium lycopodinum* | Air | China | 1 | Jiang et al. (2017) |
| *Sordariomycetes* | *Xylariales* | *Microdochiaceae* | *Microdochium nivale* | *Microdochium nivale* | wood | UK | 1 | Vanderwolf et al. (2013a) |
| *Sordariomycetes* | *Xylariales* | *Microdochiaceae* | *Microdochium seminicola* | *Microdochium seminicola* | Air | Slovakia | 1 | Ogórek et al. (2018); Ogórek (2018b) |
| *Sordariomycetes* | *Xylariales* | *Xylariaceae* | *Circinotrichum* sp. | *Circinotrichum* sp. | Air | Spain | 1 | Vanderwolf et al. (2013a); Zhang et al. (2021) |
| *Sordariomycetes* | *Xylariales* | *Xylariaceae* | *Hansfordia* sp*.* | *Hansfordia* sp. | Unreported | Antarctica | 1 | Connell and Staudigel (2013) |
| *Sordariomycetes* | *Xylariales* | *Xylariaceae* | *Helicogermslita* sp*.* | *Helicogermslita* sp*.* | Air | Spain | 1 | Vanderwolf et al. (2013a) |
| *Sordariomycetes* | *Xylariales* | *Xylariaceae* | *Kretzschmaria deusta* | *Kretzschmaria deusta* | Unreported | France | 1 | Vanderwolf et al. (2013a) |
| *Sordariomycetes* | *Xylariales* | *Xylariaceae* | *Nemania bipapillata* | *Nemania bipapillata* | Unreported | China | 1 | Zhang et al. (2017) |
| *Sordariomycetes* | *Xylariales* | *Xylariaceae* | *Nemania diffusa* | *Nemania diffusa* | Unreported | China | 1 | Zhang et al. (2017) |
| *Sordariomycetes* | *Xylariales* | *Xylariaceae* | *Nemania serpens* | *Nemania serpens* | Unreported | China | 1 | Zhang (2019) |
| *Sordariomycetes* | *Xylariales* | *Xylariaceae* | *Nemania* spp. | *Nemania* spp. | Rock | China, Thailand | 1 | Zhang et al. (2021); Suetrong et al. (2023) |
| *Sordariomycetes* | *Xylariales* | *Xylariaceae* | *Pidoplitchkoviella terricola* | *Pidoplitchkoviella terricola* | Earthworm casts | Slovakia | 1 | Vanderwolf et al. (2013a) |
| *Sordariomycetes* | *Xylariales* | *Xylariaceae* | *Rosellinia aquila* | *Rosellinia aquila* | Wood | France, UK | 2 | Vanderwolf et al. (2013a) |
| *Sordariomycetes* | *Xylariales* | *Xylariaceae* | *Stilbohypoxylon* sp. | *Stilbohypoxylon sp.* | Soil-sediment, | Thailand | 1 | Suetrong et al. (2023) |
| *Sordariomycetes* | *Xylariales* | *Xylariaceae* | *Virgaria nigra* | *Virgaria nigra* | Unreported | China | 1 | Zhang et al. (2017) |
| *Sordariomycetes* | *Xylariales* | *Xylariaceae* | *Whalleya microplaca* | *Whalleya microplaca* | Unreported | China | 1 | Zhang et al. (2021) |
| *Sordariomycetes* | *Xylariales* | *Xylariaceae* | *Xylaria anisopleura* | *Xylaria anisopleura* | Bat guano, wood | Puerto Rico | 1 | Vanderwolf et al. (2013a) |
| *Sordariomycetes* | *Xylariales* | *Xylariaceae* | *Xylaria arbuscula* | *Xylaria arbuscula* | Unreported | China | 1 | Zhang et al. (2017) |
| *Sordariomycetes* | *Xylariales* | *Xylariaceae* | *Xylaria corniformis* | *Xylaria corniformis* | Unreported, wood | Germany | 1 | Vanderwolf et al. (2013a) |
| *Sordariomycetes* | *Xylariales* | *Xylariaceae* | *Xylaria feejeensis* | *Xylaria feejeensis* | Speleothem | Malaysia | 1 | Wasti et al. (2021) |
| *Sordariomycetes* | *Xylariales* | *Xylariaceae* | *Xylaria hypoxylon* | *Xylaria hypoxylon* | Wood | China, Switzerland, UK | 3 | Vanderwolf et al. (2013a) |
| *Sordariomycetes* | *Xylariales* | *Xylariaceae* | *Xylaria kegeliana* | *Xylaria kegeliana* | Bat guano, wood | Puerto Rico | 1 | Vanderwolf et al. (2013a) |
| *Sordariomycetes* | *Xylariales* | *Xylariaceae* | *Xylaria longipes* | *Xylaria longipes* | Unreported, wood | Germany | 1 | Vanderwolf et al. (2013a) |
| *Sordariomycetes* | *Xylariales* | *Xylariaceae* | *Xylaria palmicola* | *Xylaria palmicola* | Unreported | China | 1 | Zhang et al. (2021) |
| *Sordariomycetes* | *Xylariales* | *Xylariaceae* | *Xylaria polymorpha* | *Xylaria polymorpha* | Wood, soil-sediment | Bosnia, Puerto Rico | 2 | Vanderwolf et al. (2013a) |
| *Sordariomycetes* | *Xylariales* | *Xylariaceae* | *Xylaria schweinitzii* | *Xylaria schweinitzii* | Unreported | China | 1 | Zhang et al. (2017) |
| *Sordariomycetes* | *Xylariales* | *Xylariaceae* | Xylaria spp. | Xylaria spp. | Air, rock, water, soil-sediment, wood | China, Croatia, Germany, Puerto Rico, Thailand, UK | 6 | Vanderwolf et al. (2013a); Zhang et al. (2017) |
| *Sordariomycetes* | *Xylariales* | *Xylariaceae* | *Xylaria venosula* | *Xylaria venosula* | Unreported | China | 1 | Zhang et al. (2021) |
| *Sordariomycetes* | *Xylariales* | *Xylariaceae* | Xylariaceae sp. | Xylariaceae sp. | Air | Spain, Thailand | 2 | Vanderwolf et al. (2013a) |
| *Sordariomycetes* | *Xylariales* | *Xylariaceae* | Zakatoshia sp. | Zakatoshia sp. | Air | Czech Republic | 1 | Vanderwolf et al. (2013a) |
| *Sordariomycetes* |  |  | Sordariomycetes sp. | Sordariomycete sp. | Soil-sediment | USA | 1 | Vanderwolf et al. (2013a) |
| *Sordariomycetes* |  |  | Sordariomycetidae sp. | Sordariomycetidae sp. | Soil-sediment, | Thailand | 1 | Suetrong et al. (2023) |
|  |  |  | Ascomycota spp. | Ascomycota spp. | Unreported | China, Thailand | 2 | Man (2017); Suetrong et al. (2023) |
| ***Basidiomycota*** |  |  |  |  |  |  |  |  |
| *Agaricomycetes* | *Agaricales* | *Agaricaceae* | *Agaricus* spp. | *Agaricus* spp. | Air | Germany, Spain | 2 | Vanderwolf et al. (2013a) |
| *Agaricomycetes* | *Agaricales* | *Agaricaceae* | *Coprinopsis cinerea* | *Coprinus tomentosus* | Unreported | Austria | 1 | Vanderwolf et al. (2013a) |
| *Agaricomycetes* | *Agaricales* | *Agaricaceae* | *Coprinus* sp. | *Coprinus* sp. | Air, unreported, sediment, soil, other, dung, frass (not bat), wood, bat guano | Belgium, Croatia, France, Germany, Malaysia, Puerto Rico, Spain, UK, USA | 9 | Vanderwolf et al. (2013a) |
| *Agaricomycetes* | *Agaricales* | *Agaricaceae* | *Coprinus sterquilinus* | *Coprinus sterquilinus* | Wood | Romania | 1 | Vanderwolf et al. (2013a) |
| *Agaricomycetes* | *Agaricales* | *Agaricaceae* | *Lepiota* sp. | *Lepiota* sp. | Sediment, soil, bat guano | Puerto Rico | 1 | Vanderwolf et al. (2013a) |
| *Agaricomycetes* | *Agaricales* | *Agaricaceae* | *Leucoagaricus* sp. | *Leucoagaricus* sp. | sediment | China | 1 | Man (2017) |
| *Agaricomycetes* | *Agaricales* | *Clavariaceae* | *Clavaria* sp. | *Clavaria* sp. | Wood, unreported | Brazil, Italy | 2 | Vanderwolf et al. (2013a) |
| *Agaricomycetes* | *Agaricales* | *Clitocybaceae* | *Clitocybe* sp*.* | *Clitocybe* sp*.* | Bat guano, unreported | Austria, Puerto Rico | 2 | Vanderwolf et al. (2013a) |
| *Agaricomycetes* | *Agaricales* | *Clitocybaceae* | *Collybia* sp. | *Collybia* sp. | Unreported, wood | France, Germany, Switzerland | 3 | Vanderwolf et al. (2013a) |
| *Agaricomycetes* | *Agaricales* | *Clitocybaceae* | *Gymnopus johnstonii* | *Collybia johnstoni* | Bat guano | Puerto Rico | 1 | Vanderwolf et al. (2013a) |
| *Agaricomycetes* | *Agaricales* | *Clitocybaceae* | *Tricholomopsis aurea* | *Collybia aurea* | Bat guano | Puerto Rico | 1 | Vanderwolf et al. (2013a) |
| *Agaricomycetes* | *Agaricales* | *Cortinariaceae* | Cortinarius sp. | *Cortinarius* sp. | Air | Spain | 1 | Vanderwolf et al. (2013a) |
| *Agaricomycetes* | *Agaricales* | *Crepidotaceae* | *Crepidotus applanatus* | *Crepidotus applanatus* | Wood | Romania | 1 | Vanderwolf et al. (2013a) |
| *Agaricomycetes* | *Agaricales* | *Crepidotaceae* | *Crepidotus mollis* | *Crepidotus mollis* | Unreported, wood | Germany | 1 | Vanderwolf et al. (2013a) |
| *Agaricomycetes* | *Agaricales* | *Cyphellaceae* | Chondrostereum sp. | *Chondrostereum* sp. | Bat | Brazil | 1 | Cunha et al. (2020) |
| *Agaricomycetes* | *Agaricales* | *Entolomataceae* | *Clitopilus kamaka* | *Clitopilus kamaka* | Unreported | China | 1 | Zhang et al. (2017) |
| *Agaricomycetes* | *Agaricales* | *Entolomataceae* | *Clitopilus prunulus* | *Clitopilus prunulus* | Unreported | China | 1 | Zhang et al. (2021) |
| *Agaricomycetes* | *Agaricales* | *Entolomataceae* | *Clitopilus scyphoides* | *Clitopilus scyphoides* | Unreported | Spain | 1 | Vanderwolf et al. (2013a) |
| *Agaricomycetes* | *Agaricales* | *Entolomataceae* | *Clitopilus* sp. | *Clitopilus* sp. | Unreported | China | 1 | Zhang et al. (2021) |
| *Agaricomycetes* | *Agaricales* | *Galeropsidaceae* | *Coprinarius subtilis* | *Coprinarius subtilis* | Wood | Switzerland | 1 | Vanderwolf et al. (2013a) |
| *Agaricomycetes* | *Agaricales* | *Hydnangiaceae* | *Laccaria laccata* | *Laccaria laccata* | Wood | Czech Republic | 1 | Vanderwolf et al. (2013a) |
| *Agaricomycetes* | *Agaricales* | *Hygrophoraceae* | *Hygrophorus* sp. | *Hygrophorus* sp. | Unreported | UK | 1 | Vanderwolf et al. (2013a) |
| *Agaricomycetes* | *Agaricales* | *Hymenogastraceae* | *Galerina camerina* | *Galerina camerina* | Unreported | Spain | 1 | Vanderwolf et al. (2013a) |
| *Agaricomycetes* | *Agaricales* | *Hymenogastraceae* | *Galerina marginata* | *Galerina unicolor* | Unreported, wood | Germany, Spain | 2 | Vanderwolf et al. (2013a) |
| *Agaricomycetes* | *Agaricales* | *Hymenogastraceae* | *Galerina praticola* | *Galerina praticola* | Unreported | Unknow |  | Zhang et al. (2021) |
| *Agaricomycetes* | *Agaricales* | *Hymenogastraceae* | *Galerina pumila* | *Galerina mycenopsis* | Wood | Germany | 1 | Vanderwolf et al. (2013a) |
| *Agaricomycetes* | *Agaricales* | *Hymenogastraceae* | *Galerina pumila* | *Galerina pumila* | Unreported | Unknow |  | Zhang et al. (2021) |
| *Agaricomycetes* | *Agaricales* | *Hymenogastraceae* | *Hymenogaster* sp. | *Hymenogaster* sp. | Unreported | France | 1 | Vanderwolf et al. (2013a) |
| *Agaricomycetes* | *Agaricales* | *Hymenogastraceae* | *Hymenogaster vulgaris* | *Hymenogaster vulgaris* | Unreported | France | 1 | Vanderwolf et al. (2013a) |
| *Agaricomycetes* | *Agaricales* | *Hymenogastraceae* | *Naucoria* sp. | *Naucoria* sp. | Unreported | France | 1 | Vanderwolf et al. (2013a) |
| *Agaricomycetes* | *Agaricales* | *Hymenogastraceae* | Phaeocollybia sp. | *Phaeocollybia* sp. | Unreported | China | 1 | Zhang et al. (2021) |
| *Agaricomycetes* | *Agaricales* | *Hymenogastraceae* | Psilocybe sp. | *Psilocybe* sp. | Unreported | China | 1 | Zhang et al. (2021) |
| *Agaricomycetes* | *Agaricales* | *Incertae sedis* | Baeospora myosura | Baeospora myosura | Unreported, wood | Germany | 1 | Vanderwolf et al. (2013a) |
| *Agaricomycetes* | *Agaricales* | *Incertae sedis* | Baeospora myriadophylla | Baeospora myriadophylla f. cavernatilis | Wood | Germany | 1 | Vanderwolf et al. (2013a) |
| *Agaricomycetes* | *Agaricales* | *Incertae sedis* | Baeospora sp. | Baeospora sp. | Wood | Canada | 1 | Vanderwolf et al. (2013a) |
| *Agaricomycetes* | *Agaricales* | *Incertae sedis* | *Crucibulum crucibuliforme* | *Crucibulum crucibuliforme* | Unreported, wood | Germany | 1 | Vanderwolf et al. (2013a) |
| *Agaricomycetes* | *Agaricales* | *Incertae sedis* | *Delicatula integrella* | *Delicatula integrella* | Unreported, wood | France, Spain | 2 | Vanderwolf et al. (2013a) |
| *Agaricomycetes* | *Agaricales* | *Incertae sedis* | *Delicatula microscopica* | *Delicatula microscopica* | Wood | Switzerland | 1 | Vanderwolf et al. (2013a) |
| *Agaricomycetes* | *Agaricales* | *Incertae sedis* | *Fayodia gracilipes* | *Fayodia gracilipes* | Wood | Czech Republic | 1 | Vanderwolf et al. (2013a) |
| *Agaricomycetes* | *Agaricales* | *Incertae sedis* | *Fayodia* sp. | *Fayodia* sp. | Unreported | Austria | 1 | Vanderwolf et al. (2013a) |
| *Agaricomycetes* | *Agaricales* | *Incertae sedis* | *Fistulina hepatica* | *Fistulina hepatica* | Air | Australia | 1 | Vanderwolf et al. (2013a) |
| *Agaricomycetes* | *Agaricales* | *Inocybaceae* | *Inocybe* sp. | *Inocybe* sp. | Air | Spain | 1 | Vanderwolf et al. (2013a) |
| *Agaricomycetes* | *Agaricales* | *Lycoperdaceae* | *Bovista* sp. | *Bovista* sp. | Air | Spain | 1 | Vanderwolf et al. (2013a) |
| *Agaricomycetes* | *Agaricales* | *Lycoperdaceae* | *Calvatia* sp. | *Calvatia* sp. | Air | Spain | 1 | Vanderwolf et al. (2013a) |
| *Agaricomycetes* | *Agaricales* | *Lycoperdaceae* | Lycoperdon perlatum | *Lycoperdon perlatum* | Unreported | China | 1 | Zhang et al. (2021) |
| *Agaricomycetes* | *Agaricales* | *Marasmiaceae* | *Marasmius atrorubens* | *Marasmius atrorubens* | Sediment, soil | Puerto Rico | 1 | Vanderwolf et al. (2013a) |
| *Agaricomycetes* | *Agaricales* | *Marasmiaceae* | *Marasmius epiphyllus* | *Marasmius epiphyllus* | Unreported, other | France, Spain | 2 | Vanderwolf et al. (2013a) |
| *Agaricomycetes* | *Agaricales* | *Marasmiaceae* | *Marasmius saccharinus* | *Marasmius saccharinus* | Other | Algeria, Spain | 2 | Vanderwolf et al. (2013a) |
| *Agaricomycetes* | *Agaricales* | *Marasmiaceae* | *Marasmius saccharinus* | *Marasmius sp.* | Wood | Brazil, Croatia, France, Germany, Switzerland, UK | 6 | Vanderwolf et al. (2013a) |
| *Agaricomycetes* | *Agaricales* | *Marasmiaceae* | *Tetrapyrgos nigripes* | *Tetrapyrgos nigripes* | Unreported | USA | 1 | Vanderwolf et al. (2013a) |
| *Agaricomycetes* | *Agaricales* | *Mycenaceae* | *Atheniella flavoalba* | *Atheniella flavoalba* | Unreported | France | 1 | Vanderwolf et al. (2013a) |
| *Agaricomycetes* | *Agaricales* | *Mycenaceae* | *Atheniella flavoalba* | *Mycena flavoalba* | Unreported | France | 1 | Vanderwolf et al. (2013a) |
| *Agaricomycetes* | *Agaricales* | *Mycenaceae* | Hemimycena cucullata | *Hemimycena cucullata* | Unreported | Unknow |  | Zhang et al. (2021) |
| *Agaricomycetes* | *Agaricales* | *Mycenaceae* | *Hemimycena cucullata* | *Mycena echinipes* | Sediment, soil | France | 1 | Vanderwolf et al. (2013a) |
| *Agaricomycetes* | *Agaricales* | *Mycenaceae* | *Hemimycena cucullata* | *Mycena gypsea* | Unreported | Spain | 1 | Vanderwolf et al. (2013a) |
| *Agaricomycetes* | *Agaricales* | *Mycenaceae* | *Hemimycena lactea* | *Hemimycena lactea* | Wood, unreported | Spain, UK | 2 | Vanderwolf et al. (2013a) |
| *Agaricomycetes* | *Agaricales* | *Mycenaceae* | *Mycena acicula* | *Mycena acicula* | Unreported | France | 1 | Vanderwolf et al. (2013a) |
| *Agaricomycetes* | *Agaricales* | *Mycenaceae* | *Mycena amicta* | *Mycena amicta* | Wood, unreported | Poland | 1 | Vanderwolf et al. (2013a) |
| *Agaricomycetes* | *Agaricales* | *Mycenaceae* | *Mycena capillaris* | *Mycena capillaris* | Unreported, other, wood | France, Switzerland | 2 | Vanderwolf et al. (2013a) |
| *Agaricomycetes* | *Agaricales* | *Mycenaceae* | *Mycena galericulata* | *Mycena galericulata* | Unreported, sediment, soil, wood | France, Switzerland | 2 | Vanderwolf et al. (2013a) |
| *Agaricomycetes* | *Agaricales* | *Mycenaceae* | *Mycena metata* | *Mycena metata* | Unreported | Switzerland | 1 | Vanderwolf et al. (2013a) |
| *Agaricomycetes* | *Agaricales* | *Mycenaceae* | *Mycena mucor* | *Mycena mucor* | Unreported | Spain | 1 | Vanderwolf et al. (2013a) |
| *Agaricomycetes* | *Agaricales* | *Mycenaceae* | *Mycena polyadelpha* | *Mycena polyadelpha* | Unreported | Spain | 1 | Vanderwolf et al. (2013a) |
| *Agaricomycetes* | *Agaricales* | *Mycenaceae* | *Mycena polygramma* | *Mycena polygramma* | Unreported | Austria | 1 | Vanderwolf et al. (2013a) |
| *Agaricomycetes* | *Agaricales* | *Mycenaceae* | *Mycena* sp. | *Mycena* sp. | Unreported, bat guano, cave wall, other, wood | Antarctica, Austria, Croatia, France, Germany, Puerto Rico, Spain, UK, USA | 9 | Vanderwolf et al. (2013a) |
| *Agaricomycetes* | *Agaricales* | *Mycenaceae* | *Mycena strobilicola* | *Mycena strobilicola* | Other | Poland | 1 | Vanderwolf et al. (2013a) |
| *Agaricomycetes* | *Agaricales* | *Mycenaceae* | *Mycena stylobates* | *Mycena stylobates* | Unreported | Austria | 1 | Vanderwolf et al. (2013a) |
| *Agaricomycetes* | *Agaricales* | *Mycenaceae* | *Mycena supina* | *Mycena supina* | Unreported | France | 1 | Vanderwolf et al. (2013a) |
| *Agaricomycetes* | *Agaricales* | *Mycenaceae* | *Mycena vitilis* | *Mycena vitilis* | Unreported, wood | Austria, Germany | 2 | Vanderwolf et al. (2013a) |
| *Agaricomycetes* | *Agaricales* | *Mycenaceae* | *Panellus stipticus* | *Panellus stipticus* | Wood | Bosnia | 1 | Vanderwolf et al. (2013a) |
| *Agaricomycetes* | *Agaricales* | *Mycenaceae* | *Phloeomana alba* | *Mycena alba* | Wood | UK | 1 | Vanderwolf et al. (2013a) |
| *Agaricomycetes* | *Agaricales* | *Omphalotaceae* | *Collybiopsis ramealis* | *Marasmiellus ramealis* | Unreported | Spain | 1 | Vanderwolf et al. (2013a) |
| *Agaricomycetes* | *Agaricales* | *Omphalotaceae* | *Gymnopus johnstonii* | *Gymnopus johnstonii* | Unreported | Unknow |  | Zhang et al. (2021) |
| *Agaricomycetes* | *Agaricales* | *Physalacriaceae* | *Armillaria mellea* | *Armillaria mellea* | Wood, unreported | Austria, Bosnia, Croatia, Czech Republic, Slovenia, UK, USA | 7 | Vanderwolf et al. (2013a) |
| *Agaricomycetes* | *Agaricales* | *Physalacriaceae* | *Armillaria sinapina* | *Armillaria sinapina* | Wood | USA | 1 | Held et al. (2020) |
| *Agaricomycetes* | *Agaricales* | *Physalacriaceae* | Cylindrobasidium evolvens | *Cylindrobasidium evolvens* | Unreported | China | 1 | Zhang (2019) |
| *Agaricomycetes* | *Agaricales* | *Physalacriaceae* | *Rhizomarasmius setosus* | *Rhizomarasmius setosus* | Unreported | Unknow |  | Zhang et al. 2021 |
| *Agaricomycetes* | *Agaricales* | *Physalacriaceae* | *Strobilurus esculentus* | *Strobilurus esculentus* | Unreported | Switzerland | 1 | Vanderwolf et al. (2013a) |
| *Agaricomycetes* | *Agaricales* | *Pleurotaceae* | *Hohenbuehelia petaloides* | *Hohenbuehelia petaloides* | Wood | UK | 1 | Vanderwolf et al. (2013a) |
| *Agaricomycetes* | *Agaricales* | *Pluteaceae* | *Pluteus* sp. | *Pluteus* sp. | Unreported | France | 1 | Vanderwolf et al. (2013a) |
| *Agaricomycetes* | *Agaricales* | *Pluteaceae* | *Volvariella* sp. | *Volvariella* sp. | Bat guano | Puerto Rico | 1 | Vanderwolf et al. (2013a) |
| *Agaricomycetes* | *Agaricales* | *Porotheleaceae* | *Phloeomana alba* | *Phloeomana alba* | Wood | UK | 1 | Vanderwolf et al. (2013a) |
| *Agaricomycetes* | *Agaricales* | *Psathyrellaceae* | *Candolleomyces candolleanus* | *Psathyrella candolleana* | Cave wall | China, USA | 2 | Vanderwolf et al. (2013a); Zhang et al. (2017) |
| *Agaricomycetes* | *Agaricales* | *Psathyrellaceae* | *Coprinellus disseminatus* | *Coprinellus disseminatus* | Rock surface | China, France | 1 | Vanderwolf et al. (2013a), |
| *Agaricomycetes* | *Agaricales* | *Psathyrellaceae* | *Coprinellus domesticus* | *Coprinellus domesticus* | Wood, other, cave wall | UK | 1 | Vanderwolf et al. (2013a) |
| *Agaricomycetes* | *Agaricales* | *Psathyrellaceae* | *Coprinellus micaceus* | *Coprinellus micaceus* | Unreported, wood | Algeria, France, Poland, USA | 4 | Vanderwolf et al. (2013a) |
| *Agaricomycetes* | *Agaricales* | *Psathyrellaceae* | *Coprinellus radians* | *Coprinellus radians* | Air, wood, unreported | China, Czech Republic, Germany, UK | 4 | Vanderwolf et al. (2013a) |
| *Agaricomycetes* | *Agaricales* | *Psathyrellaceae* | *Coprinellus* spp. | *Coprinellus* spp. | Sediment, soil | China, Thailand, USA | 3 | Vanderwolf et al. (2013a); Man (2017) |
| *Agaricomycetes* | *Agaricales* | *Psathyrellaceae* | *Coprinellus truncorum* | *Coprinellus truncorum* | Unreported | USA | 1 | Vanderwolf et al. (2013a) |
| *Agaricomycetes* | *Agaricales* | *Psathyrellaceae* | *Coprinellus xanthothrix* | *Coprinellus xanthothrix* | Unreported | China | 1 | Zhang et al. (2021) |
| *Agaricomycetes* | *Agaricales* | *Psathyrellaceae* | *Coprinopsis atramentaria* | *Coprinopsis atramentaria* | Wood | China, Hungary, USA | 3 | Vanderwolf et al. (2013a); (Zhang (2019)) |
| *Agaricomycetes* | *Agaricales* | *Psathyrellaceae* | *Coprinopsis cinerea* | *Coprinopsis cinerea* | Unreported | Unknow |  | Zhang et al. (2021) |
| *Agaricomycetes* | *Agaricales* | *Psathyrellaceae* | *Coprinopsis radiata* | *Coprinopsis radiata* | Cave wall, unreported | Spain, UK | 2 | Vanderwolf et al. (2013a) |
| *Agaricomycetes* | *Agaricales* | *Psathyrellaceae* | *Hormographiella* sp. | *Hormographiella* sp. | Bat | Canada | 1 | Vanderwolf et al. (2015) |
| *Agaricomycetes* | *Agaricales* | *Psathyrellaceae* | *Ozonium aureum* | *Ozonium aureum* | Unreported | USA | 1 | Vanderwolf et al. (2013a) |
| *Agaricomycetes* | *Agaricales* | *Psathyrellaceae* | *Ozonium auricomum* | *Ozonium auricomum* | Unreported, wood | Bosnia, Slovenia, UK, USA | 4 | Vanderwolf et al. (2013a) |
| *Agaricomycetes* | *Agaricales* | *Psathyrellaceae* | *Ozonium stuposum* | *Ozonium stuposum* | Wood | Czech Republic | 1 | Vanderwolf et al. (2013a) |
| *Agaricomycetes* | *Agaricales* | *Psathyrellaceae* | *Parasola plicatilis* | *Parasola plicatilis* | Unreported | Austria, Spain | 2 | Vanderwolf et al. (2013a) |
| *Agaricomycetes* | *Agaricales* | *Psathyrellaceae* | *Psathyrella corrugis* | *Psathyra corrugis* | Wood | Italy | 1 | Vanderwolf et al. (2013a) |
| *Agaricomycetes* | *Agaricales* | *Psathyrellaceae* | *Psathyrella corrugis* | *Psathyrella corrugis* | Unreported | China | 1 | Zhang et al. (2017) |
| *Agaricomycetes* | *Agaricales* | *Psathyrellaceae* | Psathyrella sp. | *Psathyrella* sp. | Cave wall, soil-sediment | Thailand, UK, USA | 3 | Vanderwolf et al. (2013a); Suetrong et al. (2023) |
| *Agaricomycetes* | *Agaricales* | *Psathyrellaceae* | *Tulosesus ephemerus* | *Coprinellus ephemerus* | Wood | Switzerland | 1 | Vanderwolf et al. (2013a) |
| *Agaricomycetes* | *Agaricales* | *Schizophyllaceae* | *Schizophyllum commune* | *Schizophyllum commune* | Air, rock surface, wood | Bosnia, Brazil, China, Croatia, Czech Republic, Slovenia, Spain | 7 | Vanderwolf et al. (2013a); Zhang et al. (2017); Cunha et al. (2020) |
| *Agaricomycetes* | *Agaricales* | *Schizophyllaceae* | Schizophyllum sp. | *Schizophyllum* sp. | Air | Thailand | 1 | Suetrong et al. (2023) |
| *Agaricomycetes* | *Agaricales* | *Strophariaceae* | Agrocybe sp. | Agrocybe sp. | Air | Spain | 1 | Vanderwolf et al. (2013a) |
| *Agaricomycetes* | *Agaricales* | *Strophariaceae* | *Deconica hartii* | *Deconica hartii* | Wood | Canada | 1 | Vanderwolf et al. (2013a) |
| *Agaricomycetes* | *Agaricales* | *Strophariaceae* | *Deconica hartii* | *Melanotus hartii* | Wood | Canada | 1 | Vanderwolf et al. (2013a) |
| *Agaricomycetes* | *Agaricales* | *Strophariaceae* | Deconica sp. | *Deconica* sp. | Air, | Thailand | 1 | Suetrong et al. (2023) |
| *Agaricomycetes* | *Agaricales* | *Strophariaceae* | Hyphodontia palmae | *Hyphodontia palmae* | Unreported | China | 1 | Zhang et al. (2021) |
| *Agaricomycetes* | *Agaricales* | *Strophariaceae* | *Hypholoma dispersum* | *Hypholoma dispersum* | Wood, unreported | Poland | 1 | Vanderwolf et al. (2013a) |
| *Agaricomycetes* | *Agaricales* | *Strophariaceae* | [*Hypholoma radicosum*](https://www.indexfungorum.org/Names/NamesRecord.asp?RecordID=276049) | [*Hypholoma radicosum*](https://www.indexfungorum.org/Names/NamesRecord.asp?RecordID=276049) | Unreported | France | 1 | Vanderwolf et al. (2013a) |
| *Agaricomycetes* | *Agaricales* | *Strophariaceae* | *Hypholoma fasciculare* | *Hypholoma fasciculare* | Unreported, wood | Romania, Slovenia, UK | 3 | Vanderwolf et al. (2013a) |
| *Agaricomycetes* | *Agaricales* | *Strophariaceae* | *Hypholoma* sp. | *Hypholoma* sp. | Wood, unreported | Croatia, UK | 2 | Vanderwolf et al. (2013a) |
| *Agaricomycetes* | *Agaricales* | *Strophariaceae* | *Phloeomana minutula* | *Phloeomana minutula* | Unreported | Unknow |  | Zhang et al. (2021) |
| *Agaricomycetes* | *Agaricales* | *Strophariaceae* | *Pholiota multicingulata* | *Pholiota multicingulata* | Unreported | China | 1 | Zhang et al. (2021) |
| *Agaricomycetes* | *Agaricales* | *Strophariaceae* | *Pholiota* sp. | *Pholiota* sp. | Unreported | USA | 1 | Vanderwolf et al. (2013a) |
| *Agaricomycetes* | *Agaricales* | *Tricholomataceae* | *Tricholoma saponaceum* | *Tricholoma saponaceum* | Cave wall | France | 1 | Vanderwolf et al. (2013a) |
| *Agaricomycetes* | *Agaricales* | *Tricholomataceae* | *Tricholomopsis aurea* | *Tricholomopsis aurea* | Bat guano | Puerto Rico | 1 | Vanderwolf et al. (2013a) |
| *Agaricomycetes* | *Agaricales* | *Tubariaceae* | *Phaeomarasmius* sp. | *Phaeomarasmius* sp. | Unreported | Austria | 1 | Vanderwolf et al. (2013a) |
| *Agaricomycetes* | *Agaricales* | *Tubariaceae* | *Tubaria furfuracea* | *Tubaria furfuracea* | Unreported, wood | Germany | 1 | Vanderwolf et al. (2013a) |
| *Agaricomycetes* | *Agaricales* |  | Agaricales sp. | Agaricales sp. | Unreported | Thailand | 1 | Suetrong et al. (2023) |
| *Agaricomycetes* | *Agaricales* |  | Agaricomycetes | *Agaricales* | Wood | UK | 1 | Vanderwolf et al. (2013a) |
| *Agaricomycetes* | *Amylocorticiales* | *Amylocorticiaceae* | *Agroathelia rolfsii* | *Athelia rolfsii* | Unreported | Poland | 1 | Pusz et al. (2018b) |
| *Agaricomycetes* | *Amylocorticiales* | *Amylocorticiaceae* | *Hypochnus mollis* | *Hypochnicellum molle* | Wood | USA | 1 | Held et al. (2020) |
| *Agaricomycetes* | *Auriculariales* | *Auriculariaceae* | *Auricularia auricula-judae* | *Auricularia auricula-judae* | Sediment, soil | Puerto Rico | 1 | Vanderwolf et al. (2013a) |
| *Agaricomycetes* | *Auriculariales* | *Auriculariaceae* | *Auricularia fuscosuccinea* | *Auricularia fuscosuccinea* | Bat guano | Puerto Rico | 1 | Vanderwolf et al. (2013a) |
| *Agaricomycetes* | *Auriculariales* | *Auriculariaceae* | *Auricularia nigricans* | *Auricularia polytricha* | Bat guano | Puerto Rico | 1 | Vanderwolf et al. (2013a) |
| *Agaricomycetes* | *Auriculariales* | *Auriculariaceae* | Elmerina caryae | *Elmerina caryae* | Wood | Romania | 1 | Vanderwolf et al. (2013a) |
| *Agaricomycetes* | *Auriculariales* | *Auriculariaceae* | *Exidia glandulosa* | *Exidia glandulosa* | Unreported | Antarctica | 1 | Connell and Staudigel (2013) |
| *Agaricomycetes* | *Auriculariales* | *Incertae sedis* | *Elmerina caryae* | *Protomerulius caryae* | Wood | Romania | 1 | Vanderwolf et al. (2013a) |
| *Agaricomycetes* | *Boletales* | *Boletaceae* | *Boletus* sp. | *Boletus* sp. | Air | Spain | 1 | Vanderwolf et al. (2013a) |
| *Agaricomycetes* | *Boletales* | *Coniophoraceae* | *Coniophora puteana* | *Coniophora cerebella* | Wood, air | Australia, Croatia, Slovenia | 3 | Vanderwolf et al. (2013a) |
| *Agaricomycetes* | *Boletales* | *Coniophoraceae* | *Coniophora puteana* | *Coniophora puteana* | Soil, wood | China, Romania, UK | 3 | Vanderwolf et al. (2013a), |
| *Agaricomycetes* | *Boletales* | *Coniophoraceae* | *Coniophora* sp. | *Coniophora* sp. | Wood | Czech Republic | 1 | Vanderwolf et al. (2013a) |
| *Agaricomycetes* | *Boletales* | *Hygrophoropsidaceae* | *Leucogyrophana mollusca* | *Leucogyrophana mollusca* | Unreported | France, Italy | 2 | Vanderwolf et al. (2013a) |
| *Agaricomycetes* | *Boletales* | *Paxillaceae* | *Paxillus* sp. | *Paxillus* sp. | Unreported | Croatia | 1 | Vanderwolf et al. (2013a) |
| *Agaricomycetes* | *Boletales* | *Sclerodermataceae* | *Scleroderma* sp. | *Scleroderma* sp. | Air, unreported | Croatia, Spain | 2 | Vanderwolf et al. (2013a) |
| *Agaricomycetes* | *Boletales* | *Serpulaceae* | *Serpula himantioides* | *Serpula himantioides* | Wood | Germany | 1 | Vanderwolf et al. (2013a) |
| *Agaricomycetes* | *Boletales* | *Serpulaceae* | *Serpula lacrymans* | *Serpula lacrymans* | Unreported, wood | Czech Republic, Switzerland, UK | 3 | Vanderwolf et al. (2013a) |
| *Agaricomycetes* | *Boletales* | *Tapinellaceae* | *Tapinella panuoides* | *Tapinella panuoides* | Unreported, wood | Austria, Bosnia, Croatia, Poland, Romania, Slovenia, USA | 7 | Vanderwolf et al. (2013a) |
| *Agaricomycetes* | *Cantharellales* | *Botryobasidiaceae* | Alysidium sp. | Alysidium sp. | Air | Spain | 1 | Vanderwolf et al. (2013a) |
| *Agaricomycetes* | *Cantharellales* | *Ceratobasidiaceae* | *Ceratobasidium* sp. | *Ceratobasidium* sp. | Air | Spain | 1 | Vanderwolf et al. (2013a) |
| *Agaricomycetes* | *Cantharellales* | *Ceratobasidiaceae* | *Rhizoctonia solani* | *Rhizoctonia solani* | Unreported | Poland | 1 | Pusz et al. (2018a) |
| *Agaricomycetes* | *Cantharellales* | *Ceratobasidiaceae* | *Rhizoctonia solani* | *Thanatephorus cucumeris* | Sediment, soil | Australia | 1 | Vanderwolf et al. (2013a) |
| *Agaricomycetes* | *Cantharellales* | *Hydnaceae* | *Craterellus minimus* | *Craterellus minimus* | Unreported | Spain | 1 | Vanderwolf et al. (2013a) |
| *Agaricomycetes* | *Cantharellales* | *Hydnaceae* | *Hydnum Spainuliferum* | *Hydnum Spainuliferum* | Wood | Algeria | 1 | Vanderwolf et al. (2013a) |
| *Agaricomycetes* | *Cantharellales* | *Hydnaceae* | *Sistotrema brinkmannii* | *Sistotrema brinkmannii* | Asymptomatic specimens of *Proteus anguinus*, wood | Antarctica, Slovenia, USA | 2 | Connell and Staudigel (2013); Held et al. (2020) |
| *Agaricomycetes* | *Cerrenaceae* | *Cerrenaceae* | *Cerrena unicolor* | *Cerrena unicolor* | Unreported | Canada | 1 | Vanderwolf et al. (2013a) |
| *Agaricomycetes* | *Cerrenaceae* | *Cerrenaceae* | Cerrenaceae sp. | *Cerrenaceae* sp. | Soil-sediment, | Thailand | 1 | Suetrong et al. (2023) |
| *Agaricomycetes* | *Cystofilobasidiales* | *Mrakiaceae* | *Tausonia pullulans* | *Tausonia pullulans* | Soil-sediment, asymptomatic specimens of *Proteus anguinus* | Slovenia, Spain, USA | 3 | Vanderwolf et al. (2013a); Zhang et al. (2014) |
| *Agaricomycetes* | *Geastrales* | *Geastraceae* | *Geastrum minimum* | *Geastrum minimum* | bat guano | Puerto Rico | 1 | Vanderwolf et al. (2013a) |
| *Agaricomycetes* | *Geastrales* | *Geastraceae* | *Geastrum saccatum* | *Geastrum saccatum* | Sediment, soil | Puerto Rico | 1 | Vanderwolf et al. (2013a) |
| *Agaricomycetes* | *Gloeophyllales* | *Gloeophyllaceae* | *Gloeophyllum abietinum* | *Gloeophyllum abietinum* | wood, bat guano | Bosnia, Germany, Romania | 3 | Vanderwolf et al. (2013a) |
| *Agaricomycetes* | *Gloeophyllales* | *Gloeophyllaceae* | *Gloeophyllum odoratum* | *Gloeophyllum odoratum* | Unreported, wood | Romania, Slovenia, USA | 3 | Vanderwolf et al. (2013a) |
| *Agaricomycetes* | *Gloeophyllales* | *Gloeophyllaceae* | *Gloeophyllum sepiarium* | *Gloeophyllum sepiarium* | Wood | Bosnia | 1 | Vanderwolf et al. (2013a) |
| *Agaricomycetes* | *Gloeophyllales* | *Gloeophyllaceae* | *Gloeophyllum* sp. | *Gloeophyllum* sp. | Wood | Czech Republic | 1 | Vanderwolf et al. (2013a) |
| *Agaricomycetes* | *Gloeophyllales* | *Gloeophyllaceae* | *Gloeophyllum trabeum* | *Gloeophyllum trabeum* | Wood | Poland | 1 | Vanderwolf et al. (2013a) |
| *Agaricomycetes* | *Gloeophyllales* | *Gloeophyllaceae* | *Neolentinus lepideus* | *Neolentinus suffrutescens* | Unreported, wood | Bosnia, Czech Republic, Germany, UK | 4 | Vanderwolf et al. (2013a) |
| *Agaricomycetes* | *Gomphales* | *Gomphaceae* | *Ramaria* sp. | *Ramaria* sp. | Sediment, soil, bat guano | Puerto Rico | 1 | Vanderwolf et al. (2013a) |
| *Agaricomycetes* | *Hymenochaetales* | *Chaetoporellaceae* | *Kneiffiella floccosa* | *Hyphodontia floccosa* | Wood | USA | 1 | Held et al. (2020) |
| *Agaricomycetes* | *Hymenochaetales* | *Hymenochaetaceae* | *Fomitiporia punctata* | *Phellinus punctatus* | Wood | Romania | 1 | Vanderwolf et al. (2013a) |
| *Agaricomycetes* | *Hymenochaetales* | *Hymenochaetaceae* | *Fuscoporia ferruginosa* | *Phellinus ferruginosus* | Wood | Romania | 1 | Vanderwolf et al. (2013a) |
| *Agaricomycetes* | *Hymenochaetales* | *Hymenochaetaceae* | *Fuscoporia gilva* | *Fuscoporia gilva* | Bat guano | Puerto Rico | 1 | Vanderwolf et al. (2013a) |
| *Agaricomycetes* | *Hymenochaetales* | *Hymenochaetaceae* | *Fuscoporia gilva* | *Phellinus gilvus* | Unreported | Unknow |  | Zhang et al. (2021) |
| *Agaricomycetes* | *Hymenochaetales* | *Hymenochaetaceae* | *Galerina* spp. | *Galerina* spp. | Wood | Czech Republic, Germany | 2 | Vanderwolf et al. (2013a) |
| *Agaricomycetes* | *Hymenochaetales* | *Hymenochaetaceae* | *Hydnoporia corrugata* | *Hymenochaete corrugata* | Unreported | France | 1 | Vanderwolf et al. (2013a) |
| *Agaricomycetes* | *Hymenochaetales* | *Hymenochaetaceae* | *Hymenochaete* sp. | *Hymenochaete* sp. | Unreported | Antarctica | 1 | Connell and Staudigel (2013) |
| *Agaricomycetes* | *Hymenochaetales* | *Hymenochaetaceae* | *Onnia tomentosa* | *Onnia tomentosa* | Wood | Croatia | 1 | Vanderwolf et al. (2013a) |
| *Agaricomycetes* | *Hymenochaetales* | *Hymenochaetaceae* | *Pseudoinonotus dryadeus* | *Inonotus dryadeus* | Air | Australia | 1 | Vanderwolf et al. (2013a) |
| *Agaricomycetes* | *Hymenochaetales* | *Hymenochaetaceae* | *Pseudoinonotus dryadeus* | *Pseudoinonotus dryadeus* | Air | Australia | 1 | Vanderwolf et al. (2013a) |
| *Agaricomycetes* | *Hymenochaetales* | *Hymenochaetaceae* | *Pyrrhoderma noxium* | *Pyrrhoderma noxium* | Water | Malaysia | 1 | Wasti et al. (2021) |
| *Agaricomycetes* | *Hymenochaetales* | *Hymenochaetaceae* | Pyrrhoderma sp. | *Pyrrhoderma* sp. | Organic litter | Thailand | 1 | Suetrong et al. (2023) |
| *Agaricomycetes* | *Hymenochaetales* | *Hyphodontiaceae* | *Hastodontia hastata* | *Hyphodontia hastata* | Wood | Germany | 1 | Vanderwolf et al. (2013a) |
| *Agaricomycetes* | *Hymenochaetales* | *Hyphodontiaceae* | *Hyphodontia arguta* | *Hyphodontia arguta* | Wood | France | 1 | Vanderwolf et al. (2013a) |
| *Agaricomycetes* | *Hymenochaetales* | *Incertae sedis* | Trichaptum sp. | *Trichaptum* sp. | Unreported | Antarctica, Thailand | 1 | Connell and Staudigel (2013); Suetrong et al. (2023) |
| *Agaricomycetes* | *Hymenochaetales* | *Rickenellaceae* | *Resinicium bicolor* | *Resinicium bicolor* | Unreported | Antarctica | 1 | Connell and Staudigel (2013) |
| *Agaricomycetes* | *Hymenochaetales* | *Schizoporaceae* | *Schizopora paradoxa* | *Schizopora paradoxa* | Wood, unreported | Brazil, Romania | 2 | Vanderwolf et al. (2013a) |
| *Agaricomycetes* | *Hymenochaetales* | *Schizoporaceae* | *Xylodon flaviporus* | *Xylodon flaviporus* | Asymptomatic specimens of *Proteus anguinus* | Slovenia | 1 | Savković et al. (2025) |
| *Agaricomycetes* | *Hymenochaetales* | *Schizoporaceae* | *Xylodon rimosissimus* | *Xylodon rimosissimus* | Unreported | Antarctica | 1 | Connell and Staudigel (2013) |
| *Agaricomycetes* | *Incertae sedis* | *Incertae sedis* | *Bridgeoporus nobilissimus* | *Bridgeoporus nobilissimus* | Wood | Germany | 1 | Vanderwolf et al. (2013a) |
| *Agaricomycetes* | *Jaapiales* | *Jaapiaceae* | *Jaapia argillacea* | *Jaapia argillacea* | Wood | USA | 1 | Held et al. (2020) |
| *Agaricomycetes* | *Pleosporales* | *Incertae sedis* | *Climacocystis borealis* | *Climacocystis borealis* | Wood | Switzerland | 1 | Vanderwolf et al. (2013a) |
| *Agaricomycetes* | *Polyporales* | *Cerrenaceae* | *Lividopora vincta* | *Rigidoporus vinctus* | Unreported | China | 1 | Zhang (2019) |
| *Agaricomycetes* | *Polyporales* | *Dacryobolaceae* | *Amaropostia stiptica* | *Postia stiptica* | Wood | Croatia, Czech Republic, Romania | 3 | Vanderwolf et al. (2013a) |
| *Agaricomycetes* | *Polyporales* | *Dacryobolaceae* | *Osteina obducta* | *Osteina obducta* | Wood | Czech Republic | 1 | Vanderwolf et al. (2013a) |
| *Agaricomycetes* | *Polyporales* | *Dacryobolaceae* | *Postia balsamea* | *Postia balsamea* | Wood, air | Germany, Australia | 2 | Vanderwolf et al. (2013a) |
| *Agaricomycetes* | *Polyporales* | *Dacryobolaceae* | *Spongiporus floriformis* | *Postia floriformis* | Wood | Croatia, USA | 2 | Vanderwolf et al. (2013a); Held et al. (2020) |
| *Agaricomycetes* | *Polyporales* | *Fibroporiaceae* | *Fibroporia vaillantii* | *Fibroporia vaillantii* | Wood, cave wall | Bosnia, Croatia, Poland, Romania, Slovenia, UK | 6 | Vanderwolf et al. (2013a) |
| *Agaricomycetes* | *Polyporales* | *Fomitopsidaceae* | *Antrodia sinuosa* | *Amyloporia sinuosa* | Cave wall | UK | 1 | Vanderwolf et al. (2013a) |
| *Agaricomycetes* | *Polyporales* | *Fomitopsidaceae* | *Antrodia sinuosa* | *Antrodia sinuosa* | Cave wall | UK | 1 | Vanderwolf et al. (2013a) |
| *Agaricomycetes* | *Polyporales* | *Fomitopsidaceae* | *Daedalea xantha* | *Antrodia xantha* | Wood | Czech Republic | 1 | Vanderwolf et al. (2013a) |
| *Agaricomycetes* | *Polyporales* | *Fomitopsidaceae* | *Fomitopsis pinicola* | *Fomitopsis pinicola* | Unreported, wood | Austria, Czech Republic, Germany | 3 | Vanderwolf et al. (2013a) |
| *Agaricomycetes* | *Polyporales* | *Fomitopsidaceae* | *Fomitopsis pulvina* | *Buglossoporus pulvinus* | Wood | Czech Republic | 1 | Vanderwolf et al. (2013a) |
| *Agaricomycetes* | *Polyporales* | *Fomitopsidaceae* | *Fomitopsis quercina* | *Daedalea quercina* | Wood | UK | 1 | Vanderwolf et al. (2013a) |
| *Agaricomycetes* | *Polyporales* | *Fomitopsidaceae* | *Neoantrodia serialis* | *Antrodia serialis* | Wood | Austria, Bosnia, Croatia, Czech Republic, Poland, Romania | 6 | Vanderwolf et al. (2013a) |
| *Agaricomycetes* | *Polyporales* | *Fomitopsidaceae* | *Neoantrodia serialis* | *Neoantrodia serialis* | wood | Austria, Bosnia, Croatia, Czech Republic, Poland, Romania | 6 | Vanderwolf et al. (2013a) |
| *Agaricomycetes* | *Polyporales* | *Fomitopsidaceae* | *Resinoporia crassa* | *Antrodia crassa* | Wood | Slovenia | 1 | Vanderwolf et al. (2013a) |
| *Agaricomycetes* | *Polyporales* | *Fomitopsidaceae* | *Resinoporia crassa* | *Resinoporia crassa* | wood | Slovenia | 1 | Vanderwolf et al. (2013a) |
| *Agaricomycetes* | *Polyporales* | *Fomitopsidaceae* | *Rhodofomes roseus* | *Fomitopsis rosea* | Wood | Bosnia, Poland, Romania | 3 | Vanderwolf et al. (2013a) |
| *Agaricomycetes* | *Polyporales* | *Fomitopsidaceae* | *Rhodofomes roseus* | *Rhodofomes roseus* | Unreported | Unknow |  | Zhang et al. 2021 |
| *Agaricomycetes* | *Polyporales* | *Incertae sedis* | *Gloeohypochnicium analogum* | *Hypochnicium analogum* | Wood, sediment, soil | Brazil | 1 | Vanderwolf et al. (2013a) |
| *Agaricomycetes* | *Polyporales* | *Incertae sedis* | *Neohypochnicium punctulatum* | *Hypochnicium punctulatum* | Wood, sediment, soil | Brazil | 1 | Vanderwolf et al. (2013a) |
| *Agaricomycetes* | *Polyporales* | *Incertae sedis* | *Phanerodontia chrysosporium* | *Phanerodontia chrysosporium* | Unreported | Unknow |  | Zhang et al. (2021) |
| *Agaricomycetes* | *Polyporales* | *Incertae sedis* | *Rhodonia placenta* | *Rhodonia placenta* | Air | Australia | 1 | Vanderwolf et al. (2013a) |
| *Agaricomycetes* | *Polyporales* | *Incrustoporiaceae* | *Incrustoporia chrysella* | *Skeletocutis chrysella* | Unreported | Antarctica | 1 | Connell and Staudigel (2013) |
| *Agaricomycetes* | *Polyporales* | *Irpicaceae* | *Byssomerulius corium* | *Byssomerulius corium* | Unreported | Unknow |  | Zhang et al. (2021) |
| *Agaricomycetes* | *Polyporales* | *Irpicaceae* | *Ceriporia* sp. | *Ceriporia* sp. | Air | Thailand | 1 | Suetrong et al. (2023) |
| *Agaricomycetes* | *Polyporales* | *Irpicaceae* | *Cyanosporus caesius* | *Leptoporus caesius* f. *monstrosus* | Wood | Germany | 1 | Vanderwolf et al. (2013a) |
| *Agaricomycetes* | *Polyporales* | *Irpicaceae* | *Flavodon flavus* | *Flavodon flavus* | Unreported | China | 1 | Zhang et al. (2021) |
| *Agaricomycetes* | *Polyporales* | *Irpicaceae* | *Hydnopolyporus palmatus* | *Hydnopolyporus palmatus* | Wood | Brazil | 1 | Vanderwolf et al. (2013a) |
| *Agaricomycetes* | *Polyporales* | *Irpicaceae* | *Irpex laceratus* | *Ceriporia lacerata* | Unreported | China | 1 | Man et al. (2015); Man (2017) |
| *Agaricomycetes* | *Polyporales* | *Irpicaceae* | *Irpex lacteus* | *Irpex cf. lacteus* | Air | Brazil | 1 | Cunha et al. (2020) |
| *Agaricomycetes* | *Polyporales* | *Irpicaceae* | *Irpex lacteus* | *Irpex lacteus* | Sediment | Antarctica, China | 2 | Connell and Staudigel (2013); Man (2017) |
| *Agaricomycetes* | *Polyporales* | *Irpicaceae* | *Irpex* sp. | *Irpex* sp. | Air | Brazil | 1 | Savković et al. (2025) |
| *Agaricomycetes* | *Polyporales* | *Irpicaceae* | *Physisporinus vitreus* | *Leptoporus undatus* f. *adiposus* | Wood | Czech Republic | 1 | Vanderwolf et al. (2013a) |
| *Agaricomycetes* | *Polyporales* | *Irpicaceae* | *Physisporinus vitreus* | *Leptoporus undatus* f. *tuberosa* | Wood | Czech Republic | 1 | Vanderwolf et al. (2013a) |
| *Agaricomycetes* | *Polyporales* | *Irpicaceae* | *Spongiporus floriformis* | *Leptoporus floriformis* f. *subterranea* | Wood | Czech Republic | 1 | Vanderwolf et al. (2013a) |
| *Agaricomycetes* | *Polyporales* | *Meripilaceae* | *Meripilus sanguinolentus* | *Rigidoporus sanguinolentus* | Bat guano | Poland | 1 | Vanderwolf et al. (2013a) |
| *Agaricomycetes* | *Polyporales* | *Meripilaceae* | *Meripilus vitreus* | *Physisporinus vitreus* | Wood | China, CzechA Republic | 2 | Vanderwolf et al. (2013a); Zhang et al. (2021) |
| *Agaricomycetes* | *Polyporales* | *Meripilaceae* | *Physisporinus vitreus* | *Physisporinus vitreus* f. *pergameneus* | Wood | Czech Republic | 1 | Vanderwolf et al. (2013a) |
| *Agaricomycetes* | *Polyporales* | *Meripilaceae* | *Physisporinus vitreus* | *Rigidoporus vitreus* | Wood, cave wall, wood | Croatia, Czech Republic, Poland, Romania, UK | 5 | Vanderwolf et al. (2013a) |
| *Agaricomycetes* | *Polyporales* | *Meripilaceae* | *Rigidoporus lineatus* | *Rigidoporus lineatus* | Air | Australia | 1 | Vanderwolf et al. (2013a) |
| *Agaricomycetes* | *Polyporales* | *Meripilaceae* | *Rigidoporus microporus* | *Rigidoporus microporus* | Wood, bat guano | Puerto Rico | 1 | Vanderwolf et al. (2013a) |
| *Agaricomycetes* | *Polyporales* | *Meripilaceae* | Rigidoporus spp. | *Rigidoporus* spp. | Bat guano | Brazil, China | 2 | Zhang et al. (2017); Cunha et al. (2020) |
| *Agaricomycetes* | *Polyporales* | *Meripilaceae* | *Rigidoporus undatus* | *Rigidoporus undatus* | Wood | Czech Republic | 1 | Vanderwolf et al. (2013a) |
| *Agaricomycetes* | *Polyporales* | *Meruliaceae* | *Byssomerulius corium* | *Merulius papyrinus* | Unreported | Belgium | 1 | Vanderwolf et al. (2013a) |
| *Agaricomycetes* | *Polyporales* | *Meruliaceae* | *Ceriporiopsis subvermispor* | *Ceriporiopsis subvermispor* | Unreported | Antarctica | 1 | Connell and Staudigel (2013) |
| *Agaricomycetes* | *Polyporales* | *Meruliaceae* | *Merulius melanoceras* | *Merulius melanoceras* | Unreported | France, Italy | 2 | Vanderwolf et al. (2013a) |
| *Agaricomycetes* | *Polyporales* | *Meruliaceae* | *Merulius* sp. | *Merulius* sp. | Wood | CzechA Republic, UK | 2 | Vanderwolf et al. (2013a) |
| *Agaricomycetes* | *Polyporales* | *Meruliaceae* | *Mycoacia livida* | *Phlebia livida* | Unreported | China | 1 | Zhang et al. (2021) |
| *Agaricomycetes* | *Polyporales* | *Meruliaceae* | *Phlebia acerina* | *Phlebia acerina* | Air | Spain | 1 | Savković et al. (2025) |
| *Agaricomycetes* | *Polyporales* | *Meruliaceae* | *Phlebia rufa* | *Phlebia rufa* | Unreported | China | 1 | Zhang et al. (2021) |
| *Agaricomycetes* | *Polyporales* | *Meruliaceae* | *Phlebia tremellosa* | *Phlebia tremellosa* | Unreported | France, Italy | 2 | Vanderwolf et al. (2013a) |
| *Agaricomycetes* | *Polyporales* | *Panaceae* | *Panus neostrigosus* | *Panus neostrigosus* | Unreported | Unknow |  | Zhang et al. (2021) |
| *Agaricomycetes* | *Polyporales* | *Panaceae* | *Panus* sp. | *Panus* sp. | Unreported | Austria | 1 | Vanderwolf et al. (2013a) |
| *Agaricomycetes* | *Polyporales* | *Phanerochaetaceae* | *Bjerkandera adusta* | *Bjerkandera adusta* | Air, wood, sediment, asymptomatic specimens of Proteus anguinus | China, Germany, Slovakia, Slovenia, Spain | 5 | Vanderwolf et al. (2013a); Man (2017) |
| *Agaricomycetes* | *Polyporales* | *Phanerochaetaceae* | *Hyphodermella corrugata* | *Hyphodermella corrugata* | Unreported | China | 1 | Zhang et al. (2017) |
| *Agaricomycetes* | *Polyporales* | *Phanerochaetaceae* | *Hyphodermella rosae* | *Hyphodermella rosae* | Air | Spain | 1 | Savković et al. (2025) |
| *Agaricomycetes* | *Polyporales* | *Phanerochaetaceae* | Hyphodermella sp. | *Hyphodermella sp.* | Unreported | China | 1 | Zhang et al. (2017) |
| *Agaricomycetes* | *Polyporales* | *Phanerochaetaceae* | *Phaeophlebiopsis peniophoroides* | *Phaeophlebiopsis peniophoroides* | Air | Spain | 1 | Savković et al. (2025) |
| *Agaricomycetes* | *Polyporales* | *Phanerochaetaceae* | Phanerochaetaceae sp. | Phanerochaetaceae sp. | Air | Thailand | 1 | Suetrong et al. (2023) |
| *Agaricomycetes* | *Polyporales* | *Phanerochaetaceae* | *Phanerochaete livescens* | *Phanerochaete livescens* | Air | Spain | 1 | Savković et al. (2025) |
| *Agaricomycetes* | *Polyporales* | *Phanerochaetaceae* | *Phanerochaete sordida* | *Phanerochaete sordida* | Unreported | China | 1 | Zhang et al. (2017) |
| *Agaricomycetes* | *Polyporales* | *Phanerochaetaceae* | Phanerochaete sp. | *Phanerochaete sp.* | Air | Antarctica, Spain | 2 | Connell and Staudigel (2013),Vanderwolf et al. (2013a) |
| *Agaricomycetes* | *Polyporales* | *Phanerochaetaceae* | *Phanerodontia chrysosporium* | *Sporotrichum pruinosum* | Bat guano | France | 1 | Vanderwolf et al. (2013a) |
| *Agaricomycetes* | *Polyporales* | *Phanerochaetaceae* | *Phlebiopsis gigantea* | *Phlebiopsis gigantea* | Air | Slovakia, Slovenia | 2 | Ogórek (2018b) |
| *Agaricomycetes* | *Polyporales* | *Phanerochaetaceae* | Phlebiopsis sp. | *Phlebiopsis* sp. | Unreported | China | 1 | Zhang et al. (2021) |
| *Agaricomycetes* | *Polyporales* | *Phanerochaetaceae* | *Sporotrichum flavissimum* | *Sporotrichum flavissimum* | Insects | USA | 1 | Vanderwolf et al. (2013a) |
| *Agaricomycetes* | *Polyporales* | *Phanerochaetaceae* | *Sporotrichum* sp. | *Sporotrichum* sp. | Air, bat guano, unreported, wood | Canada, China, Czech Republic, France, USA | 5 | Vanderwolf et al. (2013a) |
| *Agaricomycetes* | *Polyporales* | *Podoscyphaceae* | *Abortiporus biennis* | *Abortiporus biennis* | Wood, water | Romania, Spain | 2 | Vanderwolf et al. (2013a) |
| *Agaricomycetes* | *Polyporales* | *Podoscyphaceae* | *Abortiporus* sp. | *Abortiporus* sp. | Unreported | Spain | 1 | Busquets et al. (2014) |
| *Agaricomycetes* | *Polyporales* | *Podoscyphaceae* | *Podoscypha* sp. | *Podoscypha* sp. | Bat guano | Puerto Rico | 1 | Vanderwolf et al. (2013a) |
| *Agaricomycetes* | *Polyporales* | *Polyporaceae* | *Cerioporus varius* | *Cerioporus varius* | Wood | Germany | 1 | Vanderwolf et al. (2013a) |
| *Agaricomycetes* | *Polyporales* | *Polyporaceae* | *Cerioporus varius* | *Polyporus varius* | Wood | Germany | 1 | Vanderwolf et al. (2013a) |
| *Agaricomycetes* | *Polyporales* | *Polyporaceae* | *Cerrena hydnoides* | *Hexagonia hydnoides* | Bat guano | Puerto Rico | 1 | Vanderwolf et al. (2013a) |
| *Agaricomycetes* | *Polyporales* | *Polyporaceae* | *Coriolopsis gallica* | *Coriolopsis gallica* | Wood | Croatia | 1 | Vanderwolf et al. (2013a) |
| *Agaricomycetes* | *Polyporales* | *Polyporaceae* | *Cyanosporus caesius* | *Postia caesia* | Unreported | Unknow |  | Zhang et al. (2021) |
| *Agaricomycetes* | *Polyporales* | *Polyporaceae* | *Donkioporia expansa* | *Donkioporia expansa* | Wood | Slovenia | 1 | Vanderwolf et al. (2013a) |
| *Agaricomycetes* | *Polyporales* | *Polyporaceae* | *Favolus tenuiculus* | *Favolus tenuiculus* | Wood | Puerto Rico | 1 | Vanderwolf et al. (2013a) |
| *Agaricomycetes* | *Polyporales* | *Polyporaceae* | *Fomes fomentarius* | *Fomes fomentarius* | Air, wood | Croatia, Romania, Slovakia, Slovenia | 4 | Vanderwolf et al. (2013a) |
| *Agaricomycetes* | *Polyporales* | *Polyporaceae* | *Fomes* sp. | *Fomes* sp. | Unreported | USA | 1 | Vanderwolf et al. (2013a) |
| *Agaricomycetes* | *Polyporales* | *Polyporaceae* | *Ganoderma applanatum* | *Ganoderma applanatum* | Unreported | Antarctica | 1 | Connell and Staudigel (2013) |
| *Agaricomycetes* | *Polyporales* | *Polyporaceae* | *Ganoderma australe* | *Ganoderma australe* | Speleothem | Malaysia | 1 | Wasti et al. (2021) |
| *Agaricomycetes* | *Polyporales* | *Polyporaceae* | *Ganoderma carnosum* | *Ganoderma carnosum* | Unreported | China | 1 | Zhang et al. (2021) |
| *Agaricomycetes* | *Polyporales* | *Polyporaceae* | *Ganoderma gibbosum* | *Ganoderma gibbosum* | Unreported | China | 1 | Zhang et al. (2017) |
| *Agaricomycetes* | *Polyporales* | *Polyporaceae* | *Ganoderma lipsiense* | *Ganoderma lipsiense* | Wood | Slovenia, USA | 2 | Vanderwolf et al. (2013a) |
| *Agaricomycetes* | *Polyporales* | *Polyporaceae* | *Ganoderma lucidum* | *Ganoderma lucidum* | Wood | Croatia, Romania | 2 | Vanderwolf et al. (2013a) |
| *Agaricomycetes* | *Polyporales* | *Polyporaceae* | *Ganoderma resinaceum* | *Ganoderma resinaceum* | Bat guano, wood | Puerto Rico, Romania | 2 | Vanderwolf et al. (2013a) |
| *Agaricomycetes* | *Polyporales* | *Polyporaceae* | *Ganoderma* sp. | *Ganoderma* sp. | Air | Spain | 1 | Vanderwolf et al. (2013a) |
| *Agaricomycetes* | *Polyporales* | *Polyporaceae* | *Heterobasidion annosum* | *Fomes annosus f. scutatus* | Wood | Bosnia | 1 | Vanderwolf et al. (2013a) |
| *Agaricomycetes* | *Polyporales* | *Polyporaceae* | *Heterobasidion annosum* | *Fomes cryptarum* | Wood | Croatia, Romania | 2 | Vanderwolf et al. (2013a) |
| *Agaricomycetes* | *Polyporales* | *Polyporaceae* | *Lentinus* sp. | *Lentinus* sp. | Wood | Czech Republic | 1 | Vanderwolf et al. (2013a) |
| *Agaricomycetes* | *Polyporales* | *Polyporaceae* | *Lenzites betulinus* | *Lenzites betulina* | Wood | Bosnia, Croatia, Romania, Slovenia | 4 | Vanderwolf et al. (2013a) |
| *Agaricomycetes* | *Polyporales* | *Polyporaceae* | *Neoantrodia serialis* | *Trametes serialis* f. *corallopoda* | Wood | Romania | 1 | Vanderwolf et al. (2013a) |
| *Agaricomycetes* | *Polyporales* | *Polyporaceae* | *Neoantrodia serialis* | *Trametes serialis* f. *ptychogaster* | Wood | Romania | 1 | Vanderwolf et al. (2013a) |
| *Agaricomycetes* | *Polyporales* | *Polyporaceae* | *Neoantrodia serialis* | *Trametes serialis* f. *tuberosa* | Wood | Romania | 1 | Vanderwolf et al. (2013a) |
| *Agaricomycetes* | *Polyporales* | *Polyporaceae* | *Neoantrodia serialis* | *Trametes serialis* var. *resupinata* | Wood | Romania | 1 | Vanderwolf et al. (2013a) |
| *Agaricomycetes* | *Polyporales* | *Polyporaceae* | *Panus neostrigosus* | *Lentinus strigosus* | Wood | Slovenia | 1 | Vanderwolf et al. (2013a) |
| *Agaricomycetes* | *Polyporales* | *Polyporaceae* | *Physisporinus vitreus* | *Polyporus adiposus* | Wood | UK | 1 | Vanderwolf et al. (2013a) |
| *Agaricomycetes* | *Polyporales* | *Polyporaceae* | *Podofomes mollis* | *Cerioporus mollis* | Unreported | China | 1 | Zhang et al. (2017) |
| *Agaricomycetes* | *Polyporales* | *Polyporaceae* | Polyporus spp. | *Polyporus* spp. | Air, bat guano, wood | Antarctica, Puerto Rico, Thailand, UK | 4 | Connell and Staudigel (2013),Vanderwolf et al. (2013a); Suetrong et al. (2023) |
| *Agaricomycetes* | *Polyporales* | *Polyporaceae* | *Polyporus venetus* | *Polyporus venetus* | Wood | Italy | 1 | Vanderwolf et al. (2013a) |
| *Agaricomycetes* | *Polyporales* | *Polyporaceae* | *Poria* sp. | *Poria* sp. | Wood | Czech Republic | 1 | Vanderwolf et al. (2013a) |
| *Agaricomycetes* | *Polyporales* | *Polyporaceae* | *Porogramme epimiltina* | *Tinctoporellus epimiltinus* | Unreported | China | 1 | Zhang et al. (2017) |
| *Agaricomycetes* | *Polyporales* | *Polyporaceae* | *Rhodofomes roseus* | *Fomes roseus* f. *flabelliformis* | Wood | Romania | 1 | Vanderwolf et al. (2013a) |
| *Agaricomycetes* | *Polyporales* | *Polyporaceae* | *Trametes cubensis* | *Trametes cubensis* | Unreported | Antarctica | 1 | Connell and Staudigel (2013) |
| *Agaricomycetes* | *Polyporales* | *Polyporaceae* | *Trametes gibbosa* | *Trametes gibbosa* | Wood | Croatia, Slovenia | 2 | Vanderwolf et al. (2013a) |
| *Agaricomycetes* | *Polyporales* | *Polyporaceae* | *Trametes hirsuta* | *Trametes hirsuta* | Air, rock surface, Wood, sediment, rock surface | China, Slovenia | 2 | Vanderwolf et al. (2013a); Man et al. (2015); Man (2017); Ogórek et al. ( 2018a) |
| *Agaricomycetes* | *Polyporales* | *Polyporaceae* | *Trametes ochracea* | *Trametes ochracea* | Wood | Romania | 1 | Vanderwolf et al. (2013a) |
| *Agaricomycetes* | *Polyporales* | *Polyporaceae* | *Trametes pubescens* | *Trametes pubescens* | Wood | Bosnia, Canada | 2 | Vanderwolf et al. (2013a) |
| *Agaricomycetes* | *Polyporales* | *Polyporaceae* | *Trametes* sp. | *Trametes* sp. | Bat guano | Puerto Rico | 1 | Vanderwolf et al. (2013a) |
| *Agaricomycetes* | *Polyporales* | *Polyporaceae* | *Trametes trogii* | *Coriolopsis trogii* | Air | China | 1 | Vanderwolf et al. (2013a) |
| *Agaricomycetes* | *Polyporales* | *Polyporaceae* | *Trametes versicolor* | *Trametes versicolor* | Air, wood | Bosnia, China, Croatia, France, Germany, Romania, Slovenia, Spain, UK | 9 | Vanderwolf et al. (2013a); Zhang et al. 2017 |
| *Agaricomycetes* | *Polyporales* | *Polyporaceae* | *Trametes villosa* | *Trametes villosa* | Air | Brazil | 1 | Cunha et al. (2020) |
| *Agaricomycetes* | *Polyporales* | *Steccherinaceae* | *Flaviporus brownii* | *Flaviporus brownii* | Wood | Austria, Croatia, Czech Republic, France, Germany, Slovenia | 6 | Vanderwolf et al. (2013a) |
| *Agaricomycetes* | *Polyporales* | *Steccherinaceae* | *Junghuhnia nitida* | *Junghuhnia nitida* | Wood | Slovenia | 1 | Vanderwolf et al. (2013a) |
| *Agaricomycetes* | *Polyporales* | *Steccherinaceae* | Steccherinum sp. | *Steccherinum* sp. | Unreported | Unknow |  | Zhang (2019) |
| *Agaricomycetes* | *Polyporales* |  | Polyporales sp. | *Polyporales* sp. | Water | Thailand | 1 | Suetrong et al. (2023) |
| *Agaricomycetes* | *Rickenellaceae* | *Rickenellaceae* | *Cotylidia aurantiaca* | *Cotylidia aurantiaca* | Wood | Puerto Rico | 1 | Vanderwolf et al. (2013a) |
| *Agaricomycetes* | *Russulales* | *Bondarzewiaceae* | *Heterobasidion annosum* | *Heterobasidion annosum* | Isopod feces, wood | Austria, Bosnia, Croatia, Czech Republic, Poland, Romania, Slovenia, UK | 8 | Vanderwolf et al. (2013a); Nováková et al. (2018) |
| *Agaricomycetes* | *Russulales* | *Bondarzewiaceae* | *Heterobasidion annosum* | *Spainiger meineckellus* | Cave wall | UK | 1 | Vanderwolf et al. (2013a) |
| *Agaricomycetes* | *Russulales* | *Incertae sedis* | *Gloeohypochnicium analogum* | *Gloeohypochnicium analogum* | Wood, soil-sediment | Brazil | 1 | Vanderwolf et al. (2013a) |
| *Agaricomycetes* | *Russulales* | *Peniophoraceae* | Entomocorticium sp. | *Entomocorticium* sp. | Unreported | Poland | 1 | Pusz et al. (2017) |
| *Agaricomycetes* | *Russulales* | *Peniophoraceae* | *Peniophora cinerea* | *Peniophora cinerea* | Unreported | China | 1 | Zhang et al. (2017) |
| *Agaricomycetes* | *Russulales* | *Peniophoraceae* | *Peniophora incarnata* | *Peniophora incarnata* | Unreported | China | 1 | Zhang (2019) |
| *Agaricomycetes* | *Russulales* | *Peniophoraceae* | *Peniophora lassa* | *Duportella lassa* | Unreported | China | 1 | Zhang et al. (2021) |
| *Agaricomycetes* | *Russulales* | *Peniophoraceae* | *Peniophora limitata* | *Peniophora limitata* | Unreported | China | 1 | Zhang et al. (2017) |
| *Agaricomycetes* | *Russulales* | *Peniophoraceae* | *Peniophora lycii* | *Peniophora lycii* | Air | Antarctica, Spain | 2 | Connell and Staudigel (2013) |
| *Agaricomycetes* | *Russulales* | *Peniophoraceae* | *Peniophora quercina* | *Peniophora quercina* | Unreported | France | 1 | Vanderwolf et al. (2013a) |
| *Agaricomycetes* | *Russulales* | *Peniophoraceae* | Peniophora sp. | *Peniophora* sp. | Unreported | China | 1 | Zhang et al. (2017) |
| *Agaricomycetes* | *Russulales* | *Polyporaceae* | *Perenniporia medulla-panis* | *Perenniporia medulla-panis* | Unreported | China | 1 | Zhang et al. (2021) |
| *Agaricomycetes* | *Russulales* | *Russulaceae* | *Russula* sp. | *Russula* sp. | Air | Spain | 1 | Vanderwolf et al. (2013a) |
| *Agaricomycetes* | *Russulales* | *Stereaceae* | Stereaceae sp. | Stereaceae sp. | Air | Thailand | 1 | Suetrong et al. (2023) |
| *Agaricomycetes* | *Russulales* | *Stereaceae* | *Stereum hirsutum* | *Stereum hirsutum* | Unreported, wood | Bosnia, Croatia, Czech Republic, France, Germany, Slovenia, Spain | 7 | Vanderwolf et al. (2013a) |
| *Agaricomycetes* | *Russulales* | *Stereaceae* | *Stereum sanguinolentum* | *Stereum sanguinolentum* | Air | Antarctica, Spain | 2 | Connell and Staudigel (2013) |
| *Agaricomycetes* | *Russulales* | *Stereaceae* | *Stereum* sp. | *Stereum* sp. | Unreported, wood | Antarctica, France, Germany, USA | 4 | Connell and Staudigel (2013); Vanderwolf et al. (2013a) |
| *Agaricomycetes* | *Russulales* |  | Russulales sp. | Russulales sp. | Unreported | Thailand | 1 | Suetrong et al. (2023) |
| *Agaricomycetes* | *Thelephorales* | *Thelephoraceae* | *Thelephora lapida* | *Tomentella lapida* | Unreported | Unknow |  | Zhang et al. (2021) |
| *Agaricomycetes* | *Thelephorales* | *Thelephoraceae* | *Thelephora penicillata* | *Thelephora penicillata* | Unreported | Unknow |  | Zhang et al. (2021) |
| *Agaricomycetes* | *Thelephorales* | *Thelephoraceae* | *Thelephora penicillata* | *Thelephora spiculosa* | Unreported | Austria | 1 | Vanderwolf et al. (2013a) |
| *Agaricomycetes* | *Thelephorales* | *Thelephoraceae* | *Tomentella lapida* | *Thelephora spongiosa* | Wood | Switzerland | 1 | Vanderwolf et al. (2013a) |
| *Agaricomycetes* | *Thelephorales* | *Thelephoraceae* | *Tomentella* sp. | *Tomentella* sp. | Wood, air | Brazil, Spain | 2 | Vanderwolf et al. (2013a) |
| *Agaricomycetes* | *Trechisporales* | *Hydnodontaceae* | *Xenasmatella alnicola* | *Trechispora alnicola* | Wood | Germany | 1 | Vanderwolf et al. (2013a) |
| *Agaricomycetes* |  |  | Agaricomycetes *sp.* | *Agaricomycetes sp.* | Air, water, soil-sediment, Organic litter, | Thailand | 1 | Suetrong et al. (2023) |
| *Cystobasidiomycetes* | *Cystobasidiales* | *Cystobasidiaceae* | *Cystobasidium minutum* | *Cystobasidium minuta* | Soil-sediment | Italy | 1 | Vanderwolf et al. (2013a) |
| *Cystobasidiomycetes* | *Cystobasidiales* | *Cystobasidiaceae* | *Cystobasidium minutum* | *Rhodotorula minuta* var. *minuta* | Sediment, soil | Italy | 1 | Vanderwolf et al. (2013a) |
| *Cystobasidiomycetes* | *Cystobasidiales* | *Cystobasidiaceae* | *Cystobasidium slooffiae* | *Cystobasidium slooffiae* | Unreported | China | 1 | Zhang (2019) |
| *Cystobasidiomycetes* | *Cystobasidiales* | *Cystofilobasidiaceae* | *Cystofilobasidium macerans* | *Cystofilobasidium macerans* | Symptomatic specimens of *Proteus anguinus* | Antarctica, Slovenia | 2 | Connell and Staudigel (2013) |
| *Cystobasidiomycetes* | *Cystobasidiales* | *Cystofilobasidiaceae* | *Cystofilobasidium* sp. | *Cystofilobasidium* sp. | Unreported | Canada | 1 | Vanderwolf et al. (2013a) |
| *Cystobasidiomycetes* | *Sakaguchiales* | *Sakaguchiaceae* | *Sakaguchia* sp. | *Sakaguchia* sp. | Bat | Brazil | 1 | Cunha et al. (2020) |
| *Dacrymycetes* | *Dacrymycetales* | *Dacrymycetaceae* | *Calocera cornea* | *Calocera cornea* | Wood | USA | 1 | Held et al. (2020) |
| *Exobasidiomycetes* | *Exobasidiales* | *Brachybasidiaceae* | *Meira nashicola* | *Meira nashicola* | Unreported | China | 1 | Zhang et al. (2021) |
| *Exobasidiomycetes* | *Exobasidiales* | *Exobasidiaceae* | *Exobasidium* sp. | *Exobasidium* sp. | Unreported | Antarctica | 1 | Connell and Staudigel (2013) |
| *Exobasidiomycetes* | *Golubeviales* | *Golubeviaceae* | *Golubevia pallescens* | *Golubevia pallescens* | Air | China, Spain | 2 | Zhang et al. (2021) |
| *Exobasidiomycetes* | *Microstromatales* | *Incertae sedis* | *Sympodiomycopsis paphiopedili* | *Sympodiomycopsis paphiopedili* | Air | Brazil | 1 | Alves et al. (2022); Savković et al. (2025) |
| *Exobasidiomycetes* | *Tilletiales* | *Tilletiaceae* | *Tilletia* sp. | *Tilletia* sp. | Air | Spain | 1 | Vanderwolf et al. (2013a) |
| *Malasseziomycetes* | *Malasseziales* | *Malasseziaceae* | *Malassezia furfur* | *Malassezia furfur* | Cave wall | France, Germany | 2 | Vanderwolf et al. (2013a) |
| *Malasseziomycetes* | *Malasseziales* | *Malasseziaceae* | *Malassezia globosa* | *Malassezia globosa* | Unreported | Antarctica | 1 | Connell and Staudigel (2013) |
| *Malasseziomycetes* | *Malasseziales* | *Malasseziaceae* | *Malassezia restricta* | *Malassezia restricta* | Unreported | Antarctica | 1 | Connell and Staudigel (2013) |
| *Microbotryomycetes* | *Incertae sedis* | *Incertae sedis* | *Sampaiozyma ingeniosa* | *Sampaiozyma ingeniosa* | Unreported | China | 1 | Zhang et al. (2021) |
| *Microbotryomycetes* | *Kriegeriales* | *Camptobasidiaceae* | *Glaciozyma antarctica* | *Glaciozyma antarctica* | Soil | Romania | 1 | Brad et al. (2018) |
| *Microbotryomycetes* | *Kriegeriales* | *Camptobasidiaceae* | *Glaciozyma watsonii* | *Glaciozyma watsonii* | Unreported | Antarctica | 1 | Connell and Staudigel (2013) |
| *Microbotryomycetes* | *Leucosporidiales* | *Leucosporidiaceae* | *Leucosporidium fellii* | *Leucosporidium fellii* | Unreported | Canada | 1 | Vanderwolf et al. (2013a) |
| *Microbotryomycetes* | *Microbotryales* | *Microbotryaceae* | Microbotryaceae sp. | Microbotryaceae sp. | Slate precipitate | Germany | 1 | Burow et al. (2019) |
| *Microbotryomycetes* | *Sporidiobolales* | *Sporidiobolaceae* | *Rhodotorula dairenensis* | *Rhodotorula dairenensis* | Cave wall, leaf litter | Mexico, Venezuela | 2 | Vanderwolf et al. (2013a) |
| *Microbotryomycetes* | *Sporidiobolales* | *Sporidiobolaceae* | *Rhodotorula glutinis* | *Rhodotorula glutinis* | Soil-sediment | Italy, Slovakia | 2 | Vanderwolf et al. (2013a); Ogórek et al. (2016b, d) |
| *Microbotryomycetes* | *Sporidiobolales* | *Sporidiobolaceae* | *Rhodotorula glutinis* | *Rhodotorula glutinis var. glutinis* | Sediment, soil | Italy | 1 | Vanderwolf et al. (2013a) |
| *Microbotryomycetes* | *Sporidiobolales* | *Sporidiobolaceae* | *Rhodotorula mucilaginosa* | *Rhodotorula cf. mucilaginosa* | Bat | Brazil | 1 | Cunha et al. (2020) |
| *Microbotryomycetes* | *Sporidiobolales* | *Sporidiobolaceae* | *Rhodotorula mucilaginosa* | *Rhodotorula mucilaginosa* | Soil-sediment | Bahamas, China, Italy | 3 | Connell and Staudigel (2013),Vanderwolf et al. (2013a) |
| *Microbotryomycetes* | *Sporidiobolales* | *Sporidiobolaceae* | *Rhodotorula mucilaginosa* | *Rhodotorula mucilaginosa var. mucilaginosa* | Sediment, soil | Bahamas, Italy | 2 | Vanderwolf et al. (2013a) |
| *Microbotryomycetes* | *Sporidiobolales* | *Sporidiobolaceae* | *Rhodotorula mucilaginosa* | *Rhodotorula rubra* | Air, rock | Poland | 1 | Kokurewicz et al. (2016) |
| *Microbotryomycetes* | *Sporidiobolales* | *Sporidiobolaceae* | *Rhodotorula* sp. | *Rhodotorula* sp. | Air, unreported, sediment, soil, bat guano, insects | Australia, Belgium, Mexico, Spain, USA | 5 | Vanderwolf et al. (2013a) |
| *Microbotryomycetes* | *Sporidiobolales* | *Sporidiobolaceae* | *Sporobolomyces coprosmae* | *Sporobolomyces coprosmae* | Air | France | 1 | Vanderwolf et al. (2013a) |
| *Microbotryomycetes* | *Sporidiobolales* | *Sporidiobolaceae* | *Sporobolomyces roseus* | *Sporobolomyces roseus* | Air, Sediment, soil | France, Italy | 2 | Vanderwolf et al. (2013); Martin-Sanchez et al. (2014) |
| *Microbotryomycetes* | *Sporidiobolales* | *Sporidiobolaceae* | Sporobolomyces sp. | *Sporobolomyces* sp. | Air | Antarctica, France | 2 | Connell and Staudigel (2013); Martin-Sanchez et al. (2014) |
| *Microbotryomycetes* | *Sporidiobolales* | *Sporidiobolaceae* | *Sporotrichum flavissimum* | *Sporobolomyces ruberrimus* | Air | France | 1 | Martin-Sanchez et al. (2014) |
| *Pucciniomycetes* | *Pucciniales* | *Pucciniaceae* | *Puccinia* sp. | *Puccinia* sp. | Air | Spain | 1 | Vanderwolf et al. (2013a) |
| *Sordariomycetes* | *Cephalothecales* | *Cephalothecaceae* | *Victoriomyces antarcticus* | *Victoriomyces antarcticus* | Air | Spain | 1 | Savković et al. (2025) |
| *Tremellomycetes* | *Cystofilobasidiales* | *Mrakiaceae* | *Mrakia blollopis* | *Mrakia blollopis* | Air | Slovakia | 1 | Savković et al. (2025) |
| *Tremellomycetes* | *Cystofilobasidiales* | *Mrakiaceae* | *Mrakia frigida* | *Mrakia frigida* | Soil | Romania | 1 | Brad et al. (2018) |
| *Tremellomycetes* | *Cystofilobasidiales* | *Mrakiaceae* | *Mrakia frigida* | *Mrakia gelida* | Soil | Romania | 1 | Brad et al. (2018) |
| *Tremellomycetes* | *Cystofilobasidiales* | *Mrakiaceae* | *Tausonia pullulans* | *Trichosporon pullulans* | Sediment, soil | Spain | 1 | Vanderwolf et al. (2013a) |
| *Tremellomycetes* | *Filobasidiales* | *Filobasidiaceae* | *Filobasidium floriforme* | *Filobasidium floriforme* | Unreported | Antarctica | 1 | Connell and Staudigel (2013) |
| *Tremellomycetes* | *Filobasidiales* | *Filobasidiaceae* | *Filobasidium magnum* | *Filobasidium magnum* | Unreported | Unknow |  | Zhang et al. (2021) |
| *Tremellomycetes* | *Filobasidiales* | *Filobasidiaceae* | *Filobasidium stepposum* | *Filobasidium stepposum* | Air, asymptomatic specimens of Proteus anguinus | Slovenia, Spain | 2 | Savković et al. (2025) |
| *Tremellomycetes* | *Filobasidiales* | *Filobasidiaceae* | *Filobasidium wieringae* | *Filobasidium wieringae* | Air | Antarctica, Slovakia | 2 | Connell and Staudigel (2013) |
| *Tremellomycetes* | *Filobasidiales* | *Filobasidiaceae* | *Naganishia albida* | *Naganishia albida* | Unreported | Unknow |  | Zhang et al. (2021) |
| *Tremellomycetes* | *Filobasidiales* | *Filobasidiaceae* | *Naganishia diffluens* | *Naganishia diffluens* | Air, soil-sediment | Bahamas, Spain | 2 | Vanderwolf et al. (2013a) |
| *Tremellomycetes* | *Filobasidiales* | *Filobasidiaceae* | *Naganishia randhawae* | *Naganishia randhawae* | Bat guano, leaf litter | China, Mexico | 2 | Savković et al. (2025) |
| *Tremellomycetes* | *Filobasidiales* | *Piskurozymaceae* | *Solicoccozyma terricola* | *Solicoccozyma terricola* | Slate precipitate | Germany | 1 | Burow et al. (2019) |
| *Tremellomycetes* | *Holtermanniales* | *Holtermanniaceae* | *Holtermanniella wattica* | *Holtermanniella wattica* | Bat | USA | 1 | Zhang et al. (2014) |
| *Tremellomycetes* | *Tremellales* | *Bulleraceae* | *Bullera alba* | *Bullera alba* | Air | France | 1 | Martin-Sanchez et al. (2014) |
| *Tremellomycetes* | *Tremellales* | *Bulleribasidiaceae* | *Bulleribasidium variabile* | *Bulleribasidium variabile* | Soil-sediment, Air, bat guano, earthworm casts, wood, | Czech Republic, Poland, Puerto Rico, Romania, Russia, Slovakia, South Africa | 7 | Vanderwolf et al. (2013a) |
| *Tremellomycetes* | *Tremellales* | *Bulleribasidiaceae* | *Bulleribasidium variabile* | *Mingxiaea variabilis* | Bat guano | Italy | 1 | Vanderwolf et al. (2013a) |
| *Tremellomycetes* | *Tremellales* | *Bulleribasidiaceae* | *Hannaella luteola* | *Cryptococcus luteolus* | Sediment, soil | Bahamas | 1 | Vanderwolf et al. (2013a) |
| *Tremellomycetes* | *Tremellales* | *Bulleribasidiaceae* | *Hannaella luteola* | *Hannaella luteola* | Soil-sediment | Bahamas | 1 | Vanderwolf et al. (2013a) |
| *Tremellomycetes* | *Tremellales* | *Bulleribasidiaceae* | *Hannaella oryzae* | *Hannaella oryzae* | Unreported | China | 1 | Zhang et al. (2021) |
| *Tremellomycetes* | *Tremellales* | *Bulleribasidiaceae* | *Vishniacozyma dimennae* | *Vishniacozyma dimennae* | Air | France | 1 | Martin-Sanchez et al. (2014) |
| *Tremellomycetes* | *Tremellales* | *Bulleribasidiaceae* | *Vishniacozyma victoriae* | *Vishniacozyma victoriae* | Air | Slovakia | 1 | Savković et al. (2025) |
| *Tremellomycetes* | *Tremellales* | *Cryphonectriaceae* | *Cryphonectriaceae* sp. | *Cryphonectriaceae* sp. | Soil-sediment, | Thailand | 1 | Suetrong et al. (2023) |
| *Tremellomycetes* | *Tremellales* | *Cryptococcaceae* | *Cryptococcus albidus* | *Cryptococcus albidus* var. *albidus* | Bat internal tissue, sediment, soil | Bahamas, Italy, Mexico | 3 | Vanderwolf et al. (2013a) |
| *Tremellomycetes* | *Tremellales* | *Cryptococcaceae* | *Cryptococcus diffluens* var*. diffluens* | *Cryptococcus diffluens* var. *diffluens* | Sediment, soil | Bahamas | 1 | Vanderwolf et al. (2013a) |
| *Tremellomycetes* | *Tremellales* | *Cryptococcaceae* | *Cryptococcus festucosus* | *Cryptococcus festucosus* | Unreported | China | 1 | Zhang et al. (2021) |
| *Tremellomycetes* | *Tremellales* | *Cryptococcaceae* | *Cryptococcus laurentii* var*. laurentii* | *Cryptococcus laurentii* var. *laurentii* | Sediment, soil, bat guano | Bahamas, Italy | 2 | Vanderwolf et al. (2013a) |
| *Tremellomycetes* | *Tremellales* | *Cryptococcaceae* | *Cryptococcus magnus* var*. magnus* | *Cryptococcus magnus* var. *magnus* | Sediment, soil | Australia | 1 | Vanderwolf et al. (2013a) |
| *Tremellomycetes* | *Tremellales* | *Cryptococcaceae* | *Cryptococcus neoformans* var. *neoformans* | *Cryptococcus neoformans var. neoformans* | Bat guano, sediment, soil, dung, frass (not bat) | Italy, Spain, USA | 3 | Vanderwolf et al. (2013a) |
| *Tremellomycetes* | *Tremellales* | *Cryptococcaceae* | *Cryptococcus* sp. | *Cryptococcus* sp. | Slate precipitate | Germany | 1 | Burow et al. (2019) |
| *Tremellomycetes* | *Tremellales* | *Cryptococcaceae* | *Cryptococcus tephrensis* | *Cryptococcus tephrensis* | Unreported | China | 1 | Zhang (2019) |
| *Tremellomycetes* | *Tremellales* | *Cryptococcaceae* | *Cystofilobasidium macerans* | *Cryptococcus macerans* | Air | South Africa | 1 | Vanderwolf et al. (2013a) |
| *Tremellomycetes* | *Tremellales* | *Cryptococcaceae* | *Debaryomyces hansenii* | *Atelosaccharomyces hudeloi* | Sediment, soil | Italy | 1 | Vanderwolf et al. (2013a) |
| *Tremellomycetes* | *Tremellales* | *Cryptococcaceae* | *Kwoniella dendrophila* | *Kwoniella cf. dendrophila* | Bat | Brazil | 1 | Cunha et al. (2020) |
| *Tremellomycetes* | *Tremellales* | *Cryptococcaceae* | *Saitozyma podzolica* | *Cryptococcus podzolicus* | Bat guano | Japan | 1 | Vanderwolf et al. (2013a) |
| *Tremellomycetes* | *Tremellales* | *Rhynchogastremaceae* | *Papiliotrema flavescens* | *Papiliotrema flavescens* | Unreported | China | 1 | Zhang et al. (2021) |
| *Tremellomycetes* | *Tremellales* | *Rhynchogastremaceae* | *Papiliotrema laurentii* | *Papiliotrema laurentii* | Bat guano, soil-sediment, leaf litter | China, Bahamas, Italy, Mexico | 4 | Vanderwolf et al. (2013a); Zhnag et al. 2021 |
| *Tremellomycetes* | *Tremellales* | *Tremellaceae* | *Phaeotremella* sp. | *Phaeotremella* sp. | Slate precipitate | Germany | 1 | Burow et al. (2019) |
| *Tremellomycetes* | *Tremellales* | *Tremellaceae* | *Tremella mesenterica* | *Tremella mesenterica* | Wood | Croatia, Slovenia | 2 | Vanderwolf et al. (2013a) |
| *Tremellomycetes* | *Tremellales* | *Tremellaceae* | *Tremella* sp. | *Tremella* sp. | Wood | Brazil | 1 | Vanderwolf et al. (2013a) |
| *Tremellomycetes* | *Tremellales* | *Tremellaceae* | *Tulasnella aurantiaca* | *Hormomyces aurantiacus* | Unreported | Canada | 1 | Vanderwolf et al. (2013a) |
| *Tremellomycetes* | *Tremellales* | *Trimorphomycetaceae* | *Saitozyma podzolica* | *Saitozyma podzolica* | Bat internal tissue, soil-sediment | Bahamas, China, Italy, Mexico | 4 | Vanderwolf et al. (2013a) |
| *Tremellomycetes* | *Trichosporonales* | *Trichosporonaceae* | *Apiotrichum akiyoshidainum* | *Apiotrichum akiyoshidainum* | bat gunao | Japan | 1 | Takashima et al. (2020) |
| *Tremellomycetes* | *Trichosporonales* | *Trichosporonaceae* | *Apiotrichum chiropterorum* | *Apiotrichum chiropterorum* | Bat gunao | Japan, mexico | 2 | Takashima et al. (2020) |
| *Tremellomycetes* | *Trichosporonales* | *Trichosporonaceae* | *Apiotrichum coprophilum* | *Apiotrichum coprophilum* | Bat gunao | Japan | 1 | Takashima et al. (2020) |
| *Tremellomycetes* | *Trichosporonales* | *Trichosporonaceae* | *Apiotrichum dehoogii* | *Apiotrichum dehoogii* | Water | China, Germany | 2 | Burow et al. (2019) |
| *Tremellomycetes* | *Trichosporonales* | *Trichosporonaceae* | *Apiotrichum dulcitum* | *Apiotrichum dulcitum* | Water | Canada, China, Germany | 3 | Burow et al. (2019) |
| *Tremellomycetes* | *Trichosporonales* | *Trichosporonaceae* | *Apiotrichum dulcitum* | *Trichosporon dulcitum* | Bat | Canada, USA | 2 | Zhang et al. (2014); Vanderwolf et al. (2015) |
| *Tremellomycetes* | *Trichosporonales* | *Trichosporonaceae* | *Apiotrichum laibachii* | *Apiotrichum laibachii* | Dung, frass (not bat); bat guano, soil | China, Japan, Mexico, Spain | 4 | Vanderwolf et al. (2013a); Zhang et al. (2017) |
| *Tremellomycetes* | *Trichosporonales* | *Trichosporonaceae* | *Apiotrichum laibachii* | *Trichosporon laibachii* | Dung, frass (not bat); bat guano, water | China, Japan, Spain | 3 | Vanderwolf et al. (2013a) |
| *Tremellomycetes* | *Trichosporonales* | *Trichosporonaceae* | *Apiotrichum lignicola* | *Apiotrichum lignicola* | Unreported | Canada | 1 | Vanderwolf et al. (2013a) |
| *Tremellomycetes* | *Trichosporonales* | *Trichosporonaceae* | *Apiotrichum otae* | *Apiotrichum otae* | Bat gunao | Japan | 1 | Takashima et al. (2020) |
| *Tremellomycetes* | *Trichosporonales* | *Trichosporonaceae* | *Apiotrichum* sp. | *Apiotrichum* sp. | Unreported | China | 1 | Zhang et al. (2021) |
| *Tremellomycetes* | *Trichosporonales* | *Trichosporonaceae* | *Asterotremella* sp*.* | *Asterotremella* sp. | Unreported | Canada | 1 | Vanderwolf et al. (2013a) |
| *Tremellomycetes* | *Trichosporonales* | *Trichosporonaceae* | *Cutaneotrichosporon cavernicola* | *Cutaneotrichosporon cavernicola* | Bat guano | Japan | 1 | Vanderwolf et al. (2013a); Zhang et al. 2021 |
| *Tremellomycetes* | *Trichosporonales* | *Trichosporonaceae* | *Cutaneotrichosporon curvatum* | *Cutaneotrichosporon curvatum* | Air | Slovakia, Slovenia, | 1 | Ogórek et al. ( 2018); Ogórek (2018a, b) |
| *Tremellomycetes* | *Trichosporonales* | *Trichosporonaceae* | *Cutaneotrichosporon cutaneum* | *Cutaneotrichosporon cutaneum* | Soil-sediment | Italy | 1 | Vanderwolf et al. (2013a) |
| *Tremellomycetes* | *Trichosporonales* | *Trichosporonaceae* | *Cutaneotrichosporon dermatis* | *Cutaneotrichosporon dermatis* | Unreported | China | 1 | Zhang et al. (2021) |
| *Tremellomycetes* | *Trichosporonales* | *Trichosporonaceae* | *Cutaneotrichosporon jirovecii* | *Cutaneotrichosporon jirovecii* | Unreported | Bulgaria | 1 | Mitova et al. (2017) |
| *Tremellomycetes* | *Trichosporonales* | *Trichosporonaceae* | *Cutaneotrichosporon middelhovenii* | *Cutaneotrichosporon middelhovenii* | Bat guano | Japan | 1 | Takashima et al. (2020) |
| *Tremellomycetes* | *Trichosporonales* | *Trichosporonaceae* | *Cutaneotrichosporon moniliiforme* | *Cutaneotrichosporon moniliiforme* | Water | Germany | 1 | Burow et al. (2019) |
| *Tremellomycetes* | *Trichosporonales* | *Trichosporonaceae* | *Cutaneotrichosporon mucoides* | *Cutaneotrichosporon mucoides* | Cave wall, bat guano, soil-sediment | Italy | 1 | Vanderwolf et al. (2013a) |
| *Tremellomycetes* | *Trichosporonales* | *Trichosporonaceae* | *Cutaneotrichosporon mucoides* | *Trichosporon mucoides* | Cave wall, sediment, soil, bat guano | Italy | 1 | Vanderwolf et al. (2013a) |
| *Tremellomycetes* | *Trichosporonales* | *Trichosporonaceae* | *Cutaneotrichosporon smithiae* | *Cutaneotrichosporon smithiae* | Unreported | China | 1 | Zhang et al. (2021) |
| *Tremellomycetes* | *Trichosporonales* | *Trichosporonaceae* | *Cutaneotrichosporon spelunceum* | *Trichosporon spelunceum* | Bat guano | Czech Republic, Japan, Mexico | 3 | Nováková et al. (2015) |
| *Tremellomycetes* | *Trichosporonales* | *Trichosporonaceae* | *Cutaneotrichosporon moniliiforme* | *Cutaneotrichosporon moniliiforme* | Water | China, Germany | 2 | Burow et al. (2019) |
| *Tremellomycetes* | *Trichosporonales* | *Trichosporonaceae* | *Effuseotrichosporon vanderwaltii* | *Effuseotrichosporon vanderwaltii* | Soil | China | 1 | Zhang et al. (2021) |
| *Tremellomycetes* | *Trichosporonales* | *Trichosporonaceae* | *Prillingera fragicola* | *Vanrija fragicola* | Bat | USA | 1 | Zhang et al. (2014) |
| *Tremellomycetes* | *Trichosporonales* | *Trichosporonaceae* | *Trichosporon aggtelekiense* | *Trichosporon aggtelekiense* | sediment | Czech Republic, Slovakia | 2 | Nováková et al. (2015) |
| *Tremellomycetes* | *Trichosporonales* | *Trichosporonaceae* | *Trichosporon cutaneum var. cutaneum* | *Trichosporon cutaneum* var. *cutaneum* | Sediment, soil | Italy | 1 | Vanderwolf et al. (2013a) |
| *Tremellomycetes* | *Trichosporonales* | *Trichosporonaceae* | *Trichosporon lignicola* | *Trichosporon lignicola* var. *undulatum* | Unreported | Canada | 1 | Vanderwolf et al. (2013a) |
| *Tremellomycetes* | *Trichosporonales* | *Trichosporonaceae* | *Trichosporon ovoides* | *Trichosporon ovoides* | Cave wall | Italy | 1 | Vanderwolf et al. (2013a) |
| *Tremellomycetes* | *Trichosporonales* | *Trichosporonaceae* | *Trichosporon porosum* | *Trichosporon porosum* | Cave wall, bat guano | Bulgaria, Japan, Venezuela | 3 | Vanderwolf et al. (2013); Mitova et al. (2017) |
| *Tremellomycetes* | *Trichosporonales* | *Trichosporonaceae* | *Trichosporon* sp. | *Trichosporon akiyoshidainum* | Dung, frass (not bat) | China, Spain | 2 | Vanderwolf et al. (2013a); Zhang et al. (2017) |
| *Tremellomycetes* | *Trichosporonales* | *Trichosporonaceae* | *Trichosporon* sp. | *Trichosporon cavernicola* | Bat guano | China, Japan | 2 | Vanderwolf et al. (2013a) |
| *Tremellomycetes* | *Trichosporonales* | *Trichosporonaceae* | *Trichosporon* sp. | *Trichosporon chiropterorum* | Unreported | China, Italy | 2 | Vanderwolf et al. (2013a) |
| *Tremellomycetes* | *Trichosporonales* | *Trichosporonaceae* | *Trichosporon* sp. | *Trichosporon coprophilum* | Unreported | Canada | 1 | Vanderwolf et al. (2013a) |
| *Tremellomycetes* | *Trichosporonales* | *Trichosporonaceae* | *Trichosporon* sp*.* | *Trichosporon otae* | Water, precipitate | Germany | 1 | Burow et al. (2019); Zhang et al. (2021) |
| *Tremellomycetes* | *Trichosporonales* | *Trichosporonaceae* | *Trichosporon* sp*.* | *Trichosporon shinodae* | Unreported | China | 1 | Zhang et al. (2021) |
| *Tremellomycetes* | *Trichosporonales* | *Trichosporonaceae* | *Trichosporon* sp*.* | *Trichosporon* sp*.* | Water, sediment | Bulgaria, Germany, Romania | 3 | Zhang et al. (2021) |
| *Tremellomycetes* | *Trichosporonales* | *Trichosporonaceae* | *Trichosporon* spp. | *Trichosporon* spp. | Soil-sediment | Canada, China, Germany, Japan, Mexico, Romania, Spain, USA, Venezuela | 9 | Vanderwolf et al. (2013, 2015, 2019); Man (2017); Burow et al. (2019); Zhang et al. (2021) |
| *Tremellomycetes* | *Trichosporonales* | *Trichosporonaceae* | *Trichosporum multisporum* | *Trichosporon multisporum* | Dung, frass (not bat) | Spain | 1 | Vanderwolf et al. (2013a) |
| *Tritirachiomycetes* | *Tritirachiales* | *Tritirachiaceae* | *Tritirachium cinnamomeum* | *Tritirachium cinnamomeum* | Insects | Netherlands | 1 | Vanderwolf et al. (2013a) |
| *Tritirachiomycetes* | *Tritirachiales* | *Tritirachiaceae* | *Tritirachium brasiliense* | *Tritirachium brasiliense* | Sediment | Brazil | 1 | Alves et al. (2022) |
| *Tritirachiomycetes* | *Tritirachiales* | *Tritirachiaceae* | *Tritirachium dependens* | *Tritirachium dependens* | Wood | Romania | 1 | Vanderwolf et al. (2013a) |
| *Tritirachiomycetes* | *Tritirachiales* | *Tritirachiaceae* | *Tritirachium isariae* | *Tritirachium isariae* | Other, unreported | UK | 1 | Vanderwolf et al. (2013a) |
| *Tritirachiomycetes* | *Tritirachiales* | *Tritirachiaceae* | *Tritirachium oryzae* | *Tritirachium oryzae* | Air, other | Poland | 1 | Vanderwolf et al. (2013a) |
| *Tritirachiomycetes* | *Tritirachiales* | *Tritirachiaceae* | *Tritirachium* sp. | *Tritirachium* sp. | Air, wood | Czech Republic, France | 2 | Vanderwolf et al. (2013a) |
| *Ustilaginomycetes* | *Ustilaginales* | *Ustilaginaceae* | *Moesziomyces antarcticus* | *Moesziomyces antarcticus* | Unreported | Unknow |  | Zhang et al. (2021) |
| *Ustilaginomycetes* | *Ustilaginales* | *Ustilaginaceae* | *Moesziomyces antarcticus* | *Pseudozyma antarctica* | Air | Spain | 1 | Vanderwolf et al. (2013a) |
| *Ustilaginomycetes* | *Ustilaginales* | *Ustilaginaceae* | Pseudozyma sp. | *Pseudozyma* sp. | Unreported | Canada | 1 | Vanderwolf et al. (2013a) |
| *Ustilaginomycetes* | *Ustilaginales* | *Ustilaginaceae* | *Ustilago* sp. | *Ustilago* sp. | Air | Antarctica, Spain | 2 | Vanderwolf et al. (2013a); Connell and Staudigel (2013) |
| *Ustilaginomycetes* | *Ustilaginales* | *Ustilaginaceae* | *Ustilago tritici* | *Ustilago tritici* | Unreported | Antarctica | 1 | Connell and Staudigel (2013); Zhang et al. (2021) |
| *Wallemiomycetes* | *Wallemiales* | *Wallemiaceae* | *Wallemia mellicola* | *Wallemia mellicola* | Unreported | China | 1 | Zhang et al. 2021 |
| *Wallemiomycetes* | *Wallemiales* | *Wallemiaceae* | *Wallemia sebi* | *Wallemia sebi* | Air, earthworm casts | Czech Republic, Slovakia | 2 | Vanderwolf et al. (2013a) |
|  |  |  | Basidiomycetes unidentified | Basidiomycetes unidentified | Air, wood, sediment, soil, insects, bat guano, Air, other, cave wall | Belgium, Brazil, Canada, Czech Republic, Italy, Poland, South Africa, USA | 8 | Vanderwolf et al. (2013a) |
|  |  |  | Basidiomycota sp. | Basidiomycota sp. | Air | France | 1 | Martin-Sanchez et al. (2014) |
|  |  |  | Hymenomycetes unidentified | Hymenomycetes unidentified | Wood | UK | 1 | Vanderwolf et al. (2013a) |
| ***Chytridiomycota*** |  |  |  |  |  |  |  |  |
| *Chytridiomycetes* | *Cladochytriales* | *Cladochytriaceae* | *Cladochytrium tenue* | *Cladochytrium tenue* | Unreported | India | 1 | Vanderwolf et al. (2013a) |
| *Rhizophydiomycetes* | *Rhizophydiales* | *Incertae sedis* | *Batrachochytrium dendrobatidis* | *Batrachochytrium dendrobatidis* | Other | USA | 1 | Vanderwolf et al. (2013a) |
| *Rhizophydiomycetes* | *Rhizophydiales* | *Rhizophydiaceae* | *Rhizophydium* sp. | *Rhizophydium* sp. | Sediment, soil | Puerto Rico | 1 | Vanderwolf et al. (2013a) |
| ***Entomophthoromycota*** |  |  |  |  |  |  |  |  |
| *Entomophthoromycetes* | *Entomophthorales* | *Ancylistaceae* | *Conidiobolus coronatus* | *Conidiobolus coronatus* | Water | Germany | 1 | Burow et al. (2019) |
| *Entomophthoromycetes* | *Entomophthorales* | *Ancylistaceae* | *Conidiobolus* sp. | *Conidiobolus* sp. | Unreported | Italy | 1 | Vanderwolf et al. (2013a) |
| *Entomophthoromycetes* | *Entomophthorales* | *Entomophthoraceae* | *Entomophaga grylli* | *Entomophaga grylli* | Insect | Germany, Slovakia, Slovenia, | 3 | Vanderwolf et al. (2013a) |
| *Entomophthoromycetes* | *Entomophthorales* | *Entomophthoraceae* | *Entomophthora destruens* | *Conidiobolus destruens* | Insects | Czech Republic | 1 | Vanderwolf et al. (2013a) |
| *Entomophthoromycetes* | *Entomophthorales* | *Entomophthoraceae* | *Entomophthora destruens* | *Entomophthora destruens* | Insect | CzechA Republic | 1 | Vanderwolf et al. (2013a) |
| *Entomophthoromycetes* | *Entomophthorales* | *Incertae sedis* | *Entomophthorales unidentified* | *Entomophthorales unidentified* | Unreported | UK | 1 | Vanderwolf et al. (2013a) |
| ***Glomeromycota*** |  |  |  |  |  |  |  |  |
| *Glomeromycetes* | *Entrophosporales* | *Entrophosporaceae* | Entrophospora sp. | Entrophospora sp. | Unreported | China | 1 | Zhang et al. (2021) |
| ***Mucoromycota*** |  |  |  |  |  |  |  |  |
| *Kickxellomycetes* | *Kickxellales* | *Kickxellaceae* | *Coemansia aciculifera* | *Coemansia aciculifera* | Earthworm casts, air | Czech Republic, Slovakia | 2 | Vanderwolf et al. (2013a) |
| *Kickxellomycetes* | *Kickxellales* | *Kickxellaceae* | *Coemansia* sp. | *Coemansia* sp. | Unreported, sediment, soil | Czech Republic, USA | 2 | Vanderwolf et al. (2013a) |
| *Lecanoromycetes* | *Caliciales* | *Caliciaceae* | *Metatrichia vesparia* | *Mucor pyriformis* | Bat guano | Bahamas | 1 | Vanderwolf et al. (2013a) |
| *Leotiomycetes* | *Helotiales* | *Pleuroascaceae* | *Entimomentora hyalina* | *Entimomentora hyalina* | Unreported | Eastern Canada | 1 | Vanderwolf et al. (2013a) |
| *Mortierellomycetes* | *Mortierellales* | *Mortierellaceae* | *Entomortierella jenkinii* | *Mortierella jenkinii* | Bat | USA | 1 | Zhang et al. (2014) |
| *Mortierellomycetes* | *Mortierellales* | *Mortierellaceae* | *Entomortierella parvispora* | *Entomortierella parvispora* | Precipitate, sediment, rock, wood | Germany, Poland, USA | 3 | Burow et al. (2019); Held et al. (2020); Zhang et al. (2021) |
| *Mortierellomycetes* | *Mortierellales* | *Mortierellaceae* | *Entomortierella parvispora* | *Mortierella parvispora* | Slate precipitate, wood | Germany, USA | 2 | Burow et al. (2019); Held et al. (2020) |
| *Mortierellomycetes* | *Mortierellales* | *Mortierellaceae* | *Gamsiella stylospora* | *Mortierella stylospora* | Bat | USA | 1 | Zhang et al. (2014) |
| *Mortierellomycetes* | *Mortierellales* | *Mortierellaceae* | *Gryganskiella fimbricystis* | *Gryganskiella fimbricystis* | Soil, sediment | Canada, USA | 2 | Out et al. (2016) |
| *Mortierellomycetes* | *Mortierellales* | *Mortierellaceae* | *Linnemannia amoeboidea* | *Mortierella amoeboidea* | Sediment | USA | 1 | Zhang et al. (2014) |
| *Mortierellomycetes* | *Mortierellales* | *Mortierellaceae* | *Linnemannia elongata* | *Mortierella elongata* | Insects | Belgium | 1 | Vanderwolf et al. (2013a) |
| *Mortierellomycetes* | *Mortierellales* | *Mortierellaceae* | *Linnemannia exigua* | *Linnemannia exigua* | insect, sediment | Belgium, USA | 2 | Vanderwolf et al. (2013a) |
| *Mortierellomycetes* | *Mortierellales* | *Mortierellaceae* | *Linnemannia exigua* | *Mortierella* cf. *exigua* | Insects | Belgium | 1 | Vanderwolf et al. (2013a) |
| *Mortierellomycetes* | *Mortierellales* | *Mortierellaceae* | *Linnemannia gamsii* | *Linnemannia gamsii* | Insect, sediment | Belgium, China, Czech Republic, Italy, USA | 5 | Vanderwolf et al. (2013a); Zhang et al. (2014) |
| *Mortierellomycetes* | *Mortierellales* | *Mortierellaceae* | *Linnemannia gamsii* | *Mortierella* cf. *gamsii* | Wood | USA | 1 | Held et al. (2020) |
| *Mortierellomycetes* | *Mortierellales* | *Mortierellaceae* | *Linnemannia gamsii* | *Mortierella gamsii* | Air, insects | Belgium, Czech Republic, Italy | 3 | Vanderwolf et al. (2013a) |
| *Mortierellomycetes* | *Mortierellales* | *Mortierellaceae* | *Linnemannia hyalina* | *Linnemannia hyalina* | Air, soil, cave wall, sediment, environmental swab | Canada, China, Czech Republic, Poland, Slovakia, UK, USA | 7 | Vanderwolf et al. (2013a); Zhang et al. (2014) |
| *Mortierellomycetes* | *Mortierellales* | *Mortierellaceae* | *Linnemannia hyalina* | *Mortierella hyalina* | Air, other, cave wall, sediment, soil, wood | Czech Republic, UK, USA | 3 | Vanderwolf et al. (2013a); Held et al. (2020) |
| *Mortierellomycetes* | *Mortierellales* | *Mortierellaceae* | *Linnemannia nantahalensis* | *Mortierella nantahalensis* | Insects | Belgium | 1 | Vanderwolf et al. (2013a) |
| *Mortierellomycetes* | *Mortierellales* | *Mortierellaceae* | *Lunasporangiospora chienii* | *Mortierella Chienii* | Unreported | Unknow |  | Zhang et al. (2021) |
| *Mortierellomycetes* | *Mortierellales* | *Mortierellaceae* | *Lunasporangiospora chienii* | *Mortierella umbellata* | Cave wall | USA | 1 | Vanderwolf et al. (2013a) |
| *Mortierellomycetes* | *Mortierellales* | *Mortierellaceae* | *Lunasporangiospora selenospora* | *Mortierella sclerotiella* | Soil | Canada | 1 | Out et al. (2016) |
| *Mortierellomycetes* | *Mortierellales* | *Mortierellaceae* | *Lunasporangiospora selenospora* | *Mortierella selenospora* | Ssediment | China | 1 | Man et al. (2015) |
| *Mortierellomycetes* | *Mortierellales* | *Mortierellaceae* | *Mortierella alliacea* | *Mortierella alliacea* | Insects | Belgium | 1 | Vanderwolf et al. (2013a) |
| *Mortierellomycetes* | *Mortierellales* | *Mortierellaceae* | *Mortierella alpina* | *Mortierella alpina* | Air, sediment, soil, wood, insects, other, cave wall, soil-sediment, animal bone | Belgium, China, Czech Republic, France, Italy, Korea, Poland, Russia, Spain, USA | 10 | Vanderwolf et al. (2013a); Man (2017) |
| *Mortierellomycetes* | *Mortierellales* | *Mortierellaceae* | *Mortierella antarctica* | *Mortierella antarctica* | Soil | USA | 1 | Zhang et al. 2014 |
| *Mortierellomycetes* | *Mortierellales* | *Mortierellaceae* | *Mortierella bainieri* | *Mortierella bainieri* | Insects | Belgium | 1 | Vanderwolf et al. (2013a) |
| *Mortierellomycetes* | *Mortierellales* | *Mortierellaceae* | *Mortierella basiparvispora* | *Mortierella basiparvispora* | Wood | USA | 1 | Held et al. (2020) |
| *Mortierellomycetes* | *Mortierellales* | *Mortierellaceae* | *Mortierella histoplasmatoides* | *Mortierella histoplasmatoides* | Air, sediment | China, France, USA | 3 | Zhang et al. (2014) |
| *Mortierellomycetes* | *Mortierellales* | *Mortierellaceae* | *Mortierella hypsicladia* | *Mortierella hypsicladia* | Bat guano | China, Japan | 2 | Zhang et al. (2021) |
| *Mortierellomycetes* | *Mortierellales* | *Mortierellaceae* | *Mortierella indohii* | *Mortierella indohii* | Bat guano, unreported | China | 1 | Zhang et al. (2017) |
| *Mortierellomycetes* | *Mortierellales* | *Mortierellaceae* | *Mortierella multispora* | *Mortierella multispora* | Bat carcasses | China | 1 | Karunarathna et al. (2020) |
| *Mortierellomycetes* | *Mortierellales* | *Mortierellaceae* | *Mortierella oligospora* | *Mortierella oligospora* | Sediment, soil | Russia | 1 | Vanderwolf et al. (2013a) |
| *Mortierellomycetes* | *Mortierellales* | *Mortierellaceae* | *Mortierella polycephala* | *Mortierella polycephala* | Insects, soil, sediment | Belgium, Canada, China, Italy, USA | 5 | Vanderwolf et al. (2013a) |
| *Mortierellomycetes* | *Mortierellales* | *Mortierellaceae* | *Mortierella polygonia* | *Mortierella polygonia* | Soil | Canada | 1 | Out et al. (2016) |
| *Mortierellomycetes* | *Mortierellales* | *Mortierellaceae* | *Mortierella pulchella* | *Mortierella pulchella* | Insects, wood | Belgium, USA | 2 | Vanderwolf et al. (2013a); Held et al. (2020) |
| *Mortierellomycetes* | *Mortierellales* | *Mortierellaceae* | *Mortierella reticulata* | *Mortierella reticulata* | Unreported | China | 1 | Zhang et al. (2021) |
| *Mortierellomycetes* | *Mortierellales* | *Mortierellaceae* | *Mortierella rhinolophicola* | *Mortierella rhinolophicola* | Bat carcasses, bat guano | China | 1 | Karunarathna et al. (2020) |
| *Mortierellomycetes* | *Mortierellales* | *Mortierellaceae* | *Mortierella rostafinskii* | *Mortierella rostafinskii* | Soil | USA | 1 | Vanderwolf et al. (2013a) |
| *Mortierellomycetes* | *Mortierellales* | *Mortierellaceae* | *Mortierella sarnyensis* | *Mortierella sarnyensis* | Insect, sediment | Belgium, China, USA | 3 | Vanderwolf et al. (2013a) |
| *Mortierellomycetes* | *Mortierellales* | *Mortierellaceae* | *Mortierella* spp. | *Mortierella* spp. | Air, soil-sediment, dead isopod bodies, soil, cave sediment, water, bat fur, insect, sediments, environmental swab, , cave wall, wood, air, other, bat guano, earthworm casts, slate precipitate | Bahamas, Belgium, Bulgaria, Canada, China, Czech Republic, France, Germany, Italy, Japan, Netherlands, Poland, Romania, Russia, Slovakia, Slovenia, Spain, Thailand, USA | 19 | Vanderwolf et al. (2013a); Burow et al. (2019) |
| *Mortierellomycetes* | *Mortierellales* | *Mortierellaceae* | *Mortierella yunnanensis* | *Mortierella yunnanensis* | Bat carcasses | China | 1 | Karunarathna et al. (2020) |
| *Mortierellomycetes* | *Mortierellales* | *Mortierellaceae* | *Mortierella zonata* | *Mortierella zonata* | Unreported | China | 1 | Zhang et al. (2021) |
| *Mortierellomycetes* | *Mortierellales* | *Mortierellaceae* | Mortierellaceae sp. | Mortierellaceae sp. | Sediment | Canada, USA | 2 | Savković et al. (2025) |
| *Mortierellomycetes* | *Mortierellales* | *Mortierellaceae* | *Necromortierella dichotoma* | *Mortierella dichotoma* | Insects | Belgium | 1 | Vanderwolf et al. (2013a) |
| *Mortierellomycetes* | *Mortierellales* | *Mortierellaceae* | *Necromortierella dichotoma* | *Necromortierella dichotoma* | insect, sediment | Belgium, China, USA | 3 | Vanderwolf et al. (2013a); Zhang et al. (2014) |
| *Mortierellomycetes* | *Mortierellales* | *Mortierellaceae* | *Podila clonocystis* | *Podila clonocystis* | Soil | China, USA | 2 | Zhang et al. (2021) |
| *Mortierellomycetes* | *Mortierellales* | *Mortierellaceae* | *Podila epicladia* | *Mortierella epicladia* | Insects | Belgium, China | 2 | Vanderwolf et al. (2013a) |
| *Mortierellomycetes* | *Mortierellales* | *Mortierellaceae* | *Podila horticola* | *Mortierella horticola* | Unreported | China | 1 | Zhang et al. (2017) |
| *Mortierellomycetes* | *Mortierellales* | *Mortierellaceae* | *Podila humilis* | *Mortierella humilis* | Air, sediment, soil | Czech Republic, Spain | 2 | Vanderwolf et al. (2013a) |
| *Mortierellomycetes* | *Mortierellales* | *Mortierellaceae* | *Podila minutissima* | *Podila minutissima* | soil-sediment | China, Slovakia | 2 | Zhang et al. (2017) |
| *Mortierellomycetes* | *Mortierellales* | *Mortierellaceae* | *Podila verticillata* | *Mortierella verticillata* | Insect | Belgium, Brazil, China | 3 | Vanderwolf et al. (2013a) |
| *Mucoromycetes* | *Calcarisporiellales* | *Calcarisporiellaceae* | *Calcarisporiella sp.* | *Calcarisporiella sp.* | Unreported | France | 1 | Vanderwolf et al. (2013a) |
| *Mucoromycetes* | *Mucorales* | *Choanephoraceae* | *Choanephora cucurbitarum* | *Choanephora cucurbitarum* | Soil-sediment | Russia | 1 | Vanderwolf et al. (2013a) |
| *Mucoromycetes* | *Mucorales* | *Choanephoraceae* | Choanephora infundibulifera f. infundibulifera | Choanephora infundibulifera f. cucurbitarum | Soil-sediment | Russia | 1 | Vanderwolf et al. (2013a) |
| *Mucoromycetes* | *Mucorales* | *Cunninghamellaceae* | *Absidia caerulea* | *Absidia caerulea* | On wet bread | France | 1 | Zhang et al. (2021) |
| *Mucoromycetes* | *Mucorales* | *Cunninghamellaceae* | *Absidia caerulea* | *Absidia coerulea* | Insects | Belgium | 1 | Vanderwolf et al. (2013a) |
| *Mucoromycetes* | *Mucorales* | *Cunninghamellaceae* | *Absidia cylindrospora* | *Absidia cylindrospora* | Bat guano, soil-sediment, cave wall | Abkhazia, Bahamas, Belgium, Slovakia | 4 | Vanderwolf et al. (2013); Popkova and Mazina (2019) |
| *Mucoromycetes* | *Mucorales* | *Cunninghamellaceae* | *Absidia cylindrospora* var. *cylindrospora* | *Absidia cylindrospora* var. *cylindrospora* | Sediment, soil, bat guano | Bahamas, Slovakia | 2 | Vanderwolf et al. (2013a) |
| *Mucoromycetes* | *Mucorales* | *Cunninghamellaceae* | *Absidia glauca* | *Absidia glauca* | Air, bat guano, animal bone | Hungary, Poland, Slovakia | 3 | Vanderwolf et al. (2013a); Kokurewicz et al. (2016) |
| *Mucoromycetes* | *Mucorales* | *Cunninghamellaceae* | *Absidia nigra* | *Absidia cylindrospora* var. *nigra* | Sediment, soil | Slovakia | 1 | Vanderwolf et al. (2013a) |
| *Mucoromycetes* | *Mucorales* | *Cunninghamellaceae* | *Absidia repens* | *Absidia repens* | Insects | Spain | 1 | Vanderwolf et al. (2013a) |
| *Mucoromycetes* | *Mucorales* | *Cunninghamellaceae* | *Absidia sp.* | *Absidia sp.* | Insects, bat guano, sediment, soil, wood, soil-sediment | Abkhazia, France, India, Poland, Puerto Rico, Romania, USA, Venezuela | 8 | Vanderwolf et al. (2013a) |
| *Mucoromycetes* | *Mucorales* | *Cunninghamellaceae* | *Absidia Spainosa* | *Absidia Spainosa* | Sediment, soil | Romania, Switzerland | 2 | Vanderwolf et al. (2013a) |
| *Mucoromycetes* | *Mucorales* | *Cunninghamellaceae* | *Cunninghamella echinulata* | *Cunninghamella echinata* | Sediment, soil | Hungary, India | 2 | Vanderwolf et al. (2013a) |
| *Mucoromycetes* | *Mucorales* | *Cunninghamellaceae* | *Cunninghamella elegans* | *Cunninghamella bertholletiae* | Insects | Brazil | 1 | Vanderwolf et al. (2013a) |
| *Mucoromycetes* | *Mucorales* | *Cunninghamellaceae* | *Cunninghamella elegans* | *Cunninghamella elegans* | Air, bat guano, sediment, soil, wood, cave wall | Czech Republic, Hungary, India, Korea, Romania, Russia, Switzerland, Thailand, Bahamas | 9 | Vanderwolf et al. (2013a) |
| *Mucoromycetes* | *Mucorales* | *Cunninghamellaceae* | *Cunninghamella* spp. | *Cunninghamella* spp. | Insects, sediment, soil, bat guano, unreported, soil-sediment | Bahamas, China, India, Puerto Rico, Thailand, USA | 6 | Vanderwolf et al. (2013a) |
| *Mucoromycetes* | *Mucorales* | *Cunninghamellaceae* | *Gongronella* sp. | *Gongronella* sp. | Soil-sediment, | China, Thailand | 2 | Suetrong et al. (2023) |
| *Mucoromycetes* | *Mucorales* | *ichtheimiaceae* | Rhizomucor sp. | Rhizomucor sp. | Unreported | France | 1 | Pfendler et al. (2019) |
| *Mucoromycetes* | *Mucorales* | *Lichtheimiaceae* | *Lichtheimia blakesleeana* | *Absidia blakesleeana* | Sediment, soil, dung, frass (not bat) | Bahamas, Hungary | 2 | Vanderwolf et al. (2013a) |
| *Mucoromycetes* | *Mucorales* | *Lichtheimiaceae* | *Lichtheimia blakesleeana* | *Lichtheimia blakesleeana* | Dung, soil-sediment, frass (not bat) | Bahamas, Hungary | 2 | Vanderwolf et al. (2013a) |
| *Mucoromycetes* | *Mucorales* | *Lichtheimiaceae* | *Lichtheimia corymbifera* | *Absidia corymbifera* | Insects, bat guano, other, sediment, soil | China (Taiwan Province); Czech Republic, France, Hungary, India, USA | 6 | Vanderwolf et al. (2013a) |
| *Mucoromycetes* | *Mucorales* | *Lichtheimiaceae* | *Lichtheimia corymbifera* | *Lichtheimia corymbifera* | Bat guano, insect, soil-sediment | China (Taiwan Province); Czech Republic, France, Hungary, India, Poland, USA | 7 | Vanderwolf et al. (2013a) |
| *Mucoromycetes* | *Mucorales* | *Lichtheimiaceae* | *Lichtheimia ramosa* | *Lichtheimia ramosa* | Bat guano | France | 1 | Martin-Sanchez et al. (2014) |
| *Mucoromycetes* | *Mucorales* | *Lichtheimiaceae* | *Lichtheimia* sp. | *Lichtheimia* sp. | Organic litter, | Thailand | 1 | Suetrong et al. (2023) |
| *Mucoromycetes* | *Mucorales* | *Lichtheimiaceae* | *Rhizomucor pusillus* | *Rhizomucor pusillus* | Bat guano, wood, unreported, sediment, soil, other | China (Taiwan Province); Czech Republic, France, India, Kazakhstan | 5 | Vanderwolf et al. (2013a) |
| *Mucoromycetes* | *Mucorales* | *Mucoraceae* | *Actinomucor elegans* | *Actinomucor elegans* | Sediment, soil, bat guano | Bahamas, China, Hungary, Poland, Romania | 5 | Vanderwolf et al. (2013a) |
| *Mucoromycetes* | *Mucorales* | *Mucoraceae* | *Actinomucor* sp. | Actinomucor sp. | Unreported | China | 1 | Wainwright (1993) |
| *Mucoromycetes* | *Mucorales* | *Mucoraceae* | *Helicostylum pulchrum* | *Helicostylum pulchrum* | Sediment | China, Germany, USA | 3 | Zhang et al. (2014) |
| *Mucoromycetes* | *Mucorales* | *Mucoraceae* | *Helicostylum* sp. | *Helicostylum* sp. | Sediment, soil | Bahamas, USA | 2 | Vanderwolf et al. (2013a) |
| *Mucoromycetes* | *Mucorales* | *Mucoraceae* | *Mortierella gemmifera* | *Mortierella gemmifera* | Wood | USA | 1 | Held et al. (2020) |
| *Mucoromycetes* | *Mucorales* | *Mucoraceae* | *Mucor abundans* | *Mucor abundans* | Slate precipitate | Germany | 1 | Burow et al. (2019) |
| *Mucoromycetes* | *Mucorales* | *Mucoraceae* | *Mucor aligarensis* | *Mucor aligarensis* | Animal bone, soil, sediment | Bulgaria, Poland | 2 | Mitova et al. (2017); Dyląg et al. (2019) |
| *Mucoromycetes* | *Mucorales* | *Mucoraceae* | *Mucor bacilliformis* | *Mucor bacilliformis* | Insect, soil | Slovenia, Wisconsin | 2 | Vanderwolf et al. (2013a) |
| *Mucoromycetes* | *Mucorales* | *Mucoraceae* | *Mucor circinelloides* | *Mucor circinelloides* | Air, soil-sediment, dung, frass (not bat); bat guano, insect, Air, asymptomatic specimens of Proteus anguinus, bat guano, moldy ball of waste paper, wood, rock surface, soil | Bulgaria, China, Czech Republic, France, South Africa, Slovakia, Slovenia, Spain | 8 | Vanderwolf et al. (2013a); Jacobs et al. (2017); Mitova et al. (2017), this study |
| *Mucoromycetes* | *Mucorales* | *Mucoraceae* | *Mucor circinelloides* f. *circinelloides* | *Mucor circinelloides* f. *circinelloides* | Air, Sediment, soil, dung, frass (not bat); insects, bat guano, other | Czech Republic, Slovakia, Slovenia, Spain | 4 | Vanderwolf et al. (2013a) |
| *Mucoromycetes* | *Mucorales* | *Mucoraceae* | *Mucor corticola* | *Mucor corticola* | Insect, bat guano | Slovakia, Slovenia | 2 | Vanderwolf et al. (2013a) |
| *Mucoromycetes* | *Mucorales* | *Mucoraceae* | *Mucor corticola* | *Mucor hiemalis* f. *corticola* | Insects, bat guano | Slovakia, Slovenia | 2 | Vanderwolf et al. (2013a) |
| *Mucoromycetes* | *Mucorales* | *Mucoraceae* | *Mucor flavus* | *Mucor flavus* | Air, Insects, sediment, soil, slate precipitate, precipitate, soil-sediment | Belgium, China, Czech Republic, Germany, Poland, Slovenia, USA | 7 | Vanderwolf et al. (2013a); Kokurewicz et al. (2016); Burow et al. (2019) |
| *Mucoromycetes* | *Mucorales* | *Mucoraceae* | *Mucor fragilis* | *Mucor fragilis* | Air, sediment, bat bones, bat guano, rock surface, water | China, Poland | 2 | Vanderwolf et al. (2013a) |
| *Mucoromycetes* | *Mucorales* | *Mucoraceae* | *Mucor hiemalis* | *Mucor hiemalis* | Air, sediment, soil, unreported, cave wall, insects, soil-sediment, wood | Czech Republic, France, Italy, Poland, Russia, Slovakia, Slovenia | 7 | Vanderwolf et al. (2013a); Kokurewicz et al. (2016); Held et al. (2020) |
| *Mucoromycetes* | *Mucorales* | *Mucoraceae* | *Mucor hiemalis* f. *hiemalis* | *Mucor hiemalis* f. *hiemalis* | Air, sediment, soil, insects, bat guano, other | Czech Republic, France, Italy, Poland, Russia, Slovakia, Slovenia | 7 | Vanderwolf et al. (2013a); Karunarathna et al. (2020) |
| *Mucoromycetes* | *Mucorales* | *Mucoraceae* | *Mucor indicus* | *Mucor indicus* | Bat guano | China, France | 2 | Vanderwolf et al. (2013a) |
| *Mucoromycetes* | *Mucorales* | *Mucoraceae* | *Mucor irregularis* | *Mucor irregularis* | Unreported, sediment, soil, dung, frass (not bat); insects, bat guano, wood, rock surface | Belgium, China, France, Germany, Romania, Slovakia, USA, Venezuela | 8 | Vanderwolf et al. (2013a), |
| *Mucoromycetes* | *Mucorales* | *Mucoraceae* | *Mucor jansseni* | *Mucor circinelloides* f. *jansseni* | Other, rock surface, sediment, soil | China, Czech Republic | 2 | Vanderwolf et al. (2013a) |
| *Mucoromycetes* | *Mucorales* | *Mucoraceae* | *Mucor luteus* | *Mucor luteus* | Rock | China, Poland | 2 | Kokurewicz et al. (2016) |
| *Mucoromycetes* | *Mucorales* | *Mucoraceae* | *Mucor mucedo* | *Mucor mucedo* | Rock, soil-sediment, insect, wood | Belgium, France, Germany, Poland, Romania, Slovakia, Sweden, USA, Venezuela | 9 | Ogórek et al. (2013, 2014a); Vanderwolf et al. (2013a) |
| *Mucoromycetes* | *Mucorales* | *Mucoraceae* | *Mucor nederlandicus* | *Mucor subtilissimus* | Sediment, soil | Bahamas | 1 | Vanderwolf et al. (2013a) |
| *Mucoromycetes* | *Mucorales* | *Mucoraceae* | *Mucor phayaoensis* | *Mucor phayaoensis* | Water | China | 1 | This study |
| *Mucoromycetes* | *Mucorales* | *Mucoraceae* | *Mucor piriformis* | *Mucor piriformis* | Insect, dead bat fur, skin | Belgium, UK | 2 | Vanderwolf et al. (2013a) |
| *Mucoromycetes* | *Mucorales* | *Mucoraceae* | *Mucor plumbeus* | *Mucor plumbeus* | sediment, soil, insects, cave wall, soil-sediment, animal bone, bat guano, candle | China, Czech Republic, Hungary, Italy, Poland, Slovenia | 6 | Vanderwolf et al. (2013a) |
| *Mucoromycetes* | *Mucorales* | *Mucoraceae* | *Mucor racemosus* | *Mucor racemosus* | Air, insects, sediment, soil, dung, frass (not bat), other, cave wall, bat guano, candle, moldy ball of waste paper, rock surface, soil | Canada, China, Czech Republic, Hungary, Italy, Poland, Russia, Spain, USA | 9 | Vanderwolf et al. (2013a); Kokurewicz et al. (2016) |
| *Mucoromycetes* | *Mucorales* | *Mucoraceae* | *Mucor racemosus* | *Mucor racemosus* f. *racemosus* | Air, sediment, soil, bat guano | Czech Republic, Slovakia | 2 | Vanderwolf et al. (2013a) |
| *Mucoromycetes* | *Mucorales* | *Mucoraceae* | *Mucor racemosus* | *Mucor racemosus* f. *sphaerosporus* | Air, sediment, soil, bat guano, other | Poland, Slovakia, USA | 3 | Vanderwolf et al. (2013a) |
| *Mucoromycetes* | *Mucorales* | *Mucoraceae* | *Mucor ramosissimus* | *Mucor ramosissimus* | Air, sediment, soil, water, Air, soil-sediment | China, Slovakia | 2 | Vanderwolf et al. (2013a) |
| *Mucoromycetes* | *Mucorales* | *Mucoraceae* | *Mucor silvaticus* | *Mucor hiemalis f. silvaticus* | Air, bat guano, earthworm casts | Slovakia | 1 | Vanderwolf et al. (2013a) |
| *Mucoromycetes* | *Mucorales* | *Mucoraceae* | *Mucor silvaticus* | *Mucor silvaticus* | Air, bat guano, earthworm casts | Slovakia | 1 | Vanderwolf et al. (2013a) |
| *Mucoromycetes* | *Mucorales* | *Mucoraceae* | *Mucor* sp. | *Zygorhynchus* sp. | Unreported | Unknow |  | Wainwright (1993) |
| *Mucoromycetes* | *Mucorales* | *Mucoraceae* | *Mucor Spainosus* | *Mucor Spainosus* | Sediment, soil, wood | Italy, Romania | 2 | Vanderwolf et al. (2013a) |
| *Mucoromycetes* | *Mucorales* | *Mucoraceae* | Mucor spp. | Mucor spp. | Air, soil-sediment, Organic litter, skinrock, precipitate, bat guano, cave wall, wood, , insects, sediment, soil, unreported, dung, frass (not bat), slate precipitate | Algeria, Australia, Belgium, Brazil, Bulgaria, Canada, China, Czech Republic, France, Germany, Greece, India, Italy, Japan, Kazakhstan, Poland, Puerto Rico, Russia, Serbia, Slovakia, Slovenia, Spain, Switzerland, Thailand, UK, Ukraine, USA, Venezuela | 27 | Vanderwolf et al. (2013a); Taylor et al. (2013, 2014); Burow et al. (2019) |
| *Mucoromycetes* | *Mucorales* | *Mucoraceae* | *Mucor strictus* | *Mucor strictus* | Soil | Canada | 1 | Out et al. (2016) |
| *Mucoromycetes* | *Mucorales* | *Mucoraceae* | *Mucor troglophilus* | *Mucor troglophilus* | Insects | Slovenia | 1 | Vanderwolf et al. (2013a) |
| *Mucoromycetes* | *Mucorales* | *Mucoraceae* | *Thamnidium elegans* | *Thamnidium elegans* | Soil-sediment | Romania, Russia | 2 | Vanderwolf et al. (2013a, 2015) |
| *Mucoromycetes* | *Mucorales* | *Mucoraceae* | *Thamnidium* sp. | *Thamnidium* sp. | Unreported | Abkhazia, Canada | 2 | Popkova and Mazina (2019); Vanderwolf et al. (2019) |
| *Mucoromycetes* | *Mucorales* | *Phycomycetaceae* | *Phycomyces nitens* | *Phycomyces nitens* | Mammal dung | Spain | 1 | Savković et al. (2025) |
| *Mucoromycetes* | *Mucorales* | *Phycomycetaceae* | *Phycomyces* sp. | *Phycomyces* sp. | Insects, unreported | Italy, USA | 2 | Vanderwolf et al. (2013a) |
| *Mucoromycetes* | *Mucorales* | *Rhizopodaceae* | *Rhizopus arrhizus* | *Amylomyces rouxii* | Insects | Slovenia | 1 | Vanderwolf et al. (2013a) |
| *Mucoromycetes* | *Mucorales* | *Rhizopodaceae* | *Rhizopus arrhizus* | *Mucor rouxii* | Soil | China, Egypt | 2 | Elkhateeb et al. (2022) |
| *Mucoromycetes* | *Mucorales* | *Rhizopodaceae* | *Rhizopus arrhizus* | *Rhizopus arrhizus* | Air, bat carcasse, bat guano, rock surface, soil | Abkhazia, Brazil, China, France, Poland | 5 | Zhang et al. (2021); Cunha et al. (2020) |
| *Mucoromycetes* | *Mucorales* | *Rhizopodaceae* | *Rhizopus arrhizus* | *Rhizopus delemar* | Bat guano | Hungary | 1 | Vanderwolf et al. (2013a) |
| *Mucoromycetes* | *Mucorales* | *Rhizopodaceae* | *Rhizopus arrhizus* | *Rhizopus oryzae* | Air, water, sediment, soil, bat guano, cave wall, wood | China, Czech Republic, France, Hungary, Israel, Netherlands, Puerto Rico, Romania, Slovakia, USA | 10 | Vanderwolf et al. (2013a); Zhang et al. (2017) |
| *Mucoromycetes* | *Mucorales* | *Rhizopodaceae* | *Rhizopus microsporus* | *Rhizopus alpinus* | Sediment, soil | Italy | 1 | Vanderwolf et al. (2013a) |
| *Mucoromycetes* | *Mucorales* | *Rhizopodaceae* | *Rhizopus microsporus* | *Rhizopus microsporus* | Cave wall | France, Czech Republic | 2 | Vanderwolf et al. (2013a) |
| *Mucoromycetes* | *Mucorales* | *Rhizopodaceae* | Rhizopus spp. | Rhizopus spp. | Air, insects, unreported, bat guano, insects, sediment, soil | Australia, Brazil, China, India, Poland, Puerto Rico, Slovakia, Slovenia, Spain, UK, USA | 11 | Taylor et al. (2013); Vanderwolf et al. (2013a); Zhang et al. (2021) |
| *Mucoromycetes* | *Mucorales* | *Rhizopodaceae* | *Rhizopus stolonifer* | *Rhizopus stolonifer* | Air, sediment, soil | China, Czech Republic, Germany, Poland, Romania | 5 | Vanderwolf et al. (2013a); Kokurewicz et al. (2016); Zhang et al. (2021); Elkhateeb et al. (2022) |
| *Mucoromycetes* | *Mucorales* | *Rhizopodaceae* | *Rhizopus stolonifer* var. *stolonifer* | *Rhizopus stolonifer* var. *stolonifer* | Air, dung, frass (not bat); sediment, soil, bat guano, cave wall, other, wood | Czech Republic, Hungary, India, Italy, Poland, Puerto Rico, Romania, Slovakia, Spain, Venezuela | 10 | Vanderwolf et al. (2013a) |
| *Mucoromycetes* | *Mucorales* | *Syncephalastraceae* | *Circinella muscae* | *Circinella muscae* | Air, bat guano | Bahamas, Poland | 2 | Vanderwolf et al. (2013a) |
| *Mucoromycetes* | *Mucorales* | *Syncephalastraceae* | *Circinella simplex* | *Circinella simplex* | Bat guano | Hungary | 1 | Vanderwolf et al. (2013a) |
| *Mucoromycetes* | *Mucorales* | *Syncephalastraceae* | Circinella spp. | Circinella spp. | Air, Organic litter | Slovakia, Thailand | 2 | Vanderwolf et al. (2013a) |
| *Mucoromycetes* | *Mucorales* | *Syncephalastraceae* | *Circinella umbellata* | *Circinella umbellata* | Bat guano, wood | Puerto Rico, Romania | 2 | Vanderwolf et al. (2013a) |
| *Mucoromycetes* | *Mucorales* | *Syncephalastraceae* | *Syncephalastrum racemosum* | *Syncephalastrum racemosum* | Bat bones, Sediment, soil, cave wall, air | China, Israel, Russia, South Africa | 4 | Vanderwolf et al. (2013a) |
| *Mucoromycetes* | *Mucorales* | *Syncephalastraceae* | *Syncephalastrum* sp. | *Syncephalastrum* sp. | Air | Australia | 1 | Vanderwolf et al. (2013a) |
| *Mucoromycetes* | *Mucorales* | *Syncephalastraceae* | *Thamnostylum piriforme* | *Thamnostylum piriforme* | bat guano | Bahamas | 1 | Vanderwolf et al. (2013a) |
| *Umbelopsidomycetes* | *Umbelopsidales* | *Umbelopsidaceae* | *Umbelopsis angularis* | *Umbelopsis angularis* | water | Germany | 1 | Burow et al. (2019); Zhang et al. (2021) |
| *Umbelopsidomycetes* | *Umbelopsidales* | *Umbelopsidaceae* | *Umbelopsis dimorpha* | *Umbelopsis dimorpha* | Unreported | China | 1 | Zhang et al. (2021) |
| *Umbelopsidomycetes* | *Umbelopsidales* | *Umbelopsidaceae* | *Umbelopsis isabellina* | *Umbelopsis isabellina* | Air, insect, cave wall, wood, sediment | China, Czech Republic, Slovenia, USA | 4 | Vanderwolf et al. (2013a); Zhang et al. (2021) |
| *Umbelopsidomycetes* | *Umbelopsidales* | *Umbelopsidaceae* | *Umbelopsis ramanniana* | *Umbelopsis ramanniana* | Cave wall | Czech Republic, USA | 2 | Vanderwolf et al. (2013a); Zhang et al. (2014) |
| *Umbelopsidomycetes* | *Umbelopsidales* | *Umbelopsidaceae* | *Umbelopsis* sp. | *Umbelopsis* sp. | Organic litter | Thailand | 1 | Suetrong et al. (2023) |
| ***Zoopagomycota*** |  |  |  |  |  |  |  |  |
| *Zoopagomycetes* | *Zoopagales* | *Helicocephalidaceae* | *Rhopalomyces elegans* | *Rhopalomyces elegans* | Sediment, soil | Russia | 1 | Vanderwolf et al. (2013a) |
| *Zoopagomycetes* | *Zoopagales* | *Piptocephalidaceae* | *Syncephalis* sp. | *Syncephalis* sp. | Sediment, soil | India | 1 | Vanderwolf et al. (2013a) |
| *Incertae sedis* | *Incertae sedis* | *Incertae sedis* | Rhizomorpha spp. | *Rhizomorpha* spp. | Wood, cave wall | Germany, USA | 2 | Vanderwolf et al. (2013a) |
|  |  |  |  | Mycelia sterilia | insects, sediment, soil, wood, bat guano, cave wall, air, water | Bahamas, Brazil, Canada, China, Czech Republic, France, Greece, India, Israel, Italy, Korea, Poland, Puerto Rico, Russia, Slovakia, Spain, UK, Ukraine, USA | 19 | Vanderwolf et al. (2013a) |
|  |  |  |  | Paranomuraea carnea | Air, soil-sediment, bat guano, earthworm casts | Slovakia | 1 | Vanderwolf et al. (2013a) |
|  |  |  | Fungal endophyte | Fungal endophyte | Unreported | Thailand | 1 | Suetrong et al. (2023) |
|  |  |  |  | Non-sporulating black colonies | Air | Poland | 1 | Kokurewicz et al. (2016) |
|  |  |  |  | Non-sporulating white colonies | Air | Poland | 1 | Kokurewicz et al. (2016) |
| ***Protozoa*** |  |  |  |  |  |  |  |  |
| ***Amoebozoa*** |  |  |  |  |  |  |  |  |
| *Centramoebida* |  | *Acanthamoebidae* | *Acanthamoeba* sp. | *Acanthamoeba* sp. | Air | Slovenia | 1 | Vanderwolf et al. (2013a) |
| *Dictyostelea* | *Dictyostelida* | *Dictyosteliaceae* | *Cavenderia aureostipes* | *Dictyostelium aureostipes* | Unreported, sediment, soil, air | USA, Puerto Rico | 4 | Vanderwolf et al. (2013a) |
| *Dictyostelea* | *Dictyostelida* | *Dictyosteliaceae* | *Coremiostelium polycephalum* | *Dictyostelium polycephalum* | Sediment, soil, air | Bahamas, Slovenia | 2 | Vanderwolf et al. (2013a) |
| *Dictyostelea* | *Dictyostelida* | *Dictyosteliaceae* | *Dictyostelium citrinum* | *Dictyostelium citrinum* | Sediment, soil | Puerto Rico | 1 | Vanderwolf et al. (2013a) |
| *Dictyostelea* | *Dictyostelida* | *Dictyosteliaceae* | *Dictyostelium discoideum* | *Dictyostelium discoideum* | Air, unreported, sediment, soil | USA, Slovenia | 3 | Vanderwolf et al. (2013a) |
| *Dictyostelea* | *Dictyostelida* | *Dictyosteliaceae* | *Dictyostelium giganteum* | *Dictyostelium giganteum* | Sediment, soil, insects | USA, Puerto Rico | 3 | Vanderwolf et al. (2013a) |
| *Dictyostelea* | *Dictyostelida* | *Dictyosteliaceae* | *Dictyostelium leptosomum* | *Dictyostelium leptosomum* | Unreported | USA | 1 | Vanderwolf et al. (2013a) |
| *Dictyostelea* | *Dictyostelida* | *Dictyosteliaceae* | *Dictyostelium macrocephalum* | *Dictyostelium macrocephalum* | Sediment, soil | Puerto Rico | 1 | Vanderwolf et al. (2013a) |
| *Dictyostelea* | *Dictyostelida* | *Dictyosteliaceae* | *Dictyostelium mucoroides* | *Dictyostelium mucoroides* | Air, unreported, sediment, soil, insects | USA, Bahamas, Puerto Rico, Slovenia | 5 | Vanderwolf et al. (2013a) |
| *Dictyostelea* | *Dictyostelida* | *Dictyosteliaceae* | *Dictyostelium purpureum* | *Dictyostelium purpureum* | Sediment, soil, insects | USA, Bahamas, Puerto Rico | 4 | Vanderwolf et al. (2013a) |
| *Dictyostelea* | *Dictyostelida* | *Dictyosteliaceae* | *Dictyostelium rosarium* | *Dictyostelium rosarium* | Sediment, soil | USA | 2 | Vanderwolf et al. (2013a) |
| *Dictyostelea* | *Dictyostelida* | *Dictyosteliaceae* | *Dictyostelium* sp. | *Dictyostelium* sp. | Air, unreported | USA, Slovenia | 3 | Vanderwolf et al. (2013a) |
| *Dictyostelea* | *Dictyostelida* | *Dictyosteliaceae* | *Dictyostelium sphaerocephalum* | *Dictyostelium sphaerocephalum* | Sediment, soil, insects | USA | 3 | Vanderwolf et al. (2013a) |
| *Dictyostelea* | *Dictyostelida* | *Dictyosteliaceae* | *Hagiwaraea vinaceofusca* | *Dictyostelium vinaceofuscum* | Sediment, soil | Bahamas | 1 | Vanderwolf et al. (2013a) |
| *Dictyostelea* | *Dictyostelida* | *Dictyosteliaceae* | *Heterostelium candidum* | *Polysphondylium candidum* | Sediment, soil | USA | 1 | Vanderwolf et al. (2013a) |
| *Dictyostelea* | *Dictyostelida* | *Dictyosteliaceae* | *Heterostelium pallidum* | *Polysphondylium pallidum* | Unreported, sediment, soil | USA, Bahamas, Puerto Rico | 5 | Vanderwolf et al. (2013a) |
| *Dictyostelea* | *Dictyostelida* | *Dictyosteliaceae* | *Heterostelium tenuissimum* | *Polysphondylium tenuissimum* | Sediment, soil | USA, Bahamas | 2 | Vanderwolf et al. (2013a) |
| *Dictyostelea* | *Dictyostelida* | *Dictyosteliaceae* | *Polysphondylium* sp. | *Polysphondylium* sp. | Unreported | USA | 1 | Vanderwolf et al. (2013a) |
| *Dictyostelea* | *Dictyostelida* | *Dictyosteliaceae* | *Polysphondylium violaceum* | *Polysphondylium violaceum* | Unreported, sediment, soil, insects | USA, Bahamas, Puerto Rico | 5 | Vanderwolf et al. (2013a) |
| *Dictyostelea* | *Dictyostelida* | *Dictyosteliaceae* | *Raperostelium minutum* | *Dictyostelium minutum* | Unreported, sediment, soil | USA | 2 | Vanderwolf et al. (2013a) |
| *Dictyostelea* | *Dictyostelida* | *Dictyosteliaceae* | *Raperostelium tenue* | *Dictyostelium tenue* | Sediment, soil | Puerto Rico | 1 | Vanderwolf et al. (2013a) |
| *Dictyostelea* | *Dictyostelida* | *Dictyosteliaceae* | *Speleostelium caveatum* | *Dictyostelium caveatum* | Sediment, soil, guano | USA | 1 | Vanderwolf et al. (2013a) |
| *Heterolobosea* | *Schizopyrenida* | *Vahlkampfiidae* | *Vahlkampfia sp.* | *Vahlkampfia* sp. | Air | Slovenia | 1 | Vanderwolf et al. (2013a) |
| *Myxogastrea* | *Liceida* | *Reticulariidae* | *Lycogala flavofuscum* | *Lycogala flavofuscum* | Air | Slovenia | 1 | Vanderwolf et al. (2013a) |
| *Myxogastrea* | *Physarida* | *Physaridae* | *Nannengaella globulifera* | *Physarum globuliferum* | Air | Slovenia | 1 | Vanderwolf et al. (2013a) |
| *Myxogastrea* | *Physarida* | *Physaridae* | *Physarum album* | *Physarum album* | Wood | Switzerland | 1 | Vanderwolf et al. (2013a) |
| *Myxogastrea* | *Stemonitida* | *Amaurochaetidae* | *Comatricha nigra* | *Comatricha nigra* | Wood | Switzerland | 1 | Vanderwolf et al. (2013a) |
| *Myxogastrea* | *Stemonitida* | *Amaurochaetidae* | *Comatricha sp.* | *Comatricha sp.* | Sediment, soil | Puerto Rico | 1 | Vanderwolf et al. (2013a) |
| *Myxogastrea* | *Stemonitida* | *Stemonitidae* | *Stemonitis fusca* | *Stemonitis fusca* | Wood | Bosnia | 1 | Vanderwolf et al. (2013a) |
| *Myxogastrea* | *Stemonitida* | *Stemonitidae* | *Stemonitis herbatica* | *Stemonitis herbatica* | Wood | Puerto Rico | 1 | Vanderwolf et al. (2013a) |
| *Myxogastrea* | *Trichiales* | *Trichiaceae* | *Arcyria obvelata* | *Arcyria nutans* | Wood | France | 1 | Vanderwolf et al. (2013a) |
| *Myxogastrea* | *Trichiales* | *Trichiaceae* | *Arcyria* sp. | *Arcyria* sp. | Bat bones, wood | Puerto Rico | 1 | Vanderwolf et al. (2013a) |
| *Myxogastrea* | *Trichiales* | *Trichiaceae* | *Oligonema favogineum* | *Trichia favoginea* | Wood | UK | 1 | Vanderwolf et al. (2013a) |
| *Myxogastrea* | *Trichiales* | *Trichiaceae* | *Trichia scabra* | *Trichia scabra* | Air | Slovenia | 1 | Vanderwolf et al. (2013a) |
| *Myxogastrea* | *Trichiales* | *Trichiaceae* | *Trichia* sp. | *Trichia* sp. | Air | Spain | 1 | Vanderwolf et al. (2013a) |
| *Tubulinea* | *Euamoebida* | *Hartmannellidae* | *Hartmannella cantabrigiensis* | *Hartmannella cantabrigiensis* | Air | Slovenia | 1 | Vanderwolf et al. (2013a) |
| *Tubulinea* | *Euamoebida* | *Hartmannellidae* | *Hartmannella vermiformis* | *Hartmannella vermiformis* | Air | Slovenia | 1 | Vanderwolf et al. (2013a) |
| *Tubulinea* | *Hartmannellidae* | *Echinamoeba* | *Echinamoeba sp.* | *Echinamoeba sp.* | Air | Slovenia | 1 | Savković et al. (2025) |
|  | *Leptomyxida* | *Rhizamoebidae* | *Rhizamoeba neglecta* | *Rhizamoeba neglecta* | Air | Slovenia | 1 | Vanderwolf et al. (2013a) |
|  |  |  |  | Myxomycetes unidentified | Air, unreported | Spain , France | 2 | Vanderwolf et al. (2013a) |
| ***Choanozoa*** |  |  |  |  |  |  |  |  |
| *Ichthyosporea* | *Eccrinida* | *Eccriniidae* | *Enterobryus oxidi* | *Enterobryus oxidi* | Insects | USA | 1 | Vanderwolf et al. (2013a) |
| *Ichthyosporea* | *Eccrinida* | *Eccriniidae* | *Enterobryus* sp. | *Enterobryus* sp. | Insects | USA | 1 | Vanderwolf et al. (2013a) |
| ***Chromista*** |  |  |  |  |  |  |  |  |
| ***Oomycota*** |  |  |  |  |  |  |  |  |
| *Peronosporea* | *Albuginales* | *Albuginaceae* | *Albugo* sp. | *Albugo* sp. | Air | Spain | 1 | Vanderwolf et al. (2013a) |
| *Peronosporea* | *Leptomitales* | *Leptomitaceae* | *Apodachlya* sp. | *Apodachlya* sp. | Water | Puerto Rico | 1 | Vanderwolf et al. (2013a) |
| *Peronosporea* | *Leptomitales* | *Leptomitaceae* | *Leptomitus lacteus* | *Leptomitus lacteus* | Water | Germany | 1 | Vanderwolf et al. (2013a) |
| *Peronosporea* | *Peronosporales* | *Peronosporaceae* | *Peronospora conglomerata* | *Peronospora conglomerata* | Insects | Brazil | 1 | Vanderwolf et al. (2013a) |
| *Peronosporea* | *Peronosporales* | *Peronosporaceae* | *Peronospora* sp. | *Peronospora* sp. | Air | Spain | 1 | Vanderwolf et al. (2013a) |
| *Peronosporea* | *Peronosporales* | *Pythiaceae* | *Pythium salpingophorum* | *Pythium salpingophorum* | Insects | Slovenia | 1 | Vanderwolf et al. (2013a) |
| *Peronosporea* | *Peronosporales* | *Pythiaceae* | *Pythium* sp. | *Pythium* sp. | Unreported, sediment, soil, water, cave wall, air | USA, Mexico, UK, Italy , Czech Republic | 5 | Vanderwolf et al. (2013a) |
| *Peronosporea* | *Saprolegniales* | *Leptolegniaceae* | *Aphanomyces* sp. | *Aphanomyces* sp. | Water | UK | 1 | Vanderwolf et al. (2013a) |
| *Peronosporea* | *Saprolegniales* | *Saprolegniaceae* | *Achyla americana* | *Achyla americana* | Water | Puerto Rico | 1 | Vanderwolf et al. (2013a) |
| *Peronosporea* | *Saprolegniales* | *Saprolegniaceae* | *Achyla* sp. | *Achyla* sp. | Unknown | USA | 1 | Vanderwolf et al. (2013a) |
| *Peronosporea* | *Saprolegniales* | *Saprolegniaceae* | *Brevilegnia* sp. | *Brevilegnia* sp. | Water | Puerto Rico | 1 | Vanderwolf et al. (2013a) |
| *Peronosporea* | *Saprolegniales* | *Saprolegniaceae* | *Saprolegnia parasitica* | *Saprolegnia parasitica* | Water | Puerto Rico | 1 | Vanderwolf et al. (2013a) |
| *Peronosporea* | *Saprolegniales* | *Saprolegniaceae* | *Saprolegnia* sp. | *Saprolegnia* sp. | Water | Puerto Rico, Germany | 2 | Vanderwolf et al. (2013a) |
| *Peronosporea* | *Saprolegniales* | *Saprolegniaceae* | *Saprolegnia thuretii* | *Saprolegnia thuretii* | Insects | Spain | 1 | Vanderwolf et al. (2013a) |

**Supplementary** **Table 2**. List of fungal and fungus-like organisms reported in cave environments. NF-Number of locations taxa found
